# Comparative analysis of miRNA and mRNA abundance in determinate cucumber by high-throughput sequencing

**DOI:** 10.1371/journal.pone.0190691

**Published:** 2018-01-05

**Authors:** Chao Ma, Jingjing Yang, Qing Cheng, Aijun Mao, Jian Zhang, Shiping Wang, Yiqun Weng, Changlong Wen

**Affiliations:** 1 Department of Plant Science, School of Agriculture and Biology, Shanghai Jiao Tong University, Shanghai, China; 2 Center for Viticulture and Enology, School of Agriculture and Biology, Shanghai Jiao Tong University, Shanghai, China; 3 Beijing Vegetable Research Center (BVRC), Beijing Academy of Agricultural and Forestry Sciences, National Engineering Research Center for Vegetables, Beijing, China; 4 Beijing Key Laboratory of Vegetable Germplasms Improvement, Beijing, China; 5 College of Horticulture, China Agricultural University, Beijing, China; 6 Institute of Agro-food Science and Technology, Key Laboratory of Agro-products Processing Technology of Shandong, Shandong Academy of Agricultural Sciences, Jinan, People's Republic of China; 7 Horticulture Department, University of Wisconsin, Madison WI, United States of America; 8 USDA-ARS Vegetable Crops Research Unit, Madison, WI, United States of America; East Carolina University, UNITED STATES

## Abstract

Determinate cucumber is a type of short vines, fewer nodes, and terminal flowers, it is suitable for high-density planting and available harvesting in field cultivation, whereas the indeterminate cucumber is preferred to cultivate in greenhouses. However, many biotic or abiotic stresses could lead indeterminate cucumber to be determinate in greenhouse cultivation, which may decrease yield and fruit quality. Therefore, it is urgent and essential to investigate the key factors forming determinate and terminal flowering in cucumber. In this study, two close background inbred lines were selected and conducted the miRNA and mRNA high throughput sequencing. Interestingly, ethylene-associated miRNAs and mRNAs were intensively obtained, indicating that the plant hormone ethylene is a key factor impacting determinate and terminal flowering in cucumber. The ethylene metabolites analysis showed that significant higher ethylene was observed in determinate line than that in the indeterminate line. The RT-qPCR validation of ethylene related miRNAs Cas-miR172, Cas-miR396, and Cas-miR414 and their target mRNAs showed a significant negative correlation. These data suggested that ethylene-associated miRNAs might affect determinate and terminal flower phenotypes by regulating their target genes expression. This study not only provides a potential molecular mechanism for determinate formation in cucumber but also establishes a method to demonstrate important physiological processes through the comprehensive association of miRNA and mRNA high-throughput sequencing.

## Introduction

Most cucumber (*Cucumis sativus* L.) cultivars have indeterminate growth habits, enabling harvesting as long as possible, while farmers have to manage indeterminate vines; hence the indeterminate cucumber is difficult to cultivate in field conditions [1]. A few determinate cultivars have seven to ten nodes and possess short vines with terminal flowers, suitable to cultivation under field conditions. The characteristics of determinate cucumber are early flowering, easy to high-density planting, availability to mechanical harvesting, no need for pinching, and simplified management [1]. Determinate cucumbers are acceptable for pickle cultivation in open fields in US and EU regions, which are suitable for high-density cultivation and mechanical harvest. However, indeterminate cucumbers are prevalent in greenhouse cultivation, which are important for high yields in Asian and Europe. To date, several groups have attempted to fine mapping the genes that control determinate traits in cucumber [2,3]. Weng et al. (2010) reported that the *de* loci localized on chromosome 6 in the cucumber genome [4]. However, the determinate gene in cucumber was unclear so far. Many researchers have found that a few environmental factors could lead indeterminate cucumber to become determinate under greenhouse cultivation with lower temperature and decreasing light density. In this situation, plant height decreased and early terminal flowers developed on the top of shoots; this physiological determinate cucumber led to severe yield loss and decreased quality. To date, few groups have studied the molecular mechanism of the physiological determinate phenomenon in cucumber cultivation, despite an urgent need for clarification.

MicorRNAs (miRNA) are highly conserved in eukaryotes, which are found in both plants and animals and are a class of 19–24 nt noncoding small RNAs that regulate gene expression at the post-transcriptional level by degrading target mRNAs or mediating gene expression by repressing mRNA translation [5,6]. In plants, mature miRNAs are generated from primary miRNAs (pri-miRNAs) via two steps: cutting by Dicer-Like1, and loading of miRNA into the ARGONAUTE1 (AGO1) protein complex for regulating target mRNA [7]. In the years since the first plant miRNAs were reported in *Arabidopsis thaliana*, due to the development of high-throughput sequencing technology for this model plant, the latest release of the miRBase database (miRBase release 21) registered 28,645 hairpin pre-miRNAs and 35,828 mature miRNAs in 223 species (http://www.mirbase.org/). Many studies have shown that miRNAs are involved in regulating a wide range of plant resistance and developmental processes, including abiotic stress, biotic stress leaf development, plant nutrient homeostasis and vegetative phase change [8]. Many studies suggest that miRNAs play an important role in regulating flowering. For instance, the first function analysis of miRNA in regulation of flowering time was reported by Chen et al. in 2004 [9]. Plants overexpressing miR172 showed an early-flowering phenotype because *AP2* mRNA translation was repressed. Furthermore, overexpression of an At-miR156b precursor generated abnormal flowers in tomatoes by affecting meristem maintenance-related genes [10]. In addition, due to the flowering time was regulated by photoperiod, overexpressing miR159 caused delayed flowering via down-regulation of *AtMYB33* under short-day conditions [11]. With the development of high-throughput sequencing technology, more and more miRNA related to the flowering time and shoot apical dominance has been reported. It is recognized that a single gene or miRNA cannot explain the complex flowering process. Accumulating evidence has demonstrated that numerous miRNAs are involved in the flowering and gametophyte development process and have been identified by high-throughput sequencing. For example, 292 known miRNAs and 75 novel miRNAs were identified in *Oryza sativa*; 103 known miRNAs and more than half of the 75 novel miRNAs showed pollen or stage-specific expression [12]. In dicotyledonous plants, miRNAs were identified for flower-specific expression; more than 200 known miRNAs and 72 novel miRNAs were involved in grapevine flower development [13]. This previous research indicates that miRNAs play an important role in flower and shoot apical development.

In cucumber, small RNAs (sRNAs) were first reported in 2011; a total of 25 conserved miRNAs and 7 novel miRNAs were identified in leaves and phloem exudate [14], and 64 miRNAs were identified in cucumber leaves and roots in 2012 [15]. To fully understand cucumber miRNA, a mixed sample from leaves, stems, and roots was used to identify miRNA, and 110 miRNAs were reported [16]. Recent work focused on resistance to the cucumber virus; a CGMMV (Cucumber green mottle mosaic virus) inoculated cucumber plant was used to identify 8 novel and 23 known miRNAs that respond to CGMMV infection [17]. In this study, we investigated the miRNA profiles of cucumber flower in ‘G1208’ (inflorescence shoot apex determinate line) and ‘H1201’ (inflorescence shoot apex indeterminate line) using high-throughput sequencing [18]. Determination is one of the major limiting factors in cucumber growth and yield in greenhouse cultivation. To investigate the potential roles of miRNAs and their target genes in determinate traits, this study conducted high-throughput sequencing of the miRNA and mRNA of the inflorescence shoot apex for the determinate line ‘G1208’ and indeterminate cucumber line ‘H1201’. The high-throughput sequencing has been already applied in cucumber for exploring disease resistance, cold tolerance, fruit quality traits [19], but for this aim (determinacy) is for the first time. This work provides novel insights into the molecular mechanisms of forming determinate cucumber. In addition, this study provides useful information for revealing the regulation of determinate or indeterminate cucumbers and establishes a method to analyze the comprehensive association of miRNAs and mRNAs involved in determinate and early terminal flowering in plants.

## Materials and methods

### Plant grow conditions and sample preparation

Two cultivars of cucumber (*Cucumis sativus* var. *sativus* L., 2n = 2x = 14) lines ‘H1201’ and ‘G1208’ (determinate growth habit) were used in this study. ‘G1208’ is a determinate line, and the ‘H1201’ is an important commercial line which is the self-pollinated progeny of ‘G1208’. All plant material was grown in a greenhouse with 25°C/22°C (day/night) and 16/8 h (photoperiod) at Beijing Academy of Agriculture and Forestry Sciences. The shoot apical meristems were harvested from the two species at the third day of florescence. Samples were frozen immediately in liquid nitrogen and stored at −80°C until use. Ethylene production was measured according to the method described by Li [20].

### Small RNA and mRNA library construction and sequencing

An equimolar concentration of total RNA extracted from three biological replications of the shoot apical stem samples were used to construct RNA library of the two cultivars. For the small RNA libraries, the total RNA of those two cultivars flowers was extracted using the Sigma-Aldrich protocol. Samples were subjected to 15% denaturing polyacrylamide gel electrophoresis (PAGE). The 18–30 nt small RNA were separated and purified for sequencing. For the mRNA library, total RNA was isolated using a kit (Sigma-Aldrich). Finally, small RNA and mRNA samples were sent for sequencing using an Illumina HiSeqTM 2000 instrument (Biomarker, Beijing, China). Sequencing data set supporting the results of RNA sequence are available in the repository of BIGD (http://bigd.big.ac.cn/) with the accession number CRA000559.

### Identification of known and novel miRNAs

The Raw data were filtered in Perl script to delete low-quality reads, sequence adapters and other contaminants. The 16–32 nt clean reads were annotated in Rfam (Release 12.0) (http://www.sanger.ac.uk/software/Rfam) (S1 Fig) and compared to the GeneBank (http://www.ncbi.nlm.nih.gov/) to remove noncoding RNA (rRNA, tRNA, snRNA, snoRNA). The sequences were aligned against miRBase (Release 21) (http://www.mirbase.org/), and perfectly matched sequences were considered conserved cucumber miRNAs (S1 Table), those reads with non-perfect matches were considered variants of known miRNAs and unannotated reads were thought novel miRNA prediction. Prediction is based on the biological characteristics of miRNA precursors which have a landmark hairpin-stem-loop structure. All unannotated reads with a length of 16–32 nt were mapped to the cucumber genome (http://www.icugi.org/) to obtain the miRNA predicted precursor structure Novel cucumber miRNA were predicted using mireap (http://sourceforge.net/projects/mireap/) with parameter values according to the method of Jia [21], followed by secondary structure prediction using RNAfold software (http://mfold.rna.albany.edu/). The key criteria for miRNA prediction were according to a previous report [22]. The GO analysis and KEGG analysis were performed with software from the Gene Ontology Consortium (http://geneontology.org/) and Kyoto Encyclopedia of Genes and Genomes (http://www.genome.jp/kegg/) [23,24].

### Target gene prediction

The reverse-complemented miRNA sequences were used to compare the cucumber genome to predict the target sites (mismatch ≤ 3, gap ≤ 2). The sequence near the target site (approximately 1000 bp) was used for gene annotation using the cucumber genome website (http://www.icugi.org/), Softberry software (http://linux1.softberry.com/) and the UEA sRNA toolkit (http://srna-tools.cmp.uea.ac.uk/). Finally, gene-specific primers were designed to analyze gene abundance in the two cultivars (S1 Table). For the multicopy gene, only one pair of primers was designed.

### Processing of mRNA-seq data

The low-quality tags (tags with unknown nucleotides N), adaptor sequences and empty reads tags were filtered to obtain clean tags, which were mapped to the cucumber genome (http://www.icugi.org/cgi-bin/ICuGI/EST/index.cgi), and tags that mapped to multiple genes in the reference sequences were filtered. The tags that could not be aligned to the reference genes were aligned to the cucumber genome sequence (http://www.icugi.org/).

### Identification of differentially expressed genes

To compare differentially expressed genes between those RNA-seq results of ‘H1201’ and ‘G1208’, the numbers of raw clean tags in each library was normalized to the number of TPM (transcripts per million clean tags) to obtain normalized gene abundances. Genes were deemed significantly differentially expressed with a P-value < 0.05, false discovery rates (FDR) < 0.01 and a relative change threshold of 2-fold in sequence counts across libraries. For pathway enrichment analysis, we mapped all differentially expressed genes to terms in the KEGG and GO database [23,24].

### Analysis of cucumber miRNAs and target genes by qPCR

The miRNA and target gene real-time PCR assay were performed according to the method of a previous study [25, 26]. Briefly, DNase-digested RNA (1 μg) extracted from leaves or flowers were in a reverse transcription reaction (Verso cDNA Synthesis Kit, Thermo Scientific), and real-time PCR was performed according to a previous study [25]. Each reaction was repeated six biologic replicates. All primers were designed using Primer Express 3.0 (listed in S2 Table). The relative abundance of miRNAs and target genes were calculated by the 2^−ΔΔCT^ method [27] and normalized by U6 rRNA and β-actin as a reference gene, respectively.

### Metabolites analysis of ethylene in shoot apical meristems

The ethylene concentration in shoot apical meristems of determinate and indeterminate lines were analyzed, six biological replicates were performed. Tissues were enclosed in a 10 mL penicillin bottle and sealed with a rubber stopper. After incubation at 25°C for 16 h, 1 mL of head gas was withdrawn from each vial using a gas-tight syringe. All samples were injected into a gas chromatograph (GC-4CMPF/Chromatopac CR4A; Shimadzu, Kyoto, Japan) equipped with a flame ionization detector and an activated alumina column for the measurement of ethylene. The instrument was calibrated with an ethylene gas standard, and amount of ethylene released from shoot apical meristems per gram fresh weight and per hour was calculated.

## Results

### Assembly of small RNAs in determinate and indeterminate cucumbers

**Fig 1:**
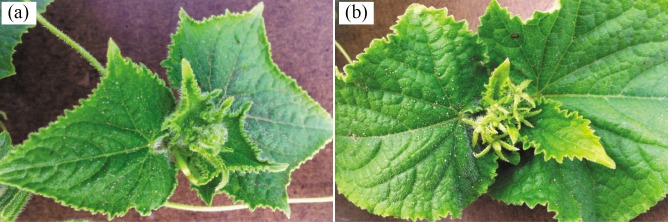
Sample for miRNA-mRNA high-throughput sequencing. (a), Normal shoot apex of ‘H1201’, indeterminate. (b), Terminal inflorescence in shoot apex of ‘G1208’, determinate.

To identify miRNA in the determinate line ‘G1208’ and indeterminate line ‘H1201’, the total RNA of the shoot apical stem was extracted to construct small RNA libraries for sequencing (Fig 1). In determinate and indeterminate libraries, a total of 13,798,612 (G1208) and 10,807,405 (H1201) reads were obtained, as shown in Table 1. After removing low-quality reads, adaptor reads, polyA/T/G/C/ reads and N% > 10% reads, 10,937,661 (79.27%) clean reads from ‘G1208’ and 8,135,923 (75.28%) clean reads from ‘H1201’ were remained. The percentage of clean reads and cluster reads were analyzed for size distribution; more than 70% of the small RNAs were 20–24 nt in length and the most common size was 24 nt (approximately 40%) (S1 Fig). This is consistent with the distribution patterns of small RNAs in many plants, such as mulberry and peanut [28, 21], but different from that of grapevine and wheat [22,29]. This implied a tissue-specific or plant-specific expression pattern for small RNAs in different species of plants. In our case, small RNAs in these two samples showed a similar distribution pattern, suggesting that these two cultivars had a similar background and were suitable for the single phenotypic analysis. The nucleotide bias in each position of the small RNA and the first nucleotide bias were checked in these two cucumber cultivars. The results showed no significant difference in each position’s nucleotide bias (S2 Fig). For the first nucleotide bias, most miRNAs (19–22 nt) tended to start with 5’-U, which is consistent with typical miRNA sequence patterns. However, we found a clear difference in the 20 nucleotide-length small RNAs between these two cultivars, in ‘G1208’, the miRNAs starting with 5’-U were the most, while in ‘H1201’, the miRNAs starting with 5’-G were the most (S3 Fig).

**Table 1:** Analysis of small RNA sequences from the flower buds of ‘G1208’ and ‘H1201’.

Type	G1208 (S1)		H1201 (S2)	
	Total reads	Percentage	Total reads	Percentage
Total reads number	13,798,612	100.00%	10,807,405	100.00%
Filter having "N" tages	6,268	0.05%	4,971	0.05%
Filter low quality tages	546,942	3.96%	406,297	3.76%
Adapter 3 insert null	0	0.00%	1	0.00%
5' adapter contaminants	5	0.00%	1	0.00%
Length < 16	1,623,783	11.77%	1,822,773	16.87%
Length > 32	281,434	2.04%	154,043	1.43%
Ploy A	402,519	2.92%	283,396	2.62%
Clean number	10,937,661	79.27%	8,135,923	75.28%
Unique number	4,673,282	33.87%	3,364,778	31.13%

### Identification of known and novel miRNAs

**Fig 2:**
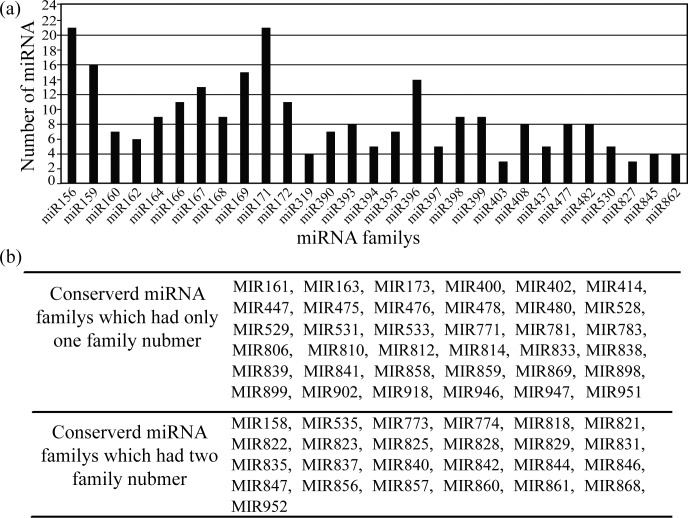
Conserved miRNAs and family numbers in cucumber. (a), The x-axis represents members in different miRNA families. The y-axis shows the conserved miRNA family identified in the two libraries, miR156 and miR171 had 21 members. (b), 37 and 25 miRNA families had only one or two members.

**Fig 3:**
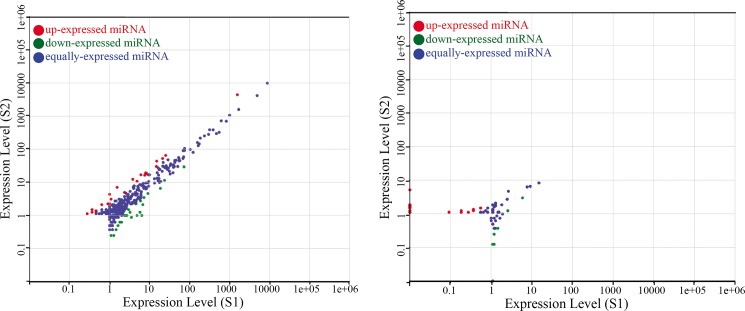
Scatter plot map of differentially expressed sRNA in the two lines. Conserved miRNA (left), novel miRNA (right). Red dots, ratio>2; blue dots, 0.5<ratio<2; green dots, ratio<0.5.

According to the sequence data, there were 250,887 total reads identified as putative miRNAs and 14,068 unique reads identified as miRNAs, which held 0.3% of total unique reads of small RNA (Table 2). To identify the conserved miRNAs in ‘G1208’ and ‘H1201’ cucumber cultivars, the small RNA sequences were aligned against miRNAs registered in the miRBase database (Release 21). After a BLASTN search (perfect match), a total of 537 conserved miRNAs belonging to 91 miRNA families were identified (S3 Table, Fig 2). In these two cucumber species, miR156 and miR171 were the largest families with 21 members, respectively, followed by miR159, miR166, miR167, miR169, miR172, miR319 and miR396, with more than 10 members. 36 families had only 1 member and 25 families had 2 members (Fig 2, S5 Table). According to the criterion for annotation of plant miRNA [30] and stabilized RNA secondary structure of the primary miRNA, this study detected a total of 581 novel miRNAs (S4 Table). The miRNA abundance comparison between ‘G1208’ and ‘H1201’ showed a weak correlation, suggesting that miRNA plays an important role in the process of determination and early terminal flowering (Fig 3).

**Table 2:** Numbers of reads for each small RNA classification identified in ‘G1208’ (S1).

Read Type	Unique reads	Percentage	Total reads	Percentage
miRNA	14,068	0.30%	250,887	2.29%
exon_antisense	69,127	1.48%	534,177	4.88%
exon_sense	141,816	3.03%	244,912	2.24%
intron_antisense	106,787	2.29%	197,787	1.81%
intron_sense	127,924	2.74%	211,982	1.94%
rRNA	190,698	4.08%	2,412,268	22.05%
repeat	6,542	0.14%	11,991	0.11%
scRNA	1	0.00%	1	0.00%
snRNA	898	0.02%	2,687	0.02%
snoRNA	872	0.02%	18,559	0.17%
tRNA	8,253	0.18%	132,360	1.21%
others	4,006,296	85.73%	6,920,050	63.27%
Total	4,673,282	100.00%	10,937,661	100.00%

### The expression pattern of miRNAs in determinate cucumber

**Fig 4:**
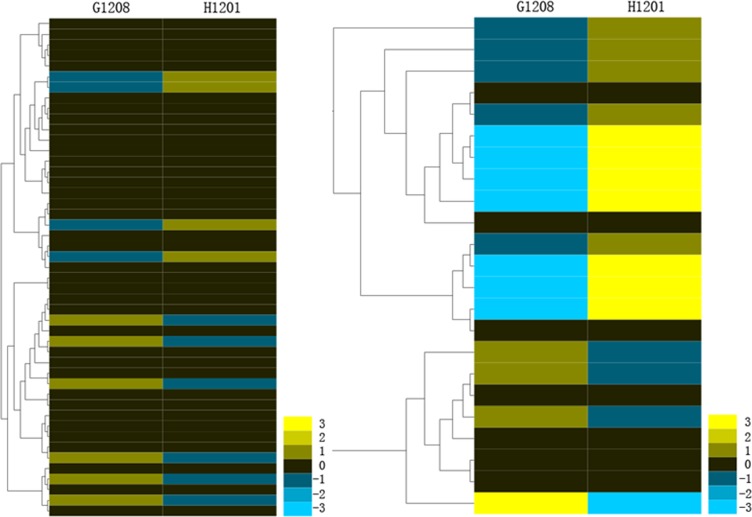
Differentially expressed miRNAs under two cucumber cultivars (S1, G1208; S2, H1201). Bar represents scale of relative miRNA expression (Log2 Fold change). The bottom right shows the color bar. The heatmap was generated by Heml 1.0. The left panel shows conserved miRNA, and the right panel shows novel miRNA in cucumber.

To determine whether miRNAs are related to determinate traits in cucumber, the miRNA abundance of ‘G1208’ and ‘H1201’ were used to generate a gene-expression heat-map. The selected criteria were as follows: the comparison has a fold change log2 scale value ≥1.0 or ≤-1.0 with q-value < 0.05. In all, 47 known miRNAs belonging to 8 miRNA families and 23 novel miRNAs were identified as differentially expressed miRNAs (Fig 4, S5 Table). The result showed that miR159, miR160, miR164, miR167, miR169, miR319, and miR398 showed an up-regulated pattern in ‘H1201’, and the expression of miR171, miR172, miR393, miR396, miR399 and miR414 showed higher abundance in ‘G1208’. For the novel miRNA, 10 showed up-regulated expression in ‘H1201’, and 13 increased in ‘G1208’ (Fig 4, S5 Table).

**Fig 5:**
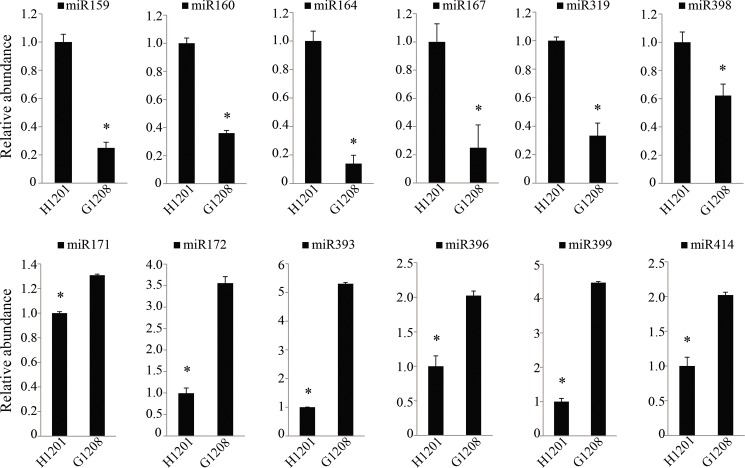
Quantitative expression analyses of several differentially expressed miRNAs in ‘H1201’ and ‘G1208’ cucumbers. The expression level in the ‘H1201’ line was set as 1. U6 rRNA was used as the reference gene for miRNA expression. The error bars correspond to ± SD (n = 6). Asterisks indicate significant differences (*P < 0.05, Student’s t-test).

To confirm the sRNA-seq results, RT-qPCR was performed to analyze miRNAs abundance in two cucumber lines. Twelve differentially expressed miRNAs were selected and validated. The results showed that the expression pattern of these miRNAs were similar to the small RNA sequence result: miR171, miR172, miR393, miR396, miR399 and miR414 were abundant in the determinate line ‘G1208’, and miR159, miR160, miR164, miR167, miR319 and miR398 were abundant in the indeterminate line ‘H1201’, indicating that they have crucial functions in controlling terminal flowering in cucumber (Fig 5).

### Target gene prediction and the GO and KEGG analysis

**Fig 6:**
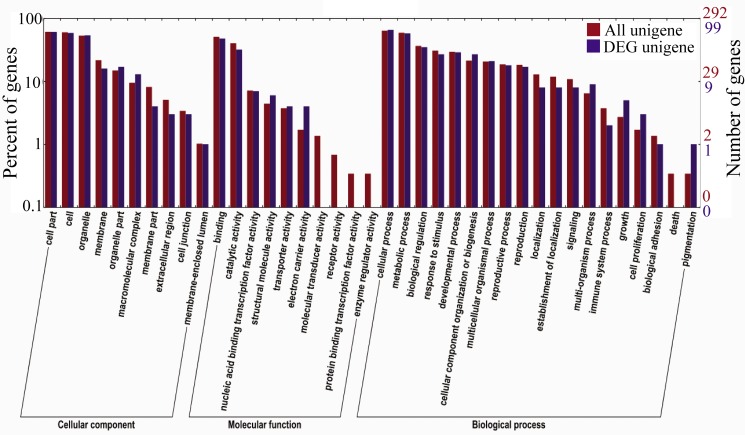
Significant gene ontology (GO) analysis of miRNA targets. The targeted genes of differentially expressed miRNAs in the ‘G1208’ and ‘H1201’ are analyzed in this figure. The left-hand scale is the percent of targeted gene numbers corresponding to GO terms. The right-hand scale is targeted gene numbers corresponding to GO terms. Red bar, all unigenes. Blue bar, differentially expressed genes’ unigenes.

Plant miRNAs are conserved, and most match target genes nearly perfectly. To further clarify the biological functions of the differentially expressed miRNAs in determinate and indeterminate cucumber lines, the target genes of all differentially expressed miRNAs were predicted by comparison to the cucumber genome. A total of 435 target genes (conserved miRNA targets 401 mRNAs, novel miRNA targets 34 mRNAs) for the 221 miRNAs (conserved miRNA 203, novel miRNA 18) were predicted (S1 and S6 Tables). For the 70 differentially expressed miRNAs, a total of 292 target genes were predicted and gene ontology (GO) analysis was performed using the Blast2go program (http://www.blast2go.com) (Fig 6). The GO analysis contained three ontologies: cellular components, molecular function and biological processes. The Blast2go result showed that these genes could be classified into 10 different cellular components, 10 different molecular functions and 19 different biological processes. The GO analysis results showed that 99 differentially expressed genes were in the 34 GO terms, including cellular components (10), molecular functions (6) and biological processes (18) (Fig 6).

To further assess the function-targeted genes, Kyoto encyclopedia of genes and genomes (KEGG) pathway analysis was performed to illuminate the biological interpretation of differentially expressed miRNA target genes. In total, 27 target genes including in 12 metabolic pathways showed significant enrichment (S7 Table). To fully understand the miRNA target gene functions, functional annotation was performed according to the cucumber genome website (details in Materials and Methods). Interestingly, many target genes of differentially expressed miRNAs played an important role in hormone signal transduction, especially in the auxin and ethylene pathway, such as miR164, miR17*2* and miR414. This indicated that ethylene might play crucial roles in controlling determinate and terminal flowering in cucumber.

### RNA-seq identified ethylene-related genes

According to the functional annotation results, several miRNA target genes were related to the ethylene pathway. The mRNA sequence was determined to confirm mRNA expression in determinate and indeterminate lines and the critical regulation and function of ethylene pathway genes in determinate and terminal flowering traits.

**Fig 7:**
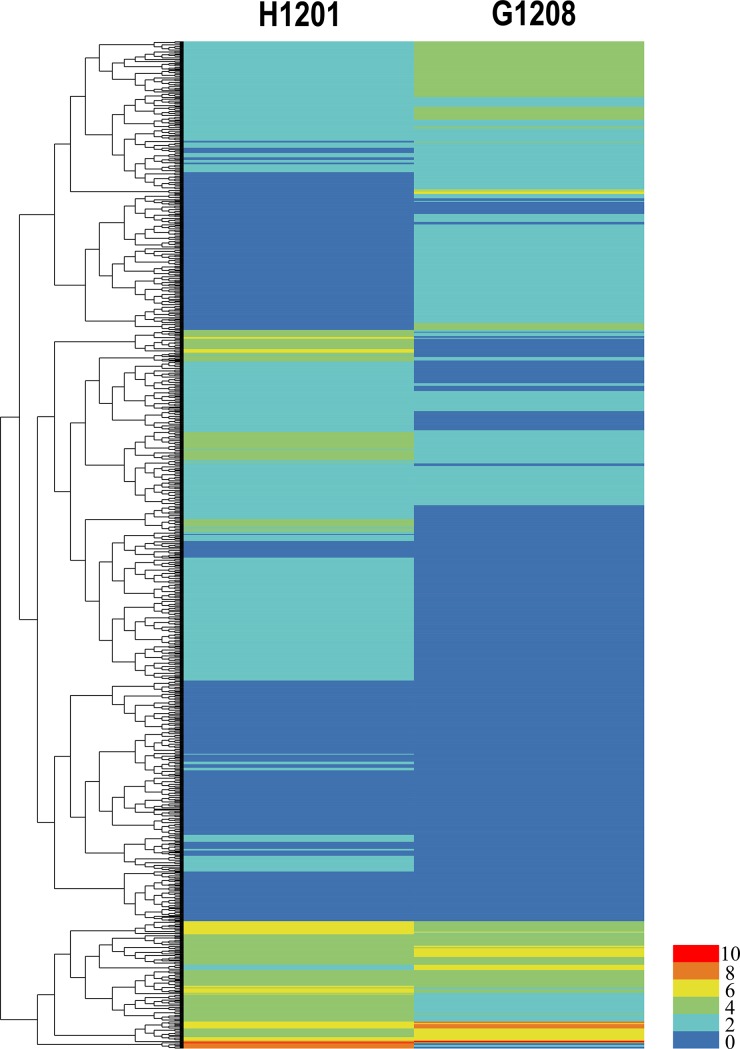
Differentially expressed mRNAs in ‘H1201’ and ‘G1208’. The bar represents the scale of relative mRNA expression (Log2 Fold change). The bottom right shows the color bar. The heatmap was generated by a Heml 1.0—Heatmap illustrator.

Two cDNA libraries were constructed from the total RNA of the ‘G1208’ and ‘H1201’ shoot apical stems. Samples were subjected to paired-end reading with the Illumina HiSeqTM 2000 platform, generating 114,130,948 and 120,045,774 paired-end raw reads in total, respectively (Table 3). The clean tags were mapped to the cucumber genome after deleting the low quality and adaptor sequences. Both libraries mapped at higher than 85% to the genome (Table 3). The clean reads from the two libraries were assembled (>200 nt reads) and compared to the cucumber EST library (http://www.icugi.org/cgi-bin/ICuGI/EST/index.cgi) version 3.0 (S8 and S9 Tables), and 613 new genes were found in these two samples (S10 Table). To compare changes in gene expression between ‘G1208’ and ‘H1201’, the gene expression levels were normalized to the RPKM (Reads Per Kilobase per Million mapped reads). Uniquely mapped reads were used to calculate the gene RPKM values. The differentially expressed genes were hierarchically clustered based on the log2 RPKM of the two samples, allowing us to observe the overall gene expression pattern. The blue bands indicate low-abundance genes, and the red bands indicate high-abundance genes (Fig 7). The comparative study of gene abundance in these two samples was filtered for corrected P values < 0.05 and log2 (fold change) values > 1. The number of differentially expressed genes (DEGs) among these comparisons displayed significant changes in expression. In total, 1,105 genes showed differential expression between the two lines (S11 Table).

**Table 3:** Analysis of mRNA sequences from the flower buds of ‘G1208’ and ‘H1201’ cultivars of cucumber.

BMK-ID	Total Reads	Mapped Reads	Uniq Mapped Reads	Multiple Map Reads
CKG1	39,279,454	34,539,803	34,110,293	429,510
CKG2	33,921,248	29,126,983	28,794,146	332,837
CKG3	40,930,246	37,875,872	37,207,978	667,894
Total	114,130,948 (100%)	101,542,658 (88.97%)	100,112,417 (87.72%)	1,430,241 (1.25%)
CKH1	35,021,944	30,953,674	30,597,401	356,273
CKH2	37,275,512	32,912,637	32,541,274	371,363
CKH3	47,748,318	43,807,826	43,136,330	671,496
Total	120,045,774 (100%)	107,674,137 (89.69%)	106,275,005 (88.53%)	1,399,132 (1.17%)

CKG1-3, the number of three biological replicates sequenced for ‘G1208’. CKH1-3, the number of three biological replicates sequenced for ‘H1201’.

**Fig 8:**
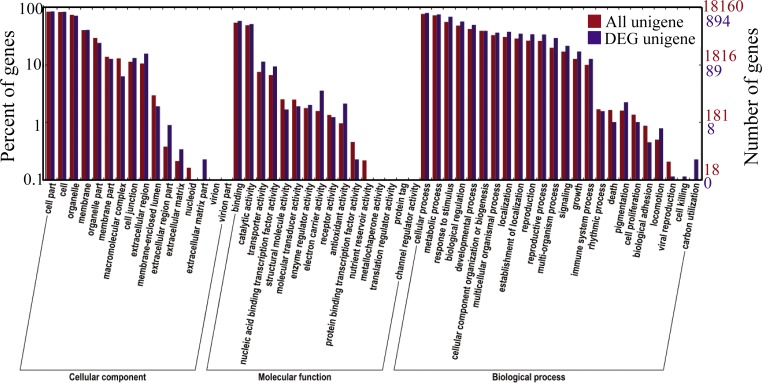
Enriched gene ontology (GO) terms. The figure provides a list of enriched GO terms within the biological process category identified from differentially expressed genes in ‘H1201’ and ‘G1208’ cucumber cultivars.

**Fig 9:**
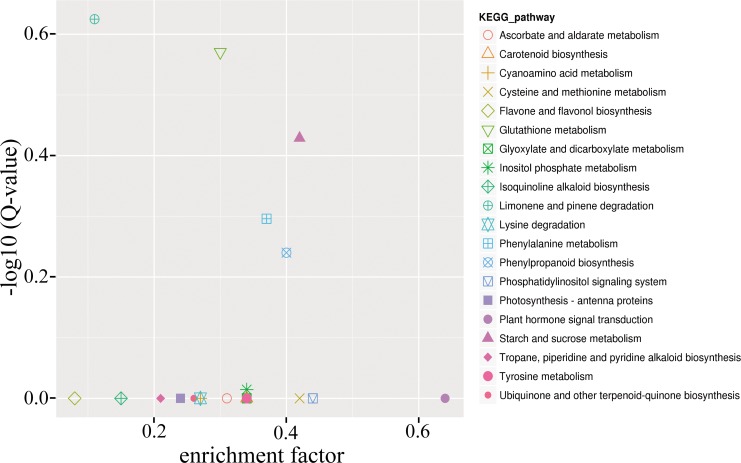
KEGG analysis of 20 most enriched pathways. The x-axis represents the enrichment factors that indicate differentially expressed gene numbers in these pathways. The y-axis represents q-values that indicate the significance of the rich factor.

To further understand the DEGs’ function, we conducted GO functional enrichment and KEGG pathway analyses. The result of DEGs GO analyses were very similar to the miRNA target gene GO analyses but include a wide range of categories compared to the miRNA target gene GO analyses, 16 different cellular components, 16 different molecular functions and 24 biological processes (Fig 8). This indicated that there were functional categories not regulated by miRNA in the determinate cucumber. KEGG pathway enrichment analysis revealed that they were assembled in 62 metabolic processes, particularly in plant hormone signal transduction, and 14 key differential expression genes were observed (S12 Table). The ratio of DEGs in different biological pathways is shown in Fig 9: most DEGs were included in the plant hormone signal transduction pathway (pink ball at lower right). In addition, the DEGs are mainly involved in the auxin and ethylene signal transduction pathway (S4 Fig). These results were consistent with miRNA-seq data, and validated the hypothesis that ethylene is important in determinate and terminal flowering in cucumber.

### Validation of the ethylene associated genes and miRNA targets in cucumber

**Fig 10:**
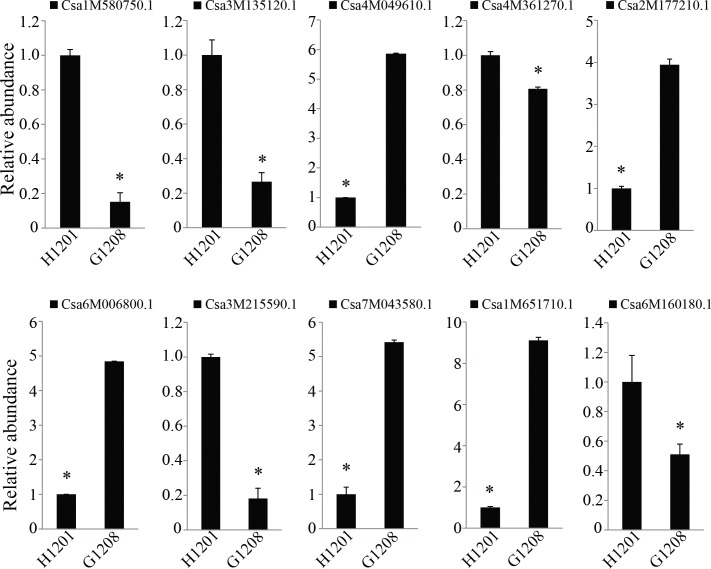
Quantitative expression analyses of ethylene-associated mRNAs in ‘H1201’ and ‘G1208’. The expression level in ‘H1201’ was set as 1. β-actin was used as the reference gene for relative gene expression analysis. The error bars correspond to ± SD (n = 6). Asterisks indicate significant differences (*P < 0.05, Student’s t-test).

**Fig 11:**
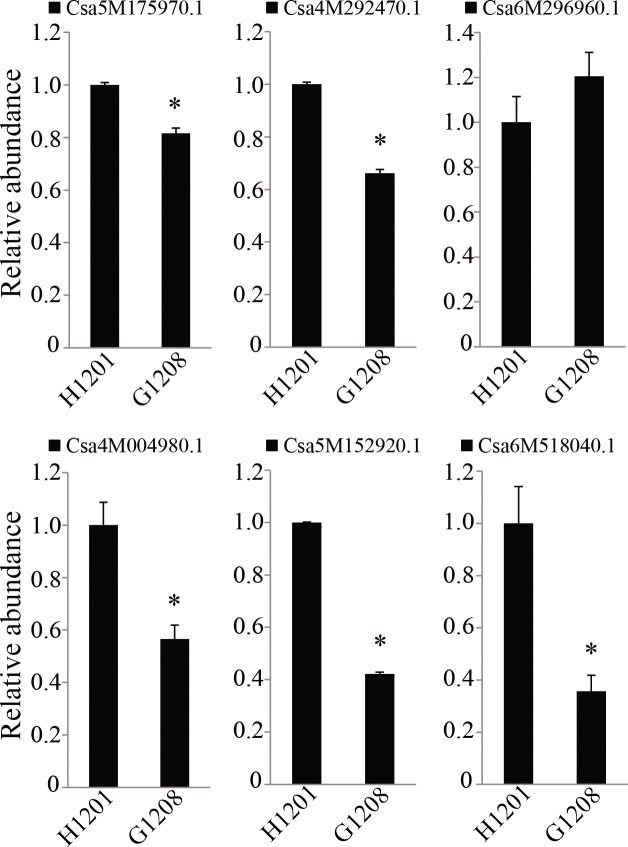
Quantitative expression analyses of ethylene-associated miRNA targets in ‘H1201’ and ‘G1208’. The expression level in ‘H1201’ was set as 1. β-actin was used as the reference gene for miRNA target genes relative expression analysis. The error bars correspond to ± SD (n = 6). Asterisks indicate significant differences (*P < 0.05, Student’s t-test).

qRT-PCR was performed to confirm the miRNA and mRNA sequencing results and clarify the relationship of differentially expressed miRNA and mRNA in the ethylene signal transduction pathway. In total, 10 ethylene-associated genes and 6 miRNA target genes were confirmed by RT-qPCR. *Csa4M049610*.*1*, *Csa2M177210*.*1*, *Csa6M006800*, *Csa7M043580*.*1*, and *Csa1M651710*.*1* showed higher expression in the determinate line ‘G1208’, and *Csa1M580750*.*1*, *Csa3M135120*.*1*, *Csa4M361270*.*1*, *Csa3M215590*.*1* and *Csa6M160180*.*1* were abundant in the indeterminate line ‘H1201’ (Fig 10). These data suggested that ethylene might affect the determinate and terminal flower phenotype in cucumber. For the ethylene-associated miRNA target genes, expression of six miRNA target genes was observed. As shown in Fig 11, the expression of Cas-miR172 targets (*Csa5M175970*.*1* and *Csa4M292470*.*1*), Cas-miR396 target (*Csa4M004980*.*1*) and Cas-miR414 targets (*Csa5M152920*.*1* and *Csa6M518040*.*1*) were down-regulated in the determinate line, consistent with the mRNA-seq results. Moreover, they showed the expected contrasting patterns of expression compared to miRNA in determinate and indeterminate cucumbers (Fig 5). These data suggested that ethylene-associated miRNAs might affect determinate and terminal flower phenotypes by regulating their target genes expression, and other ethylene-associated genes might be involved in this physiological process. This hypothesis will require molecular validation in the future.

## Discussion

### MiRNA and mRNA expression patterns in determinate and indeterminate cucumber

Inflorescence shoot apex determination is a common problem in cucumber production and leads to reduced yield and debased quality. Previous work suggests that flowering genes and phytohormones play an important role in the determinate phenotype [1,4]. In the mRNA sequence data, the expression pattern of the flowering gene was quite different between the ‘G1208’ and ‘H1201’. In total, 1,105 differentially expressed genes were identified between determinate and indeterminate cucumber, indicating that TFL induces an extensive activation of transcription (S5 Fig, S10 Table). Among them, many flowering genes showed clearly different expression, such as *FT* (Csa1M651710) and *AP*1 (Csa5M172800.1) (S11 Table). Many flowering genes were abundant in determinate cucumber, indicating that the terminal flower was induced in the inflorescence shoot apex. Moreover, GO analysis suggested an activation of gene networks involved in biological regulation, reproduction and the reproductive process (Fig 8). These observations confirmed that the terminal flowering of the ‘G1208’ cucumber was also regulated by the plant flowering pathway [31].

The identification of miRNAs is an indispensable step to facilitate our understanding of the TFL phenotype in cucumber. As a gene regulator, miRNA plays an important role in the molecular regulatory mechanisms of plant flowering and reproduction [11]. The floral transition is controlled by a complex regulatory system, which includes the photoperiod, phytohormone, temperature and plant age. For example, miR156 was reported to regulate plant flowering time by controlling its target gene Squamosa Promoter Binding-Like (*SPB/SPL*) expression in *Arabidopsis* [32]. Overexpression of miR156 prevents flowering in response to vernalization. This flowering behavior is also regulated by miR172. Overexpression of miR172 resulted in floral organ identity defects, which showed a similar phenotype to its target gene *AP2* mutants in Arabidopsis [10]. In gloxinia, miR159 was reported to regulate flowering time, and the expression level of miR159 was negatively correlated with its target gene *SsGAMYB* during flower development. Overexpression or suppression of miR159 leads to significantly late and early flowering behavior, respectively [33]. In this study, over ten million reads of 16 to 30 nt were obtained from each library. A total of 537 conserved and 581 novel miRNAs were identified in the G1208 and H1201 libraries. Most conserved miRNAs showed high sequence similarities to other plants, consistent with the previous report [25], and the majority of conserved miRNAs showed relatively higher reads. Among them, 8 known miRNAs and 23 novel miRNAs were identified as differentially expressed miRNAs between the inflorescence shoot apex determinate and indeterminate cucumber varieties (Fig 4). Cas-miR160, Cas-miR164, Cas-miR167, Cas-miR169, Cas-miR319 and Cas-miR398 were up-regulated in the ‘H1201’ cucumber cultivar, while Cas-miR171, Cas-miR172, Cas-miR393, Cas-miR396, Cas-miR399 and Cas-miR414 were up-regulated in the ‘G1208’ cucumber cultivar. This indicated that these miRNAs play a crucial role in the TFL phenotype (Fig 5). Moreover, Cas-miR172 was 3.5-fold more abundant in the inflorescence shoot apex determinate line compared to the indeterminate line; miRNAs and their target genes usually show contrasting expression patterns. Thus, the target genes of three miRNAs with high expression patterns in determinate cucumber were chosen to perform the real-time PCR assay. The Csa4M004980.1 (target of Cas-miR396), Csa5M152920.1 and Csa6M518040.1 (target of Cas-miR414), Csa5M175970.1 and Csa4M292470.1 (targets of Cas-miR172) were down-regulated in the inflorescence shoot apex determinate cucumber variety. However, not all miRNA target genes have contrasting expression patterns compared to miRNA. We could not find differential expression of Csa6M296960.1 between determinate and indeterminate cucumber, suggesting that some predicted target genes did not function in the inflorescence shoot apex or plant flowering process, or that these genes might not be miRNA target genes.

### The inflorescence shoot apex determinate phenotype is associated with phytohormones

**Fig 12:**
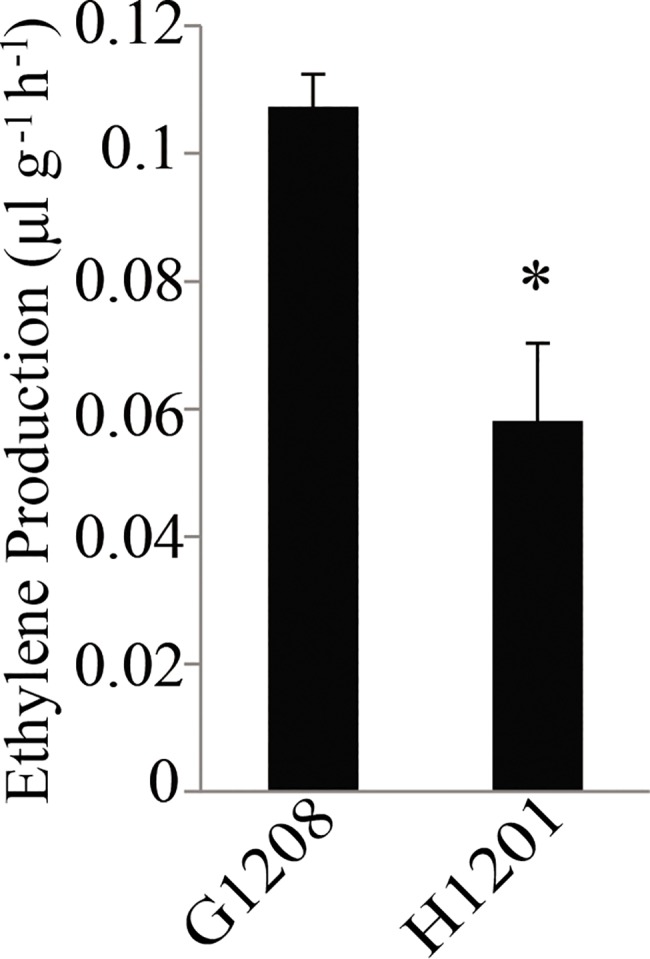
Ethylene production of determinate and indeterminate cucumbers. The x-axis label indicates different cucumber varieties; the y-axis label indicates ethylene production (*P < 0.05, Student’s t-test).

Phytohormones are signal molecules produced by plants that regulate plant development, flowering and ripening in extremely low concentrations, e.g., ethylene and auxin [34–36]. In this study, as shown in Fig 9, the plant hormone signal transduction pathway yielded the highest enrichment factors for DEGs. Intriguingly, the DEGs were found in many plant hormone transduction pathways including the auxin, cytokine, gibberellin acid (GA), abscisic acid, ethylene, brassinosteroid (BR), jasmonic acid and salicylic acid transduction pathways (S4 Fig). As a crucial plant hormone, ethylene was reported to influence many aspects of plant growth and development [34]. Moreover, we observed that ethylene production was significantly higher in the determinate cucumber than in the indeterminate line (Fig 12). The ethylene signal transduction pathway was the most DEG-enriched pathway according to the sequence data (S11 Table).

The biosynthesis of ethylene is regulated by other plant hormones. For example, increased auxin will trigger transcriptional activation of subsets of ACS genes, which produce abundant ethylene; ethylene positively controls auxin biosynthesis, increasing free IAA levels [37,38]. It is reported that both cytokinin and BR hormones control ethylene biosynthesis by enhancing the stability of type-2 ACS proteins [39]. Furthermore, GA also interacted with ethylene to regulate plant development, especially flowering and stomata development [34,40]. Several ethylene-associated genes were chosen to perform real-time PCR, and some were significant differentially expressed, including the ethylene synthesis-related genes, 1-aminocyclopropane-1-carboxylate (*ACC*, Csa1M580750.1), 1-aminocyclopropane-1-carboxylate synthase (*ACS*, Csa4M049610.1) and 1-aminocyclopropane-1-carboxylate oxidase 5 (*ACO5*, Csa4M361270.1). The expression of ethylene response gene also differed, e.g., the ethylene-responsive transcription factor 1B (ERF1 like, Csa3M135120.1 and Csa2M177210). However, not all mRNA-seq data was confirmed by RT-qPCR. The Csa1M651710.1 (*FT* gene) was abundant in ‘H1201’ in the mRNA-seq data but abundant in ‘G1208’ according to the RT-qPCR assay result. Thus, six biological replicates were created to confirm gene expression by RT-qPCR, and the result demonstrated that *FT* was highly expressed in determinate cucumber (Fig 10). *FT* homology was reported as the originator of florigen in flowering plant species [41]. This study indicated that the difference between ‘G1208’ and ‘H1201’ might be due to the hormone signal, and ethylene might play a central role in the determinate phenotype in cucumber. In this study, many miRNAs that targeted ethylene-associated genes showed different expression profiles between the determinate and indeterminate cucumber lines. For instance, Cas-miR172 and Cas-miR414 which target the AP2-like ethylene-responsive transcript factor (*RAP2-7*, Csa5M175970.1) and the zinc finger protein CONSTANS-LIKE 4 (*COL4*), respectively. Both are ethylene-responsive genes, and they are associated with flowering in plants. It was further indicated that the significant involvement of ethylene-associated genes and miRNAs in regulating the determinate which can affect inflorescence and flowering in cucumber. In summary, this study supplied new evidence of a new role for ethylene in plant flowering.

## Supporting information

S1 Fig Length comparison of small RNAs from ‘G1208’ and ‘H1201’ cucumbers. (a), distribution of small RNA length from ‘G1208’ (S1) and ‘H1201’ (S2) lines; x-axis represents length of small RNA; y-axis represents percentage of small RNA. (b), (c), sample of ‘G1208’ and ‘H1201’, respectively; x-axis represents length of small RNA. The y-axis represents numbers of small RNA identified in this study. The number of 20–25 nt sequences is greater than the numbers of other sequence lengths in the libraries.
(TIF)

S2 Fig MiRNA nucleotide bias at each position. (a) miRNAs detected from ‘G1208’, (b) miRNAs detected from ‘H1201’. Four colors indicate four nucleotides. Red represents A, green represents U, blue represents C and yellow represents G.
(TIF)

S3 Fig MiRNA first nucleotide bias. (a) miRNAs detected from ‘G1208’, (b) miRNAs detected from ‘H1201’. Four colors indicate four nucleotides. Red represents A, green represents U, blue represents C and yellow represents G.
(TIF)

S4 Fig Plant hormone signal transduction pathway and DEGs. Green color, down-regulated. Red color, up-regulated. Blue color, mixed regulated (control by several gene: one gene up regulated, while another gene was down regulated).
(TIF)

S5 Fig The volcano plot map of all mRNAs sequenced from sample ‘G1208’ and ‘H1201’. The x-axis denotes fold change (FC) (a minus 2 value represents a negative 2.0-fold change); the y-axis denotes false discovery rate (FDR) (a minus 10 value represents a negative 10.0-fold change). Green dots represent differentially expressed genes and red dots represent genes with no distinct difference.
(TIF)

S1 Table All target genes of conserved miRNAs. (XLSX)

S2 Table The primers used in this study. (XLSX)

S3 Table Detailed information on the known miRNAs. Gene families, miRNA precursor structure, miRNA precursor and muture miRNA sequence.
(XLSX)

S4 Table Detailed information on the identified novel miRNAs. Gene families, miRNA precursor structure, miRNA precursor and mature miRNA sequence.
(XLSX)

S5 Table The comparison of miRNAs abundance in ‘G1208’ and ‘H1201’. (XLSX)

S6 Table All target genes of novel miRNAs. (XLSX)

S7 Table KEGG analysis of the miRNA target genes. The function of different expression miRNA targets were enriched in 12 metabolic pathways.
(XLSX)

S8 Table Transcriptome data of ‘G1208’ align to the cucumber EST library. (XLSX)

S9 Table Transcriptome data of ‘H120’1 align to the cucumber EST library. (XLSX)

S10 Table All new genes found in ‘G1208’ and ‘H1201’ samples. (DOCX)

S11 Table The annotation of differentially expressed mRNAs in ‘G1208’ and ‘H1201’ samples. (XLSX)

S12 Table The KEGG pathways and the corresponding unigene IDs in the transcriptome of cucumber flowers. KEGG pathway enrichment analysis of the differentially expressed genes revealed effects on 62 metabolic processes.
(XLSX)

## Abbreviations

TFL: Terminal flower
FT: Flowering locus T
AP1: APETALA-1
AP2: APETALA-2
RAP2: AP2-like ethylene-responsive transcript factor
COL4: CONSTANS-LIKE 4
AGO1: ARGONAUTE-1 protein
sRNA: small RNA
GO: Gene ontology
KEGG: Kyoto encyclopedia of genes and genomes
DEGs: Different expression genes
RT-qPCR: Reverse transcription-quantitative polymerase chain reaction
GA: Gibberellin Acid
ABA: Abscisic Acid
BR: Brassinosteroid
JA: Jasmonic Acid
SA: Salicylic Acid
ACC: 1-aminocyclopropane-1-carboxylate
ACS: 1-aminocyclopropane-1-carboxylate synthase
ACO: 1-aminocyclopropane-1-carboxylate oxidase
ERF: Ethylene-responsive transcription factor
