# Supplementary figures and images for: Comparative analysis of miRNA and mRNA abundance in determinate cucumber by high-throughput sequencing
Source: PLoS One. 2018 Jan 5;13(1):e0190691. doi: 10.1371/journal.pone.0190691 (PMC5755913; doi:10.1371/journal.pone.0190691)

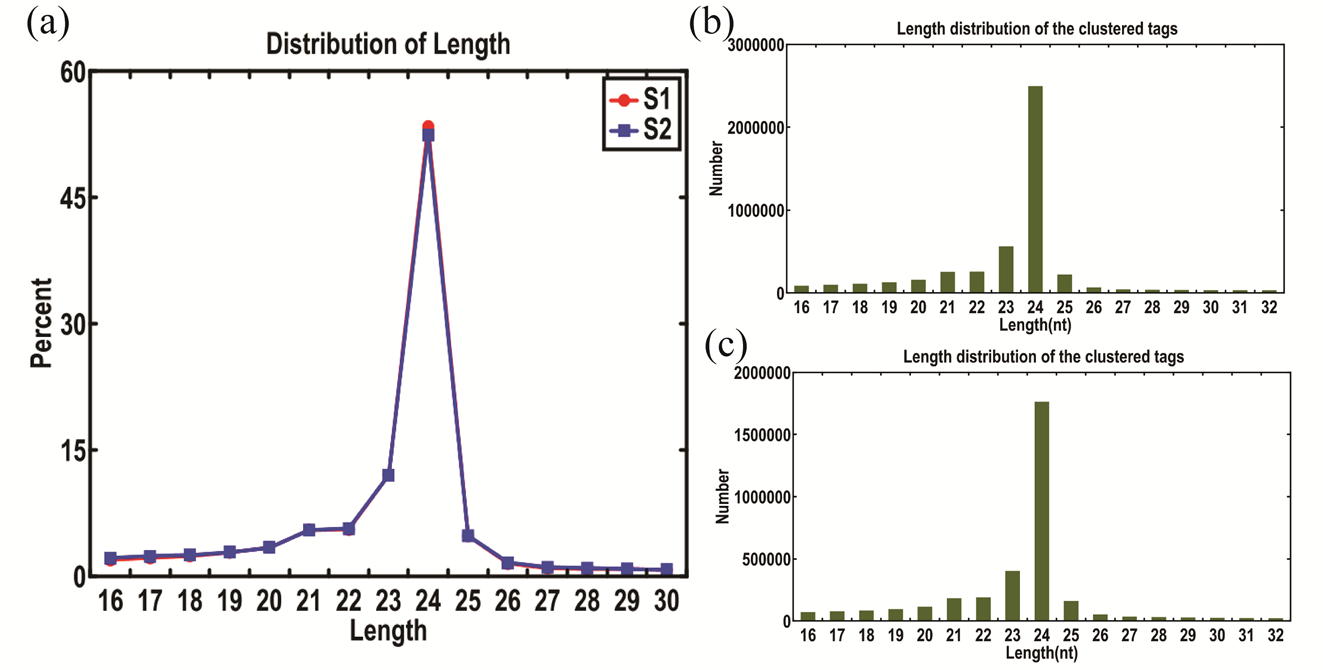

Supplement: S1 Fig — (a), distribution of small RNA length from ‘G1208’ (S1) and ‘H1201’ (S2) lines; x-axis represents length of small RNA; y-axis represents percentage of small RNA. (b), (c), sample of ‘G1208’ and ‘H1201’, respectively; x-axis represents length of small RNA. The y-axis represents numbers of small RNA identified in this study. The number of 20–25 nt sequences is greater than the numbers of other sequence lengths in the libraries. (TIF) [file pone.0190691.s001.tif]

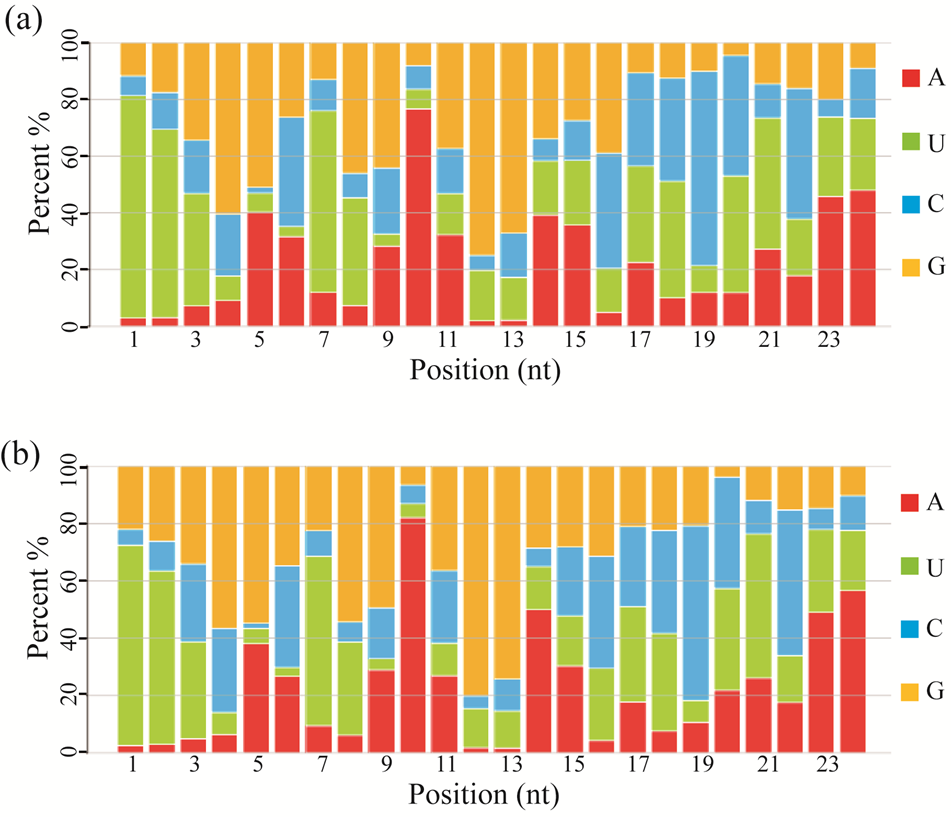

Supplement: S2 Fig — (a) miRNAs detected from ‘G1208’, (b) miRNAs detected from ‘H1201’. Four colors indicate four nucleotides. Red represents A, green represents U, blue represents C and yellow represents G. (TIF) [file pone.0190691.s002.tif]

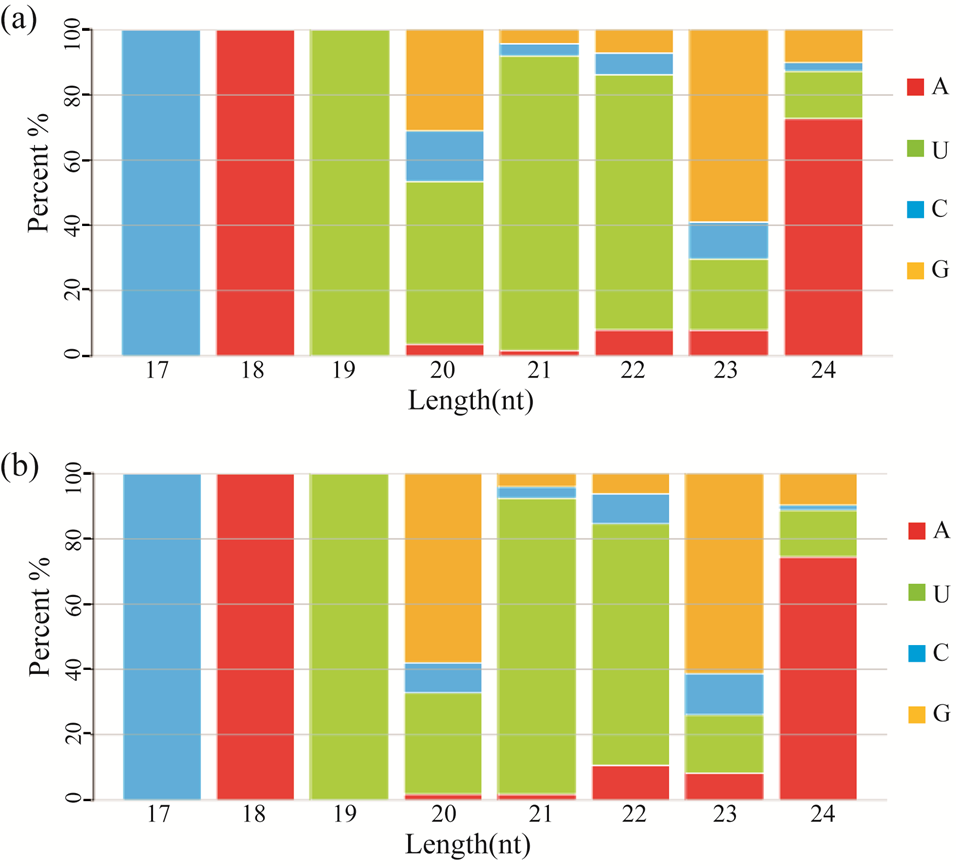

Supplement: S3 Fig — (a) miRNAs detected from ‘G1208’, (b) miRNAs detected from ‘H1201’. Four colors indicate four nucleotides. Red represents A, green represents U, blue represents C and yellow represents G. (TIF) [file pone.0190691.s003.tif]

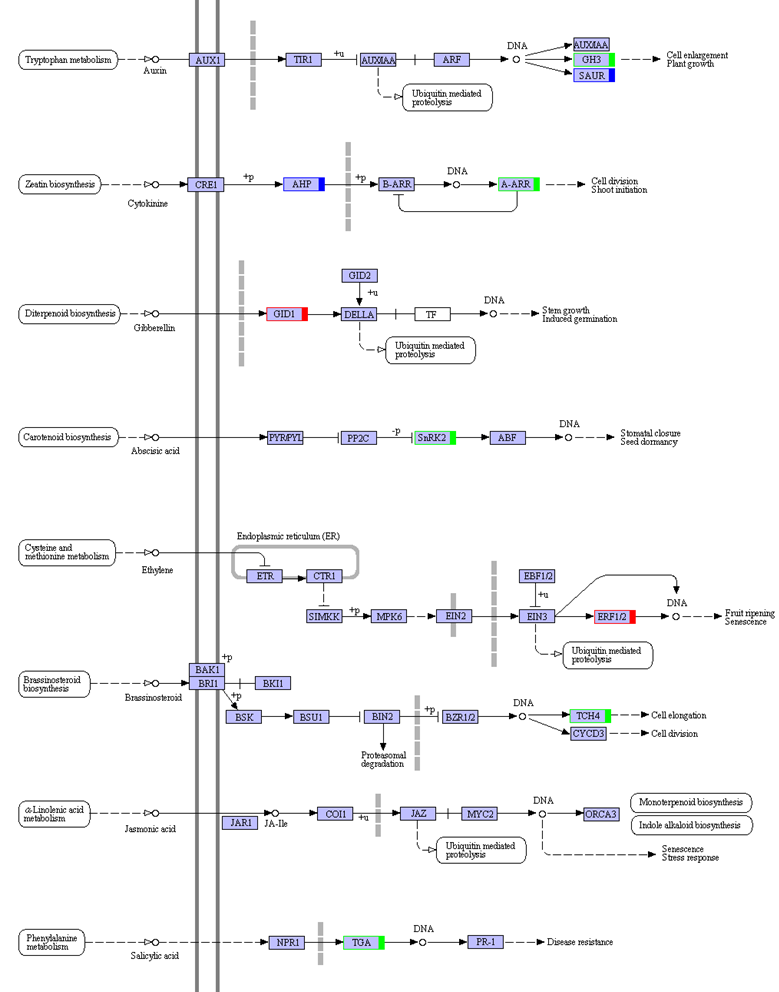

Supplement: S4 Fig — Green color, down-regulated. Red color, up-regulated. Blue color, mixed regulated (control by several gene: one gene up regulated, while another gene was down regulated). (TIF) [file pone.0190691.s004.tif]

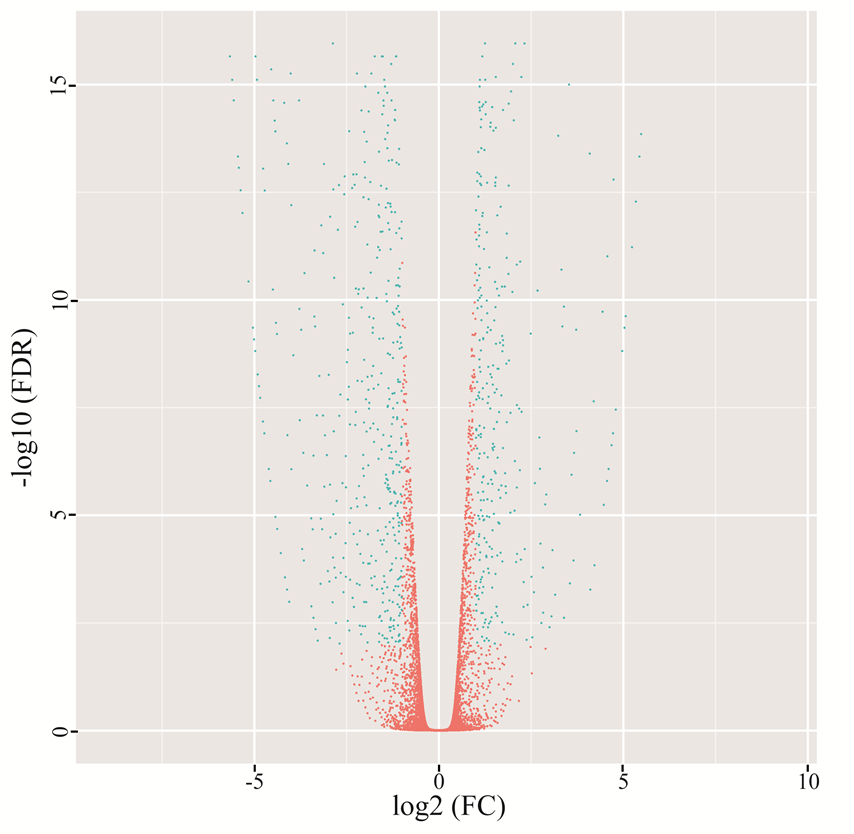

Supplement: S5 Fig — The x-axis denotes fold change (FC) (a minus 2 value represents a negative 2.0-fold change); the y-axis denotes false discovery rate (FDR) (a minus 10 value represents a negative 10.0-fold change). Green dots represent differentially expressed genes and red dots represent genes with no distinct difference. (TIF) [file pone.0190691.s005.tif]
