# Supplementary material for: Comparative analysis of miRNA and mRNA abundance in determinate cucumber by high-throughput sequencing
Source: PLoS One. 2018 Jan 5;13(1):e0190691. doi: 10.1371/journal.pone.0190691 (PMC5755913; doi:10.1371/journal.pone.0190691)
Supplement: S10 Table — (DOCX) [file pone.0190691.s015.docx]

Table S10. All new genes found in ‘G1208’ and ‘H1201’ samples.

>cucumber_newGene_1 cucumber_newGene_1.1

ATGAAGAATGGGTTTGCCAATCGGAATTCTTTGATGCTCTAATCTCTGATCAGGCATTATTAACATCCATAATTGCAGTCCCATCAAGATTTCTCAGTGTTCAAAACTTCTGATTAGTTCACTCATTTGACTGTTTAAGCTTCTTGTCATCTGCCGCATTTGCCTCATTCTGATTAGTTCACTTTGCTTCTAGACCTATTATTCCATATTGTTCCAACAACCAATACCTCTGACAGGGAAGAATCTTCTTCCAGAGCTAATTATTACCAAAGTGAAGAAGATGTCAAGCCTAAATTAAACTCACATGAAGGGATTCGTTGTGACGTTGGAGATGAAAATGGGCCCAAAATATGATTCATTTCCTTTACCTAAAAATGCACCAACAAATTGCAGATTATGGCAAATTTTCTACAGGCAGTCACCATATAAAAGATGCACATAGAGTAGCGACTTGTAGTAATATATGATCATAATGCAGTTGCAGAAAATGACGAGCAATCTCAAGTGTGCCTTCTAGTTCAGGGACCGAAAGCACGTAAAATATGGCCTCGAGAAAATGGTGTGCTTAAAAATGGGGGATTGTTTAACTACCCGAATCATTGTTTGGATCTTACATGCAATCTTTCTGGTTGTGACAGTGTTCATTCTGGTTGTTTGTTATCAAACTGCAATACTCCATACTCATTCTTGGAAGCTTCTGTATCTACCTCCCAAGTTTCATTCACTTAGCTTCATTTTATTTTTCTTGGAAGTTAATATTAAAAGCTAAAACAGTTATGCTCAGCAGTGA

>cucumber_newGene_10 cucumber_newGene_10.2

AATATATAAAATTGATAAAAAGATATTTACAAAAATATAGGAAGACATAGTAAAAAAATTTAACCATCTTAATCACGTGTGCCTAAAATAAAAATCCTCAACAGGCTCACGGCCGTGTAGCAGAGTATGGAGACAAGCTGGCGAAGGACAGTCTTGAGCAATGAGAAATTTTAAAACACTCCTAATTCTTCTCTGAATTATCCAAGAAATTTCACAACCAACTTTTTATAAACATTGGAACAACGTTATCAAATTTATATGTGAGATTGATAGAGAGAAGGAGAAGGAAGAAAGGAGCGTACCCAATGTTCATCCTTCCATCTCTCACTGAATCCTTCACCTCAATCTTCACATTCATCTTCTTCATTTCTCTTTTCATTCTCCTTCTAATCCCCAAACTCAAATCAAAACCCAATCAAAATCTCCCACCATCCCCACCTAAACTTCCTCTAATCGGCAACCTACACCAGCTAAATCACCATCCCCACATCTGTTTCCGCCGCCTATCCCAAAAGTTCGGCCCCATCATCCTCCTTCAACTCGGACAAATCCCAACTCTCATAATCTCATCACCAAAAATCGCCAAAGAAGCCTTCACAACCCACGATCTCGTTTTCTCAAGCCGTCCTTTTCTCTTCTCCGCCCAACACACCTTCTACAACTGCACTGACATTGCTTTTTCCCCTTACGGCTCCTACTGGCGCCATGTCAGAAAAATATGCATCCTCCAACTCCTCAGCAACAAAAGGGTCCAATCCTTTGCCTCCATTCGACAACAAGAAGTTGCTCGTTTGGTAAGCAGAATCTCCCACTCCAACAATCAGGTCGATTTGTCCAACTTGTTAGCGATGTATGGAAATGATGTGATCTGCCGAATGGCGCTTGGGAGGGAATTTTCCGCCGGTGGTGCGTACCATCTTCACGGGATTCAAGAGATAATCGATGACTATCAGCTTTTGCTTGGTGGGTTCTGTTTAGGGGATTTGTTCCCTTCCTTGTCATTTATTAGTAAGCTAACAGGGATGAAATCGAGGCTTGTGAGAACGTTTAAAAGGTTCGATAAGTTGTTGGATCAAGTAATAGTTGAACATCAGAGTCCCGAGGGAGAGAAACTTGGAGAATCTAAGGATCTTGTTGATGTTTTGCTTGATATACAAAAGAATGGATCTGATGATAGATTCTCCCTGACTATGGATAATGTTAAAGCCATTCTTTTGGACATGTTTATTGCAGGGACTGAAACGACGTTTACAATTCTTGATTGGGGGATGACGGAGCTGATAACACACCCAAAGGCCATGGAAAGAGTACAAAAGGAAATTCGAAGCATAGTTGGTGGCAGAAAAATTGTAACAGAGGGTGATATTTTAGAAATGCATTATTTGAAAGCTGTGGTTAAAGAGGTGCTCCGATTGCATCCACCAGCGCCGCTTGCACTTCCAAGAGAAACTACAGAAGATGTGAGGATTGAAGGTTACGATATTCCAGGTAAAACAAGGGTTTTTGTGAATGTTTGGGGAATTGGGAGAGACCCAGAATGGTGGAAGAACCCAGAAAGTTTTGAGCCAGAGAGATTTGTAGAGAATGAAGTTGATTACAGAGGACTGGATTTTGAGTTCATTCCGTTTGGTGTTGGAAGAAGGATTTGTCCAGGTATTACTATAGGGATGGCCATGATTGAGATTGCTTTTGCTCAAATTTTACATAGCTTTAATTGGGAGCTTCCAAGTGGGATTGAAATTAAGGATTTAGATACGACTGATGTTGTTGGTGTCACAATGCATAGAAAAGCTCACTTGGAGGTTGTAGCAAAGCCTTACTCTGGCTCATCCATCTCAAATTAATTACTACTTTTCTTGGAGGGTATTGAATTGAGCTCATATTAAATAATTGCTTTGAATTTCAATTGAATGAGATATTAAAAGAAGAAGACAATCCATCGTTCAATAATGTGTAAAATCCTCGATAGGCTCACAGCCGTGGATGGAACCAATGGCGAACGGAAGTCTTGAGCAATCATAAATTTTAAGATCCTCCTATATAAACATTGAAACAACGTTACAAAACATAAAGAATTAAGGAGAAGGAAGAAAGGAGGGTGTACACAATGTTCATCCTTCCATATCTCAACCAATCTCTCACTACTTCATTCTCCACATTCATCTTCTTCGTTTCTCTTCTCCTTCTCCTTCTCATTCCCAAACTCATATCAAAACCCAACCAAAATCTCCCACCATCCCCACCTAAACTTCCTCTAATCGGCAACCTCCACCAGCTAAGTCGTCATCCCCACCTCTGTTTCCACCGCCTCTACCAAAAGTTCGGCCCCATCATCCTCCTTCAACTCGGACAAATCCCAACTCTCATAATCTCATCACCAAAAATCGCCAAAGAAGCTTTCAAAACCCACGACCTCTCCTTCTCAAGCCGCCCCCTTCTCTTTTCCGCCCAACACGTCACCTACAACTGCACCGACATTGCATTTTCCCCTTACGGCTCTTACTGGCTCCAAGTCAGAAAAATATGCATCCTCCAACTCCTTAATGCCAAAAGGGTCCAATCCTTTGCCTTCATTCGACAACAAGAAGTTGCCCGTTTGGTTAACAGAATCTCCCACTCCACCGATCACGTGGATCTCACGAGCCTCTTGGCAATATATGCCAATGATGTGCTCTGTCGAATGGCGCTTGGGAGGGAATTTTCCGCCGGTGGTGCGTACCATCCATTCAAGAGTTACTCGAAGAGTATCAGGTTTTGCTTGGAGGATTCTCTTTTGCGGATTTGTTCCCTTCTTTGTCGTTTATCAGTACGCTAACGGGGACAAAATCGAGGCTTGTGAAGACGTTTAAAGCGTTCGATAAGTTAGTTGATAAAGTAATTGCTGAACATCAGAGTCCCGACCGTGAGAAACTTGGACAATCTAAGGATCTTGTTGATGTTTTGCTTGATATACAGAAGAATGGTTTTGAAGACAAATTTTTCCTTACTATGGATAACGTCAAAGGCATTATTTTGGATATGTTCGTTGCAGGAACTGACACGACTTTCATAGCTCTTGACTGGGGGATGACGGAGCTCATAACACACCCAAATGCCATGAAAAGAGCACAAAGTGAAATTCGTAGAGTTGTTGGTGATAGAAGAAATGTAACAGAGAGTGATGTTTTAGAAATGCCTTATTTGAAAGCTGTGGTTAAAGAGGTGCTCCGATTGCATTGCCGGTTTCGACTCCAAGAGAAACTATAGAAGATGTGAGGATTGAAGGGTATGATATTCCAGCTAAAACAAGGGTTTTTGTGAATGTTTGGGCAATTGGGAGAGACCCAGAATCATGGAAGGATCCAGAAACTTTTGAGCCAGAAAGGTTTTTGGAGAGTGAAGTTGATTACAAAGGGTTGAATTTTGAATTCATACCATTTGGAGCTGGAAGAAGGATTTGTCCAGGCATTACCACGATTGAGCTTGGTTTGGCTCAGATTTTGCATAGCTTTGATTGGGAACTTCCTAATGGAGTTGAAGCTAAAGATTTGGATATGACTGAAGTTTTTGGTATCACAATGCATAGAAAAGCCCACTTGGAGGTTGTGGCAAAGTCGTACTTTGCCTCGTCCCTCTCCAAATAACTGCTTTTCCTCAAGATTAAAATATTCTTCCTTTCAAGTTGGTTTATTTTTAATAAAAACTTTCAAAATGCCTTATTTAAGATCATCTACTGTCAATAAATCTTAAATTTAGTTTGTAAATTATTAGTTTAATATGATGTAAAATCAGGTTGGGATAAATTTAAAAGTAAATTAATA

>cucumber_newGene_100 cucumber_newGene_100.1

GTGGTTTTTGAAAAAGGCAAACAAAGTTATGAATGTGTGTGTGTGTATGTATGTATATATAGTAACCCCACCTTCATCACTACCCATTACTTCATTCCCATCAAATTCTCTCTCTCTCTCTCTCTCTTTGTTTGTTTTGTGTAAATCAGAAGAAGAAGAAGAAAGAAAGAAAGAAAAAGAAAAATGGCAGACGAAGGAACAACAAATTGCATCGATATCCTCCTCGCTATTCTCCTCCCTCCTCTTGGTGTCTTTCTCAAATTTGGATGCCAAGTTGAGTTTTGGATCTGCTTGGTCTTAACTTTCTTTGGTTACATCCCTGGCATTATTTATGCTGTTTATGCCATCACCAAGTGATCATCCAATTCAAAGGATTGATGCAAGAAATTATTGGAACTCATCATCATGCATGGTCTCTCCTCACCGACACTCTCCCTTCCTTTTTTCTAATTTCTCTTGATCTCTGTTTTGTGTGTGTTTGCTTGTGTGATAATCATCATCCCATCCATCAAAGACATCTACACAATTGTTGCTTCTAATTTCTTTTTTCTCTTTTGTAATTTGTAATTTTGAATTATTTGGGGGTAATTTAATCATGTGTTGTTCTTGGTTGTATTGATTGAATTGG

>cucumber_newGene_101 cucumber_newGene_101.2

GTTTAAATCCCCATTTTCCTCTGCATTGCACCGAAAATGTTGGGAATTCCGTTGCAGTTTGGGGGAATCAAAGGTGAAGACTGGTTTTATATTCCGGTAAGGGGCAAAGAATTATAATCACCAAAACCCGTCAAGGAATCCTACCAAGACCGATGAAATCGAGAGCCCATTGAGTAAAGTTGATTCTTGGAACACACAAGGCCTTCAGTTCCAGCACAGTACTTCTTTGAGGTCAATGCTTAGAATCAAAGAAAGTTGATTACGTACAACCTTGAATGGTTCCGTTTCTTGTGGCCCTTTAATGGTCTGCATGGCCTTTTCTGCATCTACAAGTCTTTGACCTCTAAAGAACTATGAGGGATAGGAGGACTTGTGATATCGAGTTTCAACTTTATTTCATTCTGCATGATCTGTGGGAATCTTTCAAGGAGTGGAGTGCATATGGCGCTGGAGTTCCTTTGGTACTCATGAAGGTACTTGACCTTCGAGTGGAATATGTTAACCCTCATGAGTATGAATTCTTTTTGTATGTGGATTTCTGTGCGTGATTTCCAGGTTGAGTGGAATATATATGTGTTTAATGAACTGTGAGTGTGAGAGTCATTTTACCAAGACTTATCTTCTACTCTACTGTGGGTTTCTTTTTAGATGTGATGATCAAGAACTTTTCCTACCATTCTTAACGTACGAGAACATTGGTTTTCCCCAGAAGTTGGCTCATTCTTTGTTTCTTTTTATTTGGTAGGCTGACTTGTGAGCACAGTGATGGAAGCATTGACTATGATTTAGGAAAAATCTTTTACATTAATCCATTAAAGACGTTTTCCAGCATGTTTCCACAAGTTAAATGGTTGATGATCCCACTGTTCTCTAGAAAAGTAAAACTTTTTTCCTAAATCGTAGCCAATGCTTCCATCGCTGGTTGTATCTCTACAAGAATCAAGATCACTATCATCGCTGGTTCACATATATTCTAGTTGGACAAACATAAATTTTATATTAAATCTCTTATAAATACACCATTCCCGTTACATACAAGGTTCACTTTTTCTCAAATTTAAATTTACACGTGAATGACTACCATATTATTATTCAACAAAACACAAACAGAAAACATAGCAGTTTACACACAAGAAGAACATAGAACATAAAAGGACTGATGATAAGAAACACACACACAACCAAGCAAGCAATTATGAAATGGATGGTTGTTCAGCAATTTGGAAAAGAAGTTCCACAATCATTAGCTACTTTTCTTGCATTTGGGGAGTTAACAAAGTTCTTAATTAAGAAAAGGGTTTCTGAGGTAACCACAGAGACGGGGCTTTTGCTCTTTGATCTTTCTGCAACATTGGTTTGATGGTGGAGTTCTTGACATGATTGACTTTCTTTTTCTTTGTTTTGTTATCTACTCTTAAACCATATTCTTAAAAATCATCCAAGTTTACTACTCAGAGAAGCAAATGGATGGCCAGGAAGAGTGTCTCGCCAAAGTGCATCATGATGGCGGAGCCCAACAAATAGGTGGGGTGGAGAGTGGAAGAAGGGAGGATATATTTGGATTTGAATGGGAGAAAGAATGGAGTGGAAGTGATGAGAGTGTTGATACAACTTTTACAAAAATATAAACGGTTGAAC

>cucumber_newGene_102 cucumber_newGene_102.2

GCGGATTATGGCATTTCCCCCCACAAAATTTCAACAAAAAATTATAATGTTGAAGGCTTAATGCGCCAAACGAAATGAGGGAATCGAAGCTTTTGGACTTTCCCACTGGAGAAGAAGAATAAGCTTGTGCGTGTGCCTGTGCTTGCAACTCCTCTCTTCAACCCGACACCACCGATTACTCGAAAGGGTCAGCAAATGGATGCTTTGGTGGTGGTTTTCTAGCACAGGGAATTTGGTTGTTTGGAAACTGGTATCGTCGTGGAAGTTGAACTCTTAGTTATCTGAAGTGATGTGGACGAATATTTTCAAAATTGGTGGTCTACATCAAATATCATGGTTCCAGTTTCTTCCTAACGAATCTGATTTGATTACCTTACCAGACAAAAGTGCGAAGGTTGAGCACAATGATGCTGCCACATTCTTGGTTCTTTCATCACATGTGCAATTACAGAAGGAAGGATTTCTAAGTACTTGGACCAACTCATTTGTAGGGCCTTGGGACCCATCTCAGGGCTTGCACAATCCTGATGAGAAAATTAAACTCTGGCTTTTTCTACCTGGACGCCATTCTTCTGTCGTTGAAACTGCTCAAGCAGCTGTTTCCAAGTTAAGGGTTGTTGCATCTGGACTGTGGATCTCTCCTGGAGATTCAGAGGAGGTTGCGGCTGCCCTTTCTCAGGCATTACGAAATTGTATCGAAAGAGCCCTAACTGGACTTTCATACATGCGGTTTGGAGATGTCTTCACAAAATATCACCACATGCAAAGTGAAGAATTATTCAGGAGAGGGCAACCTACAATGGAGTTCATATTTGCTGCTACCGAGGAGGCAATCTTTGTGCATGTCATTTTATCTGCAAAACATATTCGAGCACTATCAAGTGCCGAGATTGAAAGAGTTTTGAAGAATTCTGCTCATAATTCCTGTCTTGGACTTCCAGTGATTGTTTCGCCTCATGGAATTCGAGGTAGGTTTACTGGGTGTTGTGCCAGTGATGTTGTAAAACGGATATATTCCAGTTCTGGCAAGTCTAGGACTTCATACGGATTTGTAGGTTTACCTCATCATGTCTCTCAAGGTGGCTGCCAGTTAAAGGGGCAAAATTGCTATGTCGAAGTTACCCTAGGGTGCCCTAAATCAATGAGTGAGAAGCCACTGCAATCTAATTCAAATTACACAAAAAATGTATCAATGCCTCAAGTTACAGAATCTCTTACCGGACGAGGTGATCTGAAAGGATCGTCAAACCATTTATCTTCTCATAAAAAGACTTTCATATATCCATCAGAGGCAGTGCTTGTTTTGTTATTGCAAACATCGTTTGCCAGGTCTTCTTTGAAAAGATTTTGGCTGCAAAATTGGATAGGACCCTCATTACCTGGTTCATCTTTCAACGTGCATTGTGCTGGAAATGTAGATTATATGGAAGGGTTGTGGACTGAGACTGATAAAATACGTTCACAGCATGGTTATGATAGCAGCAGCAATAGTAACAGTAGCAGCATTGCTAGCATAAGCAGTAGTTCTAATGATAGTGATTGCAAGACAGGAGCCAGCGAACTTGAAGCAGATGCTGATTCCTTATCTTGCAGGCAGTCTGGATTGTCTTCAAATGACCAATCCGCAATCAGTTCCAGAAAATTGGGTATGAAGCGGCCTCGTTCGGGGATGCCAGATGCATTAGATCAAATGGGTACAGGTGCTCAAATTCAAGATGCTTTCAAATCTGATTTTACTTCCACTGAGCTGATTGGATCTCCTTGGGACTGGGAAGACGATGACCGGGGTGGGGACGACATAGAAGACCTCCTTTTGCATTTTGGGGGCTTTGGAGACTTCTTTGAGAATGACGTTTTACCTTTTGGGGAGCCTCCAGGAACTACAGAGTCACAATCTCTTATGTTTTCTGCTCCGGACTACACTGATGTAGGTAGCAGTCCAGTTGTGGTCATGGATGTTTCCGATCAGATGCTTTTACCTGTGGGGTTTCCATCCTTTGATAGCTTCAACCCAGCTGTTCCAATGACGACAGAGGAGGTTCTAAGCAAAGATCACGAAGTCACAAACAATGCATTGTCCTCAGTAACAGCCAACCAAACCCCAGTGTCTTCTTCTGGGGAGTTTGATCAAATCACTAAAGCTGAAGCTCTGATGACACTTGCTCCCGAATATGGAGCAGTTGAAACTCCTACAAGTGAGTTCTCTTCATCAATGTTTCGAAGTCCATATATTCCAAAAACTCGGGAGCTAGAGAGTTCAAACTTAAGCACAAACAGTTACATATATGGTGCAACACCACCCTCCTCACCACATTTTGATAGGTCCGATGAGAAGAGTGGTATATCTTCAAATACAAAACCATCTAATGTTTTACGGGCAAAAAATTATTATATACATGTTGACAATGTGAAAGAGAAACATATAAGAAAATCAGCTCCCTCTAAGAATAGCATCTCTACATCTGATGGGCTGGCATCATCTCTTTCGAATCACAATGCTGTCAAAACCACACAAAGGAAAACGACGGAGGACAGTGTCGAAGCTGATTGTTTATTTATGTCTCAGAAGCACGTTCTTGCGATGGAAGTCGAATGCTTAATGTTCCAAGCTTCTATGTGCAGATTACGACACACCTTGCAGTCGTCTGGTAGTTCCACAGTTTCTGGTACAACTCAGCTATCTAGTGATCCAAGTACTATTACAGACTATATGGCAAATGAGGTGAAGAAGAAGGATACTAGTGTTCCAATTAGAATAGCCGGAGAAGCTGATGGGGGAATACTTGATGGACACCTTAATGCACCTGTTGGTGTTTGGCGGTCTGTTGGAGTTCCCAAGGTTCCAAAGCCGTCAAACTCACCTAGTATGGAACTTGGGTCATCCTTACCCCACAATTCTTTTCATGAGGATGGAGTTCTCTCTTATGGGCAGAGACAACCACTTCAGGAGCTCCTTGATGCCTTCCCTTTAATTGTCCAGCAGGCTACTTCTTTTGTTGACCTTGCCTTGGATGCAGAATGTGGCGATGGGCCATATGGCTGGCTAGCATTACAGGAACAATGGAGGAGGGGATTTTCATGTGGGCCATCAATGGTTCATGCAGGCTGTGGAGGGACCTTAGCCTCTTGCCATGCGTTGGACATTGCAGGTGTTGAGTTAGTGGATCCTCTCACTGCTGATGTTTATGCCCCGTCTGTAATGAGTTTGCTGCAGTCTGACATGAAAACAGCCTTGAAATCTGCATTTGGCACTTTGGATGGGCCATTATCTGTAATTGATTGGTGCAAAGGTCGTGGCCAATTAGGTGATTCAGGAAGCACGGGTGATGGATTATCTGCCGAGTCTATTGTAAATGAATCTAAAGATTCTTCAAGTACTGTTATGCAAAATATTGGAGAGCCCTTAAGCCCGTCACATTCTTCTGCTAGTGGATCGTCTAGTCTCAAAGGTAGTACCACGATGGATGGTTCCAAAATGGATGAGACTTCCCAAAGGAGATCAAACCAAGAGATTTGCAGTTCAGGATCTGACCAACAACTACTTCCTTTGCGGCTGAGACCAACAGTTCTTCTTCTCCCATCACCTGCAATACTTGTTGGGTACCAAGATGATTGGCTTAAGACATCTGCCAACTCTCTGCAACTTTGGGAAAAGGCCCCTCTTGAACCTTATGCTGTACAAAAACCGATAAATTACTGCGTCATTTGCCCAGATATTGATCCTCTTGCATCAGCTGCTGCTGATTTTTTCCAGCAACTAGGAACGGTGTACGAGACATGCAAGCTGGGAACTCATACCCCGCATAATTTAGGGAATCAAATGGACACAGAGTCTGGGAAGTGGTTGTCTTCAGGTTTTGTTCTACTTGATTGCCCCCAATCAATGAAAATTGACAGCAGCAGTGCTTCAATTGTTGGTTCAATTAGTGACTATTTGCTCTCTCTCTCAAATGGATGGGACTTGACAAGTTATCTTAGATCTCTTTCAAAAGCTTTGAAAGCTTTGAAACTCTCTCCATCCATGTCAGCTAATCCAAAAGAAGGAAGTAATGGTTCTTGCATGGTACTTTATGTGATATGCCCTTTTCCCGATCCCCTGGAAGTATTGCAAACTGTTGTTGAATCTTCTGTTGCTGTTGGTTCTGTCATGCTCCAGTCAGATAGAGATAGAAGAACAATATTGTGTAGTCAGGTTGCGAAGTCGTTAAGCTGCTCAGCAGCTGTTGATGAGTCTTCAGCATCGAACGTTTTAGTTCTTCAAGGGTTTACTCTACCTAAATTGGTGTTGCAGATTGTGACAGTTGATGTGATTTTCAGAGTGAGTAGTCCATCCGTAAATGAACTTGTCATTCTTAAGGAAACAGCTTTTACTATTTATAACAAGGCTCGTCGAATATCACGGGGGACATCAAATGATGCTGCCCAGTCATCATCATTATCTAGTAGATCTCATTCAGTTCTATCATCAATGTCACCTTCTATTCCAGGGATGTGGAAGGACTGTGTTGGTCCTAGAATGACTGGTCATTCCCTCCCACGAGAGGGTGAAATTGATGGTACCCTGAGGTCTGGAAACTGGGATAATTCCTGGCAATCAAGGGCTGGAACATTAAATTGTGATCCAAACCGAATAGGAGAATATTATCTTCAAGATGATTCTTGCTACATGTTTGAACCACTTTTTATCCTGGCAGAACCTGGTTCACTAGAGCATGGAGTTTCACCAATAAATCCTGTTACACTAGGAACAGAGTCTTCAAAACCATTATCTGATGACAACAGCGGAGCCTTTTTACAGGGTACAAATTCAACAGTAGGTATGGATATGGGATCTAACTCTCAACTAGATGGGCCTGAGATGGATGGCTTTGGATGTGGCCATCAGAAGAACCCTAGTCTACACTGTTCCTATGGATGGACAGAAGATTGGCGCTGGCTAGTATGTATCTGGACAGATTCGCGAGGAGAATTGTTAGACAGCCATACATTTCCATTTGGAGGTATCAGCAGCAGGCAGGATACGAAGGGTCTGGAGTGCATTTTTGTCCAAGTTCTGCAGCAAGGCTGTATGATACTTCAGTCATGTTCACCTGATACTGGTGTCTCCAAGCCAAGGGACTTGGTTATTGCAAGAATTGGAATGTTCTATGAACTTGAGTATCTAGAGTGGCAGAAGGCCATTTACTCACTATGGGGATCTGAGGTGAAAAAATGGCCCTTGCAACTTCGCCGCTGCATGCCAGATGGGATATCATCAAGTACTAATGGGAGTTCCTTGCAACAACAAGAGATGAGTTTGATACATGATAGAAACCTACCCTCCTCCCCAAATCCTTTATATAGCCCGCATTCAAAAACCACAGGCTTTATGAAAGCTGGTATTGGACAACCTGCTATTAGAAAACAGCTAATGGGTGGTCATGCAGTCGTTGACAACTCAAGAGGGTTGATTCAGTGGGTGCATAGTATTAGTTTCGTTGCAGTTTCGATGGAGCATTCTCTTCAGCTATTACTTCAAGCTGATTCAGCATCTCCTGGTGGAAATCAAGGCAGTGTACACACGGGTTCGTCCATGTATATTGAAGGCTTTACCCCTGTAAAGTCACTTGGTTCCACATCTTCTTCTTATATACTAATCCCATCACCCAGTCTGCGTTTCCTTCCCTCTAACCCTCTTCAGCTTCCCACATGCCTCACGGCCGAATCCCCACCTTTAGCACATCTCCTACACAGTAAGGGCTCTGCAGTTCCACTTTCGACTGGATTTGCTATTTCAAGAGCTGTACCCTCAATGAGAAAGGATTCTCGAAGCAACATGAAAGAGGAATGGCCATCAGTCCTTTCGGTTAGTCTCATTGATTATTACGGAAACAATATCACACAAGAAAAGAATGTCCGCGGAGTTATCAAGCAAGTGGGAAGAAGTTCAACTGTCGAATCCAGAGACTTTGAAATAGAGACACATTTGATCCTTGAATCCATCATAGCAGAGCTTCATGCCTTATCATGGATGACTGTGAGCCCAGCATACTTGGACCGGCGAACTGCACTGCCTTTTCACTGTGATATGGTTTTAAGACTCCGAAGGATTCTTCATTTTGCTGACACGGAGCTCTCTAGGCGCGCTGAAAAAACGAAACGTTAGGACGATGTCAACTGAGGTAATGCTCAGGTTGTTTCTTGCTAATTGTTCTATTAAGACCTTCTTACCATGTAAAGTTATATGTATTATAATTCTTGTACATGCTAGATAGGTGGGCAATTCTTAATTATATGCTTAGTTGGTAGGAATTAGGGAAGAGAGAATTAAATCTCAGTTCAGGTTTATGTGAAAAATTAGGACAGTTGTATATGTAACAAAAGGGAATTGCAACTTGAAAATCAGTGTGATTGAGATCCCTCGATTTTCTCAGAATATAATCCAATGATAGAACCACTTATTTGACAGCCTCTTTTATTACTCCCAGCCAACAGTTTTCAATGACTGTCTATCATAATCGGT

>cucumber_newGene_103 cucumber_newGene_103.1

GAAAAAACCAATAGTATAATGTAAAGAAGAAAGAAAAAGACGAGCGTGTTTCTCACGCTCCATTATTCACGGAGGTCGCCCACGACGATTCTTCAGACCAATCAATGCTTCTCCCCCTTCTTAATTTCTATAAAATGGAAGCCTTGGAATGCCAAGGAAGAACCAAATCCATCAATCCATCAATCCATCAATCTCTCTCTGATTTTTAACTGAAGAGATCCTAATGGCCACTGTTGGAGGTATTAAGGAAGTCCCTTCAAACGAGAACAGCATCGAGGTTGATGAACTTGCTCGTTTTGCTGTTGACGAACACAACAAGAAAGAGAATTCACTTCTGGAATTCAGCAAGGTTGTGAAGGTGAAGGAGCAGGTGGTCGCTGGCACAGTTTATTACATAACTGTTGAGGTAACTGAGGGAGGTCAGAAGAAGGTTTATGAAGCCAAGATCTGGGTCAAGCAGTGGCAGAATTTTAAGCAGCTTCAAGAGTTCAAGCTTGCTGGTGATGGCTCTGTGGGCTCCTCTGCTTAGTTTGCTAGGGTCCTAAAATCCACGATGATATGCGTGGAATACGAATGGAGCAGAATCCTTTGCTTTAATGTGTAGTGTCTTTGATTTAAATAAATTCTGTAAGCTTATGGAGTATGTATGGATCCATTTATTATCATAATATCAATAGTTGTTGTGAATTATGTGGCTATTGCATAATATATGTATTTTCAGGAAGAAATATCATATACTTACCTTTCCTTTTCCTTCTTTAACCCTTGTGAATAAAATGATATCAAATTATAC

>cucumber_newGene_104 cucumber_newGene_104.1

TGAAAAAAAATAATGAGTCTAGACCCATGAAACAAACAAAATTGAAAATGATATTGACCATGTAGTGTTCGATTGAAGCTCATAGAGCAATGGGGAAATGATCGTATTCGACTGTTAACGAAGAACTAAAAGTGATATTCCATTATTTATCTTACAATTGATATCAATAACATTGAATGCTGATGGTACAATGATATCAAGCCCTTCCGCTCAGTGTCCATTCAGCCGTCGGTCCAACACCCGCAACCCTTACCCTTCGTCTGTTCACCAGCCTTCACAGTGACGTTCTCCGTTCAGGTTCACCAATCCTTTTGTGCCTAAACTTTGTCTAATTTTGCAGGAAACCCCAGAGCGTTTCCCCTAAGAAACTCCCAAAATAAGTTTACCCACTAATTTCCTCTCATTCAATTATCGTTTTTCTGCTGCATAGGTTGATTTTCAGTGGTGTTAATTGGTTTTCTTGGTGCTTTCCTTCATAAATGTGGATGTTAATTTCTAGTTTTACTAATCGTTTTGGAATTTAATGAGGGATGATGATTATTTAGGTTAAGAATTGTAACATTAGTCCCTATCGATAAGTAGAGAGTTTTGCATGGGTGACCCAAATTTGATTTCGCTAATTTAAAATTACCTGAATTCTATACATGATGAAAACTAACGTTATGCTTGCATCATAAAGCAACAACCAGCGTTAAATTGTTATTTGAGACTGCAGGTCTCAACCATAGCAAATTGATGAAACCCCGAGTGCGATTTCACTTTCAAGAAAAGTTGAGCCCACAATTTTAGCAATGGCCAAGAACTTTTATTTTTATAAAAGTTGCAATAGACCTTGAAATTGTGTTCCGGGTCTCATTGATTTGCGAATGGGCTTTAGGGAGATTTTATTGTTGGTTGGTGTTTTGTGATGTGAGGAAAAAAGAGTTAGTTTTGTTGTTTTTAGAATAGGGGTCATTGGACATCAGTGCAATGACATCTCGTCTGTATGAAAAAAAAATGGCCTGTTTGAATTACTTTTAAGGACTGTGACAATTATGTGGATTTGAACCTATTCAGAAAAAAAGAGGATTTAGGTGAAGGTTAAGACAAACTGAAAGGTACAAATTTCGTAATCCTACTTTCTCCCAAAGTAAATAGCACCAATAAAGAACTCGGTTGAATTTGAATTTGGGAGGCAGCTCACGTTGCTGATTGAAACTACACAAAAGAAAAGGATGGTTAGCAAGAACAAGGAAGCCATTTGCAATGGCAGAATTGGAAGCAAGGAAAGTTGAATATTCAAATACTGGCACACAAACCTTTGCTAATGGGGATTATAATTGAGGAATTCTCTTGGAAGACTTATTTTTATTAAGGATTTTGACTTTGAAGCATATGTTCGTGTCAGCTCCTGTCAAGTGATGACTGATGACTATTGTGATTGTGAGTAAGTAGGCTTCTGGGCCACTGGCTACATGCTTCTTGAATGTTTTATGCTTAATTGCTAAAAATATACTGTTAGAAAAATAACGTCACATAATATTAGAAAATACAAAACCTTATATCTAGGAGAGAATTTGTGCCCAATGAATTTCTTGTCTTCTTAAAACAAATGCTCTAGAGGGTGCTTGAGTTTTGAGAGTAATTTTCTCTCATGATAGAACAAGTTATCTTTCTTGTGAGAGGAACACCTTGTAGTGTTCACTAAATCTAACGAACCTAGATTCATTCAACAAATGTTCACTGAGCCATAAAGAGGTACACCTTAAAACAAATGTTCTCTAAATCTAACGAACCCAAGTTTGTGGAACAAAGCAAACTTTGATAAATTTATAATAGGAAGAAAGGTTTATGGGAGAAAAATGCTTATGAAAAAGGTTAAAAACCAATTTGAAGAAAGAACATAGTTATAGATTCATGACCATTTGGATAGTCTATTCCTTAAGTTGGTTAAAATTTGAAATGGCATCTATTCGTAAAAGAACGTGCTACCCATGGAGTTAAAAGGATGCAACCCTATACAAATGGTCATGTACAATTTATTCCATTTGTATACAATGTACATGGTGAAGTGGACCAACAATTTCAACCTTGGTAAAAGAAAAGGATTTATGTGAAGCTTAAGACAAACTGAAAGGTAAAAGATTCTTCTCCTACCCTCTCTCAACCACTAATAAAGATCCTAGTTGAATTTGAATTTGCGAGCTAGCTCATGTTGAGCAACAATAGAACGACTGTGAAGAAATAAACTATACAAGAGAAAATGGTAAGTATACAATTCTTTAAGCTTCCCTTCTTTTCTATGTTTATGTTCAAATCTTGTCTTCATCTTCTTTAGTTTTTTGTTTGTTGATTCAAATATTTGGGAAATGCGATTTTGGTTTTGAAACTGATGGAGATGAAATTAAGGAACCAAATGATGAGCGTTTTCCAAAGAAGACGCAACCATGATGTGAACCGAACAGCTTTTAGCTACTGGGGATTTATTTTGGTTGAGGATGGTTCTTTCTTTTATTATTATTATTATTATTTTTGTGGGTTTTGAACTCTCCCCCCTCTGAATAGATACACCTACTTGACTTGGCCAAAATTGGTAATTCTGATGGTTCCTTAGTTCATCATTCCTCTATGAAATTTTAGTTTTGGTTTTTGAGTTTAGAAGTTTTCGTTCTCTTAGGACTTCCAAATCTATCTTCATACTTGATTTTGATTGACATGGTTTGGATGAAGTAAAATAATTTTCAATAGTTTCTGGATGGTGGGGTGGTTAACAAAGTTTGTTTCATGTTTTCTCAATGTGCAATTGTGTTAATTCTGAACTTTGAATTTGTAGTCATAACTCAAACCATATACATATTAAGTGATGGATAGTATAGTTAACATGGACGTGGACGAGACTTTTGTTCGAGATTTGGAGTACAGAAACACGAACGTGTTTTGGCTATAAGAGTGACAGAAAAGTTGAGGTACAAGCATTGGACATCAAATTATTGAAATAGAAAATTGTTTTGAGGATAGAATTCAAACAATGATGTGAGAAGTGTGTAAACGAATTATTACCGGATTTATTAAGGGCCATGGGCATGTGTCGCTCGTATTATAAGTTTTTGTATTTTAATATTAATCTTGGAG

>cucumber_newGene_105 cucumber_newGene_105.1

TATATAGTCACCCTCGAAGGTATATAAACCTTTGGAAAATAGCTTTCTCTTTTTCTTTGTTTCCAAACTTCCGAACTTGACAGCTTGTTTCTGTTCAGTGTTCACTCTTCAATGGCCGCCGACAACAAACCCTCCGCCGGTGGCAAGAAGAGGAAGCAGCGTTTTCTCCCCCACAACAAGCCAGTAAAGAAGAAGGGTCATTACCCATTACGCCCGGGGGTTCAAGGTTTCTTTATCACCTGTGATGGCGGCCGAGAGCGCCAAGCCTCCATTGAAGCAATCAACGTTATTGACTCCTTTTTTGACGAGTTAGCTCTTGGAAAGAACTGTGGTGTGAAGCTGACAGAATTAGCTGATAAACCTTTAAACAAAAAAATTAAGTTTTCAGATTCTGAGTCTTCTAGTTCAGATGATGATGATGATGATAATGATAATGAGGAGGAGGAGGAGGAGAAGGGGGATGATGATGAAAATAAGAAGGTTCCTGAGGAAGAGTCTAAGTCTCCCAAGGAGCAAAATGAACCTTCTAATGAAAATGAAACAACTGACGTTGCCAATCCTCATCAAGAGGAGACTGTAGCTTCTGAAAATCAAACAGAGGAAAAATCGAATGAAAATGAAAGGGATATGAAGTGTCCTGAAGTTCCGTCAGAGGAAGTTGAGGAGCCACCAAATAAGAAACACTGCATTGAAGCAGATGCATCAAAAAGTATTATAAAAGAAAAGGAGAAGTCCATTGATCAACTGATTGAGGCTGAACTTCAAGAATTGGGAGACAAGACCAAGAGACGTTTTTTCAATCTTGATTCGGGATGTAATGGTGTTGTCTTTATCCAAATGCGGAAGAATGATGGAGAACCTGGCCCTGAAGCTGTTGTGCAGCATATGATGACATCTATTGCTTCAACAAGGAAACACATATCAAGGTTCATACTTAGAATTCTGCCAATTGAAGTGGCATGCTATGCTTCTGTAGAGGAGATCACGAGGGCAATAAAGCCTTTAATAGAAAAGAACTTTCCTGTTGAAAGTCAAAATCCTGTAAAGTTTGCAGTACTGTATGAGGCCCGTGCAAATTCTGGCATTGACAGAGCAACTATAATTAATACGGTAGCAAAAGCTGTTCCTGAGCCTCATAAAGTTGATTTGAACAATCCTGAAAAAACCATTGTGGTTGAGATTGTCAAGACAATTTGCTTGATTGGAGTAGTGGAGAAGTACAAGGAGTTGTCAAAGTACAATCTGAGGCAGCTTACATCAAGAAAGCAATAGGATAGGTTATTTATGTGTTTTTCAAGTCTGTTTCAGCTTTGGGCACTTTTTAACGTTAAAATGTATGCAACAAATTAGCTTATTAGTGTATGGGTTAGTTTAGAAAATTTTGGAACCAAACAATCATA

>cucumber_newGene_108 cucumber_newGene_108.1

CCAATTCCAATTCCAATTCCAATTCCACGGAGCTCAATCATCCCTTTCTTTTCCTCCTTTCTCCCACATTGTTCTTTGTTTTCTTCCTCTCAAGCTTTCTCCAGCTCCACGACCTCTTAACCATCTGATTGCTTAACTTTTCCCACAGTTTTTCGATCAATTGGGGTTTGCAGGAGGATTCAAGAAAAGGGTAGCAGCTGGTTTGCTGGAGTCTTCACTCTCTTAGTTTCCCTTCTTGCTTGACCTTTAGTGCTTGCTTTGTGGAGAGCTTGGACGATTAGTGTACGTGGACTGACGGAGTGGCTGTTGTGATGATTGATTGATGGGGTCTTACCTTTAGTCTCTCTGTGTTGATTTGAGAGGTTTTGTTGGAGAGTGATCGTGTGACTGAACGGGGTTCGTGAATGGAAGGTTTTCGTTTTGAAAATGGATGAGCTGTTAGAGATTGGTGCTGAGAGTTAGGAATTGGTAACGAGGCGGCGTTTTCTTTTCTGGGATGGATAGGATGGTTCAGGATCTTAGTAAGACTGGCGCCGTCGAGAGAGATGTCGAGCAGGCCATTACTGCTTTGAAGAAAGGAGCCTACTTGCTCAAGTATGGAAGAAGAGGGAAGCCAAAGTTTTGTCCTTTCCGCCTTTCCAATGATGAGTCTATTTTGATATGGTTCTCGGGGAAAGAGGAGAAGCACCTTAAGCTAAGCCACGTCTCTAGAATTATTTCTGGGCAACGGACTCCTATCTTTCAAAGATATCCGCGGCCTGAGAAGGAATGCCAATCATTTTCTCTAATATATAATGAGAGGTCGCTTGATTTGATATGCAAGGATAAAGATGAAACTGAGGTTTGGTTTAGTGGCTTAAAAGCATTAATCTCACGTATCCATAATCGAAAGTGGAGAACTGAATCACGGAGTGATGGGATGCTGTCTGAAGTAAATAGTCCTAGAACATTTACACAAAGAAGTTCTCCCTTGCATTCCCCATTTAGCAGCAATGACAGCGTGCAAAAGGAAGGTGGAGAGCCTATTAGACTTCACAGTCCCTATGGAAGTCCTCCAAAATATGGTCTGGATAAGGCATTATCAGATGTAATGTTGTATAATATTCCTCCTAAAGGTTTCTTCCACTCAGATTCTGCAAGTGGTTCAGTTCATTCTTTGTCATCTGGAGGTTCAGATAGCATGCATGGACACATGAAAGCAGTTCCAATGGATGCTTTTAGGGGTAGTCTCTCTAGTGCTGTCAGCTCATCAAGCCAAGGTTCAGGAAATGATGATTGTGATGCGTTGGGGGATGTTTTTATCTGGGGGGAAGGAACTGGTGAAGGTGTCCTTGGTGGTGGAACTCGTCGAGTTGGAAGTTCTTTTGATAGCAAAATTGATTCTTTGCTGCCAAAAGCCTTAGAGTCTGTAGTAGTACTTGATGTACAAAACATTGCCTGTGGTAATCGGCATGCTGCCTTGGTGACCAAACAAGGTGAAGTTTTCTCCTGGGGCGAAGAATATGGAGGCAGGCTTGGGCATGGAGGAGATTCTGATGCTCATCATCCAAAGCTTATAGATGCCCTGAGTAATTTAAACATTGAGCTAGTAGCCTGTGGTGAATATCATACGTGTGCTGTAACACTTTCTGGTGATCTGTATACATGGGGTGATGGCACCTATAATTCTGGCCTTCTTGGGCATGGAAAAGATGTAAGTCACTGGGTTCCAAAACGAGTAACTGGTCCCTTACAGGGCCTACACATATCGTCTATCTCTTGTGGACCTTGGCATACAGCACTGGTGACATCTGCTGGAAAATTATTTACATTTGGTGATGGCACATTTGGTGTTCTTGGTCATGGAGATCGAGACAGCATCTCAATACCTAGAGAAGTGGAGTCTCTCAAAGGTCTACGTACTGTCCGAGCTGCTTGCGGTGTTTGGCATACAGCTGCAGTTGTAGAAGTTATAATTGGAAATCCAAATTCGAATAACAACTCTTCAGGGAAGCTGTTTACTTGGGGGGATGGGGATAAAGGTCGGCTAGGGCATGGTGACAAAGAGGCAAGGCTTGTGCCTACTTGTGTTGCTGCTCTTGTTGAACCGAACTTTTGTCAAGTTGCTTGTGGACATAGTTTAACTGTAGCACTTACAAACTCTGGCCATATCTACACTATGGGAAGCCCTGTTTATGGTCAGCTAGGGAATCCTCAGGCAGATGGAAAGCTCCCAGCACGAGTTGAAGGAAAACTCTCAAAAAGCTTTGTGGAGGAGCTAGCCTGTGGTGCTTACCATGTTGCTGTTTTAACTTCTAGAACTGAGGTTTACACATGGGGCAAGGGGGCGAATGGTCGTTTAGGTCATGGCAGCACTGATGATAGAAATACTCCGACATTAGTTGAGGCTCTTAAAGATAAGCAAGTTAAAAGTATTGCTTGTGGCACCAATTTTACTGCTGCTATTTGCCTTCATAAGTGGGTTTCAGGTGTTGATCAATCCATGTGTTCTGGTTGCCGTCTACCATTCAACTTTAAAAGGAAACGCCACAATTGTTATAATTGTGGTCTTGTGTTTTGTCATTCTTGCAGCAGTAAGAAGTCTCTCAAGGCTTCAATGGCACCAAACCCCAACAAAGTTTATCGTGTTTGTGATAATTGCTATCATAAACTAAAAAAATTTATTGAAATGGACACTTCTTCCCAGTCTTCACTGAGCAGAAGAGGGAGCATTAATCAAGGATCAACGGAACCCGTGAACAAAAATGATAAGTTGGACTTGAAACCTCGTTCTCAGCTTGCTAGGCTTCCTTCTGTTGAATCATTCAAGCAGGTGGAAAGTCAGTTTTCAAAGAAAAACAAGAAACTAGAATTTAGCAGCAGTAGGGTCTCACCAATTCCTAGTGGAGGCTCTCAGCGGGGATCTCTTAATATCTCTAAATCTTTTAACCCAGTCTTTGGATCTTCAAAGAAGTTCTTTGCTGCATCTCTTCCTGGATCAAGAATTGTTTCACGAGCTACATCTCCAATATCTAGGCGTCCCAGCCCTCCACGTTCAACTACTCCTACTCCTACTCTTGGAGGACTTACCTCTCCAAGGATTGCTGTGAATGATGCCAAAAGCACAAACAACAGACTTAGCCAGGAGGTTATTAGATTAAGGGCTCAGGTTGAAAACCTTACACGCAAGGCACAACTTCAAGAAGTTGAACTAGAAAGAACAAGTAAACAGTTGAAGGAAGCAATATCAATAGCAAGTGAGGAGGCTGCAAGATGCAATGCAGCCAAAGAAGTGATCAAGTCACTAACTGCACAACTGAAGGAAATGGCTGAAAGGCTGCCAGTTGGAGCTGCTCGTAACATTAAATCAACTCTTGCCTCTTTCAGCTCCGGTCCACCCTTCAATCATTTGATCAACACTTTCATAGACCAATTAAGTGGTCAAGAAACATCCTTAGAGACAGACTCAAACAGTTCAAGTGTCCAGTTGCTTTCTAATGGATCAAGCACTGCAAATAACCAGTCTTCAACTCAAAGCAAATCGAGTCAATTGGATTCAGCAAAGAATGGAAATAGAATCAAAGAAACTGAATCTCGCCAAGAAGCAGAATGGGTCGAGCAAGATGAGCCTGGTGTATATATTACACTCACCTCACAACCAGGAGGTGCCAAGGATCTTAAGCGAGTTCGTTTCAGTCGAAAGCGGTTTACAGAGAAACAAGCAGAGCACTGGTGGGCAGAGAACCGTGCACGAGTTTACGAGCGATACAATGTACGGGTGATGGACAAGTCTAGTATAGGGATTGGAAGTGAAGAGTTGACTCATTGACTTGTGCAAACAGCCACAGAAATCAAATCATTTTGTATATGAAAAACAAAAAGGTTTGTTCAATTTCCAAGCTCTACTTAACTACTGGGTCATATTGACCATTGAGATTTAGATTGGAACTATTAGAGGGAAAATTAGATGAATTTGATGATTTGTTTTTCGAATGTCGATTTCATATTTCTTTCAGGTTTAGTGTTTTTCTTTTTTTAATTTCTTTTTCCATTCTTCCCCTGTATGGCTGTGTGGGGGTTATGAGAAATGTAAATACTACTAGTTGCTGTCTATTGATAAAATCAGTTGCAGTATAAATTCTAAAAGCATCTTCC

>cucumber_newGene_109 cucumber_newGene_109.2

CTTTACTATCTTCTGCATTTCAGAATCTTCTTTTTTTTTCTTCTTTCGATTGATACTATGTCCAGATTCTCTTTTCTTTCTCTTTTCTCTAAATTCGAGCACGATCGGAATATTGATATCGTTCACAAGTCTCAGATTCGAGTGCCAATAATAAGAGTTGTGAATTGCTTTGATTGGTGAAGGGTATATTATGAAGCTTTTCTTACACTATCACATTCCATCGCATTGCAAGGATGAAGAAGGCTTCTCGTACTCAAGAGGCATCAAAATCCAAATGTGCACCAGTTACAATCAAATCCAATTTCTAGTACTGCATCATCATCCAAGATTGTGGAGCCTTCAATGTCTCTTTGTGAGGAAATAACTAATGCGTCCATTCAAGAGAACGATGCATCTGAACCTATTTGTAATCCAAGTACAAGTATGGATTTCACTGTCAAGATCAGAAAAGTTGGTCGTATAAGATCGTATACATCCTTGTTGGTTGTTGGAGCAAGGGTGAGGTTGGCATCTATAACACACTGTTGGATAAATGTGTTGTTGATAACTTTGCATTGTGCTGTTAGGTGTGTTTTACCTTTGTAAAGTTTTATTTAGAGTGTAATTGTTATAATGGAAGTGACATA

>cucumber_newGene_11 cucumber_newGene_11.1

AGACGAACCAAAGCAACCCCTTCAATCCTGTTTCATTAAAATATTGGAGTGAATTACAAGAAGCAGGAGATAAAAGAGGGCTAGAGGGAAACCCAGAAAACTTGAAACTCTAAAATGCATCCAAGTTTAATGCAATAGAGCTAGGCGAGTAATTTTTGAAGAGGGAAGATCGGCTTGTCCAAAGACCCGTTATGGCTTGTGTGTCTTCCCATACGGAGTCACCTTTTTTTTCTTCCTCCTTGAAAATCCTTCTGTTTCTTTCATTCCAAATGTTCCAAAAGGTAGCAGCCACCAGGTGTTGATTGATGATTTGCTTTTGTGTTTTACCTTTAGTTTTGAAAAGATCTTTGCATAAGGGAGTGGAATTTGGAAAGAGCATAGGTTTGCCCAGAATAACTTCAACTTTCTTCCAGAGCAATGTTGTGTAAGGGCAATGAGTGAAGAGATGTTGTCTATCTTCCTCAGCAGCTTTGCAGAGGATGCACCAAGAGGGCTTTATAGTCCACGTAGGTAGTCTTCTCTGTAGCATATCCATTGTATTGATACACTCATGTAGGAGAGTCCATACGAAGAATTTGCATCTTTTGGGGATGGTAGAATTCCATAAGTTGTTATAGATTTTCCCATCATCTTCGTTGGTCTCGTCTTGGTTATTTGGAAGTCGTGCAAGTTTGATAGAAGCCACAGTGAAGCTGCCATTGTTATTTAAAGTCCAACGAGGGTTGTCAACTCCAAAATCAGTCCAATTTGCAGGCACAGCAATATTGTCAATAAACTGGTACTGCTCACAGTCAGACTTGAATTCATTGAAGTTCTTTGCTGCTTCGTTGGTGAAAGTACCATGTATTCTGGGGTTTCTTTCTATAAGCCATTTTCCTTCAGCTTGAACTACAGTCTGGATTGTAAATGGAATACCTTCTTCATCGACAATCATAACAAAAGCAGGTATAAAGCCCGAGTAGTTGTATTTGATTTTGATATCCGCCTCAATGAGATCGAACCTGTCTCTGGTTGCTTTTGCAACTTCAACAAATCTGCCACATGCTTCTCCGATTTGATAAAAAGACGAATAGTTCCATGCATGAAGGGTGATACCTTTGAATTTAACCCATCCACCATAACTTGACAAGAGTTTTGTGGAAGCATGTTTAGCTATATTCCAAGCCTCAAATTTGACGTAGAACTTTCCAACCGTAGTCCATGTTGGCTTTTTGGCCCTTAATCTCAAATTAATTTATTTTAATTATTCAATTAATTTATGTTTT

>cucumber_newGene_110 cucumber_newGene_110.1

ATTTGTTCTCTTTTTATCGTTTTTCCTTTTCATGGTTTTCTCTGTCCTTCAATCTCCACCGTTCTTGTTTTCTCCTATTCGCTACATCTCTTCTCCTCGTCTTTTTCTTCCTCTTCTCCTCCGTCTCTTCTTGTTCAGGTTCATTTACTGCATATGAAATAGATGCAAATGACCATTTCAAATATTGTTTCATGACTATTGCATCATCAATTGAAGGTTGGAGATATTGTAGACCAAATATAGCAGTTGATGGGACATTTTTAAAGTGTAAGTATGGTGAAACATTGTTAAGGCAGCAACTATGGATGGTAATAGTAAAATTTTCCCTCTTGTATTTAGTATAGTAGATTTAGAGAATGATGCTTCTTGGAAATGGTTTTTT

>cucumber_newGene_111 cucumber_newGene_111.3

GTTTCAATTCAATTTTGTTCATCCCGAGCGCCAAACACTTGCTACAACTCAAGAAAAAGGATAAAACATCAGAGAAGAGTTGATAGTAGAAAGAATCGGGTACATCATTCTTCAACTTTTGAATTTCATTTCTCTCATTCTTCTTTCGAAGTTTTCTTGAAAAATGGAGATTGGATCGGGTGCCATTGCCTCTTCCTCCAAAGAGCACCTTAAGATCTTTCACGAATGGTTCGGTTTGGCCGATTCAGATGGAGATGGTCGTGTTACTGGAAATGATGCCATACAGTTCTTCTCCATGTCTCATCTTTCTCGCGCCGAGCTCAAACAGGTTTGGGCTGTTGCAGACTCCAAACGACAGGGATATTTAGGGTTCAACGAGTTCGTTACTGCCATGCAGCTTATTTCTTTAGCACAAGCAGGGTATGACCTGGACTCAGATATCCTCAAGAAAGCAGCTGGCATGGAGGAAATTAAACTTCCAGTGCTAGAAGGCTTGGACGCTTTAGCTGTTAAGACTAAGAGATTAGCAATATCTAGTCAACATGAGACAAATGGAACTTTTCAACCTATGCCCCCACCATCAACTCCATGGTTTGCTACAAAATCTGGGAGCAAGATATCCCATACTGCGGTTACATCAATCATTGACGGTTTGAAGAAACTGTACAATGAAAAGCTGAAGCCTCTCGAAGCTACCTATCGGTTTAATGATTTTGTGTCCCCATTCCTGACAAGCAGTGATTTTGATGCTAAGCCAATGGTAATGTTGTTGGGCCAATACTCGACTGGAAAGACAACATTTATCAAACATTTGTTAAAATGTAATTATCCCGGAGCTCACATAGGACCTGAGCCAACAACTGATAGATTTGTAGTAGTAATGTCTGGACCAGATGAGAGAAGTGTCCCAGGAAACACTATTGCTGTTCAAGCAGACATGCCATTCTCTGGTTTGACAACCTTTGGTGGAGCGTTCTTGTCAAAGTTCGAATGTTCTCAAATGCCGCATCCTCTCCTTGATCAAATTACATTTGTGGATACTCCTGGGGTTTTATCTGGGGAGAAGCAACGCACACAGAGAAGCTATGATTTCACAGGGGTCATATCATGGTTTGCAGCGAAATGTGATCTCATCCTTCTTTTGTTTGACCCCCATAAACTGGATATCAGCGATGAGTTCAAGCGTGTTATAGGATCTCTACGTGGTCAAGATGATAAGATTCGTGTTGTTCTCAATAAAGCAGATCAAGTTGATACTCAACAATTAATGAGAGTATATGGAGCATTGATGTGGTCTCTTGGTAAAGTGTTGAATACTCCAGAAGTTGTCCGTGTTTATATTGGGTCATTTAACGACAAGCCTGTTAACGAAGCATCTGTTGGCCCAATTGGTCGGGATCTTTTTGTGAAGGAACAAGATGACCTCTTAGCAGACTTGATTGATATTCCAAAGAAAGCATGTGATCGTCGGATTAATGAATTTGTAAAACGCGCTAGAGCGGCTAAGATTCATGCCTATATCATGAGCCATCTTAAAAAGGAGATGCCTGCAATGATGGGCAAAGCTAAAACTCAACAGCGGCTTATTGATAATCTTGAAGATGAATTTGGAAAGGTTCAAAGGGAGTACCACTTACCGGCCGGGGATTTCCCAAATGTGGAGCATTTTAGAGAGGTACTAAATGGGTATAGTATTGATAAATTTGAGAAATTGAAGCCTAAAATGATTCAAGCAGTGGATGACATGCTTGGCTATGACATACCAGAGCTTTTGAAGAACTTCAAAAATCCTTATGAGTAATATATATAATAACAATATTATATAGTAATCTCAATCTCTGTTATTGCATTGCTACTTGCAATGAATATTGATGATGATGATGCAAAGTTTTGTTAGAATTCAAAATTTTGGGGACTTTTTTTTATCCATTTTTCCTTTAATTAATGCTCAAATGTATGTGTATT

>cucumber_newGene_112 cucumber_newGene_112.2

AAAAGGGTGATTTATTTGGTTAAATTAGAAGATAGTGTGGGTGTGTTATTAAGGTTGTATTGGAAAGGAAGAAAAAGAAAAATTACTCTTATTCTTTTTCTCCTTTTTGGCAGTTGCCGCAAGCCCTCCACCTTCAACCAGTCACCCACCGTCGGCTAAAAGTTCTATCCACGCTTCAACTTTGTTCACGTCTGTCGAGTTCAACCATGTGAGTTCTTCAAGTCCCACTTCAGCCGTCGAGTGTCAACCTTGTTTTGGCGCACCGCTCATGTCTGCTGTTTGAACTTTGTGCCCCTCACCGCCACTGATGGTGGCTCTTTAATGTCCTATGTTATTGCGAGCCATCGCGAAAACTGTAATTTGTGCATTTTTATATGCAGGATGACGAAAAAGAAGAAATGCAGCGAAGATAATGCAAGAACTCCATGATAAGAAGTTGTCTAGTGATTCGTAAGGCAAGATGCGATCAATCATTATGGAAGATAATTCGCTTAAAGACAAGAGTTGTAGTTGAAGAAGTCGATGTGACAAATAACAGCTACTTTATGCGCTAATATTTGTAGGCAAGGACGAATGTGCAAGCAAAGGAAGAATGAATATTCATCATAGTGTAATTAATTTACCGCCTAATTATGTCTATAAGTACCTCCAACAGTACCATGAACAAGTATTAAGAAGAGGTCATCCCTAAAAAAGCTCAGTGAAAGGACAAGTTTTAAATTCCTTCACCATTTGTAGTAGTAGTTCTTCCTCCACCTCTTCTTCGATCAACATCTGAGGGAGAGTTTAGAGAGATTAGAAATACAAGTTATTGTGGTATTTTGAAGGAAAAAAAGTCATTGATAGTGAAAGATTTTCC

>cucumber_newGene_14 cucumber_newGene_14.2

TCCAAATTCTCTGAAAACTACAAAGGTCTTCAATTGTTGGGCTGAGAGCTTAATCCTCCCCTTCGTTTTCGGAGGAGGAACTGGAGAAAATCTAGCCCTTCTTTTTCTTCCCCATTTCAACGCAAAGTAAGGAACTTTCATTTTTCAAACTCTTTTTTTTCCCCCAAATTTTGATGGGTGTTTCTGTTTGGATGCTGGGAAAATTCGAGAGGGAAAAAGTGGCTCCAACTTTCTGAAGCCGGAATTTTTGTGGGCCCTCATTGATCGCGAGGCCTGTCTCTCGGAATCCCAACACGATCTCGAGAATTTATGAGCACGATATCCCTTTAACCGATACTTTGGTTGAAGGTGTGAGATGCATGGATCCTACTATCACGTTAATTTTGCTTCTGATATTCTTCAGCATAGCATGTGCTTCAACCACTGAAAAAAATTTTGAACACTGTGAAAGGGTTGTAAAGGATTGGGCCTTATCTTCTCTTCATAAAGGTATCAACAATGATAAACATACACTAAAAGATTTACTGTTCTTTCTACATGTTCCAAGAACAGGAGGGCGATCGTACTATCACTGTTTCTTGAAAAAGTTGTACCCAAGCTCTCAAGAGTGTCCCCGTTCTTATGATAAGCTACGGTTTGATCCCAGCAAGACGAAGTGCAGATTGTTAGCTACTCATGATGATTATAGCATGATGGAAAAACTACAGAAGGAGAGGACTTCGGTGGTGACAATTGTCAGGAATCCAGTTGACCGGGTTCTTGGTTCATATGAATTTTCAGTTGAGGTAGCAGCTAGATCTTTGGTACATCCTGACCTAGCTTCTGCAACCAAAATGTCCAAACGTGTGCGTGCAAAGACAAATGGAGTGAGCACACTGGATATTTGGCCGTGGAAGTATTTGGTTCCATGGATGAGGGGAGACCTATTTACTCGAGTATGTGTACTGTTGGCTGATCTTGTGCTTCTTAATGATTATTTTCTTTTAACTCAAGTGGAGTGAAGATAAATATAATTAGATCTTGTGCATTTTAATAATGTTTAGTACTTTCTTGTTTGGTCGGGGAGCTCTATTTCTTTCTCGTTTGGTTTTCATCGATAGTTGATTAATAGGGAAACAACGGAGGTTGCCACTTCTCTGTCTTTAATTGCAGAGTTCAGGTTTTTAGGCTTGGGAGAAGGGATATTGATTTTGGAGTCTCGACTCCTTAGAGGGTTTCTCTTGCAAGTCTTTTGGCATTTGCTGGATCCCTCTCTCGTTAGTAAGTCTGTCTTGGTTGCTTTGTGGAGAAGATTTTGAAGAAAGTCAACTTCTTTTCTTGGCAAGTTTTTCTCAGTTTTGTGAATTGTATGGACTGATTTTTGAGGAAGTTAACCTTTGGGTATTGGTTTTGAAGACCTTCTGTTGTTGCTCTTTAGGCAACATTATACTCAGTTGGACCCCTTTTCTTTAATGGGGGGTTTCGTGGGTTTGGGTTTTTGAATTATTCCATTTCTTCTCCATAAAAGTAGTTGTTTCTTTATATATAAAGCTTTGGAGCTGTTTTAGTATGAATCAGAATTCAATAAGTTAAGCAAGTTGTACTCATAGTTTGAAGCCAAACCCGTCTGGAACTTTGTTTTTCGGCTTGCTTTAATGGATGATCT

>cucumber_newGene_15 cucumber_newGene_15.2

CTTTTGCGGGCCGAGTAGCTGCAAACTTAGAAGAAAAAAAGGCCATTGAGGTTAAAGTAATAATTTCTCATATAGGTAAAGGGTCAGAACTGATAAGTACATTGGGCCCTTTGTGCGATTCTTCCCTATTTGGTCAGAATATGGTTATCTACATTTCAATGGTCAGTTAGGCCATTTTCAAACTGTGCAAAACCAAAACCATGTGAACCGTCGATCTCGCTCCACTTGGAGCATTTCATTTTGTGAAGGACTACTGCTACACTCGAAGTGAAGGAGTACTGCTAAACTCGAGTGTAGCAGTTCAGGCAGTGATCGGAGACCTTGGTAAACGAATTTATCTTCCTTTGGATCTTCTCAGGAAATGATTACACCAATAGGAGTAGCATTTGCAGTGGGTTTTCTTGGATGGGTTTATCAATCACTGAAACCTTCACCTCCAAAGATATGTGGATCAGAAAATGGCCCTCCAGTGACTTCACCTAGAGTAATGCTCAATGATGGAAGGCATTTGGCCTACAGAATATTTGGAGTTTCAAATGAAGAGGCTGAATACAAAATCATTATGTGCCATGGCTTCAATAGCTCCAAAGATATGTACTTACCTGCCTCTCAAGAATTTATGGATGAACTCAAGATATGTATAGTATTATATGACAGAGCTGGCTATGGAGAGAGTGACCCGTATCCATCACGTTCGGTGAAGTCTGAAGCATTTGATATTCAAGAATTAGCAGACAAATTGCACCTTGGCACAAAATTTTATGTAATTGGATGTTCAATTGGAGCATCGGGTATTTGGAGCTGTCTGAAATACATTCCACAAAGACTTTTAGGAGCGTCCCTAGTGGTTCCCTTTGCGAATTTCTGGTGGCCTTCAGTTCCTTCAGCTTTATCACGACAGGCTTTTAGGAAGCTTCCTCAATCTTACCAACGGACGTTTCAGATTGCACATTACACACCTTGGTTATACCACTGGTGGATTACACAAAAATGGTTCCCAACATTGGGAGCTGATGGCATGTTCAGTGATTCAGATCTAGAGATATTAAAGAGACTGTCCGGAGGCTTAAATCATAACCCGGAAAAAGTGGCGCAACAAGGTGAACATGAATCACTGAACCGAGACATACTGGCTGTTTTGGGAAGAAAATGGGAGTTTGATCCCATCATCGACGTGAACAATCCATTCCCTGATAACAATGGCTCTGTTCATATTTGGCAAGGTTGCGAAGACCGTGTTGTTGCTCTTGAGTTTAATCGTTTTATAGCAGAGAAACTTCCATGGATTCAGTATCATGAAGTTCCTGATGGTGGACATCTAATAATTCATGATGTTGAAAAATGCGAAGCTATAATAAGGGCACTTTTGGCCAGATGAGTCCATCCTTGTGGGGTGACACTGCCATTGTGTACTGTACTGTTGTATTTTATTAGTTGCATTATATCCCACGATAAATTTCCATTCAGTAGTGCCAACAAACTGATCCTCCAGAAGATTGATGTATTCTGAATTGCAATTTATTTTGCTGCTGCTGATTGTATCATGTACCTGGACTGGGCTTAGAACAGCTTTGTTAAGGTGATAGATTATATTCAAAATCAATCTTTGACTTTTATGCTTTTGAAATATAAACTTGATTTGAG

>cucumber_newGene_16 cucumber_newGene_16.1

CTTTTGGAGCTTTTTATCTTCTTCCTTACGAGGACCGTCGCTTTTCTTCTTGGAATTTCTTTTCGACCGCTCGTTCACCTCCATCGTCGATGCCCACCACCAGCATGTCTCCGCCGCCTCACTCTTCTTCCCACGGCTGGATCCCCACTACTCATCAGTGGGTTTTGGTGTTTAATCATGGGATTATGAAGAAAAGAAGACACAGTGAATTGCAGGAGGATTGCGTCCACAAGCTTTACGATAGAAAGCTTTTTGGGAGATTAAATATCCCTAGGCGAGGAACCACATCATCACGCGTTGAAGATTCACTAGCAGGCGAATGGTAGTTGCAGTTGATGTTACTTGACATGATGCAATCGGCACTAACATGAACTGAAGAATAGAAACTTCCCTCCTAAAGTAGTCTATAAAAAGGGCTTCTTACCATGTAAAACTTGTCTAGTCTAAATGAACCAAAATGTAGAGAGCTCCATCAGAGTCGGTGGAAAGGACTAGCCTTTTTGCCTTCGGTAAAGAATACTTCATCTCTTTTTCGATCAACCTTATAAGTGAGAGCTTAGAGTAAGATTAGACAGAGAAACTACAACTTTTTTTTCTTCTTAACTTGTAAAAGAACTAAAGGATGCTTGCCTGCAAGTCAGCTCACATCTCTTGTTTTTTATTTTTGTACTTTGTAATGTATGAGTTTCATATTATACATGGTCGAAAGGCCTACATTCTTT

>cucumber_newGene_18 cucumber_newGene_18.3

TTTTCATAATGTTATGCAAAAAGTGTTGATTGTTCATTTGGCTGGCAATACTATGAGCTGATTAATTATATGAATTCATAGGCTGCCACATCTTTTCTGTTCTTTTTATTTGGGAAAAGGATTATATATGATTGCATCTATGGAATATTTCTAATATTACTTTTGGCATTACTTTGTGAATAAAAGATTCCTTGCCTGGTAATCTAATTGGGATATTGATAACTTGCTTTATTTGTCTGCCCTTGTTCTCGGAGTAAAGTACGCCATTGAATTCTTCTTCAAGGTGGGGGGGGCTCCGTTTCCGTGATTTATAAATTAAGCAGGAATTGATGTTGTTCACTTTGTGAAGTTGCATTGTGTACTGTATGCATATACGAGAAATTGTTATTAGAAGTACATCTCATTTTGCAAAATACTTTAATCATGGCTATTGAAACTTCAACCTGAAATTCTCCTTGATTTGATCTTGGATATACTTTGTTCCTTTGTGCCGATCTTAATTTACTTCTAAACATCAACAGAGGATATTATAAGTTGATTCAGTAAAACCAACAGAAATATAGACCCTTGTGGAAACCCAATGCTTTCTTCCACCCATATTCATTACTAATTATATGATCTTTATTTTTCTTAACATATATGTATAATTAAGGCTTTAATATAATCACCATCATATATAGCTCTCATCCAAAATCTCATATGCTAAAGTATCCTTTTTGTTTTCTTGAAAGACTTGTGTTCCTAGATCTTGGAGTATGGTTCTTAAATTAGTTTTATTTAATGATTTCAAAACTAATTCATACACATCTGATCAAGTTTTACTATTACATCGAGATGACACAAAAATCATCTGCACAACGTGATTGTGTCGATGAGGTTGCTGAGGTTGATGGATTAAGAAGTGAGGGACCATGGTCAGAAAAGAGTGAAACTATGTTTGTAGACTTAATGGATGAAGAGGTTGCAAAAGGCAATCGACCAACTACAACATTTACAAAGACTAGTTGGAATTATATAAGAAGCCAACCGAATACTAGTATGGGATATAATTATTCTTACGATCAATTGAAAAAAAGTTCAATAAACTGAGACAAATTTGCAAAGACTTCAAAAAGATATTGAGTGATATGACAGGAAATGGTTGAGATCCATTATTAGGCTCCATCAATCTTGAAGAGGAGCAATGAAACGAGCTTTTTAAGGTGAATAAGAGAGCTAAAAAGTTTAGAAAAAGTGGTTGCCCACATTATGAAAAGCTTATGAGGATTTTTGGAGATACTACTGCAACAGGTGTAAATGCTTGTCCATCAACAAGACTTATTTTAGATTCTGAAAATGAAAATGAAAGTGAAAATGATGTTGAAGAGAATGATAAATGAAAAGGCAGAAAAAGAGTTCGAAGAACTAGGAAAGACGAATTCGACAGTTTCTTTTCAACATTCATAAATGTATATGCAGAGAACGCCAAAAGAAAAAATGACATCCTTGAGAAAAAATCTATTGGTTGTACATCTAGTCAAGTTGATGAGAGCCACACATCAACTAGGAAAGATGATCTCATGAGTTGATGAAATGTTTGAACGTTTTAAATACAATGGAAGATGTTGATGGAGAGGCTTATTCTAAAATATTGAAACTCTTGCACGATGATTTTGCTTGGAGAAAAATATTTTTGCGAATGCCCGATTCAAGGAGGAGAGATTTCATAAATAGTCTCTAGATTGACCTTAGGGATGTTTAGCCTTAGGGATGGATTGTGTATGTTTGTACCTTATGTGTTATTTACTTTTTTAATTTTCTCATGATTATGAATTATTTTTTTTAACATGGCTACGTTGTTTTTGGGATGAAATGAATTTGTACGTTTTTTGTCTTTGATTTAGAGATATACTTGTGAATAGAAATGGATGTGATTAAAGTCAATCATTTTGAGGATTTTGATTCTGACGACGATGATGATATATGCATCATTTTTACTTTATTACACATGAGCTTGCACAAGATACATTCTTCTAAACAACCATGTAGAACCTCTGCACTAAGAGAACATGATTATGTGATCGAGTTGTTAAATGAAAACGATAGTAGATGTTTTGATTGTTTTAGGATGAAAGGAGTTACATTCGTAAGGTTTTGTGAATATTTAAAATCCAAGATAAATTTGAAAGCATCTAGGTATCTCACTGTTCAAGAGAAAGTAACTATATTCTTATTGATCATATCATATAATGAAAGCAATCGTATAACAGCAGAAAGATTTCAACATCCAGGTCATACTATTTCTCTAGCTTTTAATCGGGTTTTGAGAAAGGTTTGCAAGCTTGGACTAGAAATTATTAGTCCAACCAATATGTACACTATACCAATGGAGATTATATCAAATTCAAAATATTACCCTTTCTTTAAGGATTGTATTGGTGCTATTGATGGCACTCACGTTCCGACAAGTATTCCCCAAAATGAGCAAATACCATTTCGTAGAAGAAAAACTAACACAACATGGAATATAATGTGGGGCAATACTATCTTGTCGATTCAAGATATTCAAATATGCCAAGATTTTTAGCACCATTTCGTGGTCAAAGATATTATTTACGAGATTTTAAAGAAATGAGACATCGCCCTCGAGGTAAAGAAGAAGTGTTTAACTATGACATTCGTCACTGTGAAATACCATTGAACGTTGTTTTGGTGTGCTGAAGGCTCAATTTCCTATTCTTAAGCAAATGTCTCCATACCCGGTCAAGACACAAAAATATATACCGATAGCATGTTGCACAATTCACAATTATATTAGATTGCATGATCGTCATGATGATCTATTCAATGACTTTAGCAATGAATCAATGATCGTTGAACATATAGACAATTTGTCAGAAAATTTGCAGAGCGATATAATTGAATTAGATGTGAGTCGACATCATCTATGAGAAATTGCCAGAGTGAGAGATGGTATTGCCAATCAAATTTGGACAACATTTGAAGGATAGATGCAATTTTATGTTCATGTTATTCGTTTTATTTGTGTACTAGAACTTCTTTTATATTAAATGACACAAACTTTTTATAATT

>cucumber_newGene_19 cucumber_newGene_19.1

ACTGCTCAAAGGATATTAGAACTTGAAGACTATTAATATACTGTTAAGGTTCCTCTTCCTCCAAAAGTTTCTCTCAATCTTCTGATAAGACTCACGATTCATCACCGTCGGAATGACAACTCCAACAGTCAATACTTCACAGAACCTACCGGAAACTCAAGAAAATCACAATTCCAGTCAGTCTGATACCTCTGCCCAAACTTCTTTCAATCAGAATCAAGGATATCTAAATCCTTACTTTCTTCATCACAATGATAATACAAGTTTGGTGCTGGTTACAAAACAATTGACAGAAGAAAATTATATCTCTTGGAGCCGAGCAATGACCATTGGTCTCTCAGTGAAGAACAAGATTGGATTTGTTGACGAAACTATTGCCAAGCCAACCGGTGATCTTCTTCCAGCTTGGATCAAAAATAATAACATCGTTATCTCTTGAATACTGAATTCAGTGTCCAAACCCATACCAGCAAGCATCTTATTTTCAGACTCAGCAAGAGCAATATGGATTGACATTAAAGAAAGATTTCAAAAGAAGAACGCGCCAAGAATTTTTCATTGAAAACGATCCCTTGCAACCTTTGAGCAAAACCAAGATACCATTGGTATGTATTATACTAAATTCAAAAATCTTATTGATGAGTTGTATACATATAGACCAGGTTGCACTTGCAGTTATGATGGTGTACGAGAAATGACGGATTTCCTTCAAATGGAATATCTCATGGACTTTCTTATGGGATTAAATGAGAACTTCTCTCAAGCACGGGGTCAACTTCTTCTCATGGATCCTCTACCATCAACTAGTCGAGCCTTTTCTCTTCTTCTCCAAGAAGAACAACAAAGGTCAATTGGATCTCTTTCTTCTACAGCACCAACAATGGCCTTTGTGGTATCCTCTAACTCATCCAAAAATGGAACCAACAATCGACAAAGGAGAGAAAAACCCATGTGCACCCACTGCAACACTCCTGGACACAGTAGATAAATGTTATAAGCTTCATGGATATCCTCTTGGATACAAGACCAAACAACAGCAACAACGAAACAGTAACGTTGTTAATTCAGTAGCTACTCGAAACAGTGAGAATATCTCTCAAGACACCACACAGAACAGTCAAATGGTGAATAATATCAGCATTGCAGACACATTAATCCAATGTCAAAACCTTCTCAATCAGCTTCAGTCCCAAATCAATGCCTCCAACCAAATAGCTACCTCACATATAGCAGGTACTTCTTATTCATTCCCTTTATGGATAATTGATTCTAG

>cucumber_newGene_2 cucumber_newGene_2.1

GAGGTGGAAGCCGAGAAACGAATCGTGAAGGCCTCTGTTATCTTATCTCAACTCTTTTCGGAGTTTCTTTCTTCCATTTTCATCACTTCAAACATTTCTTCACATTCCCCAAATCTCTTCATTCTTCTTCCATTTGGGTCAACCGACGCCGTTCCCGGCTGATGGACGCCAACAATCAAGCCCAATCCTCTCCTTACCCTCCTCCTCAGGCCCCTGTTCCGGCTCCTCCCTTCCATCACCTCCTTCAGCAGCAACAACAACAGCTTCAGATGTTCTGGTCCTTTCAACGTCAAGAAATCGAACAGGTTAACGATTTTAAAAACCACCAGCTTCCTCTTGCCCGCATTAAGAAGATCATGAAAGCCGACGAGGACGTTCGTATGATCTCTGCCGAGGCCCCCATTTTGTTCGCTAAGGCTTGTGAGCTTTTTATTCTTGAACTCACTATTAGGTCATGGCTCCACGCTGAAGAGAATAAGAGGCGGACATTGCAGAAGAATGATATTGCTGCTGCTATCACGAGGACCGATATCTTTGATTTCTTAGTTGATATTGTTCCTAGGGATGAGATCAAGGACGAGGCTGGGTTGGGCGGTATGGTTGGAGCTACTGCTAGTGGAGTTCCGTACTATTATCCGCCTATGGGTCAACCTGCGGGGGCTCCCGGGGGAATGATGATTGGGAGACCGGCAATGGATCCCACCGGAGTTTACGCTCAGCCGCCGTCCCAGGCGTGGCAATCCGTGTGGCAAACCGCTGCTGAGGATGGTTCTTACGCGAGTGGGGCCAGCAGCGGGCAGGGTAATCTTGATGGTCAGAGCTAAGCAATTCTTGTGTAGAAGATCGATCTGCTGTGTGGTGCAACAGAAAATAGAAAAACTGATGCGTTTTGCTGATGGCCAGGGCGTTAAACGAAATGTATTTTTCTTTTTCACGTTTCCTTCTTTTCGAACAGGGTTAACATTAGAAACGTGTAAAAACTTTAACTCGATGTCGTCGAATTGACATTACTTTTGTTAACTTGCTATTTCTACGTTCCTGATATTAATTGGCTTTTAACAATTTGTTTAAGATCAATTTTAACTAGCTGATCTGTTGAGCATTATGTTAGATCTTGGGCAGGCAGATAAAA

>cucumber_newGene_20 cucumber_newGene_20.1

CTAGAGCAACTACTAGAGAAGCAGCTGATCCAGCTGCCAGAATGTAAACGACCTGAGCAAGCAGGAAAGGTAGATGATCCCAATTACTGCAAGTATCATCGGGTCATCAGTCATCCAGTAGAGAAATGCTTCGTGCTGAAGGAGCTAATTCTAAGGTTGGCTCGTGAGAAAAGGATTGAGCTAGATTTGGAGGAAGTAGCTCAAACAAACCACGCTGAAGTGACGATAATGTCTGAGGCTTCTTCGTCGAGATTGATTTTTGAGCAAAGGAAAAGCTTGGTCCAGTTCGGGACCTTTGAGCCTATAGTTGTCCAATTCTTTCAGGAAATCTCATACGAGGATCCTCAAGGAGAGAAAAGACCAATCGAAGAAGACGATGAAGGGTGGATAGTGGTGACCCACCGAAAGAAAAGACAGTCGATCCCGACCCAAAGAGAGTCTCGCTCTTACCAAAACTATAGAAGAGGAAATAAGACTCAAAAGAATAAGAAAAAGAAGAAGACTCATAAGCTTAAGCTCGTACATAATGAAGACATGAATTTCTCTCGACCTCAACGCTTAGTGACCTTGGCAGACTTCCTTCCAAAAAGCTTCCTTTGTGATCATCAGGATGAAGACCCAGAAGTCGTTGCGTGTCATGCTATCAACACAACGGAGGAAGAAATAATCCCTCCAAGATCACTAGAAGGAGAGGGAGTATCAAAAGACCTTTCAAGGTTTAATGTAGAGGATCTATTATCACTTCCTCAAGAGACCAAAACCATCCTTATTGATGCATTGCTAAATTCAAGAGCATCAAGTTCGAGTACTCCGACTATGACATATGAGAGTGGTTCTTATTGTATGTCTATAGACTTCTCAGATGAGGATTTGTTGTTGGGATCTAAACTTCATAATAGACCTTTGTATGTTTCTGGATACGTTCGAGAACAGAGAGTCGATCGAATCCTCATTGATAATGGCTCAGCTGTCAACATAATGCCAAAGTCGACTATGTGGCAATTAGGCATCTTGATGGACGAGCTCTCAAATAGCAAGCTAGTAATTCAAGGTTTTAACCAAGGTAGCCAAAGAGCAATAGGCATGATACGGTTAGAACTCATAATTGGCGACCTAAAGGCCAGTGCATTGTTTCATGTCATAGACTCAAGGACTACTTACAAGTTGTTACTAGGGCGTCCTTGGATTCATGGAAACGGAGTAGTAACTTCAACACTGCATCAGTGCTTCAAATTTTATCAGGACGGTGTAAAGAAGGTTGAAGCCGACTCTAATCCATTCTCAGAAGCTGAGTCTCACTTCGCAGATGCAAAGTTTTATTCAAAGAATAATAATATTTTAGAAGTTTTGCCTGCAGAAACCCCTCTTACAAAAGGAGAAGATAATTCACAACTGAAATCACTCGCAACCACAGAACCACATGAAAGTGCGAGAACCTTTAACTCTGGAAAGGGTGAAACATACACTCGCAACACAAAGGGTATGATCTTGAAGGATGAAAATGCTGCAAACACACCAGTTTTGCGTTATGTCCCTCTGTCAAGGCGCAAGAAAGGCGAATCACCATTCATGGAGTCTCCAAAAGGTTTGAAAGTTGGTGACATCGAAATCATAAAAGAAAGCTTCACTACACCACTAACTAAGATAGCGAAGCAGGAGGTAAAGGTAGATCTGGTGGAAGCAAACTTGCCCCAAAGGCGAACGAAAGACGGATTCGACCCCAAGGCTTACAAATTGATGGCGAAAGCAGGTTATGACTTCACAGCTCACACCGAGTTTAAAAGCTTGGAAATTCATGACCGACCTGAGCTCTCCTCAACTCAAAAGAAGCTTCTACGGGAAGGACATTCTATACCCGTGTCGAGAAAAGGACTCGGATACAAGTCGCCAGAACCGATCCGTATAACTAAAAAGGGGAAGGAAAAAGTGGTTGACATCAATCATATAACTATAGAGGAGGATGACAATACTGACGTAAAAGAAGGTGATAATCAGAGAATCTCAGTATTTGATCGAATTAGACCATCTGTTGCACGTCCCGTAGTATTTGAAAGGCTAAGCATGACAGAGGCAGAAAGAGAAAGGCTCCAGTCAGTACCAAACCTTGAGAGACATTCGGTCTTTCGAAGGCTAACTACGACTCCCATAAAGGAAGAAAGCACATGCCATGCCTTGACAACTACAAGACCATCAGCCTTTGAAAGGCTAGGTGTGTCTAAAAAGAAAAATGTACAAGCACCCCGTGCTCCGATTTTTAATCATCTCGGGGATAAAGGATCACACGACAACATTGATTCCAACATAGATACAAAGAAGAAGGAACCAATGTCTCGTGTGAAAGTTTGGCGTCGAATTAAGCATACAGATGTCGATAATTATCGTAGTAAGAAGTTTCCTTGCGAGACAAAGGAAAATGGAGAAATTCATAGCAACGTTCCTTCTCGCATGAAAAGAAAAACTTTTGTTACTCTCAATACAAGTCAAGGTTCGTTAAAGGTAAAAAGACATGATGTTATACTAACGAATCCTGAAAAAGAAGGGTCGGAACAAGGGGAATGTGAAACTTCATGTCATCACATCACCATTATTGAGGAATCAGAGACTGGAACTCATGAAGAAGACGCAGAAAATGCCCCACAAAGTTTAGAGGATGGTGGTCAATCTACTGTAGACGAGCTAAAAGAGGTGAACCTTGGTACAATAGAGGAACCACGTCCGACTTTCATTAGTGCGTCCCTCTCTAACGAAGAGGTGGATAAATATATGAGTTTGCTCACCGAATACAGGGACATCTTTGCGTGGTCGTACAAGGAGATGCCAGGACTTGACCCAAAAGTAGCAGTCCATCATCTTGCAATTAAACCCGGATATCGACCGATTAAACAAGCACAACGACGTTTTCGACCGGAGCTTATCCCTCAAATCGAGGTGGAAGTCAACAAGTTGATCGAAGCAGGATTCATTCGCGAGGTCAAATATCCAACGTGGATAGCAAACATTGTCCCTGTTAGAAAAAAAAATGGACAACTTCGCGTTTGTGTAGACTTTCGCGATCTGAATAATGCATGCCCTAAAGATGATTTTCCCTTGCCCATCACCGAAATCATGGTTGATGCAACTACTGGACACGAGGCGTTGTCGTTTATGGATGGGTCGTCTGGATATAATCAAATACGGATGGCCCTCTCAGATGAAGAAATGACAGCTTTCAGAACTCCAAAGGGAATATACTGTTACAAGGTGATGCCCTTTGGATTGAAAAATGCCGGCGCTACTTATCAACGTGCCATGCAGAAAGTGTTTGATGATATGTTGCACAGGTATGTTGAATGTTATGTTGATGATCTTGTAGTCAAAACAAAGAGACGACAAGACCATTTGAAGGATCTAAAAGTCGTGTTTGATCGCTTGCGAAAATATCAGCTAAGGATGAACCCTCTCAAGTGTGCGTTTGGTGTAACTTCAGGAAAATTTCTTGGCTTCATTGTAAGGCATCGAGGGATCGAAATAGACCAGTCCAAGATTGATGCCATTCAGAAGATGTCAAGACCTAAAAGTTTGCATGACCTAAGAAGTCTCCAAGGACGACTGGCTTACATCCGAAGGTTCATCTCTAACTTGGCCGGTCGGTGTCAACCTTTTCAAAAGTTGATGAGAAAAGGAGAAAATTTTGTGTGGGATGAAGCTTGTCAGAACGCTTTTGATAGCATAAAGAAATACTTGCTTACTCCCCCAGTGCTGGGAGCTCCAGTACCTGACAAACCACTAATATTGTACATTGCTGCACAAGAAAGGTCCTTAGGAGCATTACTGGCACAAGAAGAGGTAAAGGGAAAGGAGCGTTCTCTCTACTATCTAAGCAGAACATTAATTGGGGCTGAAGTTAACTATTCTCCTATCGAAAAGATGTGTCTTGCACTTTTCTTTGCCATTGATAAGTTGAGGCATTATATGCAGGCCTTCACGGTTCATCTAGTGGCAAAAGCAGACCCTATAAAGTATGTTCTATCCAGGCCAATCATCGCTGGACGCTTAGCCAAATGGGCGGTTCTACTCCAACAATATGACATTGTCTATATTCCCCAAAAGGCGATAAAAGGACAAGCGCTAGCAGACTTTTTAGCAGACCACCCAATTCCTTCGGATTGGAAGTTATGTGATGACCTACCAGACGATGAGGTTTTCTTCACAGAAGTTATGGAACCTTGGACTATGTACTTCGATGGTGCAGCTCGAAGAAGTGGTGCGGGGGCAGGCATTGTCCTCATTTCTCCTGAGAAGCATATGTTGCCTTATAGCTTTGCACTTTCCGAATTGTGTTCAAACAATGTGGCTGAATATCAGGCTTTGATAATTGGCCTTCAAATAGCATTAGAAATCGGAGTGTCATTCATAGAGGTCTATGGTGATTCAAAATTGATAATCAATCAACTCTCGCTTCAATATGACGTGAAACATGAAGACTTAAAGCCATATTTTGCTTATGCTCGACAATTGATGGAAAAGTTTGATAATGTGATGTTAGAACATGTCCCTAGAGTAGAAAATAAGAGAGCGGATGCATTGGCAAATTTAGCCACGGCCTTGACCATGCCAGATGATGTAACTCTGAACATACCACTTTGTCAACGATGGATTATACCCCCAGTTAGGCCTGAATGTCAGGAAGTGAACATGGCAACATCCTATTTGATTGATGAAGAAGATTGGCGTCAACCCATCATAGAGTATCTTGAACATGGAAAGCTTCCAAAGGATTCTCGTCATAAAATTGAGATACGAAGAAGGGCAGCACACTTCATTTATTACAAGGGAACTTTATATCGCCGTTCTCTTGAAGGGCTCTTCCTTCGATGTCTCGGAAAGGAAGATTCGGTAAAAGCTCTAAAGGAAGTACATGCAGGTGTTTGTGGAGCACATCAATCGGGACCAAAGCTTCAATTCCAGCTAAGAAGAATGGGCTACTACTGGCCTAAGATGATCCAAGATTCAATAGACTATGTGAAGAAGTGTGAGCCTTGTCAATACCATGCAAACTTCATACACCAACCTCCAGAACCTCTTCATCCAACTGTGGCTTCTTGGCCTTTTGAGGCTTGGGGACTCGATCTGGTTGGCCCCATTACACCAAAATCATCAGCAGGACATTCTTATATCCTAGCAGCAACAGACTATTTTTCAAAATGGGCTGAGGCCATTTCATTGAGAGAAGCCAAGAAGGAGAACGTGGCAGACTTTATTCGAACACACATCATCTATCGATACGGTATTCCACATCGAATCGTGACGGATAATGGAAAGCAATTCTCCAATAGTATGATGGACAAGTTATGTGAAAAATTCAAATTCAAGCAATATAAGTCATCCATGTACAACGCAGCTGCGAATGGACTAGCAGAAGCATTCAACAAAACATTGTGTAATCTTTTAAAGAAAATTGTCTCCAAGTCAAAGAGGGATTGGCAAGAAAAGATCGGCGAGGCATTATGGGCTTATCGGACGACTCATCGCACCCCTACAGGGGTTACACCATATTCGCTTGTTTACGGTGTGGAGGCTGTCCTTCCTCTCGAAAGGGAAATTCCGTCACTAAGAATGGCAGTACAAGAGGGATTGACTACCGAAGATAATGTGAAGTTACGTCTTCAAGAATTAGAAGCACTTGACGAAAAGCGATTAGAGGCTCAGCAAGCATTGGAATGTTATCAAGCGAGAATGTCCAAAGCTTTTGATAAACACGTTAAACCTCGCTCCTTTCAAGTCGGTGATCTAGTACTTGCCGTAAGGAGACCGATCATCACAACAAGGCATACAGGAAATAAGTTCACACCTAAATGGGATGGACCTTACATTGTTAAAGAAGTTTATACAAATGGCGCATACAAGATCGTTGATCAAGACGGACTACGAATTGGCCCAATCAATGGTAAATTTCTTAAAAAATTTTATGCTTAATTTTGTAAGGTAAAAAAAAAAAAAGTTGAACTACGTTATGACTTGATCCCTATATTATAAAGGGTACGTAGGCAGCTTAAGGAAAACCTTAAGCCCAGTCCAGCAAAAAAAGTTGAACTACGTTATGACTTGATCCCTATATTATAAAGGGTACGTAGG

>cucumber_newGene_21 cucumber_newGene_21.1

GTTCAAGTTGAGAGTTCGACAGAAACAAGTTATTTTGGAAGAAGTTCTAACGTGTCAAGAAATCGACTTCTTCTTCTTGTTGGCTTCTTGATCAAAATGGTGAGTATGCATCACAGTTACTATCAAGAACACTCTGTCAATCCTCGTGATTCTGCTGTTCGTTCCAACATACACCCGGTCTGAATGCGTGGTAAGCGGGTTAAGATTGTTCCTTCACGCCGTCCGTATGTTCGCCTTGCTGATCGAGGTAATAACAAAGTACCAGTTCCTTCATTTTTGAAAGGAAAAGGGCCTGTGACTTCGAGCAGCCTCAGAGTTTATCTGACTCTTCTAGTAGTTCATCAGATCCTTTGCCCCAACAGCATCCCTGGACCCCTCATTCTGGGCACATTTCGACTATTTGTCCATCTCATGATATTCCAACTGGGCCCACTGCTCCTTCCCCATCCATGGGTCGTGAATCTGTCCTTCCTCCCACTACAGCTCGGACATCTAATCCTCTATCTCCACCTCAGTCCACGACTTCACCCAAGCCCCAGGGTGTCACCATCAAAACTGGTAGACGTCGACTTCCACACAATATTCCTTCTGTTCCCATTGATAGCATATCTTTTCATCATGAAGAGAATGCTCAGAGGTGGAAGTATGTTGTACAACGACATTTGGCCGATGAATTGAATGTCTCTGATAAGCATCAGTCCTGTGTGACCATTATAGACTTGATTAGTCAAGTTTGGTCTATCTCGTACCATCCTGAATGTTAGGTCGTTCTATCCAAGACTCATTTGGGAAGTTATTGCCAATCTGCCATCTAACTTTGATGACCCTAGTAGCACTAATTATTAGACAATTCATTTTTGGCTTTTCTGTTGACTGGTGGCTCCCTTACTGTTTGGCCCCTGACGGGTATTCCTATTGCCTCCTTGAGTGTTAACTGTGCGATTCTTCACAAGATTGGCATTGCAAACTGGTTTCCTTCTTCTCATGCCTCCAATGTATCTGTTGCGCTTTCTAAGTTCTTGTTTTGGATTGGTACAGGCTCTACTGTTGATGCTAGTTTGTTTATTTACAATCAACTTTTACGTCATGTTGGTACCTTTGGTGTCAAGATTCTGATTGCTCTACCTCACTTCTTCTCTAGTTTGATTATCCATTTGCATCTTGATATTTTAAGGGACTTTGATGCCCCTGGCCCCCGCCCCCGCATGTTATCACTGAGTTATAGACTGTTTCAAGGCTCTCATGTCCCGATATTGAGCACATAGTTCATCCCAACCGCTCTGCCAATCCCTTTGTGTCTGATGACGTTTCTGCTTCCTCTGTTGGTGCTAAAATGGTTGTTGGTGACATTTCCCGCATTTTAAATTCTCTATTTGGTGAATCTTGGGCATTGAGCACTTCTATTTCATTACTGAGAAGGGTGGAAATTGATGCTCTCATTCGCTATCTAAAAATGTTTCTATCTTCATCTGAAAATGTTCCTCCTCCGTCTCCTTAGATACTTTCTTTGCTCAAAGGAGGAGAGAAGTGTGGGGTGTGTTGTTGTTTTGGGTATTGTGCTACTGTTTTTGCTTGATAGTATAAACGTGTACTGTGAAATTTTGGGTGAATTTTCGAACAAATATAATGGTTGGCTTCTTTCTTTTTGTAGTCTATGATTTGTTATTGAAAATGAATGAATGGGTGAACTCCTACAGTGTGTGTGGCTTCTGGCTTGTCTTGATGTTTGGCTTCAGT

>cucumber_newGene_22 cucumber_newGene_22.1

TATAAGAATAGGTGGCTACAATCACGTTCGACCTGTATTGCTCGAGCACTACTGTCATAGGCATGATTGCCCTCCGTTCAATCGACAACACATCATCGTTGTTCTGTCCAATCACCATTGTCGCTGTAGCTCTGGTACCGTCGTCGTCTACAAGGCAACGCTCCAAAAAAGTTAGGACTTTTCACATTTCAAGCTACTTGGGTGAAGAGTTTTCTTAACGATATTCTCCTTACACATGGAGCCAAAGATAGTAAGTGATGATCAAGACAAGGAGGTTGGTGGATTAGTTGTTAGAGGAGTTGATGAACAAATTGTAGAGAAGCCCAGAAAACAAAGGAAGTGGAGACCTACAATTATGTTTGATGTGACTCGTGTTCGAAGTGAAGATGTGCCTACTGAATTGAAGGATAAGATTTATACCATAGTTGAGGAATGAACCTGAACTTTTGAGATGACCCCCAGACATGTATCCTTGCATTAACGAAGGAGAGCGGGAAGAATTTGTTAGATCTAGAGTATGTCCACATTTTGAGGACAAAAGAAAAGTTCAACAAGAGAGAAGGAAGAAGAATAAGTATAACCATAAGCTTTCAAGAAAAGGTTATGCTAATCTTCGAGAGGAATTGAAAAATAACCCTTCAGAAGAAGGTGATCTTGATCGAGCTAGCATGTGGAAGAAGGCTCGTGTAGATAAGAAGAGAGAATATGACAATGAACACGTTCAAGAAATTATGAATTGTATAGATGAGATCTCAAAGACATATGTTGACAAAGAGCCATCTCCAAACGATGTTTTGACACAAGCTTTGGGTACAAAAGAGCTTAGTGGACTTTTACATGGAGTTGCCAGGTTTTTCACCCCAACTACTTACTTCCAATACAACAAAACACTCAAAGAAACGAAGTGAAGAAATTGAGAAGCTTAGTCAAGATAATAAAAAACTTCATTTGTGAGTTCAAGAATTATNNNNNNNNNNNNNNNNNNNNNNNNAACGTCTGCACATGGATCTTGCTCTAGACCAAGATTAGAATATGATATTCAATGTCAAAAAAATCAAAAAATGAGAAAATTAATATCACAACGAAGGTGGTTAAAGAGGAGGTGAATGTGGATGATGAACATAAGGATGCTATCGAGGTAATTAGTTAAGTTTTGGCAAAATTTAATGCATGAGTTTATGTGTAAGTCTTACGTAGTTTAGCATTATTGTTGGGTTTATGACTTTCTTAGGTTCCTAATGAAAAAGAAGATACTTTGTGAGTCTAATGTTAATCTGTCACTACCTTTGAAGACAATACTTAGATTTGCTGAAAAGGTAATGGACTAAGATTCAGGCATTCGTTACCAACTACCAATCTTATTGTTTGGTATCGGTAGGAAGACTTGTGTTCTCCACAAAGATATTATTGATTTCTGTAACATGTGAGATGTCAAGACTTTCACATTAGTGGCATATATGGTGTAAGTACTCATTCGTCAACTCTAGAATTTTTATATAAGTGTTCCTTAGGTATTTTGCATTCACAAGATGTTTCAAATTACATCTTCGTTGACCCATCCCTTATATTTGTTGGTCACAACACCCAAGAAGTTAGAGCTCGCAATTTATGTAGTAGATTAATGGCTTCAAAACAAAACAAACTCGTTCTGGCTCCTTTTAATTCCAGGTAAGTAATAAATATACTAATCAATTCATTAAATTGTATTGTTTCTTCTTCTGTTGATATTGGAAACATATAGCTCATACTTTAATGTGCTCTCCATTTAGGTTTGTTCATATTATGGTTCTTTAGGAAATAAAATAAGCTATAGATTATTTTTTTCTTTGAATTGAATCTTAGGATTTTAGATTATGGTAAGGCAATTTGCTTCTTTGTGTTCAATCTTAGGATTTTAAATCATATATTATGTTTTTTAAATTAAATTGTATTATTTCTTCTTCCATTGATATTGAGAAGCATAGCTCATTCTTTAACGTGCTCTCTGTTTAGTTTTTTTCATATTATGTTTCTTGGAAAACAAAATAAGCTATAGGTGATATATTACTTTGAATTGAAACTTAGGCCAACTCAGATCTCATGATCGGGTATCTCCTAACGATCCCCGCGGAGCTCCTTTTTAGTGGACGCTACTTGATTATGGGGGGATGAGACTCTACCCTAATCAATTAGCTTCTTTCTCCTTTTTAGTAGTCTCGCGTCTGAGAACACCTATCCGAACGGACTTATTCCCCTCATCTCGTGGCTTCATGGGGAACCCCCTCACTCAAGAGCACGCTTGACATCTGTCGCCCAGTGCTACCCGTAAGAGACGGCGGTGGGCTTCGGCTAACCTGTCCGCCTCTGGGTTGGGGAGTAACCCTCTTGTACGGGCCGGGGGGGTTATTGTTGTAGTATACTTTTGATTTCTAGAAACTTGTTTTGGGTTATGTTAAGGAAATTTGTTTCTTTGAATTGAATCTTAAAATTTTAGATCATATTATGTTTCTTTGAATTAAATTATATTGCTTCTTCTTGCATTGATATTGAAACACATAGCTCATATTTTAATGTGCTCTTCGTTTAGCTTTGATCATATTATGTTTCTTTAGGAAATAACATAAGCTATAGGTGATTTGTTTTTTTGAATTGAATTTTAGGATTTTAGATTATGTTAAGGCAATATAGAGGGAGGAGGGAGGGGTTCAACTTTTATTGTTGTAATTTACTTTTGATTTAGCATAGAGAAACTTGTTTTGGATTATATTAAGGCTATCTACTTGTGTTCTTTCTATGTAGTGGTTCTTGGGCACTTATGGCTATTAACGCATACAATGAGACAGTTTATAATATTGACTCACTTCGAACAACATCAAAAGTAGATATTAGATATGTAATTGACACGGCTATTACAATCTTTTTCTCTCAGAAAAATATTCAAACAACTCGAAAACAGTCAATATGGGAAATAGTGAAGTGTCCTTTGCACGTTGGAGTAGTTGAATATGGATACTATGTGATGAGATACATGAGAGATATAATCACTAATGGGAGCATAGTAGTCACAGATTCGATTGATACAAGTAGCCCATACAGTAAATTAGAGTTGGATGAAGTGCGCATGGAGCTTGCTGATTTTTTGGGTGGCCACATTCGATTTATGGTGAATATATTTTGGGTTAGATAGACAAGTTTTGTTGATACTGGATATAGGTGTGATATTTTTTTTGATATACTTGATATAGCTAGGTGAATTAGTTTAGTTGATATTTTGTTGATACACCTATAGTTAGGCTACACATAGATGGAAAGTTTAGGCTATTGTTAGAAGTAGGCTAACTATATGTATGTATACG

>cucumber_newGene_23 cucumber_newGene_23.1

AAAATCCAAAGCGTCCAATCAGCAAAATAGGGCCAAAGGAATGGACGGGCACGATTAGACTGCCATTTCGCCCTGCAAAAACTCCTTTTTTGATCTCGATCTTGTATTTCAGGGCTAGGGTTTGAGTTTTGGGCAAATTATTCAGAGATGGCAATGAAGCAATTCTTCTCCGAAATCAAGGGCTTGAAAGTTAAGGAGCTGCCTGCCTATGTGAAACCGATGCTCTCCATTGATGCTGCCAAGAAGGCTGTGGAGAGAAGTCTCGACAATTACAACGCCAAGTACATTCAAACTAGTTCAATTGATCCTCTCCTTCATGTCTGCTTTGGTGGTATGATCTTTTCGTACCTCGTTGCGCTTCCTGAGGAACGACGCCACCTTGAACATCAGCAACACGCCAAAGAACATGGCGGCCATTGATTGAGTTCTTTATATTTTGAGGGTGAAGGTGCCTCAAAGATGTTTCTGGTCAATAATTTGTTTCCTGAATGTTTTTGTATCTTAGTCTTACTGGCTGAAACTTGCAATTCTTTTCTTTTATACATGGTGCCAGAGGAAATAAACGTACTTTTCTGTGATTTCTAATCATATATTGGTAACCATTCTTGAGTCGCAGCACATGTTCATGGCTGATTTCTTTACTTTATTGCACATTATCCTTCCCCATACTTGTTTCTCT

>cucumber_newGene_24 cucumber_newGene_24.1

TCAAAATTAAGTCACATTTTCTTTGGACATCTTGAAATATCGATCATAAGCCAAATCAACGGTGTCACGTTCCATCAAAGCCTCCCTTCAATCTCTCACCAGCGTCCCTCCATGGATGAAAGCGATAGCTATAGCACATGGGACCTTTTTTCTCTGAAAAGAACGGTCATGGCTGAAGCAACGAGTTCCCTGTTTCCTCACTTTCACTGTTAATCACTGCGAAATGCCTTGAACGAGAGTTCATACATTTTCACTGAACTAGGCTATGGTTTTCGTTTTTCTTTAGCTTTTCACGAACAGGCGATGAAGCTAATTCTCTAAGCTAATGGAGCTGGTTACACATAGTGCTGCATCGTTGTTTTCATCTGCTTCATTTCTGTGTGTTTCAAATTTGAGTTTTCCTGAATGTAGACGCCGGAATTCGAAGCAGAATGTTGTTCGAGGTCGCAGAAGAAGTGATATCTGTTTCGATTTGGATGCTAATTGGATTCATTCGTCGTTGAATAGAGCGAGGAATCGTGGAGCGGAGGTGAGGACTTCAGTCGCCGTGGCGGAAGAGGTTGAGGTCTCCAATTTAAATCGGTTTGAAGTGTTTGATGGTACTCCTACGCCTTTCGGTGCCACAGCTCGAGAAGATGGGATTAATTTCGCTATCTATTCTGCCAATGCGGTTTCAGCTACTCTGTGTTTGATTCATCTTTCTGATTTACAGGAGAATAGAGTGACCCAGGAGATCACACTTGATCCCTTAACCAATAAAACTGGGGATGTCTGGCATGTATTTCTGAAGGGAGATTTTACTGAGATGCTTTATGGCTACAAATTTGATGGGAAATTTTCGCCTCAGGAAGGATGCTACTTTGACTCTTCTCGGATTTTGTTAGATCCATATGCTAAGGCTGTTATTAGTAGAGGGGAGTTTGGTGCCTTGGGACCCAATGACAACTGTTGGCCACAAATGGCTGGGATGGTACCCACTGCTAATGAAAAGATAAAAGATTTTGATTGGGAAGGAGATTTACCTGTGCAGCATGCACAGAAAGATCTCATAATATATGAAATGCATGTACGGGGGTTCACCAGGCATGAATCAAGCCAATCAGAATTTCCTGGGACCTATCTTGGTCTAGTTGAGAAGTTAGATTATTTGAAGGAACTTGGAATCAACTGTATAGAGTTAATGCCATGCCATGAATTCAACGAGTTGGAATACTTCAGCTATAACTCTGTCTTGGGTGACTATAAGATGAACTTTTGGGGATACTCAACAGTCAATTACTTTTCACCAATGATACGATATTCTTCTGCTGGCATAGGAAACTGTGGCCAGGATGCTATAAATGAATTCAAGCTTCTCATTAGAGAAGCACATAAACGTGGAATTGAGGTAATCATGGATGTAGTTTTCAATCACACGGCTGAGGGGAATGAAAACGGCCCAACTATTTCTTTTAGAGGTGTTGATAATAATGTGTATTACATGCTTGCACCAATGGGAGAGTTTTATAATTATTCGGGATGTGGGAATACATTTAACTGCAATCATCCAGCTGTTCGCACATTCATTGTGGACTGCTTGAGATATTGGGTGACAGAAATGCATGTGGATGGGTTTCGCTTTGACCTTGCTTCTATAATGACCAGAGGTAGCAGTCTGTGGGATGCAGTAAATGTGTACGGAAGTCCAATAGAAGGTGACATACTCACAACTGGCTCTCCTCTTGGCAATCCTCCATTGATTGACATGATAAGTAATGACCCAGTACTTCAAGGAGTGAAGCTCATTGCAGAAGCATGGGATGCTGGGGGCCTATACCAAGTAGGCACATTCCCTCACTGGGGTGTTTGGTCAGAATGGAACGGGAAGTATCGAGACGTTATACGGCAGTTCATCAAAGGCACAGATGGGTTTTCTGGAGCTTTTGCTGAATGCCTTTGTGGGAGCCCTAATCTTTACCAGGGAGGAGGAAGGAAGCCATGGAATAGTATCAACTTTATTTGTGCACACGATGGTTTCACTTTGGCTGATTTAGTGACTTATAATAGTAAACAAAATTTAGCAAATGGCGAAGAAAACAATGACGGTGAAAATCATAATAATAGCTGGAACTGTGGACAGGAGGGAGAGTTTGTTAGTATCTCAGTAAAGAAATTGAGGAAAAGACAGATGCGAAATTTCTTTGTCTGCCTCATGGTTTCCCAAGGCGTCCCGATGATATTAATGGGTGATGAATACGGTCACACGAAAGGGGGGAACAACAATACATATTGCCACGATAACTATCTCAATTATTTCCGGTGGGATAAGATGGAAGAGTCAACCTCTTTTGACTTCCATAGATTTTGCAGTCTTATGACCACATTTCGTCATGAATGTGAGTCACTTGGCTTAAACGATTTCCCAACGGATGAGAGATTGCAATGGCATGGCCATTCTCCTGGAAAGCCAGACTGGACTGAAGCAAGCCGTTTTGTTGCATTCACATTGGTTGATTCGGTAAAGCGAGAAGTCTACATTGCCTTCAATGCCAGTCATTTACCTGTCACGGTGACGTTGCCCGAGAGACCCGGATATAGATGGGACCCTTTGGTCGACACGGGTAAGAATTCACCATTCGACTTCCTCACAAATGACATTCCTAAAAGAGATGTGGCAATCCAACAGTATGCTCACTTCCTTGACGCGAATTATTATCCCATGCTTAGTTACTCTTCCATCATTCTTCTACTCTCTCCTGAAGAACCCGCATCATCTTGATCTTCTAATAATCGCACCTCATTTCCCTTAAAACTGCTTCCTTTCATGTGTGTATGTATATATAGATTGAAAATGCATGAGGGGTGGACAATCAACTTTTGACTCAAAATCGAGAGTACAAGCTTATGTCATCTGTGCTCGTGTTTGGAGTAAATAAATTGGGATGTGAGTGACATTTATTTCTTTATATCTTAAGTTCTTATACGACCTTTTTCTTTACATAGAGACATGGTTTCGTTTGGTAAATTAGAGTAATTTTGTTCTGTGTTTCGTGGTAGGGCTACGATTTTCCCCTTCCATAATGCTATTGTCATCTGTTGGGTGATATTTGGATA

>cucumber_newGene_26 cucumber_newGene_26.1

GAAGCCGCCACGTAGGAATGAAGAAAAAAACAACACCAATTGCCAATTGCCAATTGCCCTCCGTCTGTCTGCCTGTCTCTGTTGCTTTGTCTAGGAGGTCGTGCGGTGTAACAAAACAACCCCACTCTCACCCCCTTCTTCCCCGATTCTTCAATCCCCATTTTCCTTAAACCTCGAATCCCCTTCTACCCTTTTCATTTCCTTTCCCTCTGTCATATTTTAGGGTTCAAAATGTTCTCCTCTCTTTTCCAACCTCAACTCCTATCCAATCATGCCGTTTCCTCCACTTCTTTGGACTTTATTAAGCGCCGCTTCATACCCCCAAGGTTGAGACACGGAAGCCATAGTTGGTGTAAAGATTGATGCAGTTTGTTGAGTGATTGGAAGGCAGAGTTAGGGGATTT

>cucumber_newGene_27 cucumber_newGene_27.1

GATGGTAAGGTATCAAGCGAAGAAATTGCTCGTATTTGCATTGCTGCTTTGGAAAGTCCATATGCATGTGACAAAACGTTTGAGGTTAAAAGTGTGATTCCATTTGGTGAACCATTCACTGTGGACCCTGAGAATCCACCTCTAGAAAAAGACTACAATATTTATTTCAAAACCCTGAAGGATGGCATTACCGGGAAAGAACTCCTTGTGCAAACCCCTGCTCCAGTTTGATTGCGTTCCACGGAAAAATCTCCTTTCAGTTCGAAATATGTATTTGTTGAAACCCCCAAATTTGCATACTTATAAAAGAAGGGGCAAGAGAGGAATTTTTTGCGTGTCCCCCTTGACTGTTGGTATAAATAAGGAATGTTTTGGGATTGTTACCCTAGGTCTATTTTAGGTAAAAGCTGCTGAAGGCTTTTAGGGAGAGGATTCCAGCGTTCTCAAAGGAGCTGGGCACTGTTTGCTTGTTGTCTTTATTTTCCTTTTAACTCTTCTTTGCTGGATTTCTCATGTAATGTTGTGAACTGGATAATATTGCTCTTTCAGTATATGAAACAGAGTGTTTTCTCTGTTTTATTCTCTT

>cucumber_newGene_28 cucumber_newGene_28.1

TTTGTTTCTAATTCTCAGTTATCAAATATCAACAACAGACCTTGAACTCTCTTCTTTCATTTTTGCTTCAATCAATAATGGAAATCTCTTCCCTTGCCCTCCAACTTTCATCTCTACCGTCCTCCTCCTCCCTTCTTCTTAAGACTCCTTCGTCATCACCCACTACCACTACTAGAAGGAGTTTCTTCCTATCATCTCAACTTCCTAACCCATTTCTCCAAATTCTCGCCGGAAACCACTCTTTTCTCCACTTGAGCATCCCACAATCCTGCTCCCTCAGACTCCCAACAAAACCCGTTTCTGCAGAAGCTTGGGATTTTGGTAGGTTTTTGAGAACCTTGTACTTTTTCAATGGCCCCCCATCTCCATCCAAGTTTTTTGCGTCTCTGATTGCTCAACTGTCGGGTCCATCGCCGAGCAAACCTGTTGAAGAAATGGAAACTTCTGGCTTCATTCTAGTTGCTGGAGCAACGGGTGGGGTTGGGAGAAGGGTGGTTGATATCCTGAGGAAAAAAGGCTTGCCAGTTCGAGCTTTAGTATATCAGTCTTCCCATTTTCAGCTTATTGGTTTTCTATTTGTTGTGCTTTCATCAAATTCAAACCGCATGCTATTTGTACAACTTTTGAGAAGAGACAGAATAAGTATTGATAATGATGTCTTTTTTCTTTTTCTCAACGGTTAGTTTTATTTTATTTTATCTTTCAGGTCAGAAATGAAGAGAAAGCGAGGAAGATGTTAGGCCCAGACATTGGATTGATTGTTGGGGATGTTACGAAGGGGAGCACTTTGGCACCTGAGAACTTTAAGGGAGTGAGGAAGGTAATTAATGCTATCTCCGTCATTGTTGGACCAAAGGAAGGAGACACTCCAGAAAGAGCAAAGTATAGCCAAGGAATCAAGTTCTTTGAACCTGAGATCAAAGGCGATTCCCCAGAATTGGTGGAATTTATTGGAATGCAGAATTTGATCAATGCTGTGAAGAGTGGTGTTGGACTCCGAAATGGGAAGCTACTCTTTGGATTTGAAGGAAACAGTATCAAAGAAATTCCTTGGGGTGCTTTAGACGATGTGGTAATGGGTGGAGTAAGTGAAAGTTCATTTCAAATCGACATGAACGGTGGTGAAGCTGGTGGACCAACTGGAGTTTTTAAAGGTTTCTTCCTTTAGGCCTTTAGACAGTGTACCAGCCCAAGATTACGTCACTCTTAATAGAATGAACCATTGTGATCAAGAATCTAAGTTTGGATGCCTCTGCTTCAGGTGTTCTTTCAACTGCAAACAATGGTGGCTTTACTAGTATTCGGACAAGGAATTTTTCTGTGCCAGAAGATCTTTCTGCTTATGATGGATTGGAGTTGCGTGTCAAAGGTGATGGACGTCGGTATAAACTGATTGTCCGCACCAGCACAGACTGGGATACTGTTGGTTATACAGCCGGCTTTGACACTGCAAAAGGGGAATGGCAGTCTGTTCGTGTGCCATTTACCTCTCTGAGGCCAATCTTTCGAGCTCGTACTGTTACAGATGCCCCACCTTTTGACCCTACGAATGTTGTTTCACTGCAGCAAGTTTGAGTATGATGGGAAACTCAACCCAACTTTTGTTGAAGGTCCATTTCAACTTCCACTGTCAAGTATAAGAGCTTATATAAAAGATCCTATAACTCCCAGGTTTGTCCATGTGAGTTCAGCTGGAGTTACAAGACCTGAGAGGCCTGGGCTTGATCTCAGCAAACAACCTCCTGCTGTCCGATTGAATAAAGAATTGGATTTTATTCTTACATCCAAATTGAAGGGAGAGGACTTAATTCGGGAAAGTGGAATTCCTTATGCAATAATAAGACCTTGCGCGTTAACGGAGGAGCCTGCTGGTGCAGATCTCATTTTTGATCAAGGAGATAATATAACGGGTAAGGTATCAAGGGAAGAAATTGCTCGTATTTGCATTGCTGCTTTGGAAAGTCCATATGCATGTGACAAAACGTTTGAGGTTAAAAGTGTGATTCCATTTAGTGAACCATTCACTGTGGACCCTGAGAATCCACCTCCAGAAAAAGACTACAATATTTATTTCGAAAACCTGAAGGACGGCATTACCGGGAAAGAACTCCTTGAGCAAACCCCTGCTCCAGTTTGATCGTTGTCCACAGAAAAATCTCCTTTCAGTTTGAAATATGTATTATAGTTCGAGGAATCGAGCATTCATTTTTGGTACACCAAATATGGTAGGGGCTTTAGAACAAAAGGCTAAATATAAAATCTTGTCTGTGACTTGTCTTTAACATCTTTACAGGTTACTTTCTTTGTGTATCCACAGAAACTGTCCACAGTGTTACAAGGTGGAGGTGCATGGGATTTTATATGCTGCACCTCATCTGTAAATCAGGTTGATCATGATTTTGTTAATTCTTGTGTCCCTTTGGTCTTCTACGAGTAAATCTTGGATGGATTTTGTTTTGATTTTTCAATTGTATATGTTGTTCACACGTATATTTAATTTAATAGTTCTTTAGTTATTGGAAGCAAACACTCTTAAGCGCATATAGAAGGATGTTAGTATTAGAGGTCCCTTGTGTTCATGTTTAGAGCTTCAAGAATTGATTGTGCAAATTCTACTTTAATTCCTATTTTGTGCGAATAGAGGACATCTACATGATGACATATATTATGGTGGAAATTTTGAGATTTCGATGAATTCAAAATT

>cucumber_newGene_3 cucumber_newGene_3.1

CACAAGTCCCCAGACTCGCACCACTTCTCAAGAGTCATAGCCCCTTTTCACTGCCAGACCTTTCCCTCTCTTTTTCCCGCCATTACAATGTCTTCAACCGCCAGTTTGAGCTCTCCCATCCATTACTGACATCATACTATAGCAATCGGAGCACCCAACGCCGGCATGATCACCTCCAGAGATGTCCAAGATATCATTTCAAAGCTATCTTCTGATAAAGCTAAAACTCGCGAGGAAGGGATAAAGTTGTTGAATACATGGCTAGAGGGAGAAAAAGCAATAGATTTCTGTAAATTTATTGGACAGAACACTGCTAAGCTCAAGCCAGAAGAAATTCCATCTCCTGAAACGTGGCCTTTCATCACAAAACTGTTGATTCAATGTGTGTCTATGGAGATATCTTCAAGCAAGCGGCGATTACCAAAGTTAATGTTTGCGAAGACACTTAGGGGTGTCGTTCAAAAAGCAGAAGCTAATAAGTTTTCTGGCAATGCATTACCCCTTATATCTGTGGTCAAGGTGCTCTTTAGTCATGTATGGGACGTCTTGAGTACTACCCCATGCTTTCAATCCGAGTATGGCATCATTATTAGGCATCTAGTAGCCGTAAGAGATTATCGTTTTCATTTGAGGAAGCGCATATATTGCAATCTCATGCTTCTGTACTTGGAGAAGGTGGAAGGAAGTTTAGTCGGGAAGAATGACAATCTTTATACTCCTAAAGAGGAGCTTTTCCGTTGCATCTTAACACTCCATTCACTTTTGGAAAATCCTCCTGGCGACTTCCCGGATAGTATTAGGCAGGAAATTGTGAATGGGATTGCTAAGATTTTCTCTCTTGTGAGGGATGAAGGAAAGGTATCACGCAAACTCATTGAGTGTGTAAATACATACTTGTTAAAGGATGGTCCTAATCTAGGAAGTAGCTTATTGGAGATCCATAATTCTGTGCAAACATTTGTATTTCGATGTTGGATGGTGACTCATGATCGAGCTCTTAAGGATGCTCTTGTCCTCTATGGACGGTTACAAATAAGTTTAACGAGAGGAGCTGATGATGAGAGCATTTTGATAGAACAGCTCCTCGATGTACTTTATAAGGAACTAGATCAAAGCAGTATATTTAGTGTTGGGGTGCCTTGGTCTGATGCAAATAAAGATGATAAATTTGGAACTTTGAGTAGCTCACATTGCGGGTTGGTAGAACTTGCTGCAGCTGTGCTCTATCGGGCATGTGTTACTTCTACAAAAGCAATATCAACTGAAAAGCGGGTTAAAAGAGACCCTGCTAGTGTTCATCTTAAAGAGGCACTTGGTGAAGGGAAATGGTTATGGAATGCGGCTTTCTGTTATCTTATCCAAAATTACCATTCCCGCATCAGTAAGGATCTTTTCACCTACTGGTTCGAGGCAATTTGCTTAGGTTTTGAGAGAATATTGAAGGATGCAAATGTGGGCCATTCTTATGATGGTTTATTGTGGACATTACGGAGTTTGCAAGAACTTTCTGTAGTGTTATTGGATTCCACTGAACGGTCAGAAAGTTCGTCTGAGGATAAGTTGTATCAGGGGTGGCAATCAATATGGAGCTGTTTGATGCATGGACTGCCCATGTTTACTCATGTGTTTGTGGTGGCGGAAGCTGCTTTGTTGCTTCTAGGAAAAATAATTGAACGAGATTCCATGAAGATGTGTGTGATTCCTCAAGATGTTTGGGAACATTTATTTTTCAAACGAATGCCGTCAATATCTGCTTTGTACTTAATATCCTGTTACTTCTCGAGAAAAGGGTCTCAAGGTGACCTTCGAGACATTTTCCATCTTAGGAAAAATCTTCTGAGAGCAGTTTTAGGTCTTCTTAACTTGAAGGATGTTGCTAGTTTCAACGAGCAGTTGATATTCTTGTTGCCTGCTGCTGTTTATGCTCTTTGTGCGGGTTGTGCTCCTTCAAGGGATGTTACAGGGGTTTCAGATGAATGTTTGAAGAAAGATGTGAAAGAAACTTATCGAGATTGGTCGCAAGAAATTTTCGAGTGTTCTGTGGAAATTGTCGCCAAAATACATCTTGGTTCTAGTGTTAAGATATGCCCAACCCGAGGCCATAAAGCTATTCATCTGCCTCGTCATATAAGAGATCCGTTGTTGGAAGAAATGGTGGCTACTGTTCTTGGAGCATTGATTGATATGGAGAGGGAAAAGCTGCTTCCATCGAGTATCTTTATTTTATGTGCTTTATTGGCAAACTTCATTGATGGTTCAACTTTGATAAGGCAATGGGAGAAAGCATCTTTGTTCATTTCCAGATTGGGTGGTTACATTTTAGAAATGATGAATCATGCTGTTAATGTAATCCAAGGGTACTGCGGGGATATTAAACATCTCAATTTCTTTGGCTCTGACTCATTCCTTGAGACAACGAGCTCTGTTGTCACATCCTTTAGAAGTTTTGCTTCCAGTTTTATTTTCAACATGGAAACATGCCATAAAGCTCCGGATGTTGATCTGTCTGGTGCATTCAATCTTTCGATGGAAAATCTTTTGAAAGCACTTTCTCACCTCTATCAAGAATATTCTATATCCAATAAGAATCTCCATTCTGAGGCAAACTTGAGAGATTTTGATGCACCTGTTTCTCCTTTGGCGAATTCCCCCTCAGCTGATGCTGAAGTGAGTAGAATTTTGGATATGGAATTGGATGTGAATAATGACTCCAATGACATGGATATTAAAAGAAGTATGATGCCTGGAATGCTTTCAGCAACAGTTTGGAAGTTGAAGATGATATCTCTTATTTCAAGTTTTTCCTCAGTTTTGCTTGAAGCTACATGGGAGGTTCTGTTTGTACTCTTTGAGAATGAATGTGATTCAAAGGTATGCGAACAGATTATGTATCATCTCAGTCAAAACATCTTGTGGTCATCTTCGGAAAAAGTTCTGAACATGATGATTGTAATGGATAACATGATTAGTACAAAGGTAAATCTTAAGCTGGATTTGTATAGCACACTAGATGCTGCTGGTGGTTTACTTAGGAACCTTTCATCCTTGCATGGGGTTAGTAAGATTGGTTATCGATTTCCTAAGGATGCACAATTTGAGAAGAACTTGCTTCAGATTGGAAAATTAGTGAACAGGATTGCTGAAACTCACCTTCTTGACTGGTCGGGGCGTGTTAAGCTTATTGATTGCATTTGTAGCTTTATTTTAATCAGTCCAGAAATTGGTCAGACAATGATTGAAAGGTTATTTGTAATGCTGCGGGATCCTGAATATCGAGTTAGGTATTCCCTGGCGAAACAAATGGGCGTTCTCTTCCAGACATGGGATGGCCATGAGGAACTTTTCCAGGATATATGTTCCAGTTTTGGTGTTCCATTAGTGTTGTGCTCAAAACAAAAAGTTGTAACAGCAAAGGAAGTCCTAGATGCTGGTTTGGAGCTTGGTCCTACAATGGAAACTATTATTGTTACACTTGGACATCTTGCTTTGCATAGTGATGCAATGGAGTTGGAGGCTGTTTTCATGATGTGTGCAATCTCGGGCATTGATCCTAGTCAAAGGGAAATGGTCTCTGCTATGCTTGATAACCTGTCCAGAGAGCTCAATTATTCAGGAAGACAGAAGTACTTGGAGGAACTCATGGGATCACTTCTCTTTTGTTGGGTTACTTGTGGTGTGAGCTTAGCTGCACTTATTGAGATAAGGCAACTTTTTGTCTTGGATTCTGAACCCAGTTACTTTATTCAGTATTGTTGCCATTGGCTTCTTCCTGCTGTTATTTTACATGGCGATAATTCCAACCTTGGCTGGATTGCTAGTGTTGCTGGTGAACCAGTGGAAGCTCTAATTAGAAGCTATTTTGTGCCAATATTTTCCTATTGTATGGCACTGCATTGCAGCAAGAGATCTGGATATGAAAAGGGAGCTATAGTCCTTCAAAGTTCGATGTTGCATTTTGCTAGAATAACCGAGAGCGAAAGAGATATACTTATAAAGAAACATATGGTGTCCATTATCAGTCAGATCTTAGCTTTAGCATCATGTACATCAGAACCTATGGATCCATTCTTTCCAAAGGATACTGTTGTGCTTGCTGTTCAAACAGTTGTTGATGGTTTCTTGGAAATGGAGAGCAGAGAAACATTAAGTGGTGTTATTGACAGAATCAATGTTTTCCGTCCAGATAGATTTATAGTAGAGATGCACTACAAAATCACAGAAGCAATTCATCACAGGCATAAATCTCATCGATTGGCATCAATTGAAGCGCTGATCAACATTCTTGGTCACAGAGCTGTTGTTTCTAGCACTTCAAACTACCTATTCAATTTGATTGGGCAATTCATTGGCAATAAGTCGTTACAAGACCAAAGTTGTCACATCTTTTCAATCCTGTTGAAATCTTTCAAATCTAGTCCTGGCAAAGAAATTAGTAGAGTCCTCGGAGAACAACTTCAGTTCTTGATCTCAAAGCTGGTGGCATGTTACATCCCCTCTGAACCTGATGGAGATTCTCTTGACAATAGAACATCTCACCTCATATCTTTGATTCGCCAACTCACAGTGGATTCTGATTCATCGCTCCATGATTACATAAAAGAACTGGAGCCCTTTCCCGAGATGGATATATTTGATGATATTCGAAAATTCCATCAGGAACTATGCAGAGGGTATTCTCCAAGGGATCATTTATTGAGATTAGTCAACCGTTCTGGCAACCTACCGCCAAGATTACTACTATGGAGTTTAAAAGCATTGCACAAGAAGTTGATTGGGGGTCGAGTTTTTCACTCTGAGAAGATCCAGAGTGTTGATTGGCACAATGATCATGAAGTGGAACTTGCAGTCTGGAAACTTATGCGAATGTGTAGTTCAGATGATACGAGTTGTATAAGGGAGCTGGTGTCAGATTTTGTATCTAGGGTTGGTATAGGTGACCCACACTGTGTTGTTTTCCATCTACCTGGGGACTCTAAGACCATTCACATTTTTCGCCCAGTTGTTAATGGCAATGCTTCAGAAATAGACTTGAAAATTGAAACTGGGATATGTAAAGATCTTCTGGTTGAACTTCTAAAACGTTTGAAAAGGTACCTGATGGATGATTCTGTTAAAATAGTAGATATGACGTCCCAAGTTCTCCAGGCGATCCTCTCCACTGAAAAAGGTCAAAGTACTCTACTCAAGTTTGATTCTTATGAGAGATCACTTTTAGAGGTTCACTCGAGAGGGCTTAATATTAAGCTGGTGGATGGGTACTTGTTGGACCTTGAAAGCAGATTTAAAGCGGAGGCTATTTCAGTGGAAAGTTCCACCGTGTGGGAGACCAATGGTAAAACATTTGAGAGGTGGATTTGTCCGTTAGTGTACTCACTAATTGGACACTCAAATGATGTGATTCTAAGATTATGTCTAGATATTGTGTTGCTAAAAGCTGAGATTGCCGAGCTGCTTTTACCCACTGTAGTTGTCAATCTAGCTGGAACAAAGGATTTAGATATTGATCTACAAAAGCTAATTTCAGTTCAGGTGCAAGAACACATATTTGTAGAATCAAACAAGTTGATCAAGTCAATTCAAGTACTTTTGAATACCCTCAATGAACTTCGGCTATATCATGTCATGGAGCGATCTTTTGTATCTTTGAGGAAAGATAATTCTAAGCCTTCTAAGGGATCAAGTAAGAGTTCTAGATCCCGTTCCACCTCTGTTAATTGTAGAGATCCAGTGGCTGCATCTAATTCTTCTGTGATGCCACCAGTCTCCTGGGACAAAGTTTATTGGCTTTCTATTGACTACCTTATTGTTGCTAAAGCTGCAATTTATTCTGGTTCATACTTTACATCTGTCATGTACGTTGAGCATTGGTGTGAAGAGCATTTTGGATGCCTTTCCCTGGGGACCCCAGATTTTTCTTACGTGGAAACAATGCCACGTCATATTGAAATACTTGTCTCAGCAGTCACTCAAATTAATGAACCCGACAGCTTGTATGGAATTATACGTTCTCACAAGTTGTCGTCACAAATCATCACCTTTGAGCACGAGGGAAATTGGAGTAAGGCCCTTGAATACTATGACTTGCGAGTACGATCAGATTCCCTGGTTCAAGAGAATGGTGTTGTTAAAAACATTTATATGGATAAGCAACCACAACGTCATCAGTCAATTTCTGCCTTGGAAGATGCCTCAGGACACTGGAAACCTTACAAAGGGGTTATTAGATCTCTGCAGAAAATTGGTTGTGCACACGTTCTGGATTTATATTGCCAGGGTTTAACATTCCGGGATGACCATGTTCAGCATGATTTAGAGTTCATGGAACTGCAGTATGAAGCTGCCTGGCGTGCAGGAAACTGGGATTTTTCTTTGCTATATGCAGGACCTGATTCTGGTTCATCAAGTTATCAGACCAAGAATATTCATTTCAACGAAAACCTGCATAGTTGTTTGCGGGCATTGCAAGAAGGAGACTTCGATGAATTTTATAAGAAGTTCAAAGATTCAAAGCGGGAGTTAGTGTGGTCCATAACTCATGCAAGTGAAGAAAGCACCGAATATATATATTCAACTATAATTAAGCTTCAGATTTTTTATCATCTTGGCCTGGCTTGGGGATTACGTTGGGCGGATTCTGAATATTCAACTTTTTTCAATGGGAATCCAAAAGTGTTATCAGATCATGTGATTCCCACAATGGACCAGTTATCTTTGCTGAATTCTGATTGGAGCTGCATTCTGAAAAGTACTCAGCTACATATGGATTTATTAGAACCCTTTATAGCATTTCGTCGAGTTCTACTTCAGGTTTTGAGAAGTAAGGAATGTATGGTGGAACATCTTTTGCAATCTGCTTCCACCCTTCGCAAGGTGATACAATACCATCTAGAAGCTGCATTCAATTTTCTTTTCTCCCTCTTTTTTCCTTTTTTGATTTATAGTTTTATAATTTTCAGGGATCCAGATATTCTCAAGCAGCTGCCGCTTTGCATGAGTTTAAGTCTCTCTCTCTTCAGGAAGCAGAAGAGAACACGCCATTGTATTGGCTTGGAAGGCTCGAAGAAGCAAAGCTGTTGCGTGCCCAAGGTCGGCACTCAATGGCAATTAGCCTTGCAGAACATGTTTCTCAATACTTCCAATCCAGTGAGGAAACTTCAGATGTATTACGCTTGGTTGGGAAGTGGCTAGCTGAAACTAGATCGAGCAATTCAAGAACTATTTTGGAGAAGTACCTAAAACCAGCCGTATCTCTTGCTGAGGGTCAGGAGTTCCTTAATAAGAAATCACTTGAGAGACAGAGCCAAACTAATTTTCATCTTGCACATTATGCTGATGCGTTGTTTAGGAGTTATGAGGAAAGACTCAGCTCTAATGAATGGCAAGCAGCAATGCATTTGAGGAAGCATAAGACAATGGAGTTGGAAGCACTCATTAGACGACTAAAAAGTTCAACAAAGGGCGAGAAGACTGACTTTACAGTGAAAATACAAGAATTACAAAAGCAACTTTCCATGGACAGGGAAGAGGCTGACAAATTGCAGGATGACCGAGACAATTTTCTCAACCTAGCATTAGAAGGATATAAACGATGCTTGGAGGTTGGTGACAAATATGACGTGAGAGTGGTGTTTCGACTAGTTTCATTGTGGTTCAGCCTTTCTTCTAGGCCAAATGTCATAAACAACATGCTGAGCACCATTGTTCAGTCGTACAAGTTTATACCTCTGGTTTACCAAATTGCTTCAAGAATGGGCTGCGCAAAGGATGGTCAAGGGCCAAACAATTTTCAGGTAGCCTTGGTTTCTCTCGTGAAAAAAATGGCAATAGACCATCCATACCACACTATATTTCAGCTTCTTGCCCTGGCAAACGGTGATCGTGTCAAAGATAAACAACGCAGCAGAAACTCATTTATTGTGGATATGGACAAAAAATTTGCTGCAGAGTATCTATTAGAGGAATTGTCATCCAACCATGGTGCTTTGATTAGACAAGTCAAGCAAATGGTAGAAATCTATATCAAGCTTGCAGAACTGGAGACAAGGAGAGAGGATACTAATAAAAGGATGATGCTTCCAAGAGAACTGCGTAGTCTTCAGCCATTGGAACTTGTACCTGTAGTGACGGCTACCTTTCCAGTTGACCGAAGTTGTCAATATCAGGAAGGCTCCTTCCCGTATTTCAAAGGCTTAGGTGATACAGTAAGGATTATGAATGGCATAAACGCTCCAAAAGTTATTGAATGTGAAGGTTCTGACGGCCACAGATACCGACAACTTGCAAAATCAGGAAATGATGATTTGCGACAAGATGCTGTAAGATTTCAGTGATGAAATGATGGTACCATTGGAATCCCTAAGTAAGACCTTTTATCATTTGCAGGTTATGGAACAATTTTTTGGATTAGTCAACACCTTTCTCCAAAACTATCAGGATGCAAAAAGGCGGAGATTAGGAATACGGACGTACAAGGTCGTTCCCTTTACTCCAAGTGCTGGTGTTCTTGAATGGGTTGATGGAACTATTCCTCTTGGAGAATATCTTATAGGAAGGTTAGTAAATTTCAGTGGTTTTTGTTCCTCCGTGGGATTGGTTCTGTTAGTATTAAAGCCTCCAAATAAATCCCAGCACCAGAAATGGAGGAGCGCATGGTCGCTACGGAATAGGAGATTGGTCATTTCTTGAATGCAGAGATTACATCGCAAAAGAGAAGGACAAGCGTAAAGCATTCCAAGAAGTTTCTGAGAATTTCAGGCCCGTCATGCATTACTTTTTCTTGGAGAGATTCTTACAACCAGCTGATTGGTTTGAGAAGAGGCTTGCCTACACTCGTAGTGTGGCTGCTAGTTCAATGGTTGGTTATATTGTTGGCTTAGGAGATCGACATTCCATGAATATTCTAATCGATCAAGCTACAGCCGAGGTCGTTCACATTGATCTGGGAGTTGCCTTTGAACAAGGCTTGATGCTTAAGACACCTGAGCGAGTTCCGTTTAGACTTACGAGGGATGTGATTGACGGTATGGGTGTTGCTGGGGTGGAAGGGGTTTTCAGAAGATGTTGCGAGGAAACTCTCTCTGTTATGCGGACAAATAAAGAGGCGCTGCTAACCATTGTTGAAGTTTTCATCCATGATCCCCTTTACAAATGGGCTTTATCCCCTCTTAAGGCATTGCAGCGTCAAAAGGAAACCGATGATGACTTGGAAACTAGTCTCGAAGGCTCAGAAGATGAATATGAAGGGAATAAGGATGCTGCACGTGCACTGTTGCGAGTCAAACAAAAACTAGATGGATATGAAGATGGCGAAATGAGAAGTGTCCATGGACAGGTACAACAGCTGATACAGGATGCTATCGACCCAGATCGTTTGTGTCACATGTTTCCTGGCTGGGGAGCATGGTTGTGATTATCTTATTTACATATCGAAATCCCCAATTGAGCCACCCTATATCTTGTACAGGCGGATCTTTCTTCTTGCTCTGTTTGTATATTTCACATGTACATTCTCTCCCACCCCAAAATGAAATATGATGTCAATAACCTTCTTTAGTGGTCGTCAACAATCTTTTCTCAAGAAATTTATTTCAAATACTGAGATCTTTTCTGGATGTTGATTTTTTATATTTAGGAACTTTTAATCAAGAAGTAGGACAGTCTAAACCAACTCAACTTGACTAATGGTTGATGGAGGTTTAATACCAAGGAAATGTTCATAAACTCTTACCATCATCATATGTTAAAACTTCCTAATTCTCATTGTTTTTACATCATTTTGCCTACTTTTTAGTTTCTGCATTGAATTTTTTTTATTTATCGCTAGATCAATATGAATTTAACTTTTTTTTGTAGACTAATGCAAACATAGCTCAAAAATAAAAGAGAATATTCACTTGTTTTTCTCCAAGATTGTCTTTTACAGCTATTCTTAAACAAACTAAGATCGTTT

>cucumber_newGene_33 cucumber_newGene_33.1

AAAGAAAACCCTAAATCATTCAAAACAAAAAGAAATAAAATCATTAGAAACAAAAGGAGAGATTTGGACTAAAAGAACATATTTTTAACATAGAAATTAGATCCAATCAATCTAATTCTTCTAATAGAACCGATTTGAAGTTTGAACAATTTAAAAGAAAAATCAACAATGGAAATCTGGTAATCAGAAATTTCAGGAAGAGGAGATCGATCTGATTGCACTTAAAAGTTCATCAACTCATCAATGTAATTACAATTTGAATCTAATTGAATCCAAGAAAACAGAGTTCCCCACCCCTTCTTCTCAATGGGGTTCCACAAAAAATTTACATCAATTTCTCAAAAGGATCTCAAGATCCAATTCGAATGAAGAAGGTTAAGAAATCACATCTCTGAATCGAACGAAAATGGGTTGAACCCGGATCGATTCTTTGTCTTCTTTACGAATCTCCATGTTTATGCCAACACACAAGCATCGCCACGCAGCGTCTCATGATGTAGAATCGAGCCTTCTGTTCCTTTGCCAAGCTGCTGTACTTTCTTCCAATGGAATTAGAAGAGCTTTTCTGTGAATAACTTCGAGGGAGATCGTGGGGGTTCTTGGAATTAGAAGATGGGATGCTTTTCTGTGAAGAACATCGCAGCAATGGCGATTTGGAGGTTGACCCGCTTCTCAGGAATGACGATTTCGCATTTGTGGATGAATGATGAGAGCAAACAGAGGGCATCCATTCAATTTGGGGAAGTGGAAGGGAAAATAATGGAAGCAGTGAGAAAGGAGACAATTTGCTAGCTGTTTATGACGTGTAAGGTATGTGTCTCATACTCTCACCTCAACTTCTACAAATAGATCGAATGCTTTTTTTCAAAATAAAAACAACACAAATTCTTTTTTCCTTTAAAATGATGAGTATCTGCAGAATAAGGACTTTAATTTGCTTTTATTTTTCCCCCTCCAGAGGGGGGAAACATGCAATTATTTGCCTATTTTTGTTCTTATTAGAAAAGAAGGGATCTATGACTCAACCTTTTCTTTCCTTCTGTCTTTTTGTTACACTTGCACCATTTTTTTCCCTTCTTCAAGAACTCTTCATAAAAAAAATTCACCCTACAAAGTTCTCTCTGTTTTAGTTTTTTCAAGAACTCATTGTACTTCACTGTAGTCCATATATCCCTAAAATAGCAAGAAGTTCAATAGAATAGACAGAAGAAGAAGAAATGAAACTTCATTCTTAAAGATATCTAGATAAATAGTTAGAAGGAACAATTATCTGGTGAGACCGCACACTGTTGTTGCTGTTAGTTTTTTACTCTGCTGGATGTAGAAAGAAGTATCTAAACTTTATAGACCATAGTTTAGGGACCATAAATCTACACTTTGAAGACATTTGGAGTGGACTATTATAACTTCCACTTTCTTACATTAAAATTATTTAAAAACTCTCCTCCACTATAATAATGTTTACTATTTTGTGTTATCCCTTCGTCCATTTGTAACCTAGATTAAACTATCGTTACATTATGACTATGAGTTGAGTGGGTTTGAAGAAAATGAGAGGATTAGGGGAGATATTATTAATTAGAGTTTGATTGAATGGTCAAGGAAAAGTAGTTGATGAGTCAAAGTAATGATCACATGGTATGTTGTGGTTTTAGAGTTAAAACCCAAAATGCATCTCTCTGTTTTTCTTCTCTTCCTTTCTATCTGGGGGTAGATTATTGTTAATCATTATTAATATGCTGCTTGAAATTTATTACATTATTAATTAATCATGTGGGACTGAGAAAGTGGGTATTTTGGGTGTCCCACATGCAATCATGTGAGGTTAATGTTTTGGAAGGGATAGAAAAAAAAATGAAGG

>cucumber_newGene_35 cucumber_newGene_35.1

TAGTACTAATACTTTGAGATTAACAGTCACATTTGTAATTGTTCTTGGAAAGGAATAACATAATTTATTCTAAGAAATGGACATGTAGTGTGAAACTAAATTAAAAGGGAAAAAAACCCTAAAAATAGTTCACCAAAAATATGTAAACTGAAATTTTGTTCTGAATCTCTGATAAAGCAAAAAAGTCCTTTTTATCATAAATAGCTTTCAAACAACACCACGTTACTTTTTTCAGTTGAACCACTGAGTACTTAACTTTACTCTTCAGCTTATCATTCCTCACTACGCTACACTTCTCAGTTAGATCACTTCTTGAAAGTGTAATTAAAGTTGGTTAGATATGCTTGTTTTGATCTTTATTGTGTAGTCTCCACCATAGAACGATCATGCAGGTAAGGAAGTCCACTAATAAGCAATCTAAGATTTCCCTTCAGCATAGTGGGTTTATTACCTGTTTTCTTCTTGCGGAAATTACACTTCCACATGCAAGTTTTGCTCAATGAACTTTTTCTGCCACAATGACCATAATTCAATTTATGGCTTGATATTGTTACCAAAATTGTTAAGAAATTTATTGTTTGTTTTCATGCTTGCTTTCTTCTCATGTAATGGCTACGTGGATATTAATATTTGAACTGTTATTGTTATTTTGTCCTATGCCTATTCATATAATTTTCTCTTCTTTTAGCGCACGGATCCAGTAGAACCGCGGGAGTTGAACGTTTTAGGTCAAATCAGTCCCCTCTTTGGATGTTAAGAAAGTTTAAGATTAAGTTGTATGAATTTCGGTCAGATAATTGTGATAGTATATGTAAAACTAAACAATTTTGATCTAAGAAGATGCACTAAATTATAATCTGTTTGACCTTACACTGCAAAAGTTTTCCTTTTATAATCTGCTATGCTTTTAGACCAACTGCATCAAGTAGTAAGCATGTTATTTTTACGCCATGTTTTTAACCTTGGGAGTTCTATGACAACCTGAGTAAAAGTTACAAACATCATCCAGGTGTGATCTAAGATGCATGACACTGTAGTTACAGGAGGGAAGGGATGGTCTAATCTCATCACATTGAAAAACTTGCCCATCACCTCTGGTGAATTATAGAAGTATCAAAGGGCACACCAAATGATCCTTATCAGTGGAATGGATATGTGTGTTGTTGATTCTCTCCTTCTTAGGATGACGTTGAAAGATGTCACTCTGGAAGGAATTTGCTTTCGATATGGTCTCTGACATAGAGACAGCAGAGGACAGGACGTCCAGATTGATGATACGCAACATGTACTTAAACATTTCCTATCAATTATATTATTACGTTGTCTATCAGTAAGTCAACAATTGCCAATTATTGAATACCTTTGAGATATTGAAACTGTATCTCCAAGAAGATTGAGATTTTCATTTTCATTCTAGTTTGTGCAACTCATATGCACTTATGTCACAAGCTCTAGTGGATGTACTCTAGTGAAGCCTCTCGGTCTCTTGACTAATAATGACTGCTGTGAAACTTGATGATTATACAAGTTCACGGTCTCTCTCCACTGAAAATCTTTTCTTCATTCTTTCCTCTTCAGAATTTGGATTCAACTTCTCAACAAAGTCACATCATAATAGTAAATATTACTATTCATAGATATAAGTGAG

>cucumber_newGene_36 cucumber_newGene_36.1

GAGAAAGGCATTTTTGCTATAAAAGAAAAGCAATTGTTATCAATTGAAGTTGGGAATGCCCCCAAGTGCCATAAACACCCCCAAAAGGAGGAGGGAACCACTTGAGAAGTTGCTCAAAAGATTGGGTGAAAACGGTGCCATAAGAATAGAGAGTTCTTGCAAGTGGCTAGAATCAAATTGCTCGAGAGGGAGTAAACGAGGAATAGAAGCACAAAGGCCAAAGGGAATCTTAACCCCGAAGCAGTATTAGCACACTGGCGGCTTGAGAAAAGTAGGATGAAAAAGTGTCAATAAGAAACCAGCCAAAAGTAACAATACTGTGTTCCCAAAATAAAGAAGATATCACTGAAGGAGTGTTAAAAAGTGGATTTGCAAACCCTATCCAGCAAAGGTGCATCATATCAAGCAGCTCAAAGGGACAAATTTGTCCCAGCAAAGGATTGATAAAAGTTCCATTAAAATGGATCTTCTTGGCCCAAGCCTGGAGTGGAGTGCAGCACAAAGAGGGTCCATAGGCAGTGGTCAGTAAACAAGTCCAAGTTAACTAAAATTCATTAGAAACATAAACTGTGGACTAAATAGGGGGAAATTGAAGGCTCAAGATTTAATATCAAATTCGATTCGATTCTCCCGCTCCAAATTTAAAAAAAAATAACTAATAATGTAAAGAGTGTATCAGAAACCTAAGTCATGCATTGACACAATCAATGCATAGTACTTGTATGATGCTATGCTATGCTCAATTATAAAAATAGTACAATTTAATGGCGTACTATTTTGGAAATTATTTGGCATCCACCTTCCACTAAATTGACTTAAAATCTACATGAATGGTTAGTAGACCATGTCTTCATTTGTAGGCCCATAAGTTTTATAGCAGATGTTCATTGAGAATGAAG

>cucumber_newGene_38 cucumber_newGene_38.2

CTTGGACTTGTTTTGGAATGAAGTAATACTTTGGTGGTAAGAACGCTTTGCTATCGGAGAGCGTGAAAGGAGACTTCTATACCGTACTGAGTTATCGCGGAGGAACTGCTACACACTTCTCCTCATTTTGGCGGGAGAATCAGATGTTATCATTTTCCTCCCGCTCGGTCCACCGTAGTGTGTTGTTTGTATAATTTTCCGTCCATCTTCTTCTTCCTCTTCTTCTTCTTCTTCTTTCCCGCTGTTCTGGCCAAAATTTGTGTGCAAGATCTATCAAATTGGAAACTGCTAGCGGTTATCGCTTAATAGGACAGTGTCGAGTGGGGAAGTCGGCGTAAGCAACCTTGGCCTCTATTCATCAGCATACTGCTTTCAGTTCTTCCAGTTTCTAGGAATCCAATTTTCCTCCAGTTTCGAAGACCATGCGTTGGTAGTGCGTCTTGGGTTGCTGTTCGAATTATCTTTCAACAACAATGGAGGGTGCTGGTGCTGCTGCTGCTCCTATTTCTTCTGGACCGACCACTCGTCGCTCGAAAAGGACTCGGGCTCAGACTGTACCCGCCGAAGTTCAACCTACCTATGAAGATGGTGGTGGTGCCGACAACAATGACAGGACCAGCGATGCTAGCGGCCAGGCCGACCGAGATAGCTCACCTGAAAACTTTGAGGAGTCTCGGCCTCCTAGAACTAAGCGGCATCGATTGGAAGGCACTTCGAATGCTGCACATGAAGTTTCCGAGCAGAGTCTGATTGATGTTATAAAAGGAAATGGGAAATTTATTCCTCAAGTTGTTAAGCGTTGGGTGGAGAGGTATGAAAAAGATCCAAAAACTTCAATGGTCGAGCTCCTGGCAACGCTGTTTGAGGCATGTGGAGCTAAATACCATATCAAGGGTGACTTCCTGGAGGAGACTGATGTTGATGATGTTGTTGTTGCACTTGTTAATCTCGCTAAAAGGGGTGAAGTTGAAGACTATCAAAGCTCTAAAAGGAAGGAGTTCAAAAGCTTCAAAGATAATCTCGAGTCATTCTGGGACCACTTGGTCCATGAGTGCCAACATGGGCCTTTATTTGATCAGGTGTTATTTGATAAATGCGTGGACTACATAATTGCATTATCATGTACTCCTCCAAGGGTTTATCGTCAAGTAGCTTCATTGATGGGTCTACAACTCGTTACATCTTTCATTGGTGTTGCCAAAATGCTTGGTGTTCAACGTGAAACTACTCGTAGACAGTTAGATGCTGAAAAGAAGAAACGAGTTGAGGGACCTCTTGTGGAGTCTTTAAATAAAAGGTTTTCTATGACTCATGAAAATATCACAGTGTTGGAGGAAATGATGCGCAAGATTTTTACAGGGTTATTTGTGCATCGCTATCGTGACATTGATCCAAACATTAGAATGTCGTGCATACAATCATTAGGAATATGGATTTTGTCCTACCCATCATTATTTTTGCAGGATTTATACTTAAAGTATCTTGGGTGGACATTGAATGATAAAAATGCTGGAGTCAGAAAAGTTTCTGTTCTTGCATTGCAGAATCTTTATGAGGTGGACGATAATGTGCCAACACTTAGTCTTTTCACTGAGAGGTTTTCTAATCGGATGATTGAATTGGCAGATGACATTGATGTTTCTGTGGCTGTGTGTGCCATAGGACTTGTTAAACAACTGCTAAGACATCAACTTTTAGCAGACGATGACTTAGGTCCCCTTTATGATTTACTGATTGATGATCCACCAGAGATCAGACATGCCATAGGAGCATTAGTGTATGATCACTTGATTGCTCAGAAGTTCACTAGCTCCCAATCTTCTCGGAGAGGTGATGGCAATAATTCTTCTGAGGTTCATCTTGGCAGAATGTTGCAAATCCTAAGAGAGTTCTCAACGGATCCAATATTAAGTATCTATGTTGTCGATGATGTTTGGGAATATATGAACGCCATGAAGGACTGGAAGTGCATTGTTTCCAGGCTCCTAGATGAAAATCCAAGATCTGAGCTGACTGATGAGGATGCCACAAACTTGGTTCGTCTTCTTTCTGCATCTATCAAAAAGGCAGTTGGGGAAAGGATTGTTCCTGCCACAGATAATCGAAAGCAGTACTTCAGTAAAGCTCAAAAGGAAGTATTTGAAAGCAACAGACGGGACATAACTGTTGCCATTATGAAGAATTATCCCGTACTTCTGCGCAAGTTCATGGCTGATAAAGCAAAAGTTCCATCTTTAGTTGAAATTATCATACACATGAATCTTGAACTTTATTCCCTGAAGAGGCAAGAGCAGAATTATAAAAATGTTCTTCAACTAATGAAAGAAGCATTTTTCAAGCATGGCGACAAGGAAGCATTGAGATCATGCATGAAGGCAATTAACTTATGTTGCACTGAGAGTCGAGGGGAGTTGCAAGATTTTTCTCGTAATAAATTGAAGGAACTTGAAGATGAGCTTTTTGCAAAACTAAAGCATGCTATGAGAGAGTTAGAGGATGGTGGTGATGAGTACTCTCTTCTTGTAAATTTGAAAAGGTTGTATGAGTTTCAATTATCCAGACCTGTTCCTATGGAAAGCATATATGGCGATATTATGATGATTCTTCAGAAATTTAGAAGCATGGACGATGAGGTTGTATGTTTTCTACTTCTCAACCTATACTTACATTTAGCGTGGTCTCTACACTCTATCATAAATAGTGAAACAGTTTCAATAGAATCTTTGTCATCCTTATTGAATAAACGGAATGCATTGCTTGAGCATCTGGACCAATACCTGAATGATCCTACTGAAGTTTGTAAAAGTGGTAATCAGCTGGCCTATCGAGTTTGTACCATTCTTGCGGAGATGTGGTTTTTATTTAGGAAGGAAAATTATTCTTCAACAAAACTGGAAAGATTAGGCTATTGCCCTGATGCATCTACTGTTAAAAACTTTTGGAGGTTATGTGAGCGACAGCTAAGTATTTCAGATGAGTGTGAAGATGAGGGTGCTAGTAAAGAATATGTGGAAGAGACAAATAAAGATGCAATCATGATTGCTGCCTCAAAGTTGGTTGCAAGTGATACAGTTTCTAAGGAGTATCTTGGTCCAGCAATTATTTCTCATTTTCTGATACATGGGACAAGTGTAGCAGATATTGTTAAGCATTTCATTGCCATGTTGAAAAAGAAGGATGACAATATCCCCAATATCTTTCTTGAAGCCATGAAAAGAGCCTACCATCGACATACGGTAGAACTTTCGACAAATAGTGATGGGCCTTCAACGGGCAAATCCTTTCTAGAATGTAGGGAGTTGGCTGCTCGGCTTTCTGGAACATATGTGGGTGCAGCTAGGAACAAGCATAGATTGGATATTTTAAAGATTGTTAAGGATGGAATTGAGCATGCCTTCTCTGATGTACCGAAGAATTTATCTTTCCTAGAATGTGCCATTCTACATTTTGTATCCAAACTGTCTACACCTGATATCCTGGAAATTATCAAGGATGTCCAGAATCGAACAGGAAATATTAATACAGATGAAGACCCAAGTGGGTGGCGCCCGTATCATACATTTGTTGACAGCTTACGTGAAAAGTATGCTAAAAGTGATGGTTTGCAAGATGAGAAAGAAGGAAATTCTACTAGACGTAGGGGTCGGCCCCGTAAAAAACATAACTTACAGGGAAAGAGACTTTTTGATGAGCAAAGTACAAGTGAAGAAGAAGAATCAATCAGTGCATCTGATCATGAAGATGTACATGACGAAGAGAAGCAAGACGAGGAAGATGAGGAAGAAGTACCTCTCATCCATTCAATTCGGTCATCCTCTAAATTGAGGTCACTGAGAATTTCAAGAGAGGAAAAAAAAGGGACTTCTACGGGAAAAGCTGCAGGGCTTCCATCTACATGAAGAGAACTGATTGTCTGAATTTTGCATCGAGCGATTTATCATGATGTACAAATATCCCATATAGCAGTTGTGTATTTGTACACAAAACGAAGTTATGTTGACTTAATCTTAATTGAGAGGTTGCTCGTGAAACTTTCTGAAGTGCTACCTAAGGAGTTGTGGCTATGTTGATAATAGTCGTTAATCTAGTTCGCCTCAAACATTTATGTGATAGGTTTAGAGTGTCATTAGCATGCTTTTGTGCTTAGTTTAGCTTGTTTTCTTTAATTTTCATGTTGCTTGTTTGTAAGTGGCATTTTGCACTTTGCAAGTCAGTTCAATATGAAAATGTTTAAAATGTACTATAGGAAAAACACACTAGTCAATTCTCTAATTAAGTCTTGTACTCTTTGCAAACCAAGCTTTCTTGTAATTGGCGGTCTTCAATGCTTTTTTTATTTAATGATTTTTTAAATATTTTTC

>cucumber_newGene_39 cucumber_newGene_39.2

CAAACCCAAAAAGCACTTCCTCTTCTATATACATACTACTTTCTTCCTGATCTTCATATACATACCTTGTTGTTTCAATTATAAAGACATCAGTCATGGACTACCTAAATACAAAAACCTTCTTCCGTCCTGATCAAAATTATCAGACAACCTCCTCCCACTTCTCTTTTCCATTTGGGTATGTCTTTCTACTATATGTTTCATCATAATCATATCCATCCTCTCTATTTTGTTGGGTTATATTTATATTTCTCAGGTTTGGGGTTGGAGATAATCATGAGGTTTTGTCTCCTAATTCTGTGGCTTATGTTGAGAATTACTTTCGCCCCATGATACGAGAGGAAGTGGAGGCAAAGATTAATGCTCGCTTTAACGTCTCTACCAATTACCAATCATGTTCTAATTCAAGTACTAGTAGGGAAGGAGTACTTGGTGAAGATCGTGCAGTTGTTATTGTTGGTGGAGGCCGATTGAGAACGGAGAATCAACAACAACTGAAGTTACGTTTCACGAACAAGGTTTCATCCACCGTCTTCACCGTAAATGACATCGAAGCCGAGAATGGGGAGGAGCTGCGTGTTGAATTATTTGATGCTGTCAATGATCGCATCATTGACGCTACTCATCCTTTGTCGTCGGCGTCGATTGAGGTTGTTGTCCTCGATGGAGAATTCAACGACGGGGAAGCTATAACTCAATCGGATTTCAACAGAAGTGTTGTGCCAGAAAGGCTGGGAGAACGACCTTTGTTGGTTGGTCGTGATAAGAGGTTTCGTTTAGAAAAGGGAGTTTATTCCATCACCGATTTGTCATTCACTAGAAACTCAAGCAGGAGCAGGACTAAGAAGATTTGTTTGGGACTAAGGGTTACACAGGACTCCAACAACAACTATCCCACAATTGGACACACCGTGTCTAATCCTTTTAGAGTGAAGGATCACCGAGGCCAACTGAACAAGAAACACCATCCTCCAAAAGGAGAAGATGAAGTATGGAGATTGGAAGGGATTGGGCGAAATGGCGAATATCACAAGCGTCTAACTTCCCATACTATCCTAAATGTTGATGACTTTTTGAAGGCGTATCAAAAGGACTCTCGTTCTCTAAGAAAGTGGTTAGGAAATAGAGTTTCAGAGAAGAAATGGAAGTCAATGGTAAAGCATGCTGAAGAATATGTTCCTATCACCAACGCCTTCGTCCCCACTTTTGATCCACTCACCAGCTTTCAGGTCTCTTTAATTCTCATCTATCACATCTACATTTTTAAATGCCTCTTTTCTTTCTTTCTTAGAAATATTTTTGAAAAGTTATGACTACATATTTCTTTTCATATCTTTATAAGTTGGGGTTTTCAAACTGAGAATTGACAATATAATATTATATGAAAAAAGCAGCAAAATTTGGTGGAAAATGAAGCCATGGGAGGTGTAGAAGAAGTTAGCAACCAAAACAACATTGGCGATGGGTTCCAAGATCATACATCATTATCGGCTGATGTTATTCAAGATCATTTTTATCGAATATTTCAAGGATTACCCAATACAAACATTGTTGTCACAAGGAGAAGCTTCAACCTCAAATGGCCTATATAATTATGTTGATCAACTTAGTACAAGTACATGCAACCAATCCTTTCAAGGAGCTGCCTTCAATAAATTGGTGCCTAAATCTAAATGATCCAAATTATTTGATCTAGGAGCTCTCAAAAGAAAAAAGAAAAAAA

>cucumber_newGene_4 cucumber_newGene_4.1

GTCAAAATCAAACCTTCCATTTAGTCTCTCATAGTCCCCTTGTCTTCATCCCTTCCACGAAGGGTTGCCCATGAATCAATCAAGAAGCCATGGAAGATTACAGCTCATTCCACTATACCCAATACGGCGGCGCCCGGTTCATGCCGTTATCCCGGTAGAGGAGGTATCGGAAGCAACGGAGGTGGAATTTTAATTGTCCTTAACAAATAGCGCTTCTAGTTGTATGCTTTTGATTGTTTTTGTCTTTGCAGTGTAAAGTAGCAGGAATTGATTCAACATCTTTTTACATTTGTTTGTTTCTGTGTGTTTGAATTATGTTCTAGGATGAACCAGTACAATCTCTCTCTAAACCAATCGGAACTGATGGCAGTTATCTAAGAGATTTATACCTTTTTTTTCTAGCCCACAGATTTTATATCATTTAGGTACTCTGTGTATGTTTGTTTCTGTAGTTTTTCTTTTGTTGTGTAGGTTTATAGGAATTGTTTTTTCTTCTGCTTCTTTTAAATCTAGTGGGATGTATCTTCTCAAAATGTAATGGAGATTTAGCCACTAGTTTTGTTAATAACTATTACTTTGTCAGCAATAGACAGAGAATGTATGGTTTACTTAACATGGGAACGAACATTTAGTGGTTTTTATCACAGCATTAATCAGTTATGGTGTTGGAATGGTCAAGCTTGAATTGAAAATGTAGTTTTTTTTTTTTTTTTTTTTTTTTGAATTATATGGACTGCAGATTGAACCAACCATGTTGCCTGGTTGGTTCCTTGGTGCTTTGCTAATTCTTGCCTATGCTTATTGTTTTGTTGCTTGCAAAGCATGTTTATGGTTTCTTATTGCATAAAATTTACCTGTTTCGAATTGTATCTCTTTGTTTATGTGTTGTCAATCATAGCATTTTTAAGGGAACTTAATCTTAAGCTCCCACATCACTTCGAACACGAATAATATGGATCATGCTTGCATTTTCTGAATGTGTGATTAATCATTTTAATTTTGTTTCAATGAATGAACTGTCTTTCTATTTAGCTCGTACATTTATAGCTTGGGAGCTGGGGCTCATGGGTGATTGATTTAATCGTTTTTATCATTTACTTGCGAGTTCCATTTTTGCTACAAGGAAAATTACGTTTGCAATATTGATTCTCTATGCTTCACAGGAAATCTTGTTTGAGATTTTGTTTCATCGATTAGCACCTTGCATTAGAACCATGGGTCCATAGTTATTATGTTCCTTGACTATGAAGCTCTCCTTGCAGTCAGTATGCTTTCTCATTACTTGAGAGCCCAGTTAAGATATACGTCATTCCAGTCAATCCAAATTCTTGAGCAAGGAAAAAATGGGCAAGATTAGAGAGTTACTCATTCGTACTGCAAAGACAATTATTTTAGAACATTCATTCTCTTTAACCCACCATCGTGTATGTCTATGTGTGCTTTTGAACTAACATCTTAATCATAAACAGCAAACTTGTTTATGTTGCGGTTTTGATGCAGAAGTTGGATTTTTAGGAGTTCACCTGGTCGAAGGTATTTTAAACAACTATGAGATACTAACCCAAAATACTTAATTAGACATGGCACCATTCTGTCAAAAATTGATGTCTTTCTGCGAACAACAGATATTGATACTATCAAGTTCAATCATTTGAGAGAACAAAAGAAGTTTGCTTGGTTGAGAGGATTCAAAGGCCTGGGTCGATGCCAAATATTACATTCCTAGGAGGTTGACGTCCAACCAACCCCGGGGTCATTTAAGAGCATGACGGTACAGCTCTGGCTCCATCTCTTCCTTATCATTGATGTAAGAGTCCGTTTTCACTGCTTATCATCAGATGAGAAGGTTTATGTGTGTGGCTGACAATGCTTTGTTTATCCTGGGCTTCAGTTTCCATCTTCTTTGTACTAGTTATGATTCTTGGTACTCAGTATATGGATCATCTCCCATTACAGATCATTGTTTACCTTGTTTGTTCTATTTTTTTTTCATTGATTTATTATTTCCTTCGTGTATGAATAATGTTTAGAATGAAATCACCATGGATTGGCCTGACCAATGAACAAAAAAAGTAAAAATAAA

>cucumber_newGene_40 cucumber_newGene_40.1

GGAGAGTTCAATTTGTTTTTCATTGTTACTCACAAATTTTGATGGCCAATTCTCTCTTTAGATTGTTCACTTTATTCTTCTTTGTTCTCATTTCCATCTTCCTCCTTCATCCCAATGTTGTTCTCTGTGATGTGAAAGATGATAACATTCTAAATGCCATTAACGAATATAGGGAATCAAAGAATCTATCAAGGCTTTCTTATAACAGAAATGCAGCATGTTTGGCTGGTAAACTCGTTTATAAGCTAAGAGATGAGCCTTGTTCAAGTGCTGAAAATTTCAACAAAGAAATTAGCTCAGAAACTAAGCTTGCAGATTTCCCTAAGCTCTTACGAAAATGTCACATAGCCTACAACTCCTCCGTTGACGGCATCATTCTCCCCTCTTGCGTCCGTGGACTCGATGCTATATCTGTCACCAGTAACTACACCCGTTCCCACGACGGCGAGTATATCAACGATCAGAACTATACTGGTGCTGGGGTTGGGACGATTGACGATGCTTGGGTTGTTCTCATTCTTAGCACCAACACCTCAACTGGGAACTATGACAACAGTGGATCATCGTCTTTGGTTGTTGCTGGAGGTGGTCATATTGGTGTGATGGTTGCATTGTTAGGGATGTTTGTTTCATCTTTGTTATTTTGATTTTCTTTTCTTGGGTTGAGTCTTGATCTCAAGCTTGTAAAAGGTCTTTGGTTTTGGGTTTTATGCATTGTATTTGTCATGAACTGTTGATATATTTGTTGTCTTTTTACTGATTCTCCTGAGTTAATCAGTACAGTTTATATATATAATCAAAATTTGGGCAA

>cucumber_newGene_41 cucumber_newGene_41.2

GTTTTTTTCTCTTTGATTTTTGGGAAAAAGAAACTATATTTGCTCGCGCTTCTTTTGAATTATCGATGGAAGAGCCCAAAATTCTCATGGGGCCAGATTGGAGTGCAGGCCGAAGATCACGACATTGTCTTTAATCTAGCCTTCAAATTCCAGTGCTTTGTGTGATCAGACTTGAAGAAGGACATGGCAGAGCATAAGAATATTTTCCCACATGCCACCAAAAATTGCAGCACAAACCCAGCCAAATTATTCCATATTCTTCTGATGAAAATGCCAGGTAAACCCTCGACATGCTTACTAAAATGCAGCATATTCTCATTCGTTATCTTATAGAGAACGTCGATAAGTGGGTGTAGTGGGGAGCTTGGTACTTACAAACGGCTGCTTCCTTTTTGCCCATCCATCAGATGTTTGATAAAATGGCTGAATCATTTTTCAGACGTACTGGTTCCTCTGCCGATGTCAATTCCCATGGTGGGGTTTATCCTATTGTCTGGTTTCTTCAAGAGGAAGAACAATGCGCTTTCCTTTGTCAAGAAGGCCCTCAGGTTTCTAAAGGTGTCTTAAACAAGGATGGTCCACCAGAAGCTTCGAGTACTAGAAAAACTCATTCCCATCAATGGTAAAATTGGACTCAAAAGACCAGCACTTGATGTTTTGTCTCCGACATTTCTACACCAAACATGGCCATGGAACTATTAGATCACTGGAATGATGACTATGAAGGGATCATCATTAATTCAGAGAGCCTACCCTCCAAGCATCTCTCTTCCAATTGAGACTCAAGTGGAGAAAAGGGATGTGGCTCAAACTTAGATACAGAGCAGGCTGAACTAGTTTCTGTGGCTATTAACGAAGGGTTCAACTATCATCATGCTGAAGCAGGATATGTCATGCTCACTTACTGGATTCCTGATTTGCCCTCCATGCTTCCTTCTGGCCCCTCCCATCATATTGGTGTTGCAACATTCCCATCAATGACAACAAGGGGGTACCTTTTTTCTTCTTTACATCTCATTTTCTCTCTCTGGTTAAAAGAATAAGATCCTCTTATATCCTTATTGCTATGATATATTATCATTCACTTTAGATAGTAAGCATTTAGTTCATGAGTGTTAACCCTTGAATCTATGTAGTTTGAAAGTGAAAAGCTTAAATTGGTCTAGCACATGTTTGACTTTGGATTTTATAGAAGTATAAATATCTCAAGTCTCAATTTGTCTACCAACTTCTAAGTGATAGGTCTCATATGTCTAGTCTCAATTTGTTTGACTATGTAGTATGCTCACCTACCCCAAATTCCCAACTTCTAAGTGATAGGTCTCATATGTCTCTTTAGGACTAAAATACTAACGAATTTGCTTTTGAACGATATCAATACATATACCACACACGTTGCTCATATTTGTAAACTATGAGCCTATATACCTTCTAGTTTAAGCACTTTCTCTTGCAATATGTTCGATAAAAATGTTCTTACAACTTTCTATGTATTTAAAACATACTTTAAGTAGTTGTTATGCTAGTTGGTTTGACTTGGCTTGAGTGGGCTCTTTTAGCCCAACCCACGTTCGGCCCACTGGCTTTGAGAACTGGTGACTTCATTTAGCAAGTCAGTGGGTTTCTCTTCGACTCTTTCATATTTGGTTGGTTGTTCTACATTCTAAAGTATGCCCATTAATTACTAATAGCTTTGTTTTCTATTAATATATTTTGAAGATTTTCAGACAATCACTTAAAATCACTTTCTAGTAATTCATCTTTAAAGTGTTCCTAAAACAATAGAAAGAACACCTTTGAAAGATGCTGTACTAAACACACTTGTAAAGCAGTAATGTTCCTTAACATATTCCTCAACTTACTAAAAACATACTTTAAGTAGTCGGTTCTATCATATTCAAAATCACTATACTATAATATCATTTTCTGAGAATTGCTTGTTTGATGATGTGCCCAACAATTATGCATATGACCTCTTTGTCGCGAGTCAAGCAATTTGATGCAACTCAAAACATACTGTGAATGCCTTCTCTTCTTTATCACTACTTTATCTTTAATCAGGCTCTAAATTTTCAGATGGATTCAATGTTCTTGCTTCTTGGGAATTGCGTTCAGGTATGGGTTCCTTCATTTATTGCCAAATTATGTTCTGTTATTAGAAAAATATCAAATTATCAGACTATCATTCAGGAATTGCAAAAGAAGGAAATTTGAATTGCTTTATTATGTCGGTTGGGGCTTGTGTGACACACAGATCCTTGCATGCATTTTTGTCCATTAGTTTTAGTTTTAGTCCTTCTAACGTACATTATACTTCATTCTTAGTCCTGGTTGTGGAAAAAATATATTGTTTCAAGTAATTTAAAATGTAATCTATCATACATAAATCATATCTAGATTATCACTTCGATGATAAGAAATAAAGTGTATGCGTAGGAGTATTTTAAAAGAATATAGACTTATTTATTCCTTTTATAGGCAATCTAACTTTTTTCCTCACTTTTCTGAGTTCTATTCACATTAGGGAATAGATAATGGATGTAATGCCTGATTTTGGAGATAAATGAATGAACGAGGATTGCAAGTTAACAAAGGCTTAAACGAATTGATAGCTACCTGTAAATCCTCTACCAACAAGGTGTATGTTTTTTGTTTATGGCTCAATAATAAGAACTTCAAAGTAAACCTAGGGAGAGGAATAAGCAAGTTGGGAGGATTCAAGACAAACAAGGATGGGAGTGTGAAGAAATTCGGATTCCAAATCTTGATACAAGGTGTTAGAATTGACATCTATATTAACAGAGTAATTATTCAAATAGGTCACAGATTTAGGTATTAAAACTATATTAAACCAACCAAATAAACTACACAAGAAAAGTATTAGTGAAATTAATTTGAATGATGAAGCAAGATAAGGAAACTGAAGGGGATGATGATTAGTGAGGGTTTTTTTTGTAGAACCAATGGATGGGCTTTTGATGAGTTTATTAGTATGAAGCCACCAGTTTTAGGAAGAAGACTAGATTTTCATAGCCGCCCATGAGGGCCACTACACTGGATTCCTG

>cucumber_newGene_42 cucumber_newGene_42.1

TTATCAAATTGGATCAAGTGTTGAGGCCGTAAAGAGAAAGTGGAAAGGAGAAATGCCAACGTAAGAAAGTGGAAAAAGAAAATACCAAAATTGAAGATAGGAAAAAAACATGAAAAGTGAGAAGGCTGTGATGATTATTGATTTAGCGTTTAGAAGAGTTCGTACCAAGAAGTTTGTATTACCCATCATTACCCTAGAGCCGGACTAGGGTATGATGGGAAAATAAATACCCATCATACCCTAGTCCGGCTCTAGGGTAATGATGGGTAATACAAACTTCTTGGTACGAACTCTTCTAAACGCTAAATCAATAATCATCACAGCCTTCTCACTTTTCATGTTTTTTTCCTATCTTCAATTTTGGTATTTTCTTTTTCTACTTTCTTACTTTGACTAATGGTGAGATTAGCCAAATGGATAAAATTTTTATTTAAGGGATGAGGTGAGTTTTCATCTCTCTTTGCCATTGTTTTGAGAAATGATTGATAGTAAATACATTGAGGTGTGATGTTTGTTTAATTGGTCGAATTGATGTTTCTTCTTGAAAGCGATATTGGTGAATTGGTGTTAGGATCCTTGAGTTTCGTCGTAAGCTTCGTTGGAGTTCGATTCTCCACTTTTTGGGTAAGTTGTGGAAATTACATATAGGATGAATTGAATGGATGTATTTTTTTGAGGAAGTGATTGATTTTTGGTTAATTATGTTTAAGTTGGTTCATGGCACTTTAGTTTGCTAAGGAAGCTTAAGTGATGTTAAGATAAAGAGATTTCTACTACTGGAACCATATGTAAATTTCAATGATTAAAAGAACTCAATTGTTATATGTAAACGGAATGTGTTGATGGAATTGACTGAATTGATTGAATTTGAGGATGGTTGATTGTTATGTTAAAGTATAGGTAGTGTACTGTTGGCTGAATTAACTGAGGTGGGTGATGCTCATTTGTGAGGTGGGTGATGCTCATTTGTTCAAGAAACCGTTATGCTAATGAATGATTTATGTACTGGTATGAGTAAATTGACTGTATATTTGTTGGACTAAAATAATTATATGTGGATGGCAACCAACTTGGACTGCATTTGGACTTGACTGTTTAAGATGTTAAATTGGACAGAGATGGGTTGATTGATATGTAAATTTAAATATGATTGATGTTTAGTATGATTGATTGAACTGTGATGTGTACTGGTGTTTGTAAAGGAGAAATGTCGATAGTTTTAACTGTGATTTGATGTGATTGTTATGAATTATTAAAATGATTGAGGGGAAATGGTTAGCTTTTTCTATGGGGTAGTGTGCCTTGCAGGTGTCCCACGGAATCACCACTTGTTGTGTATCCTTTAGGGGCACTAGACTGATAAGTGCGTTATTTGGAGCACAAGACTGATTATGTGCATCCTACGGGATCACGAGATTGTTATGGTACAGGTAATACGAGAAATAGATCGACAAGGGACAAAAAGGATATGTGACCGTTATAAGGGGGCCTAGTTTTCATAAAATTTGCTTCTGCCTATGGTTTTATTTTATTTTTTATTGAACTGAGTTTATGGATAGTTATTTTGTAAACAATACTTTTAGAATTTTATTTGTTAAAATTGTTTTGTGCTAAAGTAAAGACTTCAACTTAGATAGAAAAGTAAAGTGGTTAGATGATATGAGAGCTCAAGTTTTAAGTTATGTGTTGGTGACTTTAAAGGGAACTAAATACATAGTTAAGATAAGTTTATTCTAAACACAATGAATGGTTTTAGGATGAAGGTATTGAGGAAAGAACAAGAAAACTTCAGAGCATAGTTGTATTAAGTTGATTTTGAGAAACTGTTTTGAGTTTATCGTGAATGAAAAGTTACTAGTTTGGAGTTTGAAAAATGGCATGTAGTTTAACCATGCAAACATAGTTTGTTCAACAAGGAGAAATGCAAAGCCAATGTTTGGAAAACAGATTCCCATAATGGATTTAGAGTTAAGATAGTGCACTAAGAGAAAGATTAGCCTATTGGATTAAGTTTTTGTTTAATGAATTTGATTATTTTCCATGGACATGGTCATTGAAAAAGAAAAGTTGGCTATACTATGGTAAAAAGAACATGAATGACTATAAATGGTACAAATAGAGAAGTAAAGATATTTGAATTTTGGATTGAAGTGTGGCATTCGACTATAACTTTGGATTGAAGAGTCAATGTTTAGATAAAGATTATCAGAGTGGATTAAGAGTTAGAGATATAATGCACTAAGAGAAATATTAGCCTATTCGATTATGCTTTTGTTTAATGAATTTGATTATTTTCCATGGACATGGTCATTGGAAAAGAAAAGTTGGCTATACTAATGTATATGGTAAAAACAACATGGAGGACTGTAAATGGTACAGATAGAGCTGTAAGGATATTTTAATTTTGCATTAAAGTGTGGCATTGGTCTTTAACTTTTGAAATGAAGAGCCAAGATTATCAAAATGGATTAGGAATTAAGATAGTACACTAAGAGAAAGATTAGCCAAAGATGTCTAACAATCCTTCATATGGAATTGTAGAGAATTGGTCAAGGAGATAAGACTTAAGAAAGTAGGCAAATTAAGGTACTTTTTATGATTACTGAATATTTATTCATTGTCTTTTTTTATTGGTATGTTATTTTTTATTTGAACTTGTTGGATTGAAATTTTCCTCCAACCCCAACCCCTTTTGTGAGAAAGGTTTGTTTGTATGACTCACTAGGGGTTTGTGTATTTTGTGATATCTTTGGGATAATGATCCTAGTGAAGTGCAGCCCCATTTTAGATTTCATATTTCTTTCTTATCTTCAATTTTGAAGACTTTTTGTAAGGCTAATTCAGTTTTATTTTTGAATGGTTGGAGGCCCAATTTTTTCATGCAGGTAGCTGATGTATTCTTGCTCTAGTGGCACTGATGGCCTTTATGATTAGATTTCATCAAAAAGTTCGTTTTCTAAAGTATACTATATTAATGAATGGGGTGAGGGAATTTGAACCAATTAGTTCGAAGTTAATAATACAAAATGTCAAACCCTATGGTTGTTTTATCAACCTAAAGAGAAATCGCACTTAAATGTTTGATTAAACA

>cucumber_newGene_43 cucumber_newGene_43.1

CAAACTTTATCTTTTTGGTTAATTAGAAATTTTTATCTTGTTTCTGGGTAATCAAACGAATCCCCTTTTTTCAGATTCTTCTCTTCCCCTACTTCTTTTTGCTTAATTCAATCCACATCTTAATTTGCCTGTTCTTCTTTTGATTCCGGAGGTTTTGATCTTTCGATTGCGCGGTCTAGGTCGAACAGAGGGGATTAACAATGGGATTCTTGATCACATCCCTGATTTTCTCCGTGATTGGAGTCATCGCTTGTTTTTGCACCCTAATTTGCTGTAATAGAGGCGCTTCAACTAATTTGTTTCATCTTACTTTGATCATTACTGCAACAATATGCTGCTGGATGATGTAAGTTTTTTGTGTCTTAACTTTTTATTTATGAAATGTCTATTGGTCGATCGCGTTGGATATGGTTGTGCGCAATATTTTCTATTGACAATGGACCTTCAAATGCCTCTTTACTGAAATGTTCAAATCTTTGATGAAGTTTCAGTCAGAGAAACTTACAAAATCCTTGTTATTGTACTGGATTCCATCTAAAATTGACTGGCTACCATTTTTTGTTTTTTTTCATCTCCATCTAACCAGAATGTACAATTATAGAATATAGGCTTCTCATTGTGAAATGATCCAGCCTAGCATCAATAAGCCTCTAGGTTCAGTTTTCACCCATGATTTTTCCAGACATTTTGTTGAAGAATAGTCTCTATGTCTGTTATGGAGTATCCTTTCGGATAGTATATCTTTAACGCCACGATCTCTTTTTGAAACCTGTTATTGAATCTATAAACTGAGCTACTCTTGCCACAGTATCTATTATTTTATTACTAAACACTAAATATGTATAATGTGTAATGACTGTTGATGTTTTTTCTTTGCATTTGTTTGAATCTCGGTGGGCAATTGTATATCTTGCACAAATGAAGCCACTTATTGTTCCCATTCTGAATGACTCGGAGTGAAGTGTTTATTTAAGTTGTGTTATGAATTTTAAATCATCATGGGGTTGCTGCTGATGCACCCTAAAGAAGAAGATATGTTCAATTTTTCAGATTCCATGTATCTATGTTGTATTTCAAGGTTGAATCTGTGCATTTCATAATTTGAAGATGACCTTTCAATCGGGCTTCAGATTTTTGGAGGATCGACTGTTATTCATAGCTACTTATTACACAGATCTCCATTTGTGAATTAAATACTTTTGTGGTTGGGGCTGTTTGTTTCATTCTCCAACTTTTATCAATAATAATATCATTCATTTATTAATAAAGTGATCC

>cucumber_newGene_44 cucumber_newGene_44.2

AAACACCAAAATGCGAACTTGCCCTATGATCAATTCAACTGATTCCCTTCTCTTCCACTTCTTCTTCAATTTCTTTTGATTTACATTTCTCGTTGCTCCCGGAGAAGAGAGACTGTCAGGAGCTCGACGTTTTCCGGCGAACGATCCTGGGCTTTTCTTCCCCTCCTCCGACTGCCTTGCCTTTTCGTTTCGAAGGAAAAAGCATGGCTTGTGTATACATACCGGTACAGAACTCGGAGGAGGAGGTCAGGGTAGCTCTCGATCAACTTCCCAGAGATGCTTCCGACATTCTCGACATACTTAAAGCCGAGCAAGCTCCGTTAGATCTTTGGCTAATCATCGCGAGGGAATACTTCAAGCAAGGAAAACTAGAACAATTCCGCCAAATTCTGGAGGAAGGGTCCAGTCCAGAAATTGATGAATACTATGCTGATGTTAGATATGAAAGAATTGCAATCTTAAATGCCTTGGGTGCGTACTACAGCTATCTTGGAAAAATTGAAACAAAACAACGAGAAAAAGAAGAACATTTCATTTTGGCTACACAATATTACAACAAAGCATCCAGAATTGATATGCATGAACCTTCAACTTGGGTTGGAAAAGGTCAGCTTTTATTGACGAAAGGGGAAGTAGAACAGGCATTTGCTGCATTCAAGATTGTTTTAGATGGAGATCGTGACAATGTTCCTGCCCTTCTAGGACAAGCATGTGTTGAATTCAACCGTGGACATTATTCTGAATCATTAGAGCTATACAAGAGGGCCTTGCAAGTGTATCCCGATTGCCCTGCTGCTGTAAGACTTGGCATAGGCCTTTGCCGCTACCAATTGAAACAATATGGAAAAGCTCAGCAAGCATTTGAGAGAGTGTTACAGTTGGATCCAGAAAATGTTGAGGCTCTTGTTGGTCTAGCAATCATTGATCTGAACACAAATGAAGCTGGTCGAATCAGAAATGGAATGGAAAAGATGCAAAGAGCATTTGAAATATACCCTTTTTGTGCAATGGCTCTGAATTATTTGGCAAATCACTTTTTCTTTACTGGTCAACACTTTTTGGTGGAGCAATTGACTGAAACTGCACTTGCCATTACCAACCATGGACCTACAAAGTCTCATTCTTTTTATAATCTTGCTCGGTCTTACCATAGTAAGGGAGACTATGAGAAAGCTGGCTTGTACTACATGGCATCCGCTAAGGAAGCTAATAAACCCCGTGAGTTTGTATTTCCTTATTATGGTTTGGGTCAGGTTCAACTGAAGATGGGAGATCTTAGAAGTGCATTATCAAATTTTGAGAAGGTTTTGGAGGTTTATCCAGACAACTGTGAGACATTGAAAGTACTTGGACATATTTATGTTCAGCTTGGACAGGCTGAGAAGGCCCAGGAGTCTTTAAGGAAAGCTACAAAAATTGATCCACGTGACGCACAGGCTTTTTTAGATCTCGGAGAATTGCTGATTTCCACTGATGAAAGCGCTGCTTTAGATGCCTTCAAAACTGCTAGCATTTTGTTAAAAAAGGGAGGTCAAGAAGTGCCAATTGAAGTGCTCAACAATCTTGGGGTTCTTCACTTTGAAAGAGAAGAGTTTGAGCTTGCTGAGCGAATTTTCAAGGAGGCTTTAGGCGATGGAATTTGGCTAGATTTTATTGATGGTAAAGTGAGATGCCCTGCTATTGAAGCAAGTGCGTCTGTTCTTCAATACAAGGACGTTGAATTATTTTATCAATTGGAGAGGGAAGGCCGTGCAATTGTACTACCATGGAAGAAAGTCACAAGTCTATTTAACCTGGCTCGATTACTAGAGCAATTGCATAGAATTGAAGTTTCAAGTGTACTCTATCGCTTGATTTTGTTTAAGTATCCAGACTATGTAGATGCTTATTTGAGGCTTGCATCCATTGCAAAAGCTAGAAATTATGTTCAATTAAGCATTGAATTGGTTAATGATGCTCTGAAGGTGAATGACAAGTGCTCAAATGCCTTGTCTATGCTAGGTGAGCTTGAGTTAAAAAACGATGACTGGGTTAGGGCAAAAGAAACTTTTCGGGCTGCTGGTGAAGCCACCGATGGAAAGGATTCTTATGCCACTCTTTCTCTGGGGAACTGGAACTACTTTGCTGCTCTTCGAAATGAGAAAAGAAATCCCAAGTTGGAGGCTACTCATTTGGAAAAATCCAAGGAACTGTATACGAGGGTTCTGGTACAACATCCAGCTAATTTGTATGCTGCTAATGGCGCTGGGGTAATCTTGGCTGAAAAAGGTCAATTTGATGTTTCAAAAGATATTTTTACACAAGTTCAAGAAGCTGCAAGTGGCAACATTTTTGTTCAGATGCCAGATGTGTGGATCAATTTGGCACATGTTTATTTTGCTCAAGGAAATTTTTCATTAGCTGTTAAAATGTATCAAAATTGCTTGCGGAAGTTTTATTACAATACGGATTATCAAATTCTTCTATATCTAGCTCGTACGTATTATGAAGCTGAACAGTGGCAAGACTGCAAAAAAACTCTACTAAGAGCTATTCACTTGGCACCTTCAAATTACACTTTAAGATTTGATGCTGGTGTAGCAATGCAGAAGTTTTCAGCTTCAACACTACAAAAGACAAAAAGGACAGCAGATGAGGTTCGCTCAACGGTAGCAGAGCTGGAAAATGCTGTTCGTGTTTTTAGTCAGTTATCTGCTGCTTCCAACCTCCACTTTCATGGCTTTGATGAGAAGAAGATTGATACTCATGTTGGTTATTGTAAGCACTTACTGGAGGCTGCAGGAGTTCACCTCAAAGCAGCAGAACATGAAGAGCAGCAGATTCGTCAAAGACAAGAACTAGCACGCCAGGTTGCATTGGCAGAGGACGCTCGTCGTAAGGCAGATGAGCAAAGGAAATTTCAATTGGAGAGGAGAAAACTGGAAGATGAGGAAAAAAGGATGATGCAACAGGAGCAGCATTTTAAACGTGTGAAGGAGCAATGGAAGAGCATCACACCTGCTAAACGAAGAGAGAGGTCAGAGATTGATGATGATGAAGCTGGTAATAGTGAGAAGAGGAGAAGGAAAGGTGGAAAGAGGAGAAAGAAGGACAGGAAGGGGAAATCACATTATGAGACAGAGGAGGCCGACAATGATATGATGGATGATCAAGAACTATACAATGAAGACAATAACATAAGTTATAGGGAGTCCCGGAGCCAAGTGAATGATCAGGGCGATGATTTTGAAGGGAATGATCAGGATGCTCTTGCTGAAGCTGGGCTCGAAGATTCTGATGCTGAGGATGAAGCGGGTGCACCTTCATCCAATGCCGCCAGACGAAGGGCCACATGGTCGGACTCTGAAGAGGATGAGCCTATAGATACACAGCGAGAGTCAAGACTTCAAAGAGAAAACTCCGCAGGACTTGAGGATAGTGATGGAGAAATCAGATGAGATGGCAATAGTTCAAACTGGATGCGATCATTGACTCTCAAACTTGGTTCAGGAGTTTAATAGATATCCCAATCGGGTCCTACCAGCTGCCCTTAAAGTTAAGGTGAACGGCAGCAACTTGACGGATTATTACAGCTACATTGGCTGCTTCTGCTGCCTAGCATCCAAGGCATCAATGTCTTGACTAAGCCATTTGTGATGTACGGTCAAAAGAAAAGTAAATTTTAAAAGATCTCTCTTTCTCGCCTGATGTGCCTCTCAATTTATTGAATTTGTTCTGTATTTATGTAGTTAGTGGTTCTATAAGAACTGGAAATTAGATAGAGGACCGATGACTTGCATTCAGGACTAACACGAGTAGAGTAATGAAATTGTATTTCTTATTGATTATTGTAATATTTACCGTTGGTTAATTAGTAGAAAAAAGAGATGGGTTTTGTTTTTCTGCAACAGGTTATTAAAGATTTTCGAAGGTTGCCTTGACTCCTAACTTAAATCTTTA

>cucumber_newGene_45 cucumber_newGene_45.1

CAAATCTCTCTCCCTCCCTCCCTCTCTCTCTCACTATAAATATAATATTCAATTTCATTTCATTTCTTGATCGTCACATCAACAACCCTCAAGAATCCTCTTTTCCTTTGTTTCCTTAATTTAATTTTAATCTCTCACTGCCATTGGCGGCGGCGGCGACACACCCTCTCCGGCAAGATGACCGATTCCTTTTAGCTCTTCCAATCAGTCTAATGTCTTTAATTATTCAAGTTTCCAAAGGTACCAATAAATTATGGCCAAGAAGTGGTTTATCAAACATTTTATCTATTTGCACGACGACGTAGGTGAACGGATACAAGAAGCTGGTAGGATACAAAAACTCTATCTAATGCCAATATGGATAAAACAACATACATATTTTAATCTATATAAGGTGGAGATGTGTGAGGAGGGACAAGGTTGGTAAAAAAAAAAGAGGAGGAATAAGGAAGGAAAAAAAGAAAAAAAAAAGAGGAATAAG

>cucumber_newGene_46 cucumber_newGene_46.1

CAAAAACACATTTTTTGCCCTTTTCTCCCTTCTTTTTTAAGCTTCTCCCCCTTCCTTCTTCGATTTTAATTCATTCAAACGGTTGAAGAAATGTTCGGGATGGCGGCAGAGGCAATGATCGTCATGACAGTGGTGATGGTGGTCGGGTGGTTGTTATCTGGAGTTGAGGGTAAACAGCCGGTGTTGCATAACGTTGGAGGAGGAAAGTATACTTGGACAACTAATATTAACTTCACTGATTGGTCCATTCATGACCACTTTTATGTTGGCGATTGGCTTTATTTTGGATTCGATAAACATATCTACAACGTGTTAGAAGTGAACAAAACGAGTTACGAGAATTGCAACGAGAAGGATTTCATTTTCAACATAACAAAGGGAGGAAGAGATGTGTTCAATCTAACAGAAGCAAAGACATATTATTTTCTAAGTGGAAGAGGATTTTGCTTTCAAGGAATGAAAGTTGCTGTTTTTGTTGAAGAATACTCTCCTCCTCCTTCCACTATAATTTCATCACCAAATTTACCCAAAGATAATTCCAATCTTTCTCCATCTTTAAAGCCATTATTAATACCTTTCTCCTTCTTCCTATTAATTGCTCAATATTTCCTCCATGAGACTAATCCTTTCTTTTCTAAATTAGGGTTTATCGATACATTTATTTATATTTTAAATAATTAATTAGGGCTAAAATTAGGGGTTAGCTTTGCTTGGGTTTGAATCCTTTAACTTCTCACTATGATTCTAGTTTAAGTTTTGTGTTAAAGGAATGATAAAATAAATTTGAAGTTAACTATAGTTAGTCATTAGGGTAGAGCTAGTAGCTGGTTGAAAGTTTCAACTTTGACTTGTTTGAAATTTGTAATT

>cucumber_newGene_48 cucumber_newGene_48.1

TTTTTCTCCACTAGAACGCGCACAAACTCGTGATAGCGGCGCGCCATAGCGCCGTATATGGAAGCTTGAAAGAAAGTTCCCAGAAAAAAAGTTCACGGAAATACTCATCGCCAAGGAGAGATTGAGCGCGAAGCAAAATCTGGAATATAGGGGCAAAGAGTAATTATGCATTACAAGAAACAACCCTGCAGAGATTTTCAGCGTGGCACTTGTCAGTATGGTGAAAGGTGCAAATTTCTCCACGCCACTCAGCAAGCACAACAACAAAAACCTAATCCCTTTGGATTTGGAGTTCCTAACAACGGTCAATCTAAAGCGGTTGCTGATTTTGGATCCAAACAAAACCAATATAAGCCATTTGAAAACAAGTGGACTCGTCCTTCCACCCCTACGGGAAATGCTCAGTCTCGAAAACCAGACAATCATCTCCCGTCCTCTAATCACAAATGCACAGATGGAGAGTCTTGCAAGCGTCAAATCGCTGAAGATTTTCAAAATGAGAGGCCATTATGGAAATTAACTTGTTACGGCCACAATAAAAATGAACCTTGTGACATTGTTGGTGATGTTAGTTATGAAGAACTACGAACAATTGCTTATGATGAAGCCAAACGAGGAATAAGTTTGCAATCAATTGTTGAAAGAGAGAGAAATTTACTTAATTCTAAGTTAGCCGAGTTTGAGGGCCTTCTTCTTAAGCCATATGTAACACCATCAAATCGTGCTCCTGGCAACCAAAGCTCATTTTCAGGAACTAATTCTCCTTCAATTCTACCAAGTGCTCAAAATAACACTCCTTCGTTGTCAAGTTTTAGCCAGTTGGGTGCATCACTTAATACAGGATTTGGTGCTAGGCCCTCTAATCCACATAATACTGTATTTGGTCAGCAAGTTCAATTTTCAAGCCCCGTGCAAAACTCGAGTGGATTTGGAATGACCAATTTTCCATCAACAAGTGTGGTTGCAGTTGGAGGTGGAGTTGGTAGCCAAACATTTGGGAACTTCTCTACTCTAAGTGGCTTTGACATAAAAAATGCTGGAAGTAATATTTTCTCTTCAGCAGCCCTAACAAATCTCCCTCCAACGAATGCAAACTCAAGTGCCAGTGGACAAATTGCACCAAATGCCCAATTGGTAAATAAGTTACAGCCAGAAAATAGTTCCGTGGATGTTGACATTTGGATGAAAGAGAAATGGGTTCCTGGAGAGATACCGGAAATGGCTCCTCCTGATGCAGTTATTCAGTAACACCGGCGGATTTGGTATAGAGCCCCCCTCAACTTTATTGTCTTTATATAATATGTATAAACAAAACCATTTAATGCTTAATATTTATAGATAGTTTAGTGGTAACTATGCTTAGTTTAGGGTTTTCTGGATCCGCCATT

>cucumber_newGene_49 cucumber_newGene_49.1

TATCAAGGGAGAACATACAAATCAAGGAGACTAAAGCACAAGGGATAATGTTACATGGGATGATGGAGATGGCCATAAAGGACCCTCAAGAAGCATTGCTGACCTTATCTAAAGGAATAGCCAACCGAGCAGTAGGAGAGACCCAAATGAACATCGCCAGTAGTAGGAGCCACTGTATTTACATGTTCACTATTCAGCAAGAATCAGCCAAGGACAAGAGGGCCAGAACTGGAAAATTGAATCTAGTGGACTTGGCAGGGTCTGAAAAAGTGGAAAAAACTGGTGCTGAGGGGAGAGTTCTTGAAGAAGCCAAGTCCATTAACAGATCGCTTTCAGCTCTCGGGAATGTAATAAATGCTCTAACATGTGGACCAACGGGGAGAGGAAACCACATCCCATATCGTGATTCTAAGTTGACACGGATTCTACAAGATGCACTTGGGAGGGAACTCACGTACTGCTTTATTGTGTTGCTGCTCACCCAGTCCGACAAACTCCTCTGAGATTCTCTCAACCCTTCGATTTGGTGCAAGGGCAAAGCATATAAAGGCTTCTCCTCTTGTCGCAATTGAGGATAAATGTATCAGAGAACTTGAAGTTTTCACTCCAATCAAAAGGGAATCACGTGACAAACTTCTAAATGAGGAGAGCTTGGATATTGAGCATGTTCAAAAACTTGAGGAGCTGTTCATACAAGAAGGAATTCTTTTTGATCCTTGCTCCATTGAAGAATCGGAATTGGCCTATGAAGATGTTACTTCACAAACTATTTCTTCCTTGCAGCTAGCTTTGGAAGAACTCTTGAGGACCATTGGAGAGCTTAAGAAAGAGAATGAGAAGCTGATGAGAAGATTATCAGCAGCTGAACTCAATCATCACCCCATCTCTAACTCCAGCTTACTGCTTACTATTTCTTCCATTTTTTGTCTCTTTACGGATTGCTTAAAATCTCTTTTCCATTTCCCAATCAACCATTATAATTAGTTTCAAATTCAAAACCAACTATTTTGTTTTTTTTTTACTCCAACAAAATAAATTAGTCTTGCAGAATTATAAATTTCGAGTTTGAACATTGTAGCAATTCCATTTCTTCTGGCTCTATTCTTTACATTACATTGGAGACACTAAGATATCAAAGTGATTCGAATATTTTTAGTGGACTTTCATCTTTTGTGTCCTTAATTCTACATTATTTTAAGGCTAAAAATTACAGTTTGTCCCTAAACATGTAGTGAGGATTTAGTGTTTTGAGTTTT

>cucumber_newGene_5 cucumber_newGene_5.1

TCTCAGAGAGGTGTGTTGTCTTAGACTAACCAATCACTAGGTTTGGTCGGTTATATATATACAATTGTATTAGTAACTATATTGTAAATAACAATAGATGAAGTTCATGACAATTAAATCGGTTTAGGGTTTTGGTTGGTTGTCAAGAATTTCAAATCATTATCAACAATTGTCGTTGTAACTTGTCAATTTAGTCGGTCTTGGTTGTTTGATCCCACGGTCAGCTCTAGACAAGATATACCAGAATAATCAAAAGTCTGCCTTCATTTCATTTACACAAACATCAGTTTGTTTTAATGAAAGGAATCTGATACCTCCCTTTGGCATGGCAACTTGGCACTTCACAAACTGTTACTTTCAACACAACAATGAAGGCTGAAAATTGTTATAACATGTCATCCATTAGAATCTTTCATTAAAGTTGGCCCCCCCCTTCCCTATAAACTGAAATGCAAGAATGAGCAAAATGAAGAAAATGACATTGCTTCTTCAAAATGCCACCATAAAAGGTTAAAACAGCGAACCGAACAAGCAACAGGATGCTCTACTTTTGGATATTTCAACATCTGAAAAAGTGGGAAAGTTTCTTCGCAGAAAGCATCAAAAACACAGAATTACAGTTCATAAGAATCTTTGCAGAGTAATTTCCAAATTCATAATCAATGGAAACTTATGAAAAAAAACCCAAATAGTTAAAGCAATGTATTTATTTGCCCCATCAAGACATAGCAATTCCCGTAGCCTTATTAGGAATTGTATTTCATCATATAAGACCAAATGGACAGGAAAAGACACTTGCATAAACACCTATCGAAACAGCTAGCTAAGAATGGTTCTACTTGTGTCTAATCTAATCCAGAACATATAAGCCAGTAAAATGAGAGTCAGAAAAGAGCAAGTTTGAATTGAAAAACAAGCAGGGAAATTTGTTAATCAGCAGAAGAATAAAAAACTGAGTGTCTGTTCAAGAACCAAGAGATGACATATATTGAACAAAATCAAGAAAAGCAAGCAAAGGGAAGGATAAACAAGCAAAAAATCAATATCATCCAGTTAATTTGAATACAGCATTCCGTTAAATCCCTATGATCCTATCCCATTGGATAATACCATCTGATTGGTGGAAAGATTTAGAGCTGCAAACAAACCAACCAAACAATCCAAGTTAAACCATGACTTGCCAACAATCCCTTTATTCTTATCCACATCCATATATCTGTAACTAGCTAGATTGAAGGGGAAAAAAAAAGAGTCAGTTTCGAAGAAAATCACACTACAAAACAAAATCTTCTTCAATTTTCTCTCAAAGTGAAATTCCCATCTTCCACAAAGAAGAAACAGGAAGAAAATTTTCGTTATCCCCTTGACCCTTATGTCTAATTAATCAAAAGATGCAGACAGAAACACAAAGGGGTCCCAGATGGTCAAAGAAACGAACTAAAATAGAGGATCACTTTTTTCCCAGTTATCCTAAAATTTAATCGAACATCAAAAAACAAAAACAAAAAACAAAAAAAAAAAAAAGAGGAAAAGAACTGAGAGCGAGAGAGAGCGCTGGATGCAGAGGTTTATCGACCGGTTCGTGTTTTTGTGATACGAATCAGTACAAATCCCAAGAAGGGGTCGATGAACCGCTGCCTCCACTGACTCCACCTCTCATTGAATCAAGAAAAACAAGGAAAAGAAAAGAGACCCATAAAAAAACAAGAACGAACAAAATCAAGAATTGTAAAAAGTTTGGGCAGATCTAAAAAAAAAAAAAGATGAAAGACAAAAACGAAAGGACGAAAGAGAGAGAACCCATTTGAATCAGCTTCGCCATTCCCATTGCCATCAATTTAAAGAAAGTGGAAAGGAAGATTGAGGAATCGCAGAAAGTGAGAGAGAGAGAGAGAGAGAGAGAGAGAGAG

>cucumber_newGene_51 cucumber_newGene_51.3

AAAGCCCGAAAAATAATGCAGAGAGCATGTAGTGGCAGTAAGGGAGCAGCTTTTTTCATTAAACCCATTTTGATTTTCATGTGGTTATGATTTGTCTGTCTAATGTGCGTGTATATACAAAGCAAACCTGTATCCTTTGCATATGAATTATGAAACAGTGAATCTTAACAGTTTTGAAGTTGGAGAAGATATTACTTCTTAAGATCCAGCATCGAAAGTCTTCAAAATTTCCCCGGAAAATCGTGGTGGGATTTCTTGATTCTTGAGAAAAAGAAGAAGAAGAAAAATTCTCTTGTTGGATTTGTTTTCAAGACTGTTCTTCCTTTTCTATTCTGTTTTGCCAATTCTTGCCTCATCAGTCATCGCCTCTCTTTCTTCCCTTTCTCTTCCTTTGAAGCTCCAAAAATGGCGACCAAACTCTGTAATATTTGATTTGTTTTTGGAGGGTTTATTTTGTTCTTTTTTGTTTTTAATTTTCTGCATTTCGACCTTCCAATTAAGCAAATCATTTTCCTCTCTAGTTTTTCAAAACGGTTATGGGTTTTTTGCGGAAGTGTCATCTAAAGTCCAAAAAATTTCAAATTCCAGCTTAACTTTTAGTGATGATTACTTCATTAAATGTACAGGAGAAGAAAACGTTCATAATTTCTGGATTCTGCATGCCTTGAAAACTTAAGTCCCTAATACAAATCGTACAACCCGTTTGGCATTTTGGCACTTTCTTAGTTTTGATTTCATTTCATTCTTCTCCTTTTGCTATATATTCTCCCTTTTCCCTTCATTATTTCTCACTCACACCAGTTCCTTCTTCTTCTTTGCTCTGTTTTTTTCGAAAAACAATGGGTTTGGTTTCATTGAGGAATTTCTTCGTCCTGTCTTTAGCTTTGAATGTGAGTTTGATTTTAAGAGCTATAAATCAGAATGAGAAGCTCCATAGCGGAGCTTCAATGGCGGTCGAGAAAGGGGCTAAAGTAGCTCAAATGAAATTTTTGTCGTTCCCATCTCTGTCATCACCATCAGCAGCTTCTCCTGAAACTCAAACTCCTCTTGGTAGAGAAAGAGTCGTTAATCTTGACCATGGTGATCCGACAATGTATGAGCAATATTGGAAGCCAATGGGGGACAAGACTACAATAGTGATCCCTGGATGGCAATCAATGAGCTATTTTTCTGATGTTACGAACCTTTGTTGGTTCATGGAGCCTGAATTTGCCAAGCAAATTGTGAGGATACACAAGGTGGTTGGTAACGCCGTGACGGAGGGGAGGCACATTGTCGTCGGGACGGGATCGTCTCAACTTCTCTTAGCTGCCCTCTATGCATTGTCCCCACAAGATTCTTCCAAACCAATCAGCGTGGTCTCTACAGCCCCGTACTACTCGTCTTACCCTTTAATGACCGATTGTGTTAAGTCGGGGATCCACAAATGGGCTGGTGATGCGAAGGTATTTGACAAGGATGAGCCTTACATTGAATTGGTCACCTCCCCCAACAATCCTGATGGCTTTGTTCGACATCCCATGGTGAACCGAACTGGTGGTATATTGGTCCACGACCTTGCTTATTATTGGCCACAATATACTCCAATATCCACCCCGGCTGACAACGACTTGTCGCTTTTCACTGTGTCAAAAAGTACTGGACATGCTGGGTTACGTATTGGGTGGGCTCTTGTGAAGGATGTTGAAGTTGCAAAGAGAATGATCAAATTCATTGAGCTTAATACAATTGGTGTGTCCAAAGACTCGCAGCTTCGAGCTGCCAAAGTTCTCGAAGTAGTATCTGACAGTTGTGAACAAGCCGGTGGTTCGGAGTATGCAGAATCCTTCTTCCATTTCAGCCACACCGTCATGACCGAGCGATGGAGGCTGCTAAGGGAGGCAGTGAAACGCAGTGGCATATTTAGTTTGCCTGACTTTTCTCCTGCACATTGCAACTTCTTTGACAACAACTTGGGAACTCAACCTGCTTTTGCATGGTTAAAATGTGATCAAGACAACGTTGAAGATTGCGAGAGCTTTCTCCGAGGCCACAAGATTCTCACCCGTGGCGGGAAGCATTTCGGCGTTGGCTCGAAATATGTTCGAATTAGCATGTTGGATCGTGAAGAAACCTACAATTTATTTGTTGAAAGATTGTCTCAGATCAGGTCATGACATCTGATATTGGTAAATGGCTAAGATGGCATTAGAGGTTGATGAAGCAATGGGGGGTTTTTGGGCTTGCAAATGTAAATTTCAAAATGAAAATGAGAGAAAGGGAAATATTTGTTATTGATTATGTTGCAAGATCTCAA

>cucumber_newGene_52 cucumber_newGene_52.2

AAAAGCCGAACGATTCATGAAATATGGGTTTATGAATGAATGTTAACATCGCTCTTTCTTTTCGGATTTAGTTCTCTCGGTTCTCAGCTATCTTTCCTCGCCATTGCCGGCAGAGAACAATTGCGCTTTTTGGTTGCAGTTCCTGACGAGAAGCTCAGTTTCCTAGGGTTTGTATTCCATGGCGTCGCACGCTCATCTCGAAGATGACGATGATTTTGGAGGCGATTTTAATGGAACACATAACAATAGGCAGTCAGGGAATAAGAGAGGTTTTGGCGATCTCGAAGACGATGAGGACGACTTCTTTGGCTCCAAAAAGGCTAACTCTAAAGTAGAAGAAACTGCTCCAGGTGTAGCGACAAGAATGATTCTGTCGCTTCGTGAGAGTCTTGAAACATGCGAGGATACACTTGCTACATGCCGGACAGAGCTTGAAACTGCTAAGTCTGAGATTCAGAAGTGGATTTCTTCATTTCAAAATGAGAATTTCATTCCTTCTGGAACTTCCCCTGAACCTAAATACGTGGTCAATTATCTTCAATCACTGAAGTCATCAGAGGAGTCTCTAAGAGACCAGCTAGAAAAGGCAAAGAAGAAAGAAGCTGCTTTTATTGTCACGCTTGCAAAACGAGAGCAGGAGATTGCAGAGTTGAAGGCTGCAGTCCGGGACTTGAAAGCACAGCTGAAGCCACCATCAATGCAGGCAAGGAGATTATTGTTAGATCCTGCAATTCATGAAGAGTTTACTCGTCTGAAGAATTTGGTTGAAGAGAAAGACAAAAAAGTGAAGGAGCTGCAAGATAACATTGCCGCAGTCAGTTTTACTCCATCTAGCAAAATGGGTAAAATGTTGATGGCTAAATGCAGGACTCTGCAAGAGGAAAATGAGGAGATAGGAAATCAAGCTGCTGAAGGAAAGATGCATGAGTTAGGAATGAAGTTGGCCTCGCAGAAAGCTCAAAATACAGAACTTAGAATTCAGTATGAAGCCTTGCAGAATCATATGGAAGGGCTGACAAATGACGTTGAGAGATCAAACGAATTGGTTATTGTCCTGCAAGAGAAACTAGCAGAGAAGGAACGCCAGATTGAAACTTTGAAGGAAACTTTACAACAGCCGAGGAGCCCATTAGTTGAGGATAAAGCTGTTTCCCCACCCACCGAGAAGCCCAAAGACGAGATGATTATATCCACCGATGCCGAAAGCTGAGATGATGATGATGATGATGAAACCATATCACCTCATACTAGTTTTCATAATTTTGCATCTAATCAACTATTTTCCTCCTGTAAATTGCACTCCATTTTAACTCGGCTC

>cucumber_newGene_53 cucumber_newGene_53.1

CTTCTCTCTCTCTCTGCTTCGGTTTCTCCTTTCATGTCTACGATAAAAACAATGTTATTCCATCATTGGTTTCTCCAATTTACACTCATAGTGACACTATTCCTAAACTTGGAAGCCATTCAAATAAACCACCAAACCCTAAAGACCCAATCTTTTCTCTCCCCATTATTCACTTTAACTCCTGGCTCAGTAATTGAAAAATTCTACTACAATCTCAACTTTCCCAAAGGCCATATTGCAATAAAGAGCTTTGATGCTGAAGTTGTCGATGAACAAGGTAACCCCGTCTCGCTTTTCGACACATATCTCCATCATTGGACACTCGTAAGATATTACCAACACAACAAAACCACAACAACAAATCATACAACAAATACAAACTCTATTATTATCGCGGGTAATAATGGAGTTTGCCAACCAAATACATTATCGTATTTTTATGGTATGGGAACCGAAGCGAGGAAAACATCAAATTTTCTTCCTGACCCATATGGAATTGAAGTTGGGAATGAAAAGGAAGTTCCTTTAGGGTTTGAAGAGAAGTGGGTTCTTAATGTTCATGCCATTGATACTAGAGGTGTGGTGGATAGAGTTGGATGTCTTGAGTGTAAATGTAGTTTGTATGATGTTAGCAAAGATGAATTGGATGATGATTATATAGGAGGATTTAAATGTTGTTATGATAAAGCTCAATGTAAAGTGAGGGAAGGTTATAATGGAGAGGAAAGGAATTTGTATATGAAATATACAGTGCAATGGGTTGATTGGGATGATGATTTTGTGATTCCTGTTAAAGTTTATGTAATTGATGTGACTGATACTTGGAAGCCGTCGATGGACTCATCGGGATTAATATCTCAAGAACATGACTGTAAGGTTGAGTATGATGTAGAATCTTGCTCCCTCAAAAACAAGTTGCATGGGAAGTGTAATGCTAACAAAAGGTCAAAGGTGATGTTTCAAGACACTGGATTCATTGTGTATGGAGCGGCTCATCAACACATTGGTGCAACCGGTGCAACCCTCTACGGAGAGGATGGAAGAGTTTTATGTTCCTCGTCACCAATATATGGAGAAGGAGACGAAATAGGAAATGAAGAAGGATATATTGTTGGAATGTCAACTTGTTATCCAAAATTAGGGGATGTGAAGATTAATAAAGGAGAAATAGGGAGTTTTGTATCAAAGTATGATCCTACACAAAACCACACAGGTGTTATGGGTATCTTTAGTATTGTGGTTGCTACCAAATTACCTAACTCATTGTCTCACATGGAAGTATGAGCAATAAATCTTGGTTAGTCACATCCTAAAGAAATTAAATAATTCATGTGAAACTTATTATTGTATTCACATATATGATATTTTGTGTAAAATTTGGC

>cucumber_newGene_54 cucumber_newGene_54.1

GTCTCATCCTCCTCTATAAAAAGCTGCACCAACTGCAATGCCCATTGTCTTACCATTGCACTCAAAATCGAGCCAATTACGCCACTAATACTTCTTTTTTTTTTTTCTCTCTTCGGCTAAAATGGCTACTCCCACTCCGGCTCCGGCTCTTAACGACGAAGAGCGATTGATTCAAAAGAAATACTGGATTGAACATTCAGCCAGATTAACGGTAGAGGCTATGCTGCTGGATTCGAACGCCTCTGATCTCGACAAAGAAGAACGACCTGAAGTGCTTTCTCTGCTTCCGCCTTACGAAGGAAAATCGGTACTGGAACTTGGAGCCGGCATTGGTCGTTTCACCGGTGATTTAGCGGTGAAGGCTGGGCAGGTTCTGGCATTGGACTTCATCGAGAGTGTGATCAAGAAGAATGAAAGTATTAATCGGCACCACAAAAACGTAAAGTTCATGTGTGCGGATGTAACTTCTCCGGAGCTTAAAATTTCGGAAAATTCGGTGGATTTGATCTTCTCGAATTGGCTGCTCATGTATTTATCTGATATCGAGGTGAAGAATCTGGCAGAAAGAATGGTGAAATGGCTAAAAGTTGGGGGCCATATTTTCTTTAGAGAATCTTGTTTCCATCAATCTGGGGATTGTAAACGAAAATACAACCCCACCCACTACCGAGAGCCACGTTTCTACACTAAGGTGTTCAAGGAGTGTCATATGCAAGATGAAAGTGGAGATAGCTATGAGCTATCTCTTGTTGGATACAAATGTATTGGAGCTTATGCAAGGAACAAGAAGAATCAGAATCAGATCTGTTGGATATGGCAGAAGAAGAAATGTTCAAACGACGATAGAGGATTCCAACGCTTCTTGGATACTGTTCAATACAAATGCAGTGGCATTCTTCGATATGAACGTGTCTTTGGATCAGGCTTTGTCAGCACTGGCGGACTCGAGACAACAAAGGAATTTGTAGCAAAACTAGATCTAAAAGCAGGACAAAGGGTTTTGGATGTGGGTTGTGGAATTGGAGGAGGAGATTTTTACATGGCGGAGAATTTCGATGTCGAAGTTGTCGGAATAGACCTATCCATCAATATGATCTCTTTAGCTTTAGAACGAGCTATTGGACTCAAATGTGCAGTTGAATTCGAAGTGGCTGATTGCACAAAGAAAGTGTATCTAGATAACACATTTGATGTCATTTACAGCCGTGACACTATTTTACATATTCAAGACAAACCTGCTTTGTTTAGATCATTTTATAAATGGGTAAAACCCGGAGGAAAGATTTTAATAAGTGATTATTGCAAAAGTGGTGGAGTTCCTTCCTCTGAATTTGCTGAGTATATTGAACAAAGAGGGTATGATTTGCATGACATCAAATCCTACGCCAAGATGCTCGAGGAAGCTGGTTTCAACAATGTGGTAGCCGAGGATCGAACTGATCAGTTCATGAGTGTTCTTCAAAGGGAACTTGACATTGTTGAAAAGGAAAAGGAAGCATTCATCCTTGACTTTTCTGAGGAAGATTACAATGATATCGTTGGAGGTTGGAAGGCAAAGTTGAAAAGAAGTTGGTGTGGAGAGCAGAAATGGGGATTGTTCATTGCCCATAAAACTACTAAATGATCTCCTCATCAATAAAAACTACTATTGTAATTAATTATTTTCTTGTTCAACTACTATGTTTTTCTTTTCTTCAATAATTTGGCAAGAATCGTACTATTGTTCACGTTTTCAAAGATGGCAATAAATTGCCTTACTTTACTCTTCGATTAAAGTTATGTTTAG

>cucumber_newGene_56 cucumber_newGene_56.4

TACACAAAGACTGTGGAAGAGTAAGGGAAGGATCCTTTGTTGGGTGGAGACAATAAAATAAGATGGCGAAATGTTCAAAGTCTTCGCAGTCGCTGCGTTGCCTTGGTCACCTCTTTTACTATTACAACCTCCTCTTCATTCTCACAATGAAGTTGCAGTTGCCGACTTCAACTCTTCACACTTTCACTTTCTCCCTCTCTCTTCTTTCTCTATGGAATTCGCTTTGGAGTCCAGTCCTAAGTAGCCTTCTCCATCTCCATCTCTCTCACTCTCAGGTCTAGATTCTTAGGCATGGCTGCTTTTGAAAAAGACGATATACCAATGCTATCAAACACTGATCCACCAATGTTTGACGAACAAGTGGATTCTTATTTTCCTTCATATGCATCCTTGGGCCGGAGTTCGTCACTATCAATTCCAGAAAATTCTTCTGGGATGTATGGTAGCGAAGCAAATCTTGTTGGTCATACTGGTCCCCTACGGATAGAAAGAAGTTCATTCATGGTGAGTGGTTCAAAAAAAACTGGCAGTAAGCCCAAAAAACTTTCACAGTCAAAGCCAGGAGTGACTGAAAGCAAGACAGCAGAGCCGCTGGCAGAAAAATTTCCTTCGTTCAAGATTGTAGATGAGTCTGATTGGTCCATTCATAACTATGCCGGTAGGAACGAACACTTAATAAAGTCTGGCCAGCTGGGATTGTGTAATGATCCTTTCTGTATAACTTGCCCAACATATAATTCCAAAGCATCACAACAGAAAAGCTCAAGAATGTCAGGCATATTTGATGCCAAGGAAAAAAAAACTCTGCCTACTTCCTTGCCAGTGAATGGATGTTCAAGTGGTCAGAAATGACAATAGTTGGCTTTCTACCATGACCAGTTTCATAATGATCTCTATGGAGGAGGGAAAGGATGGGCGAGGAAGTTTCAGTCCTTCTGGTGCTTCTTGCTTTCATATATTCCTGGTGTAATGAATCCTCATGCAAAAGTTGTTCAGCAATGGAATAAGTTTTTTGTCATATCTTGCTTGGTGGCAATCTTCTTGGACCCATTGTTTTTCTTCTTGCTGTCTGTTCAAGAGAATAATAAGTGCATTGTTATTGACTGGCCTTTGACCACAACCTTGGTGGTTTTCCGGAGCATGACTGATTTTATCTATTTCCTACACATGCTTCTTCAGTTTAGGTTGGCCTATGTAGCACCAGAATCCAGAGTCGTTGGTGCTGGCGAATTGGTTGACCATCCGAAGAAAATTGCCATGAATTACCTCAAAGGAAATTTTTTTATAGATTTGCTTGTTGTCTTGCCGCTGCCTCAGATCATAATTTTTTTAATCCTGCCAAAGTCTTTGGGATCATCTGGAGCAAATTATGCCAAAAATCTATTACGCACTGCAGTTACTGTTCAATATATTCCCAGACTGTATAGGTTTCTACCTCTTCTCGCTGGCCAGTCTCCAAGTGGCTTTGTATTTGAGACAGCATGGGCAAATTTTGTCATCAATCTTTTGACCTTTATGTTGGCTGGTCATATTGTTGGGTCTTTCTGGTATCTTCTCGGGCTTCAGAGGGTGACTCGATGTTTTCGAGAAGCTTGCATTAACACTGGAAATAGGAGCTGCTTGGAATATATAGATTGTGGGCATGGGGATGAATCTAGGAGTCAGATTCTGGATCCACATCGGGATTCATGGACGCATAATGTGAATGCTACGGCATGTTTCGGCAGTGAAGGTTTCGATTATGGAATCTACCTTCAAGCTGTAAAACTTACCACAAAAAATAGTATAATCACGAGATATACATACTCATTATTTTGGGGATTCCAGCAAATCAGTACTCTGGCGGGGAACCAAGTTCCAAGCTATTATGTCTATGAAGTCCTTTTCACTATGGGTATTATTGGGCTTGGTCTATTGCTGTTTGCTCTTCTCATCGGGAATATGCAGAACTTTCTCCAGGCTCTTGGAAGAAGGAGGTTAGAAATGTCACTACGACGTCGTGATGTCGAGCAATGGATGGAGCATCGTCGCTTACCAGAAGGTTTAAGAAGGCAAGTACGACAGGCTGAAAGATACAACTGGGCTTCTACAAGAGGAGTAAATGAAGAGAGAATTTTTGAAAATTTGCCTGAGGACCTACAAAAAAACATAAGACGGCATCTCTTTAAATTTGTCAACAAGGTTCGCATTTTTGCTCTGATGGACTATGAACCTATTTTAGACGCAATACGTGAGAGACTGCGACAAAAGACATACATAGAAGGAAGTGAAGTTTTTTCTGCTGGAGATATCATTGAGAAGATGGTTTTCATCGTACGGGGGAAAATGGAGAGCCGTGTAGATGGAAATGGGATTGTGGTTCCCTTATCTGAAGGAGATGTCTGCGGTGAGGAACTTCTAACATGGTGTCTTGAGCATTCCTCCCTAAACAAAGATATGAAGCGGCAGCAAGTTCCAGCGCCGAGATTAGTAAGCAACCGGACCGTAAGATGCTTAAGTAATGTTGAAGCATTTTCTCTCCGGGCTGTAGACCTTGAAGAAGTGACTAGTATGTTTTCTAGATTCTTGCGAAACCCGCGTGTTCAAGGAGCCATAAGGTACGAATCACCCTACTGGAGGTATTTAGCTGCAATGCGTATTCAAGTAGCATGGAGATACAGGAAAAGGCGTCTATCTCGAGTTCAAACCTCTCAATCTAACTAATTCCTTAAGGGACACCACTTTTGTGGAAAGATAAGTATTGCGTTATACATCGGTCAGCTTGAGTTCCAATCGGCTTTTTCAATGTGATCATTTCATTCTTCTTTTTTATTTAATCCATTAATCCTTTTTTTTAAAGCAACTAAAAAGTCTATCATATGGTAAAGTTAATAGGAGCTCCTGCACACTGGTGCTAAGACAGTAGTTGTATTTTTAGTCTAGTGTCATGCCAAAAGTATATTTTTTTCTTTAGTTTATCGAAACTCAAAACAAGCGAAAGTGGTAGATTAGGTACTTTTGCCTCAATTAGTTGTCCTTAGTAGGATGTAATTTGTAAAACCAAACAAGTGAGTGATTTGTTGTTGAAATTTTATTACAAAAAATGTTTATAATTAATGGGATAGTTTCTTTATTCACCCTCTCGA

>cucumber_newGene_57 cucumber_newGene_57.1

AAATAATCTCAAGAGAGTCCCCAACGAAGAGAAAGAAAGCAAAAGGGTTTTAGAGACACTCTTCTTCTGGGTTTTACCGCTTCTTCCCCCACAACGCCGAGATGATTACTGATAAGGGCAAGAAGGCCAAGCTCTCTGAAAAGCCCGAAGACGACAATTCTGAGGTCATCGATGAGAAACTCGTTATGTCGATCGAGAAGTTGCAGGAAATTCAAGACGAGCTCGAGAAGATCAACGAGGAGGCCAGTGATAAAGTATTGGAAGTGGAACAGAAGTACAATGAAGTACGAAAACCTGTCTATGACAAGCGGAATGAGATCATCAAATCAATTCCTGACTTCTGGTTAACAGCGTTCTTGAGCCATCCTGCTCTTTGTGAACTTTTGACCGAAGAGGACCAGAAGATATTCAAACATCTCACCTCCCTGGAAGTGGAAGATTTTAAAGATGTCAAATCGGGTTACTCAATTACTTTTAACTTCAACACCAACCCTTATTTTGAAGATACAAAGCTGACGAAGACCTTCACCTTTCTTGATGAAGGAACAAGGAAAATCAATGCTACATCGATAAAATGGAAAGAAGGAATGGGTTTACCTAATGGGTTTAACCACGAGAAGAAAGGGAACAAACGACCTCAGGCCGAGGAAAGCTTTTTTAGCTGGTTCAGCGATACAGAACAGAAAGATATTGATGACTTCCATGATGAGGTTGCGGAAATTATCAAGGAGGATTTATGGCCTAACCCTTTATCCTACTTCAATAATGAGGCTGATGAAGATGAATTGGATGAAGACGAATCTAATGAAGAGGGAAAAGAAGATGACGACTCAGATGATGATGATGACGATCAAGATGATGACAATGATGCTGGTGATGATGAGGATGATGACTAAGATGACTAGTGAAGTTTAATGCATCTCTTCAAATGGCTCATTTCTGTAATAGTTGCTCAAGTATCAGTTGGTAAAGGAGAATCGTTTCTTGTGGTAGCCTTGCAGTCGTGGTAGGGTAACGGTTTGTAAGTTCAGTTGGTCAGGCATTTCGACTCGACTCTACTTTCTCCCTAGGAGCCCTTTCCTTTTCTCCTTCCTCGTTGCGTAAATTACTCTCTAATATATAGGTTGTTGAGGATATTTATGGTTTTTTAATCTTATGTTTTGTTCTAATGCTAACATTGGAGAAAGTATTTTGATGTGTAAAGTTATTTTGGACTCCCCCTTAATGCATATGTTATTAATTGTGTTACTAATTACCATTGCCATCCCTCTCAAAGCTTCACTGCTAATGGTGACCTTTGCTTCTGTTCTGCCATGGCTGGTGTTCCTGAACCAAAGCCAGAGCGAGTACTTTGTACATCTTGAGCGTTCGTCGGACACAATCTGCTTCCAAGTTTTAGTTTAGTTCTTGCTTCTTTTTTTTGTCATATCAAACATTTAATGCAAA

>cucumber_newGene_58 cucumber_newGene_58.1

CAACAACATCCCCTCAGCCCTCAACAACTTCTTCTTCGAGACTCAAAGCTCCGAAACACGGATTTTGTATACGGGGTGGTGATTTTCACTGGTCACGATACAAAGGTTATTCAGAACTCAACAGATCCTCCTTCCAAGAGAAGCAAGATTGAGAAAAGGATGGATAAGATTGTGTTCTTTCTGTTTGCTGTCTTGGTTTTGTTATCAGTTGTTGGGTCAATTTTCTTTGGTGTTAAGACTAGAGACGATTTAGAAAATGGAAGAGCTACGAGATGGTACCTACGGCCAGATGATACCACAATATATTACGACCCCAAAAATGCTCCAGCTGCAGCAGTATTGCAGTTTTTGACTGCTCTTATGCTTTTTAGCTATTTGATTCCCATATCATTGTATGTGTCCATTGAAATTGTCAAAGTTCTGCAGAGTGTCTTCATCAACCAAGATCTACATATGTACCATGAGGAAACTGATAAGCCAGCTCATGCCCGTACGTCAAATTTGAATGAAGAGCTTGGCCAAGTTGACACTATTCTTTCCGATAAAACGGGTACATTGACTTGCAATTCAATGGAGTTCATCAAGTGTTCGGTGGGCGGTACTGCGTACGGGCGAGGAATTACAGAAGTAGAGAGAGCTCTTGCGAGAAGAAAGGAGTCGACTTTACCTCAAAACTTTGGGGCCGACAATGCACGTCTTAGCGGCGAAAAAACATTCGTTAAAGGGTTCAATTTCAAGGACGAAAGAATGATGGATGGTAATTGGGTGAAGGAGCCTCGAGCCAATGTAATCCAGAAGTTCCTACAGCTTTTGGCTATTTGCCATACTGCATTGCCCGAAATTGATGAGGAAACTGGAAAAATATCTTATGAAGCTGAATCACCAGATGAGGCAGCTTTTGTGATTGCAGCCAGAGAATTCGGTTTCGAGTTCTATGAAAGGAGTCAGACAAGCATTTCATTACGGGAGTTCGATCCGACCTCAGCTAAAAAAGTTGAAAGGTCAGTTCACCGTGAGTTTATAGAATAATTTTCCGTCATCTTTATGATTGTTCTCCCAATTTCCCAATCGTCTATTTACTTTGAGTTTTATGACTATGGGTTTGCAGATCATATCAGCTACTGGATGTTTTGGAGTTTAATAGCACAAGAAAACGGATGTCTGTGATTATAAGAGATTCAAAGGGAAAACTACTACTTCTTTGTAAAGGAGCTGACAGTGTTATGTTTGAAAGACTTGCAAAGAATGGAAATGAGTTTGAAGAACAAACAAAGGTGCACATCAATGAGTATGCTGATGCTGGTTTAAGAACTTTGGTTCTAGCATATCGTGAGCTGAAAGAGGAGGAGTTTAATGCATTTCATCAAGAGTTCATCAAAGCGAAAAACACGGTGAGCACAGGTCGTGATGACATAATCGATCAGTTGACAGAAAGCATTGAGAAAGATTTGATTCTTCTTGGTGCTACAGCAGTTGAAGATAAGCTTCAAAATGGGGTCCCTGAATGCATAGACAAACTTGCTCAGGCCGGAATTAAAATATGGGTTCTGACTGGTGATAAGATGGAAACAGCCATCAACATTGGCTTCGCCTGCAGTTTACTTAGACAAGGAATGAAGCAAATAATTATCAGTTCAGAGACTCCAGAAGGAAAAGCCTTAGACAAAGTCGAAGATGTTCACAAATCTGCAGCTATCAAGGCGTTTAAAACAAGTGTGATTCAACAAATAACTGATGCGAAAGCATTACTTACATCCTCAAGCGAAAC

>cucumber_newGene_59 cucumber_newGene_59.1

TTTTTATAAAATTTAATTTTGTAATCTTGACAGGAAATTTCTAACTGGGTTAGAGAGTCAGATCATAAATTTTTCTACTCACTAGTCAATACTACTCAATCTGTAGTGAACTTTTTTTAACTTTTAAAGGAAACCTTCATTTATTAGATTGCTTTCAGTTTCTTCAAGTCTTCAACCACATTTCTTGTTGTCTTTGAAAAAAAAAGAAGAAGAACAGAGCGAAACAAAGACCAATATGCTCAACCCAGATTTACTATTGTGGTTTCATCTGCAAAATCTTTATCCTTTTGAGCTAAGATAAAAAAATTTGACTTGAAGAAGAAGGCAAAAAAGGCTTTGTCAACGCTTCTGAAAAGTCTTTGGTTTGGCCGTTGACGTGAGGTTCCAGCTCTTTCGTTTTTGTTTTAGCAGCAGCCAGAGGGAAAAGAAAAATGCAACGGAAGGGTTTGAACTAGAAGGTATAGTTGGATTTATAAATGGGGAGTGGTAGAAAAAGGAGGAAGCAACATTTCAGAAGGATTCACGCTTTTCCTTGTGGTAGAGCTTCATTCAAAGATGAACACTCTTTAATTGGAGGGCCCGGATTCTCAAGGGTAGTTTACTGTAACGATCCAGATAGTTTTGAGGCTAATCTACTTAACTATGGAGGCAATTATGTCAAAACTTCCAAGTATACCGTTGCTTCATTCTTCCCCAAATCATTGTTTGAGCAATTCAGAAGGGTTGCCAACTTGTATTTCCTTCTTTGTGCTTTGCTAT

>cucumber_newGene_6 cucumber_newGene_6.3

CACCTATTAACATAAGCAAGGCAGCTACACATCCAACCTTCATTAATCTTCTCAAACATGTAAGTACTCTTTAATTTCCATATTTACTATTAAGCTTCCCAATCTTATTTAAATTCCCTCTAATTTCTTATGATTATTCTTTTTTTTTTTCTTCCTGTTATCACAATTACTGAATTATTTAAATTTAATCCACCTTAACGATCGAGTACTTTGATATTAACTTGGCCTCACCGTATCTAGACCTCTCTCCTTTAGTTTGTAAGCAAAGAAGCACAGTCAGATGCATCATAGATATGGTACCAATACCCCCAGATACTTCCTAGATGGTATTTGATATTCGACCTGCCTTTTTTTTTTTCTCTCTCAATACATGTGTCTCCTGGGATTTGTTGGACCTTTTAAGAGAAAGAAAGAAGAAAGAAAACTCATGTAATCTTCTCCAACCCAAAACTAAGTAGCCTATTGAAAAAGTCCACTGCACCAACCAAAAACTAAAGGAGAGAAAAGAAAATCAAACTCTAGAATCATTTAATCTACATCTTAAGTTTTGAGTCTTCACAGAGTCACGTTCAAAAGAAAAAGGAAAAAGGAACCCACTTGAATATGTATCTGTCTCCCAATTCTTTTAAAATTGGTGTGTTGTTGTATCATATTTATACTTCATAACTGATAGAAGTTTGGTTTCGTAAGGAACTTTGGTCTTTGCATCATGAACCCGATCATAGTATAGAGAAGAAAACGAAGTTTTGGGTTTGGAGTCAAAATACAAATAAACCAAGGAAAATTTTTGAAGTACAAATAATATTTTGTGTGGGTTTGAGGAATAATAATTAAGAATATAACAATATTTAAAAAAAATTGTAAATATAGCAAAACTATCGCTATAAACTTGTATCGTTGATAGAATTTGACAAATTTTACTATATTTATAAATCTTTTAAAATGATGTTATATACTTAATTATTTTGAATCTAATTGCTAAATTTGTTGCTATCCATTTGTTTTCCTAAATTTTCTCTCATCTATATAATATCAAAAATTACAAAGTCAAAATGAAAAAAAACCATCAAAATATATATACTAAGTGTATTAAGAGAGGAGGAGGAGAAGAAGTGTGGAAATCATAGTAATGATAATAGTAAGAAGAAATGTTGATGATAATTATAATAGTAATAATATATATTTAAAAATAATAATTTGTATGGAATAGAGTCGAGTCTACGTAATGTTGACCCATTTTACTAAAATAGTTTTGAAAGTATCCATTTTTCTCAAATTTCTTTAAAACCAACCCTTTTCAGATCTCACAATATTTGAAAGAACAGTAACTTACAATAATTTATACAATATATTTTGAACATAAAGAAAGATCACAAACAGATTCTACATCTTCCTCCTTTGACTTAATGTATGACTATGGCAACCACAGTATATAACTATCTTATTCTTGGGATTATTTGTTATTAAGAACTACTGTTTGCAGGTGAAAAATTCCTAACAAATATATTTTTAGACATTCCTCACTCTAATTTGCTGCAGAGTAAAATAATGTTGCTTCCTTTGGTACTTGTAATGATGACAATGATCTCATGCTTGGAAGCTGTTGGAACAAACAATAACAACAATCTCATGAATATGGTCATAAAAACTCAAACCTTTTTATCTCCATCATTCACCATGACACCTGGTTTAGTAATCGAGAAATTCTTCTACAATATCAACTTCCCAAAATCCCACATTGCCATAAAAAGCTTTCATGTTGAAGTTGTAGATGAATCAGGCAATCAAATCCCACTTTCACAAACTTACCTTCATCACTGGGTACTTGTGAGATATTACCAACACAAAAATGCAACAAACCCAACAATAAATACTAGCTATAACGAGCTTCAAGAACCAAACTTTATCATTGCTAGCAACAGTGGAGTCTGTGAACGGAACATTTTACCGGCCTACTACGCCATGGGATCCGAATCAAGAAAACTATCCACATTTCTTCCACACCCATATGGAATTGAAGTTGGGAATCCAAAAGAAATTCCCACAGATTATGAAGAGAGGTGGAGTCTCAATGTTCATGCCATTGATACAAGAGGAGCAGAGAACAAGTTGGGATGTATCGAGTGTCATTGCCATTTGTATAATATTACAAAAGATCGATCTGGAAGGCCATTAACGGAAGATTATAAAGGAGGTTTGAGATGTTGTTATGATAAAACAAAGTGTAGAGTGAATGCATTAAGTGATGGAGAAGATTTTCCAGAAAGGAATTTGTTTGTGAGATATAGAGTGAAGTGGGTGGATTGGAATGATTTTGTGATTCCTGTCAAAATTTATTTATTTGATGTTACTGATACTCCGAAGCCATTGTCAGACTCAACGGAAGCTTCTCAACAACATCATTGTCTGATTGAGTATGATGTAGAAGCAGAGTTTTGCTCCCTCAAAAATAAGCTTGATGATGATAAATGTAATGCTGTGAAAAAGTCGAAGGTAATGTTTCCATCAAGTGGGTATCTCATCTATGGAGTGGCTCACCAACACATTGGTGCAACTGGTGCAACATTTTACGGACAGGATGGAAGAGTTTTATGCTCTTCATCTCCAATTCATGGGAAAGAAAATGAGGAAGGATATGTGATTGGAATGACAACTTGTTATCCAAAGCCAGGTTCAATCAAGATCAACAAAGGAGAAATGGTAACTTTTGTATCCAATTATAGTTCCACACTGACCCACAGAGGAGTTATGGGTATCTTTCACATTATTGTTGCTGACAGAATATTCAAACCATCATCGACACTTTCGGAAGAAGTTGGCAATAACAACACCATCGTCATGTTATGATCTAAAAGCTTAGTTTGTACTGTTGTAGCTTTTTGAATAAGTGATCTATAGTATGAAGTAAATTAGTATTTGGTAAATCTACAGTTTTTAGATATTAAAAAGT

>cucumber_newGene_60 cucumber_newGene_60.1

ATTTGATCGAACTTTTATAGTTGCTGAAAATGGTTTTTCAGATCGATTTTGTTCACCAGATAAAAATATTTCGTCGAGTTCCATGTATTTGACAATTTCTTTATATTCTGACTGTGAGTCTGTACCACCATACACCCCTGCTTGTTCTGAAGCTGCATATAGCTACATCCCAGAAAAAAGTAACGTAGTTCTGATGAATGATATGTGTTGAATAAGTGTAGTAGCGCCAATTCCACAATGGTTGCTAAAACAAGTTGGTGATGATTTTCAAAAGGTAGCGCAACATATCACCGCTCAGTTTACGTCAGGGCGGACGGTAAATGTTCAAGTATGTAACAATTGCAAAGATTCTGGTGGTGGTGGTGTTTATGTTTATGACTTGGAATTGAATCAATCAAGATGCTGCTGTCAATCTTCCTTTGATAGGATTGAATTCTGTTCTTCCTCACTACTTGCAGCAACACCACGACCTTCTCCATCCAGATCGGGAATGTCAAATACGTCAACAGAAGCACCTTCTCCAGACAAGGATGAAAATATCGCTCCGTATGCAAATATCAGTAAGTCATTTTTACAGAATTTAGAAAAAAAGTTATCAGGAATATAACCAAAATTCATAAATGTTGTGAAAATAACATAATTACTATTGTTGCGTACATATTGACCATGTTCGACTCTTCAAATTCATCCCCTCCATACTTGAAACAAGTGAGTTTAACATCGTTCAAACCATCATCAAGTGTGTATCTTTTATGAAGGCAAGGTTTTTCTATAGCTCCACTAGCTTTACACCTATGGTTAGAGTCTCGTGTGACATGCATGAATTGAGATTTTCCAATGAGAGGGTTAATTCCTCATCCACCAATTGTATGGCCATTGACGAAGCCTCTTTCTTT

>cucumber_newGene_62 cucumber_newGene_62.6

TATATTACCCTTTAATTTAAAAAACCCTAAAAATTAACTTTGTGTCATTTTCTCCTTTTCCTTCTCCCTCACGTTCTCTTCTTGCGGCTTTCTCCTTCTCCCTCACTTTCTCTTCTACTAAGACAAAGGGCAGCCCGACTTCAACTCTTCTCCTTTCTCAGTTGCTGTCGCATCTCATTTACCGTTGTCAACCGTTCAGGCATCCATTGGTGGTCAGTTTGTACGCGTCGTGCAAGATTCTCCATCGAAGATGCGGCATGCAAGATTCTCCTTTCTCAGTCTTTTTTTTATAACCACGCACTTTCTGTTTAGGGATTGTTTTGATCGGATTGCTTTTGTTGTTGTGCATTTTCATTGAATGGTTGAAGGCACGTATCTCGTACACATCTCGCACCTATCTTGCACATTTCAAAACTTTTACTATTATTTTCAAAATCAAATTGTTACCCCTCCTCGTCGGAATCCAATTTTCGCAGCAAGATCACGAGTACCAAGTCATGTTCCACAATGAAATCACCGGAACTACCATTGTTTTTGTAGTTTTGTGAATGAAAAATGAGAAAGGAAGAAAAAGGAAATCAAAGGTTTTGTGAAAATGAGGAAAAATGGAATACGGATAGAGAGAGATAGAAAAATGAAATATTCTTTTTGGAAGTGAGAAAATGGAGGAAGAAGGAGAAGGAATTCCATAAATTGTTTTTTAGGTTTTTTTTATAACTAAAGGGTAATATAGACTTTTCCTAGTTAATTTGTCCTAAATGTCATCTTTTTTAAAATCCATCCTAAAACTCTTTAAACCCTTAAAAATTATCCTATTTTTGTTAATTTCCCAAATCTAATATGCATTCTGTGAGTGCGAGCCAAACAGTGACACGACTTCTCGTGGCGCGACTTGTGTGGAGCTAAACTAAGCTGCTCGCCGGTCGTCGGATCTCCGGTACCCTTCACTGCTGATCGTCGTCCCAACCTTTACCTTCACCAATACTTCCATTTCTGTTTCACATCCCACAGTCGATTCATCGAATTCACTGCAGATCACCGGTCGGCCGTCGGTTGATCAGAAGCCGAATTCATTCTACTTCTTCCGTTCTTTCTAAACCCACCGTGGTCACAAGCTTCCCTGGTGTACGACGAGTGGTTGGTCAAGTAAAGTTGAGGAGTTGTGGGTTAGGCAGCTGGGTGGTGAAGGTTCGGTGGTATTAATGGCTAGTCCGGGGAAAAACCTAAAGCCTGAAATTTGTGGTTTAAAGGGATTGAATCAGGAACCTGATGATAAAAACCAGCTTGTTGACAAAGAAGCTGGCCTGAGTGGTGGTGAGAGGGATATGGCTCCGGTTACCGGGAATTCCATTCACAGGTCAGGTTCTCGTCCACAGCTAGACCTTAGCAAAGCAGAAATCCAGGGAAATTTTGAGGAGAGAGATCCCACGATTCTGTTGCCTAACCAATCTGATGATATATCTCACCTGGCTCTTGACATCGGCGGATCTCTCATCAAATTGGTGTACTTTTCTAGACATGAAGATCAATCGGTTGATGATAAGAGAAAGAGAAGCGTTAAGCAGAGACTGGGATTATCAAATGGTAATAGAAGAAGTTATCCAGTTCTTGGCGGGAGGCTTCATTTTGTGAAGTTTGAGACAAGCAAGATTAATGAGTGTTTAGACTTCATCCATTCCAAGCAGCTACATCGTGGTGCTTACAAAGATTTACCTCATTGGCATTCAGATGGCCCAGAAAATGAAAATGCCATCATTAAGGCTACAGGTGGAGGGGCATACAAGTTTGCAGATCTTTTTAAAGAAAGACTTGGAGTTAGCATTGATAAAGAAGATGAGATGGATTGTCTTGTGGCTGGAGCAAATTTTCTGCTAAAGGCAATACGCCATGAAGCTTTCACTCATATGGAGGGTAAAAAAGAGTTTGTGCAACTTGACCACAATGAATTGTTTCCTTATCTGCTTGTTAACATTGGATCTGGCGTCAGCATGATCAAGGTTGATGGTGATGGGAAATTTCAGAGAGTTAGCGGGACAAATGTTGGAGGGGGCACTTACTGGGGCTTAGGAAGACTATTAACAAAATGTAATAGTTTTGATGAGTTGCTAGAGCTTAGTCAAAAGGGAGATAATAGCACAATTGACATGCTTGTTGGGGACATTTATGGTGGCATGGATTACTCTAAGATCGGTCTCTCTGCTTCAACAATTGCATCAAGTTTTGGCAAGACCATCTCAGTGAACAAGGAGCTTGAGGACTACAGACCAGAAGATATCTCATTGTCTCTCTTGCGAATGATCTCATATAACATTGGACAGATTTCGTACTTGAATGCTCTCCGGTTTGGGCTTAAGAGAATATTTTTTGGAGGATTCTTTATCAGGGGACATGCCTATACCATGGATACAATTTCCTTCGCAGTTCACTTCTGGTCAAAAGGAGAAGCACAAGCAATGTTCTTGCGTCATGAGGGGTTCTTAGGAGCCTTAGGTGCATTCATGAGCTATGAAAAGCATGGTCTTGATGATCTATTGGCCCATCAGTTAGTTGAAAGGTTTCCCATGGGTGCACCATACACGGGAGGGAAAGTTCATGGGCCTCCACTTGGTGATTTGAATGAGAAAATTTCTTGGATGGAGAAATTCTTGCAGAAGAGAACAGAAATTACTGCACCTGTCCCTACGGCTCCCCCTGGAACTACTGGACTTGGAGGCTTTGAAGTCCCTTTATCCAAAGGAGAAACCTTGCGATCTGATGCAAGTGCCTTAAATGTTGGTGTACTCCATTTGGTTCCCACTTTGGAGGTGTTTCCACTGTTAGCTGATCCAAAGACATATGAGCCTAACACAATTGATCTCTCTGATCACAGTGAGTTGGAGTACTGGTTCACCGTTCTTTCAGAGCATGTACCGGACCTTGTTGATAAGGCTGTTGCAAGTGAAGGAGGGACTGATGATGCCAAGAGAAGAGGTGATGCATTTGCTCGGGCATTTTCTGCCCACTTGAGGAGGTTGATGGAGGAACCTACGGCATATGGAAAGTTAGGTTTGGCTAATCTTCTGGAACTGAGGGAAGAGTGCTTGAGGGAATTCCAATTTTTTGATGCCTATAGAAGCATAAAGCAGAGGGAAAATGAGGCATCACTTGCCGTACTGCCTGACCTGCTGTTGGAACTTGATAGTATGAATGAGGAAGCAAGACTGCTTACTCTAATTGAAGGTGTTCTTGCTGCAAACATTTTTGATTGGGGTTCTCGTGCTTGTGTTGATCTTTATCACAAGGGGACAATAATTGAGATTTACCGAATGAGTCGCAATAAGATGCAGAGGCCATGGCGGGTCGACGATTTTGATGCCTTCAGAGAGAGAATGTTAGGATCTGGGGATGAGAGACCCCCTCCACATAAAAGAGCTCTTCTCTTTGTGGACAACTCAGGTGCTGATGTTGTTCTAGGGATGCTTCCCTTGGCAAGGGAACTCCTCCGACGAGGAACAGAAGTTGTTTTAGTTGCAAACTCTCTTCCTGCCCTAAATGATGTAACTGCAATGGAGCTTCCTGACATTGTAGCTGAGGCTGCCAAGCACTGTGACATACTTCGTAGAGCTGCTGAAGCAGGGGGATTGATTGTGGATGCCATGATCGACTCTGCAGACGGTTCTAAAGAGGGATCATCTTTTGTTCCTTTGATGGTTGTTGAAAATGGGTGTGGTAGTCCATGTATAGATTTAAGACAAGTCAGCTCTGAGTTGGCTGCTGCAGCTAAAGACGCTGATTTGGTAATCTTGGAAGGGATGGGGAGAGCTCTTCACACAAACTTCAATGCCCAATTTAAATGTGATGCTTTGAAGCTTGCCATGGTGAAAAATCAGAGGCTGGCAGAAAAATTGATCAAAGGAAACATATATGACTGTGTTTGCAGATATGAACCATCACATTGAAGCCTTTTTCTAAGTCCTCACTTGTTTCTTCATCAAATTTTTGGAAATATTCTTTAGCTTTTTAATGCGAATACTTATCATTAT

>cucumber_newGene_63 cucumber_newGene_63.1

CCAATTTTAAGCGTCCTTTCTAGTTCTTCATTCTATTTCTTGCAATTTGCATTGCGCCACCAACCACTGCTTCTTCCTTCACCAACCTTTTCTCCTCGGCGGACGCTCCGAGGCCTTTCTCTTTTGCTTCGTTCTCTTCTCCGATCTCAGATCTCGAGCTCAATCCTTCGAAATGCAGAGCTCTGCGTTCACCTTCTCCTCTTCTCTTCCTCTTCTCAAGCCTCGCCGGCCTCATACTTCCTCCTTCACTTCTCCCTCCAACTCAATTCGGTTATCTTCTTCGTCTTCCACAAATTCCAGAGATCTTGGAGACCTTAACAATGTTGGCATCCCTTCCTCTTGGCCCCGCCGATCTTGGACTCTCTCTTCATCTCCTTTTTCGTCATCCAAACTCCGGCCATGGAGTGGTGTACCCTCGCTTGCTTCGGATTCCGATGCGAGTCACTTCAAGGTTCAGGCTACTGCGGTGCCGGATAGCTCAGAAGAGTCTGCGAGTGATGGCGGAAGCTTAATGAAAACGTTGGAGCTCGGGTTGTTGTTTGGCTTATGGTACCTTTTCAACATCTACTTTAACATCTACAATAAGCAGGTTCTCAAAGTGTATCCATTCCCTGTAACTGTCACCGGAGTTCAATTTGCTGTGGGCACTGTACTTGTTCTCCTCATGTGGGGACTTAATCTCTACAAAAAACCAAAGATTAGTGGTGCTCAGCTTGCTGCTATTCTGCCGTTGGCAATTGTTCACACATTGGGAAATCTTTTCACAAATATGAGTCTTGGGAAAGTGGCAGTATCGTTCACTCACACAATCAAAGCCATGGAGCCATTTTTTTCAGTTGTCCTGTCAGCAATGTTTCTTGGAGAGACTCCTACTCCATGGGTCATTTTATCCCTTCTGCCAATCGTTGGTGGTGTTGCATTGGCATCTGCCACTGAGGCCTCCTTCAACTGGGCCGGATTTTCGAGCGCAATGGCATCTAATGTGACCAATCAATCTCGTAATGTTCTTAGCAAAAAGGTCATGGTGAAAAAAGAGGATTCGATGGACAACATCACCCTCTTTTCAATTATAACAGTCATGTCCTTTTTCTTACTAACCCCTGTGGCCATCTTCATGGAAGGTGTCAAGTTCACTCCTGCATACATTCAATCAGCTGGATTGAACATGAATCAACTATACACTAGGTCTCTTCTAGCTGCCCTTTGTTTCCATGCTTACCAACAGGTTTCTTACATGATATTGCAAAGAGTATCGCCCGTTACCCACTCGGTAGGCAATTGTGTAAAGCGTGTTGTGGTCATCGTGAGCTCCGTTATCTTCTTCCAAACACCTGTTTCTCCAATCAACTCTATTGGCACTGGAATAGCTCTTGCTGGGGTTTTCCTCTATTCGAGGGTTAAGCGCATCAAAGCTAAGCCGAAGACAGCTTAAAAAACCCCCCATGAAATTTTGCCTCGGTAAGTTGGTAGTTGCTATCCATGTCGGAAAAAAAAAAAAAATCCATCAAAGATTTGTTTGTAGTTGCAATGCAATCTCCTCTTGTAGAAAACAAACTGTTACGGGTTTTGTTTAATTTCAGTTTTGTACTATTTCTGTGCTTCAGGATGACTTTTTATTCTTTGTGGATAAAGTCATCTTCATTCCTTCTAGACAGTCTTGTGCCCATTTCAATTCACTAGCAATAACCACACGATTCACCTATGCCCTAAATGT

>cucumber_newGene_64 cucumber_newGene_64.1

TTTTTTTTTTCTCTATGAAGAGAGTTCACTTTGATCAGAAAATTGAAGAGACCATATATGAGTTCTTCATTCATCTAATTTTAAGTTATTCTTGAGTGTTTTTTAGTATAAATTAGTTCCTTATAACTCTACTAAGAATATATGTCAAGAAAGCAAGGATCTTGAAAACTGACAGCCCTAAGCAGCAATCCAAAAGATCAAAATGGGATCAACAACAACAAGCAACCATTATTACAGCCCTTGTGCAGCTTGCAAATTCCTAAGAAGAAAATGCTTAGCAGGCTGTGTTTTTGCACCCTATTTTCCGCCTGAAGAACCCCAAAAATTTGCCAATGTCCACAAGGTATTTGGAGCGAGTAACGTGGCGAAGCTCTTGAACGAAGTTCTTCCTCATCAACGACAAGATGCCGTCGTTTCGTTGGCCTACGAAGCTGAGGCAAGAATCAGAGATCCTGTTTATGGGTGTGTTGGAGCCATTTCTTTCCTCCAAAAACAGGTCCAAAGGCTTCAAAAGGAACTTGATGCTGCTAAAGCTCGCCTTTTTCTCTACTCTTGCACTGATTTCTCAACTCCCTTTTTGCCTCACTCTCAGATAATTAATACTCCTCATCATCCAGTGATTTGGAATAATAATAGTAATTATAATATCAACAATCATAATGATATTAATAATCCTTCTATGTATTTTAATGGAGGAATTTGAAAGATGAAAAGGGTTAGTGGGTTGAGTTTATTGAAGGGTTTGTTTAATATTCTTATAAGAACTCAATATTTGAGATCTCTTTGTGGTTGGAAAGAGTTATTGTAGACTATATATATCAATATTGGCTTGTGCTATTAGGGTTTAGTTTTGATGTATTCTATCATATTATTATTATTAGAGTCCAAATCTTT

>cucumber_newGene_65 cucumber_newGene_65.1

CACCACCAAAGCTACCAATAATTGGAAACCTTCATCAATTGGGCAGATATCCTCATCGATCTCTTCAAGATCTCTCTAAAAAGTATGGTTCTCTCATGTTTCTACAACTGGGATCCACTCCAACTCTTGTGGTTTCCTCAGCAGATATGGCCAGAGAGATATTCAAGAATCATGATATCACTTTCTCTAACAGACCCAAATCAACAGCAGCCAATTTGTTTTTCTATGGATACAAAGATGTTGGCTTTTCTCCATATGGAGAATACTGGAGAGGGTTAAAGAAGATTTGCACTCTTGAACTTCTAAGTCAGAGAAGAGTACAAGGATTTCAGTATGTAAGGGAAGAAGAAGTTGAAATCTTGGTGAGTAAGGTACATAAAGCCACATCTGAAGGTGTATCTGTTAATCTTAGTGATTTGATTACCTCAACCTCTAATAACATAATTTCAAGATGTATATTTGGAGAAAAGTTTGAGGATGAAAATGGAAAGAGTAGATTTGGTGAGCTAACAAGAAAGATGGCCAAGTTAGTTGTGGGTTTCAGCGTGGGAGATTTCTTCCCTGCTTTTGGATGGGTTGACAATATCACTGGCTTGATTGGGAAACTGAAAGAAACTTCTGGTGCACTTGATGCTTTTCTTGAACAGTTTATTGCAGAGCACAAGACAAAGAAGAAAGATGATTTTCGAAGTGACAGAGAAGATTTCGTCGACATTCTTCTCCGAGTTCAACAGAGGGATGATCTTGGTATTGAATTTACTCAAGAAAGTCTAAAATCAGTGTTAGAGGATATGTTCATAGCTGGAACGGATACAACTACATCAGTATTGGAATGGACAATTGCAGAGCTAGCAAGAAATCCAACCATGATGAAGAAAGCACAAGAAGAAGTAAGAAAAGTAGTGGGGAAAAAGACAAAGATAGATGAGAATGATATCCTTAAAATGAAATATCTAGAATGTGTTATCAAAGAATCACTAAGGGTTCATCCACCAGCTCCTCTATTGTTACCTAGAGAAACATCTGAAATGGTAAAGCTTGGAGGTTACTGTATTCCATCAAAAACCAGAGTGTTTTTCAATGCTTGGGCGATTCAAA

>cucumber_newGene_66 cucumber_newGene_66.1

ATTATCTATAAATACACTCTAAAACTCTCCAACCAGATTAAGTTTGGTGGGAGAGCAAGATTCAAAAGAAATGGATCCCATCTCATCTTTGCTCTTCTTCATCTCCATCTTCTTACTTTTACTAAAACTATTCTTCTTCAAACCGAAAACCCCCAACTTCCCTCCTTCGCCTCCAAAACTTCCCCTAATTGGCAACCTTCACCAACTAGGCTCCCTACCACACCAATCCTTGGCCACTCTTTCCCAAAAATATGGCCCTCTAATGCTCTTAAAGCTAGGCCAAGCTCCTGTCCTCATAATTTCCTCTGTGAAAATAGCCGAACAAGTCATGAAAACCCATGTCCTCGTATTCTCTAACCGACCCCAAACGACTGCGGCAAAAACCTTGCTTTACGGATGTCAAGATGTGGGTTTTGCCCCTTATGGCGAGTACTGGCGCCAAGCAAGAAAAATATGTGTCCTTGAGCTTTTTAGTGTGAAAAGAGTTGAGTCTTTTCAATACGTTAGAGATGAAGAAATCGATTCTCTTATTAATAAGATTCGTAAAATTGGGAGTGATCAAAGTTTAGATCTTGGACATTTGTTTTTTCAAACATCAAATAATATTGTTTCAAGATGTGTTCTGGGAGAGAAATTTGAGGATGAAGATGGGAAGAGTAGATTTGAGGAGATATCAAGAAAGGCTATGGTGATAGTAACAGCGTTTTGTGTTGAGGATTTTTTCCCTTCATTTGGTTGGATTATTGATGTTATTAGAGGATTTCATTGGGATTTGAAGAATTGTTTCAAAACATTAGATGAGTTTTTTAGTAAAGTTGTTGAAGAACATAAGGAGAAGATCAAGCGATCAGGAGGTGTAATTAATATCGATGATTATGAATCGAAAAAGGATTTTGTGGATATTATGCTACAACTTCAACAAGATGACAAGCTTGACTATCATTTCTCTCTTGATAACCTCAAAGCAATTGTTTTGGACATGTTTGTAGGTGGAAGTGACTCAACAGCAACAGGATTGGAATGGACAATGACAGAGCTAATGAGAAAACCAAGAATCATGAAGAAAGTTCAAGAAGAAGTTAGAACAATAATTGGCAAAAAATCAAAGATAGAAGCAGAAGACATTAAAAAAATGGAATACATGCAATGTGTGATTAAAGAGTCTCTAAGGCTTCACCCACCCGTTCCTCTTTTAGTGCCAAGAGAAACAATGGCAGACGTTGAGATTGAAGGTTACTATATTCCGTCAAAAACAAGAGTGTTTGTGAATGCTTGGGCCATTCAAAGAGACCCTCAATTTTGGGATAATCCAAATGAGTTCATTCCAGAGAGATTTATGGACAAAACTAATTCAGCTGATTATAAAGGTCAAAACTTTGAGTTCATTCCATTTGGTAGTGGAAGAAGGAAGTGTCCTGGATTGTCATTTGCGATTGCTTCTTTTGAATTTGTTTTGGCTAATATTCTCTATTGGTTTGATTGGAAGCTTCCTGATGGTTGTGAATCATTGGATGTTGAAGAATCTAATGGACTCACTGTTCGTAAGAAAAAGTCACTCCATCTTAACCCCATACCTTATGTTGTGTCTAATTAATCATTCTCAGTCTTATAATTATCCTTTTGTATTGTTGTTAATTAAAAATGTCAG

>cucumber_newGene_67 cucumber_newGene_67.1

AAATGGATCCCATCTCATCTTTACTCTTCTTTATCTTCATCTTCTTACTTTCACTAAAACTATTCTTCTTCAAACCCAAAACCCCCAACTTCCCTCCTTCACCTCTAAGACTTCCCCTAATTGGCAACCTTCACCAACTAGGCTCCCTACCACACCAATCCTTGGCCACTCTTTCCCAAAAATATGGCCCTCTAATGCTCTTAAAGCTAGGCCAAGCTCCTGTCCTCGTAATTTCCTCGGCGAAAATGGCCGAACAAGTCATGAAAACCCATGACCTTGTATTCTCTAACCGACCCCAAACGACTGCGGCAAAAACCTTGCTTTATGGATGCCAAGATATGGGTTTTGCCCCATATGGTGAGTACTGGCGCCAAGCAAGAAAAATATGTGCTCTTGAGCTTTTTAGTGTGAAAAGAGTTGAGTCTTTTCAATACGTTAGAGATGAAGAAATTGATTCTCTTGTTAATAAGATTCGTAAGAGTGGGAGTGATGGTAGTTTGGATCTTGGGCATTTGTTTTTCCGAACATCAAATAATATTGTTTCAAGATGTGTTATGGGAGAGAAATTTGAGGATGAAGATGGGAAGAGTAGATTTGAGGAGATATCAAGAAAGGTTATGGTGATAATAACAGCGTTTTGTTTTGAGGATTTTTTCCCTTCATTTGGTTGGATTATTGATGTTATTAGAGGATTTCATTGGGATTTGAAGAATTGTTTCAAAACATTAGATGAGTTTTTTAGTAAAGTTGTTGAAGAACATAAGGAGAAGATCAAGCGATCAGGAGGTGTAATTAATATCGATGATTATGAATCGAAAAAGGATTTTGTGGATATTATGCTACAACTTCAACAAGATGACAAGCTTGACTATCATTTCTCTCTTGATAACCTCAAAGCAATTGTTTTGGACATGTTTATAGGTGGAAGTGACACAACAGCAACAGGGTTGGAATGGACAATGACAGAGCTAATGAGAAAACCAAGAATCATGAAGAAAGTTCAAGAAGAAGTTAGAACAATAATTGGCAAAAAATCAAAGATAGAAGCAGAAGACATTCAAAAAATGGAATACATGCAATGTGTGATTAAAGAGTCTCTAAGGCTTCACCCACCCGTTCCTCTTTTAGTGCCAAGAGAAACAATGGCAGACGTTGAGATTGAAGGTTACTATATTCCGTCAAAAACAAGAGTGTTTGTGAATGCTTGGGCCATTCAAAGAGACCCTCAATTTTGGGTTAATCCAAATGAGTTCATTCCAGAGAGATTTATGGACAAAACTAATTCAGCTGATTATAAAGGTCAAAACTTTGAGTTCATTCCATTTGGTAGTGGAAGAAGGAA

>cucumber_newGene_68 cucumber_newGene_68.1

TTAAAAAGACTGGAAATTGATCCACACCTTTCTTCTTCTTTTGTTTGGTGTCTCTTTCCTTTTTTCTTATTTTTCTTCAAACCCAATGTCATGCCACATGACACATTCAAACACACAGGGGTCCACTATCTCCTTTCCCTAAATTTCCTTTATCACTATTGTTTTTAATTAATTAATTGTTTTTGTCTTTATCATCATGAACGCAACAAGCATTGGCTTGATCTGAGAAATTACCTTTTTTATTTCCCATCTTAAATCCCCAAAGTTAGTTTCTCACACCACATCAGAAAGAAAGCTTAAGGTTGCAAATCGAAAATGGAACCGAGAGGGATTATCAGTCCGAGAAGATCTAAACCCAAAAAGACGCCACCCGCCCATCTTCCTGGCAGCACATCTGCCCCTGAATTAACCATTCAGTCAGAAATTCCCAGTACCACATCCAATCGGCGCACAACTAAATCAGCAAGTCAACCTTCGCCTTCAACCAAGAAAGGCAAAGCAGGTGGGAGTGGCTCTCGTAAGGATGGTTCTGGTTCGGGTTCGAGTCATAAAAAGAATTCTGATTATTCTCCAAATCATTCTCCCAGCCCTGCCAAAACTCGTGATCATCTTCTTCAAAGTCCATTACAACAACTTTCTATTCACGACGTGAACGATGTTGCGAATAACGTCCATCAATCAAGATCGATACACAAAACAGTGAGTTCAGATACAATTATTGAACAACAACATAATTCTGAAAGCAATGCTGCAACAATGTTATTTAAAATTTACGGGGTGATTGCATCTCATCGTCAAGGAAACACATCCATTAAATCTTACTTTAAAAACCTCAAGGCATTATGGGATGAAGCTGCAGCTTCTTCAATCCACACTAATTCCCCTCAATCTTCATCCAATAATACAACTGTGGAGCAGAGTGGGCTCATGGAAAGAGAAAAACTAATGCAATTTCTTTTGGGACTAAACGATTCTTATTCCTCACTTTGCTCCCAAATCCTTCTTGAGAGGCCATCTCCAACGGTGGACCAAGCTTATTCTCTCATAATTCTAGAAGAAAAAACTAGGAAATCCAAAATGGCCAAAAAGAATATTATGTAGTAATGATCAAAATAAACATGTTGCCTTGTTCATCAACAATGGTGATGATAATGCTTATTTTTTTCTTTTTTCTTCTTTTGGTTGAATTGTATTCTTATGTGTTTCATCATTCCTCCTCCGTTGTTTGAGCTTTTTCTCTAGATCCTCCAGAATATCAATACTTGTAAACTCTTG

>cucumber_newGene_69 cucumber_newGene_69.2

TTTCTCTTCTCATTCACTTCGTAGCGAACCAATTCCCTCTCTTCCAAGATGCAAATCTTCGTTAAGACCCTCACAGGAAAGACCATCACCCTTGAGGTGGAGTCTTCCGACACCATCGACAACGTTAAGGCTAAAATCCAGGATAAGGAAGGGATTCCACCGGATCAGCAACGATTGATCTTCGCCGGCAAGCAACTTGAGGATGGCCGAACTCTTGCAGACTACAACATCCAGAAAGAATCAACTCTTCACCTTGTCCTAAGGCTTCGTGGTGGCATGCAGATCTTTGTAAAAACCCTTACCGGAAAGACAATCACCCTTGAGGTGGAGTCTTCTGACACTATTGACAACGTTAAAGCCAAGATCCAGGATAAGGAAGGAATCCCCCCGGACCAGCAACGATTGATCTTCGCTGGAAAGCAACTTGAGGACGGCCGAACCCTTGCCGATTACAATATCCAGAAGGAATCAACTCTCCACCTCGTGCTCCGGCTCCGTGGTGGCATGCAAATCTTTGTCAAGACTCTTACCGGAAAGACTATCACCCTCGAGGTGGAGTCCTCCGATACCATTGATAACGTCAAGGCCAAGATCCAGGATAAGGAAGGTATTCCCCCAGACCAGCAAAGACTGATCTTCGCCGGAAAGCAGTTGGAAGATGGACGAACACTTGCCGATTACAACATCCAGAAGGAATCCACTCTTCATCTTGTCCTCCGTCTCCGTGGTGGTATGCAGATCTTCGTCAAGACTTTGACTGGGAAGACCATTACCCTCGAGGTTGAGAGCTCTGATACCATTGACAAGATTACAATATCCAGAAGGAGTCGACCCTTCATCTTGTGTTGCGTCTCCGTGGTGGTATGCAAATTTTTGTCAAAACTCTCACCGGAAAGACCATTACCTTGGAGGTTGAGTCTTCAGACACAATTGACAACGTGAAGGCCAAGATTCAGGACAAGGAAGGCATTCCCCCAGACCAGCAGAGGTTGATCTTTGCCGGAAAGCAGCTGGAGGATGGAAGGACTCTGGCGGATTACAACATTCAAAAGGAGTCCACCCTTCACCTTGTTCTTCGTCTGAGGGGAGGAATGCAGATCTTCGTGAAGACCTTGACGGGAAAGACCATCACTTTGGAGGTGGAGAGTTCTGATACAATCGACAATGTGAAAGCAAAAATTCAGGACAAGGAGGGAATTCCACCAGACCAGCAGAGATTGATCTTTGCCGGAAAGCAGCTGGAAGATGGAAGGACCCTTGCTGATTATAACATTCAGAAGGAGTCGACCCTTCACCTCGTCCTTCGTCTCCGTGGTGGCTTTTAAATTGTTCTTCTGAAAGTGGAATATTTGGTTTGCGTGGATGGCCAATATGTTTGTGGCCATGTGGATGAATTTGGG

>cucumber_newGene_7 cucumber_newGene_7.1

AATTAATCATTCTCTTCTCTTCTTTCTTTCAACAAATATCTCTTCTCTTTTCTTTTTTAATCTTTCTTTTTTCTTTTTCTTTGAGAACTAATTATTTAATTTATTTTTTGCATTTCTGTTTCCCAAAAATGAAGTTTTTCTTTGGGGGGCAAAAGAAGGAACATATGTGATATGTTGTTGACAGAAGATAGAGAGCACAGATGATGATATGCAAAAACTCATCATCATTATCATTGTTATCAATACTATTCTTTGTTTTTTAGGGTTTAGAAATTTGGAGAGAGACATTATCATTATTATGCATTTCACTCCATTTGTGCTCTCTATGCTTCTGTCATCACTCTCTTCACCCCTTCCTTTTATATATATCCCATCCCTTCACTTTTAATAATCTACTCATTTTTCTAATCTCCTTTTCACCTCTAATCACTTTTAATTTCTTAATCTCTTACATATATATAGATATGTGTATCTATGTATATAGAGGTGCAATAACTAGTTGAGTTAATTATATACAGCCCTAAGAAATTAAATGTGTTGATTTTTCTATGTTTTTAGGTGCTTTATGCAGCAGTTCAAATAGCTAGCTAGGGCAAGGGATGAAAAGAACTATTCAGAAATATGCAAGATTTTAGGTCTTAGTCGTTGAGGTATTCGTAATTGAATGCAGGAAATGAATAGAATTATGATGAAGGGGTTGAAGATATCACAATTAGGAAGATGCCTATATGCCCATGAGATATTAAAAGAGTAAAGTGAATATTCCTCAAAAGAGGGAAATGAGGCTCACAATTGCCACTTGTTTATGATTCATTATATTTGTGGGTGAGAAATGCCCATTATTATATTATTCATCTTCCCAACATTATATATAAATATATATATTGTGTTTTTGTAATACACCAAAAAGATAAAAGAAAAAATGGAGGCAAATTAAAGGGAGGGGTTTGGAATAAAT

>cucumber_newGene_70 cucumber_newGene_70.1

AAGGACTGCATGGTGGAGTGAAACAGGCCAATGCGTAAAAGGAGCGTATTGTGCTTTGCAGCAATCAAGAAATTCTCAGAGATGCTGTGAAAAGGAAATCACAGTAGTCCATGTGTGGGAGTGGTTCATGTTCTTGACGATGTGGTTATTTGGCTCCAGAATATGCATTGGGAGGTCAGCTAACTCTGAAGGCCGATGTCTATAGCTTTGGAGTCCTCATTCTTGAACTAGTTAGTGGTAAACGTAGCAGTACAGTTTTTGGGGTAGAAATATCAATACTCTTGCTGGGAAGGGTGTGGGAGTTATATGAAGAAGGAAAACTCTTGGATATAGTGGATCCTAGACTAGGAGATTACCCTCAGGAAGAAGTGCTTAGGTACATGAAAGTGGCTCTTTTTTGCACACAAGCAGCTGCAAATCGACGGCCAGTGATGAGCCAAGTCATTGACATGCTCACAAAGAATAACAAGCTGAACGAGAAGCTACTTACTGCCCCAGGGTTCTACGGAGGCTTGGGGACTAGTGACATTCCTTCAGTGAGCAAGAAAACCTCAAACGCTTCCAGTAGCAACGAGATGAGCTTTGCAGGCATCTCCATAACTAAAATCACCCCCAGATGAAGAATGATGACGAAGATTGAAGTTCAAGGCTTAGGCAGATTATAAAAGGTTGAGTTCATGGGTATTAGTTAGTTTGATTTGGTTGAAGCATCTATTATTAAAATTGGGTAAATATTAGAGCTTTATTTTGTTGGGGATATAGAGTTGCTTACACAAGTTGCAGCCTCATGATCATTTATCAACTGGTATGATGTTTATACCATTCATATTTTTCTCCATTGAAATGAATGTTCCAATGGCTCTTATCTCTTCCCATTTTTCTTAGATCCTGCTTTTCA

>cucumber_newGene_71 cucumber_newGene_71.2

CTAAAACCATTTCCATTACGTTCCACTCTCTTCTTCCTCCTCCTCCGTAGATCGAGAACCGCCCAAACCCCTCCTTTTCTCAATCCCGCCGCCGTTGGACCCTTATCGCCGCCGTTAACTCCGCCGTATACCTTCAATTTTTGCCTCACAACTGATAATCAACGGTTGTGGGATATTGTGTTCTCTTCGTCTGTGATTTTCCTTTTCGTTCTTTTTGATGATTGGGTTTTGTTTTTGATGAAATTTCGGCGATGAAGATGATGAATAACGGCTATAACAGTAGCTTCAACAGCAATAGTGTTGGATTGATGTCGAACACGGGAGCGACGACGGCGTCTTCGTCGTCGTCGACTTCTACAACGAATGCGAATTCACAATCGCAAGGTCTCAAAACCTATTTTAAAACCCCTGAAGGACGGTACAAGCTTCATTATGAGAAGACTCATCCTCCCGGTTTTCTTCCTTTTTCTCATGGCAAATCAGTCTCTCAGGTGACTCTGGCGCAGCTCAAGGACAAGCCGGCGCAGGCTGGTCCAGCTCCGAGTTCGAGTTCTAGTGCGAGTGCGAGTAGTGGAGTGAGATATGCGGCTGCGAAGTTCCTTGGGGTTGGAGGTGGAAATGGGGTTCGAGCGATTGGGTTTGCAGGTGGTAATGGTACTGGTAAAGCTGTTAATGGAACTAGTAGAAGTGGCTCACTTGTAGGCTCAAACGGCAGTCATTCCATTCTTAACCCCAACTATGATGGTAAAGGAACCTATTTGATTTTCAATGTTGGAGATACCATCTTCATTGGCGACCTTAATTCACCTGATAAGGATCCTATAAAGTCCTTGCACTTTAGCAATTCAAATCCTATCTGCCATGCCTTTGATCCTGAAGCTAAGGATGGTCATGACCTACTAATTGGATTGAATTCTGGAGATGTATACTCGGTATCTTTAAGACAGCAGTTACAAGATGTTGGGAAGAAGCTTGTTGGAGCTCAACATTATAACAAAGAGGGCAGTGTCAATAACAGTCGATGTACCAGCGTCGCATGGATTCCAAAAAGTGATGGTGCTTTTGTTGTTGCTCATGCTGACGGAAATTTGTATGTCTATGAAAAGAGTAAGGATGCTAGCGTGGACACTTCTTTCCCTGTCATAAAGGATCAAACTCAATTTTCAGTGGCACATGCACGGTCCAGTAAGAGCAATCCAATTGCTAGGTGGCATATTTGCCAAGGATCAATCAACAATGTTGCTTTCTCGACTGATGGAGTATATTTGGCAACCGTTGGTAGAGATGGATATTTACGGGTATTTGATTATTCAAAAGATCAGTTGATATGTGGTGGCAAAAGTTATTATGGGGCTCTTCTTTGTTGTACATGGAGTGGGGATGGAAAATATATCTTAACTGGTGGTGAAGATGATCTTGTCACAGTATGGAGCATGGAAGATAGGAAGGTGGTGGCATGGGGCGAGGGTCATAATTCATGGGTCAGTGGTGTGGCTTTTGATTCATATTGGTCGTCACCAACTTCAGACGACATAGAGGAAAATGTTGTTTACCGGTTTGGTTCAGTTGGTCAGGACACCCAATTGCTCTTGTGGGATTTGTCAATGGATGAAATTGTGGTTCCCCTTCGGCGGTGCCCTCCTGGTGGATCCCCGACATTTAGCACCGGAAGCCAGTCAGCTCATTGGGATAGCATTACCCCATTAGGCACTCTTCAACCTGCTCCATGCATGAGGGACGTTCCTAAACTTTCTCCAGTAGTAGCTCACCGTGTCCATACCGAACCCTTGTCTGGCTTACTATTCACCCAAGAGTCTATCCTCACAGCATGCCGAGAAGGACATGTGAAGATTTGGATGAGACCGTCCTCTGAATCACAGTCGAGCAACTCAGAAGCCCTCGTCGGAGGTTCTAGCTCGTTGGCAGGTTCCAGAGTTGGTTCCTCTATTTTGAAATAGTTTTGGCAAAATTCAGTTTCAACATACCGGGATCTCGCTTGTACATGATTATGCCGTTCCACCCAGTTGGGTCCTGCTATTTGGCGAATGGTGACGGGACAGTTTTTTCTTTTGAGGTCTTCCTTCCTTATTCTCTAGAGATTAGGAAAGAGTATTACGTTTGGTCAGCATATTGACTCGCTGCGAATTTGCAGTGGGATTAACCAATGGCTCTGCTTATAATCACTCTTGTTTGACATATAATATGCCATAGTAAAATGCACAGGCTATTGTTACACATCGCGGGGAACGTAACATAACTCATCAATTAATAGGCAGGGGGGGAGATGACAAAAAATAAGTTTATCGAGGGAAAACGAAAAGGTATGTCAGTG

>cucumber_newGene_73 cucumber_newGene_73.1

TAAGGACGATTGTGCGGCACTTGTTCTAACTCGATGAACAAGTCGGCCAATAAAAATCTGAAAATCACTGACATAGTCGACATATGATGTAACCTTCTTTTGCATTCTTGGAAAATTTGTTTATAAAACGTGAGAAAGAAAAAGATACATTTAGATGTTATAATATCCATTGAAGACTGTCAGTTGCAAGGAAAGATGTTTGCTTGCAAAGAGTACAACTAATGTTTGGGCTGTGATTCAATTGGTATGTCTCCCTTATAGTACATTGTGAACATTTTCACTTCTGGTAGACTTGCATGTGAAAATGCCAAACGTCACACCTAAGCTTTATGACTTAAAATGAAAGGCAACTACTTAAATTAGGACAAGTATGACTGTGACACTACTCATCAAAGGATGTCTATTATCCTTGAAATAGGTATCGTGCTATTTTGTCCTCTAATTTCTGAATTGCGATGTCACATGATTGCAAGGCTTTAACAACGTCACAAGTTCTACGTCGCGTGCATTTCCTCCACTGGTATAATATGTTATTTGGTGTGATCAAAGGTGATGTTTCTCCCATATCCTCCACTGGTATAATATGTTGTTTGGTGTGATCAAAGGTGATGTTTCTCCCATATCCTTATGAATTTCTTGGTAAAAGCCAAATTGTCACTCAAACTAATTTGGATAGTATGATTGTATAATATAAAAGTTTTTGTAGAAGAGTGATAAGTAGCATGAATGCATACTTACAAGATAGAAAGATTGAAGGAATGAGGACTCATGAATATCTTTTGGTTCTTAAGGTTTGTGTGCCACTAAACTCTTGCACTTATGTAGATTAGTTCTTGCGCATCGTATTCTCCCAAATAAATAGATCCACATTTGTGGGGAAAGTTGGCCATCTTGTGATAATGAACTTCTGTTGGAATAGAATAATGTGTGCCAAAATAATAAGCCAACCAGTCATGGACATAATGCATGAAAAAGTGAAAGACATTCGAAAAGTTGTAGATATTTTCATCGGCCATTGAGCTTACAATCTTAAAGAGTCTGGGATGAAGGAAGGATCTCTTTTGAGGAAATACGAAAAGGTCATCCGATTGGATTTGAGGCTTCAGTTAACATACCTAGCCCATGAGATTTGCTGGCAGAACTGGGATAGCAAGATTGAAGATATTTTCATTCGACCATTAAGCTTACAATCTTAAAAAGTTGGGATGAAGGAAGGATCCCTTCTGAGGAAATAAGAAAAGGCATAACCAACAAGATAGAAATGCAACTAAATATGGTTCATCCTTCAAAGAAAATGTTTCCTGTTGTGGACCTTTCTAGAGACTTGCTCCACATCTCATCATGATTTTGGATGGATTTGGAGCATGATGCATTATTTTGTTTGTGTAGTTAGCTTATCATAGTTTCTAGAGCCAAGATACCATAATGAAACCTAGGAACTCATAGTCATTATTGGAACTTCATGTCCTTAAAAGATATTTTGCCTTATCTCGTAAGGTGACTAACTCTGTAAGGGAAGATATGCTTTTCTTGTGGAAATTCCTTCAATAAGGAAATGGATAATTCACCTACCATGGTATGTAGAGTATTTGTTGAAGGATACCATACTTCACAAAATGTTCGAACCACATCACTATTACGATCATATATGTACAAAGTTCAAAGTTGTTTGGACGCCAAATAAATGAGCAATCATAAGTAAGTTGCCTATTTCGACTAGCACAAGCCATTTCCAATAACAACCAATAAAGTTGAATTCATCTAGGACCATAGTCATGGCACCCCAACGACTTTGGCCTTTAATTATATGATTGTGAGTGAGAACTTGATCATAATTATGTGCCTCATCATGGATAGAAGGTTGAAAGACTCATGCATTTTCTTTAAATCAAACGTATCTCTACTGATTTGAGAAGAATCAGGTTGTCACCTAAGTTTGGCCAATAATTTGCAAAAGGGCAAGCCAATGGTTTCTCTACAAGGTCTCTTTTTACATGATAGTCGTCATGTGAAATAAATCATTGTTCTGAAAAAGAAATAGAAATATCACTAAAAACTGATGACATAGTCTAGTAAGAAGGAATATATGAAAAGTAAAGTATAATAAAAATCCTAAGAGATTTTATTTTTGAAGCATGCATTGATATTGTGGTATTACCACTTCAAAATCGAGTTGCAGTTGAAAGTCGAGGAGTGGTATAATAGAGTTGCCTAGACTGTTTTGAGTTCCTTTCTAACACGAGCCAAATCATGTTGCAATAGGAAGTTGAAGAGTGGCATGATAGAGTGCCCACGCCTTTCTGTGGTTATCTCTAACATGAAATAACTCGAGTTGTACTAGGGAGTCGAGGAGTGACATGATAGTGTGTCTAGACCATTATGGGGTTATCTCTAACATGAATCAACCTGAGTTGCAACAGGAATTCAAGGAATTACATGATATAGTTGTTTGGTTTGCTTGAACCAATCTGAGAAGATGAGTGACATGATAGAGTGTCTAGACCATTATGGGGTTATCTGTAACATGAACCAAGCTGAGTTGTAACAGGAAGTCAAGGAATGACATGATAGAGTTGCTTGGTCTGCTTGAACCAATCTGAGAAGTAGATATCTCTTTATATGTGCTTGTATGGAGCCTTTAAGGAATAGCTAAAAAAAATTATATATAAAAGGAAAAAAAACCTTTGTTAGAGTTTTCGTAAGAAAAAAAAAAGGCAAAAAGTAAAAAAAAGAAGGAACTTTTTTCCTAACTTCATAGATTTGTTTTCTTCTGCATGATGAGGAAAGAATGTTCAAAATGTAGTCTATTTATATGGACAACAAGTTAATTTCCTAATAGGATTTGTAATCGTGCCAAATCCTTTTTTAGTAATAAAAAAATCTAACCATGTATTTTCATTCTTTAAGCTTTCATTCTTTAAGCTTTCTAGCGTAAGACTGTTCACTTTGTGTGTGTTCTTCACTTTTACTGGAAAACTCAACCTCGTTTGAAACGTCAAATCACTTGTCTGAAACCTCAAAGCTTTAAAGTTAAACCAACCTTGAAACCCTTGAAACTTCCAAATTCCTCAAAAGGAAGGAGGAATCTTCAACTCCATCGAAAAGAAGAAGCTTATGGGAAAACTCAACCCTTCAACCTAGAAAGATTATGAATCTTCCTCGTAGGAATCTAGACTTGAAGGGTCTACGAGAAAGACTTCAGTCTATCCCAAACTTAAAATCTAGACATGTTTTAAAGTAAGTCCAACACCCATTAGGGTATACTCAAGAACCAATCGCATCTACAAACGATTGGATGAGAGACACTACAAGAAAGAAAGCGTTCGTTGATGAACAAAAGCATAGTCGAAGGGCAAATAAAACCCTTAAGCATGTATAGAAAGGGGCTTAGAGACAAGCTCAGTTCTAGTGTTTATCCTCCATCATTTGCAACCTAGAAGGAGAATAAATGTGGTGTTTAGGCATCAAGAGGTTGTTCGTTTTATATAGAGAGGTAACGTGATTCTTATAAATAAGGAAACTTAGATGGCTATCCCATAATTCTTCTTTATCATTTGTATTCGGAGAGGTGAGCAAGTGTGGCATTAGGCATCAAGAGGTTGCTCACCTTAATTAGAAGATGATTATGTAATCTCTATCGCATCAAGGGCCACCATTTGGTTTAATTATCCAAAACGCAGAAAGCTTCCTTCATATTTCTACAGCTTAAGTTAGTTCATTTTTCACAACTGCACCATCTGATGTCAACTTCTACAAAATCTCTCGTGTAAATAGTAATACATATATTTCACAACTGCACCATCTGATGTCAACTTCTACATAATCTCTCGTGTAAATAGTAATACATATATATAACTAAGTTTGATAGATTTTTTTTTATGTTCAATCTTGATTTATCTAAGAAACCTCTTGCTTCGCTTGTGCAAGTGAGAGAAAAACTTGTACTTAATGCAGAATTAGTAGTTATATAAGGAAAGTATCATATACCTTTTATTGCACGATCCTAACCTAAAGACATAGTGAGCATATTCCATGTGAGACCATGTTTATTACATAGCATAGTTGCATACATAGAATCTCAATGGCGAGTGATTTTCTAAAAGAAACATTAGTGTACTAAAGCATTCTTTCCAAGTCTTGCTTTGTTGTAAATGTTTAACGCTCTTAAAGTTTATTGAATAGCTTGTTAATTTGAAAGAAATTTTTTAAGCTAAAGCTTCAATGTGATGATTGTATTTGGATACAAGTAAACTATTAACTTTATTCCTATTATAAGCTAAGTTATTATGTGCACATAATGAATCAAGACAACCATAGGGTGTACGGATCACATGGCAGTAAGAAGTTAGCCAATGTGTTGAAATGAGTGTATTGAGTTAATAGCCTGAACAACGAGAAGTAAATTGGAAACTTTTTCAAGGGATTCTAATGATTGTGGTAAATCACAGTGGTTTATGCATTGAAGTAGTTTATGCATTGAAATGGTTAATGTTCTTGGTGAAAAAGAAGTAGTTTATGCATTGAAATGGTTAATATTCTTGGTGAAAAAACTAAGAGAAACTAATGTATGTTTTATTACTGTCTTGTCATAAAGATATCATGTGTTGTGTTTATCGCTTAGTAGTTCTAAAAGTGCAAGTTCAATTTAAGATGGAGCATTTCACTTAGAGGTGTCAAAACTACTTAAGACGCGTTTTTGTGTCGCAACGTGAGAGTCGAAACACGTTGCATGGCGAAGCGTGGCAATTTGGCTATGACATAATTTACGTCGTTACATTTTAGTTTTCTAATAGTCGTGGATTGTGTACCTTTGACTGAGTGCTTTACTATACACGTGCAGGCTCCAACTAAAAGTTGATCTTTGAAATATTTATGGTTTCTTTATAAAAGTTGATCTTTGAAATATTTATTGTCCTTTTTATATAGTTTAAGATATGTGAATTCATTTTAAAAATTCATTTCATACAAGTAATTGACACAAGATGTAGAATTTAGTTTTAATTAATTTTAGTCGTCACTATTTATTATTATTTAAGATTTTTTATATTTTTCTAACTAATTTTAAACTTAGGCCAAAAAAGTACCATTTTTTTTTGTAGTAGAATTTTTTATAAAAAATATATAACAAATCAATAAAATATTTACATGGTATAGAAACAAGTGATTAATTGGCCACACACGACACACCCGTGAGTAGTACTCTTCAAAACAATTGTGTACTACACTCTAAATAAACGATCTAAAGTTTATTTAGGTCATGTGATATACGATTTTTTAGTTTTATTTAATGATGTAAAAAAGACTTTAAATTTAAACAATTGATTATGATAAGCAATCGTGTAGTCATTAAAAACATCAGATCTGACGATCGTGTTGACAATGAAATGTATCAATTATGTTATACGAGTGATTGATTAGATCATGTCGTTCATTTTAAACAATGAGAAAAAAAATTTCAAAATTAAATGATTGTATTAGTTATTTAAAATAATATTGAAACGATTTTGATGTTCTCGAATACAAGGAAGAATATTGAAACAATCATGAAATAACTTTAAATAATCTTGTCGAAAATGGAGAAGAATAAATTTTATTTAAAAGTAGGAGGAAAATATGGAAGATGAGAGGAAAAAATGTAGAAGGGAAGAAATTACAAGGAAGGAAGAAGAAAGTGAAGCAACGAATTGTTCACATTTTTCAGAAATAGACATTTATATGGGTAAAGAAGTCACTTTGAGCTTTCTTTGAGGTCAAAGGCTCAATAAGCGAAAAAGGTTAGGGAAACGAAGAAGAGAGTTAGTGGAGAAGAAAGGAGTGTGTGGTCTCGCAGCAGAATGAAGATCTCTTCTATTCCCTTATCTTGGAACCAAAATTCATGGGTTCAAACGGATGTCAAAGGACGCCAGAACTGCCTCAATGAACAATGGTTGGTAGAAAAGGAAAGAAGCTCCCCAACTAAAGAGGCACTTATCTATAGGCCCACTAGCGAGAATGGTCTCAATCGCGGGAAAGAGGAAGAAGGAGAGGGAAGGAAGCCTAATTCCTGATTCTATCTATTGTATATCATTTCTTGCCCGAGCGCATATCATGATCGTCGTTTAGATGTAATTAATTATGTATTGACTGAAATATTTTGGATATTTCTAATTGTGGATTTTAAGTTCCTTTTAAAGTAGAATAATAATTCATAGAAG

>cucumber_newGene_75 cucumber_newGene_75.2

TGGAAGTATGTGGGAAAATTGCTCTAACCATAAACCTTGTAAAGATTACCACATTGTTAATAGTTTTCTCTTTAAAGGTGATGTTTTGTGTGTCCCTCATACATCTTTAAGAGAAGCAATAATTAAAGAAACACATTCTAGTGGATTAGCAGGACATTTTGGCCGAGACAAAACTTTGGTTACAATTATCTCTAAATTCTTTTGGCCACAACTCAATAGGAATGTTACTAACTTCATCAAAAGATGCTCCATTTGCCAAACAGCCAAAGGTAACTCTCAAAATACAGCCACCCACAAACTGACGGCCAAACAGAGGTGACCAACCGAACTCTTGGCAACCTCATTCGATGCCTTAGCGGGGACAAGCCTAAACAATGGGACCTAGCTTTACCTCAAGCATAATTCGC

>cucumber_newGene_76 cucumber_newGene_76.1

TAGGACTGTAGTTTTAGGTGAAATGGCAAGTGGAATGATGGGGATGAATAAATCACCTTTAACGATGTTACAGTGAGGGGCTAGAACTTCAGGCTCTGATCTTTAAATTTATGATGGCAGAAGATTCTCTAAAACGTGAGTCTAGGAGCAAGGTTCGCCATGCTGACATCCAAGCTTCCAAGGTTTGTGTTATTTGCTTCTTAAGCTTTACTTTAATATGAAGTTCTTGCCATTCCATGTCATTTTCACTTCTCCTGTTGGTTGTAGCCAACATTATTCATACCCTCTTGGGCCCAAGACCTATGTTGAAGATGTTACTTGATGCTCAAGGAGGTTTTTTCTTTTTTTTTTTACCCCTTTTCTTTTAATTGTTGGATGACAATATGAATCTGTTTTTGTATTTCCGTCGCAAGTGACAATATTTTGTCACTGTTTAATTGTTTCAAGTAGAGTCCAATGATTTCCCATCTTCTGAAACTTTCAA

>cucumber_newGene_77 cucumber_newGene_77.1

TTCCTCTTCATCAATTTTCCGTTTCGAACAAATCACGATTCACATTCATTACTGCTCATTGCTGTTCCCTTTCCATTATCCCACCAAACTGATGAGCGTTGACGAAGCTGCTTCTTCTTCTTCCTCCTCCTCCTCCTCCTCCTCCTCATTTCCATCCCTACCCTCTCCTCTCTTTTCAGCACCAGCTTCTCCCATTGCTTTTACTCCGCCAGATTCCACCTTCATTTCCTCCAAATCTAACACCCCTTTTTTCCCCCCCTCTCATTTCTCAAGAACTAGTAAGATTGGAGTTTTGGGTTCTTTTTATTTCTTGTTTATTTCTTGTTCTCTGACTTTTGCTTTCTGCGAGTCTGGCTTTTGGTTGATATTGGGAGGGAAAGATTGTTGTTGGTTTCAAATGGAGTAATTGATTTATGGAAATATCTTTTGATTTTGTGCTCGATTTTGGGTGTGAACTTTCTGTCCCCTTTATTGATTGTGCTGTTCTACTCCAACCTTATTATGATTTACGGGTTTCGGTGCCATTCTCAGGACTCCTCTTTATTTTCTTTTACTTTTTTTGTCTCTCATATATTCTTGAATTCGATTTCTTCATTGTAGATAGGGGAGAGTTGCATCTTTAGGTGGAATTTGACTTCCTGAAGGATCCAAATTTCAGAAGGTAATTTCTAAGCTAGTCTGGTTTTATAGAGTTCTATTGGGGCTTGGCCGATGGCTTTTTATCCAAAGGAGTACTTGAGGATTATCTTTTCAAGACTGTTTTAGTTGGAGATTTAGCTTGCGGAGTAATTGAGACTTTTTTTTTTCAAGATTTGCTAGAGACTACCCTAATTTGAAGTCAACCACCTGAACTATCTTTTATTAATGTTTAGTTCTTTCAGAAACATTGTTAAACGGACCAAAAGTCTTAGTTCATCGGAGTCTAAGATCATTATTCTTTTTGAGAATTCTTGCAAAGCAAGCTTTTTAGCTGGGAATATTGGAGATGGTAGTGATCCAATCAAGATGAATGTTGAATCAGAACCTGCAACTGAATGGGAGAGTTTACTTGAACCTTTTGATCTCACAAAGCTTAGGAAGTCTCACATTCTAATTACTCCTGTTCAACTTTGTAAACTGCTTGAGCTCCCACTTGATGTTCCCACATTGTTGGAAATATTTGAACGGGTGGGTGGCCAGAAGGGCTATTGCCATACATTTGATGTATACTATGTGTTTATAAATAAGCTTGGGGCAATTGGAAAGTTCAAGCTTATAGATAAACTTTTAATGCAGATGAAAGAAGAAGGGATTGTTTTTCGAGAGTCCATTTTCATGATAATTATGAAACATTATGGAAAAGCTGGACAACCTGGACAAGCAATTAGATTGCTCCTAGATATGAGGGCTGTCTATTTATGTGAACCAACTTTCAAATCTTATGATTTAGTTTTGGAAATACTTGTGACCGGTAATTGCCCACAAGTTGCTACAAATGTTTTCTATGACATGTTGAGTAAGGGTGTCTCTCCCACTGTTTTTACTTTTGGCATTGTGATGAAAGCTCTCTGTATGTTTAATGAGGTTGATTCTGCATGCTCACTCCTTAGAGATATGACAAAGCATGGATGTGTACCCAATTCTATTGTTTATCAAACTTTAATACATGCACTATCCCAAAAAAACCAAGTTAGTGAAGCCTTAAAGCTTTTGGAAGAAATGTTTGTTATGGGATGCATGCCTGATGTTCAAACTTTCAATGATGTTATTCATGGCCTCTGCAAGGTTAATAAAATTCATGATGCAACAAAATTGGTTGATCGAATGCTTCTTCGAGGTTTTTACCCAGACAATATGACGTATGGTTTTCTGTTGCATGGTCTATGTAGGATTGGAAAACTCAATGAAGCAAGAAAAATATTGATCAAAATCCCTTGTCCAAACAATGCAATCCTCAATACTTTAATCAATGGGTACGTTATGAGTGGACAGCTTAAGGAAGCTCAAAGTTTTCTTAATGAGACTATGATAAATTTTGGTTTTCAGCCTGATATTTTTACATACAACATCTTGATGCATGGTCTCTGCAAAGAGGGGAGTCTTAGTTTTGCTCGTGATTTGGTCAATGAGATGTCGAGGAGGGGTTGTGAGCCGAATGTGATCACATACGCGATACTGGTTAATGGGCTTTGTAAAGCAGGGCTTCTTGAAGAAGCTGGACTTGTTTTACATGAGATGTCAGCAAGGGGATTGACTATAAATTCAGTTATATACAATTGCTTGATATGTGCTCTATGTAGGAAAGAGAAGGTTCATGTTGCTTTAAATCTGTTGAGTGAAATGTGCACGAAAGGGTGCAAGCCTGACTTGTTTACGTATAATTCTCTAATCTATGGATTGTGCAAGGTTGATAGGATTGATGAGGCCTTCAGGTTGTTTCATAATATGTTATTGGATGGTGCAGTTGCCAACAATGTGACTTACAATACACTGATTCATGCACTTTTAAGGAGGGGTGCATTCCAAAAAGCACTCACACTTGTTAATGATATGTTATTTAGAGGATGCACTCTTGATAAGATTACTTACAATGGTCTGATTAAAGCATTTTGCAAAGTTGGTAATATTGAAAAAGGATTGGAACTATATGAGCAAATGATAATGGATGGACTTGGTGCTGATACTATATCGTGTAATATTATGATCAATGGGCTCTGTAAAGTTGGAAAAGTGGACAACGCATTTGAATTTCTAAGAGATGCAATCAATCGAGGTTTTGTGCCTGACATAGTTACTTATAACAGTGTTTTAAATGGACTTTGTAAGGTTGGACGCATCAAGGAAGCTTTAAATCTATTTGATAGATTACAAGTTGAAGGTGTTCGCCCCGATGCTTTTACCTATAACACTTTTATCAGTTGGCAGTGTAAAGAAGGCATGGTGAATGATGCTTGTTTATTTTTTTATAGAGGCATAGAAAATGGTTTTGTACCCAGTAATCTTACTTGGAATGTGTTGGTCTATACTTTGTTAAAACAAAGTAACCAGGAGAATAATTTTTTTGTGTTAGACGAGTTATGGTGATAAGATGTACTTGTGAAATAATTTTTCACTTTAGATATTTCAGTTTCTTCTTGTGAAGCATACATTTTGCAGAAATTTTAAAAATGTTAGTAGTTTAGCGAATTGGTGGCACCCTGAGAAACAAAATGGAGAATCATGAGGCTTAATTTCTTGTCGCTTGATTGAATAAGTTTTGTGCATGTTCATACCTCATTCAAATAGGCTTGATTGAGTTTC

>cucumber_newGene_78 cucumber_newGene_78.1

CCTCTCCCCCACCTTGTTCTCTTTCGTTGGTTGGCCGCCACACCCGCCGCCGCTTCCTCACGCTTCAGGACAACCACAAACTGCGCCGCTCATCTCCTTCACGTCGTTTGGTGTTGCGTTGCAGCCTTGTCTCCATCTAATGATGTGCCGCTCGATTCACCAGACGATGACGTGCACGCCGGTGAGCAAAGCCCCACCACATCGCCCTCAACACAGTCGTAAGCCGGTTCTTCAACACCTCTCCTATAGCCGTCGTGCGTGGTAGCTCACTAGAGGCCAAGCAAATCTTTGTCCATCTCCATTCTTCTTTCATATCCTATTCTTAGAATTTGAATATTAGAGGAGTTGGAGCTGCTGTCCAGCCGTTTAAGAACCCAAAATAGAGTGTTTTGACACAACTTTGGTAAATTTCTAAACTTCTTGGAGTCAATCTTAAATACCTGTTTGATTCTAGTGCTATCTTTAAGTAACCGATGATGGTGGACGGTTGATTTGAACTAAGAAGGTACTCAGGATGTTGCCCATTGAAATCAAGAGTACTATGTTGAGTTCTTGGTAACGTTTGGAATAGTTTCGATAAATCATCTTTGGTTTTTATTGTGGTTTGAGCTACACAAGAAGAATTAAAATATACAGTTGGATTCGTTTGAATGTTAACTCAATTTCTAACAAGTTTAATGAATAAAATTTGAGCAGATTGTGAGGCTAAGTGAATGATTTCTGAAGTAGAAAATAGATCATAGACTTTTCACGTAATTCCAAGAAGCCTCTGGTTTTTCCCAAATTAAAAACCCTCATTCCTCTCGCAAATAAGCCATCTCAAAACGCGAAGAAGACCATCTTTGGACGACTGTCGTTCCCGTCACCACCACTGCCCTCTCATTCCCGTCTCCTCACTCTAGTACTGTGGCAACCTCCTCTATCGAACGACAAACCTCCTCTATGGTAACTTGTTTAATTCATGCCTGTCATCTCCAATCCGCCGCAATCTCTTCAACATTCAAACCACGACCGTCACCTCCTCTGCTGGTAAATCATTATTTCCTTTTCTCTTTCTCTCTCTGCGCAAAGTTGTTCTCCTGTTACTATTTCCCTCAACTCCATGAGTCGTCGGTAAGGTTTGAAATTTTCAATTTCGTTTGAATTCCCTCTTTTGGACAAACACCCCTACGTGATGTATGTGAAATAAATGTTGGATTTATCTTTAACTTAAATTAGACCAAAAAATGTTAAGACTTGAATCTGGAGGAGAGCAAGACAAAAAGGGAGAAGGTGCTAAAAATGTCCTTCATGTGTTTAGGTTTATGAGATATTTATTTAAGGAAATTTCGATTTAGTATATATTCTTTTATTATATGACACCTCCCCTTGTCTTAATTTTATATAATACTATTTGGTGATGTGTTTTGTTGGTTTTTCTGGTGTGCATTTTTTTATGAGAAGATTGTTCTTTGGTGTGTTTAGGGAAACCTAATAACTTCATCCTTAGTTGAGAAAGAGTTCTCTAACTCTAACTTAGGAGGAGCTTAAAGGTAAGTATTTGTTGATGGCAGTAGACAGTGGGCCTTTATTTATCAAATTGTGGGGTCTAATTGAATTTCTTTAACTTCAAATTTTGCTTCAATCTTTCTATTTTAGTATTTTTAGAACACTTACTTTTGGTCATTGATGTTAATTTTTCTTTATTTTTGTCTTTACACTCTTAAATTAATT

>cucumber_newGene_8 cucumber_newGene_8.1

CGCCGTCTCATACTGAGGCTATCTTCTACCTCTTCAAACCACTGAGACTCAAAAACCTCAATATCACCTTCAAGATGGATCATACGAAGCTCCAGAAAATGGCTGGTGCAGTTCGCACTGGTGGAAAAGGTAGCGTGAGAAGGAAGAAGAAGGCTGTCCACAAGACAACTACCACAGACGATAAACGGCTTCAAAGTACCTTGAAGAGAATAGGAGTTAATGCAATCCCTGCTATTGAGGAAGTCAACATTTTCAAGGATGATGTAGTTATTCAATTTACTAACCCAAAGGTTCAGGCTTCTATTGCGGCAAACACATGGGTTGTTAGTGGTTCTCCTCAAACGAAAAAATTGCAGGATATTCTGCCCGGAATTATCAACCAATTGGGACCCGATAACTTAGACAACTTGAGGAAGTTGGCTGAGCAGTTCCAGAAGCAAGCACCTGGAGTAGGTGCTGGTGCAGCAGCAGCTGCCGCCAATGGTGAGGATGATGACGATGATGTTCCTGACCTTGTTGAAGGGCAGACCTTCGAAGCTGCT

>cucumber_newGene_80 cucumber_newGene_80.1

CAAAAACTTTGTGTTTGTAAAGAACCTGGCCATGTTTTTCCACAGCTTTCTTGAACTTAAAATTTCTTTCATTCTCACAAACAAACTACAATCTCAATTCTAACAAAAACACTTTCAAGCTCAAGAAAGCTGTGGGAAATTATGACACTTTCAGGCTCTCTTCAATTTCTTCTCAATTCCTTCCCTTCCAGGTACTCCAACAGGTTTGAAGCATGCAAATGCATGGGTAATGAAGGGTGAGTGAGTGAATTTGTGCTGAGTTTGAGAATTTTAAAATTGAGATTCATTCATTCAATAATTTTATTTTCTTTTGAAGAACAAAAAAGGGTGAAAAGGGAATTTAGTGTGCAGAGAAGATTCCCCCGCCCAACTTTTGTTTATTAATATCATCAATCATAATAATACTCATCTTCATGTAGGGTATACTGCTTCTGCCCCCCTCTCTCAGTTCTGCCACTCTCAACTTTTTTTTTTTCTTCTCTCTTTATCAAAAACATTACATACATATATACATTTAAAAGTTGTTTAATTTAATTTTGGTCCCTTATATGCTCCTACATTTCTGTGAGAAAGGTCTAATTTTGTCACCATCTCACCCCCTTCCCCTTTTTTTAACCACTCTCTGTCTTTCTTCCCTTCTATACATATCTCTCTCTCTATCTTTTTTTAACCCTTTCACCATTTTTCACTCATCCTTATTCAAATTAAATCAACCAAAAATTGGCTTTTATTCATTTACTGCATCCTAATTTTCTTCCAAACCCAAGAATATACATATATATATATATTCATGATATTTTCAATAAATAGTTTCATTTGGTTCGATTTGGTTGGTTTGAATCTTGAATCTAAACGAGACATTTAGAGTGGGGGAGAGAAATGTGATATGAATATATTGATAATTGGCAAAGCTCCCACTGCATCAATAAAGGGGTCTCAATTGGAGAATCATATTTTTCTTTTTCTTTTACTAATTTATTTATTTACTTACATCTATTACCATATATGTCCCAAACTTTATACATTTATTTTGGAAATTTTATTTCAATTTGGAAGAAGAAAACTAATGGGTTTTCATGGGTGATTTTAAGCTTATTTTTAA

>cucumber_newGene_82 cucumber_newGene_82.1

TTCTTAACCAAACTTTTTCCATCAAGTAATGTAATAGAGACTCAATTCATATCTAGTTTGTTGATTACGCTAAAGAAACAACGGGTGGTCTATTCTTTGATCCAAAAGAAAATAGAGTGTTTGTATCGAAAAATGCTACTTTCTTGGAGGAAGACCACATGAGAGATCATTAACCACAAAGAAAATTAGTATTAAATGAAGCTACAGATGAATCAACAAGGGTTGTTGATGAAGCTGGTCCCTTATCAAGAGTTGATGAAATCAATGCATCATGTCAGTCTCATCCTTCTCAATCGTTGAGAATGCCTCAACGCAGTGGGAGGATTGTATCACAACCTAACTGTTACTTGAGTTTTGAGTTTAACTGAAACTCAAGTTGTCATACTAAATGATGGCATTGAGGATCCATTGTTCTATAAACAGGCAATGAATGATGTAGACAAGGATCCATGGGTTAAAGCCATGAACCTTAAAATAGAGTCTCTGTACTTCAATTCAGTGTGGGAGCTTCTAGATCTACGTAAAGGAGTGAAACCTATAGGTGAAAATAGATATATAAAATGAAGAGAGATACAGCTGGGAAGGTACAGACCTTCAAAGCTAGACTTGTAGCAAAAGAGTATACCCAAAGGCAACTGGTTGACTATGAGGAAACTTTTTCCCACGTTGCTATAATAAAGTCTATAAGAATTCTATTGCCCATAGCCACTTTTTATGATTAAGAAATATGGCAAATGGATGTCAAGACTACTTTTCTTAATGGCAATCTTGAAAAAGTATCTTTCTGTCTCAGCCCGAGGGGTTTATAATCTCAGTTTTTTATTTCCGTCTCAAAACGTTGAAAAAAGGGTCAATGCTACGGCTTAGACAGAAACTATCCAATTTAGAGCAATATCAAAGGTCTCGAAAGTTTACATCTAACTATAGGGTGGTCTCATATTGAGTAATATTAAAGTTTTTTTTACCAAAAAAGTCGAACAATGATGCCCCAGACTCAAGTTTTCAGTTTCGTCAAAAAACATTGAAAAAAGGACCAATCCTACAGCCTAGAAAGAAACTACCCAACTTAGAACAATGTCAAAGGTCTTGAAAGTTCACATTTAAATACTGGGTGGTCTCATTTTAAATAATATTCAAGTTTTTTACTCACAAAAGTCAAACAGTGATGCCCAGACTCAGTTTTTCAGTTCCGTCACGAAACATTGAAAATATGGTCAATGCTATGCCTAGAAAGAAACTACCCAACTTAGAACAATGTTAAAGGTCTTGAAAATTCACATCTAACTACTGGGTGGTCTCATTTTGAGTAATATTAAACTTTTTTGCAAACAAAAGCCGAACATTGATGTCCAAGATTCAGTTTTTCAGTTCCATTTCGAAACGTTGAAAAAAGGGCCTATAAACGACCTAGAAAAAACCTATCCAACTTATAACAATGTCAAAAGTCTCGAAAGTTCACATCTAACTACTGGGTGGTCTCATTTTGAGTGATATTAAAGTTTTTTGCCCACAAAAGACAAACAGTGATGCCCCTGACTCAGTTTTTATGTTTTGTCCCGAAACGTTGAAAAAAGGGTCAATGCTACGACCTAAAAAGAAACTACCCAAGTTAGAGCAATGTCAAACATCTCGAAAGTTCACATTTTAAATAATATTAAAGTTTTTTGCCCACAAATACCGAACAGTGATGCCCTAGACTCAGTTTTTCATTTCTGTTTCGAAACGTTGAAAAAATGATCAATGCGTCGTCTTAGAAAGAAACAACCCGACTTAGAACAATGTTAAAAGTTTCGAAAGTTCACATCTAACTACTAGGTAATATCATTTTGAGTAATATTAAAGTCTTTTGCCCAGATAACAATGTGATAGCTTGTAATTGGTATGAATATAACATGTCATAATCATTTTTCTCTTCATCCTATACGAATTATTAGAGACATCCACAGAGATTTGGTTTATATGTGAGTTTATAAATTTAAATTGTGCAAAACAACATGTTTGGTTAAATATATCACACCACTTGTCTTTTATTAGAAGTAACCATTTTGCTATTTTATTACAATATTTAATGCTTTGTCATAATCTCCCAAAGTCTAATAACACCCAATTCAATACATTGTTTGTTATCAAATTTTAGTTCAGATAACTAAGAATAAGAATTTTATACTTATTGTAGGCGCTAGGCAAAATGTCAGACAAGTTTGACCAATGACAATAACGATGGTAACAAAGACATTAAATTAGACACCAGGAACAATAACAACAATTGTTCTAGTGTGAACGTAAATGATGTCGTTGTTGAGGTTGTCATGCCACCACCAGAACTCCAACCATCTCAAAAGAAGAGGAAAAATATGTCTATGATTTTTTTCATTTTGCGGTTGGACGTGGATTTAGCTTCAAACCAAACCAACGAACTGCGAACACCCCTAGAGACAACGACGCTGAGCGACAGACAGAGGCGTGGACGAACAAGAAAGACTTGCGTCGGCTGTATGAGGCGAATAGAGAAACTCAACGTTCGTCGATGAGGCAAAAAGCAACGAAAAGCGTTGACTGATGGTGGACGAACGATGAAGGAGGTGACGGCTAGGGTTTTGAGAGGAAGAAAATTGAAGAAGATGATGGCACGCTTTACGAGAGGTCATATTTAATAATAATAATAATAATTCTATTATTATTTCCCTTTTTCCTTTTCTAAATAACACACTAACCCTTTTTCT

>cucumber_newGene_84 cucumber_newGene_84.1

TCTTTTTTTTCTTCTTCTCCTTCTCCTTCTCCTAAGACAAAAAATAAAGGCTTCGTGCCCTAAAAAAAACATGACACCTCCTCATCTCCTCTTCCAAAAATAAATTATTCCTCTTCTTTAGAAAATAGAGGCTAATTTAATTCCAATTCTTCGAAAAAAAAAAAAGAGAAATCTTTTCTAACGGAAACCTTTAAGGCTTCAACTTCATCTCTTCTTTTTGCTGAAAACTCTCCAAAACAGTAGTGTTCAACGTTCCATCTTCATCAAATTTTTGGTATGTTCCAACTCCTAACTTTTGTGATCAATTTCTTAAGAGTTTCTAATTAGTTTAATTTTCTAACTATTTAGATGGCTGAGGACAGTTCAAGAAAAGATCTAGCATGGAAATATGCTCGATTGCAAAATGAGCGAGATGTTAATACGTTATTGTATGGTTTTTGTTTAAAAGCAACTAAAGGAGGAGTTTTTAGAATGAAACAACATCTCGTTGGTGGTTATAGAAAGGCCACAGCTGGTAGAAAATGCCTTGATCACGCGAAGGAAGAAATTAAAGCTTATATGTCCAAGAAAAAGGAGATTAAAGAGCAAAGAAATTTGATTGTTGATATTGATGCAAAGACTTATGGTTTTGAATATGAAGATAACGATGTGTAAGTAACCTGAATGAAAGAGTGGATCGGGTTTAAAGAGGCCAAGTCAAAAAGGTCCAATGGATGTATATTGTACACCTAACCTGAAAGTTGTTGATAATAGAAAGAATTACAAAAAAAAAAGGGAAAACAAAACACAATGATTGAAGCATGCAAAAGGAGACGAGTGAGCAAATCATCCAAAGAATTGCTCGTTGGATTTATAATGTTGGAGTGCCTCTAAATGCTTGTACTTATGATAGCTTTGCTCCCTATGAATGAGGCAATAAGACAATTCGATCCTGGATTGAAACTTCATGAATTGAGATTGCCGTGTTTGAAAAAGAAATTATAATCCACAAATAAGTTGATGAAGAGCCATAAGGTGAATGGTGAAGGTTGGATGCACTATTATGGCTGATGATGGACACAAGAAATAGGACATTAACTTTTTAGTTAATAGTCCTAAATGCACTATGTTGATTCTGTTGATGCTTCATCTTATGTGAAGGATGGAAAGAAAATGTTTGAGTTGGCTTGACAATTTCGTAAAGGTCATTGGAGAGGCCACCATGTTACCTCAACAAATGTTATGGCATAAAAAAAAGTTTGAAGTACCAACTCTTTATTAAATTTGTTAGTTATGCCTACAGATTTAAGAAGTTTAAATATGTTCGATTGTTAGAAGCAAAACAACCACAATTAATTTGCTCTCTATGTGCCGTGCATTGCTTTAGATTTGATGTTGGAAGATATGTACAAGATCCCACATATCCACAACCCATTTAAAAGAGGCATGGGTGTTAGCAATTTCATTTTTGTTTGTTCACGATTGTTAAACATGATAAGGCGCAAGACCAACTAAGAATCGTTCCGCTACAACTTGCATGACACTATCAAAGTTTACATCGCCAGAAAAACAATTTGAGAACTATGTTTATTTCGGATTAATGGAAAAACAACAAATGGAGTAAAAAACAAGGAAAACAGCTGCTCAAATAATTTTGTTCATAACTTTTTGGAATACCATCATGTTTGCTCTTAAAGAATCCAACTGTTTGGTGCGAGTACTTAGGTTGGTGGATGGCGAGAAGAAGCCTCCCGTCAAATACATTTATGAGGCTATGGATAGAGCCAAGGAGGCCATTGCAAAATCCTTTGATGGTAAGGAAGAAAAGGACATGCATTTTCACAATAATTGATCGACGGTGGGAGCTTCAGTTGCATTGTTCTTTGCATGCAATAGGATGTTATCTAAACCCAGAATTCTATTACTCCAATCCAAATATTCAAGCTGATGATGAAATTGTCAATGGTTGTATACACGTATAACAAAGATGGTTGCTTCCTTGGACATTCAAGATAAGATACTTAGAAAGCTAACCTTACAAGAAAGTTGAAGAACTATTTGGACGGGCTTTAGCAATTAGACAGAGGGACAAAATTTCTTCAGGGAAATGGTGGGATGATTTCAGGCAATCAACTCCCAACTTGCAGAAGTTCACTGTGAGAATTTTAGATCTCACTTGTAGTGCCTTGACATGAACGTAATTGGATCGTTTTTGAACAAGTGATGTTTCAAGAGCTGCTGGACCAAAAGAACTTGCCTATTATTCTAGAGTTGGTTCCTTAAGAACCAAGTCTAAGACTAAGCTTCATGTTTATCTTCATCTTCGGCACAACTACTGCTCCAACCCAGTTACTTTGGATGACTTTGAGATGGGCAAAGAAGGTATAGATGGTTGTAAGTCGATTGATGGAGAGAATGAAGACGAAAACCTATTTAGTGAAGATGACTCCTATCTTTAGGATTTTAGCATTGTATTTGTAATTGTTTTTGTGTCATTTTACTTAAGTTGGTGGTAAATGAACAGTATTTAAACCTTATTGTTGCAACATCATCGCGTCTATGGGGTTTTTTTTGAGGCGAAAGGTTTGAGGTTGCCATTTGTGATTGGTGATCAATTTTAAAAATAAATGGTCTACTTTTACCAAAGATAGGTTTGGAGGTCAATGAGTGTAGGGAAGATTTTATTCTACTATCTAAAATTATTATTAAATTCAAGAGAAA

>cucumber_newGene_86 cucumber_newGene_86.2

AATCAACGTTGGGTCGTCGAATTATAAACGACCCAAAAATTCGTTGGGGAAATGTGCTTAAGGTCCATGGAGAATTTTACTTTCTCATTATTGGGAGTGGTTAGAGCTCGTTGTTGCTAGAAATACGGCAGTACTCAAGCGAGCCTCCCTATTTAATGCTGTGATGGCTTCCCTATATACATATGATCGCAACAGTGACATTGATCGGGCTTTCTGCGAGGCATGGTGTCCTTCAACAAACACTCTTCACACCTCAGCTGGTGAGATGTCCATCTCCTTATGGGACTTATGGATACTAGGAGGACTCCCGATTAAAGGTAGATTTTATGAAGAAAGCATTCCTTGCCACCAAGACTTAATAGGATCACCTGATGTATGTCCAAGAAGTTGTGAGCATTTATTTGCAGCCTATTACCGCATTGTTTCTCAACGCATGGATCACTCTCAAATCACTGTAAGTGAATGGATCTCTTTTTGGGTTACACGATCTGAGGTAAAGTATTCGAAACCGCCTCCTCGAAAGCCTAAAAAGACTTCTCGCCCTCGCTCAACTCATAATCCAGATGGAATTCCCATCAGACGTCCTGACTGGTCTAAAGCTGAACTCAAAGTGTTCCTTGACTTAAGCGTCACTGATGATCATAGGGACAAAACCTATCTAGCGGCATTTCTCTCTTGTTGGCTATGTGTGTTTGTGTTCCCTGATAAGCAACTTTCCCTTCGTCCGGAGGTTTTTAAGGTTGCTAGCCTCATGGCGGAAGGCTACACATTTAGCCTTGCCGTCCCTGTTTTAGCTAATATTTATAGTGGCCTACGCCAAGTTCATGATTCTACTTCATCACTTGGTTATTCAAATGCCTGTTTTCCTCTTCACTATGTCCATGGGTGGCTTGCTCTTTACTTCAACACTCATTATAAAGCCCCTAGGAGTCTTAGGGGTCCTCGCATGGTTGAATTTTCTGGTGAAGGTGGGGCCAAATATTATACTAATCTTGAGGCTCGAACACATATCCACAAGGGAAAGTATGTGTCATGGCATGCGTGTCTTCCAACAAAGAATAAGGACGAACTCTTAACGGATGATGGAGAGTTAATTAGTTGGAATGCTTCATTTTTCATAAGCATTCACTCTTGTTTTCTATCCTCGCAATGTGGATCCTCCACAGTTATTGAGCCTTATAGTCCTTGTCGCTTTAGCCGACAATTTGGATTTTATCAAGACGTGCCATATGACTTAGGTTAAGAAATTCCCGAAGCCGATTTTTTAAAGGTAAGATATTGTTGGATGATTTGTATTCGCGAGAATACACTTTCCCAAGTATATCTTCCTGTGTCTGCCCCTAATCCAGACACACATGTTACCTCTCATTATAAGGTCTGGTGGCTTGCAAAACACGGTGATTACCTCCAAGAAGGAGTACAACATTTGATAGATCGTCCTACTCCCCCTCACATCAAATCCAAAACCACAAAAAAGATTGAACATAATTTTGGTAGTGGAATTCAGAAGATATGCTCCGATGAAACTGATGAACGACTTGTGGAGAAAATTGAGGGAGGGACAAAACGCTTGGTGGATAATTTGTCTCTTTCTACACGATTTAAACATCTTATTAAAGGTGGTATTGACAATGTGGGTAAGGACAACCGCTTATCGATAGCTGCCAAGCGTCCTTCTAAACGTATTGAGAACAATCAAAGCAGCAATGATGATCGCCATTGGAAGAGACCCAAGAAGCCCAACAAACAGTCGATCGATGATGAAGAGTCTCCTATTCGGGTCCCTGATGCTGCACAATTCTTTTCGTCTCCCTTTTTATTTATTTATTTATTGTATTTTCTCTCCTTTTACTCCTCTTATTTCTTGTTGTTAATATTATTTTGCAGTCTTCTTTAGGTTTTCCTTAAGCTGCCTACGTACCCTTTATAATATAGGGATCAAGTCATAACGTAGTTCAACTTTTTTTTTTTTTACCTTACAAAATTAAGCATAAAATTTTTTAAGAAATTTACCATTGATTGGGCCAATTCGTAGTCCGTCTTGATCAACGATCTTGTATGCGCCATTTGTATAAACTTCTTTAACAATGTAAGGTCCATCCCATTTAGGTGTGAACTTATTTCCTGTATGCCTTGTTGTGATGATCGGTCTCCTTACGGCAAGTACTAGATCACCGACTTGAAAGGAGCGAGGTTTAACGTGTTTATCAAAAGCTTTGGACATTCTCGCTTGATAACATTCCAATGCTTGCTGAGCCTCTAATCGCTTTTCGTCAAGTGCTTCTAATTCTTGAAGACGTAACTTCACATTATCTTCGGTAGTCAATCCCTCTTGTACTGCCATTCTTAGTGACGGAATTTCCCTTTCGAGAGGAAGGACAGCCTCCACACCGTAAACAAGCGAATATGGTGTAACCCCTGTAGGGGTGCGATGAGTCGTCCGATAAGCCCATAATGCCTCGCCGATCTTTTCTTGCCAATCCCTCTTTGACTTGGAGACAATTTTCTTTAAAAGATTACACAATGTTTTGTTGAATGCTTCTGCTAGTCCATTCGCAGCTGCGTTGTACATGGATGACTTATATTGCTTGAATTTGAATTTTTCACATAACTTGTCCATCATACTATTGGAGAATTGCTTTCCATTATCCGTCACGATTCGATGTGGAATACCGTATCGATAGATGATGTGTGTTCGAATAAAGTCTGCCACGTTCTCCTTCTTGGCTTCTCTCAATGAAATGGCCTCAGCCCATTTTGAAAAATAGTCTGTTGCTGCTAGGATATAAGAATGTCCTGCTGATGATTTTGGTGTAATGGGGCCAACCAGATCGAGTCCCCAAGCCTCAAAAGGCCAAGAAGCCACAGTTGGATGAAGAGGTTCTGGAGGTTGGTGTATGAAGTTTGCATGGTATTGACAAGGCTCACACTTCTTCACATAGTCTATTGAATCTTGGATCATCTTAGGCCAGTAGTAGCCCATTCTTCTTAGCTGGAATTGAAGCTTTGGTCCCGATTGATGTGCTCCACAAACACCTGCATGTACTTCCTTTAGAGCTTTTACCGAATCTTCCTTTCCGAGACATCGAAGGAAGAGCCCTTCAAGAGAACGGCGATATAAAGTTCCCTTGTAATAAATGAAGTGTGCTGCCCTTCTTCGTATCTCAATTTTATGACGAGAATCCTTTGGAAGCTTTCCATGTTCAAGATACTCTATGATGGGTTGACGCCAATCTTCTTCATCAATCAAATAGGATGTTGCCATGTTCACTTCCTGACATTCAGGCCTAACTGGGGGTATAATCCATCGTTGACAAAGTGGTATGTTCAGAGTTACATCATCTGGCATGGTCAAGGCCGTGGCTAAATTTGCCAATGCATCCGCTCTCTTATTTTCTACTCTAGGGACATGTTCTAACATCACATTATCAAACTTTTCCATCAATTGTCGAGCATAAGCAAAATATGGCTTTAAGTCTTCATGTTTCACGTCATATTGAAGCGAGAGTTGATTGATTATCAATTTTGAATCACCATAGACCTCTATGAATGACACTCCGATTTCTAATGCTATTTGAAGGCCAATTATCAAAGCCTGATATTCAGCCACATTGTTTGAACACAATTCGGAAAGTGCAAAGCTATAAGGCAACATATGCTTCTCAGGAGAAATGAGGACAATGCCTGCCCCCGCACCACTTCTTCGAGCTGCACCATCGAAGTACATAGTCCAAGGTTCCATAACTTCTGTGAAGAAAACCTCATCGTCTGGTAGGTCATCACATAACTTCCAATCCGAAGGAATTGGGTGGTCTGCTAAAAAGTCTGCTAGCGCTTGTCCTTTTATCGCCTTTTGGGGAATATAGACAATGTCATATTGTTGGAGTAGAACCGCCCATTTGGCTAAGCGTCCAGCGATGATTGGCCTGGATAGAACATACTTTATAGGGTCTGCTTTTGCCACTAGATGAACCGTGAAGGCCTGCATATAATGCCTCAACTTATCAATGGCAAAGAAAAGTGCAAGACACATCTTTTCGATAGGAGAATAGTTAACTTCAGCCCCAATTAATGTTCTGCTTAGATAGTAGAGAGAACGCTCCTTTCCCTTTACCTCTTCTTGTGCCAGTAATGCTCCTAAGGACCTTTCTTGTGCAGCAATGTACAATATTAGTGGTTTGTCAGGTACTGGAGCTCCCAGCACTGGGGGAGTAAGCAAGTATTTCTTTATGCTATCAAAAGCGTTCTGACAAGCTTCATCCCACACAAAATTTTCTCCTTTTCTCATCAACTTTTGAAAAGGTTGACACCGACCGGCCAAGTTAGAGATGAACCTTCGGATGTAAGCCAGTCGTCCTTGGAGACTTCTTAGGTCATGCAAACTTTTAGGTCTTGACATCTTCTGAATGGCATCAATCTTGGACTGGTCTATTTCGATCCCTCGATGCCTTACAATGAAGCCAAGAAATTTTCCTGAAGTTACACCAAACGCACACTTGAGAGGGTTCATCCTTAGCTGATATTTTCGCAAGCGATCAAACACGACTTTTAGATCCTTCAAATGGTCTTGTCGTCTCTTTGTTTTGACTACAAGATCATCAACATAACATTCAACATACCTGTGCAACATATCATCAAACACTTTCTGCATGGCACGTTGATAAGTAGCGCCGGCATTTTTCAATCCAAAGGGCATCACCTTGTAACAGTATATTCCCTTTGGAGTTCTGAAAGCTGTCATTTCTTCATCTGAGAGGGCCATCCGTATTTGATTATATCCAGACGACCCATCCATAAACGACAACGCCTCGTGTCCAGTAGTTGCATCAACCATGATTTCGGTGATGGGCAAGGGAAAATCATCTTTAGGGCATGCATTATTCAGATCGCGAAAGTCTACACAAACGCGAAGTTGTCCATTTTTTTTTCTAACAGGGACAATGTTTGCTATCCACGTTGGATATTTGACCTCGCGAATGAATCCTGCTTCGATCAACTTGTTGACTTCCACCTCGATTTGAGGGATAAGCTCCGGTCGAAAACGTCGTTGTGCTTGTTTAATCGGTCGATATCCGGGTTTAATTGCAAGATGATGGACTGCTACTTTTGGGTCAAGTCCTGGCATCTCCTTGTACGACCACGCAAAGATGTCCCTGTATTCGGTGAGCAAACTCATATATTTATCCACCTCTTCGTTAGAGAGGGACGCACTAATGAAAGTCGGACGTGGTTCCTCTATTGTACCAAGGTTCACCTCTTTTAGCTCGTCTACAGTAGATTGACCACCATCCTCTAAACTTTGTGGGGCATTTTCTGCGTCTTCTTCATGAGTTCCAGTCTCTGATTCCTCAATAATGGTGATGTGATGACATGAAGTTTCACATTCCCCTTGTTCCGACCCTTCTTTTTCAGGATTCGTTAGTATAACATCATGTCTTTTTACCTTTAACGAACCTTGACTTGTATTGAGAGTAACAAAAGTTTTTCTTTTCATGCGAGAAGGAACGTTGCTATGAATTTCTCCATTTTCCTTTGTCTCGCAAGGAAACTTCTTACTACGATAATTATCGACATCTGTATGCTTAATTCGACGCCAAACTTTCACACGAGACATTGGTTCCTTCTTCTTTGTATCTATGTTGGAATCAATGTTGTCGTGTGATCCTTTATCCCCGAGATGATTAAAAATCGGAGCACGGGGTGCTTGTACATTTTTCTTTTTAGACACACCTAGCCTTTCAAAGGCTGATGGTCTTGTAGTTGTCAAGGCATGGCATGTGCTTTCTTCCTTTATGGGAGTCGTAGTTAGCCTTCGAAAGACCGAATGTCTCTCAAGGTTTGGTACTGACTGGAGCCTTTCTCTTTCTGCCTCTGTCATGCTTAGCCTTTCAAATACTACGGGACGTGCAACAGATGGTCTAATTCGATCAAATACTGAGATTCTCTGATTATCACCTTCTTTTACGTCAGTATTGTCATCCTCCTCTATAGTTATATGATTGATGTCAACCACTTTTTCCTTCCCCTTTTTAGTTATACGGATCGGTTCTGGCGACTTGTATCCGAGTCCTTTTCTCGACACGGGTATAGAATGTCCTTCCCGTAGAAGCTTCTTTTGAGTTGAGGAGAGCTCAGGTCGGTCATGAATTTCCAAGCTTTTAAACTCGGTGTGAGCTGTGAAGTCATAACCTGCTTTCGCCATCAATTTGTAAGCCTTGGGGTCGAATCCGTCTTTCGTTCGCCTTTGGGGCAAGTTTGCTTCCACCAGATCTACCTTTACCTCCTGCTTCGCTATCTTAGTTAGTGGTGTAGTGAAGCTTTCTTTTATGATTTCGATGTCACCAACTTTCAAACCTTTTGGAGACTCCATGAATGGTGATTCGCCTTTCTTGTGCCTTGACAGAGGGACATACATGAAACCTTTTGGAGACTCCATGAATGGTGATTCGCCTTTCTTGCGCCTTGACAGAGGGACATAACGCAAAACTGGTGTGTTTGCAGCATTTTCATCCTTCAAGATCATACCCTTTGTGTTGCGAGTGTATGTTTCACCCTTTCCAGAGTTAAAGGTTCTCGCACTTTCATGTGGTTCTGTGGTTGCGAGTGATTTCAGTTGTGAATTATCTTCTCCTTTTGTAAGAGGGGTTTCTGCAGGCAAAACTTCTAAAATATTATTATTCTTTGAATAAAACTTTGCATCTGCGAAGTGAGACTCAGCTTCTGAGAATGGATTAGAGTCGGCTTCAACCTTCTTTACACCGTCCTGATAAAATTTGAAGCACTGATGCAGTGTTGAAGTTACTACTCCGTTTCCATGAATCCAAGGACGCCCTAGTAACAACTTGTAAGTAGTCCTTGAGTCTATGACATGAAACAATGCACTGGCCTTTAGGTCGCCAATTATGAGTTCTAACCGTATCATGCCTATTGCTCTTTGGCTACCTTGGTTAAAACCTTGAATTACTAGCTTGCTATTTGAGAGCTCGTCCATCAAGATGCCTAATTGCCACATAGTCGACTTTGGCATTATGTTGACAGCTGAGCCATTATCAATGAGGATTCGATCGACTCTCTGTTCTCGAACGTATCCAGAAACATACAAAGGTCTATTATGAAGTTTAGATCCCAACAACAAATCCTCATCTGAGAAGTCTATAGACATACAATAAGAACCACTCTCATATGTCATAGTCGGAGTACTCGAACTTGATGCTCTTGAATTTAGCAATGCATCAATAAGGATGGTTTTGGTCTCTTGAGGAAGTGATAATAGATCCTCTACATTAAACCTTGAAAGGTCTTTTGATACTCCCTCTCCTTCTAGTGATCTTGGAGGGATTATTTCTTCCTCCGTTGTGTTGATAGCATGACACGCAACGACTTCTGGGTCTTCATCCTGATGATCACAAAGGAAGCTTTTTGGAAGGAAGTCTGCCAAGGTCACTAAGCGTTGAGGTCGAGAGAAATTCATGTCTTCATTATGTACGAGCTTAAGCTTATGAGTCTTCTTCTTTTTCTTATTCTTTTGAGTCTTATTTCCTCTTCTATAGTTTTGGTAAGAGCGAGACTCTCTTTGGGTCGGGATCGACTGTCTTTTCTTTCGGTGGGTCACCACTATCCACCCTTCATCGTCTTCTTCGATTGGTCTTTTCTCTCCTTGAGGATCCTCGTATGAGATTTCCTGAAAGAATTGGACAACTATAGGCTCAAAGGTCCCGAACTGGACCAAGCTTTTCCTTTGCTCAAAAATCAATCTCGACGAAGAAGCCTCAGACATTATCGTCACTTCAGCGTGGTTTGTTTGAGCTACTTCCTCCAAATCTAGCTCAATCCTTTTCTCACGAGCCAACCTTAGAATTAGCTCCTTCAGCACGAAGCATTTCTCTACTGGATGACTGATGACCCGATGATACTTGCAGTAATTGGGATCATCTACCTTTCCTGCTTGCTCAGGTCGTTTACATTCTGGCAGCTGGATCAGCTGCTTCTCTAGTAGTTGCTCTAG

>cucumber_newGene_89 cucumber_newGene_89.1

GGGAATTGGAGCCTTCTTCGTGAAAAAGCCCCTTCTTCTCAAAATTTCTCTTGCCAACCTCCCTTCATGCCGCAATTTCTCTCTATCTCTCCACCGCCAACACCCACTACTCTTGCGGCACCCGGTCCAAGAGAAGAAAGACTGAGCTTTCTCTATGCGATTCCATGATCTACTTGGGAGGAATCCGTCGATGTGGTCAAGCTCACACTCAATGTGTGGAAGAAAAGAATACAAATTGAATCATCATTGGAGAGGAATGAAGTAGGGAGAATGGGTCTTAATAGAGTAAAATAAAAGTGAACAAGGTGAGACCCACTCCGATCCCAACAATAGGGCTAGGGCCCCGATCCTCTTCTTCACGAGCTCCTCCTCTTTCTACTACCCAAGTTCGCCGGAGCTATTACTGCTATGGTTTCAAGGTTTGCCTTCCAATTTCCCATTATTTAGTTATTTTCAACAATTAAATTCAATTATTTTGTAGGATGGAATTGCTATTTTCCAGAGTGGGAACTAATTAGACAAAATACTAGTTGCTGGAAAAAAGAATTGATTAGATCATTTTTCTATTTACTATTCACATGGGAAAAGCCTTCGTCTGTAGGAAAATACCCAAAATGAAGGCTTGTTGTTGATTAGATTAGATCATTTTTGTTGTTGTTGCCTTGTTTCTCCTTCTTTAGTATACAGTATATTATGTTTTGATACTTTTAAAATATATCTATCCATCGAATCTTATTTAGAAGAATTTTTACAGAAAATCAACTAACAAGGAAAGAGGTAACTCATACTTGTTATTAAAACAATCTTGATTTTTCAATGAGTTGGAATTGAAAATTTGATTTAGAAGAATTGTGGTTTCAGGTGGAGCACTTTTTGAGTTGCTCTGCCTTATCTTCAACTTGGACTGGCCCTGCCATATGACTTTTTGGTCTCCATCTACCTTCAATCATGAGATATTTTTTGAGAGAAAAAAAAGACAAAATAGAAAAAGATAGAATTTGAAATCAAGTTATTTCTATACTTGCAATGGTCTTTTCATCATTATTTTAAACTGTTACTCAAAATCATTATGAAACAAAGCAAACAAAGCAAGTTTGATTTTTGTGTTATTTAGAGTTGATAGTACAAGAAAGCAAGTTTACAAGGAAGGGGACAAAGAAGTTGTTCTCTCCATGGTCACTGTGATTAATTGTAAATATTATTTTTTTTTATCTATCCTTCTGTTTCTAAATATATGTATACCATATTATAATAACATTATGAAATATTTAACAATGTCTTCAAATTTTTAAGCAACAATTTTTGCATACGGGCAAACAAGCAGTGGAAAAACTTTCACAATGAATGGTGTCACTCAATATTCTGTGGCAGGCATTTATAGTTACATAGAGTCCATCAGAGATGTCATAGTGCCATGGAACTCTTCTCATTTCACACCACAACAAAGAACTAAATAGTATATGTTTTGGTGTAATAAAATTATCCTTATTTCTAATGCCACCGTGTTGTTGGACTAAAAGAAACATAATAAATCATTTGTTTATGTAAGATATTTATTAAGATAAATCATTTGTTGGACTAAGGATTTTGTTCGGACCCTCTTCATCTACTAGGTACTTTTCTCTCTGTCTTTCAAAACTATTAAACACTCAAAGTT

>cucumber_newGene_9 cucumber_newGene_9.1

TTTCATTATCGACTACAGATCGAGTAAAATTTGAAGTAAATTTTCACCAATTTCCATCTTGTGAGATGAAAAATCTGACATGAAATCGAAGGATTTGATGTAAATTTTGATTAAAGGTTGCTGACTACATTGCCACTGTCATGTATGGGACCAACATTCAGAATTTGGATAGGGATTCCAAGCTGTAACCAATTGGGCATTGTCTGGTAATCAAATCTGAACTAAAAACCCCATTATTTCCATATCGCATTTAAAAGAAAAATTCAGATCATCTCATCATTTATATTTTATCAGCATTTTGTTATCTTGGAAAACATTTCTTTAGCTTTATTTATTCATATTACTGAAAAAATTATAATAACGTCCATCCTGTTAGCAAATGGCTGATGTCTGCAATTTTTGTCAGTTGGATACATTTAGTCAAAAACTTGTTAGCAAATTGGGGATTTAGTGTTGAATTAGGGTTTTTTTAGTGCAATTAGGTCTGTAAGCTTGTTGTTGGTGGGATTGTGGCCAATATCGATGATGTGAGGTTTTTTTCTTACTATCCTCCCACTATTTTTCAGGGTCTAGTGGTAATCACTACATACTTTTGCTTTGCAAAGTGTATGAATAGTTCTATGCCACTCACACTGTGATAGAGCTTCAATTTTAGTTTTAAACTTTCAAACTGTTTAGTTTGTCAAACAGTTCTTTGTTTTCTTTCTTCTAGAATATTTTTTTACAAGCACAGTGATTATATAACTGGTGAAGGTTAGTGTTCTTGTTTAGATTCAAACTACTATTCACAAATCCTTTATTTTAGGATACCTAAACCATATGTTATGGATGCCATTACAACCTCACATGAATTAACAGTCGAGAAGATCTTAGGTATATAAGCAAGGAAATTAACCCCATTGGTATGATGCCTTTTAGCTAGAGTTCAAAAGCAAAAATATACAAGCCCAATTCCAAGATGAACAATATCATACCTTTACAAAAATGTGGGGACTCATTATCTTTATACGTATTCAAATATATAATCAGGAAATTATTGGATATACGTATATTTATTATGTGAGAGTTAGATGCTTAGATATATGCAACCAAGAAGATAAAACGGTTGACTATTTTGGTTATGTATCATGAAAGTATTATATAAAGGATTTTATGAGTCTGTGAAATTAGTTGAAAGGGAAAATGAGCTAAAAAGTATGGAAAAGGAATTTAAAAGAGCAGATATGGTGGTATGTCAATATCCTAGATATTTCCTTGTCGTACTTTCACGTAATTTCCTAGGTTTAAAGATGCATTCCTGAAAGGATCACATTACCTATATATATATGTTCAAATCTATTCATGATGGATCTTTTTGAAAAGATGCGGAAGAAACATTTGATAAAATGTCAATAATTGATCCAACAAACACCATATATCTTATTTGAACAGTCTAGATCTTTAAAATTGATTTAATATATATGTATATATTTTAGTTAATCCCTTTCTTTTGCCGGTGAAAATTTCTAATATTATAGATCTGTATCTGAATTCTTGCCGTACTTTAAAAGTTTGCAGTGCAACTGTTAGGTGAGAAAAACTGAACCCTTGATCAAAGTTGATGTAATCTAGGATTTGTTTACGAGTACATAGTATATTTTGGTAGCTGTAGTTCTTTTAAATATGCATTTTTTCATCGTAGTGAAGTTTTCTTCTCTACTTGTTACTTTTCCCGTAAGAATTTTCTAGTATAGTATATATTATTTGCAATTTCCCTGTTTTATCTTTGTCGAATATTAATTTTCAACAGTGAATTCCAATTGTTTACCACGGAGATCGATGAGAATTTAGGGACTTTTGAACATTGATTTCCAGCTTCTTCTTCTTCTTTTTTTTTTTTTTTTAATTTTTTTAAATGAGGTTTCTAATCATCCATAGACTGTAGAGCGTTCAATAAAGAAAGCAAGAACAAAACACGCCTAGGCTTGACCTGCTGACCCCAAGGACATGAGCTAAACTATTTGAAAAGTATGACCATTGTGCTAGTCTGTGTAATACTATAGCAATAACCACCCTTGCCCGAAATATTGAGCAACAAGGAGTAAGGACTAAGGACCGTGCAATCCTCGATGGAGCTTATTTATGATTTCGTATCGAAGTTGGTAAACAAACAAATAGGTGTTCAACGGACAACAAAGATTAATCTGGTTCGTGAAATACTTTTCTTTTGTGATCAATAATTCTGACTTTAATGAAAGTGAGAGAGCATACTACAGCTTCTCAAACGTCGTAGTCAATCACTGAAATGAAAGTCGAGAAGAACGGAAGGGGAGGGGTGAAACAACCTAACTTTTGGAACATACTACAATCTCAAGTCACAATCTTTTCTTTCATTTAAGTTTAGTATATTGGATGGAAAGAAAGTTGAAACTAGTAAAAAGACTAGTAATTCATAACACAATGTAGAGAAGGAAACTAGAGTGGGTTAAAAGTCTCGTGATCTTTTGAGTATCAAGATGACCGATAAACTAACTACAACAACTTTACTAAGTTAACGACAAAATGAGTACAATCTCAAAAGAGGGAAGCCAAACAAATGTTAGTTGGGATGTCTTAACATCTCATTAAGAACGTACAAACATCCTTTTTCAGCTATATCTGTTCTCTTCTAGCTCCCTTAGCTTAGACTACTAGATTTTAAACTTACCATAAAGAAGGATTCACATTCTCTTTTATATTTTTCTTATTAGTAATCCTTCGGGACGAGGAATGATTTTGTCCTAAGAATCTATAAGATCTAGAGATGTAATTTAACCTTTAATTGAACTTTCTTCCTAGTTTTGTCACAGCATGGTCCAGTAATAAAAACCAGTTCTCCTACTTATAAACAAAAACCTTAACCTAATATTGACCTAGAGCATAGGAAATGAAGACAAAACCTATCTACAAAATGAAAACCTTGAAAAGAAGCATTAATGTTAGGCACTCAAGAAGGGGAAGAAATCAAGAGGAGCCAAGCAAAAAGATAGATTACTCTAGGAGAAAAGAGGAGCCAAACACTTGGCAGAAAATGAAGGGTTCAAACTCAAAGAAAAGAATGGGAGTGTCCTTTTGCAGACACACAGAGAGGAGGTGTTCAAAAAAGGATCTTCGTACCAAATACATCTAACAAATTTCCCATTCAGTGGAGCACTTAAGGGAGCAGCTGAAAAAGGCCAAAATGCAATCTCTTGGAAAGTTACAAGCCTTGACCCATGTGCTTTCCTCTATTTCCATCCTCTGCTGTTCTATTATTTATCTCTTTTCTTCCTTTTTGTGGAAAACTATTACCACTATCTCTCAAAATTATATCGAACTTTTTACCATGTTCCTCTTTTTTTAACTTTTCCTCTCACCTTTTTGCCTAATGTTACTAACTTTACCATCCTCTTTTGCCCTATATTGGAGTAGGATGGATGTTTCCTTTATTGGGTCACTGAGAAAGTTTTAAACTTGAAGGCTCAAAAAGGATATTTCTTTTCCCTAACAAGAATCTCTAATCATTTTTTTTGGAACAAATTGCAAATACCACCTTCGAAGTATGATGGTAGTTGCAATCACACTCAAGTTTTTTATTGTACAAATCGATCCCCAAACTTCTACAAGTGTTAAAATTAGATCCTCAAACTTACAAAACTGATAGAATGGTATGGTCATCTCTTATTTCATGAATTAGTCGATATTATTGAACTAGAGTCAAGATTTCTAGGATTAAGTTCATTATGCCCGTCTAGAAAATAAGGAAAAGAAAAAACTAAATTAAGATTAAAATACCATTCTATCAGTTTTCTTAGACCAAGTCCAAATTTTCAAATCACAAATACTAATGATATTCTTTCCCCCTGAATTTTTTTTTCATGTTATTCTTGTTCAATTGTATGTCATCAACTACAAAATTTCATCCCTAGATACGAAGGTTTAACTTTTCAGTGAACAAATCAACAAAGAAATTTACATATTTACTTTCAGTAACAAAAGAAAAAAAAAATACAGTGACTTATACCTTCGAGATTGCCCTAACTCTATGTTGAATTCACCAATTCTTTTATGAATTCAACAGATCTTTTCAAATCTTGGGAGAAACTAGCTTCTCTAAATGAAAAGGAACATGATTTCAAAGGACCATAATGGAATAAAGGGGGGTAATGAAGGAATATGTTTTTTATTTTTAAAAATAAGGCAAAATTAGGGGAACTTCCAGGGGAGGAGCGGACATTTTTTGAGAGAAAAGAAAGTTTTAAATAATCTCAATGATTTCACAAATACTTGCATTTTAGCCTTTTAGGGCTGGCAACTTATAAAGATATGTTCCAAAAAAGCATATGCCACATATGTACATTAATTTAGACTATACCCTATGGGTTTTTTCTTCTTTTAATAATATTATTATCAATTTTAC

>cucumber_newGene_90 cucumber_newGene_90.4

CGGCAAGCCCTTCCCCTCTTCTCTTGGAGCCACCATATTCTCTCTCGATTTAGATCTCGGGAACCTTTACGAGCCATTTTGTCTCAACCTCAATCTCTCTTTCGATTGTCGACCCCCATTTTCACTTCCCTCACGCATCGGCTGCAACCCACGTCCACAGCAACAAATGATGGTTGTGACGCACTGGCTCACAACGTCGATGCTAACACTGACAACCATTCTTAATGCAACTCCACTCTGTCACGTTATCGCACAACCTCCACCTCGATATCTAAATCACAGTTGCATCGATTCTTCTCACTCAATATTCTTGTCGATAGTTGGATCTCGGAGTTTCGGTTTAAAAGTCTTTGGAGATTTTGGATACTGTCCAACCTTTGGAAAGCTTGGAATTGGGCGTTTTCACATAACTTAGGACAAATTGAAGTGGAAAAAGTGTATAATCCCCGGGATCAAGCCATTGACTGTAGCGACTCAAGACCAAGCCTTGATTGAACCCCCATTATTCTTGTAGACATTCATTTTTTATGTATATAGTTAAATTTTTGTATTACATTCATGGGGTGGTTAGTTGTATCAAGAGAGACACTAAGAACTCAAAATTTGAAGGATCTCGTATTAGGTTGTAAACAAATATGTAATACCTAGTTTGAATTTTAACTCTACTTGAAAATTTGGAACCGTCTGAATGTGTGTGGTAAGACACACTTGTTGTGTTGTAGTATCTGAAGTTTGGTTTTATGCATGTAGAAGTGAAAATTTCTCCCCTTTTCAGGTTTGG

>cucumber_newGene_91 cucumber_newGene_91.1

ATTAGCACTCCACCGGGTACCCTCTTTCGCACTGCTCCCTTCAGAAATAGGGATTATTAGCTTCCATTTGAACGTCTTCTCCAGCCGACGACCAGGGTCCATCACCGCCACTGCCCCCTCGTGGCCGTCTCCCCACTCCAACGCCGCTTGTTCCTCCTCCACTGGATGACGAAACGTTCTGTAATTTCCAATCCATCGGTTATCTGTTCAATCCTGCCATGCCCGTAATCTCCAATCTGCCACATTCTCTTCAACATTCAATCCTCATCCGTCACCTACTCTACCGCCGACGTTCCGAAGGGATATTCTCGACATGTAATGTAATGCTTAATGTCGTGGTACCATGGTTTTCTGACAAACTCTTGCTCAACGCTCATGCAATACACCGGTGCTTCACGCTTTTCAATTCTTATAGGCTGCATGTCCTCGTTGAAAGCCACATTAAACATGGAAGATAGAGTAGCCAATGCATCTACTATTTGATTACTTTCACGTGGGACATGTTTGAATGTAATTGACTTGAAAGTTTGAGTCAATTTTTGAATGTACTTGTTATAAGGAATCAATTTAGAGTCTCTTGTCTCCCATTTCCCATTGAGTTGATGTATTAGCAAGAGAAAAC

>cucumber_newGene_92 cucumber_newGene_92.1

TCTCTCTCTCTCTCGATCTGAGAATTGATCATATCATGTCAACCGAAGGAAGAAAAGATCCGACTGAAGCCGGGTCATCGTCGAGGGCGACGACGACGACGGCGGCGTTGAGTCGGTACGAATCACAAAAGCGACGTGATTGGAACACGTTCGGACAGTATCTGAAGAATCAAACGCCGGCGGTATCACTGTCGCAGTGTAACTGCAACCACGTGCTTGAATTCTTGAGGTATTTGGACCAGTTCGGAAAAACTAAGGTTCATTTACATGGCTGTGTTTTTTTCGGGCAGCCTGACCCACCTGCCCCTTGTACCTGCCCACTCCGTCAAGCATGGGGAAGTCTCGACGCCCTCATCGGCCGTCTTCGAGCTGCCTATGAAGAACACGGCGGATCGCCTGAAACAAACCCTTTCGGAAATGGAGCAATTCGTGTTTATTTAAGGGAAGTTAAGGAGTGTCAAGCTAAAGCTAGAGGAATTCCGTACAAGAAAAAGAAGAGAAAAAGAACTCAATTGAAATCCAACGATGATGATATCAACAACAACAACAACAACACCAACTCCAACAGCAAACAACCCATCAAGCATCTCCCTTGATTCAATTTATCCTGGGCAAGAAAGCAACTGATCAAGAGTGTATAAATATGGAACGAGTTGAAGGGTTTTTTTTCTATATGATAAATTAAAATACTTATGTTATTGTAGAGTTAATTATATATAAAGAAATAGAGAATTTGAATAAAAAAAATTAAATTAGCATATAGTAATGTAGCAAAAGGTAGCCTTAGCAAGCAATGTGTTTGCTCCCATTTTAGTTAATTTAGAAAAAGAAAAGAAAAAAAAAAAAAAGAGTCCCCCAACTTGTAGGTTCACTTTTTAATTATTCCATATTAGAAGGTCACAAACCAAGGTACTATACATTGTCAGTTTTACCCTTCACATTTAAGAAGAAATTTGTC

>cucumber_newGene_96 cucumber_newGene_96.1

CCAACATCTCCCCAATGTTGAGACTCCTTAGGCATGCCAGTCAGAGTCTTTTCTTTTCAACATCAAATCCTTTCTCTGTTTTTGCAATGATCACTCTGTACAAAATCCAACACCATTCGTGTTTAAATCCCAAACAACATACCGACACAAGAAGAGAGCACCCATTTGAAGAAGAAGACCCTAAACTTTCAGCCATGGCTACTCATAATCCCAGATCAAGTGTGTGACATTTGTGGTGATATTGTGTATAGGGAAGTAGTTTTCTCTTGGTCTGCCTGCTAAATAGTCTTAAACATTCAAACAATATTGGAAGAATGGAGAGCTCTTCATCAATGGATTCCAACAGTAACAACATGAACAACATGTTATTGCATGAGGGTTTATTTGAAGGATATCCCAAAATGTTGGGTTTGTGAGTCATGTGCTTGTCCATTGGGGGAGCCAAACATCATTATTTTGGATGAATTTGAGGAAGCACATACAAAAACAACAAATGTTGATGTTGCTGCATTGGCTTGTGAGCCAAACATCATTATTTTGGATGAATTTGAGTAAACATATACAAAAACAAAAAATGTTGATGTTGCTGCATCAGTTTGTGAGCCAAACATCATTATTTTGGATGAATTTGAGGAAACAGATACAAAAACAACAAATATTAATGTTGCTTCTGTACTTGGACAACAACCTCCTCCAGCCACAACCGAATCTCAAGTGAACAACGAGATGAACCAGTGGGACGTGTGGAGCTATTCAAAGAAACGCACGCTAATAAGAGTGGCCAGTTCGTGAATCAAGCAGCCAAGGATGTGCATAATCAAATGCTAGAATTACTATTGGTACCCACTTTAGAAGGTTCTCAACCACTTTCAGGCGACGAAATATGCGAAACAGTTTTGGGGAGACGACCAGGCTATTCAAAAGATCTCGATTGGGGCCCCAAACCTAAGTCAAGGAAGGATGGTAGTAGTGCATCAAATTTAGTGTCTCAAGAAATACACGATAGGGAGGTGATGGAACTAAGAAATACCGTTGAGACCTCGCAATCTCGGATTAAAGAACTTAAAGAAGGGCAAAGACGTATGATTGAAGAACAAATAAAAACGTCAGAGATGCATGCCCGACAAATAGAAAAAATGAAAAAAATGATTGAAGAAATGACAAGAGCAAAACGAGGACCTTGAACATAAGGAAAAAATGTTGATATTAAGGCAAGAAAAAGGTTCAAGTCCACCAAAGACTGATGAAGAAATCATGGTTATGGTTCTTGGAAAGAGACCATTATACATGGAAGGATTGGGATATAAATTGAAACCTCCACGAAGTAAAGAAGCATCTCTTACGGACAAGAGTATGTCGAATCTCTAGAGACTCATCTTGTAAGGACCGAAGAATTATTAGATGATCGGTGTCGAGGTTATGAAAGAAAGTTCAGCCAAATTGAAGAAATGACAATGACTCAAAAGTTATTGCAAGATCAACCACAGAACAAGGGCTTCAAAATAAGTTTAGTCAAGTCAATACAAT

>cucumber_newGene_97 cucumber_newGene_97.1

TAAAAAGAAATCATATTTGGGATTTTGATCCAGTTTAAATCACCCCAATTGAAGGGACCAAACACAAAATAAATAAAGTTTTGTAGGCCAAAACAAAATAAAAACAACAAAAAGGGTTTCTATTACTATTACTATTTAGTTTTGACCAAATCCCAAAAAAATGATAGAAATTTACCAAAAGAGGTATACTGTTTTTGTTTTTGGAAGAACTGAAGAGAAAAGGCCTTACAATCATTGCTGAAACAGCATAGACCAAACCGATAAGCTTTCAACATTCTCTGATTACACGAAAGCGATTCAAAGAAAGACAATTTGAAACGACGGATACCCAAATCAATTCAATCAAAATCATCAACATAGAAGAAGAAGAAGGAGGAGGTATTTCAATCTATGATGCAGTGTACTTGGGAGTAGAAGACGTGGTACGGTGGGAGAAATGGAACAATCGAAGATATGGTTGTTAGAAGGTAATTAGAATATGACTTCTCAATTGGCTGTTCTGTTTCAGCCCATAATATGTTTGCGGTAATGCTTGACTGAATTTACCCCCATTGTTTTAGCAGAAAGAGTGTGAGTGTTGAATATGGTGGTTGGGGTTGATAATTTAAGTCATTTTTAGCTTGCCCACTTAAGAAAATAACATCTCTCAAACTTATAGATTAAGCTATTTGATCATTGGGCCGTGTTCAAGCTTCTACCTCTAAGTTTTTGTAATTCTACTTCTTCTCTTCTTTCATGTTTTATTTTAAAGTTCACTTTCTAGTTTAATCCTCTTCCATTGGAATGTTCTGCAAATCATCTAATCAAATCCACAAATTGGCATCCAATTCTCCCTCTTCTCCATAAATTTGCAGTGCCAAGCTGGTTATATGTGTTGAGAATTTGATTCTCCTCCACCTCATTAGGGAGGCTTGGTTTTATCCATTTGCGTTTTTGTTGGTTTAATTATGTTATGTTAATGGTTCTTATAGATAAGATTGTTCAAAATGAGTAGGATAACTTTGATTCATTCTCCCAAATAATGGAGAACTCCTGATGATACTGAGGCTTGAATTGAGTAAACCCTAAAGCTTTGTTTAGTTATAGAAAGTCATTAGCAGGTGCGAACTTCTAGAGATTGGTAGGGCCAAAGAAGTAAGAAAGATGGAAGTTTGTTTTTCATATAAAAGGAAAAAGGAAAGGGAGAATGATGTGATATATTATGCAGCAATTATGAATGAAGCTTCATAACTGATATGCCTTTGTTTTTTGCAAGCTTCAGCATCCATTCACTCTCTTTTCAGTGCCCCAAATATAGGATCATATAGAAAAATAGACAATTTTAGTTACAATATTTTTATCCTTATGATATTATTATTTGACATCATAATTGTCTTCGTATTCTTATCCTTATATGAAATTATTTGGCTTTATCATTTTCTTTATGTTCTTATCCCTATATGATATTATTTGGCGTTTTGTTGATTGTTTAATCATTTTCATCTCTTTCTACTCTTTTTTCTTTATTAACCGCTCAATTATTGTGTATGATGCAAGAACTATTTGAGGAAAGAAAATCTTTATTTTGTAGGTGTAGTCCTATTTTGTTTTGGATTTGAGTGTTGTTGGAATAAATGGTTATTATTTATAACTCTTTGTATATGGAGCATCATTTCATGAAAATTGTTAATAACTAAAAGATATGATTTTTTGAATGGCTACGAGTAAGTCTAAAAATAAGTTATTTTGGTTGAGAGAAGATTTACATTATAATTCTTTGTCTTCTATATGCGGATTTCTCTTCATATTCACAATCTTGATGTTCGGTAGTTTCACAAGGTTTCGTTCGTTGATTTTCTATACGGGGTGCTACAGTTAGAACAAAGGTGATATGTTCTGTTTTGAAAGACAATTACCCTAAATACTCAAGCACTAAGGGTGAGTAAATTTGTTTTAAGGAGACAACGTGAATTCATTGGACTTAGATCTATATTTACGTATGATTTTTTGTTTATTGTAATAATAATTTTCTTAACAATTTGATAATCAGTGTAACAATCTTTAAGACAAATTTCAACTGTGCATTAACTTTAAAGGCTTGGTAATTCGCTCAGTTGAATGAAAATGTGCTTAGCTATTTTTTATTCCACACACACCTCACGTCTATGATTTGTGTCAAAAGTGAATTTTGTAATCAAGTTTATATTAACTAGTCTTCTCGAAGAATTGAAATAAT

>cucumber_newGene_273 cucumber_newGene_273.1

CGCAATCATCGCAAGGCATGGAAATCCCGCCGCAAGGACGGAAAGAAAGAAACCAAAAGAAAAAGAAAAAGAGAAGGAAAAGAACCCTAAAAATGGCAGTGTTCAGCGCATAGTCTTAAATGGTCTCCCTGTCTTCTTCGTCTTTCGATATCCATTTCATAATTGCTCGTGCCTATGGCTTCTAGCAGCTCTTCTCCCCATTCTTCTTCTCCTTCTTCTTCTTCTTCTTCAGTTGCTATTTATTCAAGTCAGTTCTCCGTTTCTTTTGGTCCGGTGTTGCTCTTTCTGTTCTGCAGGGGAGGTTTTCAAGAGGTTTAATGGCGAGGCTTATTCGAAATGCATCTTTTCTTGGGCGCTCTCTTTTTCAGCGCGAGGGCTACAGGAGAGGCATATTAGGAACTTCTACTCAAATATGTAATTTTTCAACCAAAGATGGGAGTGATTCTTCTGAAAAGAAGTTGTCTAAGAAAGAGCTGGCCTTACAACAAGCCTTGGGTCAGATAACTACCACATTTGGAAAGGGGTCGATCATGTGGCTTGGCCGTTCTGCATCATCTAAAAATGTCCCTGTGGTTTCCACAGGTTCGTTTTCTTTGGATATGGCTCTAGGAGTAGGTGGCTTTCCTAAGGGTCGTGTAATCGAGATTTATGGTCCAGAGGCATCTGGAAAGACAACTCTTGCATTGCATGTAATCGCTGAATCCCAGAAGCAAGGAGGTAGCTGTGTCTTTGTTGATGCGGAGCATGCCTTTGATCCAGCCCTCGCTCAGGCTATTGGAGTGAACACTGAGAACCTCTACCTATCTCAACCTGACTGTGGTGAACAAGCTCTTAGTTTTGTGGATTCCCAAATCCGACGTGGTTCTGTTGATGTTGTTGTTGTTAACAATGTAGCTGCTCTTGTGCCTAAAGGTGAAGTCGATGGTGAGATGGGTGATACCCCCATGGCTATGCAGGCTAGACTCATGGGCCAAGCACTTCGCAAATTGTGCCATTCTTTATTCCTATCACAGACTATATTAATATTTATTAGTCAGATAAGGTCAAAGCCATCCACTTTTGGAGGATCTGAAGTTGTTACATGTTGTGGTAATGCGGTAAAGTTTTATGCCTCGGTCCGTCTTTTTGTTTGCAGAATAGGTTATGTCAAGAAGGGGGAAGAGGTGATAGGAAGCCGCGTTCAAGTAAAAGTGGTAAAGAATAAGCTTGCTCCTCCATTTCGTATTGCTCAATTTGAGCTCGAGCGTGGAAAAGGTATATGTAAGGAGTCGGAGATTATAATTTTGGGGTTGAAATATAAATTCATGTCCCAGGCAGCTTCACTCTATCGCTTTCATGGTCGAAGCTTCTACGGGAAAGAATCCTTGAAAACTTTTCTGTTAGAGAGTGAAGATGCTAGGGAAGAACTAATTACGAAACTACGGGAGAAACTACTCGATGCTGAAATGGGTAAGCCACAGAATAGAGATGAAACAGAAGGAAGTCTTCAAGAAGATGTTATCACACCACCTAATTCTAGGGACGAAGATGCAGTTACTGCTGTAAAAGTGATGAAGAATAATAAGCATGCTCCATTTCGAAATGTTCAATTCGAGCTCAAGTCTGGAAAAGGTATAAGTAAGGAGTCAGAGATTATAGATTTGGCGTTGAAATATAAATTCATAACCAAGGCAGGTTCATTCTTTAAGTATAATGGTCGAAACTTCCATGGCAAAGAAGCCTTGAAAACTTTTCTGTCAAAGAATGAAGATGTTAGGAAAGAACTAATTACAAAACTTCAAGAATATATTATCACGCCACCTGATTCGACGGATGAAGATGCAGTTACTGCTGCAGAAGCATAAGCTTGTGGATGCCCTTCTAATTGTGAATTCAAGCACTTCCGTTCTTATTATCCTTCATCTATTCCTTCTCTCTCTTTGCCATGCACAAAATTTTGGGTCTCCTTGCCACATTTTTATATCTTTTAGTTTGTGCTTTTGTGGTTCCAATGATGGATGATGATTACCATTGTAATTTTACATGAAGGATAGAATATTGACAATGGATCTTTTTAAAGTTTGTAATACATATAATTTATTTTGTCCTCTTGACCCATATGTTCTTTT

>cucumber_newGene_274 cucumber_newGene_274.2

GAGATTTCTCCGGCGCCAACGAAACCGGCGAAATGCTGAGAAGACAACCCACCATGATCGACGTCGAAATCGAGGATAAGGAAGAACTCGAACAACTCCGTAAACGCCCCGCCGCCGCCTCCACCTACTCCTCTCTCCTCCATAACCTCAGTCGGAATAAAGACCCGGCCTCCAAAGCTCAGCGACTCGGTATTTCCTCTTGAACTTATCCATTTCATATACTTCTCTGATCTCCGATTAGTTGGCCTAGTGAACGGGAACGATAGGGGATTAGTCCGATTCTGATCGTCCGCGTTTGCGGAAAATTCTAGGAAGGCTACAGCAAATTCTGCTTCGATCAGTGAAGCTGCCTTTCTAAAGAGTGTGAAGAAGATTCTGTGGCTCACTCTGGTTTTCTTCATATTTCTATGGGCGAGAGGAATGGAAAAAGTTTCGGAGACTCTTTCTCTACTCATAATTCACGTGCAGGTGTTTATTTCATTTATCACTGAAATGTTGTTGTATGATTTAGGAAAAGTTTGTAAGCCAATAAGATAAACTACGAATGTGTATGCCAAAGAAATCAGTAGGATCAAGAAGCATGATTTGTAATTGAAATAAAACGTTGTTGTTATATGACTAAGATCTTGAGCAAAATACATTGAGGATTGGATTTAGTATCTCTTTGGTCCCTAAGGTTGGCCACTTTTAG

>cucumber_newGene_275 cucumber_newGene_275.1

CTGAAATGGAGCGCGAGCAAAGCAAGAGCTCATGGGTACCAGCAGCCGATTATTCTTCTCTCTCTCGTTCATGGACCCACATGAGCTCACAATTCTTCATAATTTTAAGCTCCAGGCTCGCCAATGTCCCACTGTAGTTTCTACTTTATCAGCACTTTTTCTGTTCGCTTTTTCCAATGCATTGTTTATTTATTATTATCTTTGATTAATACTAGCACGCGTATGGTTCTCATTGCTCAATTATGAATGAGTCTGTGGGACACTGTTCCCTTTTTACTCACCCTGCTTTTGTCTGATTCCTTCCGGACAGTTCTTTGATACGTGTTCCAAAAGAATTTTGAAGCTTCGGATTAGTTAATACGTATAGCTATAAAGGGTTGCTTTAAGCTAACCCTTTTTGTTGCTGAATCCCTTGTGATTCATTTTGTTCTCAACTCAATGCAGAACGTGTTGGTTGGGTTGTTTTTTAGTTCGTGAGTTCAAGGGGATTCTTTCTCTGTTTCTCTTTGGAAACTGAATGTTTGTTTTGTTTAAGTTGTTTTGGGAAGTGGGAAGTGTTGGAATCTTGAAGTTGTTTTCTTTTTTTGAAGCTGAAGAAAGAGTCTGGCTTACCTCATTGCTTGGATAAGTTATGGCATCTTGTTTAGGAGTTGGGGTGTCATTGTTTGGAGCTTCAGTTCAAAAGAATAGGCATTCATGGGGAGTGCGAGTAAGAAGTGAAGGTAGAGCTTCAGCTGATTCAGTTTCAGCTGTGGTGAATGGAGCTACTGTGGTCGGGGAGGAGGGAAGTTCGTCTTCAATAGATAAGGCCAATGGTTGGTTAAAGTCTAAAGCTGCAGAGAAGAAGATCAAGTTGAAAGATGATGTTCCAGAGAAGTTGGAGCTATTTTGGGATGATGGATATGGGACTGTGACTGTTAAAGATTACTTTGATACCGCCAAAGATTTTACTCAGCATCCTGATGGCGGTCCACCTCGCTGGTTTTGCCCTGTTTCATCTGGATCTCCATTAAAGGGTTCTCCAATTCTTTTGTTTTTACCAGGAATGGATGGCACTGGATGTGGCCTTATCTTGCATCATAAAGCTCTTGGGAAGGCTTTTGAAGTTCGGTGCCTACATATTCCTGTTCAAGATCGAACGCCATTTGAAGGCCTTGTGAAGCTTGTTGAAGAAACTATCAGGTCAGAGCATGCTTGTTCCCCAAACAAACCAATTTATTTAGTTGGGGACTCATTTGGAGGATGCTTAGCACTCGCAGTGGCTTCACGTAATCCTAAAATTGACCTTGTTCTCATTTTATCAAATCCAGCTACTTCTTTTGGCCGGTCTCAATTGCAACCCCTGTTTCCGTTTTTAGAAGCTATGCCTGATCTACTACATGAAACAGTTCCTTATGTTCTTAGCTTTATTATGGGTGAACCATTGAAAATGGCAACGGTCAATGTTGAAAGCAAGCTTCCCCCAGTGCAACGATTTGAAAAAGTATCACAAAATTTGACTGCTTTACTACCATATCTTTCGGATTTGGCTAGCATTATACCGAAGGATACTCTTCTTTGGAAGCTGAAGCTGCTCAAATCAGCTGCTGCGTACGCTAATTCTCGACTCCATGCTGTTAATGCGGAAGTACTTGTGCTTGCTAGTGGCAAGGATAACATGGTTCCAAGTGGTGATGAATCCCTACGACTTCGAAGATCATTGCAAAATTGTACAGTTCGCTATTTCAAGGAAAATGGGCACACCTTATTATTGGAAGATGGTATTGGTTTACTGACAGTAATTAAAGGCGCTCAAAAGTACCGCCGATCAAGAAAGTACGATACTGTTTTGGATTATCTTCCACCCAGTTTAGCAGAGTACAATTATGCTTTTACTCAAGTGACGGGACTATTTCATTTTCTCACTGGTTCTACAATGTTCTCAACTTTGGGAGATGGAACTGTAGTGAAAGGTCTTTCTGGAGTTCCTAAGGAAGGTCCCGTGTTGCTAGTAGGTTATCACAACTTATTAGGATTGGAGCTAACCCCTTTAGTTGAAGGATTCTTGAGAGAGAAGAGTATTTTGGTTCGTGGAGTAGCACATCCGGAGTTGTTCTTAGGAAACCTCGAGTCTGAATCTCCTGAAGTATCTTTGATTGACTGGGTGAAAGTATTTGGTGCAGTACCAGTCACTGCTAACAATCTATACAAATTGTTATCACAAAAGGCACACGTTCTTCTCTATCCTGGGGGTGCACGCGAGGCTCTTCACTACAAGGGTGAAGAATATAAGCTGTTCTGGCCAAATCAACAGGAATTTGTGAGAATGGCTGCTCGTTTTGGGGCTACAATTGTACCATTTGGTGCTGTTGGAGAAGACGACATAGCACAAATGCTCTTAGATTATAATGACCTCATAAAGATTCCTATGGTTAGTGACTACATCAGAGAGGCAAATCAGAATTCTGCTAAAGTGAGGGATGCGGACAAAGGAGAGGTTGGTAGCCAGAATCTTTTCCCGCCTCTGCTCTTTCCCAAAATACCCGGCCGTTTCTATTATCTGTTTGGTAAGCCAATAGTAACAAAAGGAAGAGAGGAAATCCTGAAGGACAAGAATAATGCAAACCAATTGTATGATCAGGTTAAATCTGAAGTTGAACATTGTTTAGCTTACTTGATTAAGAAGCGGAAAGAGGATCCGTATCGAAACTTTATCGACAGAACAGTGTATAAAGCCATTTATTCCTCTCAACATGAAGTTCCTACATTTGACCTATGAGAAATGATTCTTTAAGTTTAAGTTGGATACAATCATTTTATTCAAGATTGAAGAACCTTCGGAGGTTTGCTTTCAAATATTTCCATCCGAATATGTCAAGTCGTGTTCTAGAAGGAAGATATCAACTGGTACGGATTATGAGCTTGATTTTGGCTTTGTTTTCTCCCATTCTAGCTTTAGGTGCCTGTACTATAAACAATTAGGAATACAAAGAAAGTCCAAACTCTGATTAAACCACACAAAAAAGGTTACCTTGTAAATTTTTAGCTTATTTGGTTACCCATTTTTTCTAATAGATACATAGCTATTGGAAGCTTTCCTGTCTTCTGGATTTGAATATTTAGACTTTGGATGAGTTCAATGAAGAAATTGTTATTGGCCTTGTGATATCAAAAAGTAAAAATTCCGAGAAGTTGGGGTACTGTTTGGTAATTCATAATGGGATGAATTTTCAATGGAATGTAATCCAAAACTCATGTTTGGATTGAACATTTTTTGTCTGATTTGTAATGTAAAATTATTCCGTTCCGACAGTATTCAATCCAACCATTTTCC

>cucumber_newGene_276 cucumber_newGene_276.1

CGCGAAAGCCCTATCTTTCTAGATTGTTCATCGTTTCCAGACTTTCGGTTCTGTTCTCAGATCGCTCTCAAGTTTCGTCGTCTCCTCAGGCTTCGTATTTGGATTAGAACGAGCTATAGTTGATCGTAAGCTTGAGGTCTATTGGGTTGGAGTTTGGTGCATTAGGTTTTTTTCCCCCCTCTATTCTTCATTTTAGCAATTGGGGTCCTTTGCTTCTTTTTTTTTTGTTGTAATTTTTATATTTATATGATTGCTCGATAAGCTTGTAGTGTAGAACTCGAGTGGGAAACATCAATGGCGAACAAAATTATTTTTTTCGATCTTTACATCAAGATGATATTCACTGAAGTTTTATGAAGATGGAAAAAAGGCTCAACATGACATGCATTCTTTCTTTTGGAATCCAGACAAATGCTAGTCTCGTTAGTCCTTCTATTAACACTCTTCTCCAAAAAACAATTCTTTAAGCACTTGAAGTAACCTCTTTGTAATTTTTTTTTTGCTACAAGGACTTTTGATTCCATAATTTTCCAAATACGCGTTATTTTGCAATCAATGTTTGGGAACGGTGATTGTGTAGGTTTTTAGTTAAAAAAAAGTCCTCCTTTCCTTGCAAATCTTTATTTAACTTATCATAAATGTGTAGGTTTTTAGTAAGCTTGAAGTGCACTGGTTAAAAGAAAAACAAAAACAAAGAAAAGAATAAGCAAAAGAAAGCTC

>cucumber_newGene_278 cucumber_newGene_278.1

ATTCCTCACTCCTCTCTTTCTCTCTCACACACACACACACCCAAACTCTCTCTTTTCTCCCCTTCCATTTTTCCTTTCTCCCCAAACACCTCTTATTTTCCCGGGAATCTTATTATTTTCTCGGGAAAATCACCCCTCCTTCTTTCAATGCTTCTTCTTCTTCCTTAAAACACCAAACCCACAAAAACAAAGCCCCCCTCTGCTTCCATCTCCTTAAGCTACAACTAAAACAATCAGGATGGGAAACGACACCGCTTTAACTCTCGAAGAGATCAAGAATGAAACTGTTGATTTGGAGAAAATCCCCATTGAGGAAGTGTTTGAGCAGCTGAAATGTACAAGAGAAGGTTTAAGTTCTCAAGAAGGGGAGAATCGTCTCCAAATCTTTGGTCCCAACAAGCTAGAAGAGAAAAAGGAAAGCAAGATCTTGAAGTTTCTTGGGTTTATGTGGAATCCTCTGTCTTGGGTCATGGAAGCTGCAGCTATCATGGCCATTGCCTTGGCCAACGGTGGCAATCGGGGACCGGATTGGCAGGATTTCGTTGGTATCGTCTGTCTTTTGGTTATCAACTCCACCATTAGTTTCATTGAAGAGAACAATGCTGGTAATGCAGCTGCTGCTCTTATGGCTGGTCTTGCACCTAAAACCAAGGTTCTGAGGGATGGAAAATGGTGTGAACAAGATGCATCAATTCTAGTACCAGGAGATGTGATCAGTGTGAAATTGGGAGATATCATCCCAGCCGATGCTCGTCTTCTCGAAGGTGATCCTTTAAAGGTTGATCAATCTGCCTTGACCGGAGAATCACTTCCCGTGACCAAGAATCCAGGGGATGAAGTTTTCTCTGGTTCCACTTGCAAACAAGGAGAAATCGAGGCTATTGTTATTGCTACTGGTGTGCACACTTTCTTTGGTAAAGCAGCTCATCTTGTTGATAGTACTAACCAAGTTGGACACTTCCAAAAGGTTCTCACTGCCATTGGAAACTTTTGTATTTGCTCCATTGCCATTGGTATGTTGATTGAGATCCTCGTTATGTACCCGATCCAACACCGTGCCTATAGAGATGGTATCGACAACCTTTTGGTTCTCTTGATTGGAGGCATTCCAATTGCTATGCCTACTGTCTTGTCAGTGACTATGGCTATTGGTTCTCACAAGCTGTCTCAACAAGGTGCTATTACCAAGAGAATGACTGCTATCGAAGAAATGGCCGGTATGGACGTACTTTGCAGTGACAAAACTGGAACTCTGACTCTTAACAAGTTGAGTGTTGACAAGAACTTGATTGAGGTGTTTGTCAAGGGTGTTGACAAAGAACATGTCATTCTGCTTGCTGCAAGGGCATCTAGGACTGAAAACCAGGATGCAATTGATGCTGCCATGGTTGGAATGCTCGCTGACCCGAAAGAGGCAAGAGCTGGAATTAGAGAAATTCACTTCTTCCCATTTAATCCTGTGGATAAGAGAACTGCTTTGACTTACATTGATTCTAATGGAAACTGGCACCGTGCTAGCAAAGGTGCTCCTGAACAGATCTTGACTCTTTGCAACTGCAAAGAGGACTTCAAGAGGAAGGTGTTTTCTGTAATTGACAAGTTTGCCGAGCGTGGACTTCGATCTTTGGCTGTTTCTAGACAGGAAGTGCCTGAAAAGAATAAGGAAAGTCCCGGTGCTCCATGGCAATTTGTTGGTTTGTTGCCCCTTTTCGATCCTCCTAGACACGATAGTGCTGAAACCATCCGCAGGGCTCTCAACTTGGGTGTGAATGTCAAGATGATTACTGGTGATCAACTTGCTATTGCAAAGGAAACCGGTCGAAGACTTGGAATGGGAACGAACATGTATCCATCTTCTTCCTTACTCGGTCAGCACAAGGACGAAAGCATTGCTGGCCTTCCAGTTGAGGAGTTGATTGAGAAGGCTGATGGATTTGCTGGAGTGTTTCCAGAACACAAATACGAAATCGTAAAGAAGTTGCAAGAAAGAAAGCACATTTGTGGAATGACTGGAGATGGTGTGAATGATGCTCCTGCTTTGAAGAAGGCAGACATTGGTATTGCTGTTGCTGATGCTACTGATGCTGCACGAGGTGCATCTGACATTGTTCTTACCGAGCCTGGTTTGAGCGTTATCATTAGTGCCGTGTTGACCAGCAGAGCAATTTTCCAAAGGATGAAGAACTATACTATTTATGCAGTCTCCATCACTATTCGTATTGTGTTTGGTTTCATGTTGATTGCTCTCATTTGGAAATTTGATTTCTCTCCCTTCATGGTTTTGATTATTGCTATCTTGAACGATGGTACAATTATGACCATCTCAAAGGACAGGGTGAAGCCATCTCCATTACCTGACAGTTGGAAACTAAAGGAGATTTTCGCAACCGGAATAGTACTTGGTGGGTACTTGGCTTTGATGACGGTTATATTCTTTTGGTTAATGGACGGAACAAATTTCTTTTCGGATAAATTCGGTGTTAGATCCATCCGTCATAATGAAGATGAGATGATGGCTGCTTTGTACCTGCAAGTCAGTATTGTCAGCCAGGCTTTGATTTTTGTAACAAGGTCGCGTGGCTGGTCGTACGCTGAGCGACCTGGACTTCTATTGGTCGGCGCTTTCTTCATTGCACAGCTGGTTGCAACCTTGATCGCAGTATATGCGAACTGGGGATTTGCAAAGATCAAGGGAATCGGATGGGGATGGGCCGGTGTCATCTGGCTTTACAGCATTGTTTTCTATATTCCACTCGATGTCATGAAGTTCGCTATTCGATACATATTGAGCGGAAAGGCATGGCTCAACTTGTTAGAAAACAAGACCGCTTTCACCACCAAGAAAGACTATGGAAAAGAGGAAAGAGAAGCACAATGGGCACTTGCTCAAAGAACATTACATGGACTTCAACCACCCGAGTCGACAAACATCTTCTCCGAAAAGAGCAGCTACAGAGAACTCTCTGAGATCGCCGAGCAAGCTAAGAGACGTGCCGAGATTGCAAGGCTCCGAGAGCTGAACACACTCAAGGGACACGTCGAGTCTGTGGTGAAGTTGAAGGGACTCGACATCGACACCATTCAGCAGCACTACACAGTGTGAGAAAAGTATTATTGCTTCCTCATATAAAATGAGAGATTTTAATGAAATTGTGATTTCACTTTGCTGTGCCAAGGAAAGAAAAAGAGAAAATGTTTGAGAAAGAGACAACAAAACAAACATTTAAAAAAAAAAAAAGAAGGAAGTTGCTAATTTGCCAAACCTTTCCAACATACAGAGAGATTGTGTGGTGCCTTTGCCTTGTAATTTTTCCTAGTTTTTTGCTTACTATTTTTCTTCCATATGTATTTGCAATGTTAGTAAGATAAAAGAATTGTGTTTGGCTTTGACAAACTCCTAGTCCTCACCATCATCATAATGCAATTTTTAAGCATTCAAATCAAATTTTAATACTCTCCTCTTTGATCATTAATATTCAAA

>cucumber_newGene_279 cucumber_newGene_279.1

AGTCAATACAATTTAAAGCTATTTTAGTTTTTGTGCACACATCTTATGGTGGAAGAAACTTTGGGTATCTGCCTACGCAATATATGTGAGTTGCAACAAACCCACATTTTAGTTTTCCCTACAATTTGTAAGAAATTCTACACTCTTTAGTTTCTCCTACACTTTCCAAACACTCCGCCCTTTTCTTTTCCCTTTATTTTTGCCTCTTTCTAAAAACACTCCCACTATTCTCTCTATATTTTCTTCTACTATCATGCTCCATCATACCCCAATATGAAGAGAGCATCATATCCTTCCATTGCACACAGCCTGCTTGCCCACCATTATCACAAACCGCTCCTCTGCCAATTGCACGCACCTACTCGCACATCGCCACCGGATCCAAATTAGCTGTTGTTATCTCTTTATTAGCTGTTATTATCTCTTTATGTTGCACGTCTACCTATTCGCTGCCGGTTTAGTGATTTTAACTCTTTATCCAAGGTGGACGATGACATAAGTACATGACCGTATGATCATTCAATTCTACACCTCTGAAGTAGCCATTGATCACAGGCCGTATGGGAGGATGAGCACGTTGATGCTATACACGATAGGCGTTGAGAGGCGGTTCTTTGATGACAAGAAGATCCAAATCAACGTGTTTTTGACTATTTACGTGCATCTAGTTTCTATGAAGTTGCACGTATTAAATTTTTGCACTTGATTGGCACCTCATCATCACATTAGTAGAGCGATAGCATCAAGAGACACACACCTTCCATATGTTGCATGGAGAGTGTACAATCACCTTACAAGACGTAGCTATCCAGTTCAAGCTTTCCATTACCAGACGAGCACTTACTGGATCACTTAACTATGATTGGGCCAGTTGTGTTACGATCTGCTAGGAGTAAAGACAAACGATAATATTCTTAAGGGCGATCGTCTTAGCTTGCCATGGTTGGCACATCAGTTTGACGATTTTGTCCATCATTTTTACAATGCTATTGAAAACCAAATACTGCAGTATACTCGTGCTTATATCCTCACACTTATCGGTGGATTGGTATTTCCTGATAAATCCAATAGTCGGATGCACTTGATGTACCTCCCTCTACGGGAAGATCTTAACATAACTGAGACATATAGTTGGAGAGCTAGATGTCTTGCATGGTTGTATAGACAATTGTGCAACGCAATGGTTATTGATGGGAAGAACATTGAAGTGTTAGGTTTAATCTAGATCCATTCCAAGAACACGCCGACGTTGAACTTTGGGAAGTTCTTGAGAGGGCACATTTAAAGGATGTCATAGGGAGGAATACATTGGGCTTGGATGCTGAGGTATGTTTCATTCCAATCGTCTATTGTGAG

>cucumber_newGene_280 cucumber_newGene_280.2

AGCCACTCCATTTCCTCTCCATTGCCGCCGTGTTCTCCAAACGTCCGCCCCCAATTCCCCGCCGATGGAGAACATTCTGGATGAGTATGGCTATTACTGGGAAACCAATATGTTTCTCCAAACCGAAGATTTGGACAGTTGGGGATTAGATGAAGCATTTTATGGTTCTTATGATTCGAGCTCACCGGACGGAACGTTGTCATTGTCAGAGGCGTCGAAGAATATCTTATCGGAGAGGAATCGGAGGAAGAAGCTTAATGATAGGCTTCTTGCTCTCAGAGCCGTCGTCCCTAATATCACCAAAATGGATAAGGCGTCAATAATAAAAGATGCGATCGGGTACATCCAAGAACTCCGTGCAGAAGAAAATCAAATCGAAACAGAGATTTCAAATCTAGAATCAAACGTATCCAAGAGCACAACATCAAGTGAAGACGACGATCATGGAAACAACAATGGAAATACAAGAAAGAGGGGAACAAATAATTGTAATCGACAAATACGAGAGAAGCCATCGTCTTTCCCGATCGAAATTCTCGACTTGAACGTGAATTACATGGGAGAGAAGACGATGGTGGTGAGTATGACGTGTCAAAGAAGAAACAACGCCGTATTCAAAATTTGTCAAGTGATTGAATCTTTGAAGCTGAAGATTATTATGGCCAATATTACCGTCGTTGCCAATAGGCTTCTCAGTACGCTCTTCCTTGAGGTACCATTCCTTTCCTTTTTAACACCTTTTTGCTTTCTATTCAATAATCAATCATCAAAGGCATCACTATGTTATTGTTCATGATTATATGCCTGCCATAATTGGAAGAGTTGTCTATATTACAACCTCCATTCATCACTTGTCATGACACTGCTATCTAAGATGAAAAAATCTTAGGTGTTGTTTTGGATTGTCAAAAGAAACTCGATAATTCGAATAACTTTAGCTATCCAATCTAAATTATAAGAATTGAGTTTAGCTTTGGTTAAATCTTTTGAAAACTGTCCAACCATTTACTTTCCTTTTTTTCTTCCATCTTTTGTGGACTCAACCCAGGGATTGCCTAACATCCCTCAATACTTCATTTTTTCTTCCATTCACATCGAACTAGGTCCTGAGATTGAAAGAAAAAAGGAAGAGTATTAAGGAATCTCGGACAATCTCGGAGTTGAGTACAGTCGAGATTCTTCTTTGTTCCCAATCTCACTTGGCCCGACTTCCATCTAAAAAATCACTGTCCTTCAACTCGACGAAGAAAACTCTAAGATGTCCCCAGAATCTATGATCTTCTTAATCTTTCCTGTCCCTGATCTTGGTCAATCATCACTAGTATTAGCCTTGGGATTGGAAATATCTTGTCTTGCTTTCTTACCTAATTAAGTAACTACGTAGGCAAATCATAGCGATATATGCTTAAACTAATATCAAAC

>cucumber_newGene_281 cucumber_newGene_281.1

AAAAAACAAAGGTTCAATGGGAGGCATTGCTTGGACTGAAGAAGAAGATTACTTACTCAAGAAATGCATTGAACAATATGGAGAAGGGAAATGGCATCGTGTTCCTCAATTGGCTGGTTTAAACAGATGCAGAAAGAGTTGTAGACTAAGATGGTTAAATTACCTTCGTCCAAATATCAAAAGAGGAAGCTTTACACCACAAGAAGTTGACCTCATTCTTAACCTTCACAATATCCTCGGCAACAGGTGGTCAATTATTGCAGGAAGATTGCCAGGAAGAACAGCAAACGACATAAAGAACTATTGGAATTGTCATTTGAGCAAAAAGTTGAATGGTCAGGGAGTTGAGAAGCCAAATTCCACCAAAGAAGGATCCAATTTTGGGAAACAGTCGAAGTGGAAGCCACTTCAAGAAGAAAGCAGCAAATCGAAAGGAAAAGAATACGTTGATGATGATCAAAATAATGAAAGCCAAGGAATATTGGTACAAAATAATCAGAATCAAAATACACAACCAATTGTTGTTGAACAAAATAGTAGCATGAGTTTAGGAAATATGCAAATGGACTTATTCCAATTTGATCAACAAGTGCTCAAAGCAATGGAGGATGAAGATGGC

>cucumber_newGene_284 cucumber_newGene_284.1

GATTTTTAGTTGCAATCGGGGGTGAGGAATCTTTTGGTTTCGAGTGGAAAGTTTTACTGCTTTAGGAAAATTTTATAATTTGGAAATCCGATTCCAAGGTTCGAGAATCAAGCAGCAATGGCGGAGATGAGCAAATGTTTCCATTTCCAGTATCTCTAATGCTTGAACTTCAGGGAAAGAGACTATGAAGCAGCCTCTTTGCTGCATATCTCACGAGTTTCAAAGGGTTGCACTATCTCATCCTGGAAAAATAGCTGTAATTCATGCCTCCGGTGGAGTTCAACTTTTCCGCCAGTTACACGGCGCCGGCGGAGGCGGCGAAGCTGATGATTTCTTCCAGGGGCGCGCCACATCCTCTTTTCCTCCCATGTACGAAGCCGACCGGTGCTTCACTTACTCGCAGCTGTTGGCCTCCGTTGATTCTCTCAGCTCCCGCCTTCTTGCAACCGTTCGCGGTCCTCAATTAAACGCGCCTACTGCTCCTCGACCAGCGAATGATCAGCCGGCAAAGACTAGTCCAGTGGCTAGTGAATTGTCTGAAGCATCAACTGAGCTTGAGAGCAGCAATATACCGAAAATATTTGGAATATATATGCCTCCTTCAGTTGAATACATAATTTCTGTTCTTTCTGTACTGAGATGCGGAGGGGCTTTTATGCCGTTAGATCCTGCATGGCCTAAAAGGAGGATTCTGTCAGTTGTTTCTTCATTGAAAATTGATCTTATTATCTACTCTGGGTCTTCATTTTGTGTAGATGGGTATCATGTGACTGAGGGATTTCGTTGGCTCGAGGAAATCAGTGGTTATTCAACCCTTTGTTTTAATATGGAAGAAAGTTCTGTTCGAGAGCATAATAGTGCAGTTGATTTAGTTTTTCCTTGTGAGGATGAGAAAGCGAGGTTGTTTTGTTATGTTATGTATACATCTGGATCTACTGGAAAGCCTAAAGGCATATGTGGCACCGAACAAGGTCTTCTAAATCGCTTTCAATGGATGCAAGAAAATTTTCCTTCAACCAGAGAGGAACTTTTATTATTCAAGACATCAATTAGCTTTATTGATCACATTCAAGAATTTCTTAGTGCCATATTAACAGCTTCTGTCTTGGTTACACCTCCAATGAAAGAGCTGAAAGAAAATTTATGTTCTGTTGTCAATTTTATTCAGGCGTATTCTATTAGTAAGCTTACTGCTGTTCCATCACTAATGAGGACGCTTCTTCCTGCATTGCAAAGATTCTGTGGGGTCAAATGTTCCCTGAGATTGTTAATTCTGAGTGGTGAAACTCTGCCAATACTGCTATGGGATGCACTTGTCAAGCTATTACCAGAAACCACTATTTTGAATTTGTATGGTAGTACAGAGGTATCTGGTGATTGTACATATTTCGATTGCAAGAAAATGCCAATGATTTTGGAGACAGACGCAATCAAGACTGTTCCAATTGGTGTGCCGATTTCTCACTGTGATGTTGTGGTTGTTGGTGACAATGATGCACTGAACCTGGGAGAACTATGTGTTGGTGGTCCCTGTGTATGCAGTGGATATTATTCAGATTCTGTTTTTCTCCCTTTGGATGGTATAAAGTTTTCTCAGGACTTTATTCATGAAGGTTCATTCAATGTAACGTGTAGTCAAATTTATATCAGGACTGGTGATTTTGTCCAACAGCTTCGAAGTGGTGACTTGGTGTTCTTGGGGAGAAAAGATCGTATTATCAAAGTTAATGGGCAACGTATTTCTTTAGAAGAGATTGAGGATGCTTTAAGGGAACATCCGGATGTAGTAGATGCAGCTGTTGTTTCCAGGAAAAGTGATTGGGAACTTGAGTATCTAGTGGCGTTTCTAGTTTTAAAAGATAACGAGAAAAGTGAAGTATTCAGATCCACTGTCAGAAGTTGGATGGTGGAAAAAGTTCCATTGGCTATGATTCCAAATAGCTTTTTCTTCACTGACTCAATTCCTATGACAACCAGTGGAAAAGTTGATTATGAGATCTTGACACATTCAAGACCTCTTTGGGAGCAGGTGCATGAAAGTATTGATGAAACTTGGGCAAATGAATTCATTCAAATCATAAAAAAGGCCTTTTCCGATGCTTTAATGGTTGAAGAGATCTCCAGTGGTGACGACTTCTTTACAATGGGTGGTAACTCTATAACTGCAGCGCATGTTTCACATAGATTAGGGATAGATATGAGATGGCTGTATCACTATCCAAGTCCAGCTAAGCTTCTTACGGTTATTCTAGAGAAGAAAGGATTGGATATCATACGTATCAATGAGGATGCTGACTCAAGAAGGAACCTGAAAACTGATAGGTGGAACAAATATTCTTTGGATGATTCTGAGTTTCTGAACCATTTTGATCTTAAAGAGGGTGGGAGTTCTGGAAAAAGGAAACAAGTTCAACCAAATGGTGATTTTTCAAGGGCAGTTGTACCAAGGAATAACAATTCTTTATTGTCGAAACACTATAAGGCGGTTTCCGACTGTTCTATCAATTTGGAAAATATAAGTCAAGTTGGTGGACACCTGTGGCATTCTCCTTTAACATCTGTATCATGTGCATTCAGTCGATGCAACAAGGTTGTGTATGAACGCAAGTATATTGGTGATAATAAACGTGCAGGAACTTTGTTGGTAAAGTCCCCAAGAGGTGAAAATGGGTCTATGAAAAAACTATGGCAAGTTCATATGGAATCTTGCGTGGATGCTTCACCACTTCTTGTGTTTAAACACCCCAATATCTACTTGTTTATTGGTTCTCACTCACATAAGTTTGTCTGCGTGGATGCAAAAAATGCTTCTCTTCGTTGGGAGATACGGCTAGAAGGAAGAATTGAATGTTCCGCAGCAATTGTTGGGGACTTTTCTCAGGTTGTTGTCGGATGCTACAAAGGGAATATATATTTTCTCGAGTTTTCCACTGGCGTTATCCTATGGACATTCCAAACATATGGTGAGGTAAAGTCGCAGCCAGTGGTTGACCCAGATAGAAATTTGATCTGGTGTGGATCGTATGACCATAACTTATATGCACTTGACTACGTGAGGCATTCTTGTGTTTATAAACTTCCATGTGGAGGAAGTTTATATGGATCACCTGCAATTGATGGGGTTCAACATAGACTTTATGTGGCTTCAACCGGTGGACGAATAAGTGCTCTATTGATAAAGGATTTCCCTTTCAATTCTTTGTGGCATTATGATTTAGAAGCGCCAGTCTTTGGGTCCCTTGCAATTGATCCAGTTACTAGAAATGTTATTTGTTGCCTGGTGGATGGCCATGTTGTTGCGTTGGATTCAAGTGGATCTGTTTCATGGAAGAGTAAAACTGGAGGCCCTATATTTGCTGGACCCTGCATATCTACTTCCATTCCTTCGCAGGTTCTCATATGTTCCAGGAATGGAAGCATTTATTCTTTTGAACTGGAAAGTGGAGATTTAGTGTGGGAGTACAACATTGGCAATCCGATAACTGCATCTGCTTGTGTTGATGAGCATCTGCAACTTGTACCTGAAACTTCCATCTCCTCGGACAGGTTGATTTGTGTTTGCTC

>cucumber_newGene_287 cucumber_newGene_287.4

CTCTGACTCACCCATCGCCGGCGTTCATCTCCGATCGTCTCTCCTTCTCAGAAGTCTCTGATCGTCGCTCGCGCTTGGCTCTCCCTCATCTTGCGTTGCATTGTTGTGTTGCCGTCGGCTACTATACAACTTATCTCGAGGACAAACTAATCGGATTATTTTTCTCAAGGTAAATAACTTTGTTTCCACATTGCTTGAGTGAGTAAATATTTTTGCTCAATTTAGAACTTTTGGACCATATTTGAACAAGATTTATTCTACAACTTAACCGACTTCTTAGAATTGTTGAATGACATTGTCTTTGCTTTGGTAGTTTTATCATTTCATTCCACTTGGTTTAAGTGAAAATGAGAGGGTTTCACTCCTTCTCATATTGGTCTTCTTCGTTTGTCTTCGAATCTTACTGTTGTCGGATTGTGAATACTATTAGACAGTTAATATGTTACGAATTAGTTATCAGTTTTCTTCAACTTCCAGCTTGTTAAAGTAGTTTCATTTCTGTTACAACCATTGTTAGAACTCTCATGTACAGACCTGTATAAATACCCTCACTTGTTCATAAATAAAATGAGTGAAAGCTTATTCAAAACTCTGTTCAATATGGTATCAGAGCACACCCTTTCCTCCAAAATTTTCTGGTGGCCGAATTCTGTTCATACTCTGCCACTAAAACCTTCCGCTGCAAGAATGACGACTCCGACCATCAATGTGTCACACACCTCGCCGAAAAACCAAGAAAATCCCAATTCAAATCAGTCTGATACCTCCGCCCAAACCTCTTTCGATCAAAACCAAGGATATTTGAATCCTTATTTCCTTCATCACAACGATAATACAAACTTAGTACTTGTCACGGAGCAGTTGACTGAGGAGAATTACGTCTCTTGGAGCCGAGCAATGACCATTGGACTCTCTGTGAAGAATAAGATTGGTTTCGTTGATGGAACTATCGCACGACCAACTGGAGATCTCCTTCCAGTCTGGATCAGAAATAACAATATTGTTATTTCTTGGATACTAAACTCAGTCTCCAAACCCATCTCAGCCAATATTCTCTTCTCAGATTTGGCAAGAACAATATGGGTAGAGCTCAAGGAAAGATTCCAAAAGAAGAATGCCCCAAGGATATTTCAATTGAAACGATCCCTGGCAACACTATCACAAAACCAAGACTCCATTGGTATGTACTTTACTAAGTTCAAAACTTTGTTTGATGAATTAAATACATACAGACCAGCCTGCAACTGTGGAACTTGTACTTGTGATGGCCTAAAAGAAATGGCAGACTTCCTTCAGATGGAGTATCTCGTGGATTTCTTATGGGACTCAATGAGAATTTTTTCCAGGCACAAGCTCAACTCCTACTTATGGATCCTCTTCCATCTACAAGCCGAGCCTATTCTCTTCTCCTAAAAGAAGAACAACAAAGATCAATCAGATCTTTTGCCTCCTCAACATCTGCAATGGCATTCGCATAGACAAATGCTACAAAATCCATGGATATCCTCCTGGATATAGAACAAAACAGCAACAGCAGAGAAATAATAATGCTGTTAATTCAGTAACAATTCAGAATGATGAGATTGCACCTCAAGGGACCACGGAACTCACTTCAAATCCAAAGATTAACAACACTGCAGAAGCCTTGATACAGTGTCAGAATCTTCTTAATCAACTTCAGTGCCAAATAAAATGCTTCCAGTCAACAAACTGCCTGTCATGTAGCAGGACAAGTTCACTTTGAAGAAGATTGGCAGTGCTGAACTTATATACGGTCTCTATGTCTTCAGTTTTGCAAAAGACTCCACTGTGATTTACCTGTCTATGCTGTAACTAATAATGAAGCTTCTTTGTGGCATCAAAGACTTGCTCATCCATCTTTTGATACATTGAAATCTCTGCAAAATTTGTTGCATTTGAAATCTTTTTATTCTCATCATTGTACTACTTGTCCATTAGCCAAACAAAGAAGACTCTCCTTTTCATCAAATAATCATTTGTCTTGCAATGCTTTTGATCTTATCCACACTGATATTTGGGGCCCTTTATCTACTGCCACTCATGAAGGTTATACTTATTTTCTTACCATAGTGGATGACGCTACTCGTTTTACATGGGTCTTCATACTTAAACAAAAGTCTGATGTCAAACTTGTCATACCTCAGTTTTTTAAGCTTATTGAAACCCAATATGGTAAAACAGTCAAGCAAGTGCATTCTGATAATGACCCTGAACTCATGTTCACTGATTTTTTTAAAGAAAAAGGAGTGTTGCATCAATTCTCTTGTGTTAGTTGTCCTCAACAGAACTCTGTGGTTGAAAGAAAGCATCAACACATACTCAATACAACCCGAACACTATTCTTCCAATCAGGTATCCCTTTAACCTTTTGGGGAGAATGCATACTGACTGCTGTATTTTTTATCAACAGAACTCCTTCGAAGTTGCTAAGTTGGAAGTCTCCATTCCAATTGCTTAACAACAACTTGCCCAACTATCAACCTCTAAAGGTATTCGGATGTCTATGCTATGCTTCCACTTCCACACAATCGCTCTAAATTTCAACCAAGAGCAATACCGGCTGTTCTCATAGGCTATCCACAAGGCATGAAGGCATACAAACTATAAAACATTGACCAAAAGAAGTTTTTT

>cucumber_newGene_288 cucumber_newGene_288.3

AAAAAACTTAAAGTTAAACCGTTATCTAAGGAGATCTTAATGTTATTTTGTTTTAAGATCTACATTTATAGAAGATTCTAAGGTTGGATTCTCATCTCAAAGTTTTCTATTCTTGACATTTCTTGCTGATTTTTGTAGCTTGTTCATATTACGAGTTATTAATGAATTTATTAGAGCACTAGTTAAAGGTATATATGTAAGATTGGTATGGTATTGTATGGTATTAGTAAGGGGTATTATGGGTAATTATGTAGTAGTGTCTTGGTTATAAACTTATAAAAACAAGGGGAGTTGTGGACCTTAGAGGGTTTGTGGATGGTTTTGGTGGGAGTTTCCATTGGGAGAAAGATAGCCCTCTCGAAAGACTATCCGTGTATTGTAACCATGCTTGATATTGCAATATACTACTATCTTTGTGCTCTTATTGATTTCTTTCTTTTGAGTTTTTTCGTGAGTTGGGATTCGAACAGATTTTTTTTGTACTTGGATGAATAGGGTTGCGATGGTTTGCATTTTTTCCCCTTCCAAATGTTCAAGGATAAACTTGTGGCCCCTCATCTTGGACATGATTTCAACACGTTGTGCACTGTGAATGTGGAAGAACCATCACCACCTCCTGAACCGAGTAAGTTTTCATTGTTTTGTTGGCTCTTTTGGCTATGGAATGATGATAAACAACCTCTAACAGATGAATTGGAATGATGCTTACTAACTAATTCAATCTTTGTTGTTGAAATTGTTTTCTTAATGTGTATATCATCAACAAGCGCAGACGCTGAGTTCACATTTTATTAAATGTAGGCAAAAGTCACTCATTTTTTTTGTACTAATTTCAAGTTTTGTTTTAACCTCGTCTCGAGCTTCCATTTGTTTGACATCAAAGATTTAGACTCCAATATGATTATGGCTTTTGCTTGATGTTTTACCACAAAAGGCAAGTGCTTCATTTTTT

>cucumber_newGene_290 cucumber_newGene_290.1

CTGCAAATGACCCACCTCCTGGTTCGTCTTCCCCATTACTCTCCCATTCGTGCCCCACTACCTCCTCGTTCGTCACCATCCATCTAAGTCATATTCATGTTCAATGAAAATATTGAGCGAAGAGGAACAAGAGCCAGTAAGTTCATCCATGGCTTCTCTATGGTGGAGATGGAGACTTTAACCTTCGTCTGTTTTTAAATTAAATTAATTTTCAAAAGGTTACAAATTTTTTTTTAAAAAAACAAAACCCATCTTTTTCCTAAATGTCTTCCCTTTCCTTCTTTCAATTTCATCCCCAAATGCCTTTCCTTTCTCCTCCCAACACCATCCCTGACTTTCTCCATCACATTTCCTCTCTTTCCAATCCCTTAGATAGGGCTTCTAGACTCCATTCTCTTATCACCAACTTGGAGGATGAAATGAAGATGATTGATGCCTTCAAGCGCGAGCTTCCCTTGTGTATGCTTCTCTTGAATGATGGGCCTCAAGTTTAGCGAAAGGTCTTGGCAATCCAACTTCAGTGCGAGGTTGAGCCTGTTTCTGCTGGGGTTCAGTATGATAGAGAGTTCATTGTGTGCTGTCTTGATCTTCTTTCAGGACTCGCAGAAGGTCTTGGCAGTGGAATTGAAAGCTTGGTTTCTCAAAGCAATTTGAGGGACTTGCTCTTGCAATGCTGCATGGATGAAGCTTCTGATGTCCGACAGAGTGCCTTTGCATTGCTTGGGGATCTTGGAAGAGTAAGCATTTGGATTGATCTCTTAGATTGTTTTTCCTTTGCTTCTTTTTGATTTAGTAGGTTATTCAAAAATCTTAATTGCAACGAGCCTCTCAGCATTAATCATGCTTGCCTATTTTCCCTTGAAAAAGTAATGAATTGTTCTTCAGTGGAGGGATGAAGCACATGATTTATACGCAAGTCTATGCTGACTACTAGGAACTTCTGTTTCTTGATATTTTTCTTGTGTTTATGGTTTATTTATATTATGTGGTCAGGTATGCCACGTCCATTTGCAACTGCTTTTATCAGAATTTCTTACCGCTGCAGCAAAGCAACTAGTAATTAAGCTATTTTATGGCTTACATTTCTAATTTGAGGTTATTTTACTGGTCTTATATGAGTAATTTGCACAGGATGCTCCTAAGCTGAAGGAAATTGTTTATGTGGCAAACAATTTCTGCTGGGCAATTGGAGAATTAGCTGTTAAGGTATGTGTAAACTTTTCGTACACTCACAGAGTGTCATATATGAGCTTGTATGGATACAGGTTTTCACACATCCCTTTTATTGTAACTATTTAAGTTTATGTTTGGCTTTGGGATGAAGAACATGTATTAGAAAAAAAGTTTTTTCTTTCTTCTTTCTCTCCCTCTCTTCTCATGCAAGTAAATTTTTGTATTGATGTAATGCATCCGATGGTAAAATGGCCTCAACCTGAGAATTCAACACTATTCCCTGCTATAATGTTAAAGAAAAGGAATTATTTCAGATCAATAGCTTAAAGAAAAGCCAGAACTTCAACCAGAGGGTTTTTTTATCATATTATCTTTAAAATGTTGATATGATATTAACTTTACCTTCACCTATTAGATTTAGCTTTTGATATTTTGCAACGTTATTTTCTCTACAATTAATATTGATTCACTTGTTGGGGTTTTTAAAAATTTCCAAGCCTACAAGTTGGGGAATCTAAAAGTGTCGATATAGTATTAAACTTATATAACTTATATACCTTTATCCATTAGCTTAAGCTTTTGGGTTAATAAGTGGTTTGAATTTAAAATTTCTAGAATTGAGCTATGGTTATAATTATATAAATAGAAATAAATTTTCTTCTTGCCAGCAAGGTACTGGATAATTTATGATCTGCTACATGGGTAGTCAGTCTACTTTTCGTTATTGCATGGTTAGAAATTTTAATTCAGTTTGTTCTTAGGATTTCAAACTCTTGAACTCAATCATTTAAGCACAACTTTACTCATCTTGACATATCAAGAGACTTAGCTGAATAATTCTACCAAAGAGGTATGATTAAGAAATTATCTTTGCACTTGTTTAGAAATTAAACCACTCTCTGTTATCTCAGCAATATATAGTATACCAAGTCAACAAGATTCATTTGCAGGTACTTTATATTTTGTTAGACAAAGTTTCTAAATAGTTTGTATATGGAAGACCTTTTATTGGTTCCTCTTTCCATTGCAAGTTTGAAGAGATTGAAGATGTTAGAAACTTAGACATATTTACTTGTATTATAGAGATTTACTTAGACAAGATTTGTTTTATAGCTTGTACAACTATCTCGTTG

>cucumber_newGene_292 cucumber_newGene_292.1

CTGAATTAGGTCTTTGATTCAATTCTTTGAAGTGTTCATTATCTAATTCAGTATGTACTGATGATTGTTCAAATCATTCGTACTATAATCTTAGGAATGTTCAATTTGCTAAGATGTTGAGAAATTAGGGCAGGGCAATGTCTTCGATAATGCAAGTCAGGTTGAGGCAATACAGCAGGTTGGTGTTGTGATAACTGATATTTGCTGTTGGTCGTGAACAGATAGCCGATTAAGATAAGATCATATCTGCTGTCAAACTAGCTGGTAATGTTAAGCTCATCGTAACAACTCCGATCGGGTTTCATTCATTTCAATGGCTCAGAATCTCAAGGTTCTCGTCATAGGAGCTACTGGTTACATCGGAAAGTTCGTAGTAGAAGCCAGTGCCCAAGCAGGTCATCCAACTTTTGCATTGGTCCGCCAATCTACGCTCGCCGACTCCGCCAAGTCTTCTATCATCCATAATTTCAGGAACTTAGGTGTCAATTTCGTCTTCGGTGATATCTTCGACAATGAGAGTTTGGTTAGGGCAATACAGCAGGTTGATGTGGTGATATCCACCGTTGGTCGTGGTTTGTTATCCCATCAAGAAAAGATCATATCTGCTATCAAACAAGCTGGTAATGTTAAGAGATTTCTTCCCTCTGAGTTTGGCAATGATGTGGATCGTGTTCACGCCGTGGAGCCAGCAAAGTCGATGTTTGCCAGTAAGGTTGAAATTCGTAGGGCTGTTGAGGCAGAGGGAATCCCCCATACATTTGTAGTTAGCAATTTCTTCGATGGTTACTATCTTCGCAACTTCTCTCAGCCAGGAGCTACCGAACCACCTAGAGACAAAATCAAAATCTTTGGAGATGGAAATCTCAAAGTAATTTACAACAAGGAAGAAGACATTGGGACATACACCATCCGAGCCATAGATGATCCAAGAACCTTCAACAAGATCTTGTACATTAGACCTCCAGCCAACATCTACTCGACGAACGAGCTAGTCTCACTATGGGAGAAAAAAATTGGCAGGATTCTTGAAAGAACCTATGTCTCAGAGGAGGAGCTTGTGAAGAACATCCAAGAGACTCCAGTTCCACTTAGTACAGCATTAGCAATTAGTCATAGTGCTTTTGTGAAGGGAGATCATACCAACTTTGAGATTGAACCATCAATTGGAGTGGAAGCTTCAGAGCTGTACCCAAATGTTCATTACACAACTGTAGAAGACTACCTCAATCAATTTGTTTAGAAAGCAAACAGCCAATTTCATCTTTGCTTTGTTTGGATCTGTGTCCCAATACTTGTATGATGCAAAAAGAAAAAAAAACGGGATTAAAAATCACATTTTTAGTCCTATACTTGTATAGAAACAACAATTTGATTTCTAAACATTGGCTTTGTAACAATTTACTCTCTATACTTTTA

>cucumber_newGene_294 cucumber_newGene_294.1

AAAATTTTAACTTTTATTTAACTAATCCTTGAAATTTATTCAAAGATATGATTTATGATAACTGTTTTCTGAAATGATGTTTCAAGCTGTACTGTGTTTGAATGATTTACTGATGAAAGGTGTGGTGCTCAGATATATTCATGTAACTTTGAATGAGAACGTAAATGATAACCCCAAGGAATTTTGGTGAATTTAAATTCAAGAAGTGGCACAGTGTTCTTACAAGACCATGGACTGTCTATTTCTATTTTATGATTACAGCTCTACGTATCTCCTTTCCACTAACAATTTACATGGAGAAACTACTCTACATTGATGCTCTACTCTCAAGTCTCAACTATGGCAGAAAAATGTACTCTGGCAAAACTGTGTGGAAGCTTGCTCTTTTCTTCTCTTTCAGGAAGTTTGCAGCCAGGATGTGAGATTGATGAAGACATCTTCTCTACAAGGAATTGTCAAACCTCCAGTTGGATGTTTAAATCCAAATTCTTCTTCAGCACTCTTGAGCAAAGCTGAAAAGGAAGGATGGTTTAAGTATGATATTGGAACCACAAACCGTTTTCTTTCAATATCTCCCACATAGACTGCAATATGGCCTTTGGGAACACCACATTGATTTCTAGAAGAAACATTATGCATTTTGACAAATTGCTTAGCGTTGAGGAGAAGTGAAGGTAGTCTGATTCCCATGATTTCTTTTGAAAGGGTTTCTCCTTGATGCAGATCTAGTAGTTGGGAAGTGTTGTGCTTGAATGAGGATGTGTTTGAAATTTGAGTTGGAGATTGAAGTTTATTGGATCTGTATTTATGGGTTCAGAATTGAGAAGTGATGGGAAGAAAACAGTGTAATTGGGTGTCTAAGAGGGCCTACTTCATCAATGCTCACATGGGATGCTTATTCGGTTTGAATACACACACTTCATCATGTGCTCTAACTACTGGTGAGCAGTCCATTCGTTCCAATTTTGTACAGTTATAGGCCAAGATAAGAGGCCAATAAAACTTCTAAACCAAATTTGATGTCGTGGAAGATCAACTGATGACAAAGTCATGTGACAAACTTCCATCATCCGTCCCTGCCTGCATTTCTAATCTGACTGGAGAAGAATTGTATACTGGAGGGTACCAAACTGGAATAAATTATCGTTGAAAAAGACTAATCAATAAATGGATTCCAGCTCACTTGTAAAATTTTCTTTCAGACAAGAATAATATTGGTCTCCAGCATGCTATAATCTTAGATGTTTACCATTTAGGATTGTCTAATACATTTTCGCTTATGTGTGATATGAGATGCGTGTAATGTTTGTCCATAGGTCTGACGCGTCTATTACATGCAGTCAAATTCCTTTCCTTACTCATTAACTATCTTTGTCTAAATTGGTCTATGCATGTCCCTTTTCTCCACTCATTTCGTTTTCTTGATATCTTTTGTTAGAAGCACTTGTGCTGATACTATATAAAGAGCCAATAAGATTTTATATGCAAGCTGCATATTTTCTGATAAATTCTTGTATTTTACGTTTTTCCTCCTAATGGCATACCAGAAGATCGGGTGGCTTCTCCAGTTTATGTTACTTTGAATGCAGACTTTAAATGCGACTTCCATTCTTCAGAAGGAGTAGTGCTTCAAGTCTATGGAAGTGAGCAAGAGTTGTTGGGGAAGATGGGAGGCAGTTTTTCATTCTTGAACCAGCTGCTCTTTTAGGATATGGTCGCTCATTCAGGAATTAAGCCAAGATATGGAATGATTTAGAAATTTACATAGCAGGAATCTCAAAGTTTCAAACTAGGAACAACAGACTTGAAAGTGGAGCATGATTCTACCATGGATTAAGGTTGATTTTGACAGTGACCTGCTCTTGAAGGTTGACACAATTACTTGGGTCCAGGGAAACAGAGATCGGCTTACTGTTTTATTTGCTTTAACGCTGAAAAGTTACATATGCTTTTTCTGAGATTTTGTCAATTTGTTCAGAGGTCAGTATTCATAGTTGATGGATAAAGCCGAAAAACTGTATGCGAGATAGGTAGACATTCTTGAATTGAGAAAAAACTGAAGTTGGATGGTTTTCATTTTGAGCTCCTAACGATCAGTATGGAGGACATGTTTACTGAGCATTCTCAAGTCTTAAATAGTAGCAAACACTACTAGGAAATCTTTATCGCAAGAGCTTGATTTCAACAAATTCAGTGACAAAAAGGCAAGATTAAGTTGTGGCAAATGATTGTAGGATTCTAGATGAAGCATCAAAATAATTTTCTATCTAAAAAGGAATGTGATCTTGATATTATATTATACAATTCTCAAAGGAACGGAACATAATTCAATGCAGTTGCTTATTAAGAAGAAACAAGAGCAAACTACTGATCGCATATGAAAATGACCAATTTGTTATCTAATATCTTCTTGTCCATTCCCATCCGTAGGTTCAAAAACTTCACAAGATATGTTGTCTGGTGTAAAACGTGATATATTATATCTGACAGAATTTGGATGTTCGGCTTATGCTCGCAATAAACAAGATAAGTGACCATGGAGCTGAAAAGATGCGTCTACCTGGGGATCGGGTGATGTCAAAGAAAAAGCTGTGGGGTTTTTTGCGACTGTGCCATGACACCATGTTGACCAAAGAGTTCTTTGTATGGATTGCAGCAATCA

>cucumber_newGene_295 cucumber_newGene_295.1

AAAAAAGTAATGATAAAATAAAATTGGAAAGAATAAAAGAAAAGAAGAAGAATCAACAATAGCCCTAATTTTGAGTTTGATTTTAGTACTCATCTTCTCTCTCTGGGTGTCAGATTCAAGCTTTTCATATGCAAGATTCCGCCATTGGAGCCCGACTTACTGACGTTCTCATCAAGTTGGTCTCCTCTATTTCTCAACCTCCATTTCCATCTCTCCATTCTCTCTTTTGCACCTTCTTTCCTTTCTCCTCTTCCGACTGTTATTCCGTTGCTTGCTCCACTGTTATGCAAAGAATCCGAATAAATCATCCATAAAAGGGTAGCCAGCCAGTCAAGGGTTTTTTTCATCTGCGCACCCGGGTCAGCCTTTTCCTTTTCAGGAGTTATAAATTGGTTTCTAGAACAGTTCATTTGACTCAGTTTTGAAGTCTTTGAGAAACTTCCATGGCTGATAAGTTGGTGCGAAAACAAAGTAAATTGTCTGTGTACCTTTACGTTCCCAATATTATTGGTTATATAAGGGTTCTCATGAACATTGTTGCCTTTGCACTATGCTTCTCAAATAAAATAATTTTCTCAGTTCTCTATTTTGTCAGCTTTGTATGCGATGGTGTAGATGGGTGGTGTGCTCGCAAATTCAATCAAGTTTCGACGTTTGGGGCTGTGCTGGACATGGTAACAGACAGGATTAGCACTGCTTGTCTACTAGTAATTCTATCCCAAGTGTACAGGCCTGGTTTGACTTTCGTATCATTGCTTGCTTTGGATATTGCTAGCCACTGGCTTCAAATGTACAGTACCTTTTTGATAGGCAAAGCCAGTCATAAAGATGTCAAAGACAGCACCAATTGGCTTTTTAAGGCATATTATGGAAATAGGACATTTATGGCTTACTGTTGTGTGTCATGCGAGGTTCTTTACATTATTCTGTTTCTTCTGGCGGAGAAGCAAAATGAGAATTTGATGGATGTTATATCACACTCCATACAACAAAAATCATTCCTCTCACTTTTGGTTGTAATTAGCTTGTTCGGATGGGCAGTGAAGCAAGCTGTTAATGTCATACAGATGAAGACGGCGGCAGATGTTTGTGTGCTTCACGATACCAACAAAAAACAGAAGCCATGATAATGAGACCTTTGTAAAATTTGTTTCAGTAAAGAACTATTGTCTTGTTGCTCCCATG

>cucumber_newGene_296 cucumber_newGene_296.1

TCCACCGGATCCGCCGGTGGCGACTGCCGTTGCTGCCTCTGCGCGATGCAAGCCACCATAGGAGTTCGCCCTGTTGTATGCTCGTTGGTGCCCCGAAATCCCGATATCCCGAGACGCTGGTTTGCATCTTCTAGGGCCTTTTTTATGTACTAGTTCTTTAGCGTACTGGATAATTAGTGATCGTTTCACTTCTTTGATTTTATCGCCGAATCAATGTGCCCAGATAATTCGTATGTAGATCAACATTATTGAATTTCAATCAAGCTGACGTTAATCGCACCTCTAATTCTACCTCACGGAAGCTTAATTCCTGCTAGATTATGGATAGAGATGTAGTACAGTGTAGGTACTTATAGATTTCCTATCGTTTTGACTTTGGGGAAAGAGAGTAGGTCTTAGCATGTGTGCTGGAAGCCTGTAGAGTATAGGAAGGACCTTGGGAATTGTACCATGCTAATAGAGCGAGATTCTCAGAGTTCCGACAATATAATTTGTACGGCTTGCGGCTTGATTTTGTTGGTTTTTGATCTTGAAAATGGAGAGGAATCCATTATTGGGTCGGAAACTCACGGTAAAGATTAGAAGATGGTTATGAATTGAGAACATTTTAGTTCTTTTTCACAATTTTGAAGTTGATGGGAAACTGTGGTTGTTCTTTATGTACTTGTCTACAATTTATGGAGGTGTTCTTATTAACTAATAACATTTTTTATTTTAGGTATTTTTCATCATTTTCAAGTTGATGGGAAACTGTGGTTGTTCTTTATTGACTTGTATACAACTTTTGGAGGCATTCTTATGGACTTTCATTGTCGTATGTACGTGTGTCATCAAACCTTTTGTTCGTTTTTTTTCAGTACTGGAAACTTATATGAACCCCTGAACCATATCTTTAAAGCCTAAACCATTACAAAATACTATCTTCAGGCAAGTTCTTGTCGCTTTTGGTTGCAGCTGAAAACTTTTGATGTTGTGTGCTCTGCTTTATAAAATTTGAGTTCTTTTTCTGTCTGCTGACTTGTTTCCACTGATAAAACAAATAAATCCCGAAGCAAAAAATTGAGTTATTTCTCATTAATCAACATCAGTACGTAGAATCCACTGGTTATGAAACCGTATATTGCAAGATTGATATGATTAGAGTACTCGTTTGGATCTGATAAAGTTGTTCTGCTTAAGATTTTAGTGCTTATGAGATTTTTTCCCCCAAAGCTGACTAATATAATTTGTGCTAAAAGAGCCTTTCCATGTGGGCCTAATTGTTTTTTCACCATCATTATCCATTTTGTGTTGCTTAAGTTTTTCTGTTCTTTCATTGATTGGGGTAGTGCATTATTAGGCAGTCCTGAGTATACTTCACTGGCTCCCTCTACGATTGATCTTTTCCAATTTTCAGATCATTTTGATTCTCATCTTCTCAGTTACCTTGTTTTGATCTGTAAACATGTTCAAAATTTTGTTGGTTTCATTTCTAGTTTGTTTGAATAAAACCATTTGAACAAGAAACATCACATCATCACACCAAGTTGCCATTCAATAAAGTGTTTGTCTAAAAAGAAAAAGGTAAGAGAGAGAATAGCAAATTTGCAGGTAAGTTTACTCTATGAAGATCAGAGTTCTGAAAGCAGCCCCCCCACCAGGAAAAAATAGAAAAGAGAAAGCAAAACAAAAAGTGGAATGATAAATGAAGCCTTTTCAAACTGCAGGTCACTCCCAACCCAACTAACAGTCATCATGCAACGCCCAACCTTCTACAGCTTCAATGGCAGCTTTTGCTAGAGAGATCAAAGTTAAGGCTTTGGTGGACAGTCCCACCTACATGTCTAAAACGAGTTGTGCCACTGCCACCATCTATGGATGCTTGGTTGTTAATCACTTATTCACCGAAAGAAGAGTGAATCAATGTAGATTTGGTTGTACGGACGGCGACAATCCTGAGGTGAGTATTTAAACAAGAAAGATACTTAAGGAGGACTTGCTACATCTATCTTTGTGCATCACACCGTGGTTTGTTACATGACAAATATTTTTTAAAATATTTATTTTGTGTATGGATGAGAGTCATAGAAATAGATTTATTGAGAGAATAAATTTTAAATTTGCTTTTATGTGATATGAAAAACAGTTAGAATTTAGTGTCTGATTTGATAAAGTTTTATCAAATGAGAAGGAATAGATTTTAACTTTCTCCAAATCCGTAGACTAAACCATTACCATTTTTGGAGCAACTATTAGTGCAGTTGGGTCCTTTTTTTCTTTCTTATTGGTAATCTAAGTGAAAATTATTTGTAAAAGTTTAATGGCTTTTGTGAAGTTCTTGTATTGTTAATCAAATTCTAAAGGGAAATCTTGTACTTTATGTCAATCATTCCTTCCATATCTGATGAAGAAAAAAAGGCTATTTTGCTTTCTACTTTTTACAAGTTTAAGAAACCAATCTAATGCTAAATACGGAGGCAAACAACATTTGATTGGTGAGTTTGAAAGATTAGAGTTTTGAGTATATAGATGTGTTGTCTTTTCTATTCTACTTAAACTCATGATATATTATTTCAGCATAAATATTAAAACAAACATGTGGACTAAATTATTACAAAGAGA

>cucumber_newGene_297 cucumber_newGene_297.2

GAATTGTCGTCGCCTCCAATAATTTTGACGATACTGTCACTCACGCTTGATATCGTATAACCCGACCCACCTATATAATTCCCTCTCTTTCTTCCCTCACTGGCCGCTTCCAACAAATTCTTTCTTTTATCATTGTTCTTCATTTTTCAATTCAATCAAATTGCGGCCCCTTTTGCCTATGCCCCTTAAAACCCTTTTTATCAAATACGATTTCCAGTTTTAGTTTTTTTTATTGGGTTTGTTTTGTTTTTGGATTCTAATCGATTGTTATGGCGGCGAGTGCATCTTGGCATCCTCAGGAAAATGGGTTCAACGAGATCTGTGGCTTGCTGGAGCAGCAAATTTCTCCTACTTCGAATCCGGATAAGTCCCAGATTTGGCAGCAACTTCAGCAATACTCTCAGTTCCCTGACTTCAATAATTATCTTGCGTTTATTCTTGCACGAGCTGAGGGTAAATCAGTTGAGGTTCGACAAGCTGCTGGCTTACTTTTGAAAAACAACCTTAGAACTGCATACAAGTCAATGACTCCTGTCTTTCAGCAATATATTAAATCCGAATTGTTGCCATGTATGGGAGCTGCAGACAGACATATTAGGTCTACAGTCGGGACCATCATCAGTGTCATTGTTCAACTAGGGGGAATATTGGGGTGGCCTGAGTTGTTGCAAGCACTTGTAAGATGCTTAGACAGTAAAGACCAAAATCACATGGAAGGTGCTATGGATGCTTTGTCCAAGATTTGTGAAGATATACCTCAAGTGCTTGACTCTGATGTACCTGGATTGTCTGAACGCCCAATCAACGTATTTCTTCCTAGATTATTTCAGTTCTTCCAGTCTCCACATGCTACATTGAGAAAACTCTCCTTGAGTTCTGTGAATCAGTACATTATGCTGATGCCCACTGTAAGTAATAAGTATGTGATATACTTTGTCAATACTTATTTTGGACCTTGTTCTGGTCTTTGTATTGATAGTGGTTCTTACAATATGTATGATTGCAGGCGCTTTACATATCCATGGACCAGTATCTTCAAGGTTTATTTGTTCTTGCAAATGACTCTACGTCAGAAGTGCGAAAGTTGGTTTGCCAAGCATTTGTGCAGCTAATTGAAGTACGCCCAACTTTCTTGGAGCCACATCTGCGGAATGTAATCGAATACATGTTGCAAGTTAATAAGGATGCTGATGAGGAAGTTTCGCTTGAAGCCTGTGAATTTTGGTCTGCATATTGTGATGCCCAATTACCTCCTGAGAACTTAAGAGAGTTCTTGCCACGTTTAATTCCAGCATTATTGTCAAATATGGTCTATGCTGATGACGATGAATCACTTCTTGAGGCTGAGGAAGATGGATCTCTTCCAGACCGAGAGCAGGATCTAAAACCCCGTTTCCATTCATCAAGGTTACATGGGTCGGAGAATGCAGAAGATGATGATGATGACATTGTAAATATATGGAACTTGCGCAAATGCAGTGCTGCAGCTCTTGATATTCTCTCAAATGTGTTTGGGGATGACATTCTTCCTATGTTGATGCCTGTTGTCGAGGCCAATTTGTCCGCTAATGGTGATGAAGCCTGGAAAGAAAGAGAAGCTGCTGTACTGGCTCTTGGTGCGATAGCTGAAGGTTGCATTACTGGACTTTATCCTCATCTGCCTGAGATGATAGATTTCCTCTAATAAGGAGTATATCTTGTTGGACACTTTCTCGTTTCAGCAAGTTCATTGTGCAGGGCATTGGAACTCAGAAAGGTTACGAACAATTTGATAAAGTTCTCATGGGACTTCTACGAAGATTATTAGACAATAACAAGAGGGTGCAAGAAGCTGCTTGTTCTGCTTTTGCAACGCTGGAGGAGGAAGCTGCCGAGGAATTAGCACCCCATTTGAAAAATATATTACAGCACCTAATATGTGCTTTCGGAAAATATCAGAGACGGAATCTACGGATAGTGTATGATGCTATTGGAACCTTAGCAGATGCTGTAGGAGGGGAGCTAAATCAGCCTGTTTATCTTGATATTCTCATGCCACCATTAATTGCCAAATGGCAGCAACTTTCTAACTCAGACAAAGATCTTTTCCCCCTGTTGGAGTGCTTCACATCTATAGCACAGGCTTTGGGGACTGGATTTACCCAATTTGCTCCGCCTGTGTATCAGAGATGCATAAACATCATCCAGACCCAACAAATGGCAAAGGTTGAGCCTGTTTCTGCTGGGGTTCAGTATGATAGAGAGTTCATTGTGTGCTGTCTTGATCTTCTTTCAGGACTCGCAGAAGGTCTTGGCAGTGGAATTGAAAGCTTGGTTTCTCAAAGCAATTTGAGGGACTTGCTCTTGCAATGCTGCATGGATGAAGCTTCTGATGTCCGACAGAGTGCCTTTGCATTGCTTGGGGATCTTGGAAGAGTATGCCACGTCCATTTGCAACCGCGTTTATCAGAATTTCTTACCGCTGCAGCAAAGCAACTGGATACTCCTAAGCTGAAGGAAATTGTTTCTGTGGCAAACAATGCCTGCTGGGCAATTGGAGAATTAGCTGTTAAGGTACGCCAAGAAATTTCTCCTGTCGTTATGACAGTGATTTCAAGCTTGGTCCCGATTCTTCAACATGCACAGGAGCTAAATAAATCTCTGGTAGAAAACAGTGCAATTACACTTGGAAGGATCGCATGGGTTTGCCCTCAGCTAGTGTCACCACATATGGAACATTTCATCCAACCATGGTGCACTGCTTTATCCATGATACGTGATGATGTAGAAAAGGAGGATGCTTTTCGAGGTCTTTGCGCTCTGGTTAAATCAAATCCTTCAGGGGCAGTAACTTCACTTCCTTATATGTGCAAAGCTATTGCCAGTTGGCATGAAATAAGAAGTCAAGATCTTCATAATGAAGTATGCCAGGTTCTACACGGTTATAAGCAGATGCTGAGAAATGGTGGGTGGGACCAGTGTATTTCGTCTCTGGAACCATCCGTGAAGGACAAACTATCAAAATATCAAGTATAGGATGATGATGATGTTTTATCAGTTATCAGCAAGCATTCTTCTCAAACATATGCTTCAAATTCTGTCTCGAAAACCGATGGGATGGGTCGTTTTGTGGCGTTGTCTGTGTGTCCTTATTATTGATGATTGGAGCAAACTTCAAGTTAAAAAAAAAAAATAATCAAGTTTGCTGCTGAGCTTGAAGAAGATTGCACATCCTTCTTAAGATAATTATACAGGTTGACTTGGTTGGTTGAGACAGCACTTGAAAATGAAGCCAGCTGAAGTGAGAAAAAAAAATGTTGTACAGGGAGATATGTTGAGATAGTGTGGTGCCCTTCTTATAGTGTTCCCATTCCTTTTCCTTTTCTTTCATCATGAGTCTGGATTCGTTATATCGATTTTGGGGCGATGGTTTATAGGTAGAGAGTCAGCACATTTTTCTTTTCCTTTTTGATTTTACGACTTTGGTCCTATGATTTTTCCATTAATGAATAGGGCGCATCGGCGCGATAGGTTTTCTATTATGAAAGATAGAGCTGAGTTATTCTTGCTACATTTAGCAGCTGGTGGTGATTTGGGGATTTTTTGTTTGTCTTTGGAAGCAATAAGGAACCTT

>cucumber_newGene_298 cucumber_newGene_298.1

TATATATATATATATATATATTTGAAAAAGAATAATAGAAAAACCTTTTAAAATATAATGGAGGTTACCGGGAGCGGCAGAGCAAGGGACCGCGGCGGAAGTTCATCAATGGAGATAGAGCGTCGTTCCCTAAGAGCAATAACGACCTCCTTCTTCGTCGAAGTTCTAACCGGACCTTGGTTCATGGTATTCGCTTCATTCCTAATCATGTCCACCGCCGGAACGCCTTACATGTTCGGCCTTTACTCCGGCGCCATAAAATCAGTCCTCGGCTACGACCAGAGCACTCTGAATCTAATTAGCTTCTTCAAAGACCTAGGCACAAACGTGGGCATAATTGCCGGACTAATCGCGGAAATCATGCCACCGTGGGTGGTTTTGGCGATCGGAGCAGGGATGAATTTCGTGGGATACTTCATGATTTGGCTATCGGTGACGGAAAAAGTGGCGGCGCCGCCGGTGTGGCTGATGTGCCTTTACATTTGCATAGGGGCGAATTCGACGTCGTTTGCGAATACGGGGGCGTTGGTGACTTGTGTGAAGAATTATCCGGCGAGAAGAGGAGCGGTTTTAGGGATTTTGAAAGGATATGTGGGATTAAGCGGTGCAATTATGACGCAATTTTATCATGCGATTTATGGAGATGATTCGAAATCGTTGATTCTTCTTATTGCTTGGCTTCCGGCGGTGATTTTGGTGGTGTTCTTGAGGACGATTCGGATTATGAAGGTTCAACATCGGCCTAATGAACTCACAGTTTTTTACAGATTCTTGTACGTTTCTCTTGCTCTAGCCGGGTTTCTCATGGTGATGATCGTTCTTCAACAAAAATTTAATTTCTCCCGTATTGAGTACAGTTCCAGCGCCGCCGTCGTCGTCTTTCTCCTCTTTTTTCCCGTCTTCATCGTCATCGCCGAAGACTACAAATTCTGGCGCATAAAACTATCTCAACTCCTAAATCCTTCTCCACTCACAATCATCACCCAAAAGCCAACACCGCCGCCGCCGCAAAACCTCGGAACCTTCGGCATATCTCCCGCCGTGAAACCGACGTCATCAACGCCGTCCTGCTGGACAACCCCACTGAAGCCCCCTCCAAGAGGTGAAGATTACACGATTCTACAAGCGCTCTTCAGCGCCGACATGTTCCTTCTTTTCCTCTCCACCGCGTGCGGCGTCGGCGGCACTCTCACCGCCATCGACAACCTCGGCCAAATCGGCGCTTCTCTAAAGTATCCTAAACAGAGCATTAGCACATTTGTATCATTAGTAAGTATTTGGAATTACCTAGGGCGCGTAGTTTCGGGTTTCACCTCCGAAATTTTCCTCAGCAAATACAAATTCCCTCGAACCCTAATTTTAACCTTAATTCTCCTCCTCTCCTGCGTCGGCCATATCCTAATCGCCTTCAACCCTCCTGGCGGCCTCTACTTCGCCTCAATCGTAATCGGATTCTGCTACGGCGCTCAGTGGCCGATTCTATTCGCCATAATCTCAGAGATCTTCGGCCTCAAATACTATTCCACGCTTTACAATTTCGGATCCGTTGCGAGCCCGATCGGGTTGTATTTTGTGAATGTTCGAGTCGCTGGACATTTGTACGACGAGGAGGCGAAGCGGCAGTTGGCAGCGTCCGGGATGAAGAGGATGCCTGGAAAAGAATTGAACTGCGTTGGTGTGGATTGCTTTAAAATGTCGTTCATTATAATCACCGGAGTTACACTGCTTGGTGCTCTATTCTCATTTGTTTTGGTTCTTAGAACTAGAGCCTTTTATAAGACTGATATTTATAGGAAGTTTAGGGAGGAGGTTGATGAAGGTGAAGCTGCTGGAAACGACGTCGTTTCCAATAATGACAGAAATGTTGAAGCCAACAAGTAAATTAAAGCTAGCTTTACAAAGTATTTTGATCGTTTTTTCCGTTAGATTACTTTAATTAGGTTGTAACTATTGTTGTTTATTATTTATCATTTCATCTTGTGGAATGGAATTTTTATTCACATCATCAAACTAGCTAATCTATCCATCTTTGTACTAATTTAAATTGAAA

>cucumber_newGene_300 cucumber_newGene_300.1

TAAATATCATCATTACATTTCTCTCATCTTTCAATTGCTTCTAAAATAAATAAATATTAACCAAACTCTTCTACTCTATCCCTTCTTCTTCTTCTTCTTCTTCTTCAATTCATTTCATTTATTTTCATTTCTCTCCTCTCCTCCATGGCCTCTTTCTTTTTGACATAAGAGAGAGGGGTTTTCAAGAAGGAAGAAGAGGACGAAGATGAAGATGTTGCAGCAATATATAGATATGTCTGATTTTGTTGATGAAGAGTAGCAGCATAGCTTAAATTTTCATGGGGGGGTTTGTCAAAGGGTTTCCAACAGAATTTATTGGCATTCTGTCCACCTCCTCCACATATTAATTTATATTTATATATACATATATATATATATACTTTAATTTGTGTGTGTTTATTGTTATAATTAATGGTTTGGAGGTGGGCATACTGCCAATTGAGTTCTCTTTGGATCCTATTTGTAACACTCCTTCTTGAATAATATCTCTCTATTCTATCTTTCTTTTTCCTTTCCCCCTTTATTTCCTTCTTAGTTCCATCCCCATGCCCTCTCTCAAGGTTAAAACCATCTGGAAAATTGGTAGCAGGAAGAGGGAGAATACGATGGATCATCAATATCTAAAAATGGATGGCACGACCTATATATCCGATCCTCGGATAAGAGAAAGGCCTAAATGAGCAGGAGGGTGCAGCGGTGGTTGCAAAACCTTGGGCACAAACCCAGGTGAAGTGACCTTTGGGTGCAGATGTTGGTAGTATATAGCACCAAACATTCAAAAGAATATTTCACTTAAAATAAATAAATACGTATGTATGTATGTATTACTTTTGGACTTTGGCGGGGTTATGAAAACCCTTTTAATTTCAAAATTTGATGGTAAAAAGTATGGAGGTGGGAGTAACTTAAAACAGTAATAATTGTGAACTTTCAAAAAAGAAAAAGTAAAAGAAGAAGAAAATAGCAATAATGGAAAATTTTTTTGGTAGGTTTTGATTGAATGCAGAGTGTATTGATACAGATTTGGGTTCGATTTTATGTTTATTTATCCTTTAGCTATACCCAAAGATCCAAACCCCAGAATGAAAATAGGTTGTCGTTTTTTATGCATGGTGTACGTACATGGAATTTCAGAGATATATTTCCATTGTTAAAATATGGGTAATGAATATTTTAAACATTGGGAGCCATGAAAATATGTGGAAGATAAGAGATTTTTCTTTAGAAAATATATGAATATGAAAAAAATTAAATTATGAAAAATATCGTTAGAAATACTTCTAAATTTTCAAAATTTTAAAAAATGCAATTAAATCTTAAAAATGATTAAAA

>cucumber_newGene_302 cucumber_newGene_302.1

TTCATAATCATTTCATAAATTGAAATTATGCTTTTTTATTAAAGGACTTTACAAGTAAGTATTGAGTTTCTTAGTAATCGATGCGCAAATAATTATTCATGTAATTTTATTTGGGTGTCAATATGAACATAGTTCAAATTGATGCTCTAATGTCAATTTTCTCAACAAGCAAAATAAAAATCAAAACTTCAAAATATAAAATTGGACCAAATCTTACAAAATTTCAGATGGGTTAGTAACCTCTTGTAGAAACGTTTAAGTTCTAAAATTTATGGGGTGAGATTAAATTTTTTTTCTTCTGGTTATAATCTATATATCTTAATTGAAGCGAGGAGGTATGTAAATAAACAAAAAAAATTGAAACATTGGGAACTTAATTTAACATTTAGTTTAATATTTAAGAACAAGCTAAAGATGCTATGAAGTTAATTGGATTTGAAAAGAATTGGAGTACAACAATGGTTGAGTACCTGAGTTGGCTTCCTATGAATTTTTATATTTTTATCTCCATTAAAGTGGGGATGGATATGTATGGATAAGCTTGAATTGAAAAGGAGACGAATCGTCATAACAATTTTGCATATATAAAACGGGAAGACAAATGCTACGACAATTGAATAGTGAATATAGGAATCTTCTCAAGAAAGTCCGATTATTTTCGGTTGGGTAAGGCGGGAACCGAAATGGGTCGACCAATTCGTCCAAGAGCTTTCTGGGGTCTCTTGATTTCGGAAATGGCTGCGATTAGGCCGACTTTATTTCATAAGAATCCATTCCTTTTCTCGTCCTTTTCAGTTGGGAGAAGCTCTCTTTCTGCGAAGGAAGAAATCGACGAAGCACATCTGGGAGTGTCTTCTAATCTTATTCACGAGTTGGAGGTGAAAAATGTGGTTCAAGTGAAAGATGAACGTAAGCTGTTTGATGAAATGCAAAGGAGATTTTTGAGTTTTAAGAAGCACAATTATTTGGAGCATTTGGAGCATTTTCAAGCTCTTGCAGAACTGCAAACCCCTAAGGTAATTGAGCAAGATGATGAGCAAAACCCTTTCCTATTTCTTTGATTCGTTTTACCTTCTTGTACTTTTGTCCTATTTTGAGTATTTGCCATAAGTTAAGTTCACTGATGCCAACATGAGCTTAGCTCAACTGGCACCTGTATGTACATGTGACAATAGGTCCCAGGTTAACTCCCTATCACTACATTTATTATAATACCTTTAAAAAAAACACATGCTCACAAGTAGAACAGTTCATGGTGATTTCTTGTGTGGATTCTAGAGTATGCCCATCCAATATCCTTGGATTTCAACCTGGAGAAGCTTTCATGGTTCGTAACGTAGCAAACATTGTTCCGCCATGGGAGAATGGACCAACTGAAACCAATGCAGCCCTTGAATTTGCGGTGAACACACTTGAAGTTGAGAACATTTTAGTCATTGGCCACAGCAGCTGTGCTGGAATTCAGAGCCTTATGAGCATGCAAGATAATGCAACTGGCTCGAGCTTATTCTGCCATGGTAGCTTTGTTCATAAGTGGGTGGTGAATGCAAAAGCAGCTAAATTAAGGGCAAAGGCTGCTGCTGCACATCTTAGTTTTGACCAGCAATGTAAACATTGTGAGAAGGAATCAATCAACCTTTCATTGAAAAACTTGATGTCATACCCCTGGATCGAAGAAAGGTTAAAACAGGATTTGATTTCTGTTCATGGAGGATACTATGATTTCTTGAATTGTACATTTGAGAAGTGGAGTCTTGATTACAAGAACACCAGCAG

>cucumber_newGene_304 cucumber_newGene_304.2

CTTCTTCCTTTTTGTTTTTCTAATTTCTTTCTTTTGATTCATCTGTTCATATCGATTTCCCGTCGTCTTCTTCTTTCTGAAGAATGGTTGCGACGGTCACCGTCGTCGCCGACTCGCGGCACTGTTTGATTTGTCTTTCAGAAGCTTTTGATTACGTGTTGTCGGCACTGTTACCGACGTCCTGCATGGAGGAGAGAGAATTCAGTTTCTTTTTTCCTTTTTTTTTTGGTTTATTTAATTGCTTATTTATTTCCTACTGAACCTAAAAACCCTAATTAATTTCCATTATCTTCACAAAATGTATGTTCCTTCATCAAATATTGTGTTCATGATTTGAATTTTGAGCGGCGTTGTGCTGTTTCATACTCTGGATCTGGTTCATATCGTCTTCTTCATCATTATCGGATGATTCCGATCTCTCTCTCTCCCGTCTCGCTCTGCGGATTTCTATGTGTATACGCGGTTTTTTTTTTTTGTCTCCGTTATACATGTGAAAGATTCTTTACTGAAAATTATTTGTTTATCGTTCTTGAATTTGGTTAGTTTTTCACTGTCCCTTGGATAATTTGACAGAAGAGAGTGAGCACCCAGAGGCAGTTGCTATAAAGTGTATATCAATACTTTTGCGTGCTCACTTCTCTCTCTGTCAGATTCGTTAAACGGATCCAGTTTCATAATTCTTCTCCATCGATTTTTTCGGTTTTAGTTTTGATATTCTTTTATGGATTAACGAAATGGTTATTGGACTCCGAATTGTATGAATTGTATTTCTGCAGTATTGTTTCTTTAGAAGCGTTGCTTTTCATGTTGCTCACTGGCCGGTGTTATGGCCACGATCGTGAGAGAATACTGAACAACGGGACCTCTCTCAAGCCTACATCGTATAGGCCGGTCCTCTCCTAATCGGCAGATCAAGTTCAAAGCAGTGGAGGAAGAAATTGACAAAGTATGTAGTTCTGAGTAGGGATCAATACGCAGAGCCTAGAGATGGCATGGTCCGAAATTTTGTTTCAACTTCAGAAGATGGTTCTTTCCAACCTGAACCTGCCCATTGAACGTGGAGATCCTAATCCATTATTTTGTGTTTATTCAAAGACTGGCCTTGTGCACATATAAAGGTGAACTGTGAATGAATCTTTTGCCTTCCCCTTGGTGGGGCGTCCGTACAGAAACGACCGGCTCAAAAATGATGGAATCAGAAACCTTGAGATAAGAGCTTAACACAGTTGATCTCCGTTGGTTGGATCACCAATTTCTGTGTTGTACAATAGCGAAGTAAACATTTATTATGGTTTATATATAAACATGTTTTTGATTCCTTAAACAATCATATCGGTCTTCTTCACTTTCAATCGGACTCTTGGATGGCAAGGCAGCACATATAATAGAAGTTCGATTGCCAATTTCTCAGTGCAGCAACGGTCGAATGTATCAGGAAAATTGCTGGTGATTTTTTCGGGAATAGCTCCGTTGCACTTTTT

>cucumber_newGene_305 cucumber_newGene_305.1

TGCTCGAATTATCGGCCGAACCAGAATACATGTTCATACTCTGGATAATAGTTTCCTAACTTCTGAGTTCAGCTCAGTCTTTATTTGGAGCCGTTACTTGGCAAATCATTCCCGGCAAAAAAATTAAGCCCTAATCTATACTCTCCCCCGCTTTCCTCTGGTTGAGTTTTACCCTCGAGCTTTCCCAGCATGTCTACACTCGAAATCGAAGCTAGGGATGTCATCAAGATCGTGCTTCAGTTTTGTAAAGAAAATTCACTGCACCAAACGTTCCAGACGCTACAGAACGAGTGTCAGGTTTCTTTGAATACTGTAGACAGCATTGAAACTTTTGTTGCTGATATAAACTCTGGACGGTGGGATGCTATATTGCCTCAAGTATCTCAACTTAAGCTTCCAAGAAATAAATTAGAGGACTTGTATGAACAGATTGTATTAGAAATGATTGAGCTTCGTGAATTGGATACGGCTCGTGCTATTCTAAGGCAAACTCAAGTGATGGGTGTCATGAAGCAAGAGCAACCTGAAAGATACCTGCGACTTGAGCATTTATTAGTCCGAACATACTTTGATCCGAACGAGGCTTATCAAGATTCAACAAAAGAGAAACGCCGAGCGCAAATTGCACAAGCCCTTGCAGCTGAAGTTACCGTGGTTGCACCTTCTCGTTTAATGGCTCTGATTGGTCAGGCTTTAAAATGGCAACAACACCAAGGTTTACTTCCTCCAGGAACGCAGTTTGACTTATTTCGTGGAACTGCTGCCATGAAACAAGATGTGGAGGATGTTTATCCAACAACTCTTTCTCACACAATTAAGTTTGGTACAAAAAGTCATGCGGAATGTGCTCGATTCTCACCAGATGGGCAGTTTCTTGTTTCTTGCTCCGTAGATGGATTCATTGAGGTCTGGGATTATATAAGTGGGAAACTCAAGAAGGATCTTCAGTACCAGGCTGATGAAACTTTTATGATGCATGATGATCCGGTCCTTTGTGTTGATTTTAGTAGAGACTCAGAGATGATTGCTTCTGGTTCACAAGATGGAAAAATCAAAGTATGGCGTATCAGAACTGGCCAATGCTTGCGACGTCTTGAACGTGCGCATTCTCAGGGAGTTACAAGTGTTGTTTTCTCTCGTGACGGAACCCAGTTGCTGAGTACATCTTTTGACACCACAGCGAGAATCCATGGTCTAAAGTCTGGTAAGCTGTTGAAGGAATTCCGTGGCCATTCATCATACGTGAATGATGCAGTCTTTACTAATGATGGAGCACGTGTCATTACCGCATCTAGTGATTGCACGGTTAAGGTTTGGGATGTGAAGACAACCGATTGTCTGCATACATTCAAGCCACCACCTCCATTAAGGGGAGGAGATGCCTCGGTGAATTCAGTTCATATCTTCCCCAAAAATGCAGATCATATTGTTGTTTGTAACAAGACATCGTCAATATACATCATGACATTGCAAGGGCAGGTTGTCAAGAGCTTCTCATCTGGTAAAAGGGAAGGGGGAGATTTTGTTGCCGCTTGCTTATCACCAAAAGGTGAATGGATATACTGCATTGGTGAAGACAGGAATATGTATTGCTTCAGCCACCAATCCGGTAAGCTGGAGCATCTCATGAAGGTTCATGAGAAAGACGTGATAGGCGTGAGTCATCATCCTCATAGAAATCTCGTTGCTACTTATAGTGAAGACAGCACAATGAAATTATGGAAGCCTTGACTCTTTTACCTATGCTATTTTATGTGTAAAACTTAGCAGTTTTTAAGTTATA

>cucumber_newGene_306 cucumber_newGene_306.3

AGGTAGTAAAACTGAAGAAGCAGCTCACTAAGTTTGTTGAGGAGCGTCGTGGGCGAGTTTCTGTTAGTGCCAAGAATTTTTAGTTTCAAACTTGTTTGATTATTTCAATCATATCTAAGTTTCTACCTTGTTAGATGGTTGGAGAAGATTGATAGAAAGCAGGCTGAAATCTTGGCCTTACAAGTTGCTTTGAAAAAATGAAGGGCTCAATAGACACTTAATTCGCACTGTTGCAAAAATTTTGAAAACTTAATGAAGGGCTACTTCATAAATTTCCCTTGGTTTTGTTAGGTGGGAGGGTGTGCTGTCATTTTTATCCTTATGAACTTATGTTTGTTTGTGTTCTTCTCATGGTTTTATTTTAGCTTCCTCAAGATATTGAATGACATTCCATTGGGCATTTGTAGAGCAACAAAGTTAAATCGCTTATTGGTACAGTCCCTTCCTTGTTCTATTCTTTCATTGGTTTATTACAGTCTTCTTGTTTGTAATTTCTGCTTTAGTGGAGTCTTCCTAAGGATTGGTTGTTTTCTTGATTCTTTATAATGTTTTGAAAATCTTCGTTTCCAAACTTTGAAAGTTGTGTCTAATTGGTGCTTAAATTCTATAACATGTCTAATAGTTTCTTAAATTTATAATTGTGTTGACTGAATTCCAATTTTGTATTCAAAATCTCTATATGTTCATTGAAAGCTTTTACTTTATCCAGAGGTGGAGGAAAGTGAAGGTGAAGTCTTCTTCTTTTTGCTCAAACATGCATCCATTATCTTTTTAGTTTTAAATTTTGGATGAATATGTGGTTTTTGGTTACTGAGGTCATGATAGAAGTTGAAGATTATGATAGAAGGTTACGATAGAAGTTGAAGATTATGATAGAAGTTGAAGATTTTACGATAAGAGTATGTAGAGAAGAAGCAGCGGCAAATTTTTGTGAGTTGCTTAGAATGTTTTATAATGCAATGTCCTTCATGAAAATAGAGGTTATGGTCTTAAACTCCCTTCCTTTATTGTATATATTTATTTCTCTGTGTTTACCTAATTCATCAATTTTGTTGTTATTTCTCTGTTTACAAGGTTGACATCCCACATTCTTTTCCAAATAGAATTTTTTTTTATAATTTGAAACGTTTGGTGTTGAAAACAAGGACGACCAAGGAGGTGATAGATTTGCCAATAAGTATATTGGTGTTCTTAAAAATATGCCTCAATTTAGAATCATGGGCTGTCCATAATGAGGTGATTTGTTTTCTGAAGAATTAAGATATATGAAATACCTTAGCTTTTATGTTTCGTTCTTTTAATGAGAAGAACTAATTAGATTGCTTATTTCATGAATTTTATATAGATACTTAGGTAGTGGCAATATATATAAATGCAATGACGAAAAGCCTAAAGAAATGTTAAAAATGCTCCATAAAGAAATATGAAAAAAGAGTACTAATAATCGAAGTATTAGGATCTATTACATTATTAAAAGTACACAAACTAAAAGTCTAATAAGATTTTGAACTTGAACTTACTTGTTGATGGTACAGTTCAGTATGTGTATATCCAAACTAGTGACAAGAAATATTCATTATGTGAGGTTTTCTCAATTCTCGACTATTTGTCAAAGAAAATAAACTACTACACAATTGTGCTATTTACCATATTTGTCTTGTAGGAACTAAGGGCGACATTCAACTTTGGTTAGAATGGTGAGCTTATTTCTTATTTCAGGATAGTTCATATACTAAATTTGATGTTTATATGGTTGGTTTGATGAGTTTCAAATTTCAAAAGTCTACCTAAATCTCTATTGTTTGTTTAGCTCAAAGGTATAAAAGTGTAAAGTCTGTGGACTTGCACTGAATAGAGCCATGGTAAGTTTCTAATTTCAAAAGTCTACTATAAGCTCTATTGTTTGTTGATGGATTTTAATGGAATTTTGAAGGTTTTAGAAGAGATGTACTGGGGAACCTAGCTTTCTAAAGCATGGAGGATCTAATATGGAACCTGGTTCTAGTGGAGCCAACAAAGTTTATGAAATTTCCTTCAAAGAAGCTAATCAGAGGTGAAGGATTACCGATTGTTTGTTTATGCTTGTACAAAAAATCATGTTATATGTATTATGATAATATTTACAATGGTTTTGATTAAGGGTTGTAATTACATGCTTGTAAATTGTTCAAACTTAATTAGAAATATACAAATATTACAAAG

>cucumber_newGene_307 cucumber_newGene_307.1

CCAATATCCAATTCACTTACACTTACCCTTTCCTCCATAACTAACATACCATGAATTATGGAATGAATTCGTGTTCATTTCCCGCCACAATTCAGTAATTTTGGTTGCATTCCGATTTCATCTTCATTTGAATTTAACATTTCGGATTCAATTTCCCATCACTACTCATCACGCATTATATAAATTCAAGCCCTTCTCTCCTTTCCCCCTTCATTTCATCACTCTCCCTTTTCCTTCTCTGCGCCTCTCTCCTAATTCTACGGAACCACCATGAGAGCTCTCAGGAACGCCGTCGTCACTCACAAGAAACCCAACCCACAGAAGCTGAAGCAGAGAAAAGCTGAAATGAAATCCACGAAAAGCAAGAAGAAAGATCAAAATGCTCCAAAACGTCCAGCTACGACATTCTTCGTTTTCATGGAGGAATTTAGAAAGACGTACAAGGAGCAATTTCCTGATGCAAAAGCTGGTCCAGTGGTCGGCAAAGTTGGTGGTGAGAAATGGAAGTCCATGTCTGATGCTGAAAAAGCTCCGTATGCTGAAAAAGCTTTGAAAAGAAAGGCAGAGTATGAAATAGCTCTTGAAGCATACAAGAACAACCTCAATTGCCCTCAGAATCACAGAATGCCAACAGAATCACAGAAATCAACCTCTGAGGTCAATGATGACACAGAGCAGGAAGCTAGCTCTAGCTCTTAGATTTTTGCAGGACTTGAGTGTCCATTACAGGTAACAGATCAATCTCCATATCTTTTTCAATAAAAGTTTCATGGTTATGAGGACGAAAGATAGCAAGTAAAGGGGGTGGAATGGAAGGCAAGAATTTGAATAACTCTAAACAATTTTAGGACAAAATATTTCTTTCGATCATATGTATATATATTCTGCGTACAGCTCTGGTGGTTGGTGAACTCAACTCTCTTGAAATTATTAGGGTTG

>cucumber_newGene_308 cucumber_newGene_308.3

TGAGATTCTAAACTTTATTAAGTAGGTCTTGAGTACCTATTGTGTTTCAACTTGAATTAATCAGTACCCATAAAGTTTGGATGCTAAATTGGAGTTCTTGCAAGGCCTTAGCTACTAATTAGCTGAGAAAGAGTAAAATTAAGGCTACCTTTATAAGTGAATTAGTAAGAAATTTTGTGATTTTAGTAAGTTTTGTTATTACCCGATTAGTTTCGATATGAAATTTCCATTACCATGTTGTTGATTGGAAGAATTTAGACTCTAGAGAACTATGGAGTATTGTTCACCGCACTTAAGATCGTTAGTACAAGTGTGAGTAGGCTCTTAATTAGTGTTAACTCATTAGTTGTTGATGGGTTTGTTAAGTGTTAACTCATTACCTGGTATTTGAATTTCAGTTCTTTTTTTGCCCCAATTTAACTAGTTGATTGAGTAGAGTGTCTCATAGATTGAATTATTATGTCAATATCCATTTTTGTTTAAATGTTTATAGACATATTAAAGTTAAAATTTAGTTGTTGACATACTTTTGATGATCAAGTGAAATAGTTTCTTAATTTTTCTGTTCTATGTGAAGGGTGTATGAAAAAAGATGGGGACAGTTAGATATATTACATCTGGGCTTTTCTAGAGAAGTATTATAACCTCTTTTCGAGCTTTTATGAAAATATGTAACATTGAAAAGGTATCTCATGGGTATTTTTTCAATCTTATTTTGTTGTGATCAATTGAGATACATAATATGCACTATAAATTTGTTAAAGTGTTGGACTTATCTGTTTTTTTAATAGGGTTGAAAGAATGTCAAGGTACGTTTTGTTAAAGCATATTTTTATTTTGATAAATTTGAAGTTGTCAAGTATGTGTATAATAATGATAATGTTAATATGAGTAAATTTATGGCTCTGTATGACTGTGTGCATATTTAAGCTAATGATATTATGTATGGAAGACAACTTGCACTAAAAGGCTTAGTAAATAGTCACAAGGCACCTTTTGAATTCAGTAGATAGAAGAAACTATTGTTGAGATAAAATACAAGGAATAAAGAAAAGTTGTATGTACTCAGAATTCAAACTTATTGGTCATCATCTGATTATCATGCTATAAATGTTTTGGATAGGCGAGAAGAGCTATGAGTATCATGCACTAGAGTGACGAAGTTGAATATAAAAGAGAAAGACGTGCAATTTTTCGCAAGGCATAATCATGATAAATTAATTTTCTTCCTTACATTGTGCCATTTTGCTTTGACATTATAGTATACTTCTAACTTTATGTCTCTCCAGCATATTTGAGATGTTTTGCAAAAAATAGTTATGCTTGAAGTTGGACAATATTATATCATAGTGTAGAAATGTTTAGAGATAAGTTATTCTTAGTTTGTGTTTTGCATCTGGATTTTTTACTAAAACTTTGGCTTATGTGACTTGTGTTTCAAAAGTCTAAGATAAACTGTATAGTATGTACTGTTAAATTATTGCACAATCTGAATGCTTGAATTTATGAGTTAAGGTGAATTCAACACTACTATAAAACTCTCCCTCATTTGTGAAAATGAAAACTTGTAAAATACTAAACTAATACAATCAAATTCATCATGTGCAAAATTGTTTCATCAGTTTTGCATGATCATAGAAAGCTTATCTTCTGAAGCATGTTAATATGATTCTTGTACCAAGATTTTCAATATCTTTTAACTCATGTAGTATTTTTTTTATGTGAGATCTTAAAACCCATATATCCAAATGCAATGGGTCTTAAACCCTTTTCTTCCCTTTCTCTAATCTTATTTTTTCAGATTTAGCCTCTTTGTTTACTTGTAAAGTAACATATTTATCTTTAGTCTTTAAAATGTGTGGATGAATTTTTGCCATGTTTTCGAGTGGATTACTTTGGTGAGGAGACATGTTGATCAAGTTGTAGTGAATGATGAAACGATATTCCCTCTCTTTAAGAAATTTTGTAAGATGCAAAATAAAGGTTTCAAGTAGTTTGATTTAAGCTTTGGTTTGGTAGCTATTTTGTTTTTAAATTTTTAAAAGTTAACCTAACGATTGACATCTTTCTAAAGTTCAAAAGTTGAACTCTTAGTTCAAAACTGTCTAGTACTCCATGGTATGCATCTTTTTTCATTCCTTTATTTATTTATTTGGAGTTTTAGGTGTATATTTTTGTATCTGATGAATTATTTGGGTTTATAGTCAAGAGGGTGTGTTTAGATTTTATTGTTTGAACTTGTTGAGTGAAGAAATTGATTGTTTGGTATGCATGATCCTTGTTTTAGTAAAAAAAGGTATTTTCAGGGATGCTTTTGGATGGTGAAGTTTATTTTTTTTGGGAACCGTTGTTCTTTAATTGAGTTGTTTAATTGAATTTAGTTCTTTGAGGAAACTTTAAGACGCTTTTTGTCCATGTGTTTTAAGTATTTTTTTTTGGTTAACTTGTAGTCTTTTACATTTTCCCCACTTCTTGATTTATTGTTCTTGATGGTTTATTCACTTGTTTTCACAAAGTTTGCTCAATTGGAGTTGCAATGAAGATCAAGATTCAATCGGAGCCATTTTTTGGTTTTTAGACTACTTTAATTTTAATTTTAGTTGTCTTTCTCAGCATGGTTGTCATTTCCATAACTGACTCTAACTTGTTACTTCCCTGCACATAGAAATAGGTTCAGAATCTTATTTATGTAAGATATTTATTAAGATAAACCTTTAGCCATGGAGAACAAAAGTATAGAACTCTTAGTACTAGAAATTTTCTTGCTTATGTAAAATTTGAAGTCTACATTCTCTTGATACTAATCAGTGGGCTCTAGTGGCAGGGCCCTTCGGATTCTGAAAAGTTTTGAAGCATGGAAAGAATGTCACGACCCAACCCAAAAGTTTGGAAGAATCTACTGGAGCAGTGAAGCCATCTTGGAGCAATTGATTGATATAACGCTTTTTGTATGGTTGATAGCAAACTCTTGGTTCCAATGTACTAATTTTAGACTTCTTCGTAGCTAAAGATGACAATGTTTATTATTTTTCTCTGTAAACTACCTTTCAGCAAAAAATGATTATTAGTCAATGTGTGATATTAGT

>cucumber_newGene_309 cucumber_newGene_309.1

AGAAGACCCACCAAGCAAGAGATCCTTTGGAGACCTATCCAAAAGTTGAAAAAAATGTTACAATAGGAAAAAAGAAAGGATTCATCCATGCCAATAAGTTCAAGACATATGGGAAAGATTTACACTTATGGATAAATCATGGATGCACAAGAGTACGTTATCCAAAGCCTACAAAGATAAGTTTGTTGGTGGTTTCCATGAGTTGCTTATGTGTATAAGTTTGTTATTTTATGTTTTATAGTTTGGATTCTTACTTTCTTTTTCTCCTAAACTGTTAGAGTTTAGAGGTTATGTTCAATTACATATTTTTTTAGGACTAGAATTAGTGACAAAATGAGGAAGCTTCGAGAGGTTGTCCCCAACATGGATAAGGTAAACTATGTCATTATTGATCATTATGAATGAATTTGGATCTTTCTTTTGTGGCATATTATTGAAAATTTTGTTATGCCTTGTGGTTCTTGTACTTCCTTGAATTCCATTTTTAGCGTTTGGCTGGAACTTTACTTTATGATTAATGAAGTCTAACATGTACTCAATTAGTTGAGTAATGGGGATAAAGTTTATGGTAATTTTACTTTTGCTCGATCATGTTGTCTTGGCTTTTGTAGCTTGTGGTTAAGGTATCTGAGCTATGATCTTAATATGGAATGAGACCTAAATGCAATTCGAGTTTGTTTGATGCTTTAACATGAGAATAAAGCTGCTTAATGTCGATGAAATGAATTAAGAAGCAAGATTTTTGTAAGATAGTGGAAATAATGCACACTTTCTACCGTTATATCGCTTTGATGAATGTCGGTTAAAAGTTGCCCTATAACTGTTGTTGTGATAGAAGAAATTTACTACGTTATTCTTCTCTTCTTGCAAATAGCATTCTTATGTACTAAAATTACAACCCAATTTTCTGAATTTCAAATTAGCTTCAATTTCAAGATTGTGTTCTCTTTTTATAGTTGATTTGATCTTACAAATGGCTAAATATTAAATAGAAGTAGAATATAACTAATGGTTTGTTTGAATTTAGTTTGATTAGTTGTTTGGGATTAGTTTGAGGGATATAAGACTAGCATGTTGGGGAAAACTTAATTGGCAAGTTTAGCCCATACCCATTCCCACCCCTATTCTTATCACAATTCCCTCAATCCAAATGGCTTCTTAGGATTTCCATTTTGCACTTGTTGGATTTGTGGCTTTTTGTATGGTTTTCCCAACTCTTGAAGTATTTGATAAAAGTGTCGCTTTGAGTTCATTTTAACTTGACACGTTGTCTATTGGATTTTGATTTTGTTTTAGGTTGTGGAATTCAAGTTCATATCTGAAGTGTAGATTATTCAACGCCATTGAGTTTCAGGCTCTTTTTAGTTTGTATCTCTTGTATAGTATTTTAATTGTTCAAACTCTTTTTTAGTAATAAAGTTTTTGTCTTTGTTGATGAATCCATTACTTGCTTTATGTTAAAACATTTGTATTTTAATAGAGTTACAACTCTTTGTCAGTGTTGTAATATTGCTGTGAGTGTATACATAAAATGTTATTTGTTTATATGGTCGACAAGATTGGAAATGTGTGGGTTGTATGCTATTTGTTTATATGGTTG

>cucumber_newGene_310 cucumber_newGene_310.1

TCTCCCCTCCCCTATTTTTCTACCCTAAAATGATATCACAATTAATTTCTTCAATGTTGTTTACACATGTACTAAAAACTCGATATTGAGTTTATTGAAATAATATCTATGAATAATTTCGATTGGATGTCACTATATCTTAATAACTATTATAGATGTGGATTCAAGTGAGAGGACTAATTACCCCTATTGCCGTAATTGACACAATCAAGCTATCATTTTGGTGTATAATATCTTGTGTATTTATTTGTCGTATACTTTCCTTCTATTGCTTTTGTTTGTGTATAATTGTACATGTAATTATGGATTCTATATACAAGAAAACATCCATATTCAATAAAAAGTCCACATGGTATCAGAGCCCTAATTTTCTTTTAGAAATTTTGCCGCGGCTAGTTCAATTCCGTCCGCCGGTCATCTCCCGTTCAACTCTCCGACGACCACCTCCGTTCAGATCTCCGACGAGATCTCTGAAGCAAGTCGTACACATCTGTCACTCACACCAGATCTGTCTGTGACTCTTCTTCTGTCCACGATCCCAGATCTCACACGCCTCAGTCGCTCACGCCACCTGCCAATCCTCGCTCTGCTTGTCACGCACGTCCGCCATTTTTCGCCGAACCATCTGACCATCAATCTTCCTAGTCGCCGCCCGAAGAGATCTCTGAAGCAAACCACACGCGTTCAACTAGATCTATCCGTGGTTCTAGTTCTGTCCACGATTTCAGATCTCGCACGCGTCTGCCACTCACACCAGCCGTCTTGTTTTTGTCCGAGAGTTGAAGAGTTGCCGTTTTCGTCCACAATTCCGTCCACGATTCCAGATCTCCTAGTCACACGCACGTTCACCATTTTCGTCCGAGCATCTCACCTTCAACCTTCTTGTTTTTGTGTAACCCAGGTCTAAAGTCCAACTCATTGCCAAAAAAAAAAAAAAAAAATCAGATCCATCCACCTCTGCACAATCTATTTCCGTTTCCAGATCGTGGTTTCATTGTAGTTCACAATCTGTCGCATGGAGAAACATGATGTTCCTCGTCCTATAAGCCCCATACTTGATGGCTCCAATTATATCACTTGGGCTCACCAAATGAGGAGCTTTCTGATCGGTCGAAAACTATGGCGTATAGTTATCGGCGATATCACCAAACCCACTCCTCCCAAGAGAACAATTAGTAAAGTCACCAATAAAGACATTGATGTCAATAGAGGACGACAAGTACATTGAAAAACTTGAGGACTGAGATAGTAAAAATCATCAAATCATCACTTGGTTGGGTAACACCTCTATCCCTGCAATTCATACTCAATTTGATGCATTTGATATTGCCAAGGATCTTTGGGATTTTCGGTACACAAGGTTTCAGTCCATTGGGCTTGCTCATTATTATCAATTACACTCGTCTTGTGAGTCTAACCCAGAAGGGAGGACAATATGTGAATGAGTATCTTGCCACCCTTCAGCCAATTTGGACTCAACTAGACCAGGCAAAAATTAGTCCAGATCATATTCGCCTCATTCAAGTCCTCATGAGTTTGTGCCTGGAGTATGAATCAATTCGGGCCGCTCTCTTACATCGCAATCCTCTACCATCTCTCGATGCCGCCATTCAAGAAATTTTATTTGAAGAAAAACGTCTTGGCATTGTCTCTCCTTTACCCTCTGATGTTGCGCTTGCAACCACCTATCTACGACCTGCGAATGAGACCACTTTCTGCAAAAATTATAAACTTCATGGTCATAAGTTTGCTAATTGTCCTACTATTGAGTGCAAGTATTGTCACAAGCGAGTGCAAGTATTGTCACAAGCGAGGTCACATCCGGGAAAACTGTCCCACTCGCCCACCCCGCTCTTCTGGTCACTCCTATAAACCCAAGTCTTCTCCTAAAACTGGTTCTCACCCTGTGATTGCTACTGCTAACCCATCTGATATTATTGCCCCTCAAAATGTTCAATTAAATGATCTGCATGACTTACTGAAATAGGTGATCTCTTCCAACTCTACTGCTCTTGCAGTCACTCCAGGTTCAGGATTCTTAGACGGGACAGGTGATTGGTACGAGACGGAAGGTGGGACGATTATTTGAGCTCACATCTCCTCAGCATTCTCCTATGTTCCCACTCATCTCAGCACCAGTCACTGATAATACTATATACTAGTGGCATCTCCGTCTAGGTCATACTTCCTCTGATAAACTTCATAATTTAGTTTCTACTGGCACCTTGAAAAATGTCCCAAAATTTAGTCCCTTTGACTATTTAAATTGCAAACTTGCGAAACAACCTGCTTTATCATTTTCTAATTCTGCTTCATTATGTGATACTCATTTTGGTCTTATTCATTTTGACATTTGGGGTTCTGCTTTATGTACTACTGTTAATGGTTATTGATATTTTGTCTTATTTATTGATGACTATTCTCGTTTCACTTGGATTTACTTTCTTAAACACCGTTCTTCGTTATATCAAATTTATGTTGATTTTGCGAATATGATTCACACACAATTCTCTAGCACAATTAAAATCCTTCGCGCTGACAATGCGATGGAATATAAAGACTCCCGTCTTCTTTCCTTCATTGCCCATCAAGGCACTTTGATTCAACGCTCATGTCCCCACACCTCCCAGCAGAATGGAAGAGCTGAACGCAAACACCGTCACATTCTAGACTCTGTTCGTGCTCAACTTCTTTCTACTTCCTGTCCTGAAAAAATTTGGGGAGAGGCTGCCCTCACCTCAGTCTATGTCATCAACCGCATTCCTTCTCATGTCATTCATTCACAATGTTTCCCCTTTTGAGCGACTATATGGTACTTTCCCTTCTTACTCTAATCTCAAGGTCTTTGGTTGTGCATGTTTTGTATTATTACATCCTCATCAACATACCAAACTTGAACCAAGGGCTCGTCTTTGTTGTTTCTTGGGTTATGGTACTGAACATAAAGGATTTCGCTGTTGGGATCCCATCTCTCTGCGATTACGAATATCCCATCATGTCACCTTTTGGGAACATCGTATGTTCTCTACTCTTTCTTCATTTCATGCATTTCTTTTTGGTCCTCACTTATTTTTCACTAATCCTTCTACTGACCTATTCCCTACTTCTTGCTTATCACTACACCTTGTCCTCTACCCATATCTGAGCTCACTCAACCAGAACCTATCCCTGCGCTCCCGGATCTTCCATCCGTCTCTTGTGAGGATTCTGCATCTGCACCGGCCCGACGGTCTACCCGGGTAAGAGAATCTCCTCCTCATCTTAAAGATTACAATTGTTTTTCCACTATCATGTCTTTGGTTGAACCTTCCTCATATAAGGAAGTCAGTACTAACTCATTGTGGCAGCAAGCAATGAACGAAGAACTCCAAACCTTAGAAAAAACTCATACTTGGGACTATGTATGAAAATAAGTAAGCACAAGGTGAAAATAAGCTTTTAATCTTGTCAAACTTCCCCTATTTAGTATTTATAGACTTTTTCAGTGAAGCCTCCCATTCTGGAATGACTACCTCTACGTAACGGTGAGGATCATTCAATCTCAAAACGCCACATGGCTCCTGCCACTCTTATTTTGAACTTGGTGGGCGACAAGGTCAAATCAATTCGTTGACCTTATGACATCAACTATTTTGTGGTGAAGGAAGCTCTCATTCAC

>cucumber_newGene_312 cucumber_newGene_312.2

CACAACTCATGTCTCTTCTTCTTTCCCCCATCACTTTCCCTCTCAATTTTCTCTCCTCCCAAACACCAAACACCAGTTTCCATATCCCTTTTTGATCCTCTTTGTATTCTTCCACGGCTTTCTTGAACTGCATTTCATTATCTCTCGTTTTCTTCGAATTATATCCTTTTACACAATACATATATATATATATATATACATATTCCTCTTCCTATGTATTTCAATGGCTGCAGTTCAATAAAGCTGTGGGAAGATACAATAAAAGGATCAACAAAAACAAAAAAAAAACAAGAATTCTTCTTCTTCTTCTTCAACCTATGAAGAACGACTTGTAGGGTTCCTTTACAAAAAATTGCAGGCTACAACCAAACAAACAGAAGAAAGGAACAAGGAAAGCAATAAAAAAGAAATAAAGAAGAAGAAATTTCCCCATTTGATGGAAATTAAAAAACAAAGAATACATATTATTTTTATAAGATGTAGAGAAAAATATATTAATTAATTATCTGTATTTGTACTACCATATATCTCTTCGTGTATATATATATAAATGTAGCTCTCTATAAAGTAGCAACAAG

>cucumber_newGene_313 cucumber_newGene_313.2

CAAAGCCCTAATCGGACCAAACCCTAGGGTTTACCCTAACTGGACTAAACCCAACTTTTCTTCCAAATCTCCCAAAAACCCATTTCCCTTTCTTCTTCTCTCTCTCACTGTCAGGCCCATCTCTCTCCTCGACCTTCCTTCTTCCTCTTCTTCTCCTTCTCCTTCTGCTCCTTCTCTCTCTATCTGCATTTCTGTTCTCCCTATTCTCTATTTCTCTTCACTTTTGAGCTCCTTTTTCCGGCAATGGAGTTGGACTGTGTAAGCTTCGTGGAGAGGACCATGTGGGTGCGCTCTGTTTTGGAAGCTATGGCGGTGGACACTGTTTTCACCACTGCAAACTCCCTTGCTTGCCTTCTGGCGGCGGCAGGAGCTATGTTAAATATTGCTAATGGTACACCAGGACTGGCAGCAATTGAGGAAAGGTTTCCTTTCTCTGAACTTCACAAAAAGGAAAGCCCTGATCAGGAAAACAAAGATGGCAGTGAAACGGAGGATGATGAAGATGACGACGACGACGATGATGCGAATGATCAGGATGACGACGACGATGATGAAGATTTCTCTGGAGGTGAAGAAGGTGATGACGGTGATCCAGAGGATGATCCTGAAGCAAATGGTGATGGAAGAGCTGGAGGTGATGAGGAAGATGACGATGACGATGATGACAATGGAGATGACGATGATGAAGATGAGGATGAGGACGAAGATGAGGACGAAGAAGACGAAGAGGAGGAAACACCCCAGCCACCAGCCAAGAAGAGGAAGTGAGAATATCGATTGGCACTTCACATTGTTGAGTTTCGAGTAGCGAGGGAAGTTTAGCTAGTGTTAGCCTTTGCTTTAGGGGTAGGAACATTGTGGGCCTTGTTGGTAATGAACTTCTCATCTTATGTTTGTGATGTTGTGGATCCATATTATATATAACAATTTCTTCTGCTGAATTAGTTAGTAGTGCTATGTTGTCAGAAGCAGAGATTTTCTTGGCTTCAAGCAAAATTCCATTGAGGAAAGAATAGCAAAATTGTGATTTGAGGCCATGGGGTTAAACAAGTTGGTTAGTTAAAATCAATGTTGTTAAACTTATGTTTATTTTTCCAAAACTAGAAAATTGATATCCTTAAATATTTAACTGAT

>cucumber_newGene_315 cucumber_newGene_315.1

TAGAGCTTCTGCTGGTGCAGATAAGCAAGAACTTGCAGTTTGGTTAGCCATGCCGACTGTTATAGTTTATGCTTACTTGTTCAATCTGTTTAGGTTTGCAAATTTTTGACATGGATGACCTCCAGAAATCCATTATTTTGTTGAAATGTTGTACTTTCTCCAGAAGAACTTCCATCACCACATCAATCATCAAATCGGAAGATGGGTTTTTGTAGATCGTTCGATGTAACTTTGTAAATTGATCAAGCATGGTTGGTTGGGCTTTAGATGACAGATCAATCTTCTAAACTTTAGACTTGATCCTTGTGGCCAGTTCTTTGGCTTCTCTGTGATTCAATCGATTGTTCATCATCCACAAGTCTCAGGCTTCCAAGACTGAAGCTTTCGACCATTTCTGCTTGTGATTGATTGTATCAACTGCCAGTTTGCTGTACATATTTCGAGCTCGTTTGCCAAAGTTTCCATGCCGTCAAGTACTTGTGCAATTCATCCTTCTTGTAAGTATGTTAAGCTAGCATTACCCTTTGTAAAGATTAAATTAGGCTCTAACCTGTCTGTTCAAACTTGTAAAGTTGGCT

>cucumber_newGene_316 cucumber_newGene_316.1

TGGGGACACAGCTTTGCTTCAGCTTTTCATGGAAGACAAATATGGGAATTTGATCCAAATGCAGGAACTCCCGAAGAGCAAGCGGAAATCGAACACTTACGCCAACGTTTTACAAAAAATCATCTCAAAGGTTTTCCTAGTGGTGACTTGCTTTGGCGCCTCCAGTTTCTTAGAGAGAAAAAGTTCAAACAAAGTATACCACAGGTGAAAGTTGAAGATGGAGAAGAAATAAGTTATGATAAGGCATCAAATGCAATGAGAAGAGGAGCATATTTCCTAGCAGCCATACAAGCAAGTGATGGACATTGGCCAAGTGAATCATCAGGCCCTCTATTTTACCTGTGCCCTATGCTAATTTGCATGTACATTATGGGTACAATGGACACAATATTGAGTCCTGAGCACAAGAAAGAAATGTTGCGTTATGTTTATAACCATCAAAATGAAGATGGCGGATGGGGGTTGCATGTAGGTGGTCACAGTAACATGTTCTGCACTACCTTCAATTACATCTCTTTACGTTTACTTGGGGAAGGACCTGAGGTTGAGCAACTTTCCAGATCCAGAAATTGGATACGACAACGCGGTGGTGTCACCTCCATACCTTCTTGGGGAAAGACATGGCTCTCGGTATAAATATCAATTTCATCTCCATATTATCAATTTTGAAATTTCAATACCTATTTTTATAAAGTCTAATATTAGTTATAGTACTACGGTACATTCTATTTCATTGATATACCAAATAGGATATACATCAGTTGACATATATATACCAAAATATGAAAGTACACCTGCTCATACACAAATAAATAGAGGGCTTGCAAAAATAGCTAAA

>cucumber_newGene_322 cucumber_newGene_322.1

AATAGATATATATTTTTTTTTAAATAAAGAGATCTTGTTCCTCGTCTTCGTGACAGAGCATTGCCGGAGAAGAGAGCAAGACCGAGACAGAAAGGGCGATATTTTTCACCGATTGCCCTCTCCGTCTCGATCAACCCTTAATAATCACAATACAATTAATCTTCCTTCTTTTTCCGTTGCTTAATCTTTGATTTCTTGTGATGTATTTCCTTCCATTTCTCTTTCCGTTAATCCCCTGTATTTGATGCGACGGTTGAATGCCCGTAAAAGGGACGATTCTAAAATTACCAATATGGATCCGACCAAGCTGCACCTTCGACCCAGCACCAGACCCAATTTGTTCGCCAGGGCCAACTCCAAGAACTCCAAATCCAAATTCTGGGTTTCTCTTGCAGCCCTAATTGCGTTTGTTTTCTTTCTCTTTTCTCTCTTCGTTTTTGCAAGGAATTTAAGGAGCTCTTTGAAGAGAAGGTATGGAATCGTTATTGATGGAGGTAGCACCGGGTCTCGAATCCACGTCTTTGGGTATAGAGTCGAGTCTGGTTATGGGGTTTTTGATTTTGGGGAAGAGGGATTGGCGTCTATGAAGGTGAATCCTGGGCTGTCGGCCTATGCACGAGATCCTGATGGAGCTGGGAAGTCTTTGGTTAAACTATTGGAGTATGCCAAGAGTCGGGTTCCAAGGGATCAATGGGAATTCACTGAAATTCGGTTAATGGCAACAGCTGGGCTAAGATTGCTGGAATTGGATGTACAGAACCGCATACTAGAGTCTTGCCGGCAGGTGCTTCGGTCTTCTGGGTTTAAGTTCCATGATGAATGGGCGTCGGTCATCACAGGCTCTGATGAAGGGACGTATGCGTGGGTTGCGGCTAACTTTGCATTGGGCACTCTTGGAGGCGATCCCCTTGAGACAACTGGAATTATCGAACTTGGTGGGGCTTCAGCTCAGGTTACTTTTGTTTCAAGCGAACCAATACCATCTGAATTCTCACGTACAGTTAAATTTGGCAATATGACATACACTCTTTACTCTCACAGCTTCCTACACTTCGGGCAGAACGCTGCTCATGATTCCTTAAGAGAAGGACTTATTACAGGAGAGTTCAACTCAGGAGCTAAAACTCTTCAAAACGGATTGTCCGTGGATCCTTGTACTCCCAATGGGTACTCTCATATTTTGGAGTCGGAAGCACTATCTCCAGGTTATATGGTGGAAAGGAATAGATATTTATCAACTTTCCATTCTAAGGGAAACTTCTCTGAGTGCAGATCTGTTGCCTTAATGCTGCTGCAAAAAGGAAAAGAGAAATGCACCAATAGAAACTGCTCTGTAGGATCCATTTACACCCCAAAACTTAGAGGGAAGTTTTTGGCTACGGAGAATTTCTTCTACACATCAAAATTCTTTGGTCTTGGATCGAGAGCTTTTCTTTCAGATCTGATGGTGGCTGGAGAGGAGTTCTGTGGTCAGGACTGGTTGAAGTTGAAAGAAAGATACAAGCTACTTGAAGAAGAGGATTTGCTGCGTTATTGCTTCTCATCTGCATATATTGTGGCCCTACTTCATGACAGTCTTGGAATTGAATTAGAGGATCAGAGCATCACTGCTGCAACTCAAGTACAAAACATTCCTCTCGATTGGGCATTGGGAGCTTTCATCTTGCAAAGCACGGCTGCAATAGAAGCAGAACCAGAGCAGTGGGATTGGATTGTGGCAATCTTCGGCTATGAGTTGCCCTCTAAGTTCTCACTTGTTGCGGTATCAATTCTGTTGTTGTTTATAGCATGGTCTGTATCAAAGTGGAGGAAACCTCAGTTAAAGACGATTTACGATCTAGAGAAGGGACGGTACATAGTGACCCGTGTGACTAAATCCTGATAAAAACTAGCTTACTAGTTAAAATTTCCCTGGATCGTTTGATTGCACAAGGAAAGAACACAACTACACTACACTACATAAAATTATTATACAATTCAGCCTCCATAGTGTCCCATAGACCAATTCTTCAAGTCCAAGATGTTTGATGAGATTGTATATTCTGTGATTTTCATTCACATCTCAAGTAGACGACAACTGTTGTAGAAAAAGCCTCCCTTTTAGCTTAGCAGGAGAACAGGCCGACGTTCCCATTGTCAGTTACATCACCGTTCCCATTCCCATCGCCAGTGCCGGCTTAGGGTGAAATGTGTAGGACTTACATTAGAAAA

>cucumber_newGene_323 cucumber_newGene_323.1

TGGGAGCTTTCCTCACACTGTCGACCAATCCACGCCGTCTACATATTTGCTTCATCATTCAACCCTTCTCCTCCTATGATTCTTCTTTGATACAAGATTATTCATCATTTGGTCCTTCATTCCATTCGTGCTTCTCATTTCTGGTTTGTTTCCTTCACTCTCTTTTCTCCCATTTGCATTTGTTTTTGCTCCTTATTGCCATGAAATACAAATCTGAAGGTTTAGCACCAGTTTTGTTTCATTACTTTCTCTTTTTCAATGATTATACCTATCAATGTCATAAAACTCTATATTCTTGTACTTCTGGGAGATTATCTCAGCTTGTCCTTTGATTGTTTCTAGGTATGATTATTTAGATTTGAGAGGAGTTGGTTGTTTGATGCAACTGAAGTTCATGTGGATTGAGGAAAAAATTATTATCCCAATTTTAGCAGTTGGATAGAAAGTGTCCAGGGATGCTCGTGGTGTAATATAAGAGTTAGAATGTTTTGTCTCTAAAAAACCATACTACTCAATATTAAGGTGAGAAACTCAGGCTCTCTAACAAAGTTGGTGCAAGTGTGGTTTACATGATTAGGTTAGGTGCGGCGTTTGATTTTGTACTTCTTATCTGTTGGAGGATATTTTGTGTTGGTCTTTTTTTCATATTTTGTATAACTTCAATCCTCTCTTAGATGCCGTGGCCTTATCCTCCATTTGGAAAAGTAAAATCTCCAAGAGGTCAAAATCTTTGTCTTATGCTATTTATATGTTTGTCATTCTTAATTGTAAAACACATGCACAATGGTTATATATATTTTATGGTCAGTTGTGACTGTGAGTGTCTAAACTTATGTATCACAAAATATACATTAAATGTATGTAAGATATATACATATATTATAGTATGTTTAGTATTCTAATATTGTTTAGATTTTAGGTACAAAAAGGTAGGTTCTTTTATAACATAGGTGTTGTTAATATGTGAGCATTTTTTGGAAATAAATTCAAGATGTGAGCTTTAGAAGGAAGGTTATGATTTGGTGGGAATTTCCTGTTGTCATTGTTAAAAAAAAATATTGTTGTGCAATTAAACATGTTATCCAAGGATCTGTCTATCATTACTTTATAAACTTTTTAATGTCATTTTTAAACCGACATCAAAGTCAAACTAACATTAAAGGCCTTTAGTAACACTATCAAAGATGTCGATTTAAAAACGATATTAAAGGTCTTTTATATCACTAGCAAACATCAAATACCTTTCATTTCTTCTAG

>cucumber_newGene_324 cucumber_newGene_324.2

CTAAATTGCCCCTAAGATCTCACTTTCCTTCTTCCACATTTTTTGTGCGTAAGAATTCATTGCTCTCTCCCTCCCTCCCTGTAGACTCCTCTATGATAACTCTCTCTCTCCCTATAAACTTTATTCTTAGTTTCAGCCATTGTCTTCTGCCATCTCACTTTCGTTTCGCTTGCCGTTAACGATGAAATTGCTACAGGCGTCGTCTTGAAGGTGTTTCGCTTTCGATCTGCAACCATCAATTTTAAAAGAGCAATCCTAATGTTATTACTATTGTCGAAGACACAGATGTGTTGTGGTTGCCCATTCTTTCTGATTCCTCTTATGATGATTTTTCTGTTGTTGTCGATCATGTGGCATCTTGTTCATCAATTTTTCCTATTTCGAGTTCCTCATCAAGAAGTTCCAATTGCACATTTGATGTCGTTGATTTAATAGACTTGGATTTGTGTGTTGTAGACTTCTACATAAAAGAAGGTTCAATTTTTAACTGCAAATTAAGATGTCTTAAAGAAAACTCTTTATTTGTTTGCTACTCAACATAATTTTGAGTTTAGGATTGCCCGCCCCAATCAAACTTCTCTTAAACTTTCTTGCAAAGACACTTCTTGTCAATGACTACCTTTTTTATTTATACAACACAAGAAGTAAATGATGATGACATATTCAAATATTATTTTATGACTATGCTGCTTCAATCCATGTATGGGAGCGTTTTTCATTTGGAATATTTATGAATTTTATGTAGCAAATACTTATAAAATTTGTTTTCTT

>cucumber_newGene_326 cucumber_newGene_326.2

CCTTCTCCCTCACTCTCTTCTACTAACTAAAATGCAGTGACTTCAATTTTCTCCTTTTTCAGTCGTCGTCTCCTTTACCGTCGCCCCGATTGTTCAAGCCTCTGTGGTGGTCAGTCCATGTGTGGCGTGCAAGATTCTGCATCGGAGAAAACATCTCTTGAAGAAACTCGGGTAGGATTAGAACTGGAGCTGCCAAAAGAAGAATGGGTTCCAATTAATCATCTTCTGGTATGTAATGTTCTTTTACAAATAGTTGTTGCCCAAGGTACCATGATTGGATGAATTTGATGACACTATTTTCTTGTAAACATACATAACCATATATTTTACTCATACGCAACAATATTCTTACTCATAATTGTGCATTTCTATATCTATACATTCACATACGGCGTAGAATGTTTACCCCTTGAAAAAACAACCTACTGCATCTATCATAGCACTAGGAGTGAAATAGGGTTTACATTGACAAAGTTCAAGTACACTTCAA

>cucumber_newGene_329 cucumber_newGene_329.1

GAATTGTGTATGATATGGTACAACACCACACACCACTATAGCATTCTTGGTGTTGTGTATATCTTTTAAAATAATTTAGGGTGTTTTTGTGACCAATAATAGCAGAATTAGAAGAGAAGGCAGATCCTCCAATAATGGTGAATGCCTAAAAATTTTTCTGATTGATTATCCAATCGCCATTTTTGAACAAGCGCGGGAAGATTTGAAAAAGAACCCTTCAAAACTTGTAGGGTTCTAAAACCAATCGCCACAGAGTTCATAATCCTTTTTGTTGTGACCTTCAAACAAATTCATACAAATCCATGTTTGCTCCATCAATTTTTCATCATTCACATGATCCAGATGAATCCGAATCAAAAGTTTCAGCTCATGGGTATGGCTGAAGATCGGACCATGGCCCGTTTTTACTCTATGACAATGAAATCGGACCCTTATGCCCTAAATTTTCAGAGTATTGATGGAATAAGCTCACCTAACAACGAAGGGAGCAAGCTTATCTGGAAATGCATTGAATAATGCGAATTAACGCCACTGGCAGTTGCACATGTTGCTTCTGCAATCCACTCTCTCGTCGAGCGTGAAATTGTTACATCATTAAACAGAGCTTCTTCAGCGAGGGAAAAAAAACTGGGTGGGAGGATTGGCACTCACAAATACTGAAGATTGAGTTTCCTAATAGAAGCCGAATAACCGAAATTCTTGTGATGACGTTTATCGAGTTAACAGAGAGCATCCTTTTGTGAAAATTAGAACAATAATACCCATGGTTTAGAGTCTTCTGCTGTCATTATCTTTATAATCTTTAGATCTGCATAACAGCAAACTAGTAAAGAGAATAATTATAGGTTACCTATCTTTATAGCCCGAAAGTCAGGGTCAAGCAGTTTGTGGAGGGTATAGATTGGAACCCTATCTCGGGTAGGGAGGATGAGGAATTTAGTGGTTCTGTTTACCTTGGATGAGATTGAAAAAGCTGTTTTCAACTGCGATAGGAAGAAATCTCCAAGCGCTGATGGCTTCTCTATGGCTTTCTTTCAGGAGTCGGGACAATTGAAATCTTATCAAAAGAGATTTGGAGGGTGTGCTTAAGGAATCTGTTGAAAGAGGAAAAGAGGAATGTTGAATAGTTCATTGGTGAAAACCTTCATTTACTTGATTCCTAAAAAGGATAATGCTAACAGGGTTAAGGAATTTCAGTCCATTAGATTCATTACTTGTGTCTAAAAGATTTTGGCTAAGGTTCTTGCTAATCAGTTAAGGAAAGTGATGTCGTCTACCATTCTCTAGGCCCAGGGTGCCTCTGTGATGGAGAGGCAAATCCTGGATCAAGCTCTTATATTTAATGAAACCATTGAGGTGTATAGATTGAGGAAAAAGGAAGGTGTGATTCTAAAGCTCGATTTTGAAAAAGTATATAACCATGTGGATTGGGTGTTGATAAAAAAAGGTTTTGATTATAAATGGAGGATGTGGATGTGGGGGTGTGTGAGGAATGTGAAATATTCTATCCTCATCAATGGAACCCTTAAATGGTTGATTCAAGCTACCAAAGGTTTAAGACCAGGGGATCCTTTGTCTTCTTTCTTGTTCTTACTTGTGGTGGATGTCTTAAGTCAAATCATTTATAAGGTGGTGGAAGGAAACATTATTGATCCTTTTTGGATTCGAAGGAATGAAGTCTCATTATCGCATTGTCAATTCGTGAATGAAACAATGTTATTTTGTTCTGGAAAAGAGGAGTTCTTCATCATCCTTAACCATATGGTGGCTTTGTTTGAAGATATGTCTGGGCTAAAAATCAATAGGAGCAAATGTACTATTTTTGGAATTAACACTGATCAAGTTAAGTTGCAGAGGTGGACAAAGGTGTTTGATTATGAGATTGACTCTTTACCTTCATCATATCTTGGGCTTCCTTCGGGTCGAAGCCTAGAGCTTTGAATTTTTGAGAAAATTCGTAAGAGATTGGTGGCGTGGAAGAAAGAGTTCTTCTCAAAAGCTCGCAGATTAACCCTGATTAGATCGGTGCTGAGTGGAATCCATGTGTATAATTTATCTCTTTTCAGGACCCTCAACTCAATGTGTAAGAGCGTTGAAAAGTATATGAGAAATTGTCAATAAATGAGAAATTTTCTATTGGAAGGGGTGAAGGAAGGTCATGGATCTCACTTGGTCAATTGGGAAGCGGTGGGGCACCCTATAAGTGATGGGGGTTCAGAGATCGGTAATCTAAGAATCTAAAACAGAGCTTTGTTGCCCAAGTGACTTCAGTGCTTTTCCCTTGAGTCCAATTCTTTATGGCATAGGATTATTGTGAGTAACTAGTAAGGTGGCAACTATCCTTTTGAATGGATGACGAAAGGGGTTAAAGGCACACATCATAATCCTATTCCTTGGAAGGATATCTCTTTCAAGCTTTCGACCTTTTTCTGATTTACTCGTTGCTTTGTGGGGATGGTAAGGATACTTATTGTTTGGAGGATCAATTGGTAGTGTTGTCTTCCATTTGTTCTTTATTTTCACGTCTTTATCATTTACCCTCTTCCAAGAACTCTATGATATTGGATCTTTTGGTTGGGTCATGTCCTTTCTTTGGTTGAGGGAAGCAATTTTAGGGAGGAAAGGAGGGATGTTCGTGTTTAGAGTCCCAATTCGAGTCAGAGATAACGTGTAAATCCTTTTTCAATATGTTGTTGGATCCCTCCCTTCCTAGGGAGTTTGTTTTGATGTGGTTTATAGGATCAAGGTTCCTAGGAAAGTCAGATTTTTTATTTGGCAAGTTTTGTTTGTGCTTGTTAGGAGGAGAACTTCGCTTGTTGGACCTTTTTGTTGCATGCTTTGTCGGAAGGTGGAGGAAGCGTCTTATCTCTTCTTTTTTAGATTGCCAGTATGCATGAGGTGTGTGGAGATTTTTTAACGAGAGTTTGCTGTTAGCTATGCTGGCCATCAGAGCATTTGTACGATGATTGAGGAGTTCCTCCATCTCCCTTCAAAGAGAAAAGGGGTTTTTTTATTGCTTGCAGGGGTGTGTTGTGTTCTGTGGTAGGGAAAGGGACCATATTGAGGTTTGGTCTTTGATTAGATTTTACGTTTTTCTTTGGGCTTCAATTTCAAAGATCTTTTGTAATTATTCCACTAGTAACGTTTTACCAATTTGGACCCCCTTCTTTTATTCGGGGTGATTGATTGTAACTATTCTACGGTAACATTTTACTAAGTTGGATCATCGATTGGACGAATGATTTTTTTATGACCTTGTATTATTTCATTTTTTCTTAATGAAAGTTATTTCTATAACACACGTATATAGGTCACCA

>cucumber_newGene_330 cucumber_newGene_330.2

AGAGTAAAAGGGAAGTTGGGGGAGAAGACAATGTAAAATGTAAAAATGGCAGTATGCATTGAGGAGGCGTGAAATCTATACTTCAGAGTGAGAGATTGAAATTTTAAAGGAAAATAATAAAATAATAAAGAAAAAGAATCTGAGATCCCGCCGTTAAATGCCGACGGAGGAATTGTATTCAGGGAGCGACGGAGCGATGGGGTGGGGGTGGTCGATCTTTGAGGGAGTCGTGGCTGTCGGTTCACTTTCTCTTCTTGGATGGGCTGGCTTATGGTTCTTGAATCGAAGGCTTTATAAAGAGTACGAAGAAAAGCAAGTCCTTGTTCAAATAATCTTCAGCGTCGTCTTCGCTTTCTCCTGCAATCTACTTCAGCTCGTTCTCTTCGAGATCATTCCTGTTCTTTCCAAAGAGGCAAGATGGATGAACTGGAAGGTGGATTTGTTCTGTCTCATACTCTTGCTGGTTTTCATGTTGCCTTATTATCATTGTTACTTGATGCTTTGCAATAGTGGTGTGAGGAAGGAACGGGCTGCTCTTGGAGCCATGTTATTCTTACTGGCCTTTCTTTATGCTTTTTGGCGAATGGGCGTTCATTTTCCAATGCCTTCTCCTGACAAAGGCTTCTTTACTATGCCTCAATTGGTCAGCAGAATAGGGGTGATTGGGGTCACTGTGATGGCTGTACTATCTGGTTTTGGAGCTGTAAATTTGCCGTACAGTTATTTGTCTCTCTTCATCAGAGAGATTGAGGAAGCTGAAATTAAGGCCTTGGAAAGACAACTAATGCAATCCATCGAGACTTGTATTGCAAAGAAAAAGAAAATTATTCTTTCCCAAATGGAGATAGAGCGGGTCCTTGGCTCAGAGGAGAATTCCAAGTCTAGATCTTTCTTTAAGCGCCTAGTTGGTACTGTTGTTCGATCAGTACAAGAGGACCAAAAAGAGCAAGATATTAAAAATATGGAAGCAGAAGTACAAGCTTTGGAGGAGCTATCAAAGCAGTTATTTCTTGAAATCTATGAACTTCGTCAAGCTAAGGAGGCGGCAACTTATTCACGTACCTGGAGGGGTCACATGCAAAATCTACTCGGCTATGCATGTTCTATCTATTGTGTGTATAAAATGATTAAGTCTTTGCAAAGTGTTGTTTTCAAGGAGGCTGGTTCAGTTGATCCTGTGACAATGACGATTAGCATATTCTTCCAGTTCTTTGACATCGGGATCAATGCTGCATTATTGTCTCAGTATATATCCCTACTATTCATTGGCATGTTGATCGTAATATCTGTACGAGGCTTCTTGACCAATCTAATGAAGTTCTTTTTCGCAGTCTCTAGAGTTGGGAGTGGATCTTCGAGCAATGTCGTTCTTTTCCTCTCTGAAATTATGGGAATGTACTTTGTCTCATCTATTCTATTGATTAGAAAAAGCCTGGCAACAGAGTACAGGTTGATCATAACAGATGTATTGGGAGATATTCAGTTTGACTTTTATCACCGATGGTTCGATGCAATCTTTGTGGCCAGTGCCTTTCTTTCTCTACTTTTGCTTTCTGCACATTATACATCACGGCAAGCTGACAAACATCCAATAGACTAACTCAAATAAGATTGAGAATCAACTGGGTAGCTTGGAGAAGACAAAGACCACGGAACACCACGAAACTGAATTTTCAGTTAGGCTGGATTCTTACCTTGTCTGGACGCATTGAGTGTGGTAATAACAGAGAAGGTCGCCGGGTGTGGTCTTAGGATGGGATTCATTCTTTTGTGCACAACTTCATCATGTATAGATAAAGGTTTGACTCAGTAGTATCAAACTTTGTTCCTTCAGTAGTAAAAAAAGCGTTATTCAATTTTATTGATTATGTCAAGCAGTGAAAAGATGTGTTTTTGATATGTATGCTTGGTATTTTTTGTGTTGTGAATTGTGATGTTCTGTAATGGGATGCCTCGTTTTCACAGAAAGGTATAGCAACAATTGTTTGATGTTGTGTTTTTGAGAACTTTAAGTGGAGGTTGATAGCATGTTTGGCAGGCAAAGCC

>cucumber_newGene_331 cucumber_newGene_331.2

CAAAGACCCATGTGAGTTTTTTTTGCCTATCAAGAAAAGGTTTTTCCATGGAGTTGTATGGCATCAGGGAAAGGTTTTTATTGAGTTGTAATACAGCTCCATCTCATTGCATTCCACTAAAGACTCCCTTAGTTGAGGTGGAACACAATGGAAAGGTTTAAAGGATAGCAAATGAGAATTGCCTACAGATTAGACCAATTCTGCTCCATCGTGAACTTCCATGTCAAAAAGATTTAGCAATCAGCAGCATCAGCGAAGCCATTCAACTTCATGCACAATTTATGAAATCAGGAAGCCGAATTCGTAAAGACCTCCCGCTTTTGTTCCTTCCCATGCATGACTCGACCCATCAAAAAATGGAAGATAGATTCATGGGTTGATTCTAGAAGTTGGTCTGACATTTAATTTGTTTGTTTTGTGCAAAACTCGTTGATTCACATGTACTCGAAGTTTGGCAACTTGGGTCTTGGATACGAAGTGCTTGATGAAATGCCCGTGACAGATTTAGGTTCAATTTGACTTTTGTTACTTACGGGCTCGTTGAAAATGAGTGTTTTCTATGAAGCTCATCGTCTGTTAATTGCATTTATTTGGTTTGATATTGATTAGTAGAGGGCCTGATTTAACTCTGTCGTTACCATCTTTCTGGGGTTATTCATTAGACAGAAGGCTTTATATATTGTCTTTTCTATTAAAACTTTTAGATTCTATTGAGGCTATTAGATTGTTTGAGCTACGCCGGAAGCTGAGGATGAGACTAGACGATGCAACGGTTGTTGCTCATGAGAGCATGCCCTGATATTGGGGATTTAATGATGGGAAGGAGAGTGCATTAAGGGTGTTGAAAATTGAGCATGATGAGAGAAGAATAACTACAACGATATTTTATCAGCATTTTGAATGCAGTTTGGGTGAAGGAAGGGTTGTTTCTTTTTACACAACTGCAAATCATGAATAGAGTTGAAACAACCATTCAACATTAAGGATGTATGGTGGACCTTTCCTTTCTGCCAAGGCAATGCATTAAAGAAGCTGGGGAATTTGTAAGAACTATTCCAGTTGAATCTGGCAATCCACTATGGTGAAATTTGATATAGGGACGTATTTGATCGAATATCTTGAGCTTCTTGACAAGAATGTTAGTTGTTTTGGGAGCAATAATTGGGAAGTGGCATGATAATTAATGCAGTGGTTAAGGATTGATGGGGTAATATCCATGAATTTGCAGCAGGAGAATCAGGTAATCCTAAAACAGGAAGTGCTGGAGGAAATAATATATAATCAACCCAAACAAGAAGGATTTGAACCAAAACTTCCGGAGATTTTGCTTTAAGTAGGGATAATGCAAAGTTCCTGAATTACAAGTTACAACACCACATTGAAAAATTAGCTCCAATGCAAATTCTGACATTCAGATTCTTGAAAGTTGGTTGGTCCTGTGAGAATTGTCACGAGTTTATGAAACTGGTTTCAAAGATATACCAAAGAGAAATAAAAATGAAGGGTAGAATCCGTTTCCACCATTTCAAGGATTGTTATTTCCTTTTCCAAAGACCAATGGTAGCAGGGAGATAGTTACTTGGTTGATGTTTTTCCTTGAGCAATATCGATGAGAACTGAGAAGCTCAAAAGTTGTAATAAGTTGCAAACTATGGATATCCAATATTATTAATCACACAACTGCAACACAAAACTTTGTCCCAATGATAAAAATACCAAAGATCAAGTATGTTTCCTGAGTTTTATCTTAAATACAAAGTATTTGGGGTTTATATCTCTAGTCTTCTCTAACAAAAGTAATTTTAAGAGAATTGCTAGGGCCCTCCACTTAATCAATAAACCTTGTTAGACTGGCTTAAAAGAATGCTACTAACTCTAGCGATTGATTATTTTATGCAACTATTCGCAAGCCTTTGTCTTCGTTTAAGAATTAACAGAATACTATTGAACTTGTGACTGATTACCTGATTACTTTAGTAACTATTTTCATAATGTCATTTCCTCAGGTTGTCTAGCTTATAGTTTAGGTTGGGATGGGACTATTCTTTTGTAAGGACACTCTGCTGATTCCATGTTGGATAGGATTCAAAGTATACTACTTGAATCTAGTGGTTGATTACTTTATGCAAGTTGCAACTAGTAACCATAAAGCATTCATCCCAATTCATCCTTCTCATAGGTCGCGTGCTCACTCAGCCAAAATACACTACTGAATCTAGTGACTGATTACTTTATGTAACTATAGACCATAAAGTGCCTTCGTCCTGATTCGTCTAGCTTGTACCACGCGCTCACACAGCCAAAATAAGTAGGTTTGTATTGAAAGGCATTGACCATGATATGTGGATCCATTCCTTTTGCTCTTGTAGTTATACTTCTAGAATCCAACTTGTCTTATTATATTAGGAATTTAGTTTGAATTCGGTTGATCTGATCTGTAGTTGCTATTGAGAGATTAACTTCTGGTACAAGTATTGAATCTTTTCTGTTCCTGCAAAGCTACTTTGATGCAGATATTGTATAAACAAGAATTATTAAGGGAACTGCTTGCGCTCAGATGCTCACATGTTTTTACCTAAGATTATGGAGAAAGGTTTCTTTACAGCCCACAGTTTGATTTTGGAGATTCCCACTCCCCCACCATAGCCATTTCTTGAGGTCATGCATTAAAGCTTCATTTTTTAAAGGTAAACCACTTCTTTTAGGTTGGTAGAATAGGCAAAAGATCATGCTATAAATTTTAATATCTTTAAAATCAGATCTCATTCCCATTGTTTAAAACACCCCCTCCTCCTCCCTCTCCCCAATCTGATACTCGAACCTGAAATAATTTGATACCATAGATACTCAAATGTTGTAAAAACAAGTAGGTTGTCACATGAGA

>cucumber_newGene_333 cucumber_newGene_333.1

GATATCTGGTTGAAGCAAGAGAAGGAAAAAATACATTACTCTGATGAGTCTACATTGAGTATTACTATTCAAGTAAAAGACAATGAAAGAGTTTGGAAGATTGATCCTTTTGCTTTCCGTCAAGTGCTTGGCCAAGGAGATTAGTACGCTTATTTGGATTTTTCTAGACAAAGGTCTAAATACAGCAGAAAAACTCAAACAGAAAATTGGCTGCTTTGCAACCTTTGATTTGCTTCTTGTTAGACATCCAACCCGTAATCGCAACGACCCAATTGCAAGAGCGTTTGAATGGAAATGAGGTTCAAAATCATATCTTTTATCAGTGTCTTTATGAGAAAGAGAAGACGAGCATGTGTATATGGTCTTCGACTTCATGCCTGCCAATGGAGAGCAACTGCTGGGCTTACCTTTTGGGGATAGGCAAAATTTTTGCTGATTATTGGGTCCTGTTTCTTCCCTGAACCATTGCCGCCAAAGCTTTGTTCGAAACTGTGGATTGGAAGACTTTATTGAGTTCCTCTGAGCAAAGGTTTGCATTGGCAAGATAGTCTTGAAGTAGCTGGTTGCCTCTTCTTGGTGTTCGCTTTCTCCTTTCATTTTGAAGATTTTGGTGTGCAAGACTTCAGTTTCAAGTGGGCTCTTTTTGAGAAAGCGTTTAAGTTTTTCTTTGTATCTAGCTTTCTTCGAATTTCGTTTGCTTTGATTAACTCATTGTTTTTATGCTTGAGATTGTTATTTTTGTTCTAGCTAGTTATTTTTCCGTAGTCTTTTTTGTTCTTAGTATAATACTCTAGCATTAGTCTCTATTATTATTATTAATAAAGAGGGTCGTTTCCGTTTAAAGAAAAAGAAAGTGAGGATTAGCGAAGGAATATCAATATAGGAGTTCGAGGGAACCTACTTGATTAAAGGATCACATGGGGTTGCCAAACTGGTTGTAACATACTCGTTTTTCTCAGCCCAACTTCTG

>cucumber_newGene_336 cucumber_newGene_336.2

TCAATACATGTATGGCTTGCACATGAACCCTTCAATCGATGTAGCACTTTTTTTGTTCCTGAACCTGAGTAGTTGCTGTAATCTTCCTATTCGTTTTAATAATTTGAGAGAATTGGTACATGATTATTCATAGTTCATCTTTATTGGCGATGATTGCATAATACAAACTGTTTAACTCTAAGGATTACAAATTGTCGTACATGTATTGTCGTAGGCTGTTTTGTTCTAAGGACTAGGAACAACCCAAGAAGTTGTTCAATTTGCAGTGGCCATTTGGTTGGACGTTCTTCTTCTCCTTGATCTCTTTCTCCTCTTCTTAGGAACTTCATTTTCATTATCAGATTTGAAGAGGGACCACCATGAGTGGGGTAGTAAAATGAATAGAGAATTGGAGAGTGAGCTGGGTTCAACTGGAGTCGACAAGTTCATGAGTTTTGTTTCAAATATCATTTCCAGAAACGAACATTCTCGGTTGGTCCAGTCAAATATATTCGAATGCTATTTAAGATATTTTTTGATTAAGGGCTAGTATTTCGTACATGTGGATTACTGAAACTTGTATTAAGATGTACAAATATTATAAATCGTATTATAGTGTATATATATTAAAGTGTTGGCTATTTTCTTGTACATTGGTGTGAAACTTGTATAATTTCAAATTTGTCATTCTCAGCTACATTATTGTCATTCTAATGGTTCGTGTAATGGTGGAGCTGCATGGAGAACAGAATAAAACTATAGGAAATTGAGAAATGTGATAGTTAATAGTTGTTGTAAATT

>cucumber_newGene_337 cucumber_newGene_337.1

GTTAAATCCAAGGAGAACGAGGATTCTTTCTGGTCTATGTCATTAATTTTCCGATTTTTTTATGCAGCATATGGTTATTAGTTATATGCTTTATAATCTTAGATGTTAAGTCGTCATTGGAGTTAATAATATAATATAGTAAAAGTAGCAATAAAAGAAAGATCATATAGCTGAAGAATTCACGTGCAGGACAAAGGTTTGGCCGAAATGTAAATTCGCACTTAATAGCCACAGAGAAGACGGAATTGGCGCTGGAGCAGCAGTCCAGCCTCAGCCACAACCGTAAAAACCTCAAAAGTTTGATGAAGCAATAGTTTGAAAAGGAATGAGAGTGGAGTTAGTGGTGGCATAATGCAGTGGAATTTGATTGGATGACAGAGGCAGAGGCAGGGGGAGGAACGTTGGCGTCGGTTATAGTGTTTGGTTGTCTGTTGTTTGCAGTGAGAATGTTGGATGGTGAGCCAATGAAGAAGACGAAGATGATCCAGCAACCACAACAGCAGTTTTGATATAATATGGACAATTTATTTTTATATATTAAAAAAAAAGAGTATCGGTGTCCATTGTGAATTAAATGGGAAGGGAGATGGAGATTCTGTTTGCTTGGTGAGCAATTACGACGGAAAATGACAGGTGGAAGACGTTAAAAGCTACCCTCGCCTTCCGGTTAAATCCTCTCTGGATAGGTATTTACAAAAGTTGTGGCTTTGGTCTGATTCAAATTTTCAAGGGGAAGGGAGCCCTCAACAGCCACACACATCTTATATAATCTCTCACTCCCTAAACGACTTGTATTTTTATGTATTCCACTTTTTCCAAAATTCATTTCAAGAGAAAAATAAATAAATTCTCTCTTTCTTTCTCGTGCTTTAGCACTCTACAGCATTCCCATCGCTGTCAGAACCATTTTATTGGAATTTATCATCATATTACTTCATCATAACGACTCAAAAGTAGTAATGTATTAGGTTTTTTAAGTATAATTAAATGAAGAGAGTTTTC

>cucumber_newGene_338 cucumber_newGene_338.1

CTCGCTATATAAATTGCGCCCTTGTTTCAGATTGGTTCATCGAATTGTTTATGCATGTTTTGGATCTGCGAAGAACTATTGGAAGACAGTCTCTTGAATGATCATTGCTGGATAGATGATTATTTGATTGTAACGGAGTTAAAATATTTAGATTGCACAGCATGTTCATGTAACTGCATCGTGTATATTTTTAGCTCCAACAAAGATACAACGGAGGTGTTTAAAGCCCTAGCTTGTTAGTATGAATGCTCCTTAGGTTGGGGTTACGCACACCACCAAATTTTTTCTATATTGTTGATTCTGCTCTGTTATCTTACAAAGAATTTTTTTTCTCATGATGTGGTTCGAATTAAGAGTGATTTTGAGAATATTTTTTTGTAATGTAATATCTAAAG

>cucumber_newGene_340 cucumber_newGene_340.3

TTCAAATCTGAGTACTTCCCTCCAGTTATTTTTCCAAAACAGTGAGAAGTTAACGGTCGACTGTGACAATTTTCGTACGAAATTGAAATTTCCCCAATTCTCGAGTATACTGAATCTTACCATTTCTTCTATTCGCCAAAACCTACGAATCGAGGAAGAAGAAGAAGAAGAGACCATGGGAATCTCAAACCTCTCATACGAGGAATGTCGTCAGAAAAGAGTTGAAGAAAACAAGAAGAGAATGGAAGCCCTAAATTTGCCCTTACTTTCTCAAGCTCTCCTCGATTCTTCCCCTTCCAAATCCTCCCCTAGTAAGCAGGTCAAAGCCTCGCGTGATGCAGAAACAACTGGTGGTGGTGAGGAGGTCCAGTCGCGTAGCAAAGCAACCAACGCCTGTTTACGCTGAAGTTCTTGTCGATCGTGTGGCGATTCCTAGAAGGTATTTCTACTGAAAACTGATTGGAAAAAAGATGAAAGATTCGTCATACAGTTGGATCGTTTAGCATTGTTACTAGCTTTCTTTTGTTCGGGATTATAGGATCTCCAGAGCAAGAGATTTTTCAGCCCAGTTTTATGCTTCGGATGAAGCAAGAAAAAAAGCCTTTGAAAGAGCTCTTGAGTTACAATCTGGATTGGAGCCGAATTATCCCAGCTGTATAAAGTCGATGGTTCGGTCTCATGTTAGTGGTTGTTTCTGGCTGGGCCTTCCTTCTCATTTCTGCAAGACGCACCTTCCAAAGAATGATGGAGTAATGACTTTAATAGATGAAGATGGTGATGAGTATCCAATAATATATTTGGCACGAAAAACAGGTTTTAGTGGTGGATGGAAGGGTTTTTCAATTGCTCATAAGTTAAGTGATGGAGATGCTGTGGTTTTTCAACACATTAAACCTACAGCCTGCAAGGTGTACATCTTTAGAGCGAATGGTTCCAAAGAAGAGAGTGATTCTAACAATAGCTAAATGATTTACTTTCTTATCCCAAGTCAGGTGATAATTTGAAGAGGCTTGGGGAAGATGTGCACATAATGCAACTACTTTTTTGCAAATTTCTTTTGAGTAAAAGGCAATCTGTGTCTTGTACAATCTTGTATCAGCTGAATTATATTTGGGGGCGGTGTGTGCATTCAAGTCTCCCTTGTTCCTAACATTTTTCTTCTCTCTTTTTTGTTAAACCCTTTTTTTTGTTTTAGCTTAGTAGACTACTTGCTATCTGATAGATTTTGCTGATTTTCATTGGGAAAAAGCCTTGAAGGTTTCTAGTGGGATGGCCCCCGTGTAAGCCATTGTTACAATAGCTCTTAAACTGGTTTCTTGAGTAATATTTTGTATTCCTCTCCAGAAAGTCCAAAACGAAGGGCACATTTGGATAGATTTCTAAAAGTAATTTTATTTGAAACACGTGAACGGTGGATAGGTGAGTCCCAATAGTTCTAATTAGCATAAATTTATGGAGTGTTAATTATCTTGTAATTTCAAATTACTAGAACTGATTATGTTTAGGGTGACATATGGGGTAGTGTGTATGTGTAATTGTTGATGCTTTAATTTGATATGTAACGATCAAATTAGCACATATATTATTTATTCCATACAATTGTG

>cucumber_newGene_341 cucumber_newGene_341.1

CTTCATTCTCACTGCCAATCTCAGGGAAGTCGTTTTTACTGTTTCCAGTCGCATTATCAGTGCTGCGCTTGGATCCTCAGTGATCTCGATGACAATAGTTCGAGGTTTTATTATTGAAGATTGAGTGTGAAAATCGGCTCTGATCTCTGTGTATCTTTCTTTTGCGTCTTCATTTCTACGTAGTTTCAAATTCTTTTTGCTTGATCTTATGTGTTTCTTAATCAGGAAGTCCAATTTGATTTTATTTCAAATTTGACTTATCTGTAATTGGAATTACTTCTGCGTTCGGTTTCTGAATTTAATTAGTATACAAATTTCTTGAAAGGAATCGACGACGATAGTTCAAGGATATATTCTTGAAGATTGATTGTGAAGCGTCACAGAATCGGATCTCTCTGAATCGACGACGATAGTTCAAGGATATATTCTTGAAGATTGATTGTGAAGTGTCACAGAATCGACCTCTCGAGTTGGATTGCATGATTGTCAACTCTCCAGCAAGGTAAGTTCCCTAGACTAATACTGCCAAGTCTAACAAATTATATAGATTTTAAAAAAATCATAATGAAGGTTATAAAAAAAAAGTTTCAACGAGACCACGTGGTTATACTTTAAATTTGAGTTTTCTGGTTTTCCTTTCAATTTGTAAAAGAAAATTCGAAAACGTTTTTTTATTTATGTAAGCTGAGGATAGCCGCAGCCCTAGCTTTTATTACATAGTTTAAGCTAAATAAACGTTTTAGTTGCAGAACTAGTTAGGAGTTTCTTTAACTTAATCCTATTGTCTAGAAAGTTTAATGTAGTTCCTTGTTTGTCTTAATGTTTTGTTTTCTAAAATTAGTTCTATTGGTTAAGACATTTATACGAGCATAAAACTTGTGAATTGGACCAAATTTTGAAGATAAAAATAGAGGGATTTCAAGATTTTGTTCTCTAAACAACATTTTCAGTCACTCGACTTGTGGTCAAAAGGGCGAGACCTGACCAAACTGAACTCTTTTTTCTCTTTTCATAGTTATATAATTTATTATTTTTTTTGGGTGAAATAAATCTACGAACCAAAATTCAATTTGGTTTGTCATTGCTAACAACG

>cucumber_newGene_342 cucumber_newGene_342.1

TTTTTCTCTCTCCATACCGACGATCCTTCTTCTTCTCCTTCCCCAATTTTTTTCAGATCATCCTTCGGCCGAGTTCCACTTCCGGCTCTCCTTCACGACTGTCGAAACTCCACCATGCTTTCGAGTGAACTTTAGACCATCTCCTCGTTCCAATCTCTTCTATTAACTCCCCATTCAAGTTTGTTGTTGTTCACACCAAACCTTTCCCTTCTTCCATCGTCAGTCTCCTCGGACCTGGAGCGGCTGAGGTTTTGCCCTATGTGGATTCGAATTTGAAGAAGATTGCTAGCTGAACTCCAAAATTTAAACCATTTTTTGTGAATAAACTCGCTAGATCGGTAATTATATGTGATATGTTCCAAGGACTGGATGCAATGTGGATTAAGCGAAGTCTCTAACGCCCTGCTACTCGAGTTTGTGGATTACCGCTCCCTTCTCTGTCGACCATGCATTGATACGGGTGGCTCGATCCTTCTAGCGCCTGCTTTTGTTCTACGCATCGTTTTGGTTTCGGGATTTCTCTCAAGTTGGTTTTGAGTTTGTTGTGTTGGCGGTGGTTGTAGACACAATCGGGTGGCAGAGGAGGAGACTGCAATGGTTGAGGGAGAGAGGGTGGAGATTCAGCCGGTAATGGAGGAGGAGGAGGCTGCGACGTACGCCACACATAGTAGATGTTCGGGTGGTGGAGGAGAAGGTTATTGCAACCACAAATGGTGAGATGGTAGAGATTAAGGTGACGGAGGATGTTATTAATTTTTTAAAAAATAGTTTTGTAAATTTTTATGTGTAATCATAATTATATTATACTGTCCC

>cucumber_newGene_343 cucumber_newGene_343.1

CGTCCCTCTCCATCGCATCCTTTTCAAACGCAGCGTGGACTTTTTGGTGAAGAACACAGATTCTTGTTAGAATATCAATGGCTGAAGAAGGGCAAGTGATTGGAATTCACAATGTTAAGGAATTCGACGAGCAAGTACGCATAGGAAAGGAAGCGAGAAAACTGATTGTGGTGGATTTTACTGCTTCTTGGCGTCCACCATGCCGCTTCATAGCTCCAATATTCGCAGAGTTGGCTAAGAAGAATCCCCATGCCATTTTCCTGAAAGTGGACGTTGATGAAGTTCGAGCAATTGCTGCGAGGTTCAACGTTGATGCAATGCCGACCTTTGCTTTCGTGAGAGATGAGGTGGAAGTTAGCTCTCACAGGATCGTCGGTGCTGACAAGAATGGGCTGATGGACAAGGTAATTGAGTTGTGCGGTCCAGTTCCATCAACTTCTACTGCATAAATGGTCAGCTTGGAAGCTCATTCTGCCTCTTATTAATATGGTGCATTGTTGATGATTCTAATTTCGAGTTTTTTCATGGATTTCATGACAATATGTCTGAGATGTGTACTTGTTTAA

>cucumber_newGene_344 cucumber_newGene_344.1

CAAAAGGCAATGTGCGGCCGAATGCCGTACTAGAATCAACAATAAAACCAAAACCACTCACCGCTCTTCCCTGCCGATCTCTTCGTCCTCGCCTTCGCCGCCGTATCCGCCGGGGATTAACCGAGCTATACCCTGGAGCCTTACGGAAGGAAACACTGAAGGAGACTTGTATTTTCTAACTGCTTTACTTGGCCATTGCTTAGAGTGACGGTGGAGAGAGAGAGAGAGAGAGATGCTTAAACGGAGAAGGAGAGCAGTGGCGGTGCTCAGGAAGTTGCTAACCTTTGCTATATGCTTTATAGGCTTACTAGCGCTGCTTTCTGTTCATCTTCACTTTTTCCCGTCCTCCCAAGTCCCTGGCTTCTCCGATTCCCACAAGCTTCCTGCGCAACGTGAATTCAAACACCAGAGGTTGAGCACCGAGAATAGCTGGACTCAGGAGATTGTTCCCCTCCATCTGTCTAAAGCTCCTGTTGCTTCTCGCAAGACTTGACAGTTGGGCAGTGCAAACTTGGTTCCGGGTCTGGACAAGCTATGGAAGCCTCCATCAAACCGCGACTTTTTACCATGTGTGGATCCCAGTGTCAATTATACAGGTAAGAGTTTTACTATGTTCATCTTGTTCCAAATAAAGATTCCCTTGTACTTGATCTTAAAATTTGTGTTGTTTATGCAGCACCTATGGAGTCAAGGGGTTACCTTCTAGTTCATACAAATGGGGGACTTAATCAAATGCGTGCTGGGATATGTGACATGGTAGCTGTAGCTCGAATCATTAATGCTACACTTGTAATTCCAGAACTTGATAAACAATCGTTTTGGCAGGACACTAGCAACTTCTCGGATGTTTTTGATGAAGATCATTTTATATCTGCTCTTGCTGAGGATGTAAAAGTGATTAAAAAGCTTCCTAAAGAATTGGCTACTGCTACAAAGGTAGTTAGGCATTTTAGAAGTTGGTCAGGTATGGATTACTACGAGGATGAAATCGCTACCCTTTGGGAAGAATATCAGGTTATTCGAGCTGCAAAATCTGATTCACGGTTAGCAAACAATAATCTACCTTTAGATATTCAGAGGCTTCGCTGCCGATCTTGTTATCAAGCCCTCCGTTTTTCACCTAAAATCGAAGCGATGGGGAAATTATTGGTGGATCGAATGAGGGCCCATGGTCCTTATATTGCTCTACATTTACGATACGAGAAGGACATGCTTGCATTTAGTGGATGCACACATGATTTATCTTCTGCTGAAGCAGACGAGTTAAGGATGATTAGAGAAAATACTTCATATTGG

>cucumber_newGene_345 cucumber_newGene_345.1

TAACGACATGAGGCTTGATCTTAAATAATAAAAGTCAAACATTTAGTCTCTAGTACCTGTCATGTCCTGAAAATGTTGCATGTGGTGTTATACTTTCAACCCAATTATTTTTCCAATCAAGGGATTCCATCATATGGTATTAAGCAATTTTTTAGCTAATTGATCTGATATGCTCAAATCTATTTCAATCACAATATTAGTTCTCATGATGACATGCACAGGAAGTTTTGGTAGCATACTATTGGTAACTTGTCAACCATTTTTCCCTTAACCTTTTTGAAGTTATTTAATTCACCAATTTTATTGTTTCTGATAGTAGGTTGTGGTGTCCCTGGGAAGTGGACCATGGGATCAGCTGTTTGGTGGTGGAAACTCTCCAGCTTTTGCTGTGGCAGCACTTGCAGCCTTTGCAAGTGGACTCATTGCCATCTTGGCTCTTCCTCGGTCTGGTGCTCAGAACCCCAGAAACCTCACATGAGAGAGTTCTGCTGTTTGATACTTACCCTTCACCTACATCAACTGATAGAGCCAAAGACACATGGGATCTGACATGGGGTTGTGTTATGAGTTTCAGAGAAGTAATATCTTATACAAAGGAAACAAATGAAAAACTGGTCCTTTTGTGCAGCTGCTGCTCAGAGTGATAAAGAGAAGTTTCTGGCTTGTTGATGATGGTAGGGCTGTGTGCCTCAGCTAAGGACACAGTCCCCCCAACTTCTCTTCCCTCTATAGAGACAGCCTCATCTGTGTTGGGAGAAAAGGGATACATATTTAATGCTTTTAATTGGTTCACCTAAATCTGTGTGTTCCCTTTCTCCCATCTGCTCCTTCTTCACACAGCCATTCCACTGTCACCTTCCCCATTAACATACATACTCTCTTCTCTTATCAGGTGGTCCCATTCTTAATACACAGCAGCAACAGCAGCAACAGCAGCATTACCCATGAAGAATCCTCTTCTCTTTTTTAATCATCTATAACCAAATAAGATGACAAACTCTTCTTGAGGGTTCTTTTTTTCTTCCTTTTTTTGCATTTTCAAGTATCCTCCTTGTGATGATGGATTTTGACCCCTCCCTCCCTTCTCAAAAATCCACTCCTAATCTCCTCTCTTCGGTTGTTTTTATTGTTATTAGGCTATAATTCTTCCTCCTCCTCCTAAGCCCAAAGGTTTTACCCACCTGGAATAACAACAACAATAATAATAGGTCCAAATCAACTAATGGGGTAGAGATGTTAGCCCATTTCAAGTCTGGATTCCTGCAAGAAAAATACAGGAGGGAAAGAGAAGTAAAGAGGTGTTCCATCCATTGTAAATTGTGATCATCTCATGGCCCCCACCTTTTTTTGATCGTTAGTCTAAAATAGTATTGTAATTATTGATTGGAATGATGTTATTAGGGGTTTAATTTGTCACAAAATCTCCAAGAGGGGATCACAAAATCTTAACAATCCTATTCCACTTCCAAAGGGGCTTAAAAACAATCTCCACCCCCCACACCATAAGTGTATGTAGA

>cucumber_newGene_346 cucumber_newGene_346.1

AGGATATGCAGGTAATAACAGGGAAATACACTCAACTGTTCGGATGTTGAGGGTTTTTGCTTAGCGGTTTCCATCTCCAACCCCTCAGTGGAAATATGAGGTGTCAAAGCTCTTAGAATATTTCCACGAAGTTCATTTTCCAGGTTAGCCACTCTTCTCTGACTAATTTATAGGTTTCTTTTCTTGCTAAATGGAAAATTCTGTCTCACTCGAAAGTTTTAAATAGTAGCCACAAAATGACTCTATGTCCCATACCAGTAACTAGTGGCCTCCAAAAATGATAACAATCTTTTCCTGAGTTTGATAATGGATGTTTGGTGAAAAGGTGAGTTGAAAAATAAGTTTGGAGCATATTACCACTCTACAATGCCTTCAAGTTTCCATGAAGGGACACCTGATTGAAGAAAAGTTTGAATCAAATCATCCTAGTCTAAAATGCACCTAGAATTTTCTATGAAAAGAAAGAAATTAGGATGAATATGATGTAGAAGAGCGTGGTGTTTCCTTCTTTTTTAGACAATGGGTGGGTGTGTGGATCTTGGGATAGCATATCCTTCCTTGAATGGCCACTTCACCATGACAATTTGGTGAGATGGTATTAGCCAGCTTTTCCAAAAAGCTAGTTTCTTTTCCCAACTTAGGCACCATATAGTTGTGTGGAATAGATGAAGAGTTTTATAAGGTTGAAAACTAACAAAGCCTGAATTATGAAGAGTTAGAATGGTCTTTCAAGCACTAAACAGCCAATACATGGGCTTATGTGGAAAACACAAACATATAATCCAAATAATTGTCTGCCACAACATCCCACTTTAAATTTTCAAAAATTTTAATTAACAAACGCAACTACCCATTATACTA

>cucumber_newGene_347 cucumber_newGene_347.1

TGTCACGATAGCCCCCAAATTTCCCTGCACCGTTCCGGAACTGCAATTTTGTAAAACTTTGATTTAGGGTTTATACCTCCTTCCATTCTTCAATCTCTTCGTCTGCTTCTGCTTTTTCTCTGGCGACAATGGCGAACGAGCAACAATCGACAGGAACCTGGACCCTCGAAACAATACCTTTCATTATTCTTATTCTCATAGCATCACACATCTTCGCTTTGGTATATTGGATTTATAGGTTAGCAACTCAAAAACAACCTCCAAGGAGGAAGGCACACTGAGTTACACTACGATTCGGTCTTGGAAGAAATTTTAGTGGAACCCTAATTCATTATTTGTTATAATTTATGTCACTGAGGCAGTCATAAAAAAGAAGCCATATAACGATGTTTCTCTGTAAGTATCCGT

>cucumber_newGene_348 cucumber_newGene_348.2

AACATTCCTAATTTCAATGTGCATCTCTATTGTAATTTGAGAATTAGTCTCATTATATTATTTGAATGAAAGGCTGGTTTCCTTTCACGAAAAAAGAAATTGCTTTTTACTTCTCCAAGTGACCAAATTTTCCCATACAAAGGTATTCGGTCTTTCGTCAACTTTTGAGCTTCCTTAATCAACTTTTGTGAATACTTCAACGTCTCTTTTCTTTGATTTCTTGATGTATAATCCTTCACCAAATGTTCCTTTTATGTATATGAGGATCCTATAAACAACTTGCATATTTTCTTTAGTAGGATTGTTCATAAATTGACACACAAAACTTACGTAAAAACAAATATCACTCTGGTGTGATTTAAGCAAATCGGCCTCCTAATGAGTCTTTGATATTGTTCCATATTAGCCAATGTGTTTCTTTCCCTAAGGTGTAGTTAGGATCATTAGGAGTATTAGAGGGCTTACAACCGAGCATACCCTTCTCTTTGGGAGGTCAAGGACATATTTTCTTTGAGAGACACACATTGACACAAACCTTTCCTTGATCAAGGCACTTCCATCCCTAGAAAGTATTCGTGAGTTGAAATTGAGAAGGAAATAGTGCTGTTTTTATCATTCTATCTAATGAATCAAATATGTCTTCTATTTGATGATTCATTTTTGGGCAACGACCTCTGATATTTCTTCCATCTGCACTTTCTTTGTTTGACTGTCTTTTATTCTAGTCTTGTTAGAGTCGTTTATATTTTTCTTACTGGTTTGTATTGGCCGTGTTTAGCCCCCAAATCGACTTTATTTATTTATTTTTTGTTTGGTCCTTTATTTTGTTGGATATGATGAGGGTGTTAAGGGGTGTCAACCTAGTTGAGATGCTCGGTTGCACCTCCTAATCTTTGTTTGGTTTTGCAATTTTACTTATTGTATGATTCTCTTGTACTATGAGCTTTTGTCGTATTGGTTATCAGTCTATTATCAATAAATGAGACTGTGTTCTTGTCATAAAAAGAATCAAACACGTCTTCTAATATAGAAGTCTTAGAAGAAAGACTGACTACAAAAAATGAAAAAATCTTAGACATAAATGATGAATATATACAATAATAAACATCAAATATTGCAGGAGATAAAAGGAAATCTAACACTCCCCCACTAGCTGGTTTGTATGATATCGGCAGCCAGATTTCTAAGAAGTTTATCAATTGTCTTTCCGAAAGACCTTTAGTTAGCATATCTGATGTTTGTTCTGTCGTGAGAAGATAACTTATGCATATTACATCTGGATCAACTTTCTCCTTTATGAAATGTGTCAACTTCGATGTGCTTTGTCTGATCATGTAGGATTGAATTATGAGTTATAGCAATGGGAGCCTTGTTATCTCAATAAACTCATATAGGTGTCTTTTGAGACACTTTTAGTTCTTAGCAAACTGTCCTAGTCTTGTTTTCTTTAAGGAGCACCAACCTTGTATCATGAACCGACTGTTCCTCCTCATCCCTAATGTGTTCAACTGAAAATGGTTCTCAAGAACTAAGTCCAAATGTGAGTCCTCAAATGATGCCAACTCCTTGAAGCACTGTGAAGACACTCAGATTTCATAGGAACGCTCGTACACACTTTCTTTTTTAAAGAGTCAGAATTATCTACTAAAGACTTGGGAGAGGATTGATTAACTATTCTCTGGGTATAATAATGCAAGGGAAAAGCCGGAGGATACCTCTCAAAAGGATCTAGAATCTGTTATTGTTGTTTGATACTGTTAGCAATTTATTGAATAATTGCAAGGAGTTCATTCTGAATTTTCCATTTACCACATATAGTAAAAATTAATAGCATATAATCATTGTCAGTCTTATTTAGTTCCTTCCTAACTCTTATTTTTCAGTTCAGAAGCTGTCTCCAATTCTATTGGAGAAACGGCGGTGACGCTTGAACCTATTCCAGCATGCAGAGAAGACTTTTCTAGGTTGAAGTTGACATCATTTGATAAATTGATTGAACGAACTTGTGGTTCAATAAAGAATGTAAGGCTTTGGTGTGAGCCTGATCAGAAACCTGGAGGTAGTACAGGAAATTCCAGCATGGCACCTGATGCAGCTGAAATTCAACTCAAAAGCCCGAATTGGCTGTTTGGTCAGAGGAAGTATGAAGAAAGTGGCCCTGGTTCCAAAGGTGTTTCACATGAAGCTGCAGTCTGTATGACTGAGGATAACATAAAAATTGATGGCTTCTATGATTTAAACAAACTTTCTTCTACTGACAATTTCAGTGATGAGGAAATCTTCCAAAGGTACCTTGCATTGACATCAGTTGAAGAAGCCAACGGTTGGTATGGTGGTACATTACTTGGCGATCAAGATGAAAGCAATGAGATATATAAACACTATTCTGAGTTATGTGAGGGCCCTGCTATGATGTCTTTCCAGAATGATCCTGAAAGGGAGATGCACTATGCTGATCTTTTACGCATGGGTGCAATTGATATGATTGACAATGCTTCTATTGAAGAGGACATGGAGGTGGCTTTGAAGGAGTATGATGAAATTGGTGTGGATCTTGGAATCATTCCTTCACCGTGCAAGTTCTTTGCCGAAGATCCTAGTTGGTTGACTAGATGGATTATTGGAGAAGAGAAGCTGCAAAGGATATAAGCTGAGAATGAACTCCGTTTGCATTAACAATAATTAATTTGTGTCTGCGTGTTCTAACATTGGGAATAAAGATCAAAAGTACCAGTAAAGGTAAAAATGGAGATAGTGTAAAAATGTGCTTTCGTTTTTTCCAAGTAGGATCAACCATGTTGACTTGCCTATCTCCAGAACTTTGTTGAAAACGTCATTGAGATATTGCGGCAGTCTTCTGTCTCAGTTTTCGGCATGTTTTTGGGTATGGTTCGGAGCTCTCAACAGGCTGAAAAAGCTAGGGGTAGAAGTTGCTTGCTGACTAATAAGCAGAATGTCGAAGTCGAAAGTTAACTATGTTGGTCATAATAAATATGAAACTAAGAGATGCCTTATGATGATAGCTGCTTTTCAGCTTGGCCTCTATCCCCCGTCACTCCACTTGCATTGTTTTATACAAAATTACGGACCTATTCGGAATGTGTGTCAGATTGTTTGTCTATTATTGTTACTAGCTTATCAAATTACTCGAATATAAATATGACACAAGAAGTGTGGTATTGT

>cucumber_newGene_349 cucumber_newGene_349.1

CAAATAAACACTAACACGAAGCATCGACGCATACACGGTAGGTATTATAGCTATAATGCATAAACGGTGATGAGAACCAATTCCTGAACCCATCCACAGCCATCATCATCGCCAACGTCGAGAAATATGGGTAAGGCAGAAGGTTCCAATCCGCCTCCGCCACCCTATGCCAAAGTCCACCCCTCAAATGACCCCGACGTTGATCACAATTCATATTCTCTCGAGAAATTCAGACTTTACGAAACCAGAGCGAGGTATTATCTGATTGGGAGCGATCGCTACAAGAAATTCTTCCGAGTGTTGAAGATCGATCGCTCGGAACCATCGGAACTCAATATCAGTGAAGACCCGGTAGTGTATTCCCTGCAAGAAATCAGGAACCTCCGTCAGCGAATTGCTGAAGGGAATCGTGCTACAGGGGGGTTAAGCCCGGTTACAAAGGCTTTTGGTATTGCAGGTTGCATTAAATGTTTGGAGTCTTATTATTTGATTTTGGTTACCAAGCGTCGGCAAATTGGATGTATATGCGGTCATGCAATATATGGTATAGATGAAACCCAATTGATTACAGTTCCTCACGTCTCCGTCCAGACTGATGTAGCACATTCTAAAACTGAGCTGAGGTTAGACTATCAATCTTT

>cucumber_newGene_350 cucumber_newGene_350.1

CGGAATCTCAGCAATTCCATTATTCACGCATTCCCTCACGTCATTAAATCAAACCTACTATTTTTCGATAATTCAACTTTAAATATTATCCGTTGCTCAGTGGTCAGTGCGCTATGGCGTCGGTCGGGGAGCAAATGTATAATTATGTCTATTTTGTTTCATTGTTGTCTAAAATTTATAAATACCCAAAGGAAGAGCAATAAACAAAGAAAGGAGCAAAGTTAAAGCAATAGAAATGGCTGACAACAACAATGGTGCGTCTGCTCAAACTCCTACCATTCCTGATCTCCTCACTCCTTACAAGATGGGGAAGTTCAATCTTTCTCACAGAATCGTACTGGCTCCATTGACAAGGCAGAGATCTTACAACAATGTTCCTCAGCAACATGCCATCTTGTATTACTCTCAGAGGTCTACCAAAGGGGGTTTCTTAATAACTGAGGCCACCGGAGTTTCTGACACTGCTCAAGGGTATCCTGATACACCTGGGATATGGACAAAAGAGCAAGTTGAAGCTTGGAAACCAATTGTGGATGCTGTTCACCGCAAAGGTGGAACATTCTTTTGTCAGATATGGCATGTAGGGAGGGTTTCAAATTCAGGTTTTCAACCAAATGGACAAGCCCCAATCTCATCATCAGACAAGCCGTTGTTCCCGCAAGTTAGATCCAACGGAATTGATGTTGCACAGTTTACCCCTCCGAGACGTTTAAGGACAGATGAAATCCCTCAAATTGTCAATGATTTTAGGCTTGCGGCTAGAAATGCCATTGAAGCTGGTTTTGATGGAGTTGAAATCCATGGAGCTCATGGCTACCTTATTGAACAGTTTATGAAAGATCAAGTGAATGACCGAACAGATCAATATGGAGGGTCTCTTGAAAACCGTTGCAGATTTGCATTAGAGGTAGTTGAAGCTGTTGTAAATGAGATAGGTGGAGACAGAGTGGGGATAAGACTATCGCCGTTTGCGGATTTCATGGAGGCTGGAGATTCGAATCCAAAAGCTTTGGGAGTTTACATGGCGGAAAGTTTGAACAAATATGGAATTCTGTACTGCCACATGGTGGAGCCTCGGATGAGAAATGTTTTGGAGAAAGTACAGTGTCCTCACAGCCTGCTACCAATGAGAAAGGCGTTCAATGGTACATTCATAGCTGCTGGGGGGTATGATAAGGAAGATGGAAACAAAACCATTGCTGAGAATCGAGCCGATCTTATTGCCTACGGACGTTGGTTTTTGGCAAATCCAGACTTGCCAAAGAGATTCGAGATAAATGCTCCTCTCAACCAGTACCATAGAGATACTTTTTACTTATCAGATCCAGTTGTTGGTTACACGGACTATCCGTTTCTGGAAGAGTAACAATTCTCATTTGGAGTTCCTTCCATTTAACTTTTTGAAATAAATAGTTGGTCTCTATTTATTTTGATGATTATGTTTCCATTTCTTATGTATAACGTAATGTGCGT

>cucumber_newGene_354 cucumber_newGene_354.1

CTCTAATTCTGCGCCTCTTGTTTGTTTGTTCCCTTTTCAATTTCCTCATGGCCTTCTTTTCCGTCCTTGCCCTAGATCATGGCACACTTTGCTGCCTACCTACATTTTTCCTTCTCACACTATATTTAATTTTTCCTAACTACTATCAACATCATTTTCATTTTCCCACTTCAAAAAACTAGTTGCCAAACATTTTTGGTTTACTCATCTGAACCTCTCGACACTTCCAAGGGATGTACCTAAGAATTGTAGGCGTCATTTTGATTGGATTATCGGCATGGGCATTTTCAGCTTTTCGTCCTCCAGCTCCTAAAGTCTGTGGTTCTGTTGGGGGTCCGCCGATAACTGCACCGAGAATAAAACTTAGAGATGGGAGGCATTTGGCTTATAAGGAGCATGGTGTCCCAATAACAGTTGCCAAGTATAAGATCATATATATCCATGGCTTTTCTAATAGCAGACATGATGCAGCAGTTGGAATTTTTCCTTCTCCAGGCTTTCTTGAGGAACTCGGAGTTTATGTTGTCTCTTTTGATAGACCTGGATATGGAGAAAGTGACCCGCATCGAAAGCGAACGGTAAAGAGTTTAGCATTGGATGTTGAAGAACTTGGTGATAAACTGGGACTGGGTCCTAAGTTTTATGTGGTTGGCTTGTCAATGGGTGGCCAAGCTGTCTGGGGTTGTCTTAAGTATATCCCACATAGATTGGCAGGAGCGTCTCTACTTTGTCCAGTTATCAACTATTGGTGGCCTAGTTTTCCTGCAAACTTGTCCAGGGAAGGTTTTTCCAGCCAACTACCACAGGATCAGTGGACACAACGTGTTGCTCATCATCTTCCATGGCTAACTTATTGGTGGAACACTCAGAAACTCTTTCCTGCCTTGAGCATTTTATCCGGTAGGCATGAGATCCTTTCCAGTCAAGATCTTGAAATCATTCGCTCTTCTCAAAGGCCAGTGGATCGGGAATATGTGAAGCAACAAGGAGAATATGAATCCTTCCACCTTGACCTGATGATCGGATTTGGGAAATGGGAATTCGACCCGATGCTTCTTGAAAACATATTTCCTAAGAATGAAGGATCCGTTCATCTATGGCACGGCAACAATGACCAACTCGTGCCTGTTAAGCTACAACGTTATATCGCTCAAAAACTTCCTTGGATCCATTACCATGAGTTGCCTGGTGCAGGCCATTTGTTTGCATTCACAAGGAAAATGAGTGAAGAAATTCTGAGGTCTATGCTTGTGCAATGAAAGGAAAAGGCATCTTTTAATTGTTGTGTCGTATCCGGAATTTTCTTTAAGCTACAGCTCTTTCGATACGTAAACTTTAGAATATGCTTTTATGCACTTTTTTCTCTATTGTACTTTTATGTTCGAATGTTGAAACAAAGCAGGTTGATTTATTA

>cucumber_newGene_356 cucumber_newGene_356.2

TTCTTTCTTCCACTTTTTTATTTATTTTCCTCATGGCTTCCCTTCTTCTTTTACTTTCTCTCTCTCAAGCTCAATTTTTTAGTATATTGGAAGGGTATGCAAAAGAAAAAAAAAAAAAAAAAATCCTTTTTTAAGGACAAGGTTGTCCATTCTTTCCTCTTCTTGTTGAGTCTTGCAAATGAGAGAAAGAAAAGAGAGATAGGAAAAGGGGTGTTTGGAATGGAGTTTGCTCCGAAATCTTCTTCTTTCTTTTCTTTTTTGTCAATCCTTTTGAAAGGAGAAGAAATAAAACTACATGTCAATGTCTTTTTCTTTCATTATTTTTCAAGTTTTCTTTCTTGCTTTCTTCAAAATTGTTATTCTATGTTGTTTTTTTCCCTAACTAAAAGTAGTCTTCTTCTTCTTCGTATTTGAAAAGTTTGTTGGTGTTGATTGAACGGAAAAAATGATCTCGGACCAGGCTTCATTCCTCACACGTACCTTTTCCTTTAGTAAATTTTGGATATTGGAAAGAACAAAATAGCTTTGCTCAACTTTTCTGCAACTATTTTGTCGTGGATTTTGTGGCGGACTTTGAGTATTGACGCCTCAAGTTTTATCTTATGGATACCTCATCACCGGCCAACCTCCACACACAAGGGCTTCCCTACTCGATCTTCTAGCTTGCCAACAACCAAAACTTCAACGCATAGGTCCCTCTCAACGCAAGCTCCTCTCAACCCTTCATCAAATATTCATTTCGACCAGCACTTAGCCACTTCAACGGCAATGGCTTCTCACCCTACCACCTCTAAAACTCCCAAAATTTGTATCAGTGTAAGGCATCACACCAATCTACTCGAATACTTTATATCCACAACTTCTCAACGTCATTCTCATACTCCTACAATAACTACACCACACTACTGCTGCATACCAAATTTCAATATCTCTAATAAGGAATTTGCAAAATCTGTCTTTGGCATCTCAAAATGGCTAGAATAGGCATATCAGTGCATGATCTCAAATGGGTATAAAGGTTAGCAATTTGCTTCCTTGTATTGAGAATATAATGCTTTTGGAAAGAGTTGAATTAAATCAATTATTTTATAGACTGGA

>cucumber_newGene_357 cucumber_newGene_357.1

GGAAAACGAGAAGTTTGCTATTTCTCGCAATTTTCTAATCTATTTCTTCTTCCTTTGTCACTCACGAACAGCCCCACCCTCCCTTCGACGGGCACTTCACCTCCACTGAACAACCCGCGCCGCATCCTCTGTTGTCTGTCAGCCATCCTGCACGTTGCCGTTAGCCACGTTGCATTAGATATGGATAGCCGCGCCTGGTGTCTGCTTCTAACCGAGCTACGCCGCCGCATTTCTTCGCAGCTGCTCAGTTTCGGCCACCGAGAATTGAGGCCCAACGTCGAATCCCTCTCTCATGTTACAATTTGTCATTGGTGAAGCTTGGTCTATGTCGTTGAAGCTATTTTTTATTTGAATTTTGGAAGAGTTTGAACTGCTATCCAGCTGGTTGGATGCTCGAAATTGGCATTTTGGCACGAATGGGGTAAAACTGAAGTGGGAAGTGCTTATAGTCCTAGAGTATTGAGCTATTACTGTGAGTGGTTTATTTCTAAGTTTTCAATATAAAAGTTTTACATATGCTACTATATGATTTATGATATTACTGGTAAATACCGTGTCGTGAATTTCTTTATGACTATGTTTTATTTACGTTTGATCCATAGGTTTACTTTCCATGATATATGGATGTGTATGACTTGAATTTTATGAAAACATGTTTTTGATCATACTTGGTTTTTCTTTTAAAACATGACTAATCGATGCTTATGAGAAATGAAAAGTTACTTTCCC

>cucumber_newGene_440 cucumber_newGene_440.2

GGGAGAGAAGGATCTTGGTTCCAGGGCGTTTGTCGTCCCAGCTAAAAGTGTAGTCGTCTTCTTCGGCTCCTAGTATATGGCCATTGGACTGTATATAGCTTAAATGAATGCTGTTTTTTGAGGCTTTGTAAACCCACGAGGCAGCCCATAGAAGCTCATCCTGATACAAGTCAATACGAACTCAGGAAAAAGAACACCTTGAAGGAAAAAAAAAGATTGAAAACAAGTAGATGTATGGTCATCTTATCTTCTTACTTACATTGTATCCCGAGTAAGAACAGTAAAATGGACAGACCACTGAATGGAGTGAATCACTGTAAGAACCTCTATGCTTGTCTGCTAAATCGAATACCTGCATTGCATTAAATGGGAACTTGTTACTTTGTTTAGAGCATAATGACATGCAATGTGTATGAGGAATGAGAGTATGATTTACTTTTAAGGCTGCGTCCAGTAATTTGTTAGAATAAGAAGGGTCGGATGCTTTGAACACGATTGAAGCTGCAGCCAACGCAGCTGCGGTCTCTGCTGCTACATCAGAGCCTGGGTTTTGAGCAGTTATCTTATACACAGTACGTGGCGTGTCCATGTCTTCTGGCCTTTCCCAACATTTATGATCTAGGTTTGGATCTCCCACCTTCACACAAGTTTACTACTTACGTTAAAATCATTCTTGTCGTAGTGATTATTATTATCTTAACCATCACATTCTTTTTCCTTTCCTCTCTTTCTCTCACAAATTTGTTTACGGTCACTCTACTCACTTGAACATATAAGACATCAGGTGCAGCAGTAGCAGCTTTCAATAGATAATCCGACCCCCAACGAACTGCTGCTCTTGCATTCTCAATCTCATTCCCCATCGAGTCGCCAAACTCAATGACACTCCAAGCCAGCAATGTAGTAGTAAAGGCCATTGGCAAGCCAAACTTGACATTATCCCCAGCATCATAGTAGCCACCAACAAGGTCAACCTGAAAAGTTCATATTCACATCCCATTAAGTTAGAGCCTAAGTTATTTATCAACTAGATTAGACTTATAGAAGTTACAAACATCACAATAAGAAAAATGGAAGTAAGCACACATGATAGGAGGAGCCATCTGATAAGGCTGAATCAGCTCTCCATGTGAGACGTTGGTTAGAGGGCAGCTTTCCGGATCGCTGTCCCTCAAAGAAAAGAATAGAATACTGAAGAGCAGTAGAATAATGTTGAGAAGTGAAAGCAGAGGAAGATAAGGTAAAGAGAGACAAAATGAAGTAGAGAGCCAAAGTGATTGAGAAAGACATTGTAATATCGTTATAAGTTGTAATATAACACACTCACTTGGAATGATGAATGAAATAGAGGTGAAGTGGGGTGTTATTTATAGAGAAAGAAGTTTAGTTTAATGTTGGTTCAGAGAGAAATTGTTCACGGGATCAACTGCGCCAAATTGGAAGTTAATAGGTGAGGTAAAGACAAACCAACTTTACCGTTGGGATTGGATTGGATAAAGGAATGAGAAAGTCAGAGAGAAAGAAGAAAATACTTAAGTATTGAATTGAATTTTTGAGGTTATTGTTTAGTATTGAAATATTAGGAAAAGTAAGGCGTAATTTACGTGGAAAAAAAAGAAGGGGGAGAAACTTTGGATGCCAAAATAAGAGGCGGAAATACAGAATTGAATATAGGATCTCAGGAATTTGTCTGTATTTGTAAAATGAGAAGAGTCACGGGTGTTTTTATTTTTTCAGGATGACTTTGCTTTGGTTTGCTCAGCTCAGCTCACATGAGTTTCTCTTTTGCTACCAACAATACAAAAGGTGACCTGCCAAGCTTACGTGCAACTATGGTCACAATTTCACAGACAAAAACACAAGAGTATTATTTGCTTCAAATGGTACATGCTTGGATCCTCCAATAAGGTTGTGCTTTGTGTTTTTGTTAAAGAGAAAAAACCAACTGCTGGCTTTTTGTTGTTGGTATTGTTGAAAACACAATGCCAAATTGTTGGTTTATAAGATTAGTTGTTTTCTGTACAAGTTGGTTAAAATATTTAGGGGATATCATCACTTGGTTGTTGGCTCAAATTGACATTGAAAACCGACTTGTTTGTTCCATCGGTTGGAGATTGTTAACCTTGGTTGAAATCAACGAGAACTCCAACGAAGTTAGAAAAACACCAAGAAAGAGGTGAAGAGGGTATGTTTAACATATACTAATTGAGTTGTTCGCGAATCATGTGGTTGGAAAAGTGTATTTCAACCTCATTGATTGAACCATTGACTACATGGACAAATTTTCTTCCTTGTATAACTCTTTTAAAGGTAATAATTAAGTGTATAACAAC

>cucumber_newGene_441 cucumber_newGene_441.1

TCAACTTATACTTTTATTTATTGAGATACTATACCTTATGTCAGGTTCTGAGATGATAACTTAAAGTTTTTATTTGTAAGGCTGTGTCTTCTCTTTTACCATAAGGCTGTTTCTTCGCTTTTACCGCATAGCTTTTTAACTTGCATTCGCTTATGTGTCTTGAGTAAGTAATATAGTTATTAAATTGTAGTGTTGGGTTACCACAGGACCATGGTTTGCCACTCTGTTGGAATTTTGTGCATTCTTTGCATTCATAATGAACCGGATTTTGAACCTAAGTTGTCTTCATTGGATACCGTGGTGAGGTTTGTGGTGTAGTTTTGAATGTTTCATTCCTAGCATGTTATTTCATATGCCAACTTATGAGTCTGTTAGGTGTTATCCTTTGCTTTGAAAAAAACCACATGTTTTGGATGTTTATAGCCATGTTTTATGATCGAATTCAATGATCTTCTAAGTAGATAGAAGGAATAGAAATTTAAATTGAGTCCTTGCTTTAAAACTTTTCGTAAGCTTTGTACTTGAGGGGGTATTAGTCCTGAACTTATAAGTATTAGGGTCAAGATGTCTCCAAACATAGGGCCTAGGGGGAAGATGTACTTAAATTGGAGGCATTTTTATTTGTTATGTAAAAACAAAGAATTTAGAAGATCTTGTAAAACATTATTATGGATGTCAAGAAATAGGGCTGACCTGTTGGATCATGCTTAAGTTGATGGATAAGACTCCAGTCAGGTATAAAGCTTCGGTTATGAGCTGTTAGACCAACTGGAGATCTCTAGTTCTAAATTTAGTGACCACTTATTTACCATTGGTTAAGGTTTAAATATAGAGGGCGGATTCTTGATTATTCAAGGGTTTCCTTGTGAAAATTCTGGACTCAGCATCTTGGCTGCAATCCCTTACTTCATGACAACCTAACTAGGAGTATTTGGACTGGAAACCACTCCCATCACGCACATACACACAGATTTAAACTAGTGCTTGTAGTTTCCTAACTGTGGAAGGGTTTTATGGTGTAATGTAAACAAAAGCACTTCCCTTTATATGGCTTTTGTTCAACTTTTC

>cucumber_newGene_442 cucumber_newGene_442.3

GGAGAATACACTTCTAATTTGAACATATTCTTTTATTTTGAACTTTAAAAACAAAGCATTTTAGGGTGCGGTGGATTCCGGCTGAGATTGGCGTATAAAATCACTGCTTTTCAAATGTAAGGTCGTTTTTGTCGTTTCCCCTCTCTTCCTCTCTTTCTCCACTCTCGCATTTCTAATTCTCACCTTTGCCCTCACGTACACGCCGTAATCGTCGACTTCTTCCCCATCCTCCCCGTCCTCCTTATTCCGGTGGAATATCCTAGCATCTGATTTCTTTGCTGAACCAAGCATCTTCTGGGTTTTGTTTGCTTGTATTTCAGGCCATCTTACTCACAAACTTCTCTAAATTGATTTATTTAATTGATCCAATAATGAATTTTCTCTTCTTGTGATGGCAATTTCTTGGCTGCCTTTGAATTGAGTTTCCTGATGTTAAAGTCACTTGTTGTTGTTTATTTTTTGTGGGTTTCTTGTTGTTAAGTTCAATTCATAGATTGCTGTTTGTTATTGTTGGCTTGTTGTATTGAACGGATAGCCTTCAACCTAGTTTTTAAAACCAATGATTCTTCTGGATTTTACCTTGTTCAATACAATCAACAGTTTTTTATATATATTTATATTTTTTGTTTCTGTCCCTTGGTAAAAGTTCACCTTGTGTGAATCTTTGCATTGCTTTTCATCTGATATTGTTGTCGTTAAGTATTTTAGTTTCCGAGGTTAATTTGTTCTACTGTTTCTTCAGGTTGATTACCTAATGCATTGAACCCAGAAATTATTGATCTTATATTAGTGTACAGTAAAAAGAGACTAATCTTTGGATCAATTTAGGCCTCAAATTTTCAAGGATGAGTGTCAGCATAATTCTTAAATTTGGTTGTCTCAGAAAGTTATGAATATTATAAGCCATTCTCGCGCCTAATTTATTTAAGAACACTTAGGATGAAGTTCAGCATTTGCTGTTCAAATAGAAACCAAAGGCCTTCGAGAATGATTCAATTTATAAGTTTGGATAATTCCTAAAACCTCCCGATGACCTCACATATAGAAAAATATAAATATAAAGGAACCGTAGTTCACTAGTGCTGCAATGTCTTTACACATTTTTCTTGAAGTAAGACTATACCAAGCCTCAAAATTTATTAAAAGAACCTAGGTTAGGTTTCTTTCACTCCTAATTATTAGTCATGGAGTACTAACTGTGATCAATCTTTTCTCTCTATGTGGGTGGCATGAATTTCCAAAGAAATTAGACGATATCAGCTCTTGCTCGAGAACAATAGAATTTTCAAATCTGTTGAAGGTCCAAATTGAATGCTGAGGAAATTAACCTAACAGATCGAGTTGCTGAGAGTTTTCGCTGCAAAGTCAATTTTTGGGGAATTAATTCACTCAAATTGAAAATTTTCCATGGCGGCTTCATATGGTCAAATATCAGAAACAGTAAGTTCCATGGAGATCCCTTTGCCAGTTGATAAGTTAGCGTTGGATCTGGTTGGCCATGACTCCTCCTCTGGCCCTTCAATCAAGCCTAAAATGCTTGTGGTTCTTGTAGCTACTGGAAGCTTCAATCCTCCCACTTATATGCATTTACGTATGTTTGAGCTGGCAAGGGATGCACTGAAAGTAGAGGGCCTTTGTGTTATTGGAGGTTATATGTCTCCAGTTAATGATGCATATAAGAAAAAGGGTCTCATTTCTTCTGAACATCGAATTAAGCTTTGCAATCTAGCATGCCAAAGTTCAGAATACGTCATGGTCGATCCATGGGAGGCCAGTCAAAACACCTATCAACGCACGTTAACTGTTCTGTCTCGAGTCAAGACTTCGCTGTGCGACCATGGATTGCTACCCAAAGAATCCCTGAAGGTGATGCTTGTTTGTGGTTCTGATTTGCTTCAATCTTTTGCAACACCTGGAGTCTGGATACGCGATCAAGTTAAAATCTTATGTCGAGATTTCGGTCTCGTTTGCATTCGAAGAGAAGGACAAGATGTTGAGAAAATCATATTGGATGATGGAATTCTAAACGAGAACAGAAACAATATCAAAATTGTAGATCAAATTGTACCCAACCAAATAAGTTCCACCAGAATAAGGGATTGCATTTCAAGAGGGCTGTCAATCAAATATCTTACGGCAGATGAAGTTATTGAGTATATAAGAGAGCAACATTTGTATCTGAATCCTTGATGTTATGTTTTTTGTTCTTTTTTGATAATCACCTCATTTTAGAGATGCAAAGGAGTATCTTAATAAGTCAATTGTAAACCCCAAGAAATCCAACGTTATTATTCCTATTTCTTTTAAAAATACACCAACTTATAGTTGTAATTATTGTATGTATTGCATTACCACAG

>cucumber_newGene_443 cucumber_newGene_443.1

GTTCAATATGCCTTACTTTGGCACGATTTGCAGCAACATATGAAACAAATATTAAGCAATAGTACCAATGGAGATTCTGTTCTAGCCAAGAGATAAATTCGAAGAAGTTATAGAAATGAAACCAAGATTGAGGAGGGGATGTGATAGCTCCCAAAATAAGGATCTTGAGAAAATCATTCATTTCGGAATTTTGTGGGCTGTATGGAGTTAATTTGAAGCAATTAGGATCTATACCAAACATTCAAACTTTAGAGATGACGAAGACGCGACACTTAATACAGAAAAACATCAGATAATAGTATAAAACGCCATTGGCTCCATGCACAGAGCTGGCTAAACAACCATAGGGCGACAATGCTGCCTCAAATTTTATATATAGATCCCTTCATTTAAGGTTTGCAGAAAACAGGAGGAGGAGATTGTTGTAGCCAATTTTTATAAGGAGCCCTAGGTTTGGTATGTGCCAAAACTAGTGAGATTGTAATCAATTTCTTTCTTTTGCATTGTAATGAAGATTAGGGCATCAAGCTTCCAGCTTCAATGACAAGCTAGGACCTCCAAACGTTGATTTTGATGTTTGTTGTGCATTTATTTACGATTTAATTAAATTTCGATGAATGTTTATGGTTTTTTT

>cucumber_newGene_444 cucumber_newGene_444.2

GAAAAATCTTGAATTTCATTTTAATTTCTCTGGTGGCCGGCAAAATGAAACCACAAATATACGTTATTTTTGGGCTTTGCTCATAACTCTTCACCTCCAGGGTTTAACCTTCGTTTCAGTCTCTTCAAGCACCCCAACTATCTACCCTCTGCCATGGCCGTGCTCTCTACTGCTCCATTTTCAGACCCACTTGCCTCAATCAACATATAAAACAAACAAAGCCCTCTAAACTTTGGATTATCCCAGCACCCTTTCGAAACCTTTCAAAACGGCTCCTTCTTGCACCAAGTTGGGCAGAGTTGCAGAGAAGATGACTTTGGGAATCTCCACTTCCCAAGCCTCTTCGTTTAGAACTCATTGGCCTTCTTGTTTCTTTCTATGGCGGTCTACCTCTGTTCCTAACTCTTCTATTTCCATTTGTGCCTCTTCCAAGGCTCTCTGCAGGACGGAATTTGCTTCCCTGAAGAGTGTGGGTGGCGCTTTGCCAAATATGAATGTTATTCACGCTTCCTTTCAGTTTAGACAATGCTCTTTCTCGACCACCACTTCATTTTATGAAACTAAACAATATGGCAAGGAGAGGCCATTACATGGTGCCGGTGATTGGGTAAATGAAACTTATCAGCTGATACAAAAGAAGACAACCTTTAAGTCTATGCGAACTGGGATCCTGACAGACAATGATGAAGTAAAGCTAAGAAAAAAGGAAAATCTCACCGACTATGGGACTTCACATTGTAGTAACAATCTCCGGCCTCCATATTCTAAGGTTTCATCAAATTTGAGAAGCAAACGCTCTAATGCCAGTAATGTTTCTGATTATGTAAACTCTTCAACTAATATTTTATCAGATGAATTCAGAAAACAAGAACCTATAAATTCTGAACGAACGAAGAATGTTGTAACTATAAATAGGATGGAAAATAAGGCACCTTTACTTAAGACAACTGAGTTTTCAAGTGGTCAATGTAACGGAGACAGTAATTCTTCTGCGGGGAGATTGTCCATGACAAAGCCTGAAAATAATGATTTGTATAATCAGGGTGTTCTTATGCAAAGTAAGAAAAAGTGTACCTCTTCTCAAATTGGAAAGGGATCAATTGTGCCTCTTGTACCAGATGTTTCACTTAATGGCAGAAACCAAAGTACCTCTTTGGGAAAGGTGAATAGTGTACCGAAAACTTTGAAATTTACAGAAGCTGCTAATGGGATGGAGGGGAGTGTAGCTGTGGAAAAAATGTCAAAACGGATCATCAATGGAAGTGGCACCAAGGTGATGGAAGCACCTGCAACTGCTTGCAAGCCAGATATAAAGGAGAGGCTTATTGGTGTATATGATAGTGTTCTTGTTGTTGATAGTGTATCTGCAGCAAAGGAAGTTGTTTCAATGCTTACTACTAAGTACAGGAATCTTGTGCATGCCTGCGATACTGAGGTGGCCAAGATTGATGTGAAGCAAGAAACACCCATTGACCATGGTGAAGTAATATGCTTCAGTATTTATTCAGGACCAGGAGCAGATTTTGGGAATGGAAAATCTTGCATTTGGGTTGATGTTCTTGATGGTGGCGGGAAGGAAATTTTGCTTCAGTTTGCACCATTCTTTGAAGATCCTTTGATCAGAAAGGTCTGGCACAACTACAGTTTTGACAATCACATTATTGAAAACTATGGTATTAAGCTTTCTGGCTTCCATGCTGACACTATGCACATGGCACGGTTATGGGATTCATCAAGACGAGTGAGTGGGGGATATTCACTTGAAGCTCTTTCTAGTGATAGAAAGGTCATGTCTGATGCTGAATTGGGAGAGGAAAAAGAGTTGATTGGTAAAGTATCCATGAAAACAATCTTTGGCCGGAAGAAGAAGAAAATGGATGGATCTGAAGGCAAACTTGTAGTCATTCCCCCTGTTGAAGAACTTCAACGAGAAGAACGGAAACCGTGGGTATCTTATTCTGCTTTAGATTCAATATGCACATTGAAGCTTTATGAGAGCTTGAAAAATAAACTGTCTCACATGCCTTGGGAGAGAAATGGAGAAGCGATTCCAGGTCAAACAATGATAAACTTTTATGAAGAATATTGGAAACCATTTGGTGAACTTCTTGTCAAAATGGAAACGGAGGGAATGCTGGTTGATAGGTCATATCTTGCTGAGATAGAAAAATTGGCCATAGTGGAACAAGAGGTTGCTGCTAACAAATTTCGTAACTGGGCTTCAAAGTACTGCTCTGATGCCAAGTACATGAATGTAGGAAGTGATGCACAAGTGCGGCAATTGCTCTTTGGTGGTGCCTGTAACAGTAAGAACCCTGTGGAGTTTCTTCCAACTGAAAGGACATTTAAAGTTCCAAACAGTGAAAAAGTCATTCAAGAGGGGAAAAAAACTGCCAGCAAGTTTCGGAATATCACTTTGCATTGCATTAAGGATAAGGCTTTTTCGACAGAAATTTACACAGCATCTGGTTGGCCTTCAGTGGGGGTGGATGCTCTGAAGATCTTAGCAGGCAAGGTCTCTGCAGAATTTGATGACATCGCTAACGACTTGTGCTTTGACAATGAGGTTGACAAGGATTTTGAAATGATGCCTCATGAAGAAAGCAAAGGGCATCTGTCTGATAATGATACTGCTTTGAAAGAATTTAAATCATTGGAGGAGAGCAAGGAAGCTTGTCATGCTATTGCTGCTTTATGTGAAGTCTGCTCTATCGACACTTTGATTTCAAATTTTATCCTTCCCTTACAGGGAAGCAATATATCTGGTAAGAATGGACGTGTGCATTGTTCTCTAAATATCAACACAGAAACTGGCCGCCTCTCAGCTCGGAGACCAAATTTGCAGAATCAACCGGCTCTGGAAAAGGACCGGTATAAGATTCGTCAGGCATTTATAGCTGCTCCTGGAAATTCCCTCATTGTTGCTGATTATGGCCAGTTGGAACTTAGGATTCTTGCTCATCTTGCCAATTGTAAGAGCATGCTGGAAGCCTTTAAAGCTGGGGGAGATTTCCATTCAAGGACAGCAATGAATATGTACCCTCATATTAAAAAAGCCGTTGAAGAAGGAAGCGTGCTTCTTGAGTGGGATCCTCAACCTGGGCAAGAAAAACCTCCAGTTCCATTGTTGAAGGATGCCTTTGCTTCTGAAAGAAGGAAGGCTAAAATGCTTAATTTTTCCATTGCGTATGGCAAGACTCCTGTTGGCCTTTCCAGAGATTGGAAGGTTTCCTTGGAGGAAGCAAAGAAGACAGTTAAGTTGTGGTATAATGAAAGAAAAGAAGTTCGTGAGTGGCAAGATCTTCGAATGGCAGAGGCTGCAGAGAGTTCATGTGTTCGTACATTGCTTGGACGAGCTCGTCAGTTTCCTTCAATGAAGTATGCTACTCGTTTCCAGAAAGGACATATAGAAAGAGCTGCTATCAATACTCCCGTGCAGGGTAGTGCTGCTGACGTTGCCATGTGTGCCATGTTGGAAATATCTAAAAATTCACGTTTGAGGGAACTTGGATGGAGGCTGCTTTTACAGGTTCATGATGAAGTAATATTGGAAGGACCAACCGAGTCTGCTGAGGTTGCTAAGGCCATTGTTGTCGAGTGCATGTCAAAACCCTTCAATGGAAAGAACATTCTTAAAGTGGACCTTGTGGTGGATGCCAAGTGTGAACAAAACTGGTATTCTGCCAAATAGATCTTTAACAAAGAATTTCTGACTAAACTGACA

>cucumber_newGene_445 cucumber_newGene_445.1

CTTGGTGTAATTTTCTCATTTTCCTTCTCCCTCACGTTCTCTTCTTGTGTGGCTTTCTCCTTCTCAGCTCTTCTATTAGGGCAAAGTGCAGTTCGACTTCAACTCTTCTCCTTTCTCAGTTGTCGTCGCGTTTCCTTCACCATCGCCAACCGTTCAGGCCTCCATGGTGGTTAGTTCGTATGCGGCATGCAAGATTCTCCGTTGGAGATGCGTCGAGATTGGCGGCCTATTTTCCTCCCTTTGAGCCTCTCAAGTTCACCATTGCTCACTGTATCTTCGGCGCTAGCAACATCATCAAATTGCTTCTGTATGCTCAAATAATGTACTGAGTTTCTTACTTCTCAGTCTCCATCGAAGGTTCGTTAGCGAGCAGTAACTTAACATGATTCTTGGGGCGATTGGTGGCTACAAAAGAGTCGTGGTGGGTCATTACAATGGCTACAATACCAATATACCAATAGACAACCTGTTAGATTTGATATACACTATTAGGATGTTTTGTTTGTGGTCACGTTTTGTTTATATGAGGAAACTGTATTAATTTCATTTTGTGCATATGTTCATTTGTGTACCAAGTTTTAGTTTGCTGAATATTTATATGTTAGTTGTATAAAATAAGTTGGAAACTATTAAGTATATCTAGTTGTGA

>cucumber_newGene_447 cucumber_newGene_447.1

TGAAATTTGGATCAGTTTGGGCAATTTTCCGATATCCTCCTCTCTCACTCGCTCATCGAGCCGCTCCATTTTTCTTCTTTCTACTTTCTCCTCCAACGACGCAGCTACTACCCGCGTGTCCTGAAAGTTGCTGTGGCCACGTTCCCGATCACGAGCACGCCACTTGCTCCGACGAACGGCGCTCAACAATTCTCCCTCTCCGGCGACCTGCTTCAACAGCAGCTGCAGGCATTTTAATCTATAATGCCGCCGCAGCGGCGACATTTTGATTAAGATTACTCACAACCCTCTGGAAGGAATCCAGATCAAAATATCCACACCTCGTTAAGGATTGCAAGCTTCAAGAGGACTTGACAGATGAAATGGTTGGTTTAGCAAAGCAGTCGAAGAGAGCAGTCTGATAACGAGCCAATC

>cucumber_newGene_448 cucumber_newGene_448.1

CAGAGACAGAAAACCCAGTAAAATGTTCTTCTAGTTGAGGACATAAAAGTCTTTTAATTTTAGTATTTATCTAACATCAACTGTTTAAGGTTATTATATCATTGACAGTTTGAAGATTCTAAAATTTTCTGATATTTTGAATGACCTTTTTGGAGTGAGTGGGTGGAACTTTAGTGCCGAGTCTCTTGTATTCAAAATGAAGTGGAAAATCTGAAGAGCAGGGTTTTCGCCACTAGGCAACCAGGCAGGCACACTCATGACCGTTGAACAGCAAAATTAGGCATGAAATAGTGTTTGTGCGGTGACAGCAACCCAGGCTTTCCGCCCCCAATGTCATCATATGGCATGATTTTGAAAGCTACTTGGCTACGGCACTATCAGTCTACTTGGCCAAATGCTGCAAAAGGTTTGTGTTTCTGTTTCTGTTATCGGATAAAATTTAAGATCTCTTTTGATATTCTTTGTCTCGTTGGTCTTTTGATTTTAATTTATTTTAAATTTCATACCATAATTTAGGTGTTTCCTCTGTAAAAGAGCTGGTTTCTTTTAATCTAAATGACAAAAATAAAATGAATTTTATTTATTTATTTTTGTTTTCAAACAATCAGACCTTGATTTGGTATTTAAAAGCATGAGTTAAAGGTTTGTAATATATCAAGAAAAATTTAGGGATGGAAAATGGTGATTGTAGGCTTATTTTTTTACAACTAAAAGCAAAAATTTCAACTGCTTATAAGATGAGGCATTTGCTTTTAGTTTTTAATTTTTAAAATTTATGCTTGTTTTTTAAATTTCATACTTTTTTTTTTACATTCGTTTGTGAAATAGTTAAATTTTTAGTCTAATTTAGGACTTCGAAAGCTACTTTTTTTAAAAAAAAAAAATAAATTAAAATGTCTATTTAATTTTATTTAAGTTCCGCTTAGCTTTTTAAAACGTTGATAGAAAATGGGTTACAGAACTGGGAAACATGTGGTAGTAATGTTTAGCAGTTTAATCTTTAAAATTCGGAAGTAAAAGCCCAAACAGTTACTAAACAAGCAGCTTAAAAAATTGTGCTTGTTTTCACAAATTTTCGTGTTGTCATCTTTATTACAATTCTTAGCCAAATTCTAAAAATGACATTTATTTTAAGTGTTCACACATTGTCACATTGACATGACTTTAGTAGGAAGTAGATAACGAAAGAAACAAACTCAAGTTTTAGTTTAATTTCCAAAAACTAAAAAGAGGCCTTACAATTTCAGGAAAGGTTTGATTTTGAAGCAATCGTTGTGTTTTGGAAAATGGGAGGTTGTTTGAGTGGGGAGTTTTAATTTAAAATGTCAAAGTGTTATTTTGTGGTTGTGGTTTTTGTTTTTAAGTGTAGGGTTTGACTTTTGAATTTTAGGTTACATAATTTGAAATCCGTGAATCAAATAATGCCCGTCTTATATATAATTTACTAGTTGATTTGGTAAAGACTTCAATGCTACGTCCGGGTAAATCGAGGTCAGGATTTGGACCTATAACTAAATTCTAATAGTTGTTCAAATAAATAATATTCAAACTTAGACTTATTCTTCTAAGTTTGTTTTTTATTTCGTAACTTTAAATTTTAATTTAATTGGTCTCTAAACTTGTAGTATGCTTCCATAGTTCATCCACATTTAAGATGTGTTTAATACGTCTCTTTTTG

>cucumber_newGene_449 cucumber_newGene_449.2

GTGAAGCGTGGCGTTGGGTTTTGTTTTCGTTCAAATTTCAAATCTTCCACAAACTCTGTCCCCATCTTCTCTTATTTTTCTTCCTTTTTTTCTCTTTCCTCTAATCTTTTGAATTTTGATTTCGATTTCGATTTCGATTTCATCCATCCCCATCCCAACCCTAATTCCCAATTCTTCACATTCTGATATTCCCCATTTCTATTTTTCGATCCCTGCTTCTATGGCCGACTCCGACTCTTCCACTCCTTCCCCTGCTCCTCTATCAGCAAAAAAGGAGAATCTAACCCCCATTGGCTCCAAAATTACGGAATTGAACGAATCAAGATCAGAGCTGCTTAATAGAATTCAATCTCTTAAGCAGGATCTCCAGAATTGGAGGTCAAAGCTAGACACGCAAGTTCATACCTACCGCAGTGAACTCTCTGAACTCAAGAAATCACTTAATGTTGAAGTAGAGCACCTTCGCACGGAATTCCAAGAACTAAGGACTACGCTTCAGCAGCAACAAGAAGATGTGACTACTAGCTTGAGGAACTTGGGTCTTCAGGATGTTTCTGTGGACAATAATGAGACACAACATCAACATCCAGTAGGAAGCCCCGACAAGGAAGATGCAGACGAGGCTAAGAAGGATAACTTAACAGAGGAGAAAGGTAATGAAGCTGAGAATTAAGTGGCAGTTTTAGCATGGTACGTTTCTAAGCTTTTCATTGCATGGTAAGAGAATCTTAGCAGTTCTCTCTAGGCGATGGATTTCCACATTTTTCTATTTTGCATCATGCTCTATTTAAATTTCAATCTTTGTAACGATAGGGTTCTTTCAATATTTCCCATTGAGGTGTTCTATGATATCTCCACCCTTTAGAGCTGATCTATCTCTTTGGCTATGTCTCTTTCTCTAAATAACTATGGAGTTTATGTTGGACAAACTTTCAGTACTTCATAGCATTGGTTCATTGAAGAACGACGTGTTTCTTGGTTCTATATTTCTCTCAGTTATAAAGAATTGTGGGGCAGAGTAGTTATTCTTCTTGCAAAGGCTTGGTTTTGGTAACTATTTGGTTCGTGATTTTGATTTTTTG

>cucumber_newGene_450 cucumber_newGene_450.4

TTCAATTCTCGCGCGAAGTATCCATCTCCAATTTGACCGTCTTCCGGCCCCGTCCCCATCCGCCCCTATTTGTGACCATCTGTTCACTTCGGCGTGGCCGTCTATTTGCTTCGACGTGCCGATCTGTTCACTCCATCGCTGCCATTAACGAACAACGAAACCCCCGCAATCCCTGCGCAATCTCCAAACCTTTTCAATTCGTTCCCGTCTTCTTCGGCGCTGCCATTCGTGCCCCTCTTCTTCGTCGCTGCCATTCGTGCCCCTCGTCACCTCCTCTGCTGGATTCCTTTTCATAGGTGAAGTTCGAAATTGCTGCTTATGAGAAGTTAGTATTTGGAAAAATTAGAGATGGTATTCTTGTTGTTGCATCACATCTACTAATCAGTCCTATTCAGTTCAATCCAGTCGTGGCCGTAATCTCCAATCTGTCGCATTCTCTTGAATATTCAATCTGCATCTGTGACCTCCTCTATCGGTTTGCAGTAATGGTTAATTCAGATGATGATCAAGTTGCTACACCCCTGAAAGAGGAGAAACTAGTAGCCAAAAAAAAGAAATGTGGCCCTACGAGAATGAAAGATATAATACGTTTTAGTAGTGAGGGGCGAAGAAATGTTATTCAGTACAACGAACTAGGTCAACCTATTGAACCGAATGCAACAAAGTTGAGAGTTTTACTGGAACGACTATAAGGTTTCACGTTCCTATCACGTACTCTACTTGGCATGTCATTCCAAAAGAGATGAAAGAGAAAATCTATAAACTAATTGAGGCCAGCTTCATTCTTGACCCTAAGTCGAAGAAAAGCATACTTCAAAATACGGGAGTATGCTTTTGCAGATTCAAGTCGAGATTAACAACTACATACGTACTACTTACAAAGGGTGCTGCATTTTGGATTGACCCGTTGAAGAATCGGATTGACGGAGACATGACTGAAGTGCTTCAAATGCCATTCAATATATCAAAGAAGAAAAAACAAAATTGGAAGGTTGTGAAGGTATTTGACTTTGACCAATAGGATAGTCGTAAATAATTTATTCATTTAAATGTTTCACACGTATGTTTTTTATTGTGCATCCACAATGTCCCAAACAAAATGGAATGGTAGAATGCAGGTACTATGTTATGCGATTCATGTGTGACATAATTTCTGCGAGAAGTACTTTGATTGTAGATGTCGTAAGCATTAGAATTTTAAAACATATGTTATGTCATTGTTTGAAATGATAATAATTCTTAACCACTTTATTGTCTACGTATGAAAACTTTACCTTCTACATACTCGCAAGATGAAATCGATGAAGTTAGATCGGAATTGGCGGAGTTCCTTTCTAAGCACGTACATCGTGCTTAGCATCAGATTAGGAAAGAACTTTTTTGAAGTATTTTGTGGGGATTTTTTGTTCATATAATTTTGTTCAACCCAAATGACACATAAATAAATGTTTAAGCACATTGTTTTTAACAAACTGAAGCATGAAAGGAGTAAGTTAATAGTTATTGTATCGTATCTATATATATGTGACTTGCTTATATGTAAGTGGATATGAATGTGTGTGTGTTTATTTGTTTCTTAGGAAGAGTCCAATATATATGTGACTGGCTAATGAATTTTTTTTGTTTGGGGTCCTACTGCATTGGGATATGGATCTTCTTACAATCAAATAACTGTCGAAGGTAATTCTTTGCTTGCGTCCTTGAGGTTTTATTTTGTCAATATTGTGTGATTTGTTCATGTTCGTATACATGACAAATTGTCAATTGCTAATTCTACTTGGTTCACTAGCTTTGACTGTTTAATATATGGATTGGAGATTGGAGATGTAGGTTTTAGCATTGTGCTTATCTGTGTAATTGGTGTTTTTATTATAATTGAGATAGGGTTGGTGTTGTGTATGAAAAAGAACTGGAAATATGAGTTAGCTAGGCTTGTGAAGGTTCACACTTTTTAACATTCTGTTTGGTTTATACTTACTTTATTAGTGTCTTAGAAGTTGGTATCTTTAATTTTTGTTCTGGCATGGATGGGCCCTAGTTTGAATCTTAGTTTTAATTAGCTCACAAAATAGTGTCATGTATTGAGTAGTGCTAGTTAGGCTTAGACAAAAAAAAAGGTATAAAGCACTGTTTCATCCTTCTTCACTCCCTGTAGCAATGGTTATTTTGCATGTTTGGTTTTTTCAGTACTGCGGTCTTCCAAAATATGGATAGCTTGGTCATGGAACAGATAGTGATGTATTATACAACTTTTTCTTCTATTCCTTTTAATTTGTATTGAACAAATAGAGAATCTTACAATGGTATACTTTGTCATGTGACACATGGGATAATGCTGGACACTTCATTCCAGTAACAAGCTCTAGGCCTAAATTGCCGCAAGCCAGCAATAATGGTATCCAGTCGTCGTCGAATCCACCACCGATGTTGTCTTCGACTATCGTTATGCTTGCCACCACCGTCAAGTAAGTTATTTCTTCAACCATTGCATATGTGCCCAACAATCGCCACCACCGTGTGCCCAACCGCGTCTATCTCATAAGCTCAAAACTGGTCTGCTCGTGGTTAGATCTGCATTCAAAGTGTATTTATCTATATATATTTTTATGTGTGTTTCTAATTTGTATAACACTTAAAGTTTGTTCTTAATTAAATTGTATTATGGATGTGTTGAACTCAATATTTAATTTAATAGAATTCATCAAGTAAGGAAAGAGAGAT

>cucumber_newGene_452 cucumber_newGene_452.2

CGTCCCTCCAAACTTCCAATTGGTTTCTTTCTTTTTTTTTCTCTCTTCCTTTTCTTTCTTTTAGAAATTTGATCTTCTTCTTTCTCAGTTCTTTACTTCTTTCCTTCTACTTCTTTTCTCTATTCTTCCTCTCTTTCTTAACGTGATTTTTTAAGTTTAAAAATATTTTGCAACTTCCATAGTCGAACAAGAAGGAGAAAACCCATAATCGTTGAATACATCCTCTAGTTCAATTTTGGAGGTTTATAGTTCAAGATCAAGTATAATAGAAGAACCTCAACGTTCTGCATCTACATCTTATTCGACTTCAAAGCGGGATGGAAGTAGTAGGAAAACTTCTCTTGCTAATAGGAAAGATCGCCACCTTAAGTTTAAAGTGTTTGTTAGTAATATGGAGAATGATGTTGACAATGTCAAATCAGAGCTTGATCGTTATTTGGATGAATCTCTTTTACCACGAACATAAGACTTTAACATTTTGCAATGGTGGAAATTGAATGGAGTCAAGTATAATGTTTTGTATGAAATTGCAAAGGATGTTCTAGCTGTTCCCATCACTACTGTTGCTTCTGAATCTTAGTATTGGTGGTAGACATATAGGTCCACGTCATTCTCAAATTCATGTAAACTTATTGGAAGCTTTGATGTGTACTCAAAATTGGTT

>cucumber_newGene_454 cucumber_newGene_454.1

CCCCATTCCGATAGAAATTTCTCTTTTGCCAACCATATCTTTCTCTCTCTCTCTTGCACGCTGGTAATGACCTTTCTCCAATGAAAGACGACGATTCCATGACGATGAATCCCAATCGACAATCTTGCGTCTCTCTCTATCTTGCGTAAGCCACGACGTGTCTAGCTAACGCCACACCCAGCCACTGGGCACCGCTCGCCTCGCCAGACGACCACCACCACATGCTGAATCCTTCGAAGGTCACACAGATCTAAAGTTGTTAGACTCGATCTGTATCGTCTTGCTGAGTTCCTCAAGGATTTGATCGTCAATTGAGTTACCCCAACACCTAGTCACCATTCGTCACTATTTTAGAGTCGCATCGTCCTTAGCCTTGCTCTGTTCTAACCATTATACCTTGAATATGAAATTAGTCGATAAGTGGACAAGAAAGAAGCAAATCGCAAAGAACACCTCACGCATAGGTCAAGCGATGGGAACTCACCGGACAACTAACACTTCCAGACAAGGAACCATGTCATCATACGAGAAAAGCTCACTACAAAACAAGAATGGTAGTTAGAGTGATGTGACTTCCTAAGACTTAAAACGACGCTGACAAGAACAGAGGAATAAAAGTTTCCTGCCTAAAGTTACCTATAAAAGCACTTCATCTTCAGTAAGACAACGGAGCGACGTCTGATTGGACTGCCACACACAAAAGACACATGAATGGGCTAGAGACCTGCATGGACCAAAAATATGCTCTATATGGGCAAAAACCCGAAGAGTCGATGGCAAGGATTAGCTTAGCCACCAGATGCTCAACATTTCATCTTTCCTGATTAGAGTGTAACGTGAAAGCTTAGATGAGAGGAAAAGAGTCATCCCCCGTTTCCCCTAAGTGGTGTTTCATTCCACTCTTCTTTCCTTCCATTTTTGCATTTCTTTGTAATATATGTTGTTCATATTGTACAGTGGTCAAATGCATACATTCTTTATACATATATTACTTGTTTATGCCTTTTCAAT

>cucumber_newGene_457 cucumber_newGene_457.2

CCGATTTCTTCTTCCTCTCCGGCGAACAAGACCCGAGAATTGAGATCTTAACAATGGCGTCACAAGGCTCTGTGATTTCTGTATTCTCTATTTCCTTGCCATCACTGAACGGTTCATCGGCCTCGGTGTCTCCGACATCGCGGTCGTCACTGTTTTCCGGCTTTTGTTTCCGCAAACACCAATTACCGCTCCAATTCACTGCTTAGAGAATTTAATTGCACATATTGCTCCTTGAAGTGGAAAAAACGATTCCCCTTTCTCCTCTTGATTTTCTAATTAGCAAGGTTGGGTATTGTGTGGAGCAGCAGTCTTTAAGATTTTTGCAGCAGCAAAAGAAAAATGACTACGGAAAAGAATGAAGGAAGTTTTATACAACAAGCCATTCCACGCTTTGATGGTCACTATGATCACTGGAGCATTGGGAGTTGGTAGAACCTGGATATGTTGAACCAGCTAGCGAGTCATTGCAGACAAATGCTCAACAGAAGAAAAATGATGAAATGAAGCTGAAGGACTTGAAAGTGAAAATTTATCTTTTCCAAGCCATTGATCGCACAATTCTGAAGACCATTCTCAAGAAAAATACTGCAAAAGAAATATGGGATGCTATGAAGAAAAAGTATGAAGGAAATGCAAGAGTCAGGCGGTCTTATCTTCAAGCTCTTTGTAGAGAATTTGAAATTCTTGAGATGAAGTCTGGTGAAGGGGTGACAGAGTACTTCTCCAGAGTCATGATTGTGGCAAATAAGATGCGAACTTATGGTGAAGATATGCAAGATGTAAAAGTAGTTGAAAAAATCCTACGCTCCTTGACTGACAACTTTAATTATATTGTTTCTTCTATTGAAGAGTCAAAAGATCCCAACACTCTCACTATTGATGAATTACAAAGTTCTTTAATAGTACATGAACAAAAGTTTCAACGACGAGGTGGGGAGGAGCAAGCCTTGAAAGTGACAAATGATGAAGGAAGAGGTCGTGGTAGTGGCAGCTATAGAGGAAGAGGTCGGGGAACTTTCAACAAAGCCAATGTGCAGTGTTTTCGATGCCAAAAATTTGGATATTTTCAATATGAATGTTCTGAAAACAAAGAAGCAAACTATGCTGAATTTGATGAGGAAGAAGAAATGTTTTTGATGTCTTATGAGGAAAAACATGGAGTTCAAAGAGAAGATACATGGATTCTTGATTTTGGGTGTTCAAATCATATGTGTGGTGATCGATCAATGTTTAGTGATCTCAATGAAGATTTTCGACATTCAGTGAAATTGGGAAACAACACTAGAATGAATGTCATGGGCAAAGGAAATGTAAAGTTGCTCATAAATGGAGTTAATCACGTTGTTGCTGAGGTATATTACATTCCAGATTTAAGTAGCAACCTATTGAGCATAGGACAATTGCAAGAAAAAGGAATGTCGATTTTGATCAAGCGAGGAGAGTGCAAAATATTTCATCCAAAGATGGATTTGATTATTCAGATCAAGATGAGCAACAGTAGGATGTTTACTTTGCAAGCTCAAACTCAAATATCTTGGGGAATTGACAAAAATAGGCCAAAAATGGGGCAGATAAAGACTTTTAGGATTGATTGTAAAAAGGTTGACATTTAGGACAAATTGGAGGTGAAATGACGAAAATACCCTCATTTAATTTCAATTCACATTTAATTTCCTCCTTCCTCTTTTCTTCTTTTTACTTTTCTTCAACCCAAAAACAGAGAAGGCAGTTCAAAAAAAAAAAAAATGTAAACCTCCCGCATTTGAACTCTCCCGCCTCTGCAGTTACCACCTTCAACTTTGTGCCTTCGGTGAACGTTTTTCTCTTCCGTCCATTTCTTCTCCACTTCCATCCATTTCTTCTCCACGACTCTCCACTATATTTTCCGATTTCAACTTTTTCACCGAACGCCTCCATTTCTTCTGCACGAGTCTCTGTTTTAGTTTCAACTTCAACTTCGGTGAACGCTTCTCCACTTCCATCCATTTC

>cucumber_newGene_458 cucumber_newGene_458.2

TATTGAAATGTTGCATAATTCCGCTAATTCATTAATGGGCTAAGTGAAAGCTCATGGTGATGACACCAAGGCTACTAACTCAGTAAAGGGCCGTTTGGAGGAGGGAAAGGAAAGGAAAGGAGTCCTTTCCTTGTTTGGCCCACATATTGAGGGGATGAGGGGGGAGTAATACAAATAATCCCCATTTCCCCCTCATTTATTCCCCCTCTTGCCCCACTTAATCCCTTCAATTTCTGAACATAATTCTTTTTTTTATTCTTCTCTTTTCTCATTTATTTCTTCTCTTTCTTCTTATCACAACCAAACGAATTACCACAATCATTCTCTCTTCTTCTTCATTCTCTGTGCATCTGCTTCGTCTTCTTCATTCGACCAACCGCTAGTTATCTCTCCTTCTTTACACTTTTCCTCTCACGCATTTGCCCAAATCTATTTCCCCAAAATTGATTTCTTTCTCAACCCATTTCCCTAGATCTTACGGTTTTTTTACCATCTTCGACGCTCACCTAGCTTTTCTCTACCGTTTAACATAATCTTTTTCACGGTACTTTCATCTACACCGATGTCTTAAACCTTACATTTTTTTCTCTGTTGTCTGTCAGTCTCCGTTGTCCCCGTGGGTGTCGGTTTCATTGACGAGCCACGTTTAGAAATAAACGATGGCCTCTGATACCTTATCTCCGGTCGGGCGTTTATTGACTTTGTGGTTGCTTCCTTGCATGGCCTGTCCTAGGGACTTGCCGCTCGAAGGTTATTCATTGTATTTTCCTTGCCACTCGATTAACCCTCTTGTCATGACGACCTTCCCTCCACTAACAAGTGACAGATACTAATTGAGGGTTGTGGCTTCGGTGGTTCCAAGCTAGGTAAGGACTAAGTGTGCTCCAGTACGTAGATTACTATTTCGAGGTACTAGAAAATCTACATATTCAACAAACGTAGGTCATTCTCTATTGCATGTATATGTATTATGTTTAATACGTACCATGTAAGAAAACTTCATATAACTCTTTTTATAATGGGACATTCGTTTGACATGGCATAAACCGAAGTTTTGTGGAAAGTAATTATTTTTCTTTGT

>cucumber_newGene_459 cucumber_newGene_459.1

ACAAAAGCAAATTTTTGGAGTCCATCTAACAAAAGTATTTACCAGGGTGGCCTTTAGCATACCTTGCAATGGATCCAAACAAACTGGCCAATGTTTTGACTACCTTCACAATGGTGCAACGTCAAATTATATACTGGCGTTTGAGGCATTTTTCAATGACCACAAGAGGATTCCCCAAGTTCCATGTCACACTAAGCATAGGATTAGGCAGATTGCGTATTTTCACATGATACACATGTCTGACCTTGTATGTCAACAAAGTACACGCAAAGATTGACATTATTTCTCAATTTTGTGTCATCTGTTGCAGACAACGTCGACGAAAGTCATGGACGTTGAGGAGACAGTTTCGTAACATTTCAACCTAGTCCTTATGGTCATGCTTACTTTATACGATGAGTTGTTAGCGAAACCACATCCTGTCACAAGTTCCTGCACATATTTTAGGTGGCGGTGTTTCGAGGTTAGGTGTTTGGAATTTGGTTCAGTTATATATATCGACTTTATCCCATTTCCTATTGGCCATTAATTATGCAATGCAGAATTGCCTTGGTGCCCTTGATGGCACATACATCAAAGTGAACGTGTCAGCAAGTGATCAGCCTATATATCGAACACAGAAGAATGAAGTGATGATGAACATCCTCGGTGTATGCAACACAAAAGGGGGCTTCATTTTCGTCTCATCAGGCTAGAAAGGATCCGTAGCGGACACCCATATTCTGCACGATGCAATTTCACAACCCAATGGACTAAGGGTTCCCAAGGGGTACTATTACCTGTGCGACGCGAGCTACCCCAAATGCGAAGAGTTTTCTTGCACCGTATAGAGGCCAACGATACCAACTGCAAGAGTGGCGTGACGCCGAAAATGCTTTAACTACAGCAAAAGAGTTTTTCAACATGAAACATTTATCTGCACGTAATGTTATTGAACGAGTGTTTAGTCTTTTGAAGATTCAGGGCAATACTTCGTGGAAAGTCGTACTACCTTGTTTAGGTTTAGTGTCGCAATATTATAGTCTGTTGTTTACTCCACAATTTAATAAATCAAGAGATAACGAACGTCGACATGCTAGATGACAACGACTAGGGTGACTCAAACTACGCAACGCCTAGAGGTGACGACATCAACTACATTGAGTCCTTAAATGAATGGACTCAATGGAGGGATGGCCTTGTTGAGTCGGTGTTCAATGAATGGAACGACTAGTACGTCACAGTTAGTTGTAGGACAAGCACGTGATAAAAGATTGTGTAAAAATTGTATTTTTGCTATAGTCTGATTTTTTTATTTCGTTTATGTTATATGTACTCTTATAATACAAATGATAAGATTAATATTTGGTGTGTTCATGTTTTCGTGTATGCACGTGTTTTTTCATTTTTGTTTGTCAGCATGGCCAGTTTGTCGAGAAATCCAAAGCACCTATGGACGAGGGTGAAGGAGTCCTGCCTCGTTGGATGTCTTGTGGACCTGGTAAATGAAGGAGGGTGGAGGTCGGACAACGGGACCTTTCGATCCGATTACCTAACACAGTTGGTAAGAATGCTATCTGAGAGAATGTCTGGGTGCAGATTGATGTCCACGACTATTATCGAAAGCATAATAAAGTTACTTAGAAGAACATTCCAGTCATTCACTGAAATGTGTGGTCCCGCGTACAATGAATTCGACTAAAATGACAAGTTGAAGTGCATCGTTGCCGAGAAGGACGTCTTCGATACTTGGGTCCAGAGACACATCCTACAGTGAAAGGCCTACTGAACAAACCTTTTTCGCAAAACGACGAATTGTCGTACGTATTCAAGAAATATTGCGCTACAGGGGCACGTGTAGAGACCTTCATAGATATCGAACCTAATGAGCCAACCGACAGCAATGGAGTATGATCGAACAACGACCTTGATATGGAGTTCCTAAATATGTGCAGCCCGAGAATGAACATGTCCCAAGAAGACATGTTGGGCAGACGACCTTCTAGGTGAAGCAATGGTAGGCTTGGTTCGAATGGAACGATGAAGAAGTAGACCATGCAGTAGATTGACAATTTGGAACTCATTCATAATGTCATGGACTATACGAATGAACAGCTGAGAGCTATCGCAGAATGATCAGATAAGGCGCGACAAAGTGAAGCTGTTGTGCGGTGGGAGGTACTAGCACACTTGGAGTCCATTTTGGACCTTAGTGAAGAGGACCTGACGAAATGTATAATGGCCATGATAGAAAAAACATCGATAATGCATACATTTTTTGACATGTCCGATCACCTGAAGACCGCCTAATGCAGAATCATTCTCTTGGATAACCCCTTATTGTTGGTCCCTTTCCA

>cucumber_newGene_465 cucumber_newGene_465.1

CAAAAACCCTCACTCATGCGAAGAAGCCATCTCCAAATGCAAAGAAGGCCATATTCGGACGATGTCCGATCCCATCACCGCCACTGCCCCTCGTGTCCAAATCTCCATGATTGCATCTCCAACTTCCGCTCCGATGATACAATGGACTCCGAATTGTCTCGCCATCACTCTGCTTGCAAAAATTGAGGATTCTTTCAGTTGGTGAAGCACGGAGTGAGCGATTCGTTGATGGAGAGAATGAAGATGGAAACGCAGAAATTGTTCCAACTTCTGATTGAAGAGAAGAAAAAGCTACGGCAGAGAGAAGGTGATGTGGAGGGGTTTGGACACGCTTTCATTACAAGTGAAGAACATAAATTGGATTGGTGTGATATTTTCTTCATTGCTACATCGCCTTTACATTTCAGGAATCCCCGATTGTTTCAAAATCTCCCTCTCTCTTTGAGGTTCGATCATTCCTTACATTACAAAACTTTTTAAATGATTCTCAAACTCATAAAATCCTAGAAATTAAACTCTGAACTCATAAATATAAAACGATTCTAATTTCTTATTCATGGTACTTCACCGTCTAGGAATGGAATAAGAAAAAGAGAATATGATTTTCTATTTCAAGAATTTGATATAGTTCTGTCATACTAGCGTTTTTGGCTTGGGGGTATACTTTCAACTGTTACCCACATTCCCATTGGTCTTAAATCAGTCTACCCACAATGTCTAAGGATCAGATATGCAATTTTGAATTTTCTTAAATGTCGTTCAACAAGGTTTGGAGTCCTTTTGGGTAAGAGTCGGTAAGTATATTTGTTTTATTTTCCATTTAGAATTGGAGTTAAA

>cucumber_newGene_466 cucumber_newGene_466.1

GTTTCAAATCCCTAGACCTAGAAGGTCCCTTTGGACTATTTCCCCCATAATTAGAGTGTTCTAAAATTAAACACAAAATAAAGGTGTGTCACGTTTCCCGAAGTTTCTAGAGTCCCCACCGAAGTTCTCCAAATCGTGGCTTGCAGACCAAGTACTGCCATTCAAATTCGTTTCTTCCTCTAAACCTCCTCCCTTAATTTTGGCATTTTGGCTCCTATAAATTCACCCCCTCCTTATCCCTAATCCTTTGTCTTCCAGATTTTCCTTTAAAGCTCGTTTTTCCCATTTCGTCGCCTTCTCTCTTCATCTCAAGATGCAAATATTCGTAAAAACTCTCACTGGCAAAACAATCACCCTCGAGGTGGAAAGCTCTGATACAATTGACAATGTAAAGGCAAAAATTCAAGATAAAGAGGGCATTCCACCCGACCAACAGAGGCTTATCTTTGCTGGTAAGCAACTTGAGGACGGCCGAACGCTTGCCGATTACAATATCCAGAAGGAATCTACACTCCATTTGGTGCTTAGACTTCGCGGTGGGATGCAAATATTTGTGAAGACCCTTACTGGCAAAACCATTACCCTTGAGGTGGAAAGTTCTGATACCATTGACAATGTCAAGGCTAAAATTCAAGACAAGGAAGGTATACCACCTGACCAACAGAGACTTATTTTTGCTGGTAAGCAGCTCGAGGATGGTCGAACTCTCGCTGACTACAATATTCAGAAGGAATCTACTCTGCATTTGGTTCTTCGTCTTCGTGGGGGTATGCAAATTTTTGTGAAAACCTTAACCGGGAAAACAATTACCTTGGAAGTGGAGAGCTCTGATACAATTGATAATGTGAAAGCTAAGATCCAAGATAAGGAGGGTATTCCACCGGACCAACAGAGGTTGATTTTTGCTGGTAAGCAGCTTGAAGATGGCAGGACTCTGGCAGATTATAACATTCAAAAGGAATCAACGTTGCATTTAGTGCTCCGATTGCGCGGTGGAATGCAGATTTTCGTGAAGACATTAACAGGGAAGACAATCACATTGGAAGTCGAAAGTTCGGACACCATTGACAATGTGAAGGCGAAGATTCAAGACAAAGAGGGGATTCCTCCAGATCAACAGAGACTGATCTTTGCTGGAAAACAATTGGAGGATGGTCGGACATTGGCTGATTACAATATCCAGAAAGAGTCCACACTCCACCTTGTCCTCCGCCTGCGCGGTGGCTTTTAGGCGGCTCCGATGCCCTCTGACCCTCCTGGGATGGTTTAGTAGTAATAAAACTGGACCCAAAACTACTTTTATTTTAGTATGGTATAAAATATAATATGTTGTAATATGGCCTAA

>cucumber_newGene_467 cucumber_newGene_467.1

GATTCATACATTGATTTTAGGATATTTTTTAACTTGAGGGTAATATAGACTTTTTCCAACTAATTTGTACATCTTTTTTAAAATTCATCTCAAAACTCCTTACATCCTTCCAAATTATCCTATTTCTCATAATCCCCCATCTTCTTCCTCTTTCTTTTTCGTTCAGATCCACGGTCATCGCCCACCCTTCAACCTCACGCTGACTTCCTCTCCCCCTCCATCTTCTTTCTCCTTCTTCTTCTTCCCGTAACCTACCTCCAATCGACCACCAAATTTCTTTTCTTTTCTGTTCTCGTTTGTCTTCAACAGTCGTGCCTCAAGTCGTTGCTGTCACCGTTTGTCTCTCCCTCGTCCAGTCAAAACCGAATCGAACTCGCCATCCGTTCATCCTCTTCGGCGTTCGGTCTTCAATCGTTAAGTGCAACCAGTGATTCGTCCTGTTCGCGCCCCTCTTTCCGTCGGTAAAGCCGTTACCGTGCACATCTGCCACAGACGAGCCACTCGCCGTTCGTGAAGCCCCAACTGTCGTGAATGCTACGTCCGCGCCGGTCTTCACCGTGGAACCAGCCGTCGTCTGCTCTGTTTCCATTGGAACCGAAACGTCTCCCTCAAATCTGTTTTTCTTCCAGTCGTCCGTTCCATTTCGCGCAACCTCCGCGTGACTCCTCGACGCATAACCCCTTCGATCGAGCCGTCCACCTTTCTTTACGCCGATCTGTCCCCGTTCTGCAAAGCGCCGCCGCGTCCGTTCATCTGCCGATCGTGAGACAGTGTGCGTTTCTCCTCTCAACCGCGCGGGTTCTCTGCGTATCGTGCGTGACTTTTCTTCGATCATCGCTCCTACCTTGCTGTGAATCGTTGGGTGGAAGTTTTGGGTAAAATTGAGGTGGCAAAGGTAACAAGAGTGGCAAACCGGCGAGGGGCAGGAAGGAAGTGTGATTGCCATAGGGAACATCTTTATTTTGATTCCGCTTTATGTTTTTTATGTTTAAAACATTATGTTGGAACTTAGTTTACAAACTATACTTTTAAAGTTTTAGTTGTTTGAAATCGGGCCTGTTTAAAGTTGTTTTTCCTCGATTGTATGTTTTTC

>cucumber_newGene_468 cucumber_newGene_468.1

CGCCGCTGCCAGTCCAGATCTGCAGCGCCGTTGTTCTTCCGCCGCTGCCCAGCCGTCTCAGCCGGTTCTGTCTCTGTCGAAGCCACGGTTCTGCAACCGTCTCCGCCGTCTCTATCTCCGTCCGCGAGGCAAGTCGCACGCCTCAGTCACGAACAGCCTCGCCGACGCAGGTCACGCCGCATCGCACGAATTCGGCTACGTTGCCGGGCTACTCCCGTCCGCGCGTAATTGCTGTTGAACGTCCGAACCGTTCTGCCTTCGTCGATACCCACCGTCGCGGACCAGTTCGCGTGCAAAAGTGTTTTGCACGATGTCTTTTGTGTAGATCCTGCGGCAACTTCTATTTAACGTCGATTGTCAAAGTCTTGTTACAAGATATTGGCGACATCGGTGATTTGGAGTTAGTGTCCTTGAGTTACGCCTTAAGCTTTGTTGGTGTTCGATTCTTTACTCTTTGGGGTTTGTTTGTTGAAGTTAGTTCGACTAAGGAATTTTACATAAAGATCCAGGTAAGGGATTTTCTACTGGACTCACTTATGATTGTGAATTGTATGTCTGAATGATTATCTTTGACTGAGTAAAGATGTTGAGATGATACTTATGTTTATACACGTATGGGTGTAGATTCGGTGCTGAATGTACACTGTGGATTGACTGAAAATATATATATACATATGGATTGAAGTAGACTTAGTGTTGAATGCATATTGTGATTGTCATGGAATATATATATAAGTGTGTGTATGTGGACAGGACGAAAATTGTAGATTATGCTTGTATGAGATGCAGATGCGATCTTGTGATTGTCGTTTAGATTGGGTGTATGTTACGCTAAAGTATGACTGTGTGCTGGTGAACTGAGGGGTGTATTGACTGTATAGTTTGATTGACTGACGTATTAGTATATTATTGACAGACATATGACTAAAGTGAGATAAGTTGACTGTTAGGATGTTGAAAGAAAGGACTTTGTGATTTGAAGTAACTGAAAGATGGGTTGAATGGAATGAGGGGAAAATTGTTAGCTTTTTATTGGGATGTGTGCCTTGTAGGTGTCCCACGGGATCACCAATTTATTTTGCACCTTCGGGAGCATTTGACTGATATGTGTTCTGCAGAACACTTGACTGATATGTACGTCCCTCGGGGCGTTAGACTGAGATGTGTATCCTACGGGATCACAAGACTGCGACTGTACAGGGTGTCCCAATAGAACTTTAACAATTTATTTTCCCCTGACGAGACCAGTAGAGGGTCTCTTACTGAGTATTGTTATACTCACCCTTCTTATGTTTAATTTTCAGGCAAAGGAAATAAAGGTGGCAAATCGGCGAGGGGCAGGAAGGAAGCGT

>cucumber_newGene_470 cucumber_newGene_470.3

TGAAAGAGGCACTATCAGCATTGACATGTACATGTGATTTTAGGCATGAACCAGGAGAGTGAGAGTTTTAGTGTGTCGTGTTTCGAATTTGTCGGTATCTTTTTCCCGTTCCAGCAAGTATTCCTGATTGTGGGGATTCTTCCAGTAATGCTAGATATTGGTACTAACAACGAGAAGTTACTTAATGATCCTCTTTGTAAGTGAAAGGAAAACCTT

>cucumber_newGene_473 cucumber_newGene_473.1

GGAATATACATTTTTGAATTTTCATTGAAAGAAAAACCAAATTCAAATGCAATAATGAAGCAAACCCTAATTCATGTTTGGTCCAAAAACTGAAACAAAGGATGTTAGCTCCTCTCTGCCCACTTGGTGGGTTATGTTCATAAAGAGAGAGAAAATGGGTTGTATCTTATTCTGAGAGAAAGAGATAGAGAGAGAGATGGGGAGGGTTGCCTGGTTGGCTTCCATTTTCAGAAACCATTATGAATGTTGAAGGATGTGCGTAGAATTTGGATTGCCAAATGGGGTTTCCTCTCAGAATCATATCGTTTTTTAGTGACCCTTATTGTCTGCTTGCTGCTTACTCCAATGGAAGATGGACTTTTTACCAGTTAAATTCTTGCTTGGAACTCCGAGGAACTTTTGATGAAATGAATGGTGAAACCCCCAGTGACCTCTGAGTAAAATTCTTGGAATGATGGAGTTTGATTGTTGGAAATTTGAGGATTTATTTTGCTCATATAATGAATGATGAAATGCCCAAGTGAATTCTAGTGTTGTACGTGCTCAAAATTGCATCAGTAATCAAATTTCTTATTTGTAATGATGAACTCTTTTGGTAAATTTATGCTTGGAATGTGAAGAATTTTTACTTATATATTGAATAAGACCCAAATGAATTTGAGTATCTTCA

>cucumber_newGene_474 cucumber_newGene_474.1

CCATAATCTATTTGTTATGTGTTCTATGAACACAGAATACTCAGCCAAAGGAAAATAAAAAGAGGGTCGAGAGAGAGGGAGAGAGACTATGGTAGTCTACAGTCGTCGTCCGTCGTCGTTACTGTCATTATTTTCGCCGCCCACCGTCCTCCATTGTCGTCCACCGCCACTGCTCTCAATCAAGGTGATGTTGCTGCATTATTGGTGGCATACGATTTTACATGTCATTTCGCGGACAACTTCATAGACGACGATGATGACAAACAATCATCATCACATCAAAAAATAATATCTTCTTCATGTATATATGTTAGTCATTTTTCTATACATTTAGTTTTCGATACTCACATTTGTTTAACGTTTGTTGGTTTTTATGTCTGCAAGAATTATGACTTCATTTCTTAGTGATATTAATGAGGCAAACATTTACCTAAACTTGGATGCGTTAGATAACGCGGGAGGATCGTCTTCGATGGGCAACAATCCAGTAGATCCCTCTCAACCCTCTCCCACTCCCACTATGAGAAGTCAACAGCACTCTCGAAACATTGAGTTGGAGAGGTATGTCCAACAGAATGGAAGGATCCCCATTTCAATCCCCCAATGAGGGGACAAGCTTGTCTCACCATACGCCCCTCGTTTCAGCTGCACGATCGGTGTGTTAACGCATGACACGTTTCTGATACAAGTTCTAAAGTAGGTTGATGTACCTCGAGAGTACATCAAGGTTGTCAAGAGAGGCTTAGCGATAACAATTTTTCATGCTCAATCTCGAAGTTTCAACACTTAATCGGTTTGTTGAGCATCAAATGCTCAGCGTGTGGAAGAAATTCAGGGGAGATAACCACCGATATTTTAAGAAGTACAACAACCATAAAGAAGTATGTGCAAATCCGCAGCCAAGATTCGCCAATCGCGTGCAAAATTGGCACTTTCTTTGTGACTATTACATAACTCGACAGTTTCAGAAGCTGCAGAAAGTCGTTCATTCAAAGATAGATCGAGCTTATTGAAGAACGCAGTCATCCAGTTAATCGTGTCAAGTTGTTTCGAGAAACGCATGCCAGTAGGACTGATGAGTTCATTTCACAGTAGAAGATGCTCTTTTCCCAGCCCGTTCTAGAGGGTTCTCAACCACTCTCTGAGGACGAGATATGTAGACCCTTTTAGGTAGACAACCAGGCTACTCAAAAGGTCTTGGTGGGACTCCAACCCCAAGTCCAAAAGAGTTATGCTTTCACTTCTTCCTTCAATAATTATGACCACCAGGCACACATGATAGAGGTTAGCGAACTAAAGACCAGCCATGAAAATGTTAACCGAATATTGGAGCACAACGACTAAGGAAGGAGGAGCGAGACCGACAAATAGCAGACCATGCACGACAAAAGGAGGAGATGAAGAAGATGATCAAGGAGATGAGTTGAGCACAGAGAAAGTTGTGAACACTTGGGTGCATACTTTTCAATATTTAATTTTGTTTAATTTTGCATCTGTACACATTCTAACTGTACTTTATTTCACATTCTTTATTTGGTGTATGGGACTAGTATTATGAGATAGGTGTAGGTTTTTTTAATTTTAATGTACTTGTATTAAACATATTATTTGTAGTTTTT

>cucumber_newGene_476 cucumber_newGene_476.1

TTAGTCGTCGCTCTTCACCCTCCTTCTGCCGTCGACGACCACCGCTGCTCAGACGAAGTCATTCTCCTTCGTTGCTTGCCATTCATCTTGCCGTCAGCCACGCCATCAGCCTTGTTTGGTTTGACGAAGCCTCGGAAAGATAGCTGCAAGACATTTCCTCCACTGTTACACGCTACCGATCTCCAAAAATGCCTCTGTTCGCCGGCATTGCTCATTCTCTCAACCTCCGACGAAGTCGCGCGCATTCTTAGTCGTTGTGTTGCCGTTGCGCCAACTCCGGTGATCCCAGGCGTCCATTTTGATCCGAGCAGATACCATCTCCACCAAGCCGCACACGTGAGCCACATTTCAGACTCGAGCCGCTACCATCTTCACTGAGTCGAAGCCTCTTCTAGTCGAGCCATCCTTAGTTTTAGCCTTTCTTGCATCCTATTTGAGCGATTTGGGTTTAGTTTGCTATTTTGGAGGATTCAAAGCGTCGAAATTATCCACCCAAGGTTGAATTGGAATTCAACCACAACTCTAGAATTTGCCTGTTGGAAAGCTGAGATTGGTTCCAGATGATTGACAGAAAGCATTTTCATCACGCATTGGACTCTTGGAGCTGTGTAGTTAATTGTTTTCATCGTTTAAGCAAATGGGGTTTCAAAACTCTTCCATTTAATCTTGTTAGTTGTTGTACATATAACGATAATATATAGATTAGTGAAGTAATTATTTGCTTCCCAGCTAGAATAGATAAATATGTTCCAATTGCTTCAATGTAGTTTATCACATTTCAAGAGATTTAGTTAAACACCCTTCCCTGACATATCAAAATATTGACTGCTTAGAGAACTTTC

>cucumber_newGene_477 cucumber_newGene_477.3

TTTGTTATTGTTAAGTAGGAGACGGAGAACATGTAAATAACCTAAGAAAAACCTAGGGTAGACTAGGAAAGGGTGTCAAGACCTAGGCAAGCATGCATAAAGTAGGTGATAGATGTCGTTTTCAAGCCATGCAGCCTTTCTATAAATAGAGATACTTTGAAGGAGAAGAGCATTACATCCATCCATGCCATTTCCTTGATTCCACTTTCTAAACCTCACAAATTGAGTTTGGTTTGTAGTCTTCGGCTGTCAGACAAATTGGAGCAAAGAAGGTTCGAGTTTGAGTTCAGAAAAAGGAGGAATATCAAAATTTGGGTCAATCTGGACAAGGTGCACTTCTGAAGGGCTATGGAGCATATTGTTTGGCATTTAGAGTTGAACATTTGAGGTAAGGATCATCTTGCTCTGAGATGAGCTTATTGCTTTCATACCAACCAGTCTTTTAGATGGAATACAAATCATTATAGTTAATATTGTGTAGGGGAAAAAATCAATAAGTTGAAGTTGTATAGAAATCTAATTGAGTCAAACCTTTAGAGGTAACATTATGTATAATTTAATCTTGTAATGGGTTGGGCTACACTTGAATTTCCTTAATACTAGAATGAGATTTATGTTTCCATTGCATTTTAAAAGATTAAACGAGTAGGGTCAGCAGATATCATGATGTTAAGAGATGGTATTGGTTGACTTCACGCCATCTCATGGGTCGAGAGTAGGTGGTCCGAGTGGGGGTCTGATAGTATGGTATCAGAGAAAGTTTGGCCTTAGGGATATAAGAGTCTAAAGTTGTAAGTATGCTTTAAGACACTAGGATTCTAAAGTAAATCTAGAAGAGTAGTATTAAAATATATGTAGACTGGCTGGCAATGTAGTAAGCAAATAGAGGAGACATGATCTCACCCAAAGCATTGTAAGTATATGCTATTTTTCAAAGGTAAAACACAATTCATAGATGATGATTTGTGTCACTTGTAGGTAGCTTAGAACTTAACTAGCAATGGATTTAAGAGCCAAAATTTTAAATGATGGTTTAATAGCACTAGGAATTTTAGAGTTTAGTGGATCTATAAACTGTACGGAGGCAAATAAGTGGTTAAATCATCTGAAGGACTTTTTTTGGACAACAATTAAGATGCCTCGAAAACCAGAGGGGTAGAACTAGCAGAGATAGTACTTAGAGGAGAAGCAAAAGACTGGTGGAGGAAGGTTAAGTTAGAATTAAATCATGGGAAAAGAACAAGGTGGGAGATGTTTAGGGGAATGTTCAAGGGGAAATATTTTTCTGGACATTATTGTAGCATGAAAGAAAAAGAAGAATTCATCCAAGGAACATATATTATTACAGAATATGCAGAAAAGTTTAAGAGAGAGATAGAATATGTAACCATACCCTAGCGAATGGAGAGAGAAATATGCATGAAACTCATCGAAGGCCTCAAAATAGAATATCACACACCAGAAATTGTGGAATATAACTGGACTAGCCTATGCTCATTTGCAAAAATAACGAGGGAGATGGAAGAAAAAGTTGAAGAGAAAAACAAACTAAAATCTAATTGATGTAGGAATCTGTGGGCTCTTTTTTAGTAGGAGAGAAGTCTAGAGGAGGTACAAGAAGACGAGCATGTAAAAATTGACAGAAATACCATGGGGGACCTTATTTGGAAGACACACAACAACGTTTCTACTGTTTTCAACCAGAACAATATAAGAGAAGCTGTGTATTTCTAAAGATGGATCAACAAAGAGAAATGATAATTGTACCACCTCGTTTTATGAGGGCCCTGCAAGATGAGATCAGGATGCTTGGGATCAGACCAAATTTTTGTAGCTGAAGGAAGATGACCAAAATACTTCATAAAAATAGTATATATATGCATGTAGACATTCATATGTAAATAGTGTGCTAGACTATGTAGTAAGTCCATAAAAAGTCATCAAACCATGTGAAATTGAATAAGGTATCCTTTTCAGTTTAGTAGTTCTTGACCCTGCTTATGAATTATGTTAAACAATTACATAATGTAGGATTTCTAAAGATGACTCAAGAAAAGAGAAAGCAAAAGCAGATAGTGAAAGAAGAAAACATCCCATAAAAAGAAGAGAAGAAGATATACAATCTGCAGAGAATCTAGTGTCTCACAACAACCAATACTAGGAGAGAACAACGCATATCAAAAGTTATTAGAAGCATTAAATTTCTTAGAACCAAATCTGACAATAGAC

>cucumber_newGene_478 cucumber_newGene_478.1

TCCAATTCCAATTTTTACCAAAATTCTCTCTAATTTTCATTTTTTCAGAACTCTTTCCTCTCCATTCTATCATTTCTCTCAAAAACTCCAACAATCTTCTTTGATCCAATGGATGGGATAATCTTCTAAGAGGTAGGAGAACAATGAGTTCAATTTCTTTTAAAGTTTCTTCCATTTTTCTTCAAATTTAGCGTGTTGATTCTTTAATTTTTGTTCAAGAACGAATTCTACTTTCAATTCTATGTGTTTAATTAGTATGCAAGATACATTTTGTTTAGAAAATTAAAGTGCAAGATTCAATTGATTATCTTTGTCTACAAGAAGTTTCATATTGTTTCTTGCTTGTTATGAACTTTTTAATTTTTGAAAGATTGATTGTCTTAAAGCTTAGATAAATCATCCTTGTTCTGGGCGTAAATATGAGTTTAATTCCATCAATGAAATTCAATTAAACCCATGCTGCATTTTTCTCTGCAAAGCATGCAAGGTACTTTAATTTCTTTACAAGAACAAGAATTGAGCATGGATTGTTGTTGCAATGATTAAAAATGAATATCTCTTTTCTTGAATCTAGTTTAAGGCAAGATAACTTGATCTAATTTCTTAGTTAAGATTTTGATTCTCACGTATCTTTGATCAATGGCATGTGTGGATAAAGCAATTCTAGTACGCATGTGTTCGATCTTTCTTTGTTTAATTAAAGAATTGAATCTTAATCAACGTTCTTACAAGGATTTAAAGTTGATCTTGCAAAGACCTTGGCCAAGAATCTCCTTTTTAAAGCAAAACTCATTCATGTTTCTTTTAGCTCAATGATAAGAATTAAATAATGTCCATTGCTCCAGCTTGTTTTTCTCAGAATTAATTTTTACTTTTTGTTTGTTGATTAAATTTGCTTCTAAAAAACAACTTGCTTTGATTCTTTGGTACAAATTCTCTTGATATTTCCTGCACATCCTTTTATGTTTTCCTTTTTCTACTAACCACTTTCCTTTCAAGCTTTGGTTTTAAGATTTTTTGTTAGCAACCATTTCTTTGGGTTCGACACCCTAACTTGCGACACAGCTACGATAAAAAGTAAAAGTAAATCTAAAAATTCTTTTTGCAAGGTAAATTGGTGAGTTTATTACCCTTTCTATGTGTTCTTGGCTTGCAACTAGTATTACATTAATTTTCCATATCATTTTCTCACATATTGTTGCATGATAGATGTTTAACAACCTTATCAATTAGTGTATTTTCATCATATGTTTTTTTACCTTGACAGTACCTTTGAAACGCAAAACCAAAACATTTCTCTCTCGTTCTCCGTAAAACAAATCCTCTAGGCATCACCGTCGAAACCCTTCCTCCAACCGTACTGTCATATTGTGATCTTCCTAATTATTAAGTGTCGTCAAGTTTCCTCACGTTCGATGTCTGTCATCGTGTTCCTCGCATTCGCCGTCTATCGTCATCTTTTGATTCATATAGTTTGCTCGCAAATTTCCTCGTGTCATTTTCATTCGTCGTCTTCTGCCATCTGGTGCTTCTTCTCGTTCCCTCCTCCATTCGGCTCTTCTCGCAGTGATAGGTCCTTCGTCAAGTGGTTATTGCAGTGCTCCTTCATCCTATGCCATTAGTTCGTTCCCTTCACTCTCTTTAGTTGTTAGTTAAATCTTCTTTGGTGCAAGAAAATTAAGAATTGCTCAATGTTTTTATCAACGCGAGGGTAAGGAGGTAAGCATGCTTTATGTTTTGCAACCACAGCATACCTATTTTGTTCTACTTTTTTTCTGATGGATAGTGTACGAAGCAAGGTGGAGTTGTGGAGTGTGGGTATTATGTCATGTGATTCATGCATGATATAATATTATCAAGCAACATAACAATCGTTGAAGTAATGGAAGGATCAACCTTGACATAGTCACGGGATAATTTAGATGTCGCAAGATTGGAGTGTGTTGAATTCGTTCCAATGCATATTTACTTTGCTCAAAGGTAAAATTTGTAACATAAACAACTTTATATTTTTTTTTTTTGTGTGGGTTTGATTACATAATGTGTTGTTGTATTGACTATCCTGCAATTGTGGTAGTCATTGAAAAGTTATTTTGTTTGATTCGCTTAGACATATGTTTTAGGAATCGTTTTGACATGTACTTAAAAATTTGGTACTTTGTTGCGTTGACTATCCTACAGTTATGGTAGTCATTCAAAAGTTAATTTTGTGTGATCCGCTTAGTAATATGTTTTAGGAATTGTGTTGACATGTACTTAGGACCTTGATACTTTGTTGCATGACTATCTTGCAGTTATGGTAGTCATTGGAAAACTATTATGTTCGATATGCTTAGACATATGTTTTAGGAATTGAATCATGTTGACATGTACTTAGTTGAAGAATTTGTGAACCATGGTATCAATCAAATCCATTTATGCGTCAATTTAATTAGATTAGGTCACAGATTA

>cucumber_newGene_479 cucumber_newGene_479.1

TATATATATCACATTTTCTAAGTTTTGTTTGAGGGTGACGGCGAACTGTGGGTTAAAGGCTGTTTGAATTCATCCATCCGCCTAATTCTCTTCTCTATATTACCTATATTAATATTTACACAAACGAAAGCTTCAGATTTCGAGGCCGGCGACAAATTAGGGTTTTTTGCTTGTTGCTCTTTCTTCCCTACAGCTGCACGATGATCTACGATGTCAACTCTCCCCTCTTCCGCTCTTTCCTCACCCAGAAGGGAGGAGGAGCTTACGACAGAAGGAAAACCGAGGAGCAGAAGCCGAAAGAGCAAAGGTTCAAGGCCAACGAGAACAAGCCTGTCATGACAGAGTAAAACCAACATGAACTTGTAGGTTCTAGGTCATATTGTAGTCTAGTCGAATTTGGATTTCTTCAATGGAACAAATTTTCATATTCCTTGTATGTTACAATATTCTGCCACTGGACACTTTAAGGCATGATATAATCCAATCACCTCTTTCTTTGATGATTT

>cucumber_newGene_481 cucumber_newGene_481.1

ACCTTCTCAACCCTTCACGAGTACGGTATTCTCCATCTCAACTCGTCTTCATCCTTTCTCCACTCGTCTTCAATTACCTCTGATCTTCAACACCCACTTCGCCTTTTCTCAACCTGTTCCATCCTTTCTAGATTTCCATACGAAATCATTCCATCATCTCTTGAATCGTTATCAACCACCTTGTTTTCGGTTTAATGGTCGGGAAGAATAAATCCCTCTTTTTCTCTCAACTCATCTTCAACCTATTATGTTGTTTTTCTTTGGTAGTTGTTTTCTTGTAGGAAAAAATCTTCTCTTCCCCTAGTATTTCTTTCTCTCCCACTCTTCGTCCTTCAAAGTTTTGTTGAAGGCTTCTCTCACTGCGTTGCCTCCGAAACAAATGAGTTTTCCCTTTCGGTTTTGTTGAATGACTGGGTTTAGACTTTGCTTCCGAAACTCAAACGATTGCATGTTCACCACTCGTTTCGGTGAACATGCAATTTCAACTTGGTTTTTCCCCTTCCCCTTCCACTTTCCCTTTCTGCTTGGACTCCCATTTGTCGAGTAGATTTCTTTTCGCATTGATTTGGACACTTGCTACACTATTTTGTGCATATGGGATGTTCGAGCAAGGTATGGTTGGAGTTAAGCAGTTGGAACTAGAGTTTTTTCAAAGCCGATGATGAGGATTACAAAGTACTTGTCTGATTTTTTTTTTTGTGATTAAGTTTGTTTTCATCCAATTTTCTTCCATATATGTGCATATTTCGACATCACGATTAGAAACCACATTGTTGTCTTTTTGAAATTGTTTGAATCAAATTGTTTTTATTCTTCCTATAATTCCTCGTATTGCAATTTGAGAAGCACCTGATTACTGAATACTTGTGTGACTTTTTTGTTCGTATTACTTTTGTTTTCATTCAATTTGCTTTCATATATGTGCATATTTGAGCAATTGGAATAGAAATTACATCGTTGTCTTTTTGTAATTCTTTCAATCAAATGTTTTTTCTTCCTACCATTCATCTTATTGCAATTTTGGGCATTTTTTGTAATGGGTGTCCCTGTTATGTTTCCATTTTACTCCTTTTGTCATTTGATGCTTCCATGTTTTCGGTTCGAATTTAGTTGCAATAAATATGCCTACTACTATGAAGTATGTTATATCTATTTCCAATGAACTCCCATCCATCCATAACCATGCAATGTATTTGTTCAATCGTGTCGTTGTGTGATAGCTTATGCATTTGTCTATTCATAGCCTTTCTTTGTCATCATTGTGGTAGGTTTTGACTATCTTCTCCCTTTTATGTCATTTGCATAGATTCCCTTCTTTTCTTTTTGTTTTGTTTTGGGGTCCATAAAAGCATAATTTATTGACCACCTATCCATCTATATATACCTCTTCATTTGTTGTTTTATTGTGTGCTATTTTTGTGTTGATTTTCTTCTTCGCTTCCTCCTGTTGCTTCTTATTTTGGTAGGTAAGTTATTTTTGCTTTCTTCTTTTTTAAGTTCCAGTCATCCTTCTACATTTATAAATAGTTAAGATTTATGTTACGAATCTTGTGTGGTTGTAGTTGCTTTCAGTTCTATGTTCTGTTTAGGGCATAGTATGTGTCATGACTTCCTTTCTTTATGCATTGATATACCATATTTCACACATCCCTATGAACTTGCCAATCATGCTGAACTATTTGTTGCAAACTCTGATTTTATTGGTTGTTCATTTTCTAAGATTGTTTGTATGCATTTCCATCCCCATACTTCGACTGTATCTCCATTTCATGCATACTTTATATCGATGTTTCCACAACTAGGTCTAGTTCGTTATATTGAACGGATTTAATTACTCTGAGGAACGATCTTTTTTAATATAAAACTGTGCCATTTTTTTAATCCTTTTAACGGGTCTTTTTTTTTTTCCATTTCAACATCAACATTCTTCCAAAGTGTACGGTACGTAATCGCTACATTTTGTCCCTATAGTCTCTTTACTGCTACTTGTGTTTGCCCGAAAAACCCATTTCGTTACTTTGTTTTCAGGGGAGCCGACTCCATATTTTCGTCGTGAGAGGTATAAGGCATTGGTGTTTGTATCACCGAATGTCAGTTATTCCTGATTTTGGAGGCATTGAAGAACGACAATAAGCGCGTGACACATATACCGTATGAGACTAGACATCAAATTAGACAACTTGCCTACTTTTGTATGATACACGCCTCTGACCTCACTTGTCGCCAAAGTACTAGAATGGATAGAAGAACTTTTGCCATTCTATGTCACTTACTAAGGACAAGTGTTGGTCTGACGTCTAC

>cucumber_newGene_482 cucumber_newGene_482.1

ATTTTCAATTTCATACTTCGTAATAGTCATTTTATTTCCTTTCTTCTGATGGCTCGGATACCAGTTCTTGAAAATAGTAGAAACAAAAGTTTTTATTTCTTTGTAAAATCAACTTTGAGACAAGTATATTCTTTTTGGGGAAATTAAAAGTAGAGTTACCTTTTTACTCTTTTCTTTTCTCCCACCTTTTGGACTCGTTTGCTAGTTGTCATTTACTTTTCATTGTACTTCTTTTCTTCTTTGCATGGTTCACCGAGGGAGACAAGGAAGAAGGAAGAAGGAAGAAGGATTTCAAAAGGGGGTTTCTTAAGGAAGATGAAGAGATGAGAAAATCAACAAGATGAAGTTTATAGTAGTTCAGCATATAGCTTACATCCACTCTTCAAGAAGTTGAGGCTCTCTTGCAGAAGATGGTTCAACAAATTTCAACACAGGTTGCTTAGATTTGTTAGAAGGAAGAGGGGTGATGATAGTTCTTTGGAGGTTGAAGAAGAAGAGCTTGCTTCAGTTCCCACGGTGGAAGAAAGGATCGGAGCAAAAAAGGAGAAGAAGACGTGGGTCAGAATTGCTCTGATACCATATAAACTAATGTCCAACAGTTAATAATTGAGGTGTTTTCTCATTAATTTAACTGATGGTAATTAGGAGTATTTACAGTACTCTTTCACCGTACAAATTTGGAAAACAAAATAACAAAAATAACTAATTATAATACATAATTTATACATGATAATACCCACCAACAAAATCAAAAAATATATTTGTAAGATGATATACTTGTACTTAAT

>cucumber_newGene_484 cucumber_newGene_484.2

ACAACCTCCGCTCTTCGACGGAACCACCAGGTTATATATGGCTTACTATTGCCCATTTGCACAGCGTGCTTGGATCACAAGAAATTATAAGGGATTACAAGATAAAATAAAATTAGTGCCTCTGAACCTTCAAAACAGGCCTGCTTGGTATAAGGAAAAAGTGAACCCTACAAACAAGGTGCCAGCGTTGGAACACAATGGGAAAGTTATTGGTGAAAGCCTTGATTTGATCAAATACATAGATAGCAACTTTGAAGGCCCTTCTCTTTTCCCAGACGATGCTGCTAAAAGACAATTCGGTGAAAAGTTGATAGCTTATACTGATACCTTCACTGGGGCAGTGTACCCTTCATTTAAGGGTGACCCAGCTAAGGAAGCAGGTCCTCAGTTTGATTACTTGGAGAACGCTCTGCAAAAATTTGATGATGGCCCATTCCTTTTGGGTCAATTCAGTGGGGTGGACATTGCATACATCACATTTATCGAAAGATTTCATGTATTTTTAAACGAGGTTTTCAAGTATGATATCACAGAAGGAAGACCTCATCTAGCTACATGGATTGAGAAATTCGAGAAGATTGATGGTTACAAGCAAACAAAATATGACCCTACAGCGATTGTTGAACTTTACAAGAAGCGCTTTATGGCTTAAACTCACAACAGAAATCAGAGCTTCAACATTATCATATTGTCGATCGATAAGCATGGTAGGCTTGAGAATTTCAAAATTGCAACGTTTATTTGCATTTTTGTGTGTATTTTTAATTTAAGAGATCCAACCATCGGTGGTCGATGACAATCAACAATGTTGTAGAGTATTACTCCAAAATATTTGCATTTGATTTTTATGTAATTTTTCATTTGAATGTGGCTTAATAGATAAAATGGCTGATATCATTTTATGTGGGTATATTTGGTTATTGTTTTTG

>cucumber_newGene_485 cucumber_newGene_485.1

ATAATCATGAAATTTATAATCCTATCAATTCTATAATTTTTTATATGGCCCCACGTTAAACCAACATTTGTTCACTTTATCATTTTTTGAAAGGCTTTTCCATTTTTTCAGAGGAACAAATTTTCTGTCTCATCTTTTAATTTATATTACTTTTTACATTAATTCCAACTGCTCAGAAATGGGTGTCTGTTAGGACTTGGGATAGTGTCCATTTATCCTGAAGATGATAGTTTTAGAAGAAAAGATTACTAAACAGATTCAGTTTTGGGCGTCTTGGTCGATGCGGTGGTTTTAATCTCGACGTCTTCACCTTCCTCCTCTCTGAACTTCTTATAAATAATCGCCCTTGTAAAACTTCCATGTCCGAACCACCAAAATTAGGGAAACAACTCCTCCCAACACCGTCGTCCCCGTGATTATAAGAAATGCCTTTCTGTAACACTCCACTCCCAAGCAACTCAAATTCTCCCCTGCTCCCTCCATCTGCCTTCAGCCTCCCTGTCGTACAAATGACCCATCACTTTCACATTCATAATATACGTCCCAATAGGGTTTGCAGCTACTCCCAAATCGCTTAGAGTCGCATAGTACTTCAACCCAAAAATTTCCGATACGATTGCCAAGATCAATGGCAACTGCGCTCCAAAGCAGAACCCAATTATGATTGAAGAAAAATACAGTGAATGTGGAACTCCGAATGCGATTAGAAGATGGCTGACGCAGGATAAAATAAGCGTCCCGAAGAGAAATAGAGGACGTGGGACTTTGTACTTTTTCCAGAAATATTCCGATACGAATCCGGAGGCTACACGGCCGAGGTAATTCCATATGCTGACCAGAGAGATGAATGTGGTGGTACTCTGAGATGGGTACCCCAAAGACTCTCCGATTTGACCTAAATTGTCCATAGCCGCCAACGTTCCACCGACACCACAGATTAAGGCGATAAAAAGAATGATCATATCGACGCTGAAAATGGCTTGAGGGATTGTGTAGTCCTCTCCTCTGTTTGAGGGTTTAAACATGTTTTTAAAGCAGGAGTCCGACGGAGGGCTCAGTGGCACTGCCGAGATAAGTGGCGGCGGTGGTTGTTGAGATGCTAACTCTAGTTGTCCATTTGGATTTCCGATTTTACTCTTCCAAACAATCAGTTCCTCTCTGAAAGCAATGGCCAGAGGCAGTAAAAGCAGAAAAATCACAACAATGGCACTTCCGACGTATTGTATTTGTTGAAACCGGGTACGACCATTAACGACCCCGCAAGGCCGAGAGAAATATAAAGGATATGGTATAAAACTTTGGCCTCGTTGGGCAGCCGGAGATCCTTAATGATGCGAACGGAACGGAAGAAAACAACAGTGATGGAGGCGGGAAACCAAGCGATTAAGAGAATAAAACATTTGGAATTGTTGCCATAAAAGGCAAAGTAAAGCTGGGACAAAATGGCGCCGCTTAAGCCAACGTAACCCTTCAAAAGCCCCAAAACACAACCACGATTTTGCGGGAAGTTATTGACACAGGTTACAATAGCCCCAGTATTTGTGAAGGTCTGAGAATTAGCTCCGATGCAAATGTAGAGACACATATGCCATATTTGGGGCTTGGGGATCCGTTTGGTTACGGCTAGCCAAATCATGGTGTAGCCAAAAAGGTTCATGACGGCGCCGATTAGAAGAATCATCCAAGTCGGAGCTACCTCATTGATGAGACCGCAGATGATGCCGGCATTGCCGCCTAAGGCCTTGAAGAAGCTGAGCAAATTGAGGGTGGTTTGGTCGTAGCCAAAGGCAGATTTGATGTCGGAGGAGTAGAGGGCAAACGTGAAGGCGGCCCCGGAGACGGACATCATGAGAATGGAGGCAAAGGCTGAGAACCACCGTCCAAAGAGCAACTGATGCCAGAAGGGGTCGCCGGAGGTTGCCATTTTTGTTCCGGATGATATAGTGTCTCTCAAGAGCATTGATCTCCTTGCACCTCTTTGAATGAATTTGGGTCTCTATCCAAGTGAATGCATTCTAAATGATCTCGGAGCACTCAACCAACATCCTCTGTTTGATTAACAAGATGACAACAACGCTCTTTTGGCCCGTTGATTTGCAAGTAAGACATTGGTTTAATTTGATTATGTTACTTATGTTTTAATTTTTTCTTTACCCTGAATGGTTT

>cucumber_newGene_486 cucumber_newGene_486.1

GGTTGTTCGAGATTTTACTTGTATGGATTATGTGATGGGGTTTTAAGTTAAAACCCGTGGCTCAAACTTTGTCATCTTTCTTCCTCATCTTCTCTCCACTCTCATTCAATCTCTTCACGATTATCCGCATCTTAGTGATCTTCACCCTTCTTCTCTCTACTCCCCTGCTCTCTCAACTCCGCCCCTCCCAATTATGTCCAAGAAAGGGCTTCCCGGTGGAGCCCCGCTTTCAAAGGACGCCCCTTGGAGGGCCTCCGGCAAACCCATTCCCAAAATCAACCATGCTCCTCTGCTCCGCATTTCCCAATCTCCCTTTTCCAATTACGCTCTCTCTGTAATGAAGCATCCTGATCCTATTGGAAGTGGCTTGGCTACGGATGCCATTTTGGAAGCCGCTGGCCCTGATTGTATTGTGCCTGGACAATCCGCACCTGTTAAATTGCTTGGTCTCAAGGTATGGCCCATTGATGTCGATCTGAAGTTTCTTGAGCCTGTTGGCCGTGAGCTTAAATTACTTGGAAAGTTTATGGATGATGCGGTGGACCTCATGAACAAGTCGTTTATTGATCGCTAGACATGGCAGTTCTAATCGATGATGAAAAGTTGCATAGAAGCTGCAATTTTAGCTGGCTCTATGAGTGGCAACTTATACACCGGAATTGTCTTTTGATGATTGACTCTTATTACTAAATCTTCACATGTAAAAACAGTCTCTGGCCTCTGAAAGCGGTTTCGTTACTCCCGTTTCTGTGGCTTTTCATTTTGACTCTCAATAGTGAAGTGCCTTGTAAGGGTTTTCCATG

>cucumber_newGene_487 cucumber_newGene_487.2

CCATGAATCAATGACTTCTGTAGATACATGGAGTTTGCAATAACTTATAGACTAATCTCATTGGGTTAGATGTGAGTTAATATAATTGGGTTAGATGTGAGTTAATATAATTGGGTTAGATGTGAGTTAATATAATTGGGTTAGATGTGAGTTAATATAATTGGGTTAGATGTGAGTTAATATAATTGGGTTAGATGTGAGTTAATATAATTGGGTTAGATGTGAGTTAATATAATTGGGTTAGATGTGAGTTAGTTGGAGAAAAGATGTTGTAGATCAAATTTGGGCAAATGGATCCAATTTCTACTGAATCAGCTTAGGGCTATGAAGATATAATATTCTTCTGTGCTCCAAGTTTTCATATATTCTTTTGTGCTCCAGCTCCCGCTTCAGGTTTCTCTTCCTTTGCTTTGAATTTGGACTGATTTTGACCACCCAATAGATGTTTATGTACTTGCCAGTTGATGCCTGATGTTTTCATTGCATCCTTGCAGAACTTTGATACATTTTTATGGTTCGTGAATATTTCATTGTTTTTCGCTTTTTTCCAAATAATTTAATGAAATTTCTACTAACTAAAACACCATTTTAAAAAACAGATGGAGCAAATTTAGACTATTAGTAATAAAGTATATTAAATTCGATTATTAGGATAAAAAGTCGTAGATATATATAAATTTTCTTTTCAAATTTGGTGTATAAAACCTCAACATGTTAGAACTCTCAACTCCCTTAACGATCTAGCTTCTTGCATTTGTGTATGATTTTTGCTGCTAAAATATTTTTCATTCTTAGTTCTACTTATCTACATACATATAATACTGCCCTTGTTCTTTGATTGTTGAGTTCAAGTTATTAAGTGTTAACTATTGTTTTGCGAGTATCTCCGTGCATCTTCTAATGGTGGCTCAACATCATCTAGCAACTCCTACAAAATTAAATAACACAACAAATTAGAAAATCAATCAGAAAAGCCATTCAGCCGAATATGGCTGCCCAACTAAGAAACCAAATATGAAAATGAAAAACCAAAAAACCCTTTAGATAAATACATGGAAGCCCAACTAAGAAACCAAATATGAAAATGAAAAACCAAAAAACCCTTTAGATAAATACATGGAAGCCCAACTAAGAAACCAAATATGAAAATGAAAAACCAAAAAACCCTTTAGATAAATACATGGAAGCCCAACTAAGAAACCAAATATGAAAATGAAAAACCAAAAAACCCTTTAGATAAATTTTTTGAAATGAAACGTTTTAATTGGTCATTTCCCTTTGTCACCCGATATGGCAACCCAACTCAGAAACCAAATATGGAAAAAGGAAAAACCCTAAAGACATCTATCATCTTCTCCATTTCCTCCCTCAGGTTCTTGTTTGAAACAACGGTAGATTGATCTCCAATCTCCACCGCACTCCATTATTTGCAAGCCTCACCATTCTTCACGTTAGTATTTTCATCGTAAACATAACAATAATGGTGGTTTCTGATGTTGTGATTCAAATTCTATCCAAGCTTCCACCACAATCTCTTCTTCGATTCAAGTCTGTTTGCAAATCCTGGTATCATCTTATCAACCATCCTAAATTTGTAACAAAACATCTCTTAGATTCTTTTCCCCATAAACATGTCCTCATCAAACGTGCCCTCACCAACCACTCTGGCAAACAAGAACTTGTGTTCTCCATCCTCAAATTCTCTCTCAATGGATCTGTGTCTATTATGGACATCAATTTGACATTCCAAGAGATCGATCCACTTTTAGAACTTTGTGGCCATTCTCACGGCTTAGTTTGTCTTTCCGATTGTGACGAAGTGTTTCTTGTTAATCCTATGACCAGACAGTTTCATAAACTTCCGCCATCGATTCTTATCTTTCGTGGTTGCCATCATGACGATCCTGATTATTACTCTGCAATACCATTTACTATCGGATTTGGGTATGATGCAAAATCTAGCGATTTCAAAGTTGTTAGAATTGTGAGTTGTCGAGGACAAGCAAAATCCAGTATGAGAGTGGAAATTTATGATTTGAGCAAAGATAAATGGAGAGAAATTGAAGCTCCTGATTTGTGTGGCAATGCGAGATTTATACCTTCATTTGATATGTGTCACGAAGGAATATTTTATTGGTGGGGATATGGTGAACCAAGGATTAACGAAGTGGACTCTATTATAACATTTGACATGAGTGAAGAGATTTTTGGTAAAATTTCACTACCGGAGAGCTTCAATGACACAAAGCATAAAATAAGTTTAAGGGTTTTGAATAAATCCATTATCCTTTTTGTCTATCCATTTGAAAGTAATGAGACAAATATTGACATTTGGGAGATGGAGAAGGATGAATCTAGTGTTGTTTCGTGGTCGAAGCTATTGACCATTGACCCTCCTTTTGGAGTTGAGCATCCATTGTTGTTTGTAAGCTGTGAAGAACTGTTAATGGAGTCCAGTGAAGGACATGTGATTATGTATAACACGGCAACTCAACTATTCAAAAAGCTTCCAATAGAAGGGGATGTAACTTATGCAAAACCTCACAGATTTGAAGCTCATGATCTTTTTATTGAGAGTTTGTTACCGGTGGAAGGGGGAAGAGATATGATCAATTACGATTTTTAGTATTTTCGGGTTTTACTATTGTAAGTCATATTGTTTGATGTATGTTCTTTGCGTGATATGAGTTTTAGTATTTTTCAAATTATTTTAATTAATGTTTAGATTTTGTTACTTAGCTTTCACACAGTAAAATTGTATTGCTTTTAGAGTATAATGTTTATTTTTAATGTATAGATTTAATACATAACAAAACTTATTTCTTT

>cucumber_newGene_489 cucumber_newGene_489.2

GAGATCATACTACATAATGTCTATGTTCTTTAGCTCAGGACAACTTTGACCCCGAACCATACTACATTATGCACATGTTCTTTAGCTCGAGGCTATCTCAGGCCGAAGCCCCGATCTAAAGAAGATAAAGTAATGTAGTACGATTTCGACTTAAAGTTGGCCTGAGATATAGCGCCGAGCTAAAGACCATAAGGAAATGTAGTGTGATCTCAACCTAAAGTAGGCCCGAAATAGCCCCAAGAGAGACCAAAAAATTTCCGTCAAATAGTTTTATAATTTTGGAAAGAATCTCGAGGTCGATCTCGTCCGAGATCGACCACGAGAAAAACAAAATTTTCAGTTGTCCTAAGATGCACCCGACCGAAGGAATGGAACAGTGTGTGGCAAGGAGTGAAACATAAAATAACGGGGTAAAATAACACTAATTCCCCTTTTGCTTAATTCATGCCCTAAAAGTGGAGTTGGGCTCAAATCTACACCAGAAATGCAGACTATTATAAAATCAATACTATTATAAGTCACTTTTCGCCCCAAACGCCCTCATAATTTTTCCAATTTATGAGCTCTTCAAAAAATTTTGTACTTTGGTATAATCCAAATAATGGCTAGGGCCCTTGAATCCAAATGTTGAAGTCCAATGTCCAACCTTAAACTAAATTATCTGAAGTGACTCACAATTTGTTCAATTTTCGAACTCATCAACCAAGTTTATACTCTTCGACCTTCAACCCCAATCCAAATCTTGAAATATCTCCAACCTCAAATCAAATTTCTCTAATCTTCAGTGTCTCTTAATTTGCTTATTTTTCAAACTCTTCAATCGAACATTAATGATCGATCCTCTTTACTCAAAAAGTTGAAGTCAAATCACATTCTTTAATCCAAAATTCCCTTATCTTTATTTATTTGCCTTCATCGACCGTGGAATAAATAACTACCAACAAACTTTTGGTTTCCCATTTTCAAAATAAAATCCCTAACATATTATGTTCCACACACAATCACTTAAAAGTCAATTTTATTCACTTCCCATCCAACGATCACCACCTTCATCCAAACATTGACATGTTGCCTTCATTCAAAAAGTTGTATTCCAATCTCCTACTTCAAACCTAATTTTACCAACTTATGAACTCTCCAACCAATATTGTACTTTGTATACCAAAGTTTGGCTAGCCCCCTTAATTCCAAATAGCATAAATAAAAGCGCATACTTTAATCCAAAATTCTCTTATCCTTTTTTATCTATATCTCCATCGTGAAATGGATAACTATCAACGACTAATTTTTCTTTTTCCTCTTTCAATATATCGTCCATGAGTCCTATATATACCTTTACTCTCCTATTTCTTAGCTTCACGTCCAATAATCTACAATTTAATGCAAAGAACAGCTTTGGGACTTCCGTAAAAAACTTGTAGTCCCATCTCATAATTTAAACCATTATCTACTCTTGTACTCTAAATTTTTTCCAAT

>cucumber_newGene_490 cucumber_newGene_490.1

CGCACATATATTGAGTTTGCTAACCTGATTCGCAATCAATTCTCTTGTCCTATCAAAACTCTTCGCACTGATAATGCTTTGGAGTATAAAGAACATAAAAGTTTCCATTGTTGGGATCCTCTTTCTAAAAGACTTTGTATATATAGACATGTCACCTTTTGGGAACACACTATGTTCTTTTGTTTGTCCTTCTTTCACACCTCTTTCACAGGTCCTCGTCATTTCTTTATTGATACATCTGTTGAACTTTTTTCTTTTTCTAAATTCAATCTAGATACTGAGCATGCTCAATCTACACCTGCTCCTGCAAACTCAAACTGGTCATCTGTCTCCAATGATGTTTCTGAACTTACTCTAGATACTCCTCTTCGTCGCTCTACTCGGGTAAGAGAACCTCCCACTCATCTCACTAATTATCACTGTTTTTTCACCATTGTTTTCCTTGTTGAACCCACCTTTTACCAAGAGGCCAGCACTGACCCTTTATGAAAAATATCAATGAATGATGAATTACAGGCTCTTGAAAAGACACACTTGGAATTATGTTGATTTACCT

>cucumber_newGene_491 cucumber_newGene_491.2

GGGAATTCATGCGTGCAAACCATCCTCTCGAAACTTCCTCTCTTGTTCTTCACTCACCCCTCATGGACGCCAACCCCCTTGTGTCGTTTGTACCACTGCCAATCGTTCAATCACCACCGTTTGTCTCCCATTGGCCGTCAAACCCAATCGTCGTCGGTTGTTCGTGGGGTGTCAGTTTTGTTTGCGGCCATTCAGCTCCAACCACCGGATCCGTTGAGCCACGCACCGACTGTTCACAACTTAGTTGTTAGGCACGTCAATTTCTGCTCCGCGTCGTGCTGCGCCACCCTATGCCACCGCTAGCATTGCCGAATCTGGAAGTTGAGGTTAGCTTTGGGTTGTCCGGTGAGTTTTATGTGTTTTTCTTGAAGATCTATACTACCCATTAGCCTTTAAATGACAAGTTTCTACCATGTTTTGCTATTAGATTGGAGAGTAGCAGTGTCGTTTAGTTATTGTCTAGCGGATTTGATAAGGTTTTCGTCAAGATTTTGGTAGTTAGTAAAGATTTCGAGTCCTTCGAGATTTTAGTCGTTGTCCAGCCATTCGGAAGCTCAAATTTAGGCGTTTTGGCATCACTTCGGTAAAATTCTAAGATTTATAGAGTCGTGAGTACCCGTCTAATATTAGAGATAATATTGAATAACCCATGATGTTGGTGTTTTTTTTTCGAATTTAGAAAGTACTTATGTTGTTGTCCATTGAAGTTAAGGGTACTTTGTTAAGTTCTTGGTAGGGTTTGGAATAGTTTCCACATAACATCTCTGGTTTTTATTTTGGTTTGAGCTACACAAGAAGAGTTAAAATGTAAAGTTAGATTCTTTTGAAAGTCAACTCAATTTCTAACAAGTTTCGTGAATAAAGCTTGAGCAAATTCTAAGGCTAAATGAATGATCTTTTGAAGTAGAAAACAAGTCACAAACTTTTTACGCAATTTTGAAAAGCCTCTAGTTTTTTAAGTATTCTAGAGGGTACAAATGGAACTAAAAGCCCTATTTCTAAGTTATTATAGAAAGGCAAGAATACTTAAGGTCGTTTAAGACAAGTTTGGATAAGTTTCTATGTATTTGACTTGTTTGATTGGTATTGAGATTTTTAATGGATCTAAACCTCTAAGGTAGGAATATTGAATATAAGAGCGTATGGATACAAAAGACAACTTAAGTTTGGGTGCTTATGACGTTAAGAGTTAAGTTGAAATTTAAGGGTTCCAAAAGTGTATTCACCTAAAAGACCCATTGAGCTTGAACTAAATTGGATGCTCATTGGGTTAAGTATCTAGATTTCGAGTCAAAAGGTTCATGTATGAAAAGCTTAGTCTAATCCCAAGCGCTTTGGAAATTCATGGACCTTAGAAGTATAAAATGAATTTAGGTGGCCTTATAAGGTATTTTGCAAATAGTTGTAGTCTCGTGCTATGTGGAGACAATGATGAGACCTTAATCTATGTTTTAGGCCAAATTGAAATGGGAAAAGCGTATAATCTTGAGGGATCGAGTTTTTAAGGTAAGTAGTTACTTTCAAACATTTGATATGAAAATTGTTTTAAATGTAGCGTTGAACTTAGTTTGTGAATAAGTGTTAGTATGATTACTTGCTTTGGTAAAAGTGTACTATCAATAATGATCTCTAACAAAACACTTTGGCTTTTCATGAGTTCTCTACAATGTGAATTTGTAATACGTGTTCTTGAAAATGATTTGATTTAATTACGATTTTCGTGAGTTATGATCAAAATGTGTTTTATAATGAGTTGATAAAATATGTTTACCTTGGTTTTATCAATGAGAATGTTTTATTTGTTTATTAAGAAAACTCTATTTTAGCTCTGAATTGCTTGAAAATGTATGAAACATATATATAGATGTTGGTTTATGATAATGGAATGTGATCGAACCACAAAATCGATGTATCAAGGTCATTTTCTCGTAGGCTACATGAATGTGTTTCTGTAGCACCACCTATGGTTGTCAGGGGGTTTTGGGACTAAAAGGGTCATGACCGTAGATCCAAGAACCTGAGCCTAGGTGAGGACGTGGATGGAGGATTGTGATATGGCTTCGAGCTTAGGTTAACCCATAGTGAATGGCTCTATGTGCAGATAGGAGCGGATAGATGTTGCTATTGTGGTAGGTGTTAAATATTGTGAGCTCTGTTTAGTTACGTGTTTTCTTGTGGATATAGATAACGTGTGAGCTATTCTGGGTCGTGTATTTAGTATGCTATCATGGTTGAATCAGATGGATGTTGTTACTGTGGTAGGTGCTAAGTATGTGTGAGCTCTGTTTGACCGCGTGTTTCCTTGTGGATATGGATGGCGTGTGAGCTATTTTGGGTCGTGTATATCATTGTCGAATTGGGTGGAAATTACTATCGTGGCGGGTATTAAATATGCGTGAGCTAGTTGTGGCTATATGATTCTTTGATCATGTGTGAGCTATGCTTGATCGTATATTTAAGTGTCTTGCCATGGTCGAATTGGCTACAGGTTTGTTTGTATTACTTATGAATTATGGTAATAAATGATTTTAACGTTTTAATTATTATTTCTTTATGGATGTCACATTTAAAAAATTTGGAAGTATGTTTTACGAACTTTTAATTCTAAAAACTTATCACTCATTAAGTTTTATCTCACGTTTTCAATGTTTTCTCCTCCTCCAAGTAGCGATCAAATACTAAGCGTCGGTTGAATCTCCATCATCGATTGTAGATCTTCATTTTGTATGTATATAGTTAGTTTTGTGTTTGCATCATAGGGTAGTTAGATGTAGCAAGAGAGACATTAAGGACTTGTGCTTTGAAAGATTCTTGAATAAATTGTAATCATTTTGTATGTAATATCAAGCTTGATTCGTAATCTCTACTTGAATTCTAAGATCGTTTGGATGTGTGTGGTGAGACACACTTGTTGTGTTGTATTATCTAAATGAAGATATCTCCCCTTTCAGGTTTGGCTACCCTGTATTTTAGTGGAGGTGGTGCCAAATTTTTTATAGAATTTTGATTGAAAAAGTATTTTATTAAACCTTATATTTGAAAAAGAGAATGTTTTAAAGAGTATATTCACACCACGATCTAGGTTTGAGGAAAAAACTAGAACAAGATGTGACAATTTTGGTATCAGAGCATGGTTTTGGAAAACCTATGGGTTATGAGCATGGAGATAAGAACTAACTTGTATTAAAGAAG

>cucumber_newGene_492 cucumber_newGene_492.2

CTGGAATCCCAATTCCGACTTCCGATATCAATGTCGTTTGGTTTCGTCTCCTACGATTCAATGAAGGTCGAATTCTAGGGCTTTCTCTGTATCAGCTCCGTCGTTCTCTGTAGCGCATTACTTGGAAAGGAGCGACGGGTATACCATGCATTCAAATATGACTTGCTTCTGGCTGCATAGGACCATGTTGAATGATTATGGCTGTAAAAGAAGAATTCACAGTTGAAATTTCTGCACATGAAGTTGAGAAAGCTTTGATCACATTTCATCTCATGACGACAACGTTGCGGCCCCCATTCACAAGCCAAATGAGATTCTCATTGTTGCCAGGTGGAATCTTGTTAACAAACATAATTATTTTCACTTTGCCTGTCAATATGCCACTGATCAATAGATTGCAATTATATTTCTATGGCCAATCATTGATATCCTCCTTTCCACTTTTTTATGCCTCCTAAAACATAAGCAATATCCTATTGGTTTCATAAGGATGTTTCTTTAGTAAACTGATGTCATTTTCTTGTTTTGAAACTGTGGCTCTGATGTTTACTACTCTCCTATTAAATTATTTGATTAATTGTGGCCACCTTATAACATGAATGTGAACAAAGAATGGTGGCCCCTTACTTGTACATGATAAAGAGATCTATTGGCCTTATTAGTCACTGGATAAAGAAAATGCCTTTTGCTTTTGCC

>cucumber_newGene_493 cucumber_newGene_493.1

CTCAATTTCCTCCCTTTCCAGAAACTTCAGTAAGTTCAATATTCTTCTAATTCTTTGTGTTCTTCGTTTCCTTAGATCTTCCAATTCGCTTAATCTTAACTTTTACTCAGCTATCATAGTCAGTACCTCTGTAACCCGCTTCTATTTCTTCCTCAAGGTGATTCTATTACTTGAATTTCTTGTATCGACTCTAATACACCATTCTTTTTTTTCCTTTAGATATTTTGTACTACTCTGATTATATTTGTTCATAATCAAAGTTGAATTCGTTATAAAAGTATTTGAGTTTTCTGTTGATGATAAAGCCGAAATTTCTACGACCTAACAATGTCGACATCTATTTCTCTAGGTTTACCTTTCTTCCTCTGTAAATGTGATTTTCTATTCTTCTGCAGTAATTTAGGGAAAGAAGTGATATGGGTTCACGATTGAGAACAATTTATCTTTATTGGTATGAGATATTTTGGGTTTTGGGTTTATTCCCAAAAGTGGAAAATATCATATTAGGGAATAATGACCTTCCCTTACCTCCGTTACTGCTGCTAGTGCTCCATTGGAGGCTCTGATGTAAGTTTCATTACTCAACTTTACTTTCTAGGATTACCGGTGGAAATAAGATGTGCCCTTGATATGTTTTTTTCTGATCAACGGCAGGGTACATAGTACGGCTTTCTTCACTAAAGATTCTGTATTTTAATCAACTTTGCTTTTGTTGTTCAGTTTAGATTAGAAGGGTATAGTGCTTGAAATGGCCAAGCGGGATGCATATTTATTCAATGGTTGCTAATCATTTCAGAACTGCCTTGCTGACACACATAACGTTAAGAGGTTTTTGATTTTGATGCATTACTCGTGAATTTGATTCTTCAGGAATGTTACGGGGGACTGCAAGATTGAAGTTAAAATAAAGTTAAGGGTCATTATAATATAGGAGGACCATTGTTTGTAGAATAGCTTTCACTTCAGAATTAAGAAAGTACGTACGGTATGTGGTAATGAATAGAGGATGAATGAGCACCATTAACTCATTATACTATTTGCAGGTTGAGCTTGAAGCTTGAATCTCGAAGGATATTGTTAGGAATGATTTTCAATGAGAAACTATCTTGAGTTGGGTTGGTTCCTTTTTAACTGCCATCGTTTCTTTTGGTTTGAGGCAAGTTCGCCAATCCAAGTTTGGAAAAGGTGAAGTTTATGAGTGAGAGGTTCTTTCAAAACAGATCTTGAGGCTGACTGTTCTTCTTTGAAATCTTTGTTACTGTACTCAAAATATGAAGGCATGATGATAGAATAGTCAAGTAAAGTGCCACCTGTTAGATTTCAAGAGCTCGATCATATTCTTGGTTGGATTCAAAATCCATTTCTGTTGGGTGGTAACTTTAAACATGAAATTTTGAGGATTCATCATAAGTGTTTTAGAAAAGCACCTCAAGGCTCGATCCAAATGAGATGTCCAATAATTATTAAGAGAAGCCATATTTGGAAATGGATATCCATAGGTTTGCTTTGAGCAAAAACATTGGTAGATTATTCCGAGCATACTGTAGATGAGACAATTATGCTTTGAGAAGATGAACAATGAAAGGCTGCATGTAATCAATGGAGGTGTGGTAAGTATTAGGAAAATTTTCTCTATGACATCTTTTTGTGTTGGG

>cucumber_newGene_496 cucumber_newGene_496.2

CATCTTTTGGGAGTCGAAGAAATTTGAAGGCAAGATGGATAAGGTTGAGAAGTGGTATGGAACTGCTTACTCCATCGAGAAAGTTTCTGGTGCTCTGGTTCAGGATTGGATGCAATCTGCTCCTGATGTGAATTTGCATCTACCAGCTTCGAACATTTTCATCCCTACTGATTTATCGCTTAAGCTTGCATGTGAAAAGGTCAAGTTTCCTGTTCTTCTAAGAAAGTCGTCATATTCATCAATATGGTACAAACCCGACACAATGTTCTCCACACCCAAGGCATATGTTAAGATTGATTTTATATGTCCCCATGCTGACATCTCACCTGAAGCAGAAGTCCTAACGCAAATTTTCACGATGTTATTGGTGGATTATCTGAATGAATACGCTTATTATGCGGTGGTTGCTGGGCTATCTTATGGCATAAACGCTGTAGATTCTGGTTTCCAGGTGACTCTGAATGGTTACAACCACAAATTAAGGATTCTATTGGAAACTATAGTTACAAAAATTGCAAATTTCAGTGTGAAACCTGATAGGTTTCTCGTCATCAAGGAAACTTTGTTGAAGAACATCAAAGGCTCGATGACAGGTTTAATCGCCCTATCTGCAGGCTTTGGACTATTGTTCATTAATTCTTGGAGATCAGAAATGGCCATTAATGGACGAACTTGCAATTCTTCCACACTTAGGGGCTCGAGATCTAGATAAATTTGTTCCCACTTTGCTCTCAAGTGCCAACTTGGAATGTTTCATTGCAGGAAACATTGAAAGGACTGAAGCCGAATCAATGATCGATCACATTGAAGATAAGTTCTTCAAGGGGCCAAATCCAATATCACGACCGTTATATCCATCACAGTATCCAGCCAATAGAATTGTGAAGCTTGAGAGGGGCATTGGTTATTTCTATTCAGCAGAGGGTCTCAATTCAAATAACGAGAATTCCGCTCTTGTTCACTACATTCAGGTGCATCGAGATGAATTTCTACCGAATGTGAAGCTTCAGCTTTTTGCTCATGTGGCACAACAAGCAGCTTTTCATCAGCTTAGAACTGTTGAACAACTTGGCTATATTACTGCTCTAGTGCAAAGGAATCACCGTGGTGTACGTGGAGTTCAATTTATTATTCAATCCACTGTCAAGGGTCCTCGAGATATTGATTTGAGAGTAGAAGCGTTCCTGGAGATGTTTGAGAAAAAGCTCGTTGAGATGACCGTTGATGAATTCCAGAACAATGTAAATGCTTTAGTAGATGCGAAGCTTGAGAAATTCAAGAACTTGAAGGAAGAATCTAGATTTTATTGGGGAGAGATTTCTGGAGGGACACTGAAATTCGATAGGAGAGAATCAGAGGTTGCGATCTTAAGGACAGTCACACATCAAGATTTGATCAACTTCTTCAACGAACACATAAAAGTTGGTGCGCCTCGTAAAAAGTCTTTAAGTGTTCGTGTTTATGGAAATCTTCATTCTTCAAATTATTCTGGAGATTTAAATCAACCACTTGAACCCGACACCGTAAAAATAGATGACATTTTTAGCTTCAGAAGATCACGGTCACTTTATGGTTCCTTCAAATATGATAGTGTGAAGTTGTAGTGGCTCTGAACTCCCTTTTCTCATGCATATTTCCACACACAGCTCTTGAAAACTGAGGCTTTGAATGTTAAAATACTTGAAAAAATTTTGTTGAGTTGTGGAGCCATCTTATGTCTTGAATGAGATGAATCAAGCATTGATCTGCAGCTGGATTGGTTGTGAAGTGGAGAAGATGGGTTTGAATCCATTGGTTGCAGAAGTGTTCTCTTTTGTTATACAATAAAAGCTTTTGCAGATTTCCATGTGATAATACTTATAGTTCTCATTTGCTATAACACAATTTTGTTATTTATTTACTAACCCTACTTATGGGTGACATAATAATTATTTCCTTGTTTC

>cucumber_newGene_497 cucumber_newGene_497.1

CCAGAGTCTTTGAATTTTATATGCATAGAAAACAATATGGAGAAAATAATAATAAGAATCCCAATTTCTTAAACAGGATATTTTGCTAGCTGTTGTGTTGTTGTTGAGTTGTAAAACAGTGTGCAACTATTTAGAAGCAGACTTTATATCTCACTCAATCATCTACCTCATGATCAGAGTGTCCTTTTCCTGAAAGACTGCCAACCCATAAATTATTCCAATTTCTTCATATATATCTCTAATTGCTTCTTCACATCTCTATTATATTTTCACCCTCTAAGAAGAGAAAATAAACCATAATCTCTCTCTCTATCTCTATCTCTTTAAAGTTCCACTTCCAATGACTGAAGAAGAAGAAGAGTCTCTGAAAAGAAAAGTGAAACAAGTTGTTGAAGGAGACATAAATGAGGCTGAAATTTTGAGAAATGGAGGAGTCAATAAAGGCGCTTGGACTGCAGAAGAAGATCAAAAATTGGCTCAAGTTATTGCCATTCATGGTGCAAAAAGGTGGAAGTCCATTGCAGCTAAAGCAGGCCTCAATCGTTGTGGGAAGAGTTGCAGACTAAGATGGCTCAATTATCTCAGACCTAACATTAAGAGAGGAAATATATCAGATCAAGAAGAAGATTTGATCCTCAGGCTTCATAAACTTCTTGGCAACAGGTGGTCTTTGATTGCTGGGAGACTGCCAGGGCGAACAGATAACGAGATCAAGAACTACTGGAATTCTCATTTGAGCAAAAAAATGAACCAACAGAGTGAAAAAGAAAGAAGGAAAGGGTGTAAAAGAAAAGTGAACACAGAGTCTAGAGAAGAGGAAACGTCTCAAGAACGTGAAAACAGCTCAAACTTGGAGTATGATGTCGATAACTTGTTTGATTTTTCCAATGAGGGACCTGATTTGGAGTGGATGAGTAATTTCCTGTAATTGGGTATCATAAGCAGCAACTATAAAGCTTAGGTTGTTAGTGCACTCTCATGCAAATCATTACTAATTTATCTAAAAATTTAATCGTTTCACTCATAGCCTTTACCAAAATATTTGGTTGCTGATGTACAGATTCCTACTAACTTCATCTTCTGCAATCATCAACTATCAAAATCATAT

>cucumber_newGene_498 cucumber_newGene_498.1

GAAAAAGGAAATGTACCCTACGGTTCCTCGCACTTCTGTTCACACCCCACCCCACCTTCGCCACAGCGCACACCGCCGCTAACCCACAGGGAATTTTCTTTCTTCTCCCTCGATCTCCAGCGGCAGCTACACCAACTTTTCATTTTGCTGCACACTGTCACCAACCTCACTGCCACGCCCACCTTTTATTTCTAAGGGTTAGTGAGCTCGTTCTTTTGCTAATGGCGTCCAAACTTCTTATTCGTACGGGAAGTTCTTTGATGAATCGATTGCGATTAAGCTCAGCGCATCAGAATAACAGGATTTGGGGTAGCCAAGCTCTGTCCCATGGCCATGGCGTTGAAGTTACTCCGATGCTTTTTCCTTCAGTTTCTAAGCACCAGACTGCTCATCCTTTGCAGCAGAATGACACCGAATCGTTGAGGCAGCTCCAATCGGAAGGCATTTTCTTTCCTTTTGGGCTTCCTTCGCTCCGGTTCTTTTTGCCTGACGGAGATGCTTCTTCAACACAAGAACCGATGCTCTTATTTCCTAAAAGGACATTTCAACCTAGCACCATCAGGCGAAAAAGGAATCATGGTTTTTTTGCTCGCAAAGCAACCAAGGGAGGACGGAAAGTTATTGCTCGAAGGATTGCAAAGGGGCGCTCAAGAATAACTGCATAATGTTCATCTCAAGTCAGGTGCAATTGAATCGCCCTACCAATATCTTATTGTGTTAGTTTTTTTAAATTTAAAATTAACTCAATAGAATGGTATCGCTGGCTGAATAACCCCTTTTGTCTTTCTTCTTAGGCCTCGGAATACAGTCTGTATAAACTTCTCTGTTTTTAGAATTACCATGCATTAAACACCTTCCATATTGAAGTTTAAGTTACTCTTAATTTTTATTTCTTTCTTAACTTAGCTAGAGAATTGTTTACTTTTGGTTCATGGTGAGTGTTGATACTGGCTCCTGAGCAAATAAACAAACTCAAGTCCGAAAGTTG

>cucumber_newGene_499 cucumber_newGene_499.1

AAAACCAAGTTGAATGCGTTCTTCCGTTTCCATTTTCTATTTAAGGATTTCGACTTCTCTCTTCCTCACTGTCTTTCTCTTTCACAATTTCTCTTCTCCGCTGTCTTCTTCTTCTCCGGCTACAGAGTTCTTCGACTAGCGTTCCTCTTCTCCCTTCTTCCGCTAGGCCTTCCTCTTTCACTTTCATTCTCCGGCTAAAGGAACTATATCTAAATAATAAGTGAAAATAAAGTGAAAAGTCATGCCAAAAATGAAGCTTTGCAATATAGTGCTGGTGCATTTCTTCTTGGAAGATCTTGGCTGCCAAGGTGGGATTTACTACTTGGGATTTGAAGAACTGAAAAACTTGTGTTATTTGTTGATGAATAATGTGATTTAACAATGTCTTTGACTTGCTATTGGAGCAATGGTTTGAAGCTCTATTATCATTATCATCTGTATATGATATTGTGGTCCGATATTCATCATTATATATTCTATTTCTATTTTATATGATTTTGGTTTTTATTCGATTTCTTTTGTCATTGCAATAGATGAATTTGTTTGCCTTAAAAACATGATGCTATGATTCTA

>cucumber_newGene_501 cucumber_newGene_501.1

CTAAGATCAAGTGTAGTATCTGTTCTTATCAGTTTAATATCTGATACGTGGGCCATTGGCCCACATGATATTAAATTAATTTATTAAGGGGGAAGGCCACAGTGGTAGCTTGCTGCCACCGCCTTCGAGTGTCGCCCATGCGTTGCACTGCTGCTTGGGTTTGGCGCACTCCTCTAACGGGTATCAAATTCAACCAAAACGGACTACTAGAAGGCCTCAAATGGAATGTTTTTCGACACCACATGAAAACCGTGATGAGAAAGAAACAAAAGAAAAAGATATGGCAGCCCATATGGAGGTTCAAGAAGGCCTGACGTTAGCATGACATCAGCAAACGCTGGGCGTGGCTGACGGCTTAGAGAGGCGTGACATGACTCGACGACTCGGCTTCAGGTGGTTTGCACGTGGTCGACTCCATTTTAAGGTGGTCGGCTTTGATTTGGTGCGCTTTAGCTTCTGGCTTGCGGTTCGGGTTCGATTCCTCACTAGGTGCATGGTTTGATGCTGATTTAGTAGATTAAATTTAAAATAATTTAGTATATAGCCATATTATAAAAAACTTGCAAATATGGCATGGCAAATTCTATCAATGATAGAGTTTATCACTAATAGATTATGTTGAAAAATTGGTCTCAGTGATACAAG

>cucumber_newGene_502 cucumber_newGene_502.1

AATATAGTTAAAAGAATCTTCCCATAAGTTTAGCATGTTTTTTTGTTTTTGGAAGATCCGATTGTTTCTTTTCATCCAAGAGCTCCAAAGAATAGCAACAACAACATTAAAAAGTATGATATTACGTTTGCTATTCTGGCCAAGTTGACAAATTTCAAGGCAGAGAGATTTAGCCTCAACAGCTCTGAAGGAGTGGTTTGTATAATGGAGTAAGCGGTTCCATAAGAAGACAACTAATCTGCATTATAATAGAAGGTGGTTTATCTCTTCATTACTTTAGTTACAAAGAGCACACCAATACTTCCAAAGGTTTTCGAAGTGAAGGTGTTCAAAGCCTACGATATTTGATTAATCAGCAACAGCTAAAGCTTCCTTGACCGAGGCAATGAAGTATTGTTTGTCTTTGCTTAGGCTCCAAAGAGGCTTACTGATGCCCTTGTTTTCACATGGAACAGAGAGACTACTTATCATCTGATTTCATGTTTCAATTTCTCTATCATTCAAAGGCCTTTCGGTGTTGATGTTCCACTGTTTGTTTGAGTTATCTCAAACTTCTTTAATCAAATCATTTTTCAATTGACAAAGGACCTAAAAGTCTGGGAAACTGGATGGACAGAGGATCAAAGGAGTGCCAATTACTATGCCAGAAATAGATAATTAAACTCACCATCGTTGATGTCCCATTTGTAATGGTTTTTGAACCCACTAAGGCCTTTAACAATAGCTTTTCGCGGAGCTTTTATACTGCAGGATTTACTTTTAGTGGGGGATATATCCAATATGCTTGCTGTGATACTTTGCTTGAATAACTTTTCTCCAAAGGGCGTTCGGTTCCATTTGAAATCTTCATAACCATTTGCAGAGAAGAGCAAATTAAATTGGTTATTTTCATACTTGTGATACTTAAGCCACCTTTATCTTTTGGATTGTAACTTTTGACCACTTAATTAGATGTGAACCCATGTTGTTTGTG

>cucumber_newGene_504 cucumber_newGene_504.1

TCTCTCTCTCTCTCTTTCAAACTGTGTTCATCTCTTTTTTAACTCTCTCCAACCCTCTTCTTCTCTTTCTCTGCACTTGTTGAACCTGAGAAGCTTAGCCCAGACAAGCTTCGAACTGAAGGCAATGAGCCAGCTTCAGAAGAGCCTGTCATTGGCTTCTTCTTCCAGTAACGCCATTTCTTCCTCTGTATTTTCAACAGCGTTCTCTGCTTTTTCCTCCTCCAGACTCCGTCATTTCACCACCAAGTTGCAGGGCAGCACTCTTCAAAATTATACTAGCAAATACACACCCGTTTTACCATTTTCCACTGCTTGTAATTCAGCCAGATGTTCTCAGCTTGTTTTTAGAAAAGGTGGAGTAACATTTGCTGTCACAACTGAACGTACAAAGGCTGGGCAGTATCGTGCATGCGATGGGCCAAGTGTTTTCACTCTATATTCCCGCTTACCATTGAGATTTCCTCATTCTGCTGCATTACCAAATGCTTCTAACCAACAGCTGTTAAATGGCATTTTTGCACATAAACTTCCTAATTATAGAGCGTTTGGAAGGAAGTTCTTTTCTAACACTACAGAAGCATTGAATGGAACTAAGAAAAAGAATGTTAAATATGCAAGGAAGGGAACAAGTACATTCCCAATTGAAGGATCAAATAAAAAGTTGAAGGGTGAAAAGCCTGTTGCTACATCTAGGAAAAAAAGAATTAGTGCTTCAAAGGCCTCAGGCTCCACCTATGTTGGTGAAACAAATTCAAGCGATCAGTTGGTTGGTGCTATGAAGGAGGCAAATCTAGTTTCTTCTTCTTCATCCACTAGGAAAGCATCCAAGGGTTTGAATGAAAAGAAGTCTCGGAGCAAGAAAAAGAAGGAGGCTATTTCTTCAACCAATGATGCAGATGTTAAAGCCGTATGCAAGGATGGTCAGTCAAAGAGCTTTGGTAGCAGCAATACTGACCAGGATGTTGTACAAGATCCAAAGAAAAATGTAACCGGGGGTACTTCCAAAGATTCTGCTTCAACCAAAAAAAAATCCAATAAGCAAAAACGCAGTTCCAGCAGAAAGAAAGAAGCTACGAAAAGCGCTAATAAGTTGCGACAGAAGCCACACGTCGTAGTGGATGCAAGGGGGACATCTCAGGTAAGGACCACTTTCAAGCAGCTATATCCCCCAATGGGAAAATCTGTTGTGATTGTGGAATCTGTTGCAAAGGCAAAAGTTATTCAGAATTATCTTGGTGATATGTTTGTGGTGCTACCTAGCCATGGCCATGTTAGAGACTTGGCTGCAAGGTCTGGATCTGTAAGACCTGATGACGACTTCATTATGGTGTGGGAGGTTCCTTCTGCTGCCTGGACTCATCTCAAAAGCATTGAACTTTCCTTAAATGGAGCAGAGAACCTTATTCTTGCATCTGATCCAGATCAAGAAGGTGAAGCTATTGCTTGGCATATAATTGAGATGTTGCAGCACCAGAATTCTCTACATGAAGGTATTAGCATAGCAAGGGTTGTTTTTCATGAAATAACCGAGGCTTCTATCAAGAGTGCCCTGCAATCTCCTAGAGTAATTGATGAAAACTTGGTTCAGGCTTACCTTGCACGCCGTGCTTTAGATTATTTGATTGGATTTAATAGTTCACCATTATTATGGAAGAAATTACCAGGTTGCCGATCACCTGGGCGTGTTCAATCAGCTGCACTCGCCCTAATATGTGATAGAGAAACGGAAATTGATGAATTTCATGCACAAGAATATTGGACTATTGACATTAAGTTGAACCAAAAAAATCCTTGTTCTTCAGTGGGAGATTTTGCAGTCCCTGCACATTTGACCCATTTTGATTTTAAGAAGTTAAATCAGCTTGCAATCAGCTCCAACATGGAGGCAAAGAACATAGAAACTGCGCTGAAATCAGTAAATTTTCAAGTGCTTAGCTCTAAAAAGAGTATAGTGCAGACAAACCCTCCAATGCCATATATAACATCTACACTTCAGCAAGATGCAGCAAACAAATTTAATTTCCCTGCAAGTTACACAATGCAGCTTGCCCAGAAACTTTATGAGGGAATTCAATTGGCTGATGGAAAAGCAGCTGGTCTAATAACTTATCCAAGAACGGATGGGCTGCATATCTCTGATGAAGCCGTTAAGGACATTCACTCCCTGATTATGCAAAGATATGGGCAGGATTTTGTATCCAAAAGTGGACACAAATATTTTAAGAAGGTGAATAAAGCTCAAGAGGCTGACGAAGCAATCAGACCCACTGATGTGCAACTCTTGCCCTCAATGCTAGTGGGAATACTTGACGAAGATTCTCACAAACTATACTCTCTTATTTGGCTCAGAACGATGGCATGTCAAATGGAACCTTCTATCAGTGAACAGATTGAAATTGACTGTGGACTTACCGATGAATCCATCGTTTCCGGATCTACATGCTCAAGAGTTCAATTTCGTGGGTTTCAAGATATTTTTGAGGATCCAGAAGTTCATGCAGTCAAACATGAAAACCATGAAGAAAGTGGACAAGATGAACTGTTCAGAATTCTGAATGCATTGAAGCCAGGGGATCAATTGTCTCTACTTGCTGTTGAACTCAAGCAACATTTTACCCAGCCCCCACCCCGCTATTCAGAGGGAACATTGGTTAAAAGGATGGAGGAGCTAGGGATTGGAAGACCTTCCACATAGGCAACCACAATAAAAGTTTTACAGGACAGGAACTACGTGTCAGTGAAAAGTTGTGTTCTACATCCGGAGTTTCGTGGGCGCATAGTATCGGCATTTCTTTGTCATCATTTCTCTGAGGTCAGCAATTGTAGTTCTACTGCAGACATTGAAAATAAGCTTGATAATGTCTCAGCTGGTTTAACTGAATGGAAAGGCTTCCTAAGAGATTGTTGGACCCAGATCAGCTCATATTGTAAACGTGCTAATAATGTTAATGTTAATCAGGTGGAAAAGATGTTGGAGAAGAAATATGGGGACTTCTTATTTTCTTTTCTTCCTGATAATTCCAGAGCATGTCCAAGTTGTCCGGATGGCACTCTGTGCTTCAAAGTTAGTCGGTTTGGTGTTGGCTATTACATAGGTTGTGATCAACACCCATCATGCAAGTATGTTGCAAAAACTTTATTTGGTGAGGATGAAGATAAAGATAAAGATCCTTCTGAAATTGACAATGGGGGGGTAGAGCCAAAGGTTCTTGGTCTTCATCCAGTTTTGAAGGAAAAGGTTCTTTTGAAGACTGGTCCATTTGGGTGTTATATACAACTTGGTGAGGACAGGAAAGGGTACATGCCCAAAAGAGCCTCTGTTTTTGAGATAAAGGACGTGAACTCCATTACTCTCGATTTTGCCATTGATCGTTTACGCTACCCAATTACTTTGGGAAAACATCCAAAGGATGGCCAACCAGTGATAATAAAGATCGCAAGAACTGGATTCACAATTAGACATGGACGCACGATAGCTTCCATTCCTAAGAATTTGAAGCCTAATAATGTCGATTTAGCGAAAGCATTGAAGCTTCTGTCAAGTAGCGGTGTTAGAAGGGTCGGGCGACCTAAAGGCCTCCTTCCCAAGGTTGAGGATGACTTCTATTGAGCTTCTCAAAATAGGTGATTTATGTGTTGCCCTTTGGCTTGTTTATTCATTTGGATTTTAACTTTGATTATAAATTTAGCTTTTCTTTTTAAGGTTTTCTTCTCCTCGCTTTATCCTTTATTTTGTATTCTTTTCCAATGTGTGCATTCGTGTAAGGAGATTGGTTCGATATTCATAGTGGAAAATTAGGACATAGAATTAGTGTTTATTATTTCAG

>cucumber_newGene_506 cucumber_newGene_506.1

CTAGGGTTTTTCTGGTTTCCCAACTTCATCAAATCTTCTTCCGGAGATCTTGTGGTCAGTCAAGCAGCAATGACGAAGAGAACCAAGAAGGCAGGCATTGTTGGAAAATATGGTACCCGATATGGTGCCAGTCTGAGAAAACAGATCAAGAAGATGGAAGTCAGTCAGCACAGCAAATACTTCTGTGAGTTCTGTGGAAAGTACGCAGTCAAGAGGAAGGCAGTTGGAATTTGGGGTTGCAAAGATTGTGGTAAAGTGAAAGCTGGTGGTGCCTACACTCTCAACACTGCTAGTGCTGTGACAGTGAGAAGCACTATTAGGAGGCTGAGAGAGCAGACTGAAAGTTAAGCCATCTTCACTGCGTATTTTCGCCCTCTTTGTATGGAAAAAGATTGCTCCCTCACTACACTTTTCTTCGTTTCAATGTTGTAGGAATTGGTTATTTGAAGTTAATTGAAACTGATCCCTTTCTTCATTTCTTATACTTTTGGCCATTTTTCTATTGATCGTCAATGGAGTTTTGTTCAAGTTTACATGTTTGGTAGCTAAGGTTGACATCCATGAAATGAGTCCTCTGCTTTGTTCACAAAACTTTTTCCGATTACTAGGAG

>cucumber_newGene_507 cucumber_newGene_507.1

GAAAAACCGTCATTTCATTCATGTCTGTCTCTTCACTCCACTGCCTGGTTCCGTCCCCCATCTCCAAACTCTTTGATGGTCATCTCCGGACGACGTCCAATTCCATCACAACCACTGCCCCCTCGTGTCCGTCTCCTCACTCTAGGGCCGTCGAAACCGCTTCTACTGGATGATGAAACGTCCTCTGTCCTAATTTGTTCAATTCGTGCTCATAATCTCCAATCCGCCGCATTCTCTTCAATATTCAATCCTCAGCCGTCACCTCCTCTACCCGCAAAGAAAGTTCTTGGAATTTGAATAGTTCGAGATAGAGCATCCAAGAAGTTATACATGTCACAGGAGCAATACATAGAGAAAGTACTTGAACATTTCAAGATGAATCAAGCAAAACCAGTTAGTTCCCCTTTACCCAGTCACTTCAAACTGATCAATAAACAAAGCCCTTCTACAGATAAAGAGAAGGAGGATATGAGTAAGGTCCCATATGCTTCAGCAGTTGGAAGCCTAATGTATGCCATGGTATGTATTAGACCTGATATTGCTCATGTTGGTGGTGTTGTTAGTCGTTTTATGTCTAATCCAGGAAAACAACATTGGGAGGCAAAGTGCATCATGAGATATTTGAGAGGTACTTTCAGTTTGAAGCTCACATTTGGGGATGGAAAGCCAGTACTTGCTGGGTATACTGATTCAGATATGGCAAGGGACTTAGGCAGCAAAAAGTCTACTTCTGGTTACTTGATGATATTTTCAGGTGGTGCAGTGTCTTGGCAGTCAAGGTTGCAGAAATGTGTTTCCCTTTCTACAACTAAAGCAAAGTATATTGCAGCAGCAAAAGCATGTAAAGATATGTTGTGGATGAAGCGCTTTGTACAAGAGCTTGTCTTCAAGTAACAACGATATGTGATATATTGTGACAATCTGCTATTCACCTTGGTAATAATATGCTTCATTTCATTCAAGAACAAGGCATATTGATGTGAGATATCACTGGCTTAGAGATGCTTTAAATGATGA

>cucumber_newGene_508 cucumber_newGene_508.1

CTTCGGGTGGTTTTCCCAAGAGTCTTTAAAGCTATAACATGGTTTAGGGTAAGATAACTTAGTTAGGTTTCTAAAGAATGTTTAAGGGATTGATTTATTCTACCACAAATCATTATCAGACATACATTTATAATATGTTTCTAGAGAATTTAACAAAGAAGACACATGTGAAAGTTCAGAATTATCCCACTTGCCAATCACAAGAAATGAAACTTAAAGAATGCATAATTTCTACCTTTTGTATTGTTGGTAGCAAAAGAGAAACTCATGTGAGCTGAGCTGAGCAAACCAAAGCAAAGTCATCCTGAAAAAATAAAAACACCCGTGACTCTTCTCATTTTACAAATACAGACAAATTCCTGAGATCCTATATTCAATTCTGTATTTCCGCCTCTTATTTTGGCATCCAAAGTTTCTCCCCCTTCTTTTTTTTCCACGTAAATTACGCCTTACTTTTCCTAATATTTCAATACTAAACAATAACCTCAAAAATTCAATTCAATACTTAAGTATTTTCTTCTTTCTCTCTGACTTTCTCATTCCTTTATCCAATCCAATCCCAACGGTAAAGTTGGTTTGTCTTTACCTCACCTATTAACTTCCAATTTGGCGCAGTTGATCCCGTGAACAATTTCTCTCTGAACCAACATTAAACTAAACTTCTTTCTCTATAAATAACACCCCACTTCACCTCTATTTCATTCATCATTCCAAGTGAGTGTGTTATATTACAACTTATAACGATATTACAATGTCTTTCTCAATCACTTTGGCTCTCTACTTCATTTTGTCTCTCTTTACCTTATCTTCCTCTGCTTTCACTTCTCAACATTATTCTACTGCTCTTCAGTATTCTATTCTTTTCTTTGAGGGACAGCGATCCGGAAAGCTGCCCTCTAACCAACGTCTCACATGGAGAGCTGATTCAGCCTTATCAGATGGCTCCTCCTATCATGTTGACCTTGTTGGTGGCTACTATGATGCTGGGGATAATGTCAAGTTTGGCTTGCCAATGGCCTTTACTACTACATTGCTGGCTTGGAGTGTCATTGAGTTTGGCGACTCGATGGGGAATGAGATTGAGAATGCAAGAGCAGCAGTTCGTTGGGGGTCGGATTATCTATTGAAAGCTGCTACTGCTGCACCTGATGTCTTATATGTTCAAGTGGGAGATCCAAACCTAGATCATAAATGTTGGGAAAGGCCAGAAGACATGGACACGCCACGTACTGTGTATAAGATAACTGCTCAAAACCCAGGCTCTGATGTAGCAGCAGAGACCGCAGCTGCGTTGGCTGCAGCTTCAATCGTGTTCAAAGCATCCGACCCTTCTTATTCTAACAAATTACTGGACGCAGCCTTAAAAGTATTCGATTTAGCAGACAAGCATAGAGGTTCTTACAGTGATTCACTCCATTCAGTGGTCTGTCCATTTTACTGTTCTTACTCGGGATACAATGATGAGCTTCTATGGGCTGCCTCGTGGGTTTACAAAGCCTCAAAAAACAGCATTCATTTAAGCTATATACAGTCCAATGGCCATATACTAGGAGCCGAAGAAGACGACTACACTTTTAGCTGGGACGACAAACGCCCTGGAACCAAGATCCTTCTCTCCCAGGATTTCTTAGTGCAAAGTTCGGAGGAGTTCCAAATCTATAAAGCACACTCAGATAATTACATATGCTCCCTCATTCCAGGAACTTCCACTTCTAGTGGTCAATATACTCCTGGAGGACTATTTTTCAAAGGAAGCGAGAGCAACCTGCAATATGTAACTTCAGCAGCGTTTCTTCTTCTGACATACGCAAAATACCTAAGCTCCAATGGGGGATCCATTCGATGTGGGACTTCAAGGATTTCACCAGAAGACCTAATAGCACAAGCAAAGAAACAAGTTGATTACATATTGGGAGAAAATCCAGAGAAAATGTCATACATGGTGGGATTTGGAGAACGATACCCTCAGCATATTCATCACAGAGGTTCCTCTGTACCGTCCCTTCATTCACACCCTAATCGAGTTTCTTGCAATGATGGTTTCCAGTTCCTGTACTCTTCTTCGCCAAACCCAAATCTGCTCCTTGGTGCCATTGTTGGTGGACCTGATAATGGCGATAAATTTTCCGACGATCGGAATAACTATCAGCAGTCGGAGCCAGCTACTTATATAAACGCTCCACTTGTTGGTGCCTTAGCCTTTTTTGCAAAAACAACTTAGTAGATAGTTACATTTTAGTTAAGAAGGAAGGAGAGATGATTGGAATTTCGTCCTATAAGATTGTGTTTAGGGTTTAGCGTCTAGGGTTTAGCTCGGGTCAGAAG

>cucumber_newGene_509 cucumber_newGene_509.1

GAAATTAACGGTAAAATAATACCAAAGGGATGGAAAGTCATACCTTGGTTTCGAGGGCTTAACATGGACCAAAAACTACATCCTTCCCCTCAACAATTCAATCCTTCCAGATGGGATAATTTTGGAAGAAATTCTGGAGTTTTTACTCCCTTTGGACTAGGAGTTTGGATGTGCCCGGGACGTGATCTTGCTAGAATGGAGATCTCAATTTTCCTTCATTATTTTGTCCTCAATTACAAGATAGAGCGACTTAATCCTAAATGCAAATTGAATTACTTGCCCATTCCTCGTCCGAGAGACAAATGCTTGGCAAGAGTGATAAAAACTCCATCAAAGTGACTAAAAAGTCATCCCTCATGTAACCTTCTTTGGTCCAACAAGTGTGAACTTTGTGATCATTATTGTATCCAACCTTCTACGTATATATGACATGTACTTGTGTGTGTGAATTTTAGTTCACCCATCAAATAGAATAAATATTTATTATTGTTATGATAAACATTTTTATTTTTC

>cucumber_newGene_510 cucumber_newGene_510.1

GAAATATATTGATCAGCGAATGCATAAAGCTATTGTGGTTAAGTAGAGAAAAATCATTTTTTCAATGGAATTGATGATAAATTGGTTGTTGTTGATTGTTCCTTTCTTGGGTTTTGTTTTAGGGTTTGGGGTTTTGAAGAGATTGAATAATTTGTATTATGCACTGAAATTAGGGAAGAAATGGGATGAACTTCCTCCTGGTGATCTATCTTGGCCACTTATTGGTTCTACTCTATCCTTTCTCAAATATTTCACTTTTGGTCCACCAGAACGTTTCATTGGTGACTTCTCAAGAAGATATGGGAAACTTGATATGTACAAGACTCACATATTTGGAAAACCAACT

>cucumber_newGene_512 cucumber_newGene_512.1

AAAAAAAAAAAACAAGTGGAAGAGAAAAAAAAAATTCGACTGAGAGACGAATGGATGATGCTCTAACCCTAAAAATGAAGATATATATATATAATCAATGAATGCTACAATGCTTGGCACTTCTTGTTGAAGCAATGAGCATCGATGCATATTGGGTATAGGCACCTGGCGAGCGCAACAACGCTTCCTTTATGCTCCAAAGACATTTTTGTAACTCGATATGATTCTATCTCAAACTGGTATGATTTAGCACCGTTTCGTTCCAAATGTTTCATTCTACTCATAAACACTAATTAACTTTAAATTAGACAATTAAGCCACATTAAACAAGGGAACGACCTAGAATTAAGAAGAGTAAAATAGCACATTTCGAGTGCTATCACCAAGTGGACAAGAAAATGTGCCATCCAAATCTGAAATTTTGAGTTATATTAGGGTTAGAATTCCCCAATGGCGGAACTGAGTCGTCTACATAAAAATCTTATTATTCTTTCATTTCTTCATTAGCTTAGCAGCGGTGGTTCATCGGCTGCGCGCGGAGTGTGTTCGATCGGATTTACATCTCCCTTCCACAGGCTGTGACGAAGAATCCATCGCCATGTGATGTTGAAGCTCTCAAGAAATGTTTGCAGGAAAATAATGGCGATCGTGTCAAGTGCGAGTCTCAAATACAAGCCTTCATGTTTTCTTGTTCCCTAAAGAAATCAAATCCATCTTTGCAATCCGGGAGAAATGACTTCGACACCGGTGTTTGATTTCTATCGGCCTTCTGTTCTTTGTTCAAATTTCAAAAACCCCTTCTTTTATTTGAGTGCAGCAATGAAGAATTTTGCTTACAGATGATTGTAATTGTGCTTTCTTGGCAATAAAAATCTCAATCTTCCATTGCTAATCCTTCAACTTCATTCTGAATTTCCGTTTTGCTTTATGAAATTTCATAGTCTGAATCAAATCATTGTTCTGCTTGATATTATTAGTATTGTCTACCTACTCTCGACTTGAGATCATTACTTCTTTAATGGAAATAATATAGAACAAACAACATTAACAAAAACCAAATGCTTCATCATAGCTGCAAAAATTTCATTAAAATGGAAAGTTTATTGATAGAATTATTTACATTAGAATTTAATGCTCATAACAAACAGTAGTCAAAATCGACACTAATAATACAAATCAAGGAAAAGTATGTATTGTGAATTTGTAGAAAATTTTGCCCTTTTACTCACTTTAACCAAATGAAGTGAGTTTAAAATCAGCCTGGTGGTTGAGAAAGTACAACACAATATTTGATGAAAGGAACAATCCAATCTGAAGTTTCCCTCAGTGATTAGTAAAATATGGAATGATCAATCCCTTTTCAAGAGAAATTTAATGGAGTAATCATGAGAATTTGTTTGATTAGGGAAAAATTAAGGACTTGTGATAGCTTGAATTAACTTAATCACAAGGCAAGCATGCAACAGAAGGATTAAAAAAAAGGTTTCTGCATTTTCACTCCAAAAAAGACCAACCAAACCAACAGAAGAGAGAGAAGGAGAAAGGGGAAAAGTCCCTTTCTATTTAGAAACTATGTTCTTTCAGAGTTCAAACTCTTTTGAGACTAACATTTGTAAACTTCATGTGAAGGCAAAGCCAAGCCAAACACCAACCCC

>cucumber_newGene_513 cucumber_newGene_513.1

GGTTATATCAATGTTAAAAGAGACTTACAACAAGAGATATGAGTAGATGCTCCAGAATCTATTATCCATAAAGGAGATGAATAAGAAGTACGTGCTATGTGTGAAGTAGTTTGCTAATTGGAAGCATTTATTTGGGACTGAAGTTGATTAAGAAGATTTTGACACTGTATCAAGGCTTCTACAATGTTGTTAATCTTCGAAATTGAACTGAGTTCCGTGGTTCTTTGAGATGTTGAGGAAGCAAATGATCCGATTGACCTTTGTTGTTCTTCTTGAAGGAGAAGAGAAAAGGCCCGGCTTATAAATGGGAGAGGATCCATAAGCAGGAGTTGAGCTTGTGCATGAGAGAAATTCTGATTGAGCCCCATAAGGACCATAAGATATTCCATCTAAAGGAAGTCTGCCATTTCTTTTAGGCCATCACAAGTGCAAGTTCCATAGTTGCAAGCTGATCTGTATGTATTTAACTCATCAATAAAAGTCTTGAACTTAGTAAAATACATACAAATAGAGTCTCGGTTATGTGATAGTGTTGCTAGGGATCATTTCAATTGAAATATCCTTGGAGCATTCTTCTTTTGAAATCATTCTTTTTCCTCAACTGCGTAGAATTGGAAGAATTTGACATTTAGTTTTTGTGTGTTCTATAGGAATCGAGAAAGTTATGAGAGGA

>cucumber_newGene_514 cucumber_newGene_514.2

AAACGCTCAACTCTTTCTCACTGTCTTAATTATCAATTCTTTTTTTCTCCTATCTCTTTCTCATCCCCCTTGAAGAAAGGCGAAACTCTAGGGGGGGGGGGGATAGAAGATAAGGAAATGACCCAAAGCCAAACATGGTTTTAGGGTTTTTAGGGTTTCTTCTGGAGTTAATTTGAAGATCTGAAGAAAACTAGAAGCTTTTTTTTCTCTCATATTTTCTTTTGAGGGGAATGTTGTCTGGCTCGAGGACACTTGTTCTTTTTCATCCATTTCTTGAATTCATTAGTGTCGTCGGACCAGGCTTCATTCCCCCCAATTGCCTCCTCCTTAGTTTCCCCCATTCAAAAACACACACAGAAAGCCTCCGAAGACATTTTCTTGTAATTAAATGAGCTCGCTTTCAACGACGACGACAATGACGATGCCCCGGATGGTAAGTACTCTTCATAAATATTTGATTTTTAGCATTTGTTTGTGGTTGGAATCTGTGGAATATGAACATGAAGCTTGATGAAGAGGAACATAGTTTGAATTTTAAATATGGGGTTCTTTAGTTAATTATTTAAAGGTTGAATTTTATTAATTTTTAGTGGGTAAGAGAGAGACGTTTGTTATTGACTTTGGAATGTGATTGTGTTGTGTTTGTTTTTAGCAGTGTTTGGATATTGAAAATTGTGAGATTGTTCATTGAAAAGGTAAAAAGTCAAGTTTTGAAGTGTAAGACTATGTGCCTATAGGTTGCCTTCTCCAACAAAAAGTTTGCAGTTTTCCCCATTTGAGGAGGGCTACCACTGTCCTTCCAATTTTGTGAATCCAATAAAG

>cucumber_newGene_515 cucumber_newGene_515.4

ATCGGAGAAAGAAAATGTTGATGAAAGCCATGGGCGTCACTGCGAAGTGCGAAGTTGCCGCCGATTATGGCTCATGGCCGCTCATCACCATTCGAGCACCGTGACCCTCAGCCGTCAATGCGTTGCTCCTCGGCTCCCTCAAAGCACACTATCTCGAGATATATCTAAAATGTCGTTGTCTCCGGTGCACTAGATGGTAGTGGGCCGGTGCTTACTAGGAACTACGGTGAAGAAGCAGTTAAGGTTTAGGAAATGCCATTTGCCATTGTTATTCTGGGAGGTGGAGGAGACGATGAAATTAGTTGGTAATGTACATTTTCTTTGTGTGTTATATCCCGATGCATTACGTATCCATTCCGTTTCCATGAGAATCTAAGTCGAACCCTAGGGGTTTCACGTTGTTCTGCTCGGATTTAATGGTTTCGTGTTCAACTTAAACTGAACAAGAAAAACCCAACCCATTTGGTAATGTACTTTTCTGAGTTGTTGGTTTGGTTGGCAGAGATTTAGTTGAGAAAATGCGAGACATTTAAAAGAGTCTCTCTTTCCATTCTTCAAGCACGCCATGATGTGAAAGAAGACCAAAATTTTATTTGGTCGTTCACTGTTCAGACCAGTTGAATCATTTATCAAGGAGCACAAGAACACTCCACATAATCGTCCTAATCAGTTAGGTGTAGCAGGTAAAGAAAACAAGTGGAAATAAGGGATGAAAACAGGTGCATATTTCTTAAATAAGCTTATGGAGGCCGTTCCAGAGCCATGTTCAACTGGGTTCACACGAAGTTCCACTACAACCCTCTTAAAGATGGAAATACGTCAAGAAAGAAGGGCGAATCTATCACGAAGGAGGCAAAAACACAAGGATTATTAGCAGAACAAGTCGATGAATTTGTTGATGATTGGAGGATCAACAATGGCCGCCGTCTTACCATTTTCTTTATGGGATTTGAGAAGTCATCAAAAACCAAAACTTATTTCAATTTGAGGAAGGAGGGAAAGAGAGATAATGGAGATATTATTATTGTTGATAATGCTGAGGACGAGGATGATGATGAGTTGATCCCATTAATGTCGACCACATTTCAGAACAACTTTGACGATAGTAATGGTGAATTTTGTACGGAATTCGAGAAGTCTCAAGTAGTCTTGGCTATTCAACGACATGTCGCTCAAAGAATCCGCCAGAGAGTCACGCTGGCGGACCTGCTTTGTTGGTTTTAAAACAAATGATCCGTAAAATCTCACGCACTCTTCTTGAAGAAAACAAAGATAAATACTTTGTAAGATATATTGCCAAGAGGGAGAGAATGTAACAACAAGTGAAAACTACATAAGTTGCAGAAGAAAAAAT

>cucumber_newGene_516 cucumber_newGene_516.1

AAAATGTCAGGTTGCTACAGAGTTTGTGTTACCGGAGGGTCTGGCTATGTAGCTGCTTCACTCGTTAAAACCCTTCTTCAAAACGGCCATATCGACCATGCAACTCTGCGAAATTTGGATGATGAATCAAAGGTTGGGATTTTAAAGAGCCTCCCAAATGCAACCACCAATTTAGTGTTGTTTGAAGCCGATATTTACAAACCCCATCAATTTGAAGCTGCCATTACAGGCACTCACTTTGTTTTCCATCTCGCTACTCCTATGCACCACATTCAAGGATCTCAGTTTAGGAACACCACTGAAGCATCAGTTACAACGACGAAGATGATAACCAAATTTTGCGTGGAATCGGGAACGGTGAGGCGGTTGATCTACACAGCCTCCATCGTGTCAATGTCGCCGATGAAAGACGACGGGAGTGGTTTCAAGGAGTTCTTTGATGAAAGCTGTTGGACCCCTCTTAATCTCTCCTATCCTTTTTCTGACTCACTTATATTGGAATACGTGGAATCAAAAACGGTAACAGAAAAAGAGTTGCTTAAGTTTAGGGAGAGCGAGGAATCAGAAAGGTTGGAAGTGGTTTCATTGGCGTGCGGCCTTGTCGTTGGGGAGTCTCCTCATCCTTCTTCTGCTCTAAGCACCATGAGCATCACACCTTCTCCTGCTCTAAGCACCTATATCACATTCTCTCAGTTCATCGACGAAAGTGAACTCTTTAAATACTTTAGATCTCTTGAAGAATTGAATGGTAAAGTTCCACTCGTACATATTAATGATGTTTGTGATGCCCACATTTTCTGTATGGAACAAAGCTCAATCGATGGCAGATTCTTGTGTGCTAGTTCCTTCTTGTCTTCTTCGGATACTGCCAATTACTACCATCTTCACCATCCTCAGTTAAAACAAAAGCACGGGGTATCAGAGGAAGTTCCCCACAGAAACATCAATATGAACTCCAATAAGCTTATTGAGAGAGGTTTCATATATAAATACGATGGTGATATGATACTTGAGGATGCTTTCCGTTGCTGCAAAAACCAAATTTCCTAGATTAATTATATATATATATTGTTCTAATGAAGCTGCTCTATAAGGTAGCCGTGTGGTTCTAATTTGTTTGGTGTACGTCCACGCTCAACCAATAATGGGTTGGACCATCATATGGCTAAGGCAATCCTCCTGCAATGCAAATAACTGGAGGTTGTTTTATTCCGATTTCTTCTTCTATCTTCAAAGGGATGGGTGAGATTTCTTCTCTTATCCCAAACAGTTAAAAAAAAACTATATTGTTGGCACGGTGCTTCATATTAATGTAATTCTTTGTTAAAGGAATTGAAAATGAAAAATAACCAATAACCTAAAACAACAAGAATCAACACATAAATAAATTAATGTAATTCGATAAAAAAAAATATGTATTCAGTGAGATTTGAACGTTATTAAGAAGTAAAAGTTTGAGAATAAATGATTGCAATAATTGATGAGATTAGAAGGAAGAAGTGGAAAAAACAAAAAGATCAACCGACATGATGGGTAGGCCAAGGTTTAGGTGGCTCAGTTTCATTTACTGTCCTTTTGAAATAAAGATCGAGAACAAAGAGCCCATTATCAAAGGTTCTTTACCTTTTTCATTATTCTCTCTGATTGCTTCATTTCCCTAAATTACAATACTCATCTTTCTTCTTTATTATTATTATTAAAGATCCACATGTGAATTGACCTTTTTTTTCTATGGAGATTGCTGAATGATGCATCAAACGGAGAAGGATGAGGAAACTCATTTTCACCAATACCGTCTAAATCAAAGCAGACAACCAGTGGTTTCAAGGAGTTAATGGATGAATCCTACTAGACGCCTCTCTCAATACTTCTGTCCTTTTTCTATTTCTTTTTCTCCCATTCCAACTCCATTTTCAC

>cucumber_newGene_517 cucumber_newGene_517.2

AATAAACTCAACGAAGAACGGTCAGTTCATCAAAAGGCTAAAGCATTGTGGAGGTGGCCGTTCTCAGCGGGAAAGGCGAGAGCTGGGTTTGAGAAAATGGAGGGAAAATTCAAGTCTCTCTCATTGGTGGGAGCTGGAGCTCTCTTCGGTTCTGTTTCTACCTTCTTCATCCTCAAGCTTCTTCACAGACAGATCGCGGATCATGCTGAATCGAAGGCAACCAAGCTGCGTGGTAATGAGAATATGGTCGAGACTACTGTAAAGGGCAGTCCTGTTGTTGGGTGCACCAATCGTGGACTTTCTGATGTAGACCTTCTAACTGACGAAATTGTTTCTGAACAACTAACAAGGAACATTCAGTTCTTTGGCCTTAGCTCGCAGAAGAATGTGACTGCATCCTATGTTGTGGTAATTGGTCTTGGTGGTGTTGGGAGTCATGCTGCTATGATGCTTCTTCGATCAGGGGTTGGCAGGCTTCTCCTCGTTGACTTTGACCAGGTGTCACTTTCTTCTCTTAATCGACACGCGGTTGCAACAAGAGCAGATGTAGGTATCTCAAAAGCTCAGTGCCTGAAGGAGCACTTTCTATCCATCTTTCCAGAGTGTCAAGTAGAAGCAAAAGTGCTATTATATGATGCAACATCTGAAGAAGAAATTCTTTCCGGAAAACCTGATTTTGTTCTGGACTGCATTGACAATATTCATACAAAGGTATCACTTCTTGCTGCATGTGTACGTAGGGGATTAAAAGTATTATCAGCAACAGGAGCCGGAGCACGAGCTGACCCAACAAGAATTCGTGTGGCTGATTTAAGAGAGTCAACAATTGATCCATTATCGCGTTCTGTAATGTTTCTTACATGTGACAAGAGTTCTTTTTAAGCTAACGTTTTTTATATGCTGTCAGATTCCTTTGTTAGAAGGTTGGATCAGAGAAGTTATTGGTTTCTGGAACATGTTTTAAATTGAACTCTTAATTTAGAGGATGGGTTGTCATATATTTTGGTTCGTTTGAACTCCTAAACCACTTTTTCTTTATTTTAATATTTGGGAATTGATTAGAACTAGCAGGTAAGAGAGTGACTTAGTTAGTGAAATAGAGATTAACCTTTGCTAGTGAAACACTTAAAAGCATTTGGAAAGATCGTTTTTGTGATAAGCAGATAACTACTCATCTTGTGTTGGGTTATTAATATGCCATTTTACCATATGCCTATCAATTATATAGGAATGCACCAACATTTTAATCCTCCTTTTTGAAAATCTAGGATGTGGCTATTTTTTAAATATAGGTGAGGCACCGTTTGCGGAAAGGTTATGGCATTGAAGGGGGCATTCCTGTTGTATTTTCTTTAGAAAAACCCAAAGCCAAGCTGCTTCCATTCCAAGGACCAAGTGGAGAAGCTGAAAATCCTTCAGATTATCAGATAACTCCTGGGTTCAGAGTTCGCATCATACCCGTTCTAGGTACCATCCCTGCAATTTTTGGACAGGTAATGGCCTCATATGTTGTGACACAATTAGCAGGGCTTCAAGTGGACACAGAGCCTGTCATTAATTTGGACTCAGATCATTACAAGTTGCTTCATCAGCGACTCATTGAGCATGAAGAATCATTGTATGGCACTGCCATGGAAGTCCAGGTAGATCTTGAAGAAGTGATGTTTGTTGCAAAAGAGCTCTGGCATGGAAGAAGTGCGAGAGAGCAGTCTGCAAGAGATGTTGGACGAGGATTATGGCGAGCTATCAATGAATTAATGCTTGTGAGGTGGGACCGAGCAAAACCAGCATCCGTGTCGAACTTAATTCTATTGAAATTTAATGAGGCTGACGAACACGAGTCGTCGACCCTAGATGACATAAAGGAAAATGAACCTGAATTCTTTAGGAGAGTGACTGCTACTTTGAAGCGAGCTGAGGTGGAATTTTCTTTATGAGAAGATAAATTTCTTAGTTGGAATAGATAAGGATTTGTTGCTTTCGTTCCTAATAGTATCATTTCTTTAGTTCAGCATGGGAAGTTACTAGGTTAATAGTGACTTAAGCCTTAGATTTAGCCCAATATTATCAGTTTTACTGAAGTAAAATTTTGGTCTAGTTAGTTGTTTTTGAGATCATGGTTTATTTTGGTTTAGTTGTTTTTTGAGATAGTGATTTTTTTTGTTTTTTGTTTTTGCTTTTGAGGAAGAGATCATGAAATTTGTTCATTTTCTG

>cucumber_newGene_518 cucumber_newGene_518.3

AGAAGAGGGAAAGGGAGATTTGAGAAGTAAAAGGCAAGAAGAGTGGCAATGTAAAATATTGCGTTTAAGGGTCGCTTCATTTTCTAATTCTATCTTTGCACCCCCATCCCCACCGGAATTTTATTTTATTTTTTCCTTGCGCTCCCAATTTCGACGGAGCTAGGGTTTTTCTTTTCTTTTCTTTACTTTCTCCGATCATGGCTTTGAGAATCGTCTTCCTCATCCTCCTCATCGCCGCCATAGCCGTCGCTTGCTTCACGCTCCCTCTCCAAAAGATTTTGAAGGATTTCTTGTTATGGGTTCATCGGGATCTTGGAGTCTGGGGTCCAGTCGTGCTGTGAGTCAACTCTCCTCATTTCTGACATGTTTGAGAGTGATTTTGAAATTGGTTTAATCACTTTTGTGATTGACCATATTCAAAATCACTTTGAAATATGAAATCCTTCAAAACTAATTATGTTAGTATGAAAATTGCTTTTAGGGGGCATTTGACCCGCTAAGTGGGGTTAATAAGTTCAAAGTTAATATGTTAGCTAGAGTTAGTAAGTTCGTGTTTTGGGTGTAGAGTTGTAAGGCGAAATTTGTCTATAATCGTGAAAAGTAGATTAAAAAGAGAAGATAGAGTTGCTCGATAAATAAAGTAGAGATGGAGAGGGAGATGAGTTTGCTCGATAAATGTGAAGAGTATGAAAAAATAGGAAGGTAGAGTTACTTGATAAATGTGCAATAGTGAAGTTGTTTACACTCACTCAAGAAATTGGTGGGCCAAAGGACCCCTTAAATTGAGTTGGAATGATTGAAGTCATGTCTCAAATTGATTTGAAATAAATTATTTTCAAGCATACCCTTTTGAAATGATCCCTACGTTGGTTTAAGTATCCTCACCTCAACGAATACAATTCTTATGAAGCACTATAAAGCATTTTCTGCTGTTAAATGCAGAGCTGTTGCATACATTCCATTGACAATCATGGCTGTTCCAGCCTCAATCTTGACACTTGGGGGTGGTTACTTATTTGGGTTGCCCATTGGCATTGCTGCTGATTCTATTGGTGCAACTGCTGGAGCTGGGGCTGCATTTCTTCTTGGAAGAACAATTGGGAAGTCATTTGTTGTTTCCAAGTTGAAGGACTATCCGCAGTTTCGTTCAGTTGCAATCGCAATTCATAAGTCGGGGTTTAAGATCATTCTGTTACTTCGGCTTGTTCCTTTGCTTCCATTCAACATAATGAATTATCTTTTGTCTGTAACTCCTATTTCACTAGGAAAGTACATGTTGGCTTCCTGGTTGGGAATGATGCCAAGTACAGTTGCATTAGTTTATGTGGGAACAACTCTTAAGGATTTATCGGATGTAACTCACGGCTGGAATGAATTTCCAAAGTCTGATTGGGCATTTATCATGATGGGCCTTTTCATATCTGTGGTTCTAATAATTTGCATTACAAGAGTCGCCAAGTCTGCTCTAGATAAAGCTTTGGCTGAAAATGAGGATTATGACGATATCACATATGGTGAGCTACCGGTTGTTGCCGATTTTCTTGTAGATTTAAAGCAGCCTTTGATACTGAAGATAGACCCAACTGAAGATAACCATGAAAAATGACAATGAAAGCTTGTGGTTTGTTATGTCTCTGTACAGTTCCACGATCTCCTTTATTTGTGTAGAAGAACAAGCTTGATTTCTTTTGATTGAGCCGTGTATTATTAGAGTAGCTTAGCTTCTGCCTTGTGATTGGGGCTGAATGTATATATATATATATATACACACGATCTTGACAGATTACTTCTAGGGTGGAAATGAAGAGTAAAAATTGTCCAAGTTGGTACAAAAATCACTCAACTGGAAATATATTGTGTCTTGGGTTGATCCCTTTTTT

>cucumber_newGene_519 cucumber_newGene_519.1

AAAATATATCATTATATTTAATAAAAATTTCAAAATAGAATAAACCCATCTTCTTCTTCTTCTCTCTCTCAGTTCAGTGACTGAAGTTGAACTGAGGGAGACCCAAAAGTTGAGGATTTTGTTGTTTTTTGGTGAAATTTTACTGTTTGATATGAATAAAAAAAGAAAGAAGAAATGAAATGAAAATGGATGGGTTGGTACTTTTTGGTGTGTATTTGAAGCTGTGATTGTTTTCAAAAAAATTGAGTAGAGAGTGGCAGCATTTGTGGCCAACTCCAAATCATTGCTTTCTCTTACTTTTGTCTCCAAAAATCTTTCTCCTTATATTATTTTCACTTTCATACTTGGTTTCATCCCCCATTTTCCTCTCTTCCAAAAGCAAAAATCATTAGGGTTTTCTTTTGGGTTTTTCTCAGATCATCCCCTTTTTTTTTTCATCTGGGTTTTTGCCGGACACATATTTGCCGGAATGTAATGGGGGAATGGATGATTCAATGAGTTTTCTGTTGTGGTTTCATTTTAATGGGGTGTGTTTTTGGGCGAGAGGTATCTGAGAATCCCGTCCAGAAAGAGGAGGAAGACAGAAGACAGAACGGGGAGGAGGAAATTAGGGTTTCTGTTAAGCCGGAGGTTGTTCAGGTTCAGGTTAACAATGCTCGTAATGGTGGAAGTAGGAAGGAGGTTGAAGATGATAGAGGTTCGAGGCAGAGAAGTGAGCGGCGGCGACGGCCGAATCCGAGATCGAGTAATCCGCCGAAACATGTGCATGGGGAGCAAGTTGCCGCCGGTTGGCCGTCTTGGCTCTCTGCTGTCGCCGGTGAAGCTATCAATGGATGGATTCCTCGCCGTGCTGACTCTTTTGAGAAGCTTGATAAAATTGGGCAAGGGACGTATAGTAATGTTTACAAAGCAAGGGATTCTTTGACAGGGAAGATTGTGGCTCTAAAGAAAGTTCGTTTTGACAATTTGGAGCCTGAGAGTGTGAGATTTATGGCCCGAGAGATTCTCATCCTGCGGCGTCTTGATCATCCCAATGTTGTAAAACTCGAAGGTTTGGTGACGTCCCGGATGTCGTGTAGTTTATATCTTGTATTTGAATACATGGAGCATGATTTGGCTGGACTTGCTGCGAGCCCCACGATCAAGTTTACTGAACCGCAGGTTAAATGCTACATGAACCAATTGTTATCAGGGCTTGAACACTGTCACAACCGCTACGTGCTGCATCGTGATATAAAGGGATCAAATCTTCTGATTGGTAATGATGGGATCCTTAAGATAGCGGATTTTGGATTGGCTTCCGTCTTTGATCCCAACCACAAGCAGCCAATGACAAGTAGGGTGGTTACTCTATGGTATCGACCTCCCGAACTTCTTCTTGGAGCTACTGATTATGGTGTTGGTGTAGACCTTTGGAGTGCTGGCTGCATTTTAGCTGAGCTGTTAGCCGGAAAGCCTATAATGCCCGGTCGCACAGAGGTCGAACAGCTACATAAGATATTCAAGTTATGTGGCTCTCCTACAGATGAATACTGGAAAAAGTCAAGGTTGCCTCATGCAACCATATTCAAACCTCAACATTCATACAAGAGATGCATAACAGAGACATTTAAAGACTTCCCACCATCGTCTCTGCCACTTATCGAGACACTTCTTGCAATCGACCCAGCTGAACGCCTGACCGCCACTGCTGCTTTAAACAGTGAATTTTTCACTACCAAACCATATGCTTGTGAGCCTTCCAGCCTCCCCAAGTATCCTCCAAGCAAGGAAATGGATGCAAAACTGCGGGATGAAGAAGCAAGAAGACTAAGAGCTGCTGGCAGAAGCAATGTAGATGGGGTGAAGAAATCACGAGCACGTGATCGAGCTGTTCGTGCAATACCTGCCCCAGAAGCCAATGCTGAGCTTCAAGCCAATCTCGATAGGCGGCGTCTTATAACACACGCGAATGCGAAGAGCAAGAGTGAAAAGTTTCCTCCTCCACATCAAGATGGAGCACTTGGCTACCCTCTAGCATCTTCGCATCACATCGATCCAATCTACGATCCTCCCGACGTTCCATTTAGCACGATGAATTTCTCGTATCCAAAAGCAAACATTCACACATGGTCTGGTCCTTTGATGGACCCAGCTGCTGTTGGTGCTCCAAGACGGAAAAAACACACAGCAGGGGACGGCCATTCATCAAAATCATCTAAGGACTTACGAAAAGACAAGAGTTCTGCTCGGATCTAATTCACTTAAATCGGTTTCGATGAGCTTCAAATCGTTTAGAGATCTTCATCCATCATTTCTAAAGTCAATCCCGGGAAATTCGATTGAATTCTATGGAGAGGTAAAGAGTATATCATTTCTCTTTTTTTTATTTTCAGTTTTCTATAATTTTGTAAG

>cucumber_newGene_520 cucumber_newGene_520.1

GTTTCATATTCGGTTAGAAGAAATCTAGAAATGTGCTACATCCACCCAATTGATAAGAAGGAGTGCCAAGTTGTTATTGGAGGAGATTACATTACAACTGAGACAGGAACTGGGTTAGTCCATACGGCACCTGGCCATGGTCAGGAGGATTATGCGACTGGTTTGAAGTATGGACTCCCCATAATATCTCCTGTAGATGATGATGGGAAGTTCACTGAAGAAGCTGGTCAATTCAGCGGGCTTGATGTTCTTGGGGATGGCAATACTGCTGTTGTCAAATATTTGGACGATCACTTCTCACTTATTTTGGAAGAATCGTATGAACATAAATATCCATATGATTGGCGAACGAAGAAACCAACTATTTTTAGGGCAACTGAGCAATGGTTTGCATCAGTGGAGGGATTTCGCCAGGCTGCTGTGGATGCTATTGGCGAAGTAAAGTGGATACCTCCTCAGGCTGAAAATAGAATATCTGCAATGACTTCAAGCCGTTCTGATTGGTGCATATCTCGGCAAAGGACATGGGGTGTCCCAATACCAGTTTTTTATCATGTGGAAACAAAAGAACCTCTTATGAATGATGACACCATAAACCACATCAAGTCAATAATAGCCAAAAAGGGTAGTGATGCTTGGTGGTATATGGCAGTAGACGTTCTGCTTCCTGATAAATATCGAGAAATTGCATCTGACTATGAAAAGGGGACTGATACAATGGATGTATGGTTTGATTCAGGATCATCTTGGGCGGCAGTATTAGGGACACGAGATGGGTTTAGTTTTCCTGCTGATTTGTACCTTGAAGGTACAGATCAGCATCGTGGCTGGTTCCAAAGTTCTTTGTTGACATGCATTGCCACAAAAGGGAAGGCTCCGTATGCTAGTGTCGTTACTCACGGATTTGTTTTGGATGAGAAAGGTCTGAAAATGAGCAAATCTTTGGGTAATGTAGTTGACCCTAAGGGTGTGATTGAAGGAGGAAAGAATCAAAAGGAAGCCCCTGGCTATGGAGCAGATGTCCTGCGCCTTTGGGTTTCCAGTGTGGATTACACAGGCGATGTGATGATCGGTTCTCAAGTTCTCCGTCAAATGTCAGATATATATAGAAAACTACGGGGAACATTAAGGTTCCTTCTAGGAAATCTGCATGATTGGACAGAGGAAAATGCTGTACAATATCAGGATCTTCCAAAAATTGATCAGCATGCATTGTTTCAGCTTGAAAATGTCATTAACAACATCAGAGAAAGCTACGAAAGCTACCAATTCTTTAAGATCTTTCAGATCATTCAACGGTTTGTAATTGTTGATCTATCAAATTTCTACTTCGATGTTGCTAAAGATCGATTGTATGTTGGGGGATCCACGAGTTTTACTAGGAGAAGTTGTCAGACGGTTCTAGCAGCGCATGTAGTTTCCATAGCAAGAATAATCGCTCCAATATTGCCTCATTTGGCCGAGGATGTGTGGCAGAATCTTCCGTTCCAGCATACAGACGATGATGGTTCTGTTGCCAAATTTGTCTTTGAATCAAGGTGGCCAAGTTTAAACAAGACTAGGCTTTCTCTTCCCGAAGAAGAAATTGATTTATGGGCAAATATTCTCGAGCTAAGAACCGAGGTGAATAAAGTCCTGGAGGCTGCTCGAATTGGAAAGTTGATTGGTTCAAGCTTAGAGGCTAAAGTCCATCTGCATGCCCCAGGAGATGGTCTGTCTTCTAAGTTGTGTGAAATGTGTGAATCTAACCATGATGCAGACACATTGCAAAGAATATTTATTACATCTCAGGTTGAGGTTCATCAGTCCATAGAGACTGAGCATATAGAGAACGTACCATATACCGGAGAGTGCCTCATTGGTGGCAACAAAGTCTGGATTGGTGTGTCTCGTGCTGAAGGTTCAAAGTGTGAAAGATGTTGGAACTTTTCACTACGAGTAGGGTCTTTCCCAGAACATCCAACTCTCTGCAAGCGCTGTTTCAATGTCGTTGCTGGCCACCCAGAACCAGCCATGGCAGCAGTCAGCTGAGACTCTTGATGACGGTTATTGATTTTTGACAAAAGTTCCATTCTTTTCACCCGGTATTTGCCCTCCCTTGCTAAAGATGAGAAGTTACAATGGTGGTTGGTTTCCATTTTTTTTTCTTTGAAATAATGGTGTATATTATTTTCCATTAAATTGTATTGATGACATTAAATTATGGTGCATTTTCTTTAGTAGGCTCAGACTCAATTCATACAGCATAGCTTTAGCTTTAGCTCTTGTGATTTGCCATCGAATACACCAAATGTTCACATTCAGATAGCTGGGAAGTTTGACAGACCATAGGACGTCGAAATGATATATTGTTTTTAATTTTATTTGTAATAACTCCTCGATCTTTTTTGTGGTGAATTTAATATTAGAATGTCGAAATGAAATATTT

>cucumber_newGene_521 cucumber_newGene_521.2

GCAACTTTCATTTATGGAGGGAGACGAGAAGTAGGCTTTGTGCGGCAATGAGTGGGAGCAACGTAAACAAGGTCTTTTACGCCGATAACTACCATCCCATTCAGGCCGGCAGCATCGACGGCACCGACATTCTTTCCCACGACAACGCCGTCTACCGAGCTTTACTCTGTTCTTCTGCTGGCCTCTATGACCCCCATGGCGATCCCAAGGTCTTCGGTGACCCTTATTGCACCCTCTTCGTTGGTCGCCTTTCACATCTCACTACTGAAGACACTCTTCGCAGGGCAATGAGCAAGTATGGACAGGTTAAAAATCTACGCTTAGTCAGACACATTGTAACTGGTTCTTCACGTGGTTATGCTTTTGTTGAATATGAAACTGAAAAAGAGATGCAACGTGCATATAAGGATGCTCATCATTCAATGATAGATGATTGTGAAATTATAGTTGATTACAATCGACAACGGCTTATGTCTGGATGGATTCCTCGGAGATTAGGAGGGGGTCTTGGAGGTAAGAAGGAATCTGGACAACTTCGATTTGGAGGAAGAGAAAGACCATTCCGCGCTCCACTACGTCCGATCCCTTACGACGATTTGAAAAGGCTTGGGATTACTCCTCCTCCTGAAGGAAGATACAAGTCTCGGTTTCAGATACCTTCTCCTCCCAGAAGAGAAACGGATTCTGAACGTAGAGAAGAAGGCTCCAATAAGAATGAACCTACGGAGAAGGATGGACACAGTCATAGAAATAGTTCGATAGATGAAGATGATTGGTCACATACGAGGGGCTCAAATGACTACAGAGTGGGGCACTCACACAAGGGAAACCATGAGAAGATGAAACATGAATCTAGGAGGACCTCAAACGACAGGGATGATTATTCCCCCCACAAGAGATTTTCCGAGAAGGAAGACTATTATCAAAGCAGATCTGCTGAGAAGGAAGATCTTTATCGGAGGAGCTCTTCAGATAAGGACCGATCTCGCAGAAGAGATTCTAAAGATCGGGATGACCACTCAAGGAAGCATCACAAATCTCATAGATAGTGACTCCATTACCCATCTTAATAGGAATGAAGCCGATCTTGTTCACGTGTTGATCTTCATTTATGACGAACAAAAAAGTTAATCCATGTTAAATTAAATGAAGTTAAGTGACATTAAAGTTGAAGTTATGGAAGCCCTTTAGTTGCATTAAATGAATTCAAGTATATCTCAAGCTTCATGCTGTACAAGTCGAGCATCCATTCAGGAAACTACCGTTCAAATTGAGATTCATTTATACATGGGAGGTTCAGAGACTTTCAAGTCTGACAAAAAGGCTTCAATCTCAAATTTATGGTTCATCATTTTACTGTAACTATATAGTAAACCTTTATGTTTTTCAGATTACTTTGGAGTGAGTATCATGACACTCGTGTACTTCGAAGTTACAACCCATGAGAGTTTTGTTTTTATGGGTTAGGAAGTTACAACCCTTAAGTTGATATTTGGTTACTTAGCTAATTTTTATGTAAAATCACTTGAAGGATATAATAGAGTAAAAAGAG

>cucumber_newGene_524 cucumber_newGene_524.1

ATTAAATTGATTGTTCTATTTTGTTTCTTTTTTTGTAGGTTCATTTACTTTACTGCTTATGAAATAGATGCAAATTGCCATTTCAAATATTTTTTCATGGCTATTGCATCATCAATTGAAGGTTGGAGATTTTGTAGACCAAATATAGCCGTTAATGGGACATTTTTAAAGTGTAAGTATGGTGGAACATTGTTAACGGCAGCAACTATGGATGATCATAGTAAAATTTTCCCTCTTGCATTTAGTATAGTAGATTCAGAGAATGATGCTTCTTGGAAATGATTTTTTGAACAATTAAAACTTTCTCTTGGAGATCGAGAGGGATTGGTCATTGCTTCTGATAGACATATAAGTATTTTGAAAGGTGTTTTAGATGTGTTTCCAATGGTACAATATTGCGTTTGTGTAGAACATCTTTTGAAGAGTGTCAAGTTGTCATTTAAAGACTCTTTAGTTGAAAATATTTTTTGTCAAAGTGCATACTCTTATACAATAGATGATTCTGAGTTACATATGAGATGGATGGAGTCGATATACCCATCTATTAAAGGATATCTTATGAAAGTTGGTTTTGAGAGATGATCACGCGCATACTCTAGAAAAAGAGAAGATATCAAATAATGACAACATATATTTGTGAAAGTTTTAATTTTAAGTTGAAGATTGATAGAGACTTACCAGTTTCATCCTTGCTTGAGGCCATTAGAGAGTTTATTCAACAATGATTTTATGAGAGAAGAGAAGCAGTCTCATGTTCAAAGAGTGTTTGAGTCCTTTGGCTGAAGGAATAATAAAAAAGCTAGTTGATGAATCACAAAGCTTCATTGTTAATTCTATGAGTGAAGTTGAATTTCAAGTAGTTGATGGAGGCAAGAATTTTTTAGTAAAGTTGAATTGTAATAGCTGCACTTGTATTTTTTGGGATCTAGAAGAAATTTCATGTGCTCATGCTCTTCTTGTGATTCGTAGCCTTAATTTGGATCCTTATGCATTTGTTTCACTCCATTATTATGCTAGTGTATTATTTGCAATTTATTGTGGATTAGTTCATCTGATTGGCAACCATACTAATTGGAGTGTTGTGGAGGTGAATGACAATGTATTGCCTCTAGTATACAGACGTTCA

>cucumber_newGene_525 cucumber_newGene_525.1

ATTGTCTCATCTTTCTATTTTTATTCACCATCAATCTTCTAATTTGATTTCCCCTCCTACTCTGTTCTCCTTTTGCTTTCTTTTAACTCTTGTTTGGAATCAATCTAGAGTGCTATATACTCTTAATCCGCATTTTAGTGTGTTACACACGAAATTGGAAGTGAGTTGAAGGGGAGGAAAGGAGTCACAAAACAAAACATGGTCATACTGTCATTGGTTCATAGATGAAAATCAAGTTACTGAAGGGTAATTCAATGTCTAGTCTTCCTAACCCATAGTAGTATATGTTTCATATAGTGAAAATTGATGAATTGACGTGGATCCATGTGTCTTAGAGTTCAAATTGATTGGGAATTTAAGGATGTGGATGAAATTTTTTTACTTGTTTTTGTATTTGTTGGATTACATATATCTTCAAGGGCTTAGACTTGAAGCTTGGAGTGCATAGTTTCTTTTCTGAGGCACCATGAAGCAACATGGTTGTTCAAATGGATGCACGGAGATGGGTAAATGTTTTTTCGTTGTTGATATTGATTTCATGGGGCTGGGTAATGATTTTCAACATCTTTGTGGGTTTTTGTTATTTTTTTACTTTGTTTTAGCTGTTGGTTTATTTGACTAGTGGCTTTCATGAGATTTTGTGATCGTGTATGGTGAATCATAAAGAGACAAAACTTTGCTTTATTTTTCAATAGAGAAGTCAACTGCTTCATTTTCACTGTTGGTTAGAGGTAGAAATATAAGAAGTTTGATATTTAAGACCATTTGTGTAATTCTTTCGGTTGTTATGAATTTGTTTGATCACTCACATCGTTTATTCTTTATTTCATATTTCATTGTGTTTCTTCTAATAGCCAAAGATCTTATACGTTGTTTATGAAGTTTGAATTTAGGGTTAGTGTTTGAGGTTAGAATATTTACAAGGACTAAAATGGTGTTTAGGTGAAGTTTAGAGCCTTTATCTGTTTATTGAGAACTTCATTTAAATTTTTTGATATAGACAGGTTTTCTTGATGGTTTGGAGATGGAAAATAACTCAAACGTACAAAATTAGTTCTAAATAAATTTCAAAGGTAACTTACATAGTTACCGGTTAAGAACCCAAAAACTTTGATGTTGGATGAGATTATCAACTATGTGAAGTCATTACAACACCGAGTTGAGGTAATTATTATATTTTAGAATAAAATTTAAAAAAATGTGGTCCACTTAGCAGTTTAAAAAAAAAGTTATTTTTATGTAGATAATTGCTCTTTTTGCTCTTTTTGCTCTTTTTGGGCTTGGTTATGGTCCTTCGTACATAATTGCTCTGCATCTTGAGATAAGTTAGTTGTATAAAATTGAATTGTGTATATGATAGGATTTTCTACCTTATTGCTTATTTACGAATCAATTATACTATACCATATGAATTCATATCCAAGAAGAAGTTTGAGGTAGTGTTGTTATTAGTATACATAAGCTTCGGTTAGTACATTTTTTCTTTTTTTTGCTTTAAGAGAAACTAGTATTGATCCTTAAGTTTCATAAACTATTTTTTGAAAATATTGGACATTTTTTTCTTTAAATGATTTAAGACTTACTAAAATTTGTATAATGTAAGTTATAGTTTCTCGATCGACAACATACACCTATTTGTTACATTCAAGAGCTGTATATTTTCTTACTTGTTTTTAAGTTTCCTTTACAGTGTGTTTTAATGGGATCCTAGGATTGTCTTCTTATATGCATCTAAATAATCCAAAAATGTCAATGGAAGAAGGTGATGGAAAGCTTTGATATGAAAAAAATTGATCAACACAACAAAAGAAGGTACGTGATGTGTTCAAAACATCAGTTCTTGTTGCAATCGTTAAAGGCGAGATCGCTAGGTTGCTCCCTTTTAAAGAGGTTAGTATGCTGGTTGATTTTGTAAAATTTCATTCCTTCTAGAACTTTATAAAAATATTTGCAAATGTGTGATTTAATGGTTGT

>cucumber_newGene_526 cucumber_newGene_526.1

GGGAAAGTGGGTGAGTTATTTTGGAAAGATGAGATCCAAAAGCCTTTTCATTTAATAAGTAGGAGACGTGGCGGGTGGGAATTTCCAAGCTCATTTAACAAAAAAATTGCTTTACCAATTTGCAATACACATATCTATCTCACCCCCACAAAAAAGTAATCAAATAAAAAGTAGATTCTGATCTAAACACAGTGTCTGGCGGCGATGGAGAGATCAGCGGTGGCGATTTTGTGTTTATTGTTACTTTCTGCGGTGGTGACGGTTTCAGCGGACGCCGCCGACTGCATTGACGGCTGTTTCACTGCTTGTGTCCAGAAAGACTCTAGGGCGATGCAGCGTTGTGAGAGGAAGTGTACAATCAAGTGCGGTCCAGATAACAAAGTTGAGGAAAATACTGCACGATGAGACAGAAGAACAAAGGGTAAGGGAGTGAATGTTGTGAGAAATCTTACCTTCTTGTGTTGTAATAAAGATAGTTGAGGGAAGATACTTTATGTATGTTATAAACAAATGTTTCCTTCTTCTTCTTCTTCTTCTTTCTTGTTTTAATAACTATGATGTGATGTTTGTGAAAGCTAGACGTAAGCATTCTCTAAATTAGTGCTTTTCTTTGTGGCATATGTGTTTTTTTCTCTCTCAACATTTGAATTAGA

>cucumber_newGene_528 cucumber_newGene_528.1

GTTTAACCAACTCAAAAGCGAGAGGAGACGAGAAAAAAAGATGCTTCTTTTGAAACTTGATCTTCTTCTACAACCCCGTCGATTCCTCTCCCTGTCTCTAGAACTACTGCGACAGCATCCTTAACGATTCTCTCACAGGTCTTTGGCGTTTTTGTTGTTAAGATCCTCTAATGGTTGTTGTTCAGGCTACCAAGCTCAGCCTTCCCAACCCTTCTCTCTCTTCCCCACAAATTTCTTCCCTTCTCTTTGAACCTCATTCTCTTTCTCTTGCTCTAATGCACTCAGATTCTTCTTTCTCCCTCTACCCTTCTTTTTCCCCTCTCTCACTTTCCTCTCTTCCTTCCCCTCAAGTGGTTGTGCCTTCTCCGTGTTCTTCTGCGGCTTTTGTTGCCCTTCAGAATTCTAATTCCAACTCCGATACTAAGGTTCTGTTTGTGGTCTCTGGCCCCCACAAGGGTGGCTCTCAGATTCTCCTTCGGTTTTATGTTTTGGAGGGTTCTAAATTGTTCAGGAGAGCTCCAGTTGTTTGCACGCAGAAGGATCTTCGATCTGATGATAAATTGGGTGTTTGGGTCAATTTTAGACATGGGATTTCGGTTCGTTTAGCTGGGTCAGTCAATTTCTTCGCCATGTACTCTGTTTCGAGCATGAAGATTTGGGTTTTTGCGGTGAAGATGGTGGGAGACGGCGATGATGGGATAGGTTTGAAATTGATGAGATGTGCTGTGATTGATTGCTGCAAACCCATTTGGTCGCTTAACATTTCGTTTGGGTTTTTGCTTTTGGGGGAAGATAATGGGATTAGGGTTGTTAATTTGAGACCCTTTGTGAGAGGGCGTGGTCGAAAAGTTAGGAATCTGAATGCAAATACCTCTTCTAATGCCAAGCGTGAAGTTCAGAAGTCATTTTTGCCTCATGTAGATGTTTGTGGAACTTCTGGTGGCAATGACTTGAACGGTGGTTCTTTAGTTGTTAGCAGCAATGGATTCAATTTACAAGCCAGTAGAAGTGAAGATGCTGGAAGTTTATCTTGCAATGGATGCTTGGACGGGAAGTTGGACAAAATTTCTAGTTCTGATTGGAAGGTCTTCTAGTATGGAGATCAAGATGAAGATTGAGATCTGAAGGACAGAGCCAGCGAGAGGAAGACGAAAACCCACGATAAAGAGGAGGATTGAAATCCACGTCGAAGAGCTACAAAGACCCACGACGAAAACACAGATCTGGAGAAAACCCACAGCACAAACCCAGATCAGCACGAAAAGGAAGAGGAAAATCCAAATCCGACGAAGAGGAAGACGATGAGAGGCGAGGAGGAAGATCGTGAAGACAGTGAGAGGAAGAGGAAAACAATGGTTATTTGGGGAAGACGATGGAGATAAATAAGAAGAAGAAAAAGAAAACCACGTGGAGAGTGGTTATTTGGGGACGAAGATGGAAATAAGAGAAAGAAAAAGGAAATCACGTACGAAAATGGAAAAGGAAAAGGAAATAGAAAAATAATTAAGAAAAAAGGAAAATGAAACCAATAAAAATAATTGAGAAAAAGGAAAAG

>cucumber_newGene_529 cucumber_newGene_529.1

AAAGTATTGGAAAGATTTAATACTTTCCATATACAAAAAATCGTCCCATGTACTCCATTTTACTCGCTGAAGACAAAAACGATAGGGAGAGTTCGACAGAAAGTTTGAAGACGAAGAAGATGTTCGACGGCCGACCACTGTGAAAAAATTTTGCTCGAAACTTTGAAGAAATTTTGAGGTATCAAATAAGAGAAAGAGAACTGGAAAGCTAAAAAGAAAAGTAGAGAAGAAGGAAACTATTAAAAAGGAAAAACAAAGTGAAAAAAAATATGAAAAAAGGAAGGACAGAAACTACACAGAATTCTAAAGTGCATAAAAAAGAGCCGTCCCATGAAGGATATTGATCAGGTGCAGTTTATTTAATTTATACATACTAAGTTAAATTTAGTTCTATAAGGAAGATAGTAAAATTCTCCATGTACCTTGAGCACATTTCCCCAACGAATTTTTGGGTCGTTTATAATTCGACGACCCAACGTTGATGCTGGATCCTCATTTGGAGGCTTGTTGTGAATCGAAGACTCCAGAAACCAAGATTTTAAAGATGAATTTGGATTTACAAACCGTTCCTTGGACCATTTAGGCAAAAAGATAAAATTGTCCTCGGGTATTTGGGAGTCCATAATATTCTCAAATGCCCTCTCTCTCACAGCACGAATGATCCCTTCCTCTAAAAATTGATCTCACAGGAAGAATGATCCCTTCCTCTAAAGATTGATCTACGTCCTTCAGGATAATTAAACACCTTTTGGTCGAGTCAGAATACTCAGTGAAGTGCACCATGATGAAAAGTAGAGTTCCTG

>cucumber_newGene_531 cucumber_newGene_531.2

GGATATAGCTTAAAACTGTGAGTGAAAAAATGAGCATGAATTATGTTTACAACCTGAATTTCTAGTTACTTTCTTCTCTGTCCATACCCACAATACCCTTTCTTCTCCTTCCCCGACTTCTTCTCCTTCTTCTCCTTCCTAAATTGCTCCACATCATTTGTCGCGCACACCGATCACTTCTTCTCTTCTTCAGACTCTTGATGGAGGCCCCACTCGTTGGTCTTTCACCTCGTCAAGGGATAGTTCTTCCTCAACTTTTGTGACAGATTCCCTTTCATGAGCTACCTTCTTCTGCTCCATGGGCTGACTCTAGCCACACGCACGACAGTCGAAGCTCGATCACACTACCTTCTTTCCCATTCAAGCTCGTGGTTATTCACACTGCTTCGTCCTCTGTCAATCTCATCGGACCTGGTAATTGATTTCGTTTTCTACTTGTTTTGAATAAACTGATTTACGTTTATCCTCTTGATTTGATTAATAAGATGATTTGAATAAAAACCTAATTGCTACTTTGCATCTCAAGAATTAAAAAGCCCTAAATATCCATTTGGTTATGGTAAAATTAGGTCACTCTTTCCACTGCTTGGTTAACCTCAACTAGAAAGAATTAGGCTCATAATCTCAAAATACTCTATGCATGCAAACACATTCTACTATGTAAGTAACTGGCCTTATTAAACCTCACAATCTCTTCCTACATTGATTGACAATATACTTTATCATCATTCTTCAATTGACCACACAAGCCGAAAAGCTTATCATGACCAAGTTTTTTTTGTGAAGCTAACACTGACTTCATTCATTGAATGAATAGCAATAACCAATATGGTAGAAAGCAAATAATCACATTAGAAGCCAAATCAAACTAAAAAAGGGAAGAGAAATTTAACTTACAGTTTGCACAATTAAAATATAAGTCTAGATTTGACAATATTATTTTTATCATTTTCCTGTAAAATATTATTAAAGATCAATAAATTTTAGAACTTCAAAATAAACCTGAAACTCAACAGTTGAGTTATCAAGTTGTGGAAATTTAGAATGAATGAGGGCACAACTATTACTTTAGACACGCAACGCATTCTGATATGCCTTCAAAGATTGCAAAAGCTTACTATAATCCCACGCTTGGATAACAAAAAAAGTTGTAAGGCATGCATTTCCTAGATTATTTGAAAAACAAAATAGTGTAGATGTGTTATATTATTATTTGCGTTTATGTTATGTACAAATCATTTGTGTGTTCAATTGTTGTTGGAGAAAAATTGAAGATTCAGAGCCTCCTACTTCGAATTCTATTATGAAGATGGGCTCCAGATTATTACAATGCCCCTTTGATCATAGAAGGTTGTCAAGAAGATGGTGAAGAAGATGGTGAAGAAGATGACAATAGAGGAATTTAGATGAAGAGAGAAAGATGCAATCCTAAACTAGATGAAGGACAGTAAGGTTTTTAGATGAAGAACGAATTTGTTTATTTTATGTTTGAAATCTTTTTATGTGAATGAGATGACTTGATCTCCTAATCTCCTAATACTAGCAGCAAAATTGTAAGATTAATAGTTAGCAGGTTTTTATTTTGCTATGTTTATATTAACTGCCTTCGTTAATCAATCAACATTCTG

>cucumber_newGene_533 cucumber_newGene_533.1

CATCTCAGCCTCTCGCTCCCATGTCGCCTCTTCAATTCGATGATTCCGCCACAAAACCTTTACTAATGGAATGCTTCTATTACGAAGCATCTTTACCTCTCTAGCCAGAATCTCCACAGGTTGTTCTACATAGCTCAAATGCTCATCAATCTCCAAGGGTTCATAGTCCACAACATGAGATGTATCGGCCACATACTTCCTCAACATCGAAACATGAAAAACATTATGAACTGCAGAGAGAGATGGTGGTAATGCCAAGCGATACGCCACAACACCAACCCTCTCTAAGATCTCAAATGGTCCAACAAAACGAGGACTTAACTTTCCTTTCTTCTCAAAACGCATAACACCCTTCATAGGTGCTACCTTCAAGAATATCTCCCACCGCAAACTCAAGGTTTTTCCGTCTCACATCCGCATAACTCTTCTGTCTACTCTGCGCTGTTTGCATACGTGCTCTAATCTTTTGAATAGCCTCATTAGTAGACCGAACTAATTCAGGTCCCATCAATCTATGTTCACCAACCTCACTCCAACAAACAGGGGTTCTACAACATTTGCCATACAAAGCCTCAAATGGTGCCATACCGATGGTAGCCTGGAAACTGTTGTTATAAGCAAACTCCATCAAATGTAGATGAGAGTCCCAACTACCTGGAAACTCTATAACACAAGCTCGTAGCATATCCTCTAAAACTTGGTTCAAACGTTCAGTTTGACCATCAGTCTGTGGATGAAAGGCTGTACTGAAATCCAATCTCGTACCCATAGCAGTCTGAAGTCCCTTCCAAAACTTGGAAGTAAACCGTGCATCCCTATCAGAAACAATCGATACAGGCACTCCATGCAGTCTCACAATCTCAGTTAAATACAACTCTGCCCACTTACTAGCAGTATAAGTGGATTTTCCTGGTACAAAATGTGCCGATTTCGTAAGCCTGTCTACGACAACCAAAATCACTGTGAAACCCTTCAGGGTCTTAGGCAGCCCTGAAATAAAATCCATCGACACATTCTCCCACTTCCACTCTGGCACACTTAAAGGTTGTAACAAACCTGCTGGTTTCTGTCTAGGTGCCTTAACTTGCTGACATACTAAGCACTTACTAACAAGATCTTCCATTTCTCTCTTCATATTACGCTAGTAACAAACTCTTTTCAGATCCGGATACATTTTTGTGCTACTTGGATGCATGAGAAATGGGAAACTATGAGTCTCAACCAATAACCTTGTCTTAATTGCATTTTCTGTTGGCACACACAATTCTCTCTCAAACATAAGTTCATCATCAAAGGATATGGAAAACTCCTTAACTTGCCCTACTTCTACTAGGCGATGCTTCTCAATCAAATAAGGATCAATGAATTGAAC

>cucumber_newGene_535 cucumber_newGene_535.1

AAAAAGGGAAAGGCACCGTTTTCAAGATTGTCGTGCTTTGCCCTAAAAATCCCAATTTCCAAATTTCCCAATTTCGCATCGTCTTCTTCTTCTTCGTAATTTCTCAATTTCGAATCATCTTCTTCTTCTTCACGGCTAAGTATTCAAAATCTTCATCTTCTTCTTCACGCTTGAGCTTCCGAAATCTTCTTCTCCACGTCGTCGTTCGCCACGCTGCCGTCTCTTCACGCTTCTCCGTCTTTGAAAAGTAACCATCGATCCTTCACTCCTTTCGTCTTCGCCGTTCTTGACATTGGGGAAGCCCTATCCCTCTCTCGTTTTTGAAGACATTCAAAATTTGTTTTCACACTCGTTCGAAATCACCATTCAAAATCACCACCGGGGATTAATTTCATCTCTTCACGCCTATCTCTTCGCACTCGATCTATTCATTCTGAAAAATCCGTTGATTGAAGTCCTCCAAACAAACACATTTTGTTCTCTGCCACAAACTGAATTAGAATTTCAAAACCGACCTGAGGTTCTAGAGCAGTGTCGTCGCGACGCTTCAGGAGGCGGCCGAAGCCTATTTGGTTGGTCTGTTTGAGGATACGAATCTTTGTGCTATTCATGCTAAGAGGATAACCATTATGCCTN

>cucumber_newGene_542 cucumber_newGene_542.3

ATTATTTATGAGTGTTCGAATATGAATCTACATTCATAATTTTTATATTTATATCACATTTAAACATAAAGCTCCATTACTAATCTAAAACCGACAACAATAACACATATACTTGTTGGTTTCTCTCTCCTCATCTAATTCAAACAATTCGAATAATTTCATCATACTGTTCTAAGTTTATTCCATATGAGCTAGTAGGGGAACCTAATGAACCTATAGATCATGGTCTCCAACAATTTGAGATTAATTGGTTAAACCCTTTTTAGACCAAGTTAATCAACATTCAATAACTAATGGGTCATTCCACTAAAGTTTTGTAGTTGTACTCCCTTCACTATAGATATATTTGTGTCCATCTGATATAAATATGATCAATAAGTTAATCTTTCATGGATTATTCGTAACCTCGGTTAAAATATTGTTTTATCCCCGAGACTACATCTCGCTTCTTAAGTCTCATTGATCCACTATTAAATAATTGGTTTAAGGTTCAATCTATAAACTGAATCCCTCTTAGGCCGATGAGAGTGCAGGGCTCGTTGTTCAAGACTTGGATTTAGTCCTTAAGGAAATGAACTATCTATCAATCTTATGCCCCAGCTACCTATTTGGTCTTACCTTTGAAATGAGAGGCTTATTGAGCCAATGTTGATGAGCTGCCATCACCTTTGCAGATTTAAAGATAATTTTGTATGAATAGTTTGCTCAAGATTAATTAAGATTAAGTTACCTAGGTCATCAATAATTGAAATAGTCAGTTTTAAACAATAAACAATGTTATAACGTAAAAATGACTATTTAATAGTTCAGTCTTATGTAAACTCTTTACATAGGATGCCTCCACTTTAATGTCTCTACATGAACAATTCAGGATTACATCATTTATACTAATTACAAATCGGGCTGCATTCATAATGTCATCATAATAAGGTGCCAAACCTTTATTCACATACTATAGATTGTTTATAGTATATACTCGAACTTGATCCATGTTGATGTCTCTACATAAAATTCAAGTTTACTCAAGATAGCCTTGAGACATTGGTTTATTGGATTCAAAATTATAGTATTAATTTTACTAACAAAATATCAATAACAACCTTATTGAATAAAATATGGTTTTAAATTACAAACTACGAGTTTTATGACATAAATTCCAACAGTCAGAGGAATGCGTACTTGTCATAATGGATGTGTTTGCATCATCTGTAACAATACATCTATCTTTAGTGCATTGAACATTTAAGTCTTGATCATATAGTTGGCTAAAGCTGATTACATTAGCATTATGACCTTCTACTACATAACATCCTTAAAAGAAGGCAAACCGAGAAAGTTTAAAGTTTCCTTTCCAACAATGTTAGCACAAACATCATAACCAAATGTTACTTTACCTAAGCTAACTGATTTCACATTAGTGATATATATTTTCTCACCAATCATACGACAGAAGCTACCATTGTTGAAGTACCAATCTCCTTTAATAGAGGACCTAAAAAAGAACAACCTTGCATTTGCTCTGAACTTCTTCGACTCTCTACATCTATTTTACATAGCTTTGGTGGCTAGGAGATGTATAGTTCATCATTCTTCCTAAGGACATATGAGGATAACCATGCAATTGATAACAAAATGGTCTTTTGTGATTAGGTTTCCCATAAAAATTATAAATTTACCTCATTCTATGAGTGTGGGCATGATATGATTTTTGCCTTTGATTAAAAGGTTTAACAATCCCATAAGATTCAGGTTGTTAAGCTCGAGCAAATTCATTTTTCTGATTTCCTTTGACTTGTTGGGATTTATTGTTTTGTCTTTTGAGGCATTACCTTCTTAGAAAATTCTTTCAAGAGTATCTATTCGAGGATTAAGCATAGATTGACTTGCACATTTCATCTTTTTTAGCTGTAACATAGTGTAACTCATGCTTAAATATGAAAAAGTTGACATCAATATATGATTATGTGATAGTAAGGTCTCTATCCATTCTTTCTGAACATTTAACACTTTCCAGTCTTCTTGCAATTGATCAAACAACATTTTGTAAGATGGATATGAGGAATCTTTTTTAGATGAATTGCTTGCAAATCCCTAAAATGCTTCATAATCAAGATTCTCATATAACGAAGTGTCATACGAGATGTTACTAACAAGAGCTTTATAACCATGTAAGTATCACTACTATCATCAAAATCCATATCAGACCAAGTATGCACATAACTTTTATTCTGTCTCATCAAAATGTTCAATACTAAGCCTGAAAATGTCTGAAACTCTCACATTCTCTACATTTAAAGGATTTTCTTTCTTTGATAAGCTAGTGGAACCAAATTTCTCATTGTCTTTACAACTTTGAACTTTAAGTGGTTGTGCATAAATACTTCCATAGGCTTTGGATGTTTGGAGCGTGAGTTTTTCAATCTTCTGTCAAGGCATTTCATCATATGACGAAATTGCTAACATAACAAAGCAGTTGATTATGAGCCTCATCTAAGTTTTTGTGAACATTCACTCATATCATCATAGATAGATTGAAGGGCAATTCTCTTATTCTTTTTATCCTGCTTTACATCAAAAGACTATTCAAATGTTAGTACATAGTCAATTACTTTATCCACTTTCATGGATGTTATATTATATCTTCCTTAATCGCTCTAACTTTCATGTCAAACCATTTGGGTAATGAACACAACACTTTTTTTATAAGCTTTTATTCTAACATCTTTTATATAAATGAAAAAGACTTATTAGCTGAATCCAGAAAACAAACATTAAATTCAACAATTGTTTCATCCTCTTGCATTATGAGGTTCTCAAACTTTGTGGACAACAACTACAATCTAGATTGTTTAACTTTGGTTGTTCCTTCATGAGCCATATATAAAATATCCCAAACAAGTTTTTCTGAGGCACATGTATTAATCAATTGATCAACTTCGTTAAATATAATGCTCAATGTTTTAGAGTTTCTCAGAGAAGTGTTGTCTTCAGCAATAGTCTACTCTACCTCAAGTTTAGGAGAAACTTTCTCATCCTCTTATGACAGTTGGAGGAGTCCATCATGTGATAATAGATTTCCAAGATATATTGTTGAGTGACTTCAAAAAGGTTGTCATATTAGCCTCCAATATGCATAGTTAGTTCCATCTAGAACAAGGGCCGGCATATATTAGAACCATATTTTTTGAAGCTATCCATGATCGAAGCACTTGAGATGAAAGTGACCCACTCTGATACCAATTGAAATTTTGTAAAGAGGTTCTTTCACACAGATATAGTTGGCACACAACATGTCCTAACAAGTGCTTGTTTGTTTCTTTTATGTGATATGTGATATGAAAAAATGATTTGTAGATGTGTCATGCAAGGTTATAATAATGCGACTAAGCTGGCATCTAGGTTAGGGTTATGCAATAAAAGGTGCATGTCTTATCTATGTGTTGCTAAATACATAATGATTAATGTTTAACACAAGTTTTCCTTTTCCTCGCCCAAGTGCAAGTGAAAAAAGAGGTTTTTTGGTAAGTCTGGGGTCGAACACATGAAATTTCTACCAAATAAGTTATGACTTTTACTTCTAATTCTAGGCGTTATAAATTTTATACATTATAAAAGTAAAACTATACTAATCTAATTATTTACACTTGAGGATTTTGTAGCAGGGGAATAGAATGAAATACTTATGGAATGGAATACTTATGGAATGTGAACTAACTCGCATCAAGAAAGATGAAGGAAAGTCTTCGCATTGTAGGTGATTTAAGACATGTCAATGTTCTTGATGTGTTAAGAAAGATGAAGGAAAGTCTTTGCATTGTAGGCGATTTAAGACATGTCAATGTTCTTGATGCGATATAGATCATTTAACCATCTTATATTTAAGGAGAGCAACCTCTCGGCGGCTGAACACAAACTTGTTCTCCTTTCGGGAACCAAATGGACACAAATGATTAGAGGCAAAACTCCAGAACAAAGTTTGTTTTCAAACTCTTTTTTGTGCGCGTTTAGCAAGGTATTTACTACTTACTCTCTCAAACAGTTGGCGGCGAACGGTAATCAAGCGATTGAGTTAAGCTCTAGCTAAACATGATGAATCTTATACTTAAAGAATCCTTATTAAGCATATAACATACACTTAAACTACTTAAAACACTTCAACAAGACGAAGAGTAAAAAAATGGTAGAGAGATAGTAGATAAAACATGCATTGTATTGATACTGTAGGCTTCTGAGCCATGAGAATAATACAGTCAAACAATACATTCTCAAATACAAAAATGAAATTAAAGAAATGTGTCGAGTTGAAAAATTCATTGTTATGCATTTCTGGCTCTTTTACAAGTTATGAGAATGGTAGGTGAGCTTCTCTCTCTAAGCTCTCACTCAAAAGTTAATTGGAAAGGATTTGAAAAAAAAACTACTACTACAGATAGTGAAGTACTCTAAAACTCATCATTTCACCAGCCTCCTTAACGAATGGCCTCCTCCCTTCTTGTTCATGGTGGAGCTGGAGTCTTTTATAGACATACTTAGGCAAGACATTATATTAGTTATGATATGCTGCACATTCGTCCTTGCTATACTGCATATCTGTCCTTGCCTATGAATGTCAGTGTAGAAAGTAGTTGTTTCTTGTCATATGAGTTCCAATTACCACTCTTGTCTTCTAGCGAATCATCTTGAGTGATGTGTTGCCATCTCCCCCTACAATGTGAATTGTTGGGCTTCTTGTTTATCCTCGAGTTCTTGCTCAAGAATCGTCTTGCAATTCGCTTATTTTCTTCTTTTCATCATCCTACGTGTAAAAACACTTAAAAGCATAGTTAATTAGGTGTAATGACTTATGTAAGGTACATTCACTTAACATTATAAATTTGCACTAAAAGCTACCAGTCCTTTATCTCGCATTTTGGCTCCATTGTTTTATCTAAAAGGCCACAATAACTTATATTTTTATGAGTTATCAACAAAGAGAGACAATATTAAAAATAACAAAGAGAAAAACAATAGTAACGCACAATAATTGGTAATCCATTTTGGTGGTACAACACCTACATCTGGGAAACAATGTGCTCATGAAAGATAATACACTAGTATAATATAGTTAAAAAATTATATCATTGTACTTATTTGTTCTCTCAAGATTATAGTGACAACCGTAGAGCTAACTCTATGAACACCTAGACTCTTCCTTTTAGATTTAAGACTTATTCTCATTAGAACTTAGACTCCTCTTAAGCGTGTGAAACCCCATCTCGGGTGAAGTTATCTTTCCTCTTCACTTATGAACTCCCACCATGTGTGAAACTTATAGGTTTCACCTAAGTTAATGAAAACCCTATTCTCAATTAGTCGGGTTTAGACTCCTCCTAAAGTAGTGTAGTATGATCAAATTTAATGTAGTCTTAAAAAACCTCTTACAAGAACAATAACCACATATGCAGACGAATGATGCTTCAACTTCTCTCACAATAACAATAAAAAAAAATTCAGAACATATGCGACAACCCTAGCAACGAATGCATCACGAGCTTAAATAACAAAAGGGAGCAAGGAAACAAACCACATAACGAATTGAAACAGCTCTAGAAAAGGCAAAACCAAAACACAAAATAATGTAAATAAGGAAAGAAATATTTTGTAGGATCTGCAACTTTCATGATGATTGTTAGGACAAGAATATTCTGAAACGTATTCTAGGATGACCATACAAATAGCTCGACAACATGCTAGAAAACTTCAAATATATTTAGCCAATTTTAAAGGCATTAACAATAACAAATAATAAAATGCAAATAAAAGGTAAAAATAAAAAAAATGAAAAGAAAATAAATGAAAAAGAGCAACCACTATAATTTCAAAACAACTCTATTTTCATCCTAAGTTAAAAATAAAAAAATATTAATAAAAAAACACATGTTTGCAAATTAATTTGGTATATTGGTATGTGTTATGGTGACCTTCGTTGAAATTGAATTTATTAGAGAAATTACAAAAATAAACAAATTTTTAGGGGTCAGAGGATTTTGGGATTCTTTTTTAAAAATTTGAGTTTTACCACGAAATGGATTGTGAAAGTCCAAAACGCCTTTAAATACAATTGCATACAATTTTTAGTCACTCTTCAACAAACTCTGTTGTTATTTTGGAGAGTGTTTATGTTTTTTTTTCTTTTTTTTTCTTTTTTATTATTATAGATAATGCTCTTAATCTTTAGAGTTTTCTTGGTGAAACTTTCATG

>cucumber_newGene_544 cucumber_newGene_544.1

ATTCACTCATACCCTCTGTAGGGATAGTAGATAGGTTGTTCCCTTAAGGATTGAATCCAAGTCTTGAACAAAGGGTTCCACCCTCTCATTGGCTCGAGAGGGATTTAGTGTATAGGTTGGACCGAAAGCCAATTGTTAAAGAGTGGATCAGTTGGACTTAAGGATCAATATGTAGTCTCAAGGGTAAAATAATACTTTGACCCAACCGAGATTATGAACAATATGTGAAGGATTAACTTACTTATCATGGTTATATCATATGGATGCAAGTATGTCTATAGTGAGGGAAGTGCAACTACGGAACTTTAGTGGAATGACCTGTTAGTTCACGAAGGTTGATTAACTCGGCCTAAAAGAGTTTAGCCAAATAATCTCAGATCGTTGGAGCCCATGATCTATATGTTCATTAGGTTCTTTTGCTAGCTCACAAGGAATCAACTTAGAACAATATGATGAAATAATTTGAGTTGTTCGAATTAGGTAAAGAGGGAGTAACCGACGAATAAATTTGATATAGCTGTTGGTTATAAGCTTCTAATAGAGCGTCATATCTAAATGTTGTTGGTCAAAATTAGTTAAGATGGACAAAATGGTAAAAAGTCAAAATGTTGACTGTGGACTTGAAAATGTCAAACTTTGAATTTGATTTAAAATGTTAAATTACCATACTGTCTTTAGTCTAATTTAAATAATAACAATTGTTGAGATTTGACAAACTTATCACTTGCATGCGGCATGAAAGTGGCTTACTGCATGCAAGACACCTACCCCACTTAGCTAAGTAGTTTGCTTGTAAAAGTTTCTTTTGCAAAGATGATAGGACTCTTGTGTTTGAGAAATGCAAGAATTCATTTTGTAATTGAGAATTTCAAAATAAACCAAGGACATTTTTTTTCTCTCAACAACAAAAAGATCTAATAGTTAGGTTCCTCTAAAAGGATATCATTTCTCTCTATTTTTTCTTCCACCATTCAAGTCCCACAACTCGGTTCCAAGTCCAGAGAATAGCAGATCAACTCTAGGGATAGTCTCAGCTTCGAGTTCGTGAGGAGATTTTGAAGAGATTACAAGAAAAGAAAAGCATCATAGGTGAGTTTTCTTTTAACCCTATCATTGTTTCAATTAGGGTTGTTATGCATGTTGATTAATAAATTTGTTTAGTTGGAATTAGAGTAAAGTTTGATCCTTATTCCACTGTGCATGTCTACCGTTTCCATCAACCTCAAGCTTCACCTTCACATCAAAATGCATGTGAACATGATTAAGATACTTGGATTATTTCTTAAACCGTTTACTATAAACATGTCACATAGTTGTCAAAAATTCAAAGCTAGCCTCCATGACCATCTCGCCTAATTCCTTAGCCAAACTACCTAACCCAACTTAGAAAAATCATAACTTAAAATACATAACTCTTTTGACAAACCGTTCTTCAACAAAGTTGCTCTACAATTTCTTAACTTCCTCACCACAAAATTTTAGAGAAAAAAAGCAAAAAAGATGAGTTATGTAGCCTTCTGGGAGTGCATTTTGCTCAGATTCACTCGAGAACTGACCTTCTCACCTTATTTTATCTAAAATGACAACTTTCATGTATCTAAAATGTCCATCAGCCTTTATTAACAAACTAGACACAATTTCTTAACTTTCAAAATTAATTTGAGTGAAAATGCCTGAGTGAGTTGTGAGAAACTCCTTGCTAAAGTTAGCTAAAACTCCTAACCTCTATTTCTAACCTTTATAGCCATTTCGCGAAGCCAACCCAAAATGCATAGACACATCTCTAAGATGCTAGAGGTTATTTCCTTCCCTTGGCTGCCCAATGCTAACCTCTAAACGCCAAATGTGCTATTCTTTGATATCTAGCCAATATTTCCTGAATTTCCTTAACTTTGACTTAAGCCTAAATTTTAACCTTTCCAAAGAATTTAAGATCCTTAATCCCTTAAATCTCATTTTCACTTATCCTTAATAAATTCCAAACATCCAAAAATTTAGAATTTCACAAACAAGGTTGCTAGATAGAAGGAGACTTACAACCATAGTAGCGGGTCCAAGTTGTTTTTACAATGAGAACACGGGCTTGCTGAACAATGAGGTCAGCTAGTCGACCGTGTGGAGTTGTTTAGAGAAACACACGTTGGGAGTGGCTAGTTCATTTTCAAGCGGCTGCAGATGTGCATAATCAAATGGTGGAACTCCAGTCCCAGCCCACATTAGATGATTCTCAACCACTCTCTTTCGGACGAGATATGCAAGACCGTTTTGGGTAGACGACCAAGCTACTACAAAGGTTTTGGTTGGGATCCCAAGCCCAAGTCCCGCAAGGCTAGTGCTTAACAAACACATTTGTAACATAACAATTGTTACAATTTTTATGTGATAAATTTGTACAGATTTGTTCCTATAACATACAGATTTGTTATGTAGTTCCCATAACATACAGATTTGTTCTTGTTCCTTAGGCCGCTAGAATTTGTACGCTCCTTTCAACGCATAAGCCAACTTATTATCACCATGTAGTCCATACACCCTCATGGTGGCCTTGACTCTTTATGTGCACATAAAAGTTAGCATCATCTCAAGGAATCAACTAATGGTTTGAAAATCATTTCATAGCATTGAAATCGTAATCATCAAATCAAGCTTTAACATTATAAATCATG

>cucumber_newGene_545 cucumber_newGene_545.1

GAACTCTTCTCACTGTAGATATATTTTTGTGTCCACAGTATAGACAAATAATAGTAAGTTAGTCATTTACGAGTGTCCGTAACACCAACTAGGTCAATTTACCGTTTTACCCCTAGGTTACCTCTAATCTTAAATACCAATGCACCTTTAATGAACATCTTATCTGTGATCCTACCAATAAACAAAAACTCCTCTCGTGCCATAAAGAGGGTAGGACCTTTTGTTCAAGACCTGGAGACACCATTTAAGAGAACACTCATCTACTTACCCTAAAGTAGGGAAAGAGTGAATTCCATCTTATGTAATTACGTTCCCAGCTCCCCACTTGGTCTTGTCCCAAAAATGATAAGCTTATTGAGTTGACGATCTAACCACTCTCACCTGTAAAAATCAAAGGACAATCCCTTACGAATAGGAGTTCATAATACACTTAGGATTAAGACTAAGTTACCTAGGTCATCCTAGTAAAATAAAATCCTAACTAGTTAAAGGAGTTAATTTAGTGGTTACTATTTTGTGGTTCGGTCTTATGCAAACTCATTGCATAGGATACCTTCATTTGCATGTCAACTATACGAACGTGTTGGATCATTGCAGGCTCATTGGTCCATTCAGACCCCTCACCTCTTGGTAGAGATGAAGTTCTTTTATAGGCATTTTTTCGACAGAAAATTTCCATCTAGTGAATCTTTCTCATGTTCTGATTATTTCAGCCTTGATAGCAGACTTATCACGTCACATTCATCTTCAACTACCATTCTTGTCATCTAGTGAATCTTTCTCATGTAATGATGTGGTTCCTCGCCTAGATGTGTTAGTCGCCAAATGAGTTCCTATCGTGTAGCCTTTACGTGAGAAATTATACGTTTTGATTCTCTTCTTGTGTGTTTTGACCTCAATATTCTGATAAAAGAGGCTATAATAACATGTATTTATACACATTATCACTATCTTCATGAGAACCACATTTGGCATAGAAATAAGCTACACGATGGAAATGTGGAGCATAGGGCTCCGCCGGTGGTAATGAATGTGCATAAAATCTTGGAACAAGTAGATTTGTAGGAGTTTCCAGTTATAAGCAAACATCTTTCATTAAAAGATAAGAAAAGAAAGAGAGCTCTTAATTGGACTAAGAGAAGTATTTTCTTCGAACTTCCTTACTAGTCGATACTATTATTACATCACAAACTTGTTGTAATGCATACTGAAAAGAATGTTTGTGACAACTTGGTTGATACATTATTGAATATTGAAGAAAAAACAAAGGATACTGCAAATGCTCGATTAGACCTACCATATTGGAAAATAAGAAAGGATTTACACGTAATAGAATTTAGTAACAGATTGGTGAAGCTACATGCGAGTTACACGTTGACTAGCAGCGAGTGAGTTGTGTTTTGCAAGTACTTGAAATTAATTAAGTTTTTTGATGTATTCATTTCTAATATATCACGTTGTGTGAATGACAGAGATGGAAAAATGTCAAGTCTCAAAACACACGACTGTCATGTTTTGCTACATTGACTTCCATTTATAGGTGTTCGATCATACTTACCAAACAATGTGTACATTGTTGTTACTAAACTGTGTAGTTTTTTTCGTGACTTATGTGCAAGAACAAAAGAGTAAGCGATTTGGACTAATTGCAAGCAGATATCATAATCATACTTTGCAAATTGGAAAGAATATTCCCACCTACCTTCTTCGACGTAATGATGTATCTTGCCCTTCACCTACCATATGAAATCAAGGTTGCTGGTCTGGTTTCTTACAACTAAATGTATCTCGTTGAAAGAAGTCTCCACACATTAAAATATGTTGTTAGGATTAAAGCATGTTCTGAGGGGTCTATTGCAGAAGCATATCTGATGAATGAATCAGAGACATTTTGTTCAAGGTATTTAAGTGGGATTGAGACTTGATTCACTAGAGATGAACGAAATGATGATAGAATTCCAAATGACGAGGTGATTGGTGAGCTCGAAGTGTTAAAAAAACAGGTATATAGTTTCACACCCCCTCCTAGGTCACCTGCTTTGGATCCAAAAGGTGGGTGATGTCAACTGAAATTATGTACCTTTGAAAAATTTTCAGCTGACTCTTAACATGAAAACATAAAACATTTGCTAGCAAAAGTTCTTAAAATTAGAAATACTTTAAAAACTGTAGTGTAACCCATTTGGAAATAACTTTTGTAGATACCAAGTAAAGTATATAAGACATAGAATATCAATTTCATAAAACATAATCATACTTTTAATAAATCCTTCATTTCTTAATTGAAAACTAAATTGGCTTGGTAGTAGTAAGTTTAGCTCACGGAGATTCACAATATGTACCTGGAAAGAAGGAAAACATTTTGAAAGAGTATGTCGAAAGACTTAGTGAGCAACAGTTTAATAAAACAAGTTTTCACAAACAATTTAACATAAAAATGAAGCTTAAATAACATTTATCAATAAGCAGTCACTTCATAACATGAAACTTGAAACATTGAACTTTTGCATAGCTTTTAGAAGAAAATGATCAATAGCCCAAGATAGTATTAAACCTCTTTGTTCTAAGACGAAAAAGTCGATAGAAAACAACTTTCACTGTAATCCTCCTCCTTAAGACATAATAAATATACTCGTCCTGATGGCCCATAAACTATAGGCTCGTCAACTCTACCATCAGTGTGAAGTTATACAATAGGTCTCATGGAAAATCCTTAAACATTTCCTTTGCTTTCATAATATGTCAATAAATCATGGAAACATGGCATAGCCTTGAGAAATCAGTTCAATAAACATTTATTAACTATATCTTTCAACAATAACTTGGAAATCATGCTTTATTAATCACTTTCAAATCATTGTGTCACTCACAAGCTATTGCTTATGTACGTTGAAACCATATTTAATAAGTAGCTTTCAAATTAATGTGTTACTCACAGTACTTAAAAGAATCATTGGTCGGTATATTCTTCTAAAGCGTCCTTGATTTGAAAAACAATGTAGATTTAATCCTTTAGATATAAAAATATCCAAAACATTTCCTTAAAATTTCTAAAAATATCAAAAGAACCCAAAAATGGCCCTTCGGTGCTCATTGACGTGGTGGCTAGGGTTTGGCGCATGAATGGAATAGAGGCTTTGTTGTGTGCTAACCTTACTGAGGTGGGTCACACGCCACGTGCTAACCTCATAGTCAGGGGAGCCACACACGTTCTAGTCGTGACCTAGCGTGTCCATTGCATGCCTTGCACGCGTTGTGGTGCTTGCGTAGCCCTACACCCTAACCACTTCTTGCAAGGTCATGTGTGTAGCCTGAGTGCATCCCTGCCAAAATAACCTCTATGAAATAAAATCATGCCCATCTCTCCTAGACATACATGACATCGACCATTACCCATAACTTTATTTTTATATAGAAATCATGACCCAAATTTGAAAAATCATAACTTAAAATTCACAACTCTTTTAGAAACAATTTCTTCTATAAAGTTAATTAGATGAGCTTTGTAGCTCTTGTGGAGTCCACCTTGCTCGTATTCACCTGAAAACTGACCTTCTCTTCTTTGCTTGGTCAAAACGCCTAGCTTCTCGCCTCCAATTTTAATAGAACCCTTCCAATATAAGTCCAAAACGTCCAACTAACCTCTATGGCCACAAGCCTTTCTTGCCCAATGTCATCTTGCTTAACCAACTCGCCTATTAACCTCTTAATCCATCATCTCGTGTAGCAAATTTTGCTTGCATATTACACTTGAATGCTCATCAACTTCCTTAGAGGTTACTTATAAGCACTTCGGCCACCCACCTAACCTCTAAACGCTTTGGTCCTTCCTTTGACATTTAGCCAATTTTCCAACTTTTCGCTTAATATTTAAACATGAAACCTTAATACTTTTCCAAGAATTTTCACTTTCTTTTCTTTCAAGTCTCATTTTCTCAAGGCTTTAACAAGTTTACAAAAGTCTTAGAAATTTGGGGTTTCACATGTAGCGTACATGAATTTGATAACGTCAAGGACTAACTTCTTTGAGAATTTTATATATAATCATTTACACGTGGATTTCAAGTTATAGAAATGCGTGAGAGAGAAAATCTTTGTCATGATTTCTTCTCACTTGCTATGGGAACTTCACTCTACGTTCGATCTTATAGTAGATGCATTGTTGGTGGGGTGAGATTTCACACGATAAAGTGTGATTTCTAACGCACTACACAACACAGGGGAGGCATGGTAATTAGTGAAAGTAGTGGCAGTGGAAGTGGAAGTGGCGACAACAATTGCTATAGTATTTTAGATGAAGTGTTGGACGTTCAATATCTGATGGAGCAACGTGTTTGGCTATTTAAGTGTAGGTGGTTTGACATCGACAACAACAAAAGTCATAGGTCACACGTGGAATTATGATACAAATCAATCAACACTTCCATTTTTGGTTCGTCGAGGAACTGGTCATTCTCGCAACGCAGGGACATCAAGTATTTTACCTAGAAGACCCAAAAAATGGTACCAATTGGAAATTTTTTCAAGTGGTCCAAAATAAACGTATTAGGGACGTGCCCAAAGTAGAGGATGTTGAGAATGAGCAACCAAATGTACTGGAAGTCATTGTCGGACATCATGTGGATGAACATATTGAGGATAACACTCTGTGCAGACCTGATATTGATTCTGCAGTGGTTGGAAGAACGGTTGTGCGTCATGTCGTTAACGACTTCATAGACGATGACGATAAACAATTGTCACAAAAAAGCGGATCAAGTGAAGAAGAATAATAATAACGAATCATGTATCCATATATTTCACTATTTTATATATTTAGTTTAAGGCACTCGTATTTGTTTAATTAATTTTAGTTTTTATGTTCACAGGTACTATGTTATCATTCCCAAGCAGTTTTAAGGAGACTGATGCTTTGTTTCCTGAGTTCGACGACAAGTTCAATAATGCAGAAGGATCGTTCTTAGAGGGAGACACTTCAAATTAGTCTCGTTGGATAGGTACGTTCATAAGCATGGCAAGATTTTGATTACCATAGCCTCAGAAGTGGAGAAACCAATATCTCTACATGGTGTTCGGTTCAGCAACACCATTGATGTGTGTGAGAGCGCTATTTCATGCTTGATTTCGACGATCAACCACTTACTAGATTTTTTAAGAATCAAATGCTCACATCTTTCAAGGAGTTTAGGGGAGATTGTCATAGATATTTCAAAAAGTACAGTGACCCTGAACAGGCACGTGCCAACCCATTGCACAGATTGGTGGGACGTCTAGAAGATTGGCATTTCTTGTGCGATCATTATTTGAGCCAAGCATTTAAGATGAGTTTACATATTGCAAGGTTACATTACTTCATTTAAGTTTTTGTATGCATGTATAACTTTAAAAAATGTTTGCATGCAAGAGCAATCAAGGGCGAACAATATCACACCCTGCCTCGGGCATTCGCTTGCTCGTCCCAAGACGTGGCATGATGTCAGCCAAAGTCGTTTGCCTCCAAAACAACCTCAATTGAAACATGTCTTGAAAACATGAAACATTCAACAACTTGAAGTACCTAAAATCGTAGTGTGAACTCCTTGAAATTCTTTTTATTGAAACCAAGTGAAAAATATATCAAACGTAAAAG

>cucumber_newGene_546 cucumber_newGene_546.2

AAAAAGAAGAAAAAATAAGAACAGTGTGTTCCCAATGCGTCGTGTCCTACTTTTTTTTAAAATTAATATATCATCGTGTCTGTGCTGTGTCGTATTTATGTCGCATGTTAGTGCCTCTGCTTCTCAGAACCCAACAATCTGAATCTTGTATTGAGCAAAAGTGTAGCAGATATTCAATGTGAAGTTGCCATTGATTTAAATTTTTTGTTGCTTCCTCTAATTGTCTGTTTCCCTTAATGGGATAGACTTCTAATCTTTGAATTTTTACCTTACAATTTACATTGATGTTCGTGAAGCTGGGTTGATCCCCTCTCCCCCCAAAATAATTCTAGTTTAGAAAAACTAAAGTCTAGTTCGTTGGATTAGTGTATGTTTTTAAACTAAAGACATAATGCAAGCATAAATAGCCTTATTTCTCCTGTTTGAATGAAAGGAAAGTCACGAAAAGCTGGTAAACCTGCAGAAGGAAAAAGGTTGGTGCTTCGTGTTAAATGTGGATTACAATGTATTAAACTCTCAAAATGGACTCTGAACAAAGTGTGAAACTTCTAACCCAAAACTTCTCGTTTTCTATGTTTAGTAAGTTAGTAACCATAACTGGAATCATTTTGGGGAAGAACTCCATTTCTTTTCCTCAATTTTCTGAGTAGATTGTCCATGTCAACCCGTGTCATTGTTCTAGTAGGTTGCTACTTTTGTGGGGCACATTGTCTCTCAAGAATTAATGAACCATATGGAAGTTTCGGTGTTGATATAGCTTCCAGAAATCTTGTAGGAAGGGGTTTTAATGATCTATATGGAAATTTCAATGTTAATGTATGTATCTGCCTGTATCTTTCAGGTTAAAAGGTTTAAAGTTCTTTAATATTAAATTTGAAGAAGTCAAACAGTTTATTCCTTATTTATAGCTACCATGTAAATTTAATTATAATTCTAAAATAATGTACACATGTCTACAATTGGCTTTAATACAAGTCCTAAATTCTATTAGAAGTCTAAAAACAAGCCCATTGTGATCGCACTACATCCATTAAGTTCTACAATGAGATTGAGGTAATTTCCTTGAGCTTTCGTAATGGAATAAATTGATAACGTAACTATAGGGACTGTAAATTTCAGTTAAACGTGTCAACTGCCAAAGGAACTTCTGTAATGAATTTATGTGTGCTATGGAGGTTTTCACATGCTCAAGCACTTTACAACATTTCAAACTGTTGACCATATTTGTGTCACTGTTGATATAGTTTTGGTATATAGAACTTGAGGACGTAATTATACCACTTACGGATAATTTTGTTCAAGTTAGGTCGTTATTGTATTTTTGGTATTGTAATTGGAAAGAGATGGAAAGATTTGTGAGACAGTTTTGTTACATTGTGGTGGCTGGATTAGAGTTATGATTTATATATTTTTTTAGAGTTGAAATGTTAAAGCTATAGAAATTGTTTTGAATTGCGAGTTTGTATTAAGGGTTTTTTTTTCTGAGTATATCTTATTCCCTTACTCTTTCATCTTGTTTTTTAGTTAGTGGTCAATAAATTAGTGTTCCGTCTCTGAACTTTAATATGACTGTTGACCAAAATTATTTGATCAAACTTTTTAAAGGGTTGACATTGTGTTAACTGTTATTTCTTCTGAGATTATCAATTTTATCTTATCTCTTTATCCAGTAAACTAGTAGTAAGTCCCTTCGGTGTCCTTATTATCTGCCTAAGATTGTGCTGTCTATTTCATGTTTTTTCATTTGTTCTGATGGAAATCCTAAGCTGTGTTCTTGTTAGACTCTTTGAAAGCTATAGCACAGAAAAATATAAGAAGATTCTCGAAACTCCATTATCTTAAATTCCAAATCAGTATCCCATCATGTCAAGGTTGAAGTTTATATTTTTTGTCATATCATTTGTGTCAGTAAAGATGAAGATATTTCTCTTGTTCACTGTTTAGCAGTTTGACTTGAAATTCTCGTTCAACAAGTTTATAATATCATGTACGTGCAAAGGAAATTGCTTGGAAAATTGCACATATTGCGGCTATGTCTTATCAGATCCTGTTCTGCGTGTAGTACATTTGTTCCTTTACATAAACGTACCCCCTGGTAGTGAACAGATTACAGTGTGAATTGTTTTTTTGATCTTATACGTACATAGAAGAGAATAGGACTTTTTACAAAATATATATCTATATCTATTTATCTATAAATGTGTATATTCTCTAATCCAGCTTGTTTGAATTTTTTTATGCATTTCTACAGAACTATCATTTACATCGGTCAGGTAGAAGCGCTTGAAACCTTTCCTGAACATCTGTCAAGCTAGATTGTTAAAACGGCATAGAAGGAATGTGAACTTACCCTGACGTTCGACATCAGCATGGATGCAAAAAGCTTTACCTGTAGTTCTTCAGATGGATCGAGTTCTACAAGCTTACAATATTTAGCTTCTCTGGAGAATAAAGAGTCTTTCCATGGAGAAAGAAATATGAAGTTTTAATTCTATAATATTGGTTGCGCATGGCATGGCAATCGGTACAGACACGCAAGTAAAAGAAATTGTGTAAGTTATTGGCTTTTGCATTTCTTTAATTTCTTTGTTTTCTTCTTTTGTCACCTTCAGATTTTCCTCTATATGTAGAAGTGAATTACCGTTAGTGCAAAAGCTTCTCATGGAAAGTTTCTGGAAGTTTCTTCATGTGCATTGTTTTCCATTCTTACTGGTTTTTGTGCCATATTGGGCTCCAACACGGCCCATCCCTGCCTCTTGCGCTTCTGCTTAATTATTATATCTAAGAATCGAACAATTTTCGAAATCAATGTTTTGAGCTCTTACAGCATAATCATTTTCTAGGAAGTTATTGTCTTCTGAGGATTAAAGAATCTATATATTTTCAATCTTTCATTTTCCGTTCTCCCTGCGGTTTGGTTTATAAGAGTCACCGAGTAAGATTCATTTTTCTAATAGTTTATTTGATGCACTGTTTCCGAGTAAGAATCTAACAGTGACATCATTTGTTGCCTATATTTGTAGTTTTTGTCGCAGGGTGCTAGTGTTGGAACTACATAGACCGCTAAAATTGTCAACCGTTTTTATTAGCATAGAGGGTCCCTCATTTACTAATTGTGAAATTGACTCTTTTGTTTCCCTTCAAGAGTCTTGCCACTATCTTAAGTAAATCTGCTTATTCAAATTCTTCTTAACTATTGAGAAATTTTGGTTTTCTCGGTCTATTTATCCTTGCCTTTGTGATTTGAACATCAATTTTTACTGCCTGAGATTACTTCAAATTTTAATGAGTTTCTCCCAGAGCAAGCGTGTGAGATAATCATGCAGAGGAAAATATAAGTTTCTTGCACATTTATATTCTGCTTTTGTTGTGCTAGTTTAATTTAGATGATGTGGCCTGTGAAAGCATTCAACATTGGGTGTTATTATCAATACTTTGACGAACTAATAGTGGATGTTGTATTTAGAATTCATGTCTAGGTACTTCTAATCTTTACCTTACACCAGATGAACACGGATGAACTACTCAGTTCACAGAAGAAAGAGCTATGTGGGGGCATTTCTAAGTTGTGCTTAGGTGTTCGTGCTGTAAGGACCCACTGGCTATCCTATCGAGCGACAGGAAGCGATGGGCTGAAAGGAACTTGAAATAAATATTTATATGAATTCCAAGCACTTAGCTTAGCGATGGAAAATCGTTGCTTTGGAATCACAAAACCTTTTCTTGTCATCTTCGTGATTTTTAAATCTCTTTTTGGGCCATCATGTCAGGTAGTTGGGTAGGGGAAGGCAGGCCATAATGCTGTTGATGATCCTTTTGTCGGCGGAAAACATGCATGGAACGAATTACGTGTCGATATGAGGCAAATTAGCTTCATCGAAATGGAAAGAGATGAAAAGAGATTGGTAAGCAGCAGAAATTAATCGAGCTAACAGGTGAGAGAGCCTCGAATCTGCCATGAATTGAAAGCTACGTTTCAATTTGTATGTGTCCAGAGAAATGGCTGTATGAAACCCATGATTTTGTGCCCCTTCCAGTGGGAAACTGTGAAACTCGACCTCCAAAGCCTTCCTTTTCGACTGTCAATGGAGGGAATAGTAGCTACCAATCATATATCTTACACTAACTTTTGTTTTCTTTTTTCTTTCTTTAATTAAATTATACTGATTCATTAGGAAAGTTTACGTCCAAAAGACGAACAAACCCTCTCATTTTATAAGATTCATAAGTCACCTTTTTTTTATCTATTGAATTTCAACATGTACTCAAAAGGACAACATTGAAGGAGCACATTAAAGATTCATAAAAAAAAAATGGAAGAAACCTCCCGACTTTTATAAGATTTATAAGTTACTCTTGTTGTACATTTGTTTTGAG

>cucumber_newGene_549 cucumber_newGene_549.2

GCAGAAACACATATCAGTCAAATGCTCCCGAAGGTGCAAAATAAATTGGTGATCCCGTGGGACACCTACAAGGCACACATCCCAATAAAAAGCTAACAATTTTCCCCTCATTCCATTCAACCCATCTTTCAGTTACTTCAAATCACAAAGTCCTTTCTTTCAACATCCTAACAGTCAACTTATCTCACTTTAGTCATATGTCTGTCAATAATATACTAATACGTCAGTCAATCAAACTATACAGTCAATACACCCCTCAGTTCACCAGCACACAGTCATACTTTAGCGTAACATACACCCAATCTAAACGACAATCACAAGATCGCATCTGCATCTCATACAAGCATAATCTACAATTTTCGTCCTGTCCACATACACACACTTATATATATATTCCATGACAATCACAATATGCATTCAACACTAAGTCTACTTCAATCCATATGTATATATATATTTTCAGTCAATCCACAGTGTACATTCAGCACCGAATCTACACCCATACGTGTATAAACATAAGTATCATCTCAACATCTTTACTCAGTCAAAGATAATCATTCAGACATACAATTCACAATCATAAGTGAGTCCAGTAGAAAATCCCTTACCTGGATCTTTATGTAAAATTCCTTAGTCGAACTAACTTCAACAAACAAACCCCAAAGAGTAAAGAATCGAACACCAACAAAGCTTAAGGCGTAACTCAAGGACACTAACTCCAAATCACCGATGTCGCCTTCAAGAACAAAGATCAATTCAACCAATTAAACAACATCACATCTTAATGTATTTACAAATGTAAGACCTTATTACCGAACAATCTCATCCTTACCAATATCTTGTAACAAGACTTTGACAATCGACGTTAAATAGAAGTTGCCGCAGGATCTACACAAAAGACATCGTGCAAAACACTTTTGCACGCGAACTGGTCCGCGACGGTGGGTATCGACGAAGGCAGAACGGTTCGGACGTTCAACAGCAATTACGCGCGGACGGGAGTAGCCCGGCAACGTAGCCGAATTCGTGCGATGCGGCGTGACCTGCGTCGGCGAGGCTGTTCGTGACTGAGGCGTGCGACTTGCCTCGCGGACGGAGATAGAGACGGCGGAGACGGTTGCAGAACCGTGGCTTCGACAGAGACAGAACCGGCTGAGACGGCTGGGCAGCGGCGGAAGAACAACGGCGCTGCAGATCTGGACTGGCAGCGG

>cucumber_newGene_551 cucumber_newGene_551.1

GGAAACTTATACAGTAGATTGATGTTCTCAAAAAGTAACAAAATAGTTCTTGCTTCTTTTAATCCTGGGGGTCATTGGACTCTGCTGGCCATAAATGCATATGATGATATAGTATTTTACCTCGACTCACTAAGGACAACATTGAAGTCAACTACAAGAGCAATAGCGATGTTTCAGTCACAAAAGAACATAAAAAAAGACAGAAAATAAACTTTATGGTGAATAGTAAAGGCAAGTACAAACATTTAAAGTCATTATATTTTGTAGACTCTAGCAATTAAGATTACTTCGTTATTCAAATTTGTTGATTTTATATAATGTCCTCTACAAGTCGGGAGTACTGAGTGTGGATACTGTATCATGAGGTATATGAAAGAAATTGTGACTAGGGGAAGCATTGTCATATCCGATGCGGTATGTTATTATATCAAACAATTTCTTTTGT

>cucumber_newGene_555 cucumber_newGene_555.2

CAACCATCTTCACACACACAACTTTTTTATAAACTCAAACAAACCCTATTTCCCTCTCTCTCTTTCTCTAACACTTTTCAAATCTTCAAAGAAAGAAAATTCCAATTTTGATCAACGAACCGGCCCGCAAGCAGAGGCAGAGCATATCCGGCCCCCATGGAGGACTACAACAACAACAATGATGATGATCAACAACAGATTATAAAGATAGAAGATACTAGCACTATCACTGATGGCGGCGGCGGAGGCGACGGTGGTGGTCGTGGTGGTGGTGATGGTGGGGTTTTCTCATTTTCCGATAACATAATACCAAATGGATTGTTTGATTTTTGTGATGGTGATAAATGCTCTGTTGGGTTTATGGAGTTGCTTGGTCTTAATTACAATTCTGAATTTACTGAGGCTTTTAATAATCCTCCAGCCACCCCCAATTGTTCGTCCTCCGTCTCCTCCGCCTCTAGCGACGCTCTTAACGACGACGAACCACCACCACCACCACCACAACAACAACAACAACAACACAGCCCCACTAAACAATTGAAAGGGATAAAAAAGAGGAAGGAGAAGGAGAAGGAGAAGAAAGCAAGATTCGCATTCATGACAAAGAGTGAAGTTGATCATCTAGAAGATGGTTATCGATGGAGAAAGTACGGCCAAAAAGCTGTCAAAAACAGCCCTTTTCCTAGTAGAAGTTATTACCGTTGCACGAGTGCAGCATGTAATGTAAAGAAGAGAGTTGAACGATCTTTTGCTGATCCAACTGTAGTGGTGACCACCTATGAAGGCCAACACACTCACCCAAGCCCTATCCTTAGCCGGTCCGCCCTAGCCGTCGCCATTCCACCTCCCTCTTTCATCCCTGGGGCGGGCGGAGAGTGTGTTGGGGGCGTTGTAGCCATGCCATGGCTCAAACCTAGCAACAATGATGCTCATGGTTCGATGGTAACACGGTTCCAGCAATGTCTCATCAATACTTTCAAAACTCGACCTACATCACTGCCCAAAATGTAGCCGCCAACTATAACCGAAACAACCACATCGGAGCAGCGAACGCGGCCGGAATCCTCCAGGAGAAACGGTTTTGTAACCCAAATTCTAGTTTTCTTGTAGACCATGGCCTGCTTCAAGATGTTGTTCCTCCCCACATGCTGAAACAGGAGTAAAAATATACTATATATTCTCATATATTACATTATATAGACATATAGATATAAATATATACTATATATATATATATATTAGTTTAAGTTGGAAAAGAAAACTAAATTGGTGTTTTTGTTTGAATTTTAGCCTAGAGAACTGACTTGTGTTGTTCATAGAAGATGTTAGAGGGAACAAACAAAAATTATGAAATTGAAATTTGTTGCTGACAAAGTTTAATTTGGTGATCTTTTCCCTCTCTTGAGGAAAAAAGAGATCGATTTTCCGTATGTTGAAACTTTG

>cucumber_newGene_556 cucumber_newGene_556.1

AGAAGAATGAGTTAGAAGAGGTAGCAGTGGTTGAACATTAAAGAGGGCATTGAATAATAATGAGCTAAACAGAAATAGTAATGAAGCAGCTGGGATAGGCCATGAGCCATAGCTTTGCCCTTAAGGATAATAAACTACCAAAATAAAAAAGGCCATTTTTGCATTGAATAGATGATCAGATGATCAATAGCTCAGATCGAAGTTGAACGAAAATTGCAGCAATGGCGGTAGCAGCAGCAGCAGCAGCAGCACCTCAAGAACAGATGATGTCCTACTATGATCATGTCCAGAAGCGTAGGGAAGATAAGGGCTGCCTCTATGCTTTTTTATTTACTTTGTGTTGCTGTTGTTGCTGCTACGAAACTTGTGAGTGTTGTGTGGATGGCGTATGTTGCTGCTGCTGATCTTAATGCATATACGTATGACCCTTTCTAACGCAGCTTAGTTCAAATCAAACAAAGTTCTTAATTTGAATTAGCCACGCATCGGTATGTTTTGTAAAATTTGTTAGATATTTTTTGGTACAATTACTTTCTAGATAGAAGATTGAACCTTCTATTTGAAAAGGAATAATGATAAGAACATGATTTTTTTTAA

>cucumber_newGene_557 cucumber_newGene_557.1

TTTTTTTTTTTTCTTTTTTTACTTTTGGTTTTCCATTTCCTTTATGGGTTTTTGTATTGTATTGTATAATTTTAAAGTGAGAACAAAAAGAAAAGAAAAAAAAAGAGAGAGAGATACCTACCTGAGAGGAAATTCTATATTTGGTTTGCACCCTCCTTGTGAATTGCAATCTTTCAAACTCTTCTTCACATCCCAGGTGTGATTTTATACAACTGAAAGCAGCTTGGCTTGAGCTTAACTACATTTTTTGTTTGTTTCTCTATTAAAAAAGGTGTGTTGATCATATTGTAAGGCAACTTTACTTGTTCAGTTCTCTAGTTCTTGCTATAATATTGAACAATTGGTTTGTGAAATTTGAGATAGAATTTTTTTATTCCCAGTTCCTAAGCTTTCAGTCTTCTGCATAATCAAAGGAGCTATTAGTTTGTTCTTATTTCACCTCTTATGAACAATATTGAGTTCTGAATCTTAAACTGCTTATTCTGGTTTACAATCTTTGAAAATTTTCCATTCCTAGCCGAGTGCATTTAATGTCGGAGGAACGATTAAGGATGTTTGATAACTGGGGGTTATCGGGCTAGGCAAGCCATCTAATTTACTCCTTCATCTTTTAATTCAAACAGTTAACAGAGAGTGTATTTATGCTATTTATTAACGGTCATTGCATCAGTACACCTAAAAACCCAGTGTTGTCTACTCGAAAGGCGACTTTAAGACTTTGTAGCTTTCGCTTTTACATTATACTTTGATACAGATTTCTCATGTCTGATTGAAGCATTTAAGATAGATGCACACTAGTCATTTCGGCATCTTTTTCTGCTACTTGATCTCACGCCTTTTCTGATATCTGCTATAGAAAGTGCTTGTTTCGTGAGATTGTGTACTTGCTTGAGACGGTATACAAATCAAGTTCTGCTATGGGATATATTATTTGGTTATAGTACAGCTCCTGAGTTCTCCCTTTGTGGCATTTGAGTTCTTTGACTATTGTTTATATTTTACAAGCATCCCTTTGAAGCTTGCATTTGACATCAATGCTGAGAAGAACAAGAACCTTTGAAGATGGTCTCTATTTTCTCATATGCATGTTTTTATGAGCTTTACATTGATAATGTACACTTTTATGACTTTGTTAGTTTGTTCCGGAAGTCGAACTCATTCTTTCGACATTTATTTCCGCTGTTCCTCTCCTATGTATGTTTGCTTGTTGTCCGCTATTTTTGCTTATGCTTGAGGAAACTTTAAGATATGACAGTAAGCAAGAATGTATCTTTTCTCGAGTCACCTTTGTTAGCTCTGGTAGAACCGTAGATCATTTATACACACAGTGACAGTCGGACCATTGAGCTCATTATGTTATAAGATCAAAAAGGAGTGCTAAATGAGCCGAGGTTCGAGTTTCTTGATGAATGCTGCCTTCAGTTTTCTCAAGAAAAAGGAAAAAAAGCTCGGAGTTTATTATTACCCTTTGTTTAGAAGTTGAAAACAGTCTCCAATCCTGATATTAGTGTTTCTAATAATTCTGTTAAGGCTTTGCAGGGAAAGGACCAAAGCCCA

>cucumber_newGene_558 cucumber_newGene_558.1

CTTTAATCAGCTAAACGGGAACAACGATTGAAGCAGGTCAGCATATGCATTCGGAACACCAGTAACCAACCGGCGTCCACAAGCTCGAATTGTACTCAACTTAAGGCTGAAGGATTCAAATTCATGGCTTTAACCAATTTTATCTTGACGGTGGCCGGCGTTAGCGCCGTGGTTCTTCTTTTGAGGAGCGATGTGAAGCAATCGGCTTCAATCTTCAGGCGCAACGTCAAGCACATTCGCAAGTGGCTTGAAGAGGAATCCAAGGCTGTGGAGAACACAACCAAGGAGCTGGAGTCCAAGACTTCTCAGAAAGATATTCCCAAGGATGACAAGCACTAATGTCAATATTGGTTATTTAGCAATGTAAATCTTTTATTAAGTCAATCTCTCCCCACTCACCCAAGTTTTGTTGTCCATTTCACAACACAAAAAAGTCTCTTTACTTTTTACTGTTCCTTTCCATCCCAGTTTAGTATCTGATCCCTTTGACCAAGATGAAAAATAATGACCACTTTTGGTTACATAGAAACCCTAGTTTGTTAAACAAGATATCTTTAAATCCTTA

>cucumber_newGene_562 cucumber_newGene_562.2

AAAACATGGCTTCTCTTCTTTTCTTACTCCTGGGATGTTGTTTTAGGGAGAGAAGAAGAGATGTTCGTGACTAAAACCGTAATCCTAGTAGGGGTTGTACTTGTAAATCCTTGTTTCATCTCTTGTTAGATCCTGCCCCCTATAGAGTTAGTTTTTTACGTGGTTTGGAGGACTGATATTTCTAAGAAAGTTAGATTTACTTATTTAACTAGTGTTGCTGGGTCGGGTTAACATTGTTGATAGGCTTGGTTGGATAAGAACTTCGCTTGATGTGTCTTTTTGTTGCTTGCTTTGTCCGAAGGTGGAGGAAATCATCTCTTTTGGGATTGCCAATTTGCTTGGACTGTGCAAAGCTCTTTCTTTCAAGAGCTAGAGTGTTAGAGTGATGATCGAGGAATTCCTCCTCCATCTACCCTTGAAAGATACAGGGGGTTTTCTTTAGCTTGCCGGGGTGTGTGCGGCTATGTGGGACATATTGGGGAGAGGAATTTTAGGATAGAGAGAGGATGCATAGTGAGATTTGGTCTTTGGTTAGATTTTACGTGTATTTTTAGGTTTCCATTTTGAAGAGCTTTTGTAATTATTCACTTGGAAACATTTTGGTTAGTGGGTGACCTTTCTTTTAACAGGAGCTTGCTGGTTGTTTTCTTTAATTGTTTTCCATTAGATGGAATATATTGGTTTTGTTCCTCCTCAATTTTCGGCTATATTGTGTCTTCTGAGATGAAATGAATCGGATGAAACATTTGAGGCCTGTTTATTGGGGATTGAGACTAAAAACAAGCGATTTAAAGAACACAAAATGTGTTTGGTTTGGTAGCAATTCAAAAATTTCATTCGAACTTAAAAAATTGTTTTACATGTTTTCAATACCACTTTCCCTTTTGTATTCATTACATATCCCAAAGCCAACAACAATCTTTTTGTTCAGATTGACCCGGTGAAATACAAGAACATTTCATCTGGTTTTGGAGTTTTGCTAAAGGAGCAAGGAATCAGGGGCCTCTTTAGGGGTTGGGCGCCAACTCTACTTGGTTACAGTGCTCAAGGTGCTTGCAAGTATGGCATCTACGAATTCTTTAAGAAATACTATTCCGATATAGTGGGTCCTGAGTATGCAGCAAAGTACAAGACATTGATCTATCTTGCTGGTTCTGCGTCCGCTGAAGTGATTGCTGATGTTGCACTTTGCCCCTTTGAAGCTGTTAAAGTTAGAGTTCAGACACAGCCAGGTTTTGGCAGAGGTTTGTCAGATGGCCTTCCTAAATTTGTTAGATCTGAAGGTGCTCTTGGGTTGTACAAGGGTATTGTTCCTCTGTGGGGACGTCAAATCCCAGATACAATGATGAAGTTTGCAACATTTGAGAACTTGGTGGAACTTATCTACAAGCATGCAATCACGAAGCCAAAGAATGAATGCAGCAGCGCATTGCAGCTTGGAGTCAGCTTTGCTGGTGGATATATTGCGGGTGTGTCTTGTGCTATAGTTTCTCATCCTGCGGATAATCTCGTCTCCTTTCTCAATAATGCTAAAGGGGCTACCGTTGGCGAGGCAATTCAGAAACTTGGATTATGGGGTCTCTTCACACGAGGGCTGCCTCTACGCATAGTCATGATTGGTACCCTAACTGGAGCTCAATGGGTTATCTACGACGCCTTCAAAGTTTCTGTTGGACTGCCAACAACGGGTGGGGTTGCTCCTCCCATTGCTGCATCCCCAGAACATACAACTGCAACTGCGTAGGCTATCCAATCATTGTCACTACTTAACACGTTTTTTTTACTACAAAATTTACAAGAAAACTCTTGCTGGAAAAATATGTAAAATCAATAAATATAAATTTATAATATTGGAAAAGGATAGTTGAGAAAATTAGGTAGATATGGGCAGAGGATATGAATTACTCCACACCCTTGACCTCTTAAAGCGATCGAATGAGGCTTTATATTTTAGGTCATGACATGGCTCTGTTCTTCACCAAATATGCG

>cucumber_newGene_563 cucumber_newGene_563.1

TGAAGTTCTCAAGAGTTGTAGTCTAAATGGCCAATGCAAACAATATTACTTTCAGGTTGGTTTTCTTGTTTTCATTTTTGTTTCTCTAAACCTTTACTTGTTCAAATTATTTGGTACCAGAGAGTTTCTTTTTCTTTCTTTAACAAGTATGTTTTCTGACTTCTAACAATTACACAGATAAGGATTTCGATCTTACGCCTCAATTAGAATATAGAAAGAAATAGAGACATGTGTTGAACATGGACTCGTTTCAATTGAACTCGGAAAGTTTGTGAACTGAACTCCTTTCTTTCTCCATGGTTGGAGATGAACTCGTGTAGCTATATGATTTACATACAGGACAAGCTTCGAAGATCAACAACACACAAAATTTGTTCTACGACTTGGGTTTCTCCTTTCATCTATGATAGCTGTCTTCCTCCATGTTTTGTTCTTCTACATCTACAACATAGATTATCAGAAAAGTCAAGAACGCGACCATTGGTGCCAGGTCGGTGACGGACTTCATCCTTCTACTCGAAGTCGCCTACAAGGTCTGCTCAAGTTCAATACTTGACTTATGTCCCAATTCTAAAACGAATGCGGCTCCTCTGACTTTCTTCGGACGCCTTGCTGATGTTAGCAAAAGGGTTCCATGGATGGATGTCCTTGTCGATTTTTTGGCAATGCTTCCCCTTCCACAGATTATCCTTGTGGGAGTCTTTCCAAGTAGGAAAGGTATCAAATATTTTAAAAACAAGTGGCGACTGATTTTTCTGCCTCTCTTCCAATTTGTCCCAAGAATCATCCG

>cucumber_newGene_564 cucumber_newGene_564.1

CTTGCTTTTAATAAACAGTTCTCTTCCAGTTGGCTTGATCACTCAATAGTTCTTGGATTAAACTTCAATTTTAGAAACACTAAGCACAATCTTTGGAGCTTGATTGCTTCAACATTGTTGATTAAATCTTTAACATCCTTCAAGGTGATGGCGCGGATGTTTGGATCTTTGATGTTGATCATACTCTTTTGTCCAACATCCCTCTCTATAAGAAGCATGCTTTCAGCTTTTCAAAACAGTTTGGAGGTTAAGACCAAACGTTCAAGTTTTAAGAACATTGAAGGAGCATTGAACTCATACTGAGTTTTGGGTAGTCTCATGTATAAAACAAGGGAGAAAAGTTAAATGAAACAGCTAAAGAGGAGCGGATGAAAAGCAGCAAAGCACCAGCTCTTGAGTATACATTCAAACTCTTCAATCTTATTAAACACATAGTGATTCAAATCTTTTTTACTTCTTCAACAAGGGAAGAACTTAAATATAATCTCATTCATTCAAGTTAGGATACCATGGATGGTCTTCACTCAC

>cucumber_newGene_565 cucumber_newGene_565.1

AAAAACTACAAATAATATGTTTAATACAAGTACATTAAAATTAAAAAAACCTACACCTATCTCATAATACTAGTCCCATACACCAAATAAAGAATGTGAAATAAAGTACAGTTAGAATGTGTACAGATGCAAAATTAAACAAAATTAAATATTGAAAAGTATGCACCCAAGTGTTCACAACTTTCTCTGTGCTCAACTCATCTCCTTGATCATCTTCTTCATCTCCTCCTTTTGTCGTGCATGGTCTGCTATTTGTCGGTCTCGCTCCTCCTTCCTTAGTCGTTGTGCTCCAATATTCGGTTAACATTTTCATGGCTGGTCTTTAGTTCGCTAACCTCTATCATGTGTGCCTGGTGGTCATAATTATTGAAGGAAGAAGTGAAAGCATAACTCTTTTGGACTTGGGGTTGGAGTCCCACCAAGACCTTTTGAGTAGCCTGGTTGTCTACCTAAAAGGGTCTACATATCTCGTCCTCAGAGAGTGGTTGAGAACCCTCTAGAACGGGCTGGGACTGAGGTTCCATCATTTGATTATACACAAATAGAAGTTTGTAAGTTGGGAGATGATTAGAATTAAAATAGAATTGAAAGTATAACAAAAGTTTTCATGAACTTACAAAGAGCATCTTCTACTGTGAAATGAACTCATCAGTCCTACTGGCATGCGTTTCTCGAAACAACTTGACACGATTAACTGGATGACTGCGTTCTTCAATAAGCTCGATCTATCTTTGAATGAACGACTTTCTGCAGCTTCTATGGTTGTAAGGTAGATATTTACTTTCTATCACAAATTAAATGCAGCCTTGCAAATATTTGATTGCTCACTCATACATTCAAATAATGGTGATCTTAACGTTATATTCAAACAACAAATTAAATGAAGTGTTGTTAATTGATTTTAAAGTAGACGATATAAATCACCTGAAACTGTCGAGTTATGTAATAGTCACAAAGAAAGTGCCAATTTTGCACGCGATTGGCGAATCTTGGCTGCGGATTTGCACATACTTCTTTATGGTTGTTGTACTTCTTAAAATATCGGTGGTTATCTCCCCTGAATTTCTTCCACACGCTGAGCATTTGATGCTCAACAAACCGATTAAGTGTTGAAACTTCGAGATTGAGCATGAAAAATTGCTACAAAAGAAATAGGTTTGAGATTAGTCTTCTACATATGCAGGTAATTGAACATACATATATGTTAAAAATTTTACAAACTTATCGCTAAGCCTCTCTTGACAACCTTGATGTACTCTCGAGGTACATCAACCTACTTTAGAACTTGTATCAGAAACGTGTCATGCGTTAACACACCGATCGTGCAGCTGAAACGAGGGGCGTATGGTGAGACAAGCTTGTCCCCTCATTGGGGGATTGAAATGGGGATCCTTCCATTCTGTTGGACATACCTCTCCAACTCAATGTTTCGAGAGTGCTGTTGACTTCTCATAGTGGGAGTGGGAGAGGGTTGAGAGGGATCTACTGAAAAACATAAATTATTAGATCGATCCAGTAACCATAACTACAAGTGTTTAAGGTAACGTGACTAACCTGGATTGTTGCCCATCGAAGACGATCCTCCCGCGTTATCTAACGCATCCAAGTTTAGGTAAATGTTTGCCTCATTAATATCACTAAGAAATGAAGTCATAATTCTTGCAGACATAAAAACCAACAAACGTTAAACAAATGTGAGTATCGAAAACTAAATGTATAGAAAAATGACTAACATATATACATGAAGAAGATATTATTTTTTGATGTGATGATGATTGTTTGTCATCATCGTCGTCTATGAAGTTGTCCGCGAAATGACATGTAAAATCGTATGCCACCACTAATGCATTCACTGTAAGAGCGAACATGAACTGAAGGTCCCATTGCGAGTGAGAAGAAATCTTGAGAAATGTTT

>cucumber_newGene_566 cucumber_newGene_566.1

TGTTTGAACAGCTTCATAGCAAGAAACGAAATAGGCTTGCTCAAAGTTGTTTGAATGATCTAGTGTTCATCAAATACAATAGAGCATTAAAACGTCGATACAACCTACGAGATATTGTCGACCCCATCTCCTTGAAAGATATTGATGATAGTAACGAATGGTTGATTGGAAGATTGGATGACGATTCTGAGGAGGAGGATGAGTTGGTATTTGACGATGATTCTTTAACGTGGGGTGGTGTTTCAATAGTTGCCGGAGCAAAAGAACCATCATTCTATTCTAGAGCTAGTACTAAAAGAGCAAAGACTAATGTTTCATGTTCATCCTCGTCTACCA

>cucumber_newGene_571 cucumber_newGene_571.1
[truncated: 635,779 more chars]
